# DNER drives glycolytic reprogramming in renal cell carcinoma by activating the JAK2/STAT3 signaling pathway

**DOI:** 10.3389/fimmu.2026.1799104

**Published:** 2026-05-22

**Authors:** Anrui Li, Jing-wen Xu, Jian-hua Qin, Qi Yuan, Lichen Teng

**Affiliations:** 1 Department of Urology, Harbin Medical University Cancer Hospital, Harbin, Heilongjiang, China; 2 The Harbin Institute of Technology (HIT) Center for Life Sciences, School of Life Science and Technology, Harbin Institute of Technology, Harbin, Heilongjiang, China; 3 College of Life Science, Mudanjiang Medical University, Mudanjiang, Heilongjiang, China

**Keywords:** clear cell renal cell carcinoma, DNER, glycolysis, metabolic reprogramming, olaparib, tumor microenvironment

## Abstract

**Introduction:**

Clear cell renal cell carcinoma (ccRCC) remains a clinically challenging malignancy due to late diagnosis and limited therapeutic options. This study aimed to investigate the role of DNER in the progression of ccRCC.

**Methods:**

Bioinformatic analyses were integrated with experimental validation. Metabolism-related candidate genes were systematically screened to identify hub genes closely associated with metabolic pathway activity and immune cell infiltration. Both in vitro and in vivo functional assays were performed, along with mechanistic studies focusing on DNER-interacting proteins and downstream signaling pathways.

**Results:**

Bioinformatic analysis revealed that lipid metabolism and energy metabolism are key metabolic pathways significantly affecting the prognosis of ccRCC patients. Through systematic screening, DNER was identified as a hub gene strongly correlated with both metabolic pathway activity and immune cell infiltration. Functional experiments demonstrated that DNER promotes ccRCC cell proliferation both in vitro and in vivo. Mechanistically, DNER physically interacts with JAK2 and activates the JAK2/STAT3 signaling pathway, leading to STAT3 nuclear translocation and subsequent direct transcriptional upregulation of the glycolytic enzymes LDHA and PKM, thereby enhancing glycolytic flux. The increased lactate production drives macrophage polarization toward a pro-tumorigenic M2-like phenotype, establishing a signaling cascade that links tumor cell-intrinsic metabolic reprogramming to immunosuppressive microenvironment remodeling. Preliminary evidence also suggests that DNER overexpression may be associated with enhanced sensitivity of ccRCC cells to the PARP inhibitor Olaparib, although the underlying mechanism requires further investigation.

**Discussion:**

In conclusion, DNER drives ccRCC progression by coupling glycolytic reprogramming with immunosuppressive microenvironment formation via the JAK2/STAT3 signaling axis, and may represent a potential therapeutic target for ccRCC.

## Graphical Abstract

**Figure:**
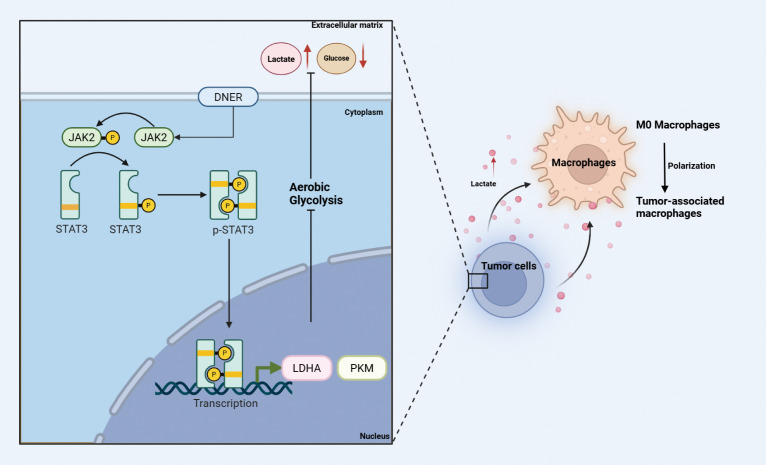

## 1 Introduction

Clear cell renal cell carcinoma (ccRCC), a malignancy arising from the renal tubular epithelium, represents the second most prevalent urological cancer worldwide (1–3). Current epidemiological statistics indicate that ccRCC accounts for approximately 2% of all newly diagnosed cancer cases globally, with an annual incidence exceeding 300,000 and nearly 134,000 related deaths, highlighting its substantial and persistent disease burden (2, 4). The often asymptomatic nature of early-stage disease results in frequent diagnosis at advanced stages with local invasion or distant metastasis, contributing to a dismal 5-year survival rate of around 15% (5, 6). Despite advances in targeted therapies, including tyrosine kinase inhibitors, and immunotherapeutic strategies for advanced ccRCC (4, 7), overall mortality rates remain largely unimproved, and conventional radiotherapy and chemotherapy demonstrate limited efficacy.

In recent years, the rapid advancement of high-throughput sequencing technologies—particularly the widespread application of transcriptomic sequencing (RNA-seq) and single-cell sequencing—has provided unprecedented tools for systematically identifying key pathogenic genes and potential therapeutic targets in ccRCC (8–10). The value of these data-driven strategies extends beyond initial candidate discovery; By integrating differentially expressed genes with detailed clinicopathological features and immune infiltration profiles, it is possible to systematically identify candidate molecules that may functionally contribute to tumor progression and immune microenvironment remodeling, thereby providing a robust data foundation for subsequent mechanistic investigation (11). Collectively, these insights provide a robust data foundation and theoretical framework for subsequent functional validation and mechanistic investigation.

Delta/Notch-like epidermal growth factor-related receptor (DNER) is a transmembrane protein originally identified in cerebellar Purkinje neurons (12, 13). As an atypical member of the Notch ligand family, it binds to and activates the Notch1 receptor, thereby modulating Notch signaling (14). Beyond the nervous system, DNER is also expressed in adult pancreatic islet cells, peripheral blood, and early pancreatic progenitor cells, where it has been implicated in the pathogenesis of type 2 diabetes (13, 15). Emerging evidence further indicates that DNER participates in the malignant progression of various cancers. For instance, studies in prostate cancer, hepatocellular carcinoma, and breast cancer have linked DNER to enhanced tumor cell proliferation, migration, and invasion (14, 16–19). Nevertheless, the expression profile, biological roles, and underlying molecular mechanisms of DNER in ccRCC remain largely unexplored and warrant further investigation.

ccRCC is distinguished from other renal malignancies by its unique metabolic phenotype, characterized by abundant cytoplasmic lipid and glycogen accumulation coupled with a constitutively activated glycolytic program driven by VHL deficiency (20, 21). This profound metabolic dependency not only sustains tumor growth but also fosters the establishment of an immunosuppressive tumor microenvironment. Given that DNER is a transmembrane protein structurally poised to transduce extracellular signals to intracellular effectors, we reasoned that DNER may serve as a critical node linking metabolic dysregulation to downstream oncogenic signaling in ccRCC. In support of this hypothesis, our preliminary bioinformatic survey of the TCGA-KIRC cohort revealed that DNER expression is strongly correlated with the activity of both glycolytic and lipid metabolism pathways, as well as significantly associated with immune cell infiltration patterns. However, whether DNER directly participates in ccRCC metabolic reprogramming and immune modulation, and through which downstream signaling mechanisms it exerts such functions, remain entirely unexplored.

Metabolic reprogramming, particularly through the “Warburg effect” or aerobic glycolysis, represents a hallmark of cancer cell biology and plays an important role in tumorigenesis and progression (22–24). This metabolic shift is characterized by tumor cells preferentially converting glucose to lactate via glycolysis even in the presence of adequate oxygen, a process that yields limited ATP but supports rapid biosynthesis and proliferation (25, 26). The reliance on glycolysis enables cancer cells to efficiently uptake glucose and generate metabolic intermediates essential for macromolecule synthesis, thus sustaining their accelerated growth (27, 28).

Growing evidence underscores the association between metabolic dysregulation and the development of renal tumors, especially in ccRCC. Aerobic glycolysis is largely driven by the activation of the VHL/HIF signaling pathway, which transcriptionally upregulates key glycolytic enzymes and transporters, including glucose transporter 1 (GLUT1), phosphoglycerate kinase 1 (PGK1), and pyruvate dehydrogenase kinase 1 (PDK1) in RCC (29–33). Given the central role of metabolic adaptation in ccRCC pathogenesis, elucidating the functional contributions of metabolism-related molecules is critical for deepening our understanding of the disease and advancing the development of novel therapeutic strategies.

## 2 Methods

Additional details are provided in **Supplementary Materials** and methods

### 2.1 Data sources and acquisition

This study obtained the public data of the renal clear cell carcinoma (KIRC) cohort from the Cancer Genome Atlas (TCGA) database. Through the GDC data portal (https://portal.gdc.cancer.gov/), the RNA sequencing gene expression data (including tumor and normal samples), detailed clinical survival information, and somatic mutation data of the “TCGA-KIRC” project were downloaded. We screened the primary tumor tissues based on the TCGA sample type codes and cleaned and integrated the clinical data, eliminating samples with missing key information. Finally, a unified dataset for subsequent analysis was constructed. All data have been de-identified and their use complies with the TCGA data access policy.

### 2.2 Functional enrichment analysis

To clarify the biological processes, molecular functions, and related signaling pathways that the target gene set is involved in, we conducted functional annotation and enrichment analysis using the DAVID bioinformatics resource. The specific steps are as follows: First, the official gene symbol list of the target genes was uploaded to the DAVID online analysis platform. The analysis species was set as “Homo sapiens”. We extracted all significantly enriched GO terms and KEGG pathways from the DAVID report, including their enrichment scores, gene proportions, and the list of key genes included, for subsequent biological function interpretation.

### 2.3 Western blot analysis

Western blotting was performed as previously described with slight modifications (34). Briefly, proteins were extracted using RIPA lysis buffer, separated by SDS-PAGE, and transferred to PVDF membranes. After blocking with 5% non-fat milk in TBST for 1 hour at room temperature, membranes were incubated overnight at 4 °C with primary antibodies, followed by incubation with HRP-conjugated secondary antibody for 55 minutes. Protein bands were visualized by enhanced chemiluminescence and quantified using ImageJ software.

### 2.4 Mouse xenograft tumor model

This study was conducted in accordance with the ARRIVE guidelines. All experimental procedures involving mice were approved by the Animal Ethics Committee of Mudanjiang Medical University (Approval No. IACUC20241015-781). Male BALB/c mice (4–6 weeks old) were used to establish xenograft tumor models. Mice were subcutaneously injected with 5 × 10^5^ RENCA cells transduced with either OV-Vector or OV-DNER into the ventral flank. When tumor volumes reached approximately 100 mm³, mice bearing DNER-overexpressing tumors were randomly assigned into groups (n = 5 per group). Treatment consisted of intraperitoneal injection of Olaparib (20 mg/kg) administered for 10 days in a discontinuous schedule: 5 consecutive days of treatment followed by a 2-day interval, for two cycles. Control animals received an equal volume of phosphate-buffered saline (PBS) via the same route. Tumor dimensions were measured every two days, and tumor volumes were calculated using the formula: Volume = (length × width²)/2. A total of 15 male BALB/c mice were randomly allocated into three experimental groups (n = 5 per group). At the experimental endpoint, all animals were euthanized by intraperitoneal injection of an overdose of Fatal Plus pentobarbital solution (86.6 mg/mL).

### 2.5 Chromatin immunoprecipitation assay

Chromatin immunoprecipitation (ChIP) was carried out following a previously established protocol with minor modifications (35). HEK293T cells (4 × 10^7^) were crosslinked with 0.75% formaldehyde and quenched with glycine. Chromatin was sonicated (30% amplitude, 10 cycles) to 200–1000 bp, incubated overnight with antibody-coated Protein A/G beads, washed, and reversed crosslinked with Proteinase K/RNase. DNA was purified and analyzed by qPCR; enrichment was normalized to Input.

### 2.6 Flow cytometry

THP-1 cells treated with conditioned medium were stained with fluorescence-conjugated antibodies against CD86 (FITC; United Biotech) and CD206 (PE; BioLegend) at 0.2 μg/mL each. After washing, cells were resuspended in PBS containing 2% FBS. Flow cytometry was performed on a FACSCalibur instrument (BD Biosciences) and data were analyzed with FlowJo software (TreeStar).

### 2.7 Cell lines and culture

The ACHN, 786-O, and HEK293T cell lines were obtained from the American Type Culture Collection (ATCC). THP-1, A496, and RENCA cells were procured from the Cell Bank of the Shanghai Institute of Biochemistry and Cell Biology, Chinese Academy of Sciences. All cells were cultured in high-glucose Dulbecco’s Modified Eagle Medium (DMEM) supplemented with penicillin (100 U/mL), streptomycin (0.1 mg/mL), and 10% fetal bovine serum (FBS). Cells were maintained in a humidified incubator at 37 °C under 5% CO_2_.

### 2.8 Quantitative real-time PCR

Total RNA was extracted from cells using a commercial RNA extraction kit (Vazyme, cat# RC13) according to the manufacturer’s protocol. Complementary DNA (cDNA) was synthesized using a reverse transcription kit (Vazyme, cat# R323) as instructed. Quantitative real-time PCR was performed on an Applied Biosystems real-time PCR system with ChamQ Universal SYBR qPCR master mix (Vazyme, cat# Q711-02). All primer sequences used in this study are listed in **Supplementary Table 5**.

### 2.9 Establishment of stable cell lines

Plasmids for the overexpression of human DNER (P44636), human STAT3 (P79684), human LDHA (P88825), and mouse DNER (P85208) were obtained from the MiaoLing Plasmid Platform. Control plasmids pLV3-CMV-MCS (P40122) and pLV2-U6-MCS-shRNA (P62081) were also sourced from MiaoLing. A non-targeting shRNA plasmid (1864) was purchased from Addgene. The following pLV3-U6-based shRNA plasmids targeting human genes were acquired from MiaoLing: pLV3-U6-DNER(human)-shRNA1 (P81589) and pLV3-U6-DNER(human)-shRNA2 (P81598).

### 2.10 Statistical analysis

Animals were randomly assigned to experimental groups, and tumor volume assessments were performed in a blinded manner. All data are presented as mean ± standard deviation (SD) and were analyzed using GraphPad Prism (version 9.0). The number of biological replicates and independent experimental repeats are indicated in the respective figure legends. Normality and homogeneity of variances were assessed using the Shapiro–Wilk test and Levene’s test, respectively. Parametric tests (Student’s *t*-test or one-way ANOVA followed by Tukey’s HSD *post hoc* test) were applied when both assumptions were met; otherwise, non-parametric alternatives (Mann–Whitney U test for two-group comparisons or Kruskal–Wallis test for multi-group comparisons) were used. For animal experiments, a sample size of five mice per group was determined by *a priori* power analysis based on previously reported tumor volume variability in ccRCC xenograft models (>80% power, α = 0.05). All statistical tests were two-sided, and *p* < 0.05 was considered statistically significant.

## 3 Result

### 3.1 Identification of key metabolic pathways in KIRC patients

**Figure 1:**
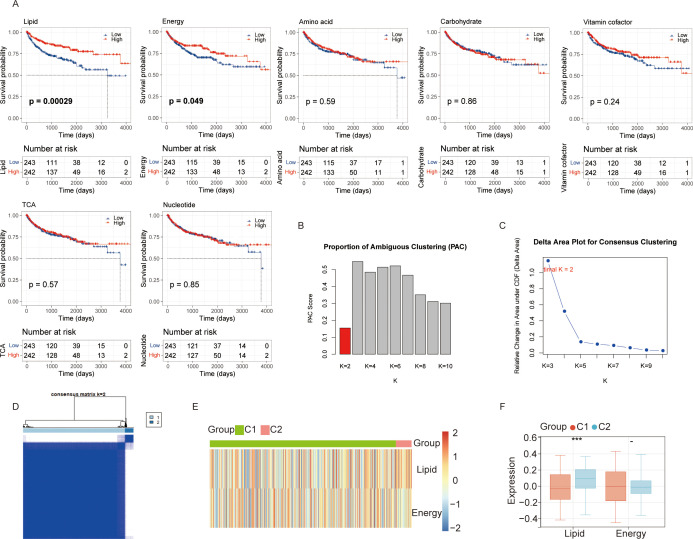
Identification of key metabolic pathways in kidney renal clear cell carcinoma (KIRC). **(A)** Kaplan-Meier curves depicting overall survival differences between high and low pathway activity groups for seven metabolic pathways in the TCGA-KIRC dataset. Red represents the high-activity group; blue represents the low-activity group. **(B)** Proportion of Ambiguous Clustering (PAC) curve evaluating the stability of consensus clustering based on lipid and energy metabolism pathways. **(C)** Delta area plot illustrating the relative change in the area under the cumulative distribution function (CDF) curve across different cluster numbers (*k* = 2–9), supporting the optimal number of clusters. **(D)** Consensus matrix heatmap for *k* = 2 based on two key metabolic pathways in the TCGA-KIRC dataset, showing clear separation of KIRC patients into two distinct clusters (C1 and C2). **(E)** Heatmap comparing the enrichment scores of the two key metabolic pathways between Cluster C1 and Cluster C2. **(F)** Box plots showing the differential activity of the two key metabolic pathways between C1 and C2. ns, not significant; *p < 0.05, **p < 0.01, ***p < 0.001.

Metabolic reprogramming reflects the adaptation of tumor cells to meet the demands of aberrant proliferation, and metabolic heterogeneity profoundly influences both therapeutic response and patient prognosis. We calculated GSVA scores for seven metabolic pathways in KIRC patients and stratified patients into high- and low-score subgroups based on the median GSVA score for each pathway. Only lipid metabolism and energy metabolism exhibited significant differences in overall survival; the K-M analysis for the remaining five pathways did not reach statistical significance (all *p* > 0.05, **Figure 1A**). Given the histopathological hallmark of KIRC as a clear cell carcinoma characterized by abundant cytoplasmic lipid and glycogen accumulation (36), we focused subsequent analyses on the lipid metabolism and energy metabolism pathways.

To systematically evaluate the metabolic patterns of KIRC patients, we performed unsupervised cluster analysis based on lipid and energy metabolic pathways (**Figures 1B, C**). As shown in **Figure 1D**, KIRC patients can be clearly divided into two distinct clusters (C1 and C2). The lipid metabolism level of cluster C2 is higher than that of cluster C1. However, there was no significant difference in the energy metabolism levels between cluster C1 and cluster C2, which might be related to the limitation of sample size (**Figures 1E, F**).

### 3.2 Identification of candidate genes related to lipid and energy metabolism in KIRC patients

**Figure 2:**
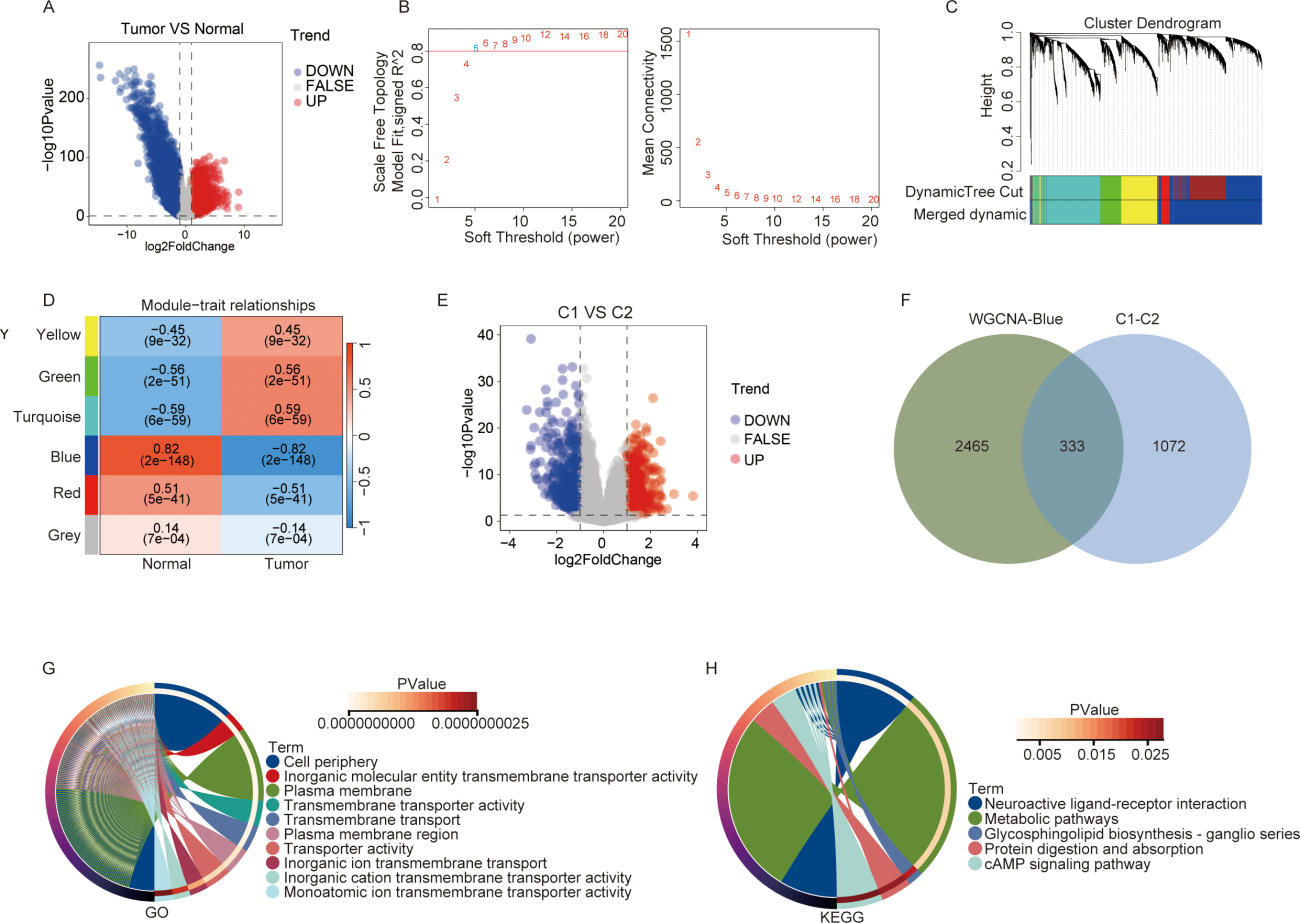
Identification of hub genes in kidney renal clear cell carcinoma (KIRC). **(A)** Volcano plots of differentially expressed genes (DEGs) between tumor and normal samples in the TCGA-KIRC dataset. **(B-D)** WGCNA was performed by DEG between tumor and normal samples. **(B)** Scale-free fitting index analysis of soft-thresholding powers. **(C)** Cluster dendrogram. **(D)** Module-trait correlation heatmap. **(E)** Volcano plots of DEGs between C1 and C2 in the TCGA-KIRC dataset. **(F)** Venn plot of the WGCNA blue module and the DEGs between C1 and C2. **(G, H)** GO enrichment analysis **(G)** and KEGG enrichment analysis **(H)** of intersection gene. ns, not significant; *p < 0.05, **p < 0.01, ***p < 0.001.

To identify candidate genes associated with lipid and energy metabolism, we first performed differential expression analysis between tumor and normal samples in the TCGA-KIRC dataset. A total of 6,592 differentially expressed genes (DEGs) were identified, comprising 3,555 up-regulated and 3,037 down-regulated genes (**Figure 2A**; **Supplementary Table 1**). The DEGs were subsequently subjected to WGCNA analysis. A soft-thresholding power of β = 5 was selected to construct a scale-free network (**Figure 2B**), and hierarchical clustering was performed to group genes with high co-expression similarity into distinct modules (**Figure 2C**). A module–trait relationship heatmap was generated based on Spearman correlation analysis (**Figure 2D**). Among all modules, the blue module exhibited the strongest correlation with KIRC traits and was therefore defined as the hub module, containing 2,789 genes potentially playing key regulatory roles in the initiation and progression of KIRC (**Supplementary Table 2**).

We also performed differential expression analysis between Cluster C1 and Cluster C2, yielding 1,405 DEGs, of which 710 were up-regulated and 695 down-regulated (**Figure 2E**; **Supplementary Table 3**). Intersecting the blue module genes with the DEGs between C1 and C2 produced a set of 333 candidate genes (**Figure 2F**). To further investigate the molecular mechanisms by which these candidate genes regulate KIRC progression, functional enrichment analysis was conducted (**Supplementary Table 4**). The top 10 enriched GO and KEGG pathways are presented in **Figures 2G, H**. GO analysis revealed that the enriched terms were predominantly associated with the cell periphery, inorganic molecular entity transmembrane transporter activity, and plasma membrane. KEGG analysis highlighted pathways mainly related to neuroactive ligand–receptor interaction, metabolic pathways, and glycosphingolipid biosynthesis–ganglio series.

### 3.3 Screening and immunological analysis of the hub gene DNER

**Figure 3:**
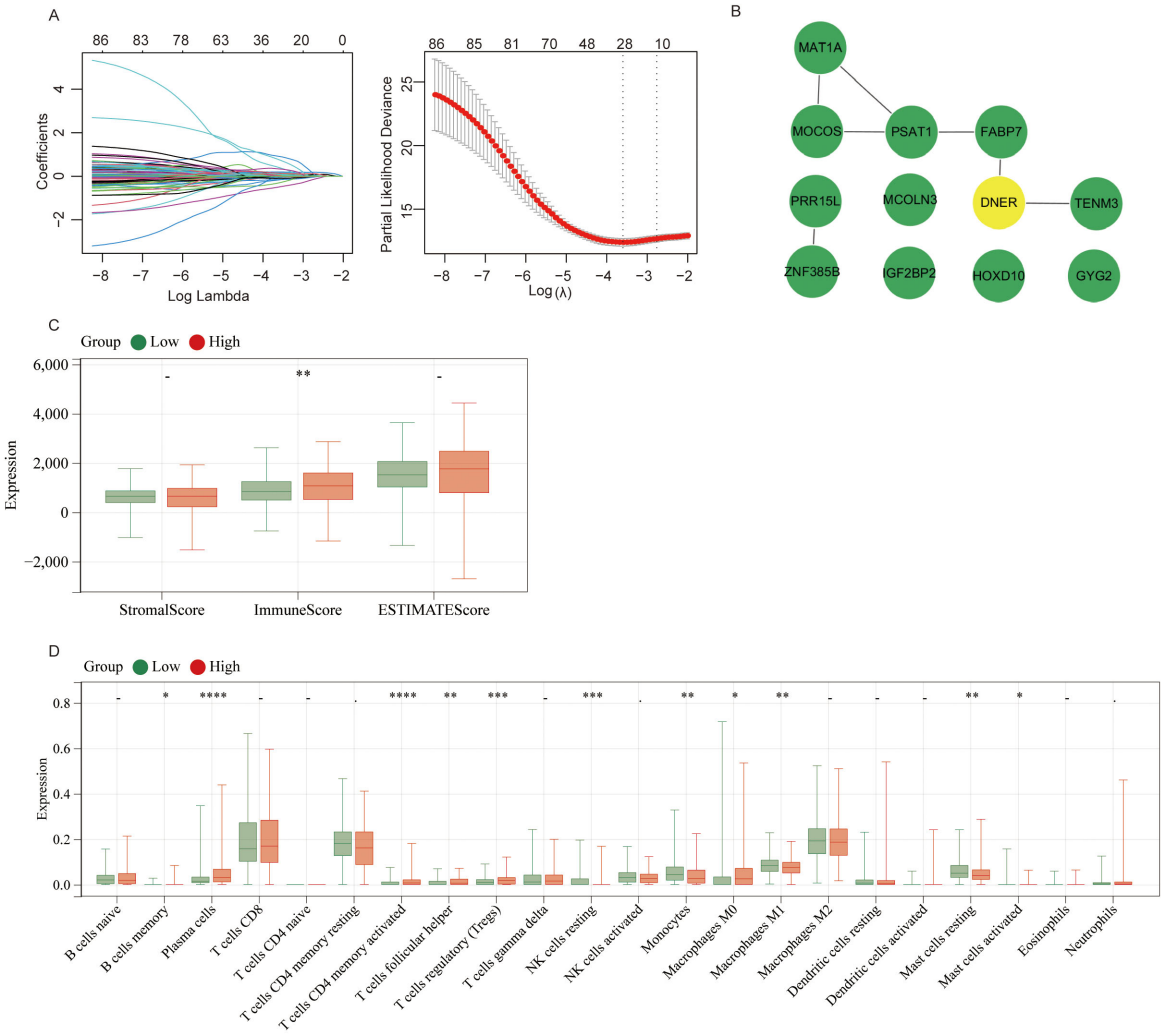
Screening and immunological analysis of the hub gene DNER. **(A)** The variation characteristics of variable coefficients and the selection process of the optimum value of the parameter λ in Lasso regression model by 10-fold cross-validation method. **(B)** Protein-protein interaction (PPI) network of the 12 key genes, with DNER identified as the core hub gene. **(C)** Comparison of ESTIMATE immune score, stromal score, and tumor purity between DNER high- and low-expression groups. **(D)** Differential infiltration of 22 immune cell subtypes between DNER high- and low-expression groups, with a significant difference observed in macrophages. ns, not significant; *p < 0.05, **p < 0.01, ***p < 0.001, **** p < 0.0001.

We performed univariate Cox regression analysis on the 333 intersecting genes and identified 89 prognostic genes significantly associated with overall survival. LASSO regression analysis further narrowed these down to 12 key genes (**Figure 3A**). Protein-protein interaction (PPI) network analysis was conducted on these 12 genes, and DNER was selected as the core gene for subsequent validation (**Figure 3B**). Immune-related analysis of DNER using the ESTIMATE algorithm revealed a significant difference in the immune score between the DNER high- and low-expression groups, whereas no significant differences were observed in the stromal score or tumor purity (**Figure 3C**). Following stratification of patients into high- and low-expression groups based on the median DNER expression level, analysis of 22 immune cell subtypes revealed a significant difference in macrophage infiltration between the two groups (**Figure 3D**).

### 3.4 DNER promotes RCC cell proliferation in an expression-dependent manner

We first examined endogenous DNER protein expression in three widely used RCC cell lines (A498, 786-O, and ACHN). DNER levels varied substantially across these models, with the lowest expression observed in 786-O cells and the highest in ACHN cells (**Supplementary Figure 1A**). To investigate whether DNER influences RCC cell proliferation, we engineered 786-O cells to stably overexpress DNER and ACHN cells to stably deplete DNER.

**Figure 4:**
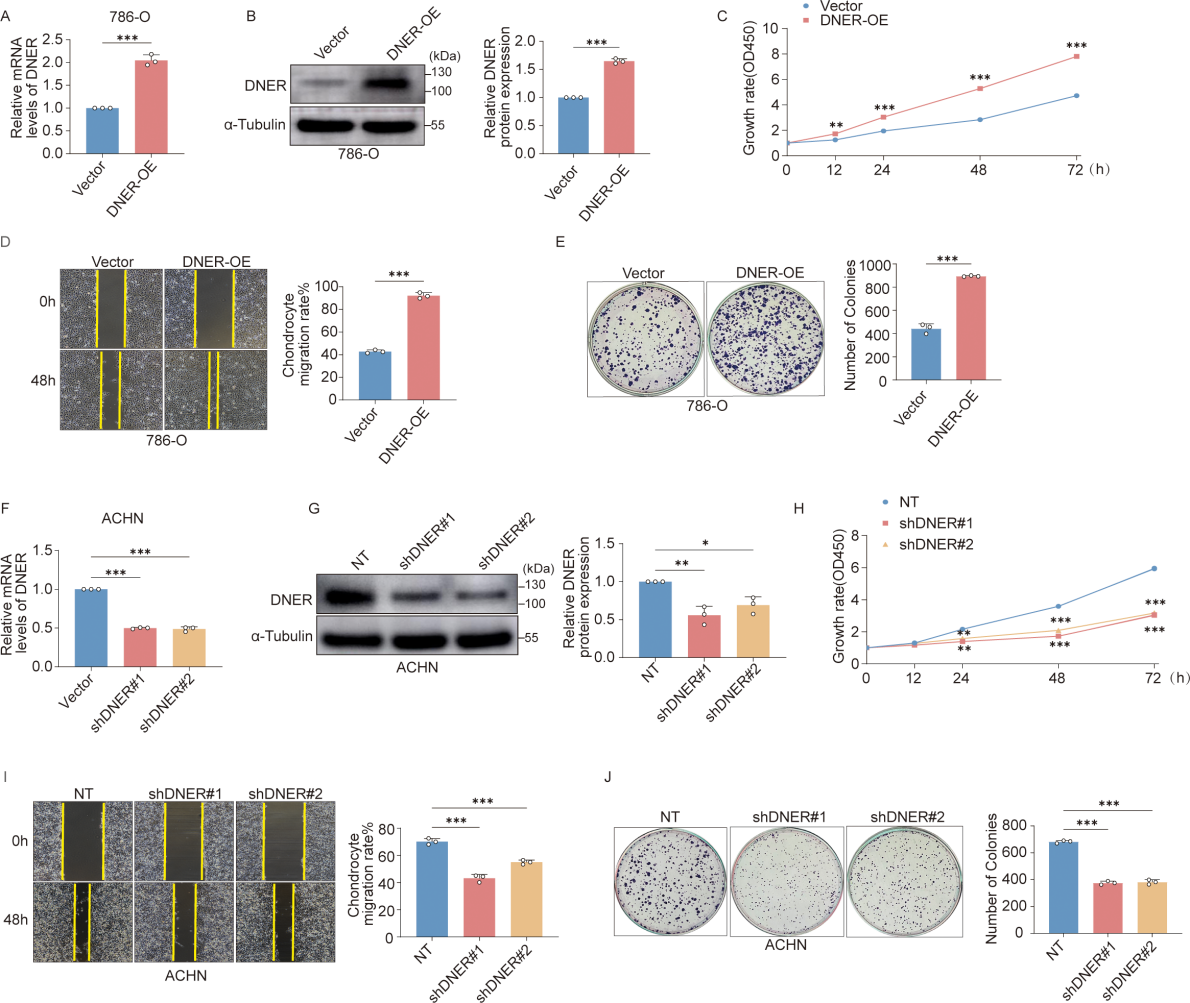
DNER promotes RCC cell proliferation. **(A, B)** Validation of DNER overexpression in 786−O cells by qRT−PCR **(A)** and Western blot **(B)**. **(C−E)** Functional assays assessing the proliferative capacity of DNER−overexpressing 786−O cells: CCK−8 assay for cell viability **(C)**, wound healing assay for migration **(D)**, and colony formation assay for clonogenicity **(E)**. **(F, G)** Validation of DNER knockdown in ACHN cells by qRT−PCR **(F)** and Western blot **(G)**. **(H–J)** Functional assays assessing the proliferative capacity of DNER−knockdown ACHN cells: CCK−8 assay for cell viability **(H)**, wound healing assay for migration **(I)**, and colony formation assay for clonogenicity **(J)**. Data are presented as mean ± SD (n = 3 independent experiments). *p* < 0.05, **p* < 0.01, ***p* < 0.001.

Stable overexpression of DNER in 786-O cells was confirmed by Western blotting (**Figures 4A, B**). Functional assays revealed that DNER overexpression significantly enhanced the proliferative and clonogenic capacity of RCC cells. Compared with control cells, DNER-overexpressing cells exhibited increased viability in CCK-8 assays, accelerated wound closure in scratch assays, and greater colony formation in clonogenic assays (**Figures 4C–E**).

Conversely, knockdown of DNER in ACHN cells (**Figures 4F, G**) produced the opposite phenotypic changes. DNER silencing led to a marked reduction in cell viability, delayed wound healing, and decreased colony formation capacity (**Figures 4H–J**). The enhanced proliferative activity observed upon DNER overexpression, together with its suppression following DNER knockdown, demonstrates that DNER plays a positive regulatory role in RCC cell growth.

### 3.5 DNER reprograms glycolysis via the JAK2/STAT3 signaling pathway in RCC cells

**Figure 5:**
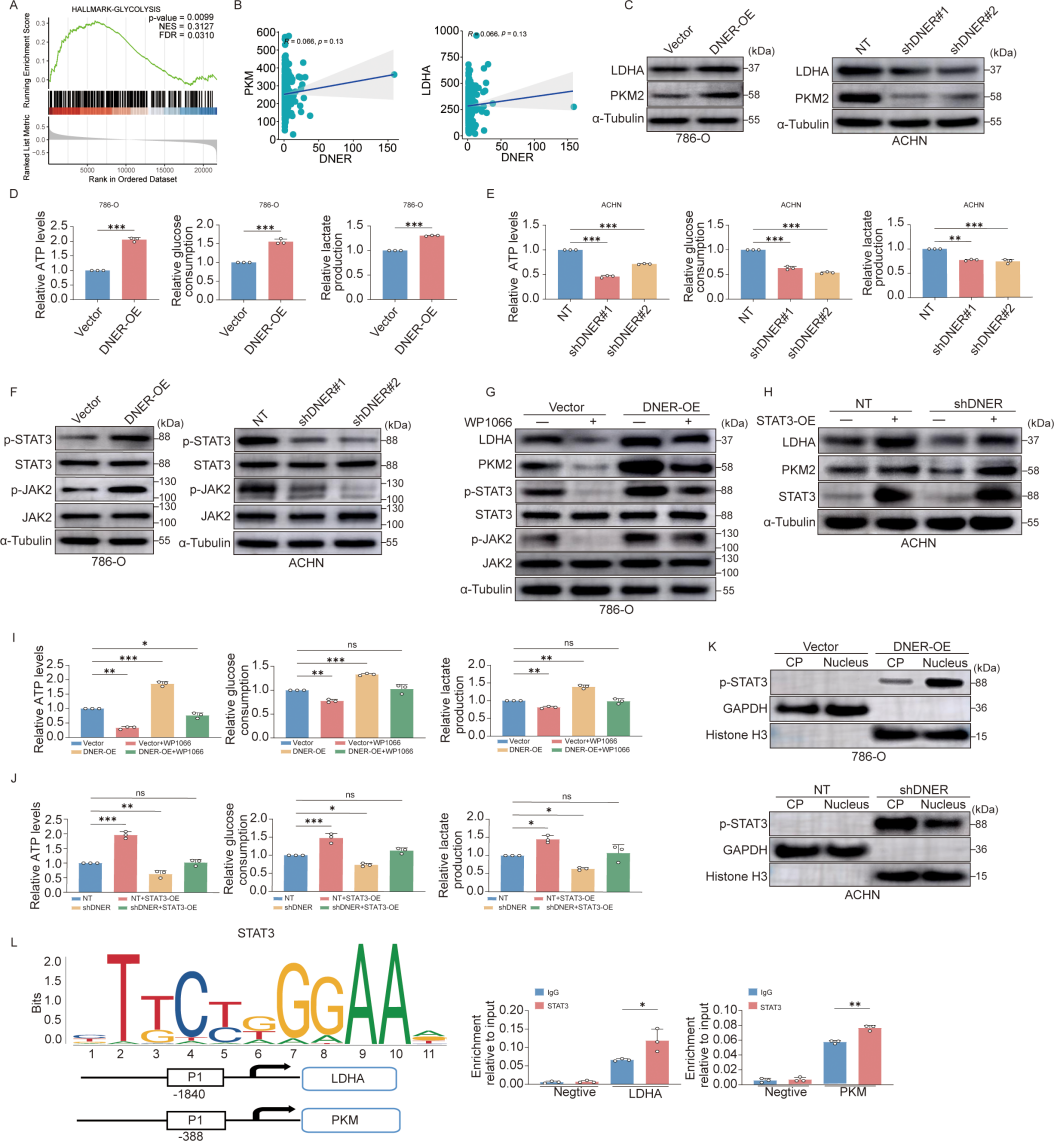
DNER reprograms glycolysis in RCC via the JAK2/STAT3 signaling pathway. **(A)** Gene set enrichment analysis (GSEA) plot showing significant enrichment of glycolysis−related pathways in DNER−high RCC samples. **(B)** Correlation between DNER mRNA expression and key glycolytic genes (LDHA, PKM) in RCC clinical specimens. **(C)** Western blot analysis of LDHA and PKM2 protein levels in control and DNER−overexpressing 786−O cells. **(D, E)** Metabolic analysis of ATP production, glucose consumption, and lactate secretion in control vs. DNER−overexpressing 786−O cells **(D)** and control vs. DNER−knockdown ACHN cells **(E)**. **(F)** Western blot analysis of total and phosphorylated JAK2 and STAT3 levels in DNER−overexpressing 786−O and DNER−knockdown ACHN cells. **(G, H)** Western blot analysis of LDHA and PKM2 expression in DNER−overexpressing 786−O cells treated with or without the JAK2/STAT3 inhibitor WP1066 **(G)**, and in DNER−knockdown ACHN cells with or without STAT3 overexpression **(H)**. **(I, J)** Metabolic analysis of ATP production, glucose consumption, and lactate secretion in DNER−overexpressing 786−O cells treated with or without 2.5 μM WP1066 **(I)**, and in DNER−knockdown ACHN cells with or without STAT3 overexpression **(J)**. **(K)** Subcellular localization of p−STAT3. Western blot analysis of p−STAT3 levels in nuclear and cytoplasmic fractions from DNER−overexpressing 786−O and DNER−knockdown ACHN cells. GAPDH and Histone H3 served as loading controls for cytoplasmic and nuclear fractions, respectively. **(L)** Schematic representation of predicted STAT3 binding motifs in the promoter regions of LDHA (−1840 bp) and PKM (−388 bp) genes relative to the transcription start site (TSS), as identified using the JASPAR database. Data are presented as mean ± SD (n = 3 independent experiments). *p* < 0.05, **p* < 0.01, ***p* < 0.001.

Initial GSEA indicated a significant association between DNER expression and glycolytic pathways in RCC (**Figure 5A**). Consistent with this, DNER mRNA levels showed a strong positive correlation with the expression of key glycolytic enzymes LDHA and PKM (**Figure 5B**; **Supplementary Figure 1B**). Functionally, DNER overexpression in 786-O cells upregulated both mRNA and protein levels of LDHA and PKM2, while its knockdown in ACHN cells downregulated their expression (**Figure 5C**; **Supplementary Figures 1C, D**). Consequently, DNER overexpression enhanced glycolytic flux, increasing ATP production, glucose consumption, and lactate secretion, whereas DNER knockdown suppressed these metabolic activities (**Figures 5D, E**).

Bioinformatic analysis further linked DNER to the JAK2/STAT3 signaling pathway (**Supplementary Figure 1E**). Notably, manipulating DNER expression specifically altered the phosphorylation levels of JAK2 and STAT3 (p-JAK2, p-STAT3) without affecting their total protein amounts (**Figure 5F**; **Supplementary Figure 1F**). Pharmacological inhibition of JAK2/STAT3 with WP1066 in DNER-overexpressing cells reversed the increases in p-JAK2, p-STAT3, LDHA, and PKM2 levels (**Figure 5G**; **Supplementary Figure 1G**) and attenuated the enhanced glycolytic phenotype (**Figure 5I**). Conversely, activating the pathway with Butyzamide in DNER-knockdown cells rescued the suppression of both pathway activity and glycolysis (**Supplementary Figures 1H-J**). To further elucidate the direct mechanism by which DNER initiates JAK2/STAT3 signaling, we performed co-immunoprecipitation (Co-IP) assays and identified a physical interaction between DNER and the JAK2 protein (**Supplementary Figure 1K**). To confirm the specificity of this axis, we ectopically expressed STAT3 in DNER-knockdown cells, which successfully restored LDHA/PKM2 expression and glycolytic activity (**Figures 5H, J**; **Supplementary Figure 1L**).

Mechanistically, subcellular fractionation revealed that DNER overexpression promoted nuclear translocation of p-STAT3, while DNER knockdown reduced it (**Figure 5K**; **Supplementary Figure 2A**), implicating enhanced STAT3 transcriptional activity. Direct binding of STAT3 to specific promoter regions of LDHA (–1840) and PKM (-380) was confirmed by ChIP (**Figure 5L**), providing a molecular basis for their transcriptional upregulation.

In summary, these data delineate a coherent signaling cascade wherein DNER activates JAK2/STAT3 phosphorylation, leading to p-STAT3 nuclear translocation. Nuclear p-STAT3 then directly transactivates LDHA and PKM, driving glycolytic reprogramming in ccRCC cells.

### 3.6 DNER-induced glycolytic shift promotes M2-like macrophage polarization via lactate production

**Figure 6:**
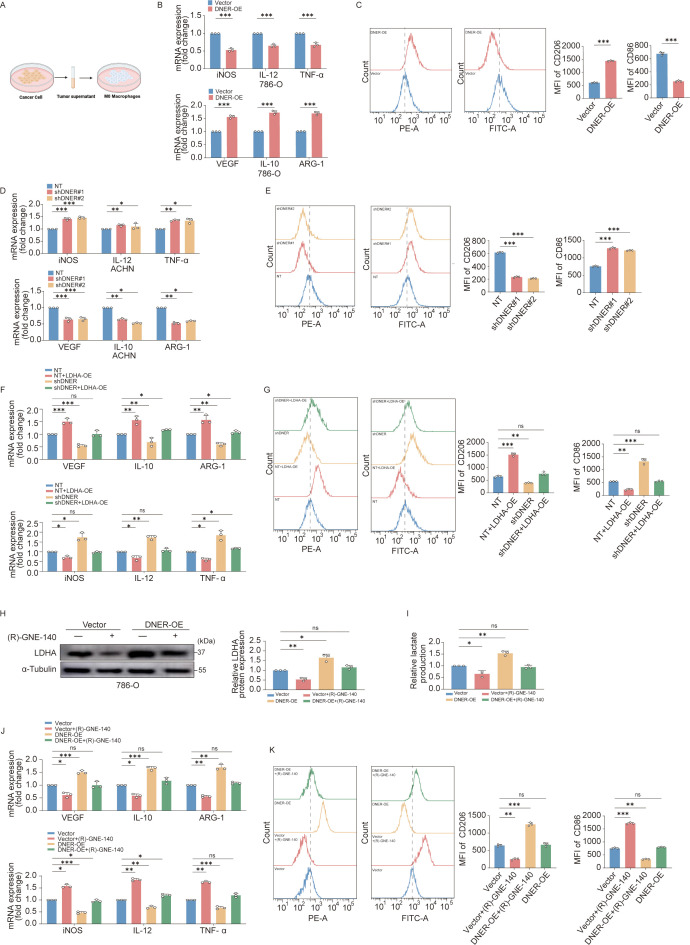
DNER promotes M2−like macrophage polarization via LDHA−mediated metabolic reprogramming. **(A)** Schematic illustration of the co−culture model. Conditioned medium (CM) derived from ccRCC cells with either DNER overexpression or DNER knockdown was collected and applied to PMA−induced M0 macrophages differentiated from THP−1 cells. **(B)** qRT−PCR analysis of M1−related (iNOS, IL−12, TNF−α) and M2−related (VEGF, IL−10, ARG−1) gene expression in macrophages treated with CM from DNER−overexpressing 786−O cells. **(C)** Flow cytometry analysis of surface markers CD86 (M1) and CD206 (M2) in macrophages treated with CM from DNER−overexpressing 786−O cells. Left panels: representative histogram overlays. Right panels: quantitative summary of mean fluorescence intensity (MFI). **(D)** qRT−PCR analysis of M1− and M2−related gene expression in macrophages treated with CM from DNER−knockdown ACHN cells. **(E)** Flow cytometry analysis of CD86 and CD206 expression in macrophages treated with CM from DNER−knockdown ACHN cells. Left: histogram overlays. Right: quantitative MFI data. **(F)** qRT−PCR analysis of M1− and M2−related gene expression in macrophages treated with CM from ACHN cells under four conditions: control, control + LDHA overexpression, DNER knockdown, and DNER knockdown + LDHA overexpression. **(G)** Flow cytometry analysis of CD86 and CD206 expression in macrophages treated with CM from the four indicated ACHN cell groups. Left: representative histograms. Right: quantitative MFI comparison. **(H)** Western blot validation of LDHA inhibition by 3 μM (R)-GNE-140 in DNER-overexpressing 786-O cells. **(I)** Lactate concentration in CM from DNER-overexpressing 786-O cells treated with or without (R)-GNE-140. **(J)** qRT-PCR analysis of M1- and M2-related gene expression in macrophages treated with CM from DNER-overexpressing 786-O cells ± (R)-GNE-140 treatment. **(K)** Flow cytometry analysis of CD86 and CD206 expression in macrophages treated with CM from the indicated groups. Left: representative histograms. Right: quantitative comparison. Data are presented as mean ± SD (n = 3 independent experiments). *p* < 0.05, **p* < 0.01, ***p* < 0.001.

To investigate the downstream immunomodulatory effect of DNER-driven metabolic reprogramming, we collected conditioned medium (CM) from RCC cells with altered DNER expression and applied it to macrophage cultures (**Figure 6A**). CM from DNER-overexpressing RCC cells induced a pronounced shift in macrophages toward a tumor-associated (M2-like) phenotype. This shift was characterized by downregulated mRNA levels of pro-inflammatory markers (iNOS, IL-12, TNF-α) and upregulated levels of pro-tumorigenic markers (VEGF, IL-10, ARG-1) (**Figure 6B**). Flow cytometry analysis further confirmed an increased proportion of CD206^+^ macrophages and a decreased proportion of CD86^+^ macrophages (**Figure 6C**). Conversely, CM from DNER-knockdown cells induced a shift toward a pro-inflammatory (M1-like) macrophage profile (**Figures 6D, E**). These results indicate that DNER-mediated alterations in RCC cells can functionally reprogram macrophage polarization within the tumor microenvironment.

Given the established role of lactate, a key glycolytic byproduct, in promoting M2-like macrophage polarization, and our prior finding that DNER upregulates LDHA expression, we hypothesized that DNER influences macrophage polarization through an LDHA-lactate axis. To test this, we rescued LDHA expression in DNER-knockdown RCC cells (**Supplementary Figure 2B**). Strikingly, LDHA overexpression effectively reversed the macrophage-polarizing effects induced by DNER knockdown, restoring the M2-like phenotype in macrophages treated with the CM (**Figures 6F, G**). Consistently, this genetic rescue restored lactate production in the CM of DNER-knockdown cells (**Supplementary Figure 2C**).

To further substantiate the causal role of lactate in this process, we employed a pharmacological approach using the LDHA inhibitor (R)-GNE-140 in DNER-overexpressing 786-O cells (**Figure 6H**). Treatment with (R)-GNE-140 markedly reduced LDHA expression and decreased lactate levels in the CM (**Figure 6I**). When macrophages were treated with CM collected from these inhibitor-treated cells, we observed a significant attenuation of M2-like tumor-associated macrophage polarization (**Figures 6J, K**). In a complementary set of experiments, we supplemented the CM of DNER-knockdown ACHN cells with exogenous sodium lactate. Notably, the addition of sodium lactate was sufficient to counteract the reduction in M2-like macrophage polarization caused by DNER depletion (**Supplementary Figures 2D, E**).

Taken together, these data establish a paracrine signaling cascade in which elevated DNER expression in RCC cells promotes LDHA-mediated lactate production. The resulting accumulation of extracellular lactate within the tumor microenvironment consequently induces macrophage polarization toward a pro-tumorigenic M2-like phenotype.

### 3.7 DNER overexpression sensitizes RCC to Olaparib by targeting metabolic-immune crosstalk

**Figure 7:**
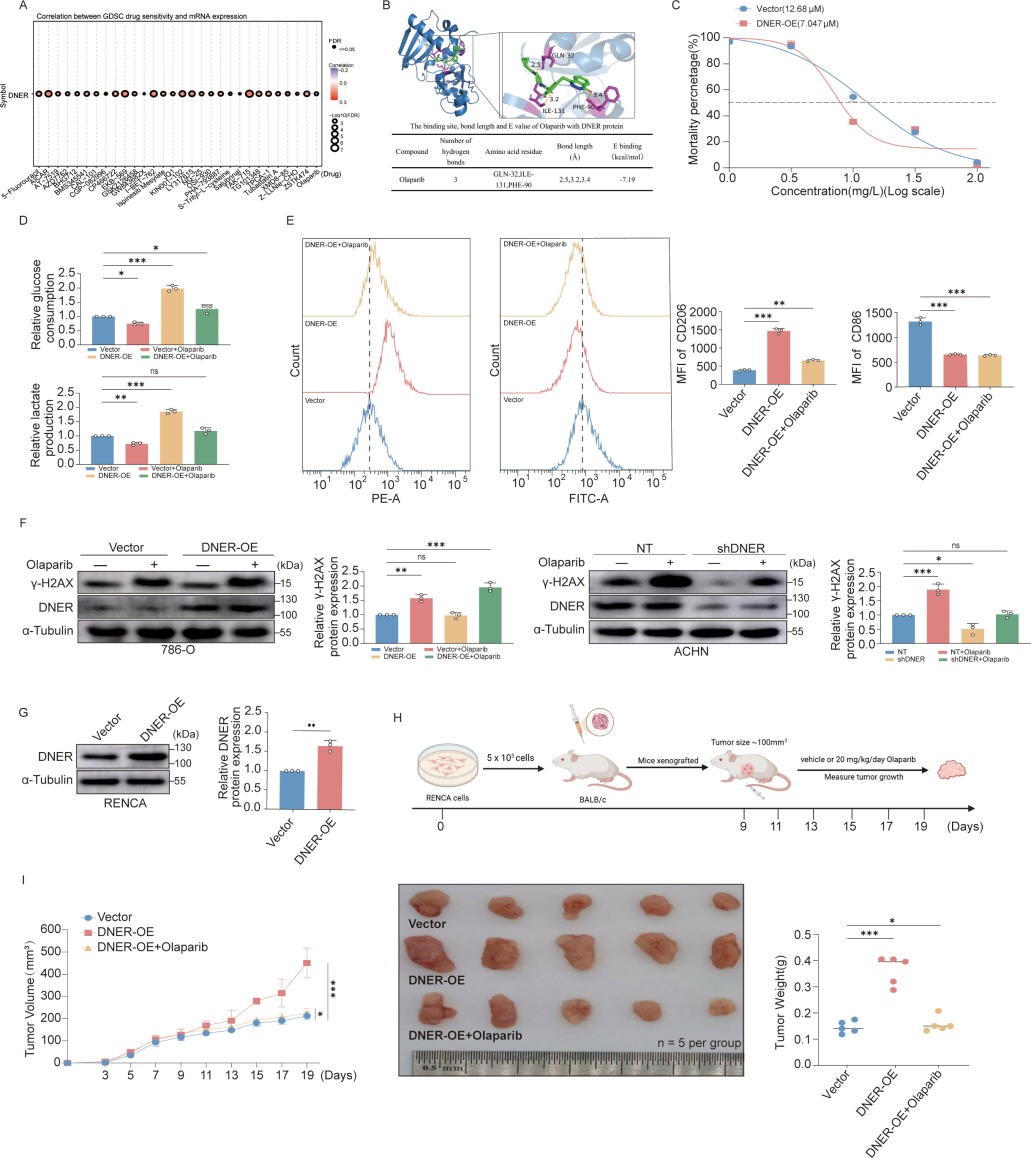
Targeting DNER enhances Olaparib efficacy and reverses immunosuppression through metabolic reprogramming. **(A)** Enrichment analysis of DNER expression based on tumor drug sensitivity databases. **(B)** Molecular docking screening of Olaparib against DNER (PDB ID: 5ONS). The docking procedure was performed using AutoDock Tools 1.5.7 and AutoDock Vina (http://vina.scripps.edu/), and the resulting conformations were visualized in PyMOL. Upper panel: Representative docking pose of Olaparib bound to the DNER protein. Lower panel: Table summarizing the binding site residues, interaction bond lengths, and corresponding binding energy (E value) for the Olaparib–DNER complex. **(C)** Cell viability assay of DNER−overexpressing and control 786−O cells treated with increasing concentrations of Olaparib (0, 1, 3.16, 10, 31.6, 100 µM). Absorbance was measured and the half−maximal inhibitory concentration (IC_50_) was calculated. **(D)** Metabolic analysis of DNER−overexpressing 786−O cells following Olaparib treatment. Upper panel: Glucose consumption in culture supernatant. Lower panel: Lactate production in culture supernatant. **(E)** Flow cytometry analysis of CD86 (M1 marker) and CD206 (M2 marker) expression in macrophages treated with conditioned medium from 786−O cells under three conditions: control, DNER overexpression, and DNER overexpression + Olaparib treatment. Left: Overlay histograms. Right: Quantified mean fluorescence intensity (MFI). **(F)** Western blot analysis of γ-H2AX protein levels. Left: γ-H2AX expression in DNER-overexpressing 786-O cells treated with or without 7 μM Olaparib. Right: γ-H2AX expression in DNER-knockdown ACHN cells treated with or without 7 μM Olaparib. **(G)** Validation of DNER overexpression in RENCA mouse cell lines by Western blot. GAPDH served as the loading control. **(H)** Schematic of the *in vivo* experimental design. RENCA cells expressing empty vector (control) or DNER were subcutaneously inoculated into BALB/c mice (n = 5 per group). Treatment consisted of intraperitoneal injection of Olaparib (20 mg/kg) administered for 10 days in a discontinuous schedule: 5 consecutive days of treatment followed by a 2-day interval, for two cycles. Control animals received an equal volume of phosphate-buffered saline (PBS) via the same route. Tumor volumes were measured every 2 days using calipers, and mice were sacrificed on day 19. **(I)** Tumor growth analysis in the xenograft model. Left: Tumor volume growth curves (n = 5 mice per group). Middle: Representative photographs of excised tumors at the experimental endpoint. Right: Quantification of final tumor weights. Data are presented as mean ± SD (n = 3 independent experiments). *p* < 0.05, **p* < 0.01, ***p* < 0.001.

Analysis of the Genomics of Drug Sensitivity in Cancer (GDSC) database predicted that elevated DNER expression is associated with increased sensitivity to Olaparib (**Figure 7A**). Molecular docking simulations further suggested potential interaction sites between Olaparib and the DNER protein, indicating favorable binding capability (**Figure 7B**).

Consistent with these predictions, DNER-overexpressing RCC cells exhibited significantly lower half-maximal inhibitory concentration (IC_50_) values for Olaparib compared to control cells (**Figure 7C**). Functionally, Olaparib treatment attenuated the enhanced glycolytic phenotype in DNER-overexpressing cells, reducing both glucose consumption and lactate production (**Figure 7D**). Moreover, conditioned medium from Olaparib-treated, DNER-high cells showed a diminished capacity to induce M2-like macrophage polarization, as evidenced by flow cytometry (**Figure 7E**).

To further explore the mechanism underlying the effect of DNER on Olaparib sensitivity, we examined the protein levels of γ-H2AX (37, 38), a well-established marker of DNA double-strand breaks (**Figure 7F**). Olaparib, as a PARP inhibitor, is known to impair DNA damage repair by trapping PARP enzymes on chromatin, thereby leading to the accumulation of unresolved DNA double-strand breaks and increased γ-H2AX expression. DNER overexpression alone did not significantly increase γ-H2AX levels, suggesting that DNER does not directly induce DNA damage. However, upon Olaparib treatment, γ-H2AX protein levels were markedly higher in DNER-overexpressing cells than in Olaparib-treated control cells, indicating that DNER sensitizes tumor cells to PARP inhibitor-induced DNA damage. Conversely, knockdown of endogenous DNER attenuated Olaparib-induced γ-H2AX upregulation, further demonstrating that endogenous DNER is required for a robust DNA damage response to PARP inhibition. Collectively, these results suggest that DNER positively regulates the sensitivity of ccRCC cells to PARP inhibitors. It should be noted, however, that the precise molecular link between DNER-mediated metabolic reprogramming and the DNA damage response remains to be fully elucidated.

To validate these findings *in vivo*, we established a mouse renal carcinoma model using RENCA cells overexpressing DNER (**Figure 7G**). Mice bearing DNER-overexpressing tumors were treated with Olaparib according to the regimen outlined (**Figure 7H**). Olaparib administration significantly inhibited tumor growth compared to the vehicle control (**Figure 7I**).

In summary, these integrative computational, molecular, and phenotypic analyses suggest that elevated DNER expression is associated with enhanced sensitivity of ccRCC cells to Olaparib. This effect is characterized by an augmented DNA damage response, suppressed glycolytic metabolism, and attenuated macrophage polarization. However, the direct mechanistic link between DNER-mediated metabolic alterations and PARP inhibitor sensitivity remains to be further investigated.

## 4 Conclusion

DNER drives the progression of this malignancy by activating the JAK2/STAT3/LDHA axis, promoting glycolysis and lactate− mediated M2 polarization, thus linking metabolic and immune suppression. DNER is a potential prognostic biomarker and therapeutic target; its high expression predicts olaparib sensitivity, supporting a precision−medicine approach for ccRCC.

## 5 Discussion

This study reveals the functional role and mechanistic significance of DNER in ccRCC through integrated multi-omics bioinformatics analysis and experimental validation, delineating a DNER-driven signaling axis that couples glycolytic reprogramming to macrophage polarization. Bioinformatics analyses confirmed that lipid metabolism and energy metabolism are the key metabolic pathways significantly associated with patient prognosis in ccRCC. Consensus clustering based on these two pathways stratified patients into two distinct metabolic subtypes (C1 and C2) with differential lipid metabolism activity, providing a metabolic framework for subsequent DNER-focused investigation.

Focusing on the key lipid and energy metabolism pathways in ccRCC, we identified DNER as a hub gene through systematic screening of metabolism-related candidates. DNER was originally identified as a transmembrane protein highly expressed in cerebellar Purkinje neurons, where it plays a critical role in neuronal development (39). Beyond the nervous system, emerging evidence has linked DNER to the proliferation and malignant progression of various cancers (40–42). Some studies indicate that DNER knockdown inhibits cancer cell proliferation, migration, and invasion *in vitro* and reduces tumor growth *in vivo* (40). Conversely, in glioblastoma, DNER-mediated inhibition of histone deacetylase (HDAC) induces specific gene products that suppress the growth of glioblastoma-derived neurospheres, promote differentiation, and ultimately inhibit tumor xenograft growth (43). These inconsistent findings underscore the context-dependent nature of DNER function. Our results demonstrate variable DNER expression across different ccRCC cell lines, and through gain- and loss-of-function experiments we confirmed that DNER directly promotes the proliferative capacity of ccRCC cells in an expression-dependent manner. Furthermore, immune infiltration analysis revealed a significant association between DNER expression and macrophage polarization within the tumor microenvironment, providing a rationale for subsequent functional.

We further demonstrated that DNER orchestrates a linear signaling cascade linking metabolism to immunity. Specifically, DNER activates JAK2/STAT3 phosphorylation without affecting total protein levels, and co-immunoprecipitation assays revealed a physical interaction between DNER and JAK2, providing mechanistic insight into the upstream activation of this pathway. However, the precise molecular details by which the intracellular domain of DNER mediates JAK2 activation, and whether additional adaptor proteins are involved in this process, remain to be elucidated by further structural and functional studies. Activated STAT3 undergoes nuclear translocation and directly binds to the promoter regions of key glycolytic enzymes LDHA and PKM, driving their transcription. This finding directly connects the canonical transcriptional regulatory function of STAT3 to tumor glycolysis, offering molecular evidence for its role in promoting metabolic reprogramming. Furthermore, DNER-driven upregulation of LDHA results in increased lactate production in the tumor microenvironment. Through both genetic rescue and pharmacological inhibition experiments, we demonstrated that lactate serves as a key metabolic signaling molecule that induces macrophage polarization toward an M2-like phenotype, consistent with established literature implicating microenvironmental lactate in fostering immunosuppressive niches (44, 45). These data establish a paracrine signaling cascade in which tumor cell-intrinsic DNER upregulation triggers JAK2/STAT3-mediated glycolytic activation, leading to lactate accumulation that subsequently remodels macrophage function toward a pro-tumorigenic phenotype. This model complements existing research on immune escape driven by tumor metabolism and provides a mechanistic explanation for the stronger immunosuppressive features observed in tumors with high DNER expression (46, 47).

Cellular DNA is continuously exposed to potentially damaging agents, and this damage must be repaired by specific DNA damage repair (DDR) pathways (48–50). Olaparib, a PARP inhibitor, has demonstrated clinical benefit in treating patients with advanced tumors harboring DDR defects (49, 51). Through computational prediction and experimental validation, we found that elevated DNER expression sensitizes ccRCC cells to Olaparib, as evidenced by reduced IC_50_ values, attenuated glycolytic activity, and diminished M2-like macrophage polarization upon treatment. γ-H2AX, the phosphorylated form of histone variant H2AX, is a well-established marker of DNA double-strand breaks (DSBs) and plays a critical role in recruiting DNA repair proteins to damage sites (52, 53). Olaparib, a PARP inhibitor, traps PARP enzymes on chromatin, blocking single-strand break repair and leading to DSB accumulation reflected by elevated γ-H2AX levels (54, 55). This association has been extensively documented across multiple cancer types. In ovarian cancer, Olaparib increased γ-H2AX expression in BRCA2-deficient cells, an effect further augmented by metabolic modulators (55, 56). In prostate cancer, Olaparib selectively increased residual γ-H2AX/53BP1 foci in ERG-positive tumors, indicating enhanced radiosensitization (57). In colorectal cancer, Olaparib alone failed to induce significant DSBs but markedly enhanced oxaliplatin-induced γ-H2AX foci formation and G2/M arrest (58, 59). Through computational prediction and experimental validation, we found that elevated DNER expression sensitizes ccRCC cells to Olaparib, as evidenced by reduced IC_50_ values, attenuated glycolytic activity, and diminished M2-like macrophage polarization. Mechanistically, DNER overexpression enhanced Olaparib-induced γ-H2AX upregulation—consistent with the established role of γ-H2AX as a pharmacodynamic biomarker of PARP inhibitor activity—suggesting that DNER may potentiate PARP inhibitor sensitivity by augmenting the DNA damage response. However, the precise molecular link between DNER-mediated metabolic reprogramming and the DNA damage repair machinery remains to be fully elucidated.

While this study provides mechanistic insights into the role of DNER in ccRCC, several limitations should be acknowledged. First, although we demonstrated a physical interaction between DNER and JAK2 by co-immunoprecipitation, the precise structural domains mediating this interaction and whether additional adaptor proteins are involved remain to be determined. Future studies employing domain mapping, proximity labeling, or structural biology approaches will be necessary to fully delineate the DNER-JAK2 activation mechanism. Second, the *in vivo* experiments primarily focused on the therapeutic efficacy of Olaparib. The construction of conditional knockout or transgenic DNER-overexpressing mouse models would enable a more rigorous assessment of the autonomous role of DNER in regulating tumor growth, metabolic phenotype, and the immune microenvironment within an intact immune system. Third, the metabolic subtyping (C1/C2) was established and evaluated solely within the TCGA cohort; its robustness and generalizability should be further validated in independent, multi-center datasets. Finally, the association between DNER expression and Olaparib sensitivity, although supported by both computational and experimental data, requires validation in prospective clinical cohorts before any translational application can be considered.

## Data Availability

The original contributions presented in the study are included in the article/**Supplementary Material**. Further inquiries can be directed to the corresponding authors.
