# Supplementary material for: DNER drives glycolytic reprogramming in renal cell carcinoma by activating the JAK2/STAT3 signaling pathway
Source: Front Immunol. 2026 May 22;17:1799104. doi: 10.3389/fimmu.2026.1799104 (PMC13236898; doi:10.3389/fimmu.2026.1799104)

**DNER drives glycolytic reprogramming in renal cell carcinoma by activating the JAK2/STAT3 signaling pathway**

An-rui Li^a^, Jing-wen Xu ^a^, Jian-hua Qin ^b^, Qi yuan^c*^, Li-chen Teng^a*^

^a^ Harbin Medical University Cancer Hospital, Heilongjiang, Harbin 150081 China

^b^ The HIT Center for Life Sciences, School of Life Science and Technology, Harbin Institute of Technology, Harbin 150080, China., Harbin 150081, China

^c^ College of Life Science, Mudanjiang Medical University, Mudanjiang 157011, China.

^*^Correspondence:

Li-Chen Teng, Harbin Medical University Cancer Hospital,

E-mail: tenglichen@hrbmu.edu.cn

Qi Yuan, College of Life Science, Mudanjiang Medical University

E-mail: yuanqi@mdjmu.edu.cn


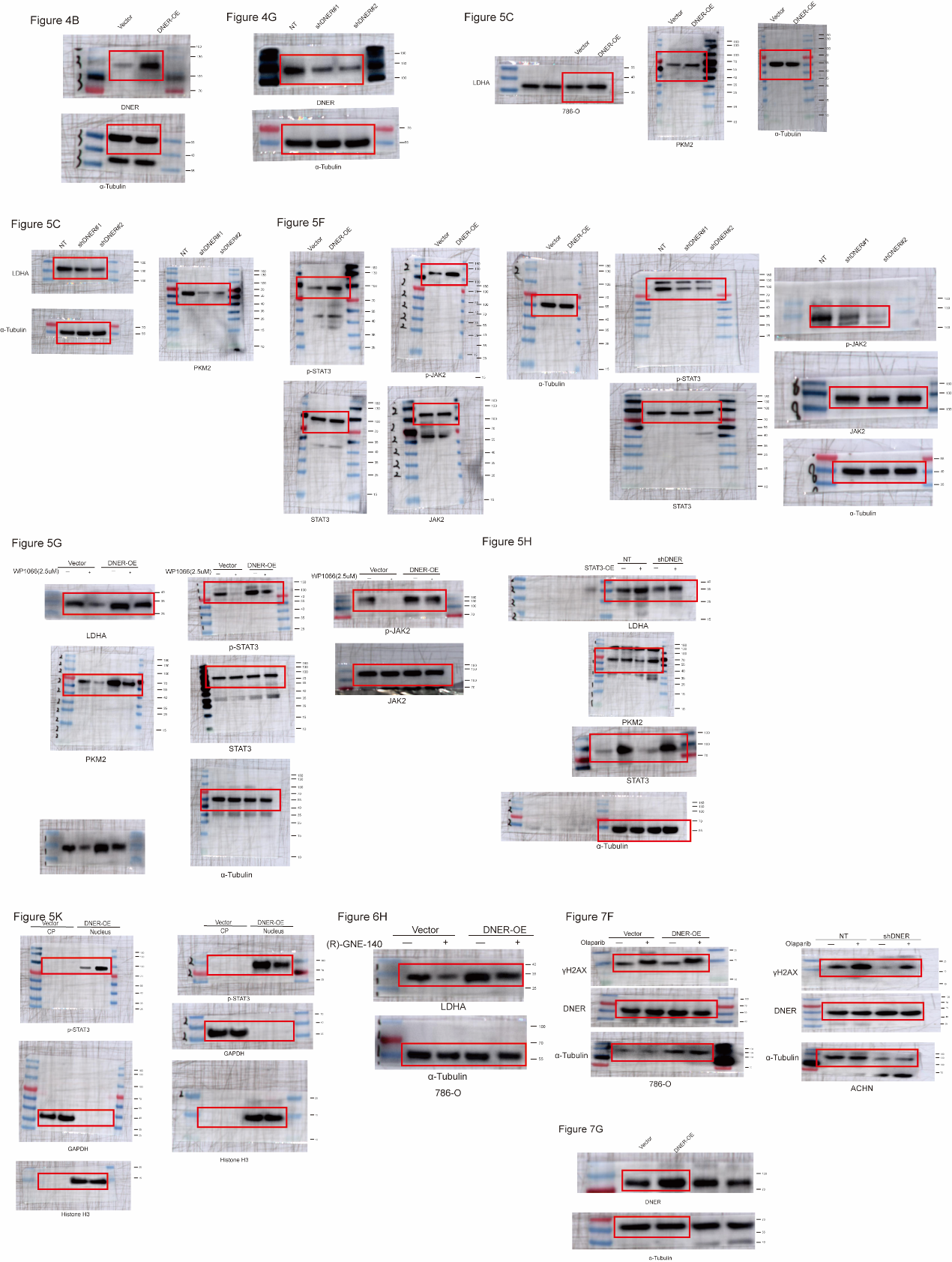


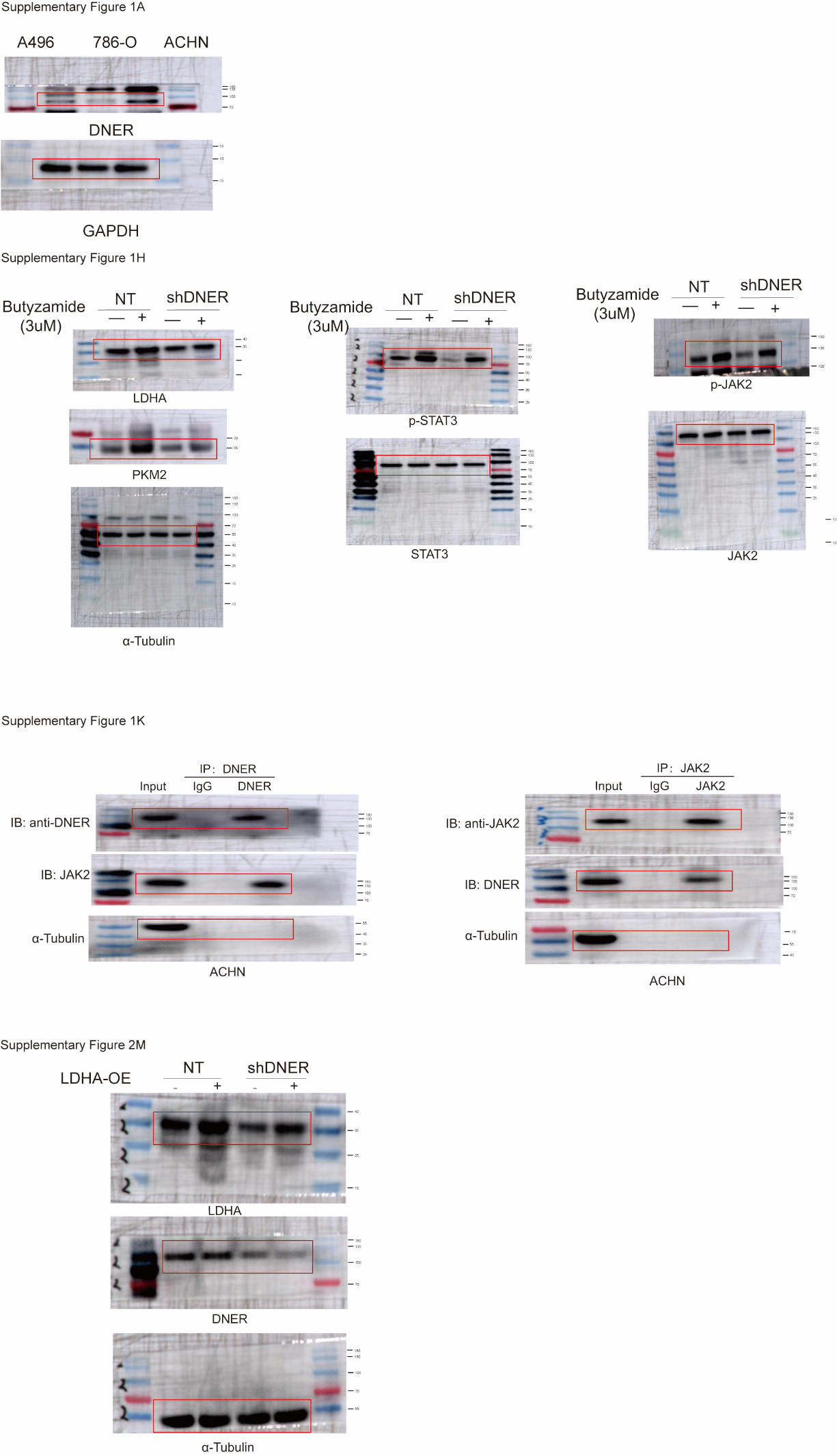

Supplement: Supplementary file 1 [file DataSheet1.docx]
