# Supplementary material for: DNER drives glycolytic reprogramming in renal cell carcinoma by activating the JAK2/STAT3 signaling pathway
Source: Front Immunol. 2026 May 22;17:1799104. doi: 10.3389/fimmu.2026.1799104 (PMC13236898; doi:10.3389/fimmu.2026.1799104)
Supplement: Supplementary file 2 [file DataSheet2.docx]

**DNER drives glycolytic reprogramming in renal cell carcinoma by activating the JAK2/STAT3 signaling pathway**

Anrui Li^a^, Jingwen Xu ^a^, Jianhua Qin ^b^, Qi yuan^c*^, Lichen Teng^a*^

^a^ Department of Urology, Harbin Medical University Cancer Hospital, Harbin, Heilongjiang, China

^b^ The HIT Center for Life Sciences, School of Life Science and Technology, Harbin Institute of Technology, Harbin 150081, China

^c^ College of Life Science, Mudanjiang Medical University, Mudanjiang 157011, China.

^*^Correspondence:

Lichen Teng, Harbin Medical University Cancer Hospital,

E-mail: tenglichen@hrbmu.edu.cn

Qi Yuan, College of Life Science, Mudanjiang Medical University

E-mail: yuanqi@mdjmu.edu.cn


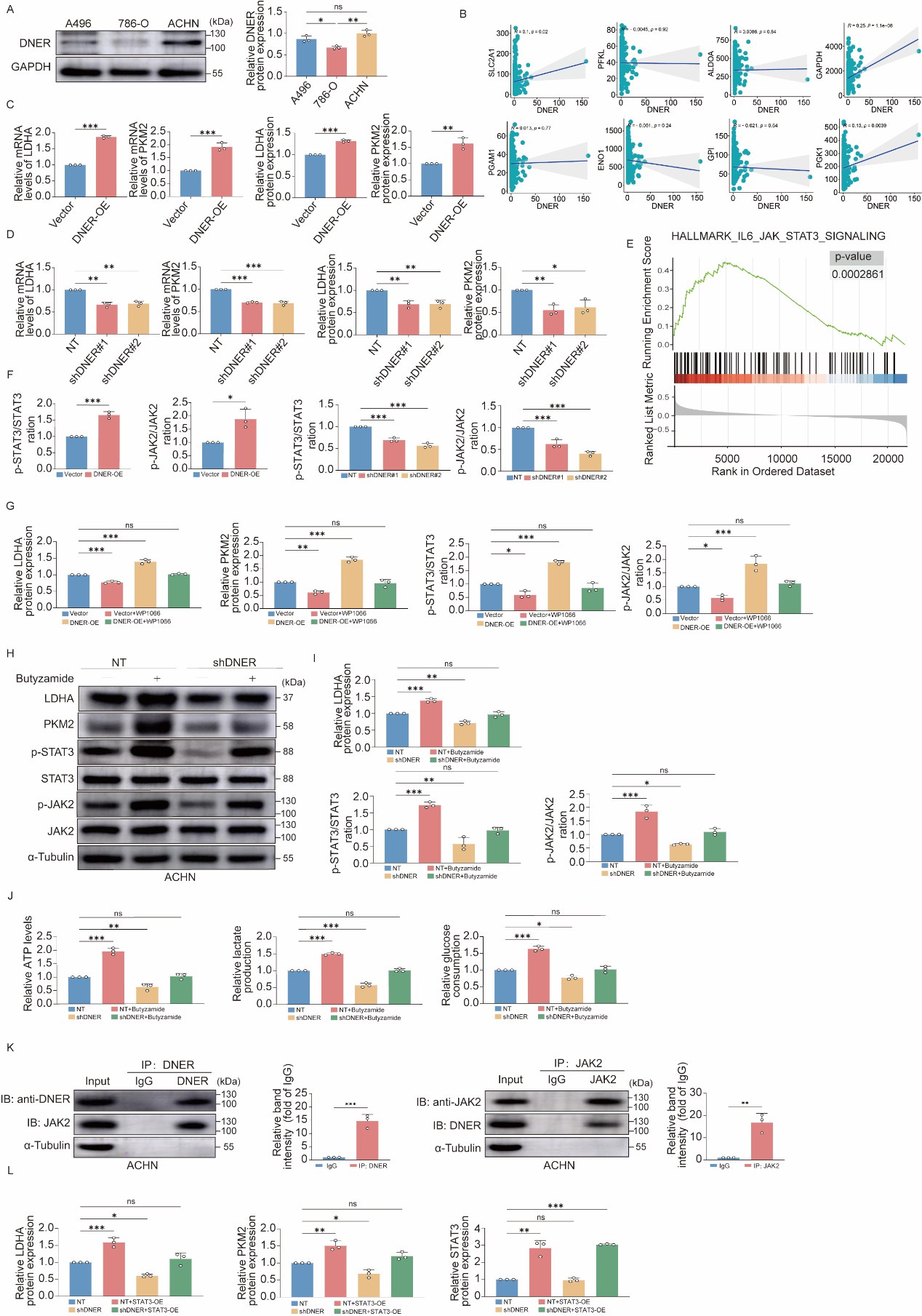


**Supplementary Figure 1. DNER regulates glycolysis through the JAK2/STAT3 signaling axis in RCC.**

(A) Western blot analysis of DNER protein expression in three RCC cell lines (A496, 786‑O, ACHN). GAPDH served as the loading control. Left: representative blots; Right: quantitative densitometric analysis.

(B) Correlation analysis between DNER expression levels and key glycolytic proteins.

(C) Expression of LDHA and PKM2 in DNER‑overexpressing cells. Left: mRNA levels measured by qRT‑PCR; Right: protein levels determined by Western blot with α‑tubulin as the loading control.

(D) Expression of LDHA and PKM2 in DNER‑knockdown cells. Left: mRNA levels measured by qRT‑PCR; Right: protein levels determined by Western blot with α‑tubulin as the loading control.

(E) GSEA results of HALLMARK_IL6_JAK_STAT3_SIGNALING.

(F) Quantitative analysis of phosphorylated‑STAT3 (p‑STAT3) to total STAT3 and phosphorylated‑JAK2 (p‑JAK2) to total JAK2 ratios by Western blot. Left: DNER‑overexpressing 786‑O cells; Right: DNER‑knockdown ACHN cells.

(G) Western blot analysis of LDHA, PKM2, and phospho‑to‑total ratios of STAT3 and JAK2 in DNER‑overexpressing 786‑O cells treated with or without the JAK2/STAT3 inhibitor WP1066. α‑tubulin served as the loading control. Left: protein levels of LDHA and PKM2; Right: quantitative ratios of p‑STAT3/STAT3 and p‑JAK2/JAK2.

(H‑I) Western blot analysis of LDHA, PKM2, p-STAT3, STAT3, p-JAK2, and JAK2 in DNER-knockdown ACHN cells treated with or without 3 μM Butyzamide (H) and corresponding quantitative densitometric analysis (I). α-tubulin served as the loading control.

(J) Metabolic analysis of ATP production, glucose consumption, and lactate secretion in DNER‑knockdown ACHN cells treated with or without Butyzamide.

(K) Co-immunoprecipitation (Co-IP) analysis of the physical interaction between DNER and JAK2 in ACHN cells. Left: immunoprecipitation (IP) with anti-DNER antibody, followed by immunoblotting (IB) for JAK2. Right: reciprocal Co-IP using anti-JAK2 antibody for IP, followed by IB for DNER. The bar graph shows the densitometric analysis of three independent experiments, presented as the fold change of the target protein band intensity in the IP group relative to the IgG control group (mean ± SD). Paired t-test revealed that the signals in both IP groups were significantly higher than their respective IgG controls

(L) Metabolic analysis of ATP production, glucose consumption, and lactate secretion in DNER-knockdown ACHN cells with or without STAT3 overexpression.

Data are presented as mean ± SD (n = 3 independent experiments). *p* < 0.05, **p* < 0.01, ***p* < 0.001.


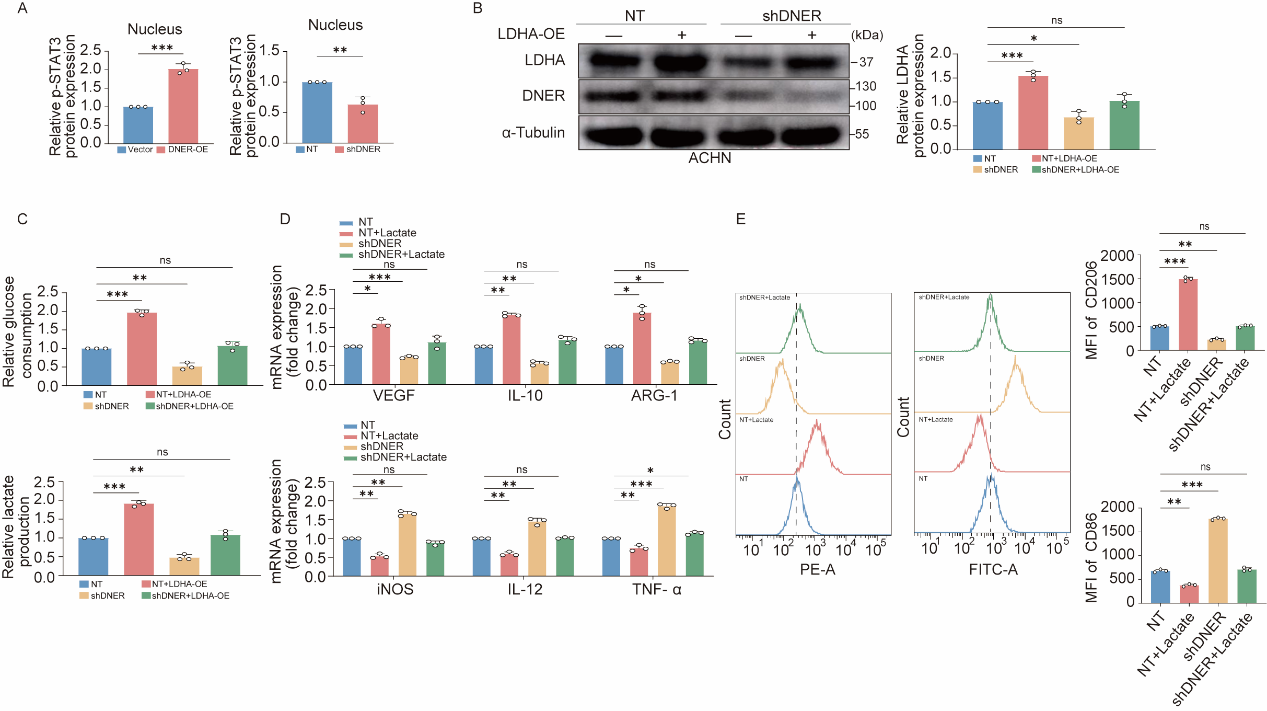


**Supplementary Figure 2. DNER-driven lactate production mediates M2-like macrophage polarization.**

(A) Quantitative analysis of nuclear p-STAT3 levels following subcellular fractionation. Upper panel: DNER-overexpressing 786-O cells; Lower panel: DNER-knockdown ACHN cells.

(B) Western blot validation of LDHA overexpression in DNER-knockdown ACHN cells. α-tubulin served as the loading control.

(C) Metabolic analysis of glucose consumption and lactate secretion in DNER-knockdown ACHN cells with or without concomitant LDHA overexpression.

(D) qRT-PCR analysis of M1- and M2-related gene expression in macrophages treated with conditioned medium (CM) from DNER-knockdown ACHN cells ± 10 mM sodium lactate treatment.

(E) Flow cytometry analysis of CD86 and CD206 expression in macrophages treated with CM from the indicated groups. Left: representative histograms; Right: quantitative comparison.

Data are presented as mean ± SD (n = 3 independent experiments). *p* < 0.05, **p* < 0.01, ***p* < 0.001.

**Supplementary Table S6**

| Gene | Species | Forward (5'-3') | Reverse (5'-3') |
| --- | --- | --- | --- |
| DNER | Human | AGTCTCAGTGGATTCACCTGCC | CCACATAGCAGGTGCCGTTGTT |
| VEGF | Human | TTGCCTTGCTGCTCTACCTCCA | GATGGCAGTAGCTGCGCTGATA |
| iNOS | Human | GAGACAGGGAAGTCTGAAGCAC | CCAGCAGTAGTTGCTCCTCTTC |
| TNF-α | Human | CTCTTCTGCCTGCTGCACTTTG | ATGGGCTACAGGCTTGTCACTC |
| IL-12 | Human | GACATTCTGCGTTCAGGTCCAG | CATTTTTGCGGCAGATGACCGTG |
| ARG1 | Human | CATTGGCTTGCGAGACGTAGAC | GCTGAAGGTCTCTTCCATCACC |
| IL-10 | Human | TCTCCGAGATGCCTTCAGCAGA | TCAGACAAGGCTTGGCAACCCA |
| PKM2 | Human | ATGGCTGACACATTCCTGGAGC | CCTTCAACGTCTCCACTGATCG |
| LDHA | Human | GGATCTCCAACATGGCAGCCTT | AGACGGCTTTCTCCCTCTTGCT |

**Supplementary Methods**

**ESTIMATE algorithm**

To quantify the infiltration levels of immune cells and stromal cells in each tumor sample, we used the ESTIMATE algorithm to analyze the gene expression data of the TCGA-KIRC cohort. The analysis was completed using the "estimate" R package. We obtained the immuneScore, stromalScore and ESTIMATEscore of each KIRC sample through this analysis.

**Metabolic pathway GSVA score**

To assess the metabolic reprogramming status of tumor samples, we employed the Gene Set Variation Analysis (GSVA) method to conduct a non-parametric unsupervised analysis of the gene expression data from the TCGA-KIRC cohort. We collected 7 specific metabolic pathway gene sets related to cancer metabolism from authoritative published literature (45). Using the GSVA R package, we calculated each predefined metabolic pathway gene set.

**Immune cell infiltration analysis**

To quantify the precise proportion of immune cell composition in the tumor microenvironment, we used the CIBERSORT algorithm to perform deconvolution analysis on the gene expression data of the TCGA-KIRC cohort. CIBERSORT is a machine learning method based on support vector regression, which can estimate the relative proportions of 22 immune cell subtypes. We used the standardized gene expression data of the TCGA-KIRC samples as the input file and ran the analysis on the official online portal of CIBERSORT (https://cibersort.stanford.edu/) with default parameters.

**Cluster analysis**

Based on the activity of metabolic pathways, we conducted a robust molecular classification of the TCGA-KIRC cohort using the consensus clustering method. This analysis employed the “ConsensusClusterPlus” R package. The input data was the standardized and filtered sample-feature matrix, and 1000 resampled with replacement were performed to evaluate the clustering stability. Through a comprehensive assessment of the consistency matrix, cumulative distribution function graph, and clustering consistency score of the results, we determined the optimal number of clusters.

**Differential expression analysis**

To identify the genes that show differential expression in different groups, we conducted a differential expression analysis on the TCGA-KIRC data. We used the “limma” R package. We extracted the log2 fold change (log2FC) and the corrected false discovery rate (FDR) after multiple tests for each gene. We set |log2FC| > 1 and FDR < 0.05 as the threshold for differential expression.

**Weighted Gene Co-expression Network Analysis (WGCNA)**

To systematically study the coordinated changes in gene expression and identify functional modules highly related to clinical phenotypes, we conducted a weighted gene co-expression network analysis on tumor samples from the TCGA-KIRC cohort. Using the “WGCNA” R package, we first calculated the correlation between all gene pairs using the Pearson correlation coefficient, and then transformed the correlation coefficient matrix into an adjacency matrix using the soft threshold power β (determined according to the scale-free topological criterion, β = 5) to emphasize strong correlations and weaken weak correlations. Subsequently, we converted the adjacency matrix into a topological overlap matrix to more robustly measure the network connectivity between genes. Using average linkage hierarchical clustering and dynamic tree cutting algorithms, we clustered genes with high topological overlap into different co-expression modules, each module being represented by a unique identifier.

**Mutation analysis**

This study utilized the mutation data provided by the TCGA-KIRC project (MAF format files) to systematically analyze the genomic variation characteristics of renal clear cell carcinoma. The “maftools” R package was used to read and process the MAF files. We first summarized the mutation data of all samples, calculated the tumor mutation burden, and counted the total number of mutations for each patient. Through the creation of a waterfall chart, the somatic mutation status of the top 10 genes with the highest mutation frequency in the cohort was presented, including single nucleotide variations, insertions, deletions, etc., and was visualized according to predefined mutation classifications (such as missense mutations, nonsense mutations, etc.) and variant allele frequencies.

**Reagents and Antibodies**

The following primary antibodies were utilized: anti-GAPDH (Proteintech, cat# 60004-1-Ig), anti-Histone H3 (Servicebio, cat# GB11102-100), anti-α-Tubulin (Proteintech, cat# 11224-1-AP), anti-STAT3 (Proteintech, cat# 10253-2-AP), anti-DNER (Proteintech, cat# 24362-1-AP), anti-DNER (Bioswamp, cat# PAB32541), anti-LDHA (Wanleibio, cat# WL03271), anti-PKM2 (Wanleibio, cat# WL03290), anti-p-JAK2 (Y007+Y1008) (Abways, cat# CY6570), anti-p-STAT3 (Y705) (Abways, cat# CY6566), anti-JAK2 (Biodragon, cat# RM8013), anti-STAT3 (Biodragon, cat# RM8301) and anti- Phospho-Histone H2A.X(Affinity, cat# AF3187).

Chemical reagents included: WP1066 (MCE, cat# HY-15312), Butyzamide (MCE, cat# HY-148748G), Olaparib (MCE, cat# HY-10162), xylene (Sinopharm Chemical Reagent, cat# 10023418), neutral paraformaldehyde fixative (Servicebio, cat# G1101), Phorbol 12-myristate 13-acetate (PMA) (MCE, cat# HY-18739), dimethyl sulfoxide (DMSO) for cell culture (Biosharp, cat# BL165B), Lactic acid sodium (MCE, cat# HY-B2227B) and (R)-GNE-140 (MCE, cat# HY-100742A)

**Bioinformatics Software and Parameters**

All bioinformatics analyses were conducted in R (version 4.3.1). Differential expression analysis between tumor and normal tissues was performed with DESeq2 (v1.40.0), applying thresholds of |log₂FC| > 1 and adjusted *p* < 0.05. Pathway activity scores were calculated using GSVA (v1.48.0). Co-expression networks were constructed with WGCNA (v1.72-1), with the soft-thresholding power selected based on scale-free topology. Consensus clustering was implemented via ConsensusClusterPlus (v1.64.0) using 1,000 iterations and an 80% subsampling ratio. Immune cell infiltration was estimated using CIBERSORT. ROC curves were generated with pROC (v1.18.0), and survival analyses relied on the survival (v3.5-5) and survminer (v0.4.9) packages..

**Co-Immunoprecipitation (Co-IP)**

ACHN cell lysates were prepared in ice-cold IP lysis buffer (Beyotime, #P0013) and cleared by centrifugation. Equal amounts of protein were incubated overnight at 4°C with 1 µg of specific antibodies or control rabbit IgG (Proteintech, #B900620), followed by incubation with Protein A/G magnetic beads. After washing, bound proteins were eluted in SDS loading buffer at 95°C for 10 min and analyzed by immunoblotting.

**Cell Viability Assay**

Stably transduced 786-O or ACHN cells in the logarithmic growth phase were re-seeded into 96-well plates. At 12, 24, 48, and 72 hours after seeding, cells were incubated with 20 μl of CCK-8 solution (5 mg/ml; Vazyme, cat# A311) for 4 hours. Absorbance was subsequently measured at 490 nm using a microplate reader.

**Colony Formation Assay**

Cells stably expressing target genes were plated during logarithmic growth and allowed to adhere overnight. Following culture in low‑serum medium for 14 days, colonies were fixed with 4% paraformaldehyde, stained with 0.1% crystal violet, and counted under a microscope.

**Wound Healing Assay**

Lentivirus‑transduced ACHN and 786‑O cells were grown to full confluence and scratched with a sterile 200 µL pipette tip. Migration was monitored by imaging the same field immediately after wounding and after 48 h of incubation. Wound closure was quantified by comparing the two time points.

**Determination and Calculation of IC₅₀ Values**

To assess the half‑maximal inhibitory concentration (IC₅₀) of Olaparib, 786‑O cells were plated at 5 × 10³ cells per well in 96‑well plates. After 24 h, cells were exposed to a range of Olaparib concentrations for 48 h. Viability was determined using the CCK‑8 assay, and the IC₅₀ was calculated by fitting a nonlinear regression curve to the log‑transformed drug concentration versus viability data.

**Collection of Conditioned Medium**

Conditioned medium (CM) was collected from subconfluent cultures prepared in 6‑cm dishes. The culture medium was replaced with serum‑free medium, and cells were incubated for 48 hours. Subsequently, the medium was collected and centrifuged at 200 × g for 3 minutes at 4°C to remove cellular debris.

**Isolation of Nuclear and Cytoplasmic Proteins**

Nuclear and cytoplasmic fractions were prepared from 2 × 10⁷ cells. Cells were resuspended in extraction buffer (20 mM HEPES, 10 mM KCl, 2 mM MgCl₂, 1 mM EDTA, 1 mM EGTA, 1 mM DTT, 1% protease inhibitor cocktail), incubated on ice for 15 min, and homogenized with a 27‑gauge needle. After centrifugation at 3,000 rpm for 5 min, the supernatant (cytoplasmic fraction) was collected. The nuclear pellet was washed, re‑homogenized with a 25‑gauge needle, centrifuged again, and resuspended in TBS containing 0.1% SDS. The nuclear lysate was briefly sonicated to shear genomic DNA.

**Metabolic Assays**

Glucose uptake, lactate secretion and ATP production were measured using commercial kits (Jiancheng Bioengineering). Glucose consumption was calculated by subtracting the residual glucose in the medium from the initial concentration. Lactate and ATP levels were normalized to total cellular protein content.

**Molecular Docking**

The interaction between olaparib and DNER (PDB ID: 5NOS) was predicted via molecular docking. The protein structure was prepared by removing water molecules and adding hydrogen atoms in AutoDock Tools 1.5.7. The 3D structure of olaparib was downloaded from PubChem (CID: 23725625) and energy-minimized. Docking simulations were performed using AutoDock Vina, with a grid box (40 Å³) covering the putative binding site. The docking pose with the lowest binding energy was selected, and the results were visualized and analyzed using PyMOL.
