# Supplementary material for: DNER drives glycolytic reprogramming in renal cell carcinoma by activating the JAK2/STAT3 signaling pathway
Source: Front Immunol. 2026 May 22;17:1799104. doi: 10.3389/fimmu.2026.1799104 (PMC13236898; doi:10.3389/fimmu.2026.1799104)
Supplement: Supplementary file 3 [file Table1.docx]

logFC AveExpr t P.Value adj.P.Val B

AQP2 -14.6914218707983 -1.68520649245482 -59.8966268380939 1.73016859856644e-256 3.78093743844725e-252 577.060086359978

PRR35 -8.80887767526601 -4.59000706603139 -58.1823909683077 5.49857382580916e-250 6.00801669077038e-246 561.944323233343

SLC9A4 -9.82943682126065 -3.2181990765207 -57.4096958500451 5.18816342730313e-247 3.77923117922851e-243 555.374682975126

FXYD4 -11.2549543741054 -2.80200050507882 -56.0738082679161 8.38877916501241e-242 4.5829997773254e-238 543.418662506389

ATP12A -8.57623780258326 -3.97732218760854 -55.4156256109443 3.31936665552572e-239 1.45076239046407e-235 537.303864217662

ACP3 -5.92892645243793 0.104680970279434 -54.9229201147845 3.01298487626551e-237 1.0973793083505e-233 532.98586721099

UMOD -14.5193430798518 -0.673243645874704 -54.5354674815702 1.0639528854228e-235 2.90993775453628e-232 529.323658528335

MUC15 -10.3357156241342 -3.26652278952562 -54.535332578932 1.06527717184324e-235 2.90993775453628e-232 529.395925021885

KNG1 -11.9725263187183 -0.778876944388282 -53.1716135426821 3.42052014601908e-230 8.30540297232832e-227 516.72270462991

HRG -9.32586926880901 -2.97013972684442 -53.0556958244975 1.01483009081029e-229 2.21770819744773e-226 515.651292495776

PIK3C2G -8.374245384837 -3.40357160825442 -52.8087377479703 1.03476374705779e-228 2.05569928767763e-225 513.266435287106

HS6ST2 -7.88731837051437 -1.69317072088018 -52.7275214739385 2.22407999377264e-228 4.05023500865946e-225 512.575022382895

TFAP2B -8.63029896664417 -2.62175192095583 -52.5641518295321 1.03896594263323e-227 1.74650174956646e-224 511.026526010765

ESRRB -6.4347841942694 -0.487179599642368 -51.9844135747497 2.52951637012158e-225 3.94839437401907e-222 505.566508122289

SEMG2 -6.12229535194183 -5.75550110117136 -51.8317322645984 1.08230815258495e-224 1.57677867056259e-221 503.004016233055

KCNJ1 -9.12154594668473 0.670860280654799 -51.5897248551937 1.09000719311862e-223 1.48874544945132e-220 501.821335294319

TMEM207 -8.76607553953759 -5.01974855551977 -51.2292814920947 3.44281512366715e-222 4.42563758220578e-219 498.126317717515

BSND -10.0994068181102 -3.60136413104085 -51.1338443799803 8.61092676199155e-222 1.04541434738779e-218 497.433174215485

TRPV6 -5.72863344056746 -1.3876902769322 -51.0903574378244 1.3080338734492e-221 1.50444548613081e-218 496.933991249821

CLDN8 -11.0759982354909 -3.0400756658307 -50.1202658609368 1.55665436612907e-217 1.70087839315093e-214 487.657622815514

DUSP9 -8.78649140840754 -1.22303168961834 -49.8692134821154 1.79810607802849e-216 1.87114343443603e-213 485.220064776026

COL4A6 -5.89071290540439 -0.887761470605079 -48.6971388078715 1.81629771924252e-211 1.80416154811849e-208 473.690468291424

SLC12A1 -11.1945796614912 1.06378322106911 -48.6342314979332 3.38696846240179e-211 3.2180618177768e-208 473.088266284513

UNCX -6.00431268950826 -5.66660726164281 -48.5629069836285 6.86905631592868e-211 6.25456198633289e-208 471.475943419768

KCNJ10 -7.31057759066957 0.250789826909116 -48.4957825811709 1.33703795549521e-210 1.16873161765747e-207 471.738136823191

GP2 -8.03012518290834 -4.31952147124563 -48.3730853570279 4.5234788582939e-210 3.80198398039602e-207 470.318212048196

ELF5 -8.58486393469314 -3.18609142024283 -47.9708536360856 2.49061878266638e-208 2.01583304657809e-205 466.47153220957

CA10 -8.27427940929861 -3.68976017567712 -47.6938859303883 3.98086432500412e-207 3.10692243193983e-204 463.664508102895

CLDN16 -7.1627200968619 -0.130010148448994 -47.4393411055073 5.1260822683711e-206 3.86276813140392e-203 461.19544334156

IRX2 -5.7229524794639 -0.532581808344667 -47.4072395725498 7.07932116173415e-206 5.15681351157921e-203 460.853120555261

NELL1 -9.05385520162576 -2.99147546730229 -47.1637090681654 8.23029716947489e-205 5.80182851756563e-202 458.40318066086

TMEM238L -6.94157746726788 -3.72592359593432 -47.0838245649684 1.84327704839212e-204 1.25878541682853e-201 457.375160437454

GABRA2 -8.77710503870088 -3.41870907985065 -46.7476238908427 5.53429364541407e-203 3.66487633434041e-200 454.183273580025

SCNN1G -9.3155225825981 -0.732402718405102 -46.6350329628295 1.73462258116569e-202 1.11490315488864e-199 453.072541755136

C4orf54 -7.93239553220527 -4.5369245671765 -45.9118947589886 2.76675456824792e-199 1.72748250228348e-196 445.508652874757

TMEM213 -10.1012113704713 -0.695706761113415 -45.7166452704035 2.04899966950544e-198 1.24379971604729e-195 443.706863781687

FAM3B -5.88612845392258 -0.748359441239732 -45.6581279777115 3.73728803044541e-198 2.20732311700874e-195 443.09727556888

GPC5 -6.51910962519824 -2.3690981575564 -45.5810916525171 8.2500405316398e-198 4.74442462468222e-195 442.245524646095

RALYL -8.28923794652074 -3.82514772397322 -45.5661432773316 9.62109463693111e-198 5.39102002822706e-195 442.101731575681

NRK -7.03642513269716 -0.830454275350655 -45.4089751326914 4.85244638880276e-197 2.65101277336267e-194 440.549033072304

PRDM16.DT -7.28932938375054 -0.859742673646534 -45.2300157857114 3.07434860178133e-196 1.63862780474945e-193 438.706038967479

RANBP3L -6.19329843979388 -0.181295799500132 -45.2095910952978 3.79639906777773e-196 1.97530259114635e-193 438.498350089944

NAT8L -6.4137620740574 -0.251670603620434 -44.6094767784498 1.91144538114399e-193 9.71414323584644e-191 432.285019299244

LOC100506444 -7.69970281866731 -4.33423679362542 -44.163866265399 1.99674265374914e-191 9.91700391190452e-189 427.478827372749

SLC26A7 -6.63619752907504 0.472962909195255 -43.8974445992311 3.25509897908868e-190 1.58074839977833e-187 424.856482882616

AQP6 -8.35346255895722 -1.62701216903358 -43.5674493079552 1.04572433559725e-188 4.96787258821887e-186 421.386184158252

SIM2 -4.41862725116076 0.858054139940567 -43.3466486048705 1.07389535356711e-187 4.99315641734086e-185 419.063586156155

GGT6 -7.86079301038553 -0.732024328122556 -43.2614887262026 2.64125163256992e-187 1.20248483180313e-184 418.163181696588

EHF -7.17920060797314 -0.524525792864516 -43.0766213886425 1.86909132268419e-186 8.33576585196278e-184 416.209130042422

CLCNKB -8.30297790478407 0.62944749586037 -42.9502387405652 7.13956794960457e-186 3.12041956805418e-183 414.871381829074

SLC4A1 -8.60585747487532 0.0818223509982468 -42.8555433147421 1.95142108131468e-185 8.36164801764112e-183 413.866687360691

FOXI1 -9.57478569339402 -3.26923354035785 -42.7709992763966 4.79325215534073e-185 2.01436421828194e-182 412.964783196708

RHCG -9.03933284437243 -1.01668263901663 -42.6941162654651 1.08613012072888e-184 4.47833991099777e-182 412.150875057022

ATP6V0A4 -9.18448605389008 -0.558703669455134 -42.5282514260432 6.35895290016175e-184 2.57337403198583e-181 410.386047085355

LINC02121 -7.0745416025347 -5.52987593829892 -42.4349493126053 1.72099320026128e-183 6.83797534641995e-181 409.020267315933

HEPACAM2 -8.47471053079073 -3.04773284704687 -42.3793285526215 3.11723839330524e-183 1.21644661801606e-180 408.789693128189

CLCNKA -7.79306665744344 0.240270769958074 -42.3039059524118 6.98037207391812e-183 2.67617668300584e-180 407.993627155434

ERVMER34.1 -5.90298193699808 -1.37779104913041 -42.1441337468009 3.85973224774402e-182 1.45425394499914e-179 406.265947809207

MFSD4A -5.02218537737348 3.82714150371543 -42.132623627261 4.36630431733763e-182 1.61723471604711e-179 406.141522477337

TYRP1 -7.71054384080167 -3.24757517335266 -41.8677916604739 7.48813992955773e-181 2.72730536467708e-178 403.295414533274

IRX1 -5.77811884885788 -1.44779966481861 -41.7152275501166 3.86489289076072e-180 1.38458203839007e-177 401.661591836555

SCNN1B -6.59270358166403 0.635280789988225 -41.6386206048474 8.82093333282039e-180 3.10909445358264e-177 400.85769742125

ADH1C -6.36858582583548 -1.35425107020806 -41.6175565063813 1.10690672731148e-179 3.83956074792663e-177 400.626430186844

GATA3.AS1 -6.8977907555902 -4.43667179428922 -41.5487999895639 2.32334291106891e-179 7.93312697431075e-177 399.688356485447

DMRT2 -8.38828014811522 -2.65024930831565 -41.5183562651275 3.22678662368872e-179 1.08484566288415e-176 399.561787356552

EPB41L4B -5.53706263468179 -0.228533460977962 -41.3271345868985 2.5466674765482e-178 8.43217035833451e-176 397.500863999512

SFRP1 -7.05281397993101 2.59805099055751 -41.2633353864765 5.07874944183163e-178 1.65650614257234e-175 396.802018733551

ATP6V1G3 -8.70382312103681 -4.32091458351394 -41.2517197210477 5.75918668443675e-178 1.85081627374995e-175 396.653633002486

POU3F4 -6.80667269842915 -5.18189954208996 -41.1828836257841 1.21368186682092e-177 3.84385359936777e-175 395.629781964238

RASL11B -5.48349558987884 0.113352732999616 -40.9251241299049 1.98912185849272e-176 6.20975428194876e-174 393.147688850526

PRR15 -4.72330610556694 0.351400924387285 -40.8968618659046 2.70428114604035e-176 8.32347265977745e-174 392.841354217139

NR0B2 -8.13511304398394 -3.8225006623831 -40.7601132251231 1.19696473714508e-175 3.63295422233769e-173 391.32727129753

FAM169A -4.46595836960436 -0.12544485995629 -40.7524462915843 1.30116265410226e-175 3.89511061371189e-173 391.267450358963

PLA2G4F -8.40217088110168 -2.46469937256254 -40.7439944038987 1.42658245446132e-175 4.21285221315448e-173 391.179811084576

LINC01606 -7.75817055856062 -4.07189728172147 -40.4572325403328 3.2550814335483e-174 9.48443927564415e-172 387.998967304178

FGF9 -7.8964671294565 -2.07785320943972 -40.3769113895548 7.83079805153206e-174 2.25166355026487e-171 387.179623086082

ADGRF1 -8.14090748672403 -0.564067387859903 -40.2716463862465 2.47727646776854e-173 7.03063930521374e-171 386.028364113808

SERPINA5 -7.25637928965705 1.99022806638204 -40.2435630701518 3.36910127029065e-173 9.43909872559762e-171 385.710888305393

SLC4A9 -6.60233260013078 -2.1193266113803 -40.0384140505965 3.19385435723131e-172 8.83484800868049e-170 383.46693581394

FAM245A -6.38718571895258 -4.7142638445855 -39.8295789009798 3.16956914783725e-171 8.65807432346094e-169 380.940115888398

CASR -7.5894053391232 -0.0943697806304088 -39.7197901646132 1.061499553861e-170 2.86382095685487e-168 379.972769368583

TMPRSS2 -7.29380853416409 0.298429854000344 -39.7186008007408 1.07549880248025e-170 2.86620430860988e-168 379.957647615339

FRMD7 -7.07213491336232 -4.54960430152727 -39.6012039912502 3.92310953154027e-170 1.03291219991264e-167 378.529092009433

OVCH2 -6.66815768274507 -3.01240399452977 -39.3932666030945 3.898696885437e-169 1.0142645599697e-166 376.3396126393

CLDN19 -7.35667913144419 -0.265723569407221 -39.2773696114812 1.40533796036227e-168 3.61304122915255e-166 375.092443294423

BRINP3 -6.89869637043667 -4.70098330597696 -39.2459861383594 1.98927976324078e-168 5.05485240303497e-166 374.579404143268

TMEM45B -4.57770184791182 1.44454280598164 -39.208024969392 3.02908835071125e-168 7.60858249748195e-166 374.312748634704

LINC01762 -5.06521821873035 -2.1444237389018 -39.0690446157777 1.41432603938991e-167 3.51218942486223e-165 372.720287000497

LHX1.DT -7.62114208327508 -3.00078353791828 -39.0631123526112 1.51056146269195e-167 3.7090224319334e-165 372.72004683068

MCCD1 -7.37815448414521 -2.76573876752436 -38.9497941980355 5.31623787757461e-167 1.29084162598487e-164 371.464959049907

ATP6V1B1 -6.67884787800817 0.887182234012684 -38.9246231103449 7.03206230692596e-167 1.68869953399179e-164 371.175196778494

SLC12A3 -8.36973303545117 0.127810416581498 -38.845159309716 1.70139551936094e-166 4.04136916136898e-164 370.300325878694

NHLRC4 -3.52284424519959 0.393048441656187 -38.7648561268572 4.15837237348938e-166 9.77128080407134e-164 369.413127742586

SLC2A12 -4.55096160116304 -0.26538330074557 -38.7623559476278 4.27575350586124e-166 9.94021716633891e-164 369.386295853564

LINC01976 -5.18345183106682 -5.53979810339516 -38.686988657815 9.89914047365825e-166 2.27711491337741e-163 368.090028961429

PLPPR1 -7.24682519527319 -1.87558530726933 -38.528784766357 5.77940165877762e-165 1.31559650467987e-162 366.786337828274

VTCN1 -6.47763931734122 -0.416181784755289 -38.5204315020986 6.3442488888844e-165 1.42928732957516e-162 366.688077354993

EPN3 -6.18814683711415 -1.21724405643945 -38.4876682152333 9.14650525029478e-165 2.03957733912951e-162 366.328057477849

SIRLNT -6.55150227810918 -5.20957063576373 -38.4030578076542 2.35401731229839e-164 5.19619599249057e-162 365.141445536281

HELT -4.80358785945081 -5.72444721402469 -38.1243411980269 5.33221139964522e-163 1.16524815716447e-160 361.774169673441

PTGER1 -6.60863094661264 -2.74700161962399 -37.9372977233423 4.35110593536939e-162 9.41432851540864e-160 360.154382296121

GCGR -7.83105075620009 -3.25022357995325 -37.7626906380879 3.09982496124873e-161 6.64122302727143e-159 358.208552605162

INPP5J -4.98199065887756 1.17909166737916 -37.7570717017634 3.30221443854254e-161 7.0061448665505e-159 358.122864856686

PROM2 -5.95958825372608 2.00252464750122 -37.7421906913972 3.90444146472605e-161 8.20420762775562e-159 357.945259177648

AQP5.AS1 -6.42211347110125 -5.20580260008698 -37.704575980992 5.96362092649042e-161 1.2411715057771e-158 357.329510774475

KLHL14 -4.31831586492111 0.483148298926528 -37.6919968763427 6.87137249959241e-161 1.41660474748673e-158 357.410540057304

ERBB4 -6.107474537519 0.284951922681353 -37.4910850330417 6.62173272469638e-160 1.35238060965224e-157 355.136468507588

LINC00645 -7.52910829345257 -2.24039710520034 -37.4866613487574 6.96080674189077e-160 1.40846768269018e-157 355.10284437781

SMIM5 -4.73755928888932 0.836238133189727 -37.4725835856793 8.15990709560238e-160 1.6359490803688e-157 354.926471800062

LOC105371267 -4.42093169685069 0.372335249689226 -37.2421374123604 1.1042601187048e-158 2.19376330673235e-156 352.337355619308

FAM167A -4.32842601210057 1.1013935349408 -37.2324696619929 1.2319585870846e-158 2.42540459491529e-156 352.21232933389

RHBG -7.43586658123336 -2.69514763170569 -37.0409651269502 1.07893277077567e-157 2.10517123569292e-155 350.067556371954

ARMH4 -3.83280157413139 2.08027791161477 -36.9864574558175 2.00251370860563e-157 3.87264885612025e-155 349.405573630559

MYO3B -5.51361896203193 0.180467504864554 -36.960703264003 2.68244106461293e-157 5.14205127938477e-155 349.142591133797

SLC15A2 -3.59167472012061 1.89192974677509 -36.9029269628584 5.16965857524024e-157 9.82369989954129e-155 348.465592827682

CHGB -5.46002381707035 -0.234179848811768 -36.8422097750063 1.03058228782833e-156 1.94149264964763e-154 347.805669906006

RAB25 -6.990083533835 -1.07806227007641 -36.7362009760056 3.44079944837736e-156 6.42664874746926e-154 346.601574627059

PRDM16 -4.3574072175677 1.30874439333415 -36.673102501174 7.05629667845996e-156 1.30679026537615e-153 345.861102003531

GRHL2 -7.00495094987392 -1.74445797890144 -36.6187176236831 1.31094393269987e-155 2.40739981187313e-153 345.271647311534

PRRG2 -3.64002169657193 0.30854060076587 -36.5738732767728 2.18531801322917e-155 3.97964621192475e-153 344.764041425043

VGLL1 -7.11325421200502 -4.87106734761388 -36.4269719527789 1.16744632552085e-154 2.10844665715762e-152 342.984011446471

HCRTR2 -4.63487743343334 -5.74420562098872 -36.2620274976712 7.68519168663004e-154 1.37659421252399e-151 340.797404304463

MAPK4 -6.92525102222417 -2.28017675318359 -36.20672118886 1.44671286917434e-153 2.57032653089974e-151 340.577634986417

KLRG2 -6.42993153046236 -3.07797051378257 -36.1739569867648 2.10478613401136e-153 3.7093460795605e-151 340.179415557427

GPC3 -4.77332853786687 2.56022725350117 -36.0903479656182 5.48226126494429e-153 9.58430843382621e-151 339.182960840086

LINC02343 -5.84874303055449 -5.40321097119248 -36.059091279585 7.84290071703725e-153 1.36024531245567e-150 338.641383308057

LINC02944 -5.22299654905101 -5.47747941721304 -36.0069990770285 1.42485332034764e-152 2.45175744957142e-150 337.980086150546

SYT7 -4.65657724691454 0.687487100815549 -35.8634060603047 7.39989831001354e-152 1.26335920131817e-149 336.623846900471

ATP6V0D2 -7.49692247146634 0.0261981513296823 -35.7433837539437 2.93789051518904e-151 4.97687763011055e-149 335.245194820594

WNK4 -4.52804895146259 2.25528337530193 -35.6325127509018 1.05154966296365e-150 1.76765498344189e-148 333.934077234594

SPTBN2 -6.36402567579318 2.31265922197974 -35.5992231495433 1.54251488140325e-150 2.57317387048131e-148 333.559968137855

ACOT12 -5.82134796171304 -4.10733055626791 -35.5147664087362 4.07979358236204e-150 6.75422190570892e-148 332.53676781104

TMEM52B -6.16442897015459 1.44007994197548 -35.4732940788451 6.57947602890108e-150 1.08106232826748e-147 332.11880514983

TMEM178A -3.65488502770448 1.28758795549184 -35.4263892321321 1.12988725913308e-149 1.83586894499468e-147 331.597483069156

SLC9A2 -6.40986587619582 -1.26092683649062 -35.4260638983837 1.13413402084053e-149 1.83586894499468e-147 331.61678165269

TMPRSS4 -6.27825166273992 -1.06324991321591 -35.4251890526073 1.14563327568739e-149 1.84084735099975e-147 331.605220008251

BMPR1B.DT -5.24295897514634 -5.46494775256631 -35.4171614619046 1.25675230956114e-149 2.00465753436786e-147 331.233824493076

TNNC1 -4.36380708566129 -1.41883833501353 -35.1531000612244 2.65160672852508e-148 4.19895375641003e-146 328.463536643185

GATA3 -4.01973470717747 2.61694907761332 -35.1413112342044 3.03885444204946e-148 4.77756015266956e-146 328.262556479988

BMP7 -6.5585970034676 -1.97415126302799 -35.1068063148155 4.52923595294583e-148 7.06981380569466e-146 327.941378845181

PCDH9 -4.0306538525391 0.908248620647315 -35.0712681503284 6.83267876942659e-148 1.05896829183177e-145 327.504091415455

ADGRF3 -3.45932158209654 -0.397911313278403 -35.0102738578707 1.38423227877873e-147 2.13025549212335e-145 326.822883422317

EGF -6.60072983154879 2.3696495968557 -34.8391132768176 1.00601222243113e-146 1.53736958718794e-144 324.783993485671

LHX1 -6.90975881085869 -2.47099070630006 -34.6020146798893 1.57831422862001e-145 2.39520144708563e-143 322.098210264148

CCDC181 -3.63999070460293 -0.301725637840526 -34.594656678365 1.71925503654201e-145 2.59109519403811e-143 322.013949479062

SUSD4 -4.66536768667585 1.08200893077367 -34.4910494624555 5.73690191073445e-145 8.58688475721096e-143 320.761141813121

CHL1 -4.85611648484817 1.75985525098012 -34.4393160552782 1.04757306381605e-144 1.55732069139946e-142 320.140348130329

OLFM3 -5.18905501466292 -5.45073867269282 -34.1633330753598 2.61461465502347e-143 3.86061986866404e-141 316.761239392403

LOC101928596 -5.36827315975158 -4.57526563570874 -34.1033907485074 5.2644938182081e-143 7.72113982612762e-141 316.156150600939

LRRC2 -4.82192343311198 -0.126939838186098 -34.0912128043576 6.06915091825688e-143 8.84194366777784e-141 316.1393035081

FLRT1 -4.75233301268483 -1.68873185224149 -34.0629360286577 8.44473118174901e-143 1.22213715572689e-140 315.820193782778

SLC52A3 -3.80407281029248 1.23355825237347 -33.9297220959917 4.00804274064138e-142 5.76235250073922e-140 314.226506697638

PAPPA -3.78662796289314 2.11945401220787 -33.8250436972201 1.36453030528109e-141 1.94895952688285e-139 312.96789323806

CLUL1 -3.74769714030055 -0.682910349233758 -33.8153358998621 1.52880150755541e-141 2.1694090483512e-139 312.934576938212

MPP7 -3.30110901782705 2.81495800599855 -33.7890621670036 2.07962602516557e-141 2.93200435664149e-139 312.527700420836

TDGF1 -6.52642125972779 -2.02122896758362 -33.7288463408317 4.21092619904203e-141 5.8988057838247e-139 311.920553157636

S100A2 -4.16285570548704 1.90832305260381 -33.6292493710062 1.35369110254471e-140 1.88421730343373e-138 310.67844675877

TSPAN8 -5.98257390538555 0.174034367761925 -33.5947742077204 2.02848545455386e-140 2.80560079989655e-138 310.307391282853

C1orf226 -3.26078549701669 1.63385318390313 -33.5662020926642 2.83654365407578e-140 3.89855273412064e-138 309.965482705075

ATP6V1C2 -3.4341305109487 1.3189570379698 -33.4371320334157 1.29140208908271e-139 1.76381311579527e-137 308.461034508216

COL26A1 -5.92757295389116 -2.3804146022165 -33.3803195398017 2.51798644233843e-139 3.41773650462247e-137 307.840823235198

L1CAM -5.69917651980374 1.92282222026721 -33.2690889497276 9.31624548496909e-139 1.25671550977179e-136 306.449001947052

F11 -5.93555784526389 -0.815147492371251 -33.229372173785 1.48681854320259e-138 1.99334022236848e-136 306.043045402622

TMEM30B -4.25760875752927 2.21597565225521 -33.1538070228093 3.62005437932663e-138 4.82372246045274e-136 305.083126267279

GRIK5 -4.67738000096813 -0.0195708316229932 -33.0821631955997 8.42083531256094e-138 1.11527584294178e-135 304.311506792772

ESRP1 -6.66947074597696 -0.853259227496821 -33.0302934858353 1.55218743800877e-137 2.04337060739793e-135 303.690833567153

FAM81A -2.80575945267937 1.64851162205012 -32.9349475445475 4.78033721033052e-137 6.25537179984149e-135 302.556091944355

CALB1 -7.75016881496172 -0.280838892906596 -32.67834364807 9.9127912937117e-136 1.28942992941358e-133 299.523978327293

SLC13A2 -7.01108110356757 -1.63934006243613 -32.6081328598437 2.2749928086441e-135 2.94174070102364e-133 298.718727063087

SIAH3 -5.34824827401371 -2.94624504217372 -32.5499795649118 4.52864663440885e-135 5.82144205304333e-133 298.041281709195

FAM83B -6.92858552504674 -3.15641703713843 -32.4837670729052 9.92135762613045e-135 1.26790308891128e-132 297.27949011766

LOC102723475 -5.14080376199223 -5.02987032392058 -32.45172990417 1.45024423468095e-134 1.84256902677226e-132 296.753650053083

LOC105374428 -5.8362906885067 -4.79833177559585 -32.1233864071079 7.14008775033797e-133 9.01921026636622e-131 292.929078116734

NIPAL1 -3.71929124584034 1.08490111588928 -32.1154282656321 7.84831163564238e-133 9.85684794101683e-131 292.857557638569

CALCA -7.23407761191352 -4.03117901036619 -32.0784703860786 1.21773897154762e-132 1.52064284258458e-130 292.474622461154

SLC4A11 -4.03797030090539 0.96650005740681 -31.9538852315984 5.35907759088317e-132 6.65408651099829e-130 290.936194845412

TFCP2L1 -6.12892226076569 3.10399882988109 -31.9523686804222 5.4566699115241e-132 6.73698347890035e-130 290.86255537174

DNER -5.47024773780192 0.243895816809434 -31.944234332858 6.01129681126929e-132 7.38004883239707e-130 290.819838641596

PROX1 -4.52119610549062 0.847261503033172 -31.9094491640048 9.09448978428827e-132 1.11028986176565e-129 290.400100667346

TJP3 -4.10559395681854 0.527143268007195 -31.9058460714903 9.49305910167943e-132 1.15251011416111e-129 290.383748687158

SCN2A -4.62181756969801 -0.680718782411481 -31.8656837024709 1.53137138153885e-131 1.84889827628555e-129 289.939508820798

CST9 -3.8765504664025 -5.93755824207055 -31.864319235769 1.55645718975643e-131 1.86886038284326e-129 289.685855635655

NUPR2 -6.77522367116698 -4.10983732185794 -31.8187567238387 2.6780484169809e-131 3.19799956591714e-129 289.3834297661

OXGR1 -5.73501017044574 -2.61104987878951 -31.7417864695132 6.70126903387616e-131 7.95884957593999e-129 288.477986569286

LYPD6B -5.98978673434131 -2.07157447409 -31.6295072005793 2.55660437473728e-130 3.01997164330453e-128 287.137272144377

LOC340268 -4.81095993107736 -5.42893040390593 -31.6187875866567 2.90541689886215e-130 3.41355244574379e-128 286.840126513156

MFSD6L -5.21895350012467 -2.42806485721914 -31.6162144574734 2.99600216333013e-130 3.50115696659109e-128 286.980900801188

LIPH -5.1294822198422 0.393484416281429 -31.3922422615198 4.34675896859399e-129 5.05264487982364e-127 284.242508432361

PSKH2 -4.1687712691343 -5.89366958281532 -31.3289109170414 9.26820882048409e-129 1.07163051510073e-126 283.339455012277

CRISP2 -4.96358959925044 -5.56588389718954 -31.3123868212213 1.12932453086295e-128 1.29890152489201e-126 283.20087271593

HOXB9 -4.56489211531657 1.58713375184063 -31.277679461311 1.71047714443662e-128 1.9570186930562e-126 282.83720440251

EDDM3A -4.73887910369239 -5.27653799125352 -31.220593756887 3.38665317164744e-128 3.85461102916726e-126 282.11158077992

CACNA2D2 -2.38466119632535 1.06940187248723 -31.2162006581966 3.56948334985324e-128 4.04165386758253e-126 282.190973581435

GRM1 -4.63170732350247 -1.89385119173717 -31.2136217221525 3.68137974746539e-128 4.14686554749284e-126 282.181320508987

PCP4 -6.67329573310959 -2.09373141276205 -31.2078061035478 3.94676299485076e-128 4.42300572956275e-126 282.090389943415

FAM222A -3.17213336241184 1.26163219208327 -31.2041695708834 4.12235080348628e-128 4.59621082186661e-126 282.007382998606

SRGAP3 -2.53350560594824 2.40597577513775 -31.0778733720104 1.87066169060252e-127 2.07510507232166e-125 280.448884980283

HYKK -1.97333477992109 1.46507975990868 -30.8717206187328 2.21562333201214e-126 2.44535437749804e-124 278.062267755508

RNF150 -3.64185087773835 3.10138284023123 -30.6889611160433 1.98854140760519e-125 2.18369826032142e-123 275.738274186855

SCHLAP1 -3.91580530446874 -5.78072112601676 -30.667022744498 2.58834043771602e-125 2.82815017927041e-123 275.443536006196

BMPR1B -5.67988573294259 0.0173052355930004 -30.6523880908503 3.08605197152167e-125 3.35519869321707e-123 275.386338760159

ABCA4 -4.51849763595447 -0.10148166490007 -30.4765703751607 2.55657871058364e-124 2.76578784962298e-122 273.313800753378

FER1L6 -5.08144319427103 -2.17719157510735 -30.3487263993311 1.19143492053257e-123 1.28258262652209e-121 271.819690269099

WNT9B -4.46854011349147 -1.3624586189652 -30.2867499286408 2.51371934206231e-123 2.68342113344585e-121 271.073039595919

CWH43 -6.23966059236361 -1.57229601636512 -30.2866324323543 2.5172806129886e-123 2.68342113344585e-121 271.035805244707

ATP1A1 -2.82051116851255 9.60132233875792 -30.1933055372882 7.75275261700679e-123 8.2243156766723e-121 269.817750884426

TCF21 -3.90292072118462 1.75603960715402 -30.1656402933775 1.08225724982046e-122 1.14253950146505e-120 269.488939218255

PRKAR2B -2.31536339770356 2.72196999397885 -30.1555162409323 1.22279566699804e-122 1.28469969763982e-120 269.355080426797

CPAMD8 -2.92780979816597 2.95727426238259 -30.1304058261109 1.65532005214385e-122 1.73079947844496e-120 269.025547979905

CEL -4.22647846814658 -1.06611202027988 -30.1220908385537 1.82995616057447e-122 1.90428723700161e-120 269.088204873021

HSPA2 -3.86792442160354 4.24582091787788 -30.113138222634 2.03864862444795e-122 2.11140229336783e-120 268.790313395132

USP44 -3.21793561111548 -1.24475707696456 -30.0983959721415 2.43546230787713e-122 2.51047914217165e-120 268.804112499465

GAL3ST3 -6.04910332540941 -3.00838594607006 -30.0534547885763 4.18879011475841e-122 4.29754133229181e-120 268.267575362753

STAP1 -4.58404400022638 -0.487096666001721 -30.0440283099145 4.69348596968312e-122 4.79283873343389e-120 268.121835043851

PCARE -5.22526328989188 -5.27457809935637 -30.0355051514736 5.20200239566466e-122 5.28741201639348e-120 267.962457671354

ABHD17C -2.17359115003575 3.02256171571617 -30.034836247704 5.24417026567192e-122 5.3055950377652e-120 267.885715198284

ERP27 -3.72093025174076 1.83915931950656 -29.9783494809995 1.03710599163905e-121 1.04441830577364e-119 267.230779338373

TMEM61 -6.38971741171179 -2.90671876216403 -29.8921261889464 2.9382126606301e-121 2.94535602168576e-119 266.319555625245

LOC101928004 -5.27466643307542 -2.54426776416533 -29.8541811588323 4.64722764585759e-121 4.63725414360392e-119 265.866496271197

TRPV5 -5.1798351986101 -4.02497423730612 -29.8214247812486 6.90426458972046e-121 6.85813154905279e-119 265.441659755076

SH3GL2 -5.8879245876637 -1.29074350551916 -29.7618916412973 1.41798969141417e-120 1.40214157133366e-118 264.708464221514

PVALB -6.75310548742195 -1.93275491911709 -29.6629260107106 4.69371853526654e-120 4.62035275455764e-118 263.51165134205

AMPH -3.5027930820504 -0.353903131341495 -29.5819795938123 1.25012118907209e-119 1.2250627060445e-117 262.571525809532

HOXB.AS3 -3.59972182708589 0.0260204562571871 -29.5254990559402 2.47701111392253e-119 2.41652338716737e-117 261.869431611268

KCTD8 -5.02076669506965 -4.24733113868891 -29.4941230393508 3.62200184850148e-119 3.51784917312457e-117 261.479521460423

PAPPA2 -5.62566099447528 0.562040636114324 -29.3910935943362 1.26197625046683e-118 1.2202640266129e-116 260.156073263362

C16orf89 -4.33216362926707 0.570641578714792 -29.3819013279309 1.41069792019268e-118 1.35806086563747e-116 260.070570906916

CNTN1 -5.81587965232007 -0.133757863302483 -29.3657961509114 1.7147822899671e-118 1.64355865713382e-116 259.872012105858

RIMBP2 -4.34003776260409 -1.47553628497877 -29.2421881281073 7.67599456572743e-118 7.32504407182714e-116 258.471282119313

CDH3 -3.89054343728833 1.04849780697996 -29.239386291283 7.94135913602403e-118 7.54532700867536e-116 258.332343543473

SLC16A5 -3.05054443581398 2.82407795317906 -29.218557034833 1.0224258134266e-117 9.67232523844651e-116 258.004762546083

MTURN -3.23672811169858 5.1205488922561 -29.1814484866976 1.60386632090297e-117 1.51074528925399e-115 257.513499876595

TNNI1 -5.1221905749534 -1.1869947958232 -29.1585916710799 2.11655239253216e-117 1.98510813021482e-115 257.429263511367

LINC01612 -6.01437950644791 -5.05500592728175 -29.0324914234578 9.7835369345472e-117 9.13673643720769e-115 255.901905010142

ENPP6 -4.41934984707133 -0.413081440461764 -29.0196073405349 1.14407588670356e-116 1.06389320647374e-114 255.728325735259

LINC02894 -5.66333461705429 -4.1739439690875 -29.0179670847887 1.16709701053095e-116 1.08070215979376e-114 255.743120754694

MRAP2 -4.47272166618764 -1.58856603755873 -29.0060813051941 1.34835808473697e-116 1.2432771825214e-114 255.610947050563

AVPR2 -3.92048315444341 0.139591199641848 -28.994197539827 1.55774786386681e-116 1.43031361634796e-114 255.415895333149

SRARP -5.44136869915998 -4.25481810257651 -28.882603618457 6.04517464268513e-116 5.52741428730536e-114 254.097602029409

ODAM -5.91837502794206 -5.20299553722156 -28.8060140238165 1.53393334488373e-115 1.396710224406e-113 253.15064480625

PEG3 -2.67574945585819 2.14714130773057 -28.7602706229045 2.67556888851969e-115 2.42610817098841e-113 252.490105427311

TNNT2 -5.29105287107157 -2.01686870236988 -28.7426235826447 3.3162046110226e-115 2.99458757705276e-113 252.405453658258

RALGPS1 -2.34264160935605 2.60509025172197 -28.740519625973 3.4021748457797e-115 3.05957723888164e-113 252.230905224795

FREM1 -4.93587234047425 1.05226775903662 -28.7276655136729 3.97803253906942e-115 3.56278463427393e-113 252.095848950907

RBBP8NL -5.61652731826521 -5.02301747046316 -28.7017824483595 5.4504188726821e-115 4.86155116835599e-113 251.884244138821

ARL4D -3.36344006320039 2.43679109368869 -28.6949127585122 5.925586393665e-115 5.26389591303908e-113 251.660023844177

F11.AS1 -5.36765956080006 -2.78623097522637 -28.5536503353101 3.30750354562378e-114 2.92627024220714e-112 250.125011420006

PPP1R1B -5.49463233623343 -2.71089728429697 -28.5248368347872 4.69773659809067e-114 4.13950152734175e-112 249.77299764939

MAP3K15 -4.19566082249433 -0.874584649871437 -28.5228398560976 4.81339057529018e-114 4.22326901923561e-112 249.731399557479

MPPED2 -3.16102721454567 1.73162462739334 -28.5225323288543 4.83145222536449e-114 4.22326901923561e-112 249.609432750782

FECH -1.87108419200775 4.93826150471703 -28.5220495773115 4.85994198342442e-114 4.2312474965647e-112 249.486865926073

CCNI2 -2.96835721219905 -0.777191817555598 -28.4008020215357 2.12884067502699e-113 1.8460934631494e-111 248.267460419148

B4GALNT3 -3.33075280195755 1.95195821969129 -28.3351617421121 4.7381396177509e-113 4.09259150461306e-111 247.30875571933

TAFA4 -3.88140320656591 -5.46467269220798 -28.2600317771503 1.1842602690123e-112 1.01888345113094e-110 246.44711984735

LMO3 -3.74349917909829 0.0872469657397213 -28.1473808823609 4.68005097713388e-112 4.0107119216983e-110 245.131552358686

CHL1.AS2 -5.37394886227295 -5.05364995041009 -28.065010808162 1.27890662580721e-111 1.0917166599127e-109 244.145272972756

DBT -1.55030092732611 5.04722934856232 -28.0582119168976 1.38957783076649e-111 1.18157370956187e-109 243.831994496591

MAP6 -2.72888626489602 1.84559859281571 -28.0552392291315 1.44092980221167e-111 1.22048988247022e-109 243.924562496441

CASZ1 -2.18216047363147 2.25811255326724 -27.9665545779356 4.2551368494662e-111 3.59025118036235e-109 242.838627587652

MPPED2.AS1 -5.00119249814973 -4.96647892192779 -27.9530010700673 5.0211284552362e-111 4.22025846662603e-109 242.773568360061

FOLR3 -5.39289754303878 -2.68532463290333 -27.9154681653885 7.94144280609296e-111 6.6492087985268e-109 242.357773147393

NOS1AP -3.25704584545621 -0.504920171499535 -27.8855836368346 1.14406399500844e-110 9.54245438279367e-109 241.983988355409

SOST -6.59557043955835 -3.84763318830423 -27.8480817315558 1.80902872639793e-110 1.50314466760357e-108 241.534867475471

LINC01665 -3.41676402089861 -6.08217099054694 -27.8179192945273 2.61529067228484e-110 2.16484647960002e-108 241.061741675758

KSR2 -4.24350560622459 0.0190333183107261 -27.8146701939971 2.72122531819106e-110 2.24403535390299e-108 241.052605229824

NKD1 -3.32890405518121 -0.266332479781247 -27.7231233408587 8.33202116455301e-110 6.8450999439465e-108 239.988526859475

SOSTDC1 -5.16457148849575 0.328335980341342 -27.702083953393 1.07762555403826e-109 8.81998173498057e-108 239.624491991701

GALNT3 -3.09998452800793 2.25557035123534 -27.6734884304259 1.52870729249282e-109 1.24652389786737e-107 239.223202927058

CA8 -3.68752640666842 0.817135477133258 -27.6178750192799 3.01795668652911e-109 2.45172518478516e-107 238.61964046237

SSC4D -2.634487075327 0.62413898548097 -27.5075659777025 1.16364965692697e-108 9.41823553808337e-107 237.339995171534

PPP2R2B -2.48454869013628 1.24735047978172 -27.4766399492622 1.69899132462228e-108 1.37003901907641e-106 236.923750546361

TUBB2B -3.28712162637436 0.647490615609173 -27.4660321549529 1.93452972168765e-108 1.5542381620603e-106 236.793381352608

ALDH3B2 -5.30493303287409 -3.97738338483363 -27.4203423217423 3.38424069213949e-108 2.70900409689833e-106 236.316507260577

TCEAL2 -5.81367287663061 -2.03136935830241 -27.407568279989 3.95708825920988e-108 3.15599451563918e-106 236.126148180636

CRHBP -4.22644183825644 0.579299407443711 -27.400821802202 4.29779290878884e-108 3.41526067039137e-106 235.954044882646

SPTB -2.85952484670159 0.857982584542568 -27.3947258010595 4.63081689255495e-108 3.6665667229349e-106 235.927063510076

OLFM4 -6.01775218916785 -3.07538862849113 -27.3371863031886 9.36765852804824e-108 7.39030475860788e-106 235.29578576961

NEXMIF -4.78214304042872 -1.13016572667456 -27.2895147383146 1.67951251004023e-107 1.32022974395357e-105 234.682290121057

LYPD6 -3.97530878130609 -1.23149026973664 -27.2756912716707 1.98936907534862e-107 1.55819650192091e-105 234.543441213838

ANGPTL1 -3.86135235338351 0.992641285103994 -27.2732352365361 2.05012495614397e-107 1.60004930952193e-105 234.38370561545

CYP2B6 -5.95545014755344 -2.76569480452383 -27.2315084560435 3.41795548931861e-107 2.65809897893522e-105 233.994643444294

ENTPD3 -4.03614024085051 -2.14149347540484 -27.2116710308855 4.35826631378483e-107 3.37734729628155e-105 233.773356467972

TCF24 -4.58624188760517 -5.03173258580462 -27.1953331453594 5.32411454141624e-107 4.11123233475509e-105 233.529561052957

PRSS22 -5.68737604885621 -3.04874160333722 -27.1802508882078 6.40478572869792e-107 4.92830220173365e-105 233.385198630284

TAGLN3 -5.29514394582905 -3.03765153991367 -27.1481636970705 9.48998009050325e-107 7.27665034799185e-105 232.996249648266

OVOL2 -5.61236449112322 -3.73463962271085 -27.1099682169273 1.51551243233995e-106 1.15798927216521e-104 232.530470813647

DIRAS1 -4.43168459128415 -0.95495973048867 -27.0733005629482 2.37546578211627e-106 1.80874751695425e-104 232.041199675644

ANXA9 -2.05356797399448 3.35642108031391 -27.049033856798 3.19845058670968e-106 2.42693543997801e-104 231.542657272538

KCTD1 -2.31724210207236 3.38060886973312 -26.9961202843519 6.11898656309358e-106 4.62692779803751e-104 230.887315540942

HTR3B -4.36059759573903 -5.63816364135079 -26.9409020501115 1.20433375716917e-105 9.07527779152337e-104 230.395680946402

ANKRD2 -4.78280792230286 -0.993124137119324 -26.9377781877087 1.25136739415417e-105 9.39729610462236e-104 230.373252489559

LINC01020 -5.53739162872693 -4.43678771098128 -26.7992898818785 6.84170618022936e-105 5.12026729988193e-103 228.726949364044

SIX4 -4.24734473468883 -1.3645611500001 -26.7928712844542 7.4023057631657e-105 5.52090743489625e-103 228.63380841301

ANKRD34B -4.92952575126434 -4.04366043075961 -26.7911750776999 7.55797882091793e-105 5.61784051610611e-103 228.624412919268

TPPP2 -4.46506698345413 -4.18455988815355 -26.7205068033732 1.79896682391883e-104 1.33263803400333e-102 227.74947021713

KCNK13 -2.80747119752069 0.918976451678199 -26.7170663943123 1.87655182999364e-104 1.38541510610983e-102 227.63379621115

LRRN2 -3.63169283123445 1.72164008878405 -26.715065586618 1.92320234058293e-104 1.41507544608615e-102 227.507954440829

VWA5B1 -5.2307313702123 -2.87789367776535 -26.6849849543803 2.78204772754083e-104 2.04013721442784e-102 227.328104557777

RBP2 -3.90654763563459 -3.45484871255623 -26.6756192631657 3.12097207943446e-104 2.28102350675188e-102 227.205456646774

LSAMP -5.11806552954019 -1.44072842138845 -26.6501544000128 4.2662057593136e-104 3.10764648194267e-102 226.860264238703

SOWAHA -4.78108823830325 -0.240606540732008 -26.6012688351597 7.77460485831503e-104 5.6444664441448e-102 226.198647008149

ZFHX2 -1.8157487003159 1.44569682432401 -26.5946912582614 8.42849588247588e-104 6.09893776555448e-102 226.147419445999

PLEKHD1 -3.64185778394408 -1.65954282328693 -26.5735403557638 1.09277763059381e-103 7.88134308955989e-102 225.965465445915

ERMP1 -2.48855313803493 5.73838262003894 -26.5690979523308 1.15403951200082e-103 8.2957978472875e-102 225.616671927394

NXPH2 -5.86864439560554 -5.15421758430226 -26.5232773884966 2.02569829424682e-103 1.45139622374347e-101 225.343698493052

MIR200CHG -5.3258248847614 -5.13695113618241 -26.5164022115797 2.20415379921449e-103 1.57409715602073e-101 225.248107278135

AGR2 -6.18803406985867 -2.80342017644883 -26.4935631661663 2.91778879956325e-103 2.07695239859465e-101 224.958209263268

RAB11FIP4 -2.10738753135859 3.89755226570914 -26.4802060772616 3.43793441074252e-103 2.43925911292066e-101 224.539537393739

C1orf116 -4.80966687237947 0.613512356477973 -26.4233312121441 6.91330982510596e-103 4.8892090488039e-101 223.959867806084

ADGRV1 -3.13628647874791 1.04836593109922 -26.3953742954101 9.74619069935362e-103 6.87043565654757e-101 223.6561696475

CDKN2B.AS1 4.03826105197212 -1.48173080802755 26.3327536602824 2.10356032894433e-102 1.47810623371127e-100 222.947289836601

BUB1B.PAK6 -4.68763550233917 -4.19827268807564 -26.3171857184763 2.54699712546553e-102 1.77825968635138e-100 222.818037064806

PAK6 -4.68763550233917 -4.19827268807564 -26.3171857184763 2.54699712546553e-102 1.77825968635138e-100 222.818037064806

FGF1 -3.84854254997923 2.6641254619018 -26.3132071653171 2.674602975564e-102 1.86140442117835e-100 222.526952875122

PIP4K2C -1.41677883216736 5.40104148779584 -26.3079999085186 2.85132879291572e-102 1.97809803528848e-100 222.398174515741

FMO5 -2.64915755594847 2.74209713706595 -26.303132076058 3.02708295211527e-102 2.0933811314106e-100 222.41459705308

SEMA6D -3.07331467623447 3.19762309667343 -26.2966837412219 3.27669606220177e-102 2.25885296679165e-100 222.30607613324

KLHL13 -2.43446603423391 3.42269329817468 -26.2921348910113 3.465067205961e-102 2.3811985425115e-100 222.246514422439

NAALADL2 -2.10913899907588 2.66145171618067 -26.2690432617497 4.60200928405406e-102 3.15259275499791e-100 222.024072349363

SLC25A35 -1.69942816505299 2.13332559210439 -26.2536568688855 5.55988749322518e-102 3.79688191842031e-100 221.909916465926

GMPR -2.78108941400584 2.62928558050585 -26.2161050677097 8.82057536573567e-102 6.00486085568292e-100 221.35128002364

CCSER1 -3.05067663644053 0.749032867799416 -26.2016315546011 1.05378912668795e-101 7.15169372220859e-100 221.30818714925

TACSTD2 -4.87679457269458 3.79150387602 -26.1884274241484 1.23947351978751e-101 8.38582502412273e-100 220.978029203849

LINC01555 -4.03563585417729 -5.41206992029958 -26.1767938735353 1.43000969396067e-101 9.6450622969514e-100 221.054675728198

COBLL1 -2.64614541293019 6.12650449600573 -26.1113747863869 3.19587315065629e-101 2.14890510650129e-99 220.00428980001

GSTM3 -3.53427117507121 4.78409226922794 -26.10588006344 3.41921600950486e-101 2.29202844956165e-99 219.937363280449

KLK6 -6.55492096636762 -3.78226172788785 -26.051535678153 6.66958018352276e-101 4.4571968119426e-99 219.554993571731

HSPB7 -3.91464582806511 1.49432128282637 -26.0412101613699 7.57243899823245e-101 5.04513748257237e-99 219.242653232745

REEP6 -3.02436784448596 1.26156462610727 -26.0340538317524 8.26895594414204e-101 5.49244663365763e-99 219.207729580814

CNKSR1 -3.73555404833133 -0.0199681656294851 -26.0118935600129 1.08590238626046e-100 7.19097722634842e-99 219.004583298615

LOC112268292 -3.4395294593409 1.3240816971764 -25.9721618612866 1.77002947029238e-100 1.16507391609335e-98 218.423233603523

TPTEP1 -3.4395294593409 1.3240816971764 -25.9721618612866 1.77002947029238e-100 1.16507391609335e-98 218.423233603523

DPP6 -4.77078122548572 -1.1227278649664 -25.8860417837413 5.10473999072258e-100 3.34996645697479e-98 217.473694922753

MAN1C1 -2.81260020295504 3.5284303959333 -25.814867305215 1.22518161302467e-99 8.01613586509823e-98 216.374869401975

EYA4 -3.76718884158347 0.653660006723751 -25.7390205164672 3.11491916993194e-99 2.0319501080753e-97 215.596679303241

ITLN1 -4.38963354040916 -2.76140175028043 -25.704364487564 4.77124780902954e-99 3.10315709436674e-97 215.313762090288

HMX2 -4.63879162470745 -5.77125178831996 -25.6853525495543 6.02875535524258e-99 3.90938845038921e-97 215.043718406262

SLC16A3 3.21200970755491 7.50451027350079 25.5215112292188 4.52792353093e-98 2.92747671365128e-96 212.777248823431

NOS1 -4.30817725362662 -0.598387293022456 -25.5001217884578 5.89162466966843e-98 3.7979254839606e-96 212.718965570144

GAS1 -2.57718380073759 1.63051667044659 -25.4458667501051 1.14886454597679e-97 7.38415791859731e-96 211.971941515654

HS6ST1 -1.97659753893633 5.15593464883118 -25.4399968993897 1.23494883401672e-97 7.9141750351224e-96 211.73208596072

B4GALNT2 -5.32275256916153 0.121314230136936 -25.4123082367249 1.73650373326385e-97 1.10958526558523e-95 211.553198524233

TFAP2A -3.78434556671899 1.03219201488096 -25.3867575005972 2.37836219767103e-97 1.51528714593892e-95 211.235859597811

FOXJ1 -4.9368799400102 -2.09644991563904 -25.379069560154 2.61445446065134e-97 1.66086259676203e-95 211.296791053637

FYB2 -4.01858643755571 -0.262648392702688 -25.3410643754084 4.17426126777344e-97 2.64406178216385e-95 210.765138141712

PLEKHB1 -2.98911886220653 2.19457325718623 -25.3290019797961 4.84255497522199e-97 3.05850733738515e-95 210.470439975606

CLDN14 -3.1846676377982 -0.0274733741774409 -25.2668143950379 1.04134150300667e-96 6.55805068161523e-95 209.880070881797

ESRRG -4.09257172600288 2.95072681861257 -25.2176073601689 1.90863097180435e-96 1.19854346628852e-94 209.049736563296

HOMER1 -2.37928785439768 2.44022759156724 -25.2152906978733 1.96385946901609e-96 1.22969114545583e-94 209.077888609604

LGI2 -3.03862094383274 1.86354132470326 -25.2128615326873 2.02348735560719e-96 1.26340769091668e-94 209.064741139083

SLC25A33 -1.82356537962867 2.33864054673056 -25.1599393313714 3.88251550571526e-96 2.41722539448421e-94 208.437614990107

SLC6A17 -3.38626376133599 -0.288003794391365 -25.126428576236 5.86579789228847e-96 3.64162731080057e-94 208.156619097939

CXCR4 3.03084472380988 7.10218770835447 25.1004151526926 8.08071287459622e-96 5.0024877747465e-94 207.610913894683

TMEM164 -1.49574006740042 4.7649822582445 -25.0961661772363 8.51479070556355e-96 5.2563198104147e-94 207.504758357165

KCNJ13 -5.21815599666179 -1.82473627401397 -25.0674263400832 1.21305985487546e-95 7.4673231010122e-94 207.443901485233

NTNG1 -5.52046185730805 -2.19644255928632 -25.0634116761176 1.27454164572504e-95 7.82375241124418e-94 207.390520809206

ACOT11 -2.36179788129223 3.07846303354455 -25.0531717262389 1.44584300087876e-95 8.85042215635952e-94 207.034824280029

SLC4A8 -2.33860574643307 1.16598320230598 -25.0459744950075 1.57984663088959e-95 9.64368391755036e-94 207.119089376946

GADL1 -4.84876200444761 -3.49819037677801 -25.0309506595662 1.90094440474951e-95 1.15714033640644e-93 207.050006999997

NDUFA4L2 6.59725827224734 9.22173379883869 24.9631937105124 4.37902287685501e-95 2.65818852577535e-93 206.02885168587

SSTR5.AS1 -5.29440743294203 -3.8111247556794 -24.9588209674589 4.62132057791129e-95 2.79749912989184e-93 206.163870892483

PDP2 -1.60302786221813 3.58025044581773 -24.9307262793977 6.5319128635586e-95 3.94314618252337e-93 205.518100896933

CROT -1.31188406136267 4.1453390049441 -24.9247048904749 7.03474349334499e-95 4.23499310082832e-93 205.419162024213

FBXO2 -3.27426636600311 1.52186844354937 -24.8957424918338 1.00500302301137e-94 6.03360743457898e-93 205.182422419927

PLPP4 -4.10974582712256 -1.09061962216271 -24.8811134645424 1.20342411328566e-94 7.2050485335977e-93 205.158699086937

HMGA2.AS1 -4.42479762083482 -3.36251839587658 -24.8412552783525 1.96618938614563e-94 1.17396548238909e-92 204.7215638749

USP46 -1.3396968378598 3.9534164852275 -24.8348087208007 2.1286745181035e-94 1.26751837177427e-92 204.321986876291

GPM6B -2.38786088558162 1.96965807164763 -24.8343054625873 2.14191037338053e-94 1.27193389645339e-92 204.433547797176

AKAP3 -2.40356509050812 0.170496162820292 -24.8051289131511 3.06813091994895e-94 1.81701531148088e-92 204.228407588187

MAGI2 -1.70070388047328 3.24529336734335 -24.799349888224 3.29448538604736e-94 1.94579430111603e-92 203.923038964541

CHP2 -4.66093667735492 -4.56076553915352 -24.7646037636219 5.05424712025703e-94 2.97710141021501e-92 203.776229266484

SEMG1 -2.78197399726986 -6.21205465032238 -24.7419300144873 6.68263189119939e-94 3.92568695479517e-92 203.462225298782

LZTS3 -2.5745918414803 3.83411782960907 -24.737115437085 7.0909234630616e-94 4.15436864445804e-92 203.105888433712

PLCL1 -2.88354037395499 4.23016133848879 -24.6873322938033 1.30923270070664e-93 7.64990968142839e-92 202.484629213674

SCNN1A -5.02865576785902 4.25883931504932 -24.6750112193943 1.5238030318666e-93 8.8799113747682e-92 202.363812348172

CARMIL1 -1.51147203836704 4.52841540215824 -24.6276452257598 2.73096793338839e-93 1.58722984703022e-91 201.74379362884

C13orf42 -3.70108432359607 -5.63624930666495 -24.5885274511672 4.42162236621612e-93 2.56301627503769e-91 201.587261502087

CACNA2D3 -2.29222340081034 0.904510751884232 -24.5734639790216 5.32311347084055e-93 3.07740737244123e-91 201.333086677057

PAQR7 -1.60433171332086 4.76927736884048 -24.5538370468788 6.77895601037577e-93 3.90872099458421e-91 200.829157819436

FOXC1 -2.24634133038047 4.13866813170869 -24.5352987469037 8.51797255527305e-93 4.89850669079952e-91 200.613569336488

WSCD2 -4.01393137472977 -1.43204276336328 -24.5299381595457 9.09941595240041e-93 5.21914794771145e-91 200.863902018697

MECOM -2.92059251187752 5.18683009205429 -24.5153969773426 1.08843828764937e-92 6.22660782722559e-91 200.364698363099

MAGI3 -2.06736416413374 3.46557814971569 -24.4821774732809 1.63876477953388e-92 9.35037251361721e-91 199.990745339067

COLGALT1 1.74748774529512 6.82611525209095 24.4720513245559 1.85647007214563e-92 1.05649584600517e-90 199.829832693835

SLC7A13 -6.33903069283092 -4.02374796131262 -24.4296365326429 3.13034085277024e-92 1.77681399105424e-90 199.649336134004

COL4A5 -2.87862781722484 3.07863086902259 -24.4234869180278 3.37667862625877e-92 1.91167248755525e-90 199.274681089729

ZNF503 -1.98083845673699 4.03844346907073 -24.4132180866584 3.83198678283614e-92 2.16383481047334e-90 199.11618860914

RNF43 -2.93792305362721 1.92712674180029 -24.4083068155668 4.0709650037885e-92 2.29285562442758e-90 199.164029727327

DANCR -1.88874733416811 3.73551525042852 -24.4054733201021 4.21556163739634e-92 2.36716439232307e-90 199.036743443872

DDB2 2.00967475303005 5.41234841118526 24.4051675846349 4.23146746940158e-92 2.36716439232307e-90 199.134384765753

LINC02381 -2.40011993716039 3.02268722382147 -24.4050922323622 4.23539686724167e-92 2.36716439232307e-90 199.061352160401

CDKN2A 5.31805661943632 1.868975755944 24.3958030278617 4.74884072998831e-92 2.64735756307231e-90 199.235421858896

SHISA2 -3.60788584185766 0.0184039904011502 -24.3888835296295 5.17135160158127e-92 2.87556098089963e-90 199.054869924306

AGBL4 -2.69724217847108 -0.71606619111486 -24.3599132926553 7.38894223904776e-92 4.09823743020078e-90 198.787322250776

LDHB -1.93865116585481 8.82447701066465 -24.3336360237429 1.02129984902724e-91 5.6502444558968e-90 198.179834404754

NRAD1 -4.51757252242901 -1.72396436318467 -24.3095829046485 1.3734888398935e-91 7.57950798439206e-90 198.154054030104

AK3 -1.49814201641041 6.11531696194302 -24.3042832223676 1.46614047141997e-91 8.07042008109337e-90 197.755087624866

HADH -1.71157278128766 5.61470524824204 -24.2243817377653 3.92281949241497e-91 2.15390387858654e-89 196.769626455422

GUCA1C -4.31022977676209 -5.34705178199825 -24.2184193807107 4.22175063613538e-91 2.31222848750542e-89 197.059144227459

PDE1A -3.33763956303153 4.12811394592074 -24.2094038886112 4.71757366904896e-91 2.57732843474317e-89 196.607528903284

PRMT6 -1.32048722381964 3.95626843403014 -24.2065279888995 4.88767756356466e-91 2.66360144131119e-89 196.590139062883

CLSTN2 -3.51949561338778 2.55480539282626 -24.1892885376073 6.04393658219534e-91 3.28552602315211e-89 196.41082885471

LINC00472 -2.33688554978078 1.73319504226281 -24.1689125364008 7.76808855039461e-91 4.21230866232688e-89 196.271974321068

NPHS2 -8.13948932797907 -3.4713563408385 -24.1603065194941 8.63671622457484e-91 4.67173662513946e-89 196.220256837348

SLC20A2 -1.35072430949439 4.98545641200437 -24.1302679184711 1.25033493363174e-90 6.74656032213689e-89 195.612860841239

VIM 2.51189814473875 11.1666269640906 24.0396681518918 3.81617432381434e-90 2.05406052951514e-88 194.537400152484

ANO5 -4.1252348018075 0.558120621211028 -23.9155802912109 1.75910929418363e-89 9.42201357985168e-88 193.15638527468

LOC102723370 -4.1252348018075 0.558120621211028 -23.9155802912109 1.75910929418363e-89 9.42201357985168e-88 193.15638527468

DNASE1 -2.70890643850218 3.64268674104784 -23.913118347424 1.8132569482812e-89 9.68828950875039e-88 192.971059301353

LINC00885 -4.85377107042401 -4.07217288119785 -23.872314207221 2.99692428907449e-89 1.5973606460767e-87 192.826796992048

SLC25A5.AS1 -2.00115632235729 0.500473332155409 -23.8684858694783 3.1415851874963e-89 1.67039078107923e-87 192.714258899058

RPS6KA6 -4.20965656222324 1.49758351134149 -23.7967194752032 7.6019023493032e-89 4.03214495241075e-87 191.625564422988

EPM2A -1.4102328536646 2.92478263109546 -23.7807848656601 9.24972222360987e-89 4.89429006664761e-87 191.434815241237

OPHN1 -1.66745154499642 3.08340308746654 -23.7568297602358 1.24227992947011e-88 6.55737760838414e-87 191.109814270637

DTX1 -2.54844825540523 2.03750379120693 -23.7247996571437 1.84281567344937e-88 9.70386768961184e-87 190.769217889637

PLAG1 -2.46481638769649 0.254545099068218 -23.6817917757347 3.12917430622888e-88 1.64379437774086e-86 190.411828653154

SCARB1 3.9599284575846 7.15703445864506 23.657538370398 4.21791501739808e-88 2.2104099970072e-86 189.932057409164

VWA2 -3.62643417273688 -1.90729156326036 -23.6234109782045 6.42019257030937e-88 3.35647053203279e-86 189.756940714374

COL9A2 -2.71164012904364 2.19828413454344 -23.6148596041031 7.13285668819495e-88 3.72015076866645e-86 189.391678250131

SLC14A2 -5.29801573203487 -2.07976291260414 -23.5846862880049 1.03410438831376e-87 5.38054361852872e-86 189.237930614187

AQP5 -4.80839322802949 -3.83905630749007 -23.5547272285727 1.49524393823369e-87 7.76141705040874e-86 188.928658248782

CORO2B -2.65763333320086 1.91739742931683 -23.5475919523662 1.63249872179326e-87 8.45379018183605e-86 188.59048339502

PVT1 4.85923336216292 3.20776788166592 23.5164409625124 2.39530837480772e-87 1.2374627402996e-85 188.461719458315

KLK1 -6.16390993273282 -2.23500775236398 -23.4962854583537 3.06967222926098e-87 1.5821119628783e-85 188.092232861302

SLC6A4 -2.51960750749239 -1.59148483076467 -23.4898109851049 3.32427582218321e-87 1.70930351863929e-85 188.128497538084

SALL3 -5.90922555404419 -4.40602421357021 -23.448162607805 5.54997879442531e-87 2.8470348965863e-85 187.614264093988

PRR15L -4.336685376139 0.966089234233007 -23.4365730553257 6.40073536509349e-87 3.27576744574679e-85 187.227231106082

SYNE4 -3.39157843907204 -0.55920412747405 -23.4208978115486 7.76249939738235e-87 3.96340886287375e-85 187.216026416769

SLC47A2 -3.9111957088248 0.975199749863882 -23.3881945631459 1.16082353287473e-86 5.91316472352247e-85 186.644983038539

ASB15 -5.19351813999074 -3.79047998272026 -23.3599947897193 1.64231070386857e-86 8.34637577014879e-85 186.532745006143

EPB41L5 -1.87197061439918 4.9608223855886 -23.3530280983574 1.78929275282694e-86 9.07225395070237e-85 186.052423622236

MARVELD2 -2.76059190138669 3.18072597970156 -23.3429853335102 2.02461917808742e-86 1.02416673376723e-84 185.97905303986

AP1M2 -3.27905470485278 3.33902143425453 -23.3422552673096 2.04288677670455e-86 1.03102089448786e-84 185.960183925269

BDH1 -2.82034256680335 2.39209900304832 -23.3372374386011 2.17298045367182e-86 1.09415073396522e-84 185.958021995382

MT1H -5.90202609101531 -0.977019320091494 -23.3192645052418 2.71073716187232e-86 1.3617871080091e-84 185.852201687234

LAPTM5 2.74452211266926 7.74915704677376 23.2382647309496 7.34231028165146e-86 3.68008042625985e-84 184.651687167391

SYT10 -5.63955036149788 -3.67812025666125 -23.2066446412627 1.08328623320428e-85 5.41061393497507e-84 184.642776593629

CYP17A1 -4.22254527695047 0.249821676605967 -23.2065573339327 1.08445014575531e-85 5.41061393497507e-84 184.45694553481

CNTN3 -4.85627463350974 -1.20452438496328 -23.199422184302 1.18392154643945e-85 5.89344819005499e-84 184.459415410509

LARS2 -1.5458854386637 4.04804535038972 -23.197399283016 1.21374764197079e-85 6.02818800454266e-84 184.166865295192

LINC00551 -4.19186677964052 -3.00764184811046 -23.1744336983206 1.609893340588e-85 7.97755083262349e-84 184.25983629966

WNT7A -4.38632311960911 -3.50475966182281 -23.1668321077592 1.76766051915472e-85 8.73952156676203e-84 184.17209912479

SLC16A7 -3.1266097379827 4.04165174496894 -23.1427420761692 2.37719075392771e-85 1.17265800328628e-83 183.488202825323

ROS1 -5.36222274502254 -4.16431260382135 -23.1408994709638 2.43167257948262e-85 1.19683200178905e-83 183.852445804934

RALBP1 -1.37436648538297 6.2110374819438 -23.1218353573238 3.07412054102995e-85 1.50963497040736e-83 183.210737532571

SLC9A3 -4.45541520442825 2.69895740883892 -23.1099842785437 3.55640777576301e-85 1.74256007003922e-83 183.132276102662

DCAF11 -1.11268566576825 6.12022371814777 -23.0901174504524 4.54052456911733e-85 2.21977815232485e-83 182.817147089797

AREL1 -1.20963904451579 5.00958201148825 -23.0879793260987 4.6614807704033e-85 2.2738245374023e-83 182.794957475791

EFHD1 -2.99766596435015 5.52275006360306 -23.076428172183 5.37288441031197e-85 2.61500318526832e-83 182.668795114263

FBN3 -5.12066124684582 -2.75324203211097 -23.0648584996361 6.19424148333595e-85 3.00806131411868e-83 182.886366955755

CFAP74 3.76327377418104 -0.655466698467619 23.0331411764721 9.14839901917224e-85 4.432815161108e-83 182.527426783288

LINC02554 -4.99207989825206 -4.7521586345448 -23.0299876432525 9.5100508152362e-85 4.59785708994152e-83 182.499584110736

FMN2 -4.61663748396554 -2.6811664502817 -23.0099565640713 1.21655551308683e-84 5.8687389906151e-83 182.224095090923

PHYHD1 -3.13052483497817 2.37922525313927 -23.0046837928291 1.29802518920409e-84 6.2479613347306e-83 181.863100818641

SHMT2 1.99940516301972 7.39250643365899 22.9877467823101 1.59847784729842e-84 7.67726074659613e-83 181.564503174182

RNVU1.32 3.24390988240054 -3.00519092063155 22.9810548310684 1.73553157080582e-84 8.31723057386396e-83 181.885356924552

SLC7A8 -3.67335709277166 4.46704235573774 -22.9802568544182 1.75263944320103e-84 8.38083802019085e-83 181.494074496621

ZNF488 -3.78511772892816 -2.68584625703574 -22.9178184421022 3.7756809002832e-84 1.80152739549976e-82 181.114588876072

PTPN4 -1.25906370781246 4.48237623246297 -22.9105579968132 4.12808285445342e-84 1.96538114636973e-82 180.630014098309

KBTBD12 -4.69635875488975 -3.18621881656974 -22.9017404628613 4.60056834199102e-84 2.18556999951152e-82 180.912819731374

MAB21L4 -5.43351015290775 -2.00277037168574 -22.8956430628743 4.95855682254134e-84 2.35052803130143e-82 180.751023482977

MTOR -1.1070669303515 5.6990524209811 -22.8727554035888 6.56914612733424e-84 3.10726299395314e-82 180.145788944232

SLC29A2 -2.48319192467375 1.82397400355683 -22.8559035401775 8.08059925331227e-84 3.81393813137436e-82 180.118398214532

SHISA3 -3.6572617999112 2.30576053608795 -22.8257156330057 1.17094950633513e-83 5.51481887110808e-82 179.659333887386

AMFR -1.53464588041586 7.05377841097525 -22.8049688171171 1.51092411462684e-83 7.1006934789119e-82 179.33648906505

PLA2G3 -3.89083625085203 -5.52703186511253 -22.7957517218931 1.6920863830413e-83 7.93501367566558e-82 179.624297212081

LOC391092 -4.31655585799733 -4.83899071573275 -22.7631315132933 2.52617481882451e-83 1.18210917164394e-81 179.231826255483

PLS3.AS1 -3.1164555256434 -2.45425151314931 -22.7611848594048 2.58731284310874e-83 1.20813135812939e-81 179.202747663132

RASSF10 -4.13722521591985 -0.777741982438804 -22.7590723126956 2.65533587733116e-83 1.23725063810912e-81 179.067578069286

LAD1 -4.01463134386466 2.62546809820079 -22.7440206746513 3.19461336387393e-83 1.48535927320717e-81 178.639757365665

TDRD5 -4.6608992777357 -2.86353754912476 -22.7296853430355 3.80971123555576e-83 1.76759277347346e-81 178.79769230941

HOXB6 -2.26762687036787 3.13599790073513 -22.7049626913713 5.16142787590427e-83 2.38967549517237e-81 178.157659944128

MYLK3 -3.13882774302224 -0.882416176903786 -22.6950862427413 5.82707580424015e-83 2.6921582991556e-81 178.336673803024

PAK5 -4.78166632723125 -5.05729363923323 -22.6730096263294 7.64188688857317e-83 3.52316780962003e-81 178.129916032631

SYP -1.73948533571558 1.23148177103776 -22.6704760712861 7.88339431324499e-83 3.6268592826809e-81 177.961768221455

DACH1 -2.62379086326036 2.49261481472351 -22.6693172665544 7.99638439918969e-83 3.67111319906496e-81 177.753366148147

ACSF2 -2.76678564221308 3.92134733858763 -22.6397949979788 1.14905429631969e-82 5.26421038521471e-81 177.314355185489

NNMT 4.55478874558961 8.45870894062128 22.6326814198557 1.25393772978976e-82 5.73269899771874e-81 177.268929120654

ADK -1.19594514880054 4.08062917171386 -22.6289026624104 1.31349073363194e-82 5.99242442631706e-81 177.196190937677

KCNJ12 -2.6081032981049 0.833040192944956 -22.612473754985 1.60706933439161e-82 7.31651795092913e-81 177.227516034527

WAKMAR2 2.363507804181 0.95293601811248 22.5929689041597 2.04191731634432e-82 9.27692705074271e-81 177.150870296625

CADPS2 -1.62572230391981 5.23679692725312 -22.5652259594548 2.87049128100626e-82 1.30142833949854e-80 176.375385290612

SCN7A -4.85927548912883 -2.99634417444107 -22.5637043971788 2.92461277122637e-82 1.32322076376004e-80 176.760631337222

CAND2 -2.06258965743009 1.35802364742861 -22.5596321176382 3.07453417072061e-82 1.38817758745367e-80 176.562486743076

EGLN3 4.22657377091245 8.24002558439306 22.5300356047405 4.42140645578497e-82 1.99218546965503e-80 176.006545877472

ACSL6 -2.70551063196339 0.827809150113987 -22.5266266913765 4.61033510473281e-82 2.07303812847173e-80 176.163432186065

FOXA3 -4.70614553881987 -2.78610444776624 -22.5210601938439 4.93634590590256e-82 2.21507119264248e-80 176.240016293417

CSPG4BP -2.79303578840497 -1.57123684459927 -22.4687105157866 9.38498058555124e-82 4.20266353967318e-80 175.610950098256

EMX1 -2.89877841215186 3.53012496753654 -22.4582591290288 1.06692380496229e-81 4.76799302859735e-80 175.101101755719

MT1G -5.52336688307714 2.45208515236258 -22.4549755720108 1.11079127005073e-81 4.95390237233031e-80 175.10998963462

SLC48A1 -1.43929431231448 5.08616159775522 -22.4524380907613 1.14592263641254e-81 5.10017258116561e-80 174.99318621652

TNS4 -3.32955895435047 -1.01482960879388 -22.4469613097142 1.22558069904675e-81 5.44362093826599e-80 175.304529245737

ALDH1A2 -3.36739018773845 2.67276329858834 -22.4466360818011 1.23048148502303e-81 5.4543026150524e-80 174.994177040217

XPNPEP2 -5.27511745140764 0.84224586469201 -22.4385478154594 1.35886876456045e-81 6.0014452120256e-80 174.960836532985

SUCLG1 -1.72248473667209 6.41665664258694 -22.4385154518855 1.35940849309142e-81 6.0014452120256e-80 174.832199743123

FAM171A1 -2.17996713353047 4.91658748531047 -22.4356496822974 1.40806063965221e-81 6.2036994270806e-80 174.791695908004

SLC30A2 -4.65884188770312 -0.312664298357896 -22.4271854550769 1.56215930496343e-81 6.8687861753251e-80 174.920353822375

FRG2B -3.5831885571428 -5.92855414411629 -22.4001007563282 2.17792776240257e-81 9.55707939594043e-80 174.786381936969

LINC02432 -3.49391193950056 -1.2863761114918 -22.3974406985728 2.2501760100996e-81 9.85432792559249e-80 174.705305019267

UXS1 -1.28565126167784 5.73160280180199 -22.3923344274692 2.39564644995578e-81 1.04704123741768e-79 174.253434159185

ZNF44 -1.3317885709137 4.03041115086222 -22.3900101627031 2.46493932794563e-81 1.07517603061069e-79 174.265521588381

S100A14 -2.81662186533831 1.29405142464682 -22.3862865832636 2.58014994877515e-81 1.1231875862666e-79 174.393688101812

CAB39L -1.6905287660082 3.83649041567518 -22.3556002931758 3.75946350280752e-81 1.63331125103087e-79 173.844861756486

UPP2 -3.66140913625185 -0.135556317418617 -22.3440658389765 4.33082159998588e-81 1.87780643699388e-79 173.946905522285

SMIM22 -4.28240579960693 -1.61487907477526 -22.3356400679431 4.80233904725953e-81 2.07812901385668e-79 173.934880887651

SLC7A14 -3.97904108700099 -4.53639452038917 -22.3148244443446 6.19907790788883e-81 2.67724208539713e-79 173.751305362294

LRRC52 -4.6633610036349 -5.455519289544 -22.3053010097283 6.96707364792569e-81 3.00298738517002e-79 173.63423186624

VEGFA 3.33082620145017 9.99731355838165 22.2959484389356 7.81378920568607e-81 3.3613136911783e-79 173.081615757898

LRP1B -4.16391675886134 -2.01101870451224 -22.2945974301756 7.94432365434785e-81 3.41075255046098e-79 173.455020554428

CFAP95 -4.43444469950771 -4.06718107906983 -22.2734868786838 1.02915958735602e-80 4.40984793382177e-79 173.242798184204

DHRS11 -1.5553982679274 2.79850101926505 -22.2661806690352 1.12561567435607e-80 4.81371415493214e-79 172.846056998775

DUSP15 -2.97807096154258 1.30471926348653 -22.250213415995 1.36903812644823e-80 5.84327933149868e-79 172.709493081797

RPRM -4.87094642828214 -4.05121896215526 -22.2493487629561 1.38362895676467e-80 5.89404358521993e-79 172.943402554023

CPEB3 -1.42268805648826 3.28314155624904 -22.246078396033 1.44023552021588e-80 6.12324257262212e-79 172.556899461922

PCDHB1 -4.31110349617587 -3.03892547806282 -22.245171060803 1.45634679457161e-80 6.17971776733462e-79 172.881533619523

NPSR1.AS1 -4.8142858573211 -4.54458870408245 -22.2378573342298 1.59296965014094e-80 6.74634995436625e-79 172.808300410019

KCNQ1 -2.10342822943206 4.44607763727919 -22.2367720288409 1.61430732627599e-80 6.82349284354143e-79 172.360800494952

PPM1K -1.56326878943943 4.41482304899319 -22.2288999944272 1.77787042361343e-80 7.50034794734059e-79 172.268436504477

POLR3B -1.27752611587212 3.25158789611222 -22.2162610850428 2.07582983445568e-80 8.7404835014181e-79 172.204285352997

MAL -5.35273518851474 4.11447997636408 -22.2109751088442 2.21479766408978e-80 9.3076871833373e-79 172.092554412632

IYD -4.34781046110493 0.288679751803823 -22.2105206867798 2.22716990555331e-80 9.34171668830257e-79 172.22980825433

NOL3 3.37694422100412 6.18407989982448 22.2089518043317 2.27041825000304e-80 9.50487548224451e-79 172.204974900642

GPAT3 -3.13407767639187 3.6172336838317 -22.2063495418933 2.34401203759635e-80 9.79420555594515e-79 172.01168641788

SLC15A4 1.78616920220714 6.22894419948301 22.2008108886315 2.50868471739016e-80 1.0462268536093e-78 171.957045440271

PCCB -1.85835888924204 4.82220786152764 -22.192744672459 2.76940887894933e-80 1.15275985203199e-78 171.814924002527

PPM1H -2.41322586113306 3.80705748645128 -22.1696967230884 3.67346379909778e-80 1.52616358178106e-78 171.557595275994

PFKFB2 -2.37429365668793 4.06342234956072 -22.1500381674365 4.67425104003614e-80 1.93826201096603e-78 171.308243563559

CHN2 -1.85211403039784 2.69526205981317 -22.1432582831179 5.07922036518824e-80 2.10220080758444e-78 171.331205733703

ARHGAP22 3.05889415991216 2.45944165977719 22.1420978607404 5.15196775277925e-80 2.12827885258005e-78 171.629497419934

FRG2C -4.69403937964918 -4.01132041208508 -22.1352543961086 5.60267248727259e-80 2.3100981483843e-78 171.550658141634

APCDD1L.DT -3.72640843985621 -1.2616973132413 -22.1253485535814 6.32577622014661e-80 2.60333686890516e-78 171.364740235168

AGAP2 2.91179917754854 2.3481902201692 22.1191213469593 6.82737254333595e-80 2.80448443965264e-78 171.348489950703

DDX25 -3.38271332745439 -1.68949734698866 -22.1062398210811 7.99471777394532e-80 3.27783428731758e-78 171.166212593554

SLC16A1.AS1 3.33375850079003 1.78463419387492 22.0981418550339 8.82865783600823e-80 3.61297115524883e-78 171.104364989911

SCD 3.37490233530171 8.09140443646219 22.0969021571882 8.9637827584369e-80 3.66141204897423e-78 170.659658118487

PACRG -3.22786992392918 0.303329985182209 -22.0936439455969 9.32886103143779e-80 3.80342537537332e-78 170.880384424177

CLDN11 -2.76132615631683 0.773761239203794 -22.0607336233594 1.39615375103779e-79 5.68159179169995e-78 170.463200803521

SLC1A4 2.01006215733038 4.88866820878262 22.0539588547876 1.51696694741081e-79 6.16176184047741e-78 170.322856651058

CAV2 1.99090523524268 6.97203291267025 22.0243019042454 2.18143481982616e-79 8.84432191422283e-78 169.764836012523

SERPINA4 -4.98935386225755 -2.013414657484 -22.0235731557321 2.20099311285868e-79 8.9070930546853e-78 170.098891765134

MAP3K9 -2.11904697887716 2.02181967304672 -22.005537668602 2.74505324054356e-79 1.10882899197039e-77 169.701692521699

SFTA1P 4.90132909232937 -0.902482259828913 22.0016066153906 2.88044258493702e-79 1.16137106657987e-77 169.924273742575

LAMA4 2.82358248138619 6.91568189842172 21.997335966617 3.03510317553024e-79 1.22002463244859e-77 169.485025033164

PLXNA4 -3.0979521790928 0.614026479615608 -21.9972827622925 3.03708140782517e-79 1.22002463244859e-77 169.685158279088

CTH -1.9188607530029 1.82776111750707 -21.9881742058488 3.39547381160247e-79 1.36149154504493e-77 169.52508026347

NUDT10 -3.19790265480643 -1.46712536683661 -21.9635049269253 4.59296717168934e-79 1.83828043228804e-77 169.420047514043

ANGPTL4 5.61259914300782 8.43146003781334 21.9626113436832 4.64349711872987e-79 1.85510681052292e-77 169.165664023827

RHBDF2 1.96670135335478 5.43764545954062 21.9455938841902 5.71920291774401e-79 2.28068871097555e-77 168.927629027096

DEGS2 -2.92005959011648 1.46509155430742 -21.9314569287937 6.79990613324217e-79 2.70670944862916e-77 168.80459793744

APELA -5.14404398612752 -4.10284128305746 -21.925204598225 7.34083395215908e-79 2.91671353375514e-77 168.98174346709

GRIK2 -3.29517186052354 -1.91180523723074 -21.9205236733776 7.77378143565432e-79 3.08312968626777e-77 168.908191229041

PML 1.25818813130391 6.70012960956779 21.8939304920905 1.07648571263689e-78 4.26167432577065e-77 168.157668603097

NAP1L2 -3.18237655393028 1.37101398924772 -21.8914747385196 1.10933496278233e-78 4.38377883213062e-77 168.301975893586

C2orf15 -2.01556599031389 0.70236192453639 -21.8647604032404 1.53837291173171e-78 6.06824246210705e-77 168.13375045627

NUDT4 -1.75005499386303 5.39898408563187 -21.8511907626761 1.81628075659706e-78 7.15156457187666e-77 167.632176051443

WNT8B -3.47654520630429 -3.64127500077424 -21.8503649186615 1.83472973946247e-78 7.21121384828658e-77 168.081606146698

ACKR2 -2.56576483610448 -0.308352970638215 -21.8219189500329 2.5986185344466e-78 1.01952622680901e-76 167.657700932749

DDN -5.03022768145058 -2.06807881729767 -21.8108083976766 2.97701630071262e-78 1.16589134801923e-76 167.489749338102

VAT1L -3.76578515026605 0.453012919590894 -21.8086235328991 3.05767010136089e-78 1.19533568381108e-76 167.3488016468

PROX1.AS1 -4.57532976501773 -4.42080187410205 -21.8082853331249 3.07034840907661e-78 1.19814863899198e-76 167.568040801504

TCAIM -1.07903404242602 4.79036208712475 -21.8077377204713 3.09098862914965e-78 1.20405302161867e-76 167.109941253276

SLC5A7 -4.07008703721757 -4.46725755969942 -21.8042772527166 3.22466389668681e-78 1.25388932623304e-76 167.521766778804

PDHB -1.18471411083834 5.5446021733121 -21.7918299820132 3.75508439104117e-78 1.45754634453681e-76 166.903217706367

NDNF -5.2511917509612 0.889213619415889 -21.7732350384718 4.71423008215694e-78 1.82659698555631e-76 166.818250490859

WNK3 -2.36836028645812 0.901695685053725 -21.7725058064366 4.75647077135203e-78 1.8397018719709e-76 166.958958165164

TARID -3.12601520809719 -1.98964326739295 -21.7700913771813 4.89904490265209e-78 1.89149873246742e-76 167.08008448059

CLNK -3.63689237199638 -0.255019784393685 -21.7576602836413 5.70359410772715e-78 2.19824765495875e-76 166.799311970109

MARCHF10 -4.25204522921689 -2.67170352012616 -21.7296800363671 8.03101876121155e-78 3.08982135543585e-76 166.579615355767

FSTL4 -3.47017072271987 -0.567571132678913 -21.7194063892682 9.10615896742861e-78 3.49730917249943e-76 166.368332132528

ATP2C2 -2.86882533448713 -0.776033903345242 -21.7190013854292 9.15137156568932e-78 3.50850741798261e-76 166.414661161671

C1orf127 2.8422586573509 -1.57124152485092 21.7146591665368 9.6504432793732e-78 3.69336492091318e-76 166.428420235341

SVOPL -4.70952121518289 -2.79820039394502 -21.6878452636912 1.33949421701686e-77 5.11747677001215e-76 166.060208420844

C3 4.45447022752409 9.63195223259866 21.68363195997 1.41030616132376e-77 5.37860742467854e-76 165.59377453901

THRB -2.29735794276997 3.56934882969813 -21.6813542967355 1.45013198324864e-77 5.52085962193947e-76 165.59760390326

FGFBP1 -4.68240028779549 -4.801714043776 -21.6730598652477 1.60490266625935e-77 6.09946747230704e-76 165.9225033858

RRS1.DT -3.37644408455263 -3.63994932530613 -21.6479839182737 2.18063328812133e-77 8.27315611897839e-76 165.61690569704

LOC100130691 -2.38992581682524 2.02812943385369 -21.6323306810292 2.64046047463766e-77 1.00003436312403e-75 165.113718150793

CAPZA3 -2.86872515624749 -6.01265455259918 -21.6217087729388 3.00651297558702e-77 1.13670117743085e-75 165.296874129518

DUSP26 -3.64469859111503 -1.9986401471614 -21.5853246441101 4.68998968793854e-77 1.77012685061349e-75 164.813078537656

RASSF8 -1.58639482439023 4.91364633101084 -21.5702394165111 5.63929479917448e-77 2.12475015942e-75 164.202827059942

MYOZ2 -3.27112798634743 -3.22956290692428 -21.5643200263474 6.06227104290797e-77 2.28018604304076e-75 164.594823576336

HOXB8 -2.93623470061468 2.29383468997536 -21.5631702984254 6.14803200911488e-77 2.30846981950494e-75 164.224549662322

FGD5.AS1 -1.16044458837971 6.76153597844818 -21.5488553142386 7.32304799543517e-77 2.74494970573319e-75 163.94675970409

TTPA -4.22207783182681 -4.04471422067265 -21.5396701347742 8.19266799683328e-77 3.06565708449996e-75 164.294540605875

CCBE1 -4.11207510039541 -0.581950162809996 -21.5343596350256 8.74178000764444e-77 3.26554048730007e-75 164.070572780837

HOXB5 -2.1541741232862 1.71315213187851 -21.5255772910595 9.73177562848493e-77 3.62915516739388e-75 163.876898443986

SAMD12 -2.4097737484898 2.81761955845111 -21.5222926195623 1.01301843016585e-76 3.77129331421029e-75 163.703130321031

KCNH3 -2.86305461846752 -1.09143859859657 -21.5189211452295 1.05560816742947e-76 3.92316416374765e-75 163.99304421742

SLC16A1 2.44639414986268 6.08169105012263 21.5141220331396 1.11933740431261e-76 4.15295081433674e-75 163.625037017283

TAPBP 1.59486796385828 8.39292800100331 21.5095248129531 1.18398884762557e-76 4.38537428595959e-75 163.459343088247

TMSB10 2.03459712925153 9.77000203161663 21.4870286260901 1.5583752819468e-76 5.76229696047098e-75 163.203947151599

EPHA1 -1.79074064362872 2.01371061020239 -21.4794696080178 1.70908243723082e-76 6.30888150351437e-75 163.306888229957

PAK6.AS1 -4.59273247606362 -3.4705740117467 -21.4786718567944 1.72581388185679e-76 6.35990063410058e-75 163.536708244339

ARHGEF39 2.69891170953012 2.14423174160188 21.4722806768559 1.86590455842996e-76 6.86458119787372e-75 163.462813288807

TBC1D24 -1.44716257591217 4.06423758786589 -21.4608826236882 2.14454641056172e-76 7.87643238823618e-75 162.899047136803

DEFB132 -3.45996974938107 -5.9820529288387 -21.4509578639784 2.42083333006046e-76 8.87625348352536e-75 163.221061885584

WNK1 -1.61668882497492 7.51297053633846 -21.437680768609 2.84686074909912e-76 1.04208455527744e-74 162.614324859509

IGSF10 -3.10293835758742 -1.30353743278607 -21.4273169952193 3.23084233581636e-76 1.18066216663202e-74 162.879969845915

MOGAT2 -4.64422239882414 -4.25413924090719 -21.4259786718749 3.28406242406447e-76 1.19810711440869e-74 162.908769367791

KLK7 -5.7774761026966 -4.32030583645512 -21.4201954558125 3.52429847749887e-76 1.28360824381305e-74 162.813857499403

LINC00461 -4.46554583769297 -4.35986009277521 -21.4140984419719 3.7966140586512e-76 1.38048930155914e-74 162.768971746727

PRTG -2.46211731032362 0.67255888034482 -21.4066448961585 4.15824724123544e-76 1.50947137811824e-74 162.51475243919

PPP1R1A -5.49873759964458 2.25233196927896 -21.4038092173917 4.30469862483572e-76 1.5600427702908e-74 162.262924029243

OXNAD1 -1.35040901701424 3.06677882090842 -21.3668298381216 6.76009182064476e-76 2.44583255888328e-74 161.84241899149

SCN2B -4.35514784365318 -2.50877038896828 -21.3521025646198 8.09099633761013e-76 2.92252137133544e-74 161.970501791138

NR3C2 -2.4541685719335 3.85504860969846 -21.3432209475453 9.01712178083424e-76 3.24971551769362e-74 161.458515470085

RAB3B -4.06687967430208 -0.965332805643347 -21.3430143240172 9.03988391412364e-76 3.24971551769362e-74 161.779483373181

PAFAH2 -1.0679517115517 4.68071296045267 -21.3430001698588 9.04144526956354e-76 3.24971551769362e-74 161.441862798084

CCDC186 -1.33522398263507 4.74258238816179 -21.3420848093593 9.14299380112978e-76 3.28081844886846e-74 161.424815316544

NR1I3 -2.22831542473484 -0.783845501870925 -21.3405964287439 9.31055159417123e-76 3.33546695061351e-74 161.828812789167

INSRR -4.44988769231908 -2.64003136106281 -21.3285029442785 1.07908699004897e-75 3.85945793674961e-74 161.681601527947

TDRD1 -4.19720343968851 -2.88496632623775 -21.3262616474721 1.1090014391377e-75 3.95996870089478e-74 161.675335262455

NDST3 -3.9418423026712 -2.82393995653268 -21.313237610923 1.2999821726695e-75 4.62679322790661e-74 161.521241119146

ZNF395 2.52427934528696 7.89269581221677 21.2796184606863 1.95900199472072e-75 6.96098708790762e-74 160.660207323627

HPD -5.03064550553118 1.55140540650497 -21.2788749014228 1.97684875008404e-75 7.0129993077251e-74 160.761456424689

FAM215A -3.26971969192093 -4.8663786475593 -21.260965768008 2.45940835461845e-75 8.71076997949384e-74 160.914176228459

IGFBP3 3.42289483592049 10.401047978227 21.2602337024161 2.48146327292453e-75 8.77466292932359e-74 160.432658113368

TRNP1 -3.16089922563672 1.33578308167145 -21.25410618569 2.67399149774026e-75 9.4401835541386e-74 160.534465532293

ATP5F1A -1.66008018099613 8.76236820456265 -21.2520551875563 2.74171366201806e-75 9.66365623485172e-74 160.384031236836

HPCAL4 -4.40401867320949 -1.54778951256196 -21.2494107791982 2.83156574390494e-75 9.96428441248869e-74 160.655973691029

KLHL1 -3.13843049014846 -5.80844841710472 -21.2465862555774 2.93079071200243e-75 1.02968761140497e-73 160.73880397571

SFXN2 -1.99512442467872 3.83465857287293 -21.2273351212087 3.70619092118357e-75 1.29793894552283e-73 160.052291315622

NECTIN4 -3.52974100804097 -0.50526192230743 -21.2187800623944 4.11363994340296e-75 1.43832597893096e-73 160.266985614352

SLC25A25 -1.87775617827647 3.94824289639541 -21.2183580888863 4.13485735566871e-75 1.44343510852122e-73 159.93930282472

KIF5A -2.9592893956587 -1.35883473010084 -21.1822418388718 6.42191409389522e-75 2.23824702861072e-73 159.905893291092

DIO1 -4.47920560932128 -0.371434107220714 -21.1455929613386 1.00378405746612e-74 3.49294474646609e-73 159.280942083604

SGCZ -3.50602059813942 -5.79483939398391 -21.1403645893014 1.06981611660436e-74 3.71680311544597e-73 159.450187709884

DCXR.DT -3.69053535086641 -4.99756851002785 -21.1368431267755 1.11672015381365e-74 3.87360087639519e-73 159.40791713401

TAP1 1.89464704814313 7.00561053364365 21.1340910076609 1.15480317742006e-74 3.99935243045335e-73 158.89582153176

ILDR1 -2.51332334626349 2.62638282963522 -21.0950989776861 1.85701501442476e-74 6.42109954275702e-73 158.510792626955

ARRB2 1.53988160459753 5.6834096488079 21.0807476093181 2.21174255851732e-74 7.6319447951227e-73 158.310421310924

RIPPLY1 -3.03421644722056 -2.41445460947553 -21.0806570877033 2.21418249215567e-74 7.6319447951227e-73 158.704382747605

GPD1L -1.91504388500038 4.69475312453632 -21.0568370252304 2.95941039520457e-74 1.01845661994339e-72 157.950608161367

HSD11B2 -4.25614009035917 5.37206645755506 -21.0473337719293 3.32249962609206e-74 1.14161296114764e-72 157.870488127411

HS3ST6 -3.60224147208035 -5.4997200474765 -21.0021769602816 5.7575605301653e-74 1.97519576555263e-72 157.775090002606

RIOX2 -1.41936629326193 3.45336043819237 -21.0008164051721 5.8537133315427e-74 2.00503444254236e-72 157.34244555701

ZNF728 -4.19660645708225 -3.972036679873 -20.9997796053304 5.92806179136576e-74 2.02732291591105e-72 157.734938543535

PDLIM1 1.74128178284515 7.26841772882129 20.9968941542881 6.13998174172604e-74 2.0965159531553e-72 157.216375549427

MTMR10 -1.16297952523409 5.89678922800584 -20.9957373088463 6.22705672840648e-74 2.12293089993552e-72 157.198564211932

CTXND1 -3.33724210115874 0.395149567487183 -20.9817998935165 7.37836059901718e-74 2.51151579704552e-72 157.302162338843

PSMB8 1.60696018626699 6.8429075770388 20.9679181358157 8.73645533831011e-74 2.96917198301852e-72 156.871332367482

KDF1 -2.73738742856278 1.41782915293068 -20.9670583566544 8.82835089282115e-74 2.99574459721771e-72 157.061808484227

WASF3 -1.76434964584862 3.07163130460319 -20.9629718757553 9.27850724128524e-74 3.14361579447762e-72 156.899052924815

PTPN13 -1.92103888766377 5.46667827769064 -20.9593007254467 9.70243877759413e-74 3.28215781125023e-72 156.760804591666

YBX3 1.53000630701363 7.90006752947426 20.9520158215326 1.06018487861537e-73 3.58086864797243e-72 156.666107200427

PARD3B -1.45500024349958 4.23767795314573 -20.9392577694969 1.2382344594304e-73 4.17579284597724e-72 156.538600080941

BRCC3 -1.14970358901893 3.78943714891707 -20.937602794743 1.2634216353886e-73 4.25416841265749e-72 156.558608343692

CEACAM7 -3.45294487109286 -5.6347796388971 -20.8737633191298 2.74671995582334e-73 9.23447249147806e-72 156.219723471829

C10orf82 -3.66192267316612 -3.12187917093779 -20.8721749473629 2.8002947062911e-73 9.4001290655268e-72 156.180218201657

HLA.F 2.39266768486196 6.79521343374164 20.8465831409475 3.8226002788066e-73 1.28121601062516e-71 155.434680903738

SYT1 -3.03491566816572 -0.563129246021875 -20.838201586339 4.23270224910406e-73 1.41649681852482e-71 155.678893821008

RNF149 1.32856167049053 6.35539662571705 20.827062303663 4.84656835665981e-73 1.61739643004367e-71 155.169209811137

STAC3 2.41169400482078 1.66537899332553 20.8270410206182 4.84782254920881e-73 1.61739643004367e-71 155.633462478625

MRPL33 -1.06765496299344 4.67282484746564 -20.8127402769759 5.76831263540853e-73 1.92156914667047e-71 154.991899714615

SPAG4 4.16055254310764 4.80414663048698 20.7823244936186 8.34841207360046e-73 2.77683179671828e-71 155.01908202885

FGF10 -4.36315740238498 -4.51314299387448 -20.7712891618615 9.54654924621544e-73 3.17052797382289e-71 154.972017553046

ATP4B -3.81989352638138 -4.30102139325505 -20.7476129639039 1.27288216286373e-72 4.22098541806692e-71 154.688345067984

SMIM43 4.92327811312185 -0.578651304901298 20.7421506174976 1.36022347263052e-72 4.50378235566587e-71 154.62729695881

P2RX2 -3.25311295687278 -5.39410633840249 -20.736794013169 1.45168761298679e-72 4.79935391930414e-71 154.562521610519

ADM 2.89433710533754 7.10176277593298 20.729647324867 1.58335821289339e-72 5.22675634839263e-71 154.024979770384

ZNF503.AS2 -1.85223667356471 1.36345447408188 -20.7055159653722 2.12266104182783e-72 6.9964572770835e-71 153.966511359757

MYH7 -3.39153101988561 -4.77006767331017 -20.6991707078818 2.29271489168771e-72 7.5455871277186e-71 154.107084161653

SLC24A2 -4.68965562686407 -3.93435851930193 -20.6837666817577 2.76436092248424e-72 9.07050739325046e-71 153.89750708709

PHKA2 2.21079766465003 6.76407028790865 20.6813311060876 2.84734215659765e-72 9.32878083180336e-71 153.421183344872

DACH2 -4.13214316059788 -3.25545281648485 -20.6779144647466 2.96796079367718e-72 9.70940826709991e-71 153.820797078474

DEPTOR -1.63719768045493 4.79014335160216 -20.6646155803304 3.48809549074536e-72 1.13939238803077e-70 153.185613332013

LINC01561 -4.21675880623115 -4.51479604325412 -20.648055336812 4.26487592955216e-72 1.39104975654482e-70 153.482379519208

MRGPRF -2.83826040078473 1.11907224748521 -20.6408291303271 4.65591123076412e-72 1.51632828801622e-70 153.124569272531

FA2H -3.42135750072267 0.111448291242127 -20.6179982253961 6.14270784360462e-72 1.99756837062934e-70 152.92023732648

SIGLEC8 5.74015967588128 1.79740489955649 20.6046209831144 7.22551294240325e-72 2.34619813269448e-70 152.963639878197

TNFRSF10B 1.53714532303507 6.43816550312183 20.5901443287045 8.61317942254972e-72 2.79263812939138e-70 152.299970468929

GPR182 -2.93988802762808 -1.38427336609298 -20.5845053171168 9.2231592770891e-72 2.98598073603301e-70 152.658511701184

NME8 2.77350450348183 -1.25016740667826 20.5826793035958 9.4297907342294e-72 3.04836119696916e-70 152.700038073579

KIAA1549L -2.8822266200345 0.00326220124103779 -20.5819604455339 9.5123999845892e-72 3.07052403047604e-70 152.535620997113

CGN -3.04048747880334 3.63379974817176 -20.5754792539052 1.02906297118371e-71 3.31683084207634e-70 152.134944476458

HOXD8 -1.83514935376877 4.9546519011957 -20.5731938913732 1.05799581323439e-71 3.40506369758632e-70 152.07528755934

DIRAS3 -2.4122516445095 0.752784575292261 -20.5714964860929 1.08000991490313e-71 3.47080245152618e-70 152.361683194625

GJA8 -3.31473454271924 -5.40025021460356 -20.5692884506452 1.10933293201706e-71 3.559802138527e-70 152.538304502355

L3MBTL4.AS1 2.74988285177095 -1.00034301797332 20.5691584928396 1.1110833720774e-71 3.56019133871077e-70 152.536675498339

EHD2 2.31559763252557 7.95343649064644 20.5257292901807 1.88161792314136e-71 6.02035087473033e-70 151.497473885283

SREBF2 -1.2819206074205 6.52429326197969 -20.5077666158683 2.33955961479725e-71 7.47461933657374e-70 151.28570228103

LINC02303 -5.08424015320095 -4.82481538413803 -20.4966534462818 2.6770455236232e-71 8.54036143470625e-70 151.644427417166

ZPLD2P -2.65864732855828 0.122752583619278 -20.4890307498389 2.93625278879805e-71 9.35363443055449e-70 151.413383775309

MBOAT2 -1.70439647303127 4.200814114252 -20.4659284382165 3.88529261907447e-71 1.23588500152306e-69 150.795082826342

WNT7B -4.78735337330891 -1.98536053050089 -20.4555126361105 4.40812059805161e-71 1.40015493356427e-69 151.053768848799

FDX1 -1.16186350498453 4.62320227459195 -20.4506297401019 4.67687511150091e-71 1.48336359668548e-69 150.602337442407

ADAMTS16 -3.1951069257314 0.933564018724492 -20.4427659925777 5.14453608890468e-71 1.62932677030194e-69 150.718176615303

SAP30 2.48323878149874 3.78621338875904 20.4403837575309 5.29522519847818e-71 1.67462454793551e-69 150.850970734172

PTPRE 1.81354202618347 5.04616254328037 20.4262416566899 6.28509709144211e-71 1.98193689378477e-69 150.471990498777

RNF212B -3.46266911465475 -2.31528360499516 -20.4237531173635 6.4775058282937e-71 2.0396676493617e-69 150.733690849824

CYFIP2 -2.46121051853496 7.61260155411189 -20.4122894614773 7.44270955130243e-71 2.34022347949082e-69 150.175949508539

DNASE1L3 -2.71850593157129 2.40999849974703 -20.3779895694439 1.12767819329731e-70 3.54068269513307e-69 149.819910344072

UGT2A1 -5.11382557909404 -4.90637471870136 -20.3676418863014 1.27824553184836e-70 4.00193404118655e-69 150.088336910666

CCDC160 -2.87546254581282 0.326254541220403 -20.3665514302963 1.29523872338163e-70 4.049335024615e-69 149.896452787927

TMC4 -3.09902602157451 3.45707567458356 -20.3508225790733 1.56701654559158e-70 4.89200179583039e-69 149.421973729304

USP53 -1.4956958232968 5.02297290364203 -20.3483957594854 1.61374932483876e-70 5.03070813062787e-69 149.352844075164

SELENBP1 -1.78198076775337 5.15083696719608 -20.3444829037798 1.69204905695681e-70 5.26728604582295e-69 149.305084236335

NAV2 -1.63430955102676 4.86693636939427 -20.3402102005934 1.78189193514793e-70 5.53907318048191e-69 149.256029762071

SIM1 -5.16483130036789 1.60593316399848 -20.3135150128939 2.4617328799309e-70 7.64151258879687e-69 149.043964000124

CLIC5 -3.48101932969022 3.49959952942147 -20.3115350759404 2.52144679133172e-70 7.81576974907405e-69 148.94677555076

E2F2 2.87965085816745 0.467181852107982 20.3074550350399 2.64910224460419e-70 8.19983446902768e-69 149.377138487334

XAGE2 -2.85370420925445 -5.93314693115212 -20.2952297998563 3.07158424101144e-70 9.49410614127624e-69 149.233013485572

THSD4 -2.38184996622212 4.06237713299568 -20.2941066028449 3.11362509383263e-70 9.61045892309668e-69 148.716324391921

ESM1 3.88682864161173 7.17157162378649 20.2925251965166 3.1737930228102e-70 9.78235527890993e-69 148.808413009958

KCNA4 -3.57413425869614 -5.02064202805054 -20.281869306008 3.61065171624267e-70 1.11131791485987e-68 149.070594007936

AP5M1 -1.16880209480697 5.28644244355468 -20.2633969359507 4.51505234913255e-70 1.38772769318697e-68 148.322597423769

ATP6V1H -1.2699927573922 5.57365754845009 -20.2513100940714 5.22604821664034e-70 1.60400044491912e-68 148.175095544353

OMA1 -1.03873643977752 3.68802758151123 -20.2510282158099 5.24390076259188e-70 1.60722248758654e-68 148.255788909077

PPM1E -2.79603214097482 -0.412967109626787 -20.2364916876776 6.25212179175976e-70 1.91087576944512e-68 148.398644537447

LILRB1 3.20613155112095 3.39934288846772 20.2349013041234 6.37356569219057e-70 1.94527278032738e-68 148.432360347948

GABRP -3.55479889170675 -0.296998438715653 -20.229314929969 6.81913602114845e-70 2.07836233570651e-68 148.254035084116

CNP -1.18380170849618 6.43627442714247 -20.2246341778321 7.21634959167722e-70 2.19636333742232e-68 147.85861943702

TTC36 -2.91058689673478 -1.63254278618889 -20.2238072310864 7.2888907844278e-70 2.21535647165648e-68 148.31377574227

MPP2 -2.66581542262996 0.182968087796986 -20.2233055102142 7.33325728401603e-70 2.22574543649448e-68 148.197734873839

BSPRY -3.14313067658174 1.85921029077281 -20.2058515910338 9.05675413620843e-70 2.74503811565274e-68 147.76965739841

UMODL1 -3.89047462233361 -4.08837463287555 -20.2040565150334 9.25550793845256e-70 2.80139355926598e-68 148.122392846794

MCM10 2.84750315671569 0.39168810041078 20.1606321578441 1.56464212184839e-69 4.72920114643883e-68 147.609153676661

TRPV2 2.10442288988822 4.26626676266878 20.1488681684236 1.80372291968895e-69 5.44430344806115e-68 147.249279066805

APEH -1.31750704934407 6.0414704566076 -20.1400100219718 2.00754784334736e-69 6.05116455457515e-68 146.833652782511

CAV1 2.27561049262285 7.55059302370518 20.1394126840558 2.02209319208602e-69 6.08661191827216e-68 146.832951906358

ARHGDIB 1.67823596517976 7.70295093804377 20.1358660463081 2.11064954078871e-69 6.34443251923737e-68 146.780109275421

C21orf62 -3.29709371253728 1.33488445406608 -20.1352814276269 2.12561452665699e-69 6.38063932019715e-68 146.958747191517

ADAMTS19 -4.39665899802966 -5.069331340256 -20.1254997339276 2.39233150552996e-69 7.17141569140553e-68 147.182711564851

CD1D 2.54925074145938 2.70592876478753 20.1189270230143 2.59008451827794e-69 7.7535776682093e-68 147.044531802761

KRT7 -3.91004307128569 3.37961016454842 -20.1053340913619 3.05239429867175e-69 9.1250304526503e-68 146.463059106536

GABARAPL1 -1.91170377748241 6.75864420100422 -20.1044773447867 3.08415331502327e-69 9.2073773761207e-68 146.426264376395

SLC13A3 -5.19174185324967 1.82276942280048 -20.0822762605627 4.03284411778013e-69 1.20231572313573e-67 146.244447129244

CR2 -4.59485141230022 -2.32155534931936 -20.06304402124 5.08734162448627e-69 1.5146277455027e-67 146.333990620758

LINC02611 2.85869182654428 -0.278686692622059 20.0476793632977 6.12449110984387e-69 1.82093203025059e-67 146.252748518159

SYNJ2BP -1.31422963683776 5.58866556923001 -20.0447960073529 6.34147461616092e-69 1.88288376069246e-67 145.682792449119

IKBIP 1.83819654552024 5.4461508527719 20.0371159428641 6.95764463443541e-69 2.06303131881027e-67 145.724999033724

ACSL4 -1.5442712525337 6.38242897825097 -20.0301022362031 7.5724669238199e-69 2.24229159466445e-67 145.515668195773

POMGNT2 -1.14762452453906 3.92576859270091 -20.0138106955599 9.21826941361474e-69 2.72593831523306e-67 145.366646657903

WIPF1 1.52817364340277 6.45662345098831 19.9928998317907 1.1864761576915e-68 3.50379236135572e-67 145.080278059062

BCL9 -1.05473345852574 4.38427814196504 -19.9905470745122 1.22064721421847e-68 3.59983853877412e-67 145.05856649397

DAG1 -1.21395061234275 6.73110546104081 -19.9742851533703 1.48529723579558e-68 4.37442055173056e-67 144.843686959423

ALDH6A1 -2.93748536204679 5.88618295601352 -19.9707413297154 1.55018492166641e-68 4.55937968952571e-67 144.816531629845

RPS24P17 -3.92671439995871 -4.12536887718341 -19.9619581983954 1.72347705740088e-68 5.06225055583084e-67 145.208441051829

ENO2 3.27036933145855 6.90220900033689 19.9610651019149 1.74214757529519e-68 5.11022160576185e-67 144.790371761899

HMGN1P17 -3.10917336374202 -4.91010876209343 -19.9556582961015 1.8595734465863e-68 5.44735369011401e-67 145.147944722118

IGSF11 -3.56192006696021 -1.07360537851894 -19.9548272598067 1.87831024515728e-68 5.49487467033762e-67 145.0068657211

NCCRP1 -3.15034188796171 -1.78571522793388 -19.9505208143283 1.97846880076853e-68 5.78014421165703e-67 145.021004906832

HACD3 -1.61110411823524 6.38608439765738 -19.9176992193714 2.93935723520066e-68 8.5759377384299e-67 144.162498246468

CHRDL1 -4.7590502672752 0.224257149864323 -19.9084988388318 3.28423856129206e-68 9.5693953706554e-67 144.244900182587

ACAD8 -1.07727124940384 4.64526926890377 -19.8964544496498 3.79755635456271e-68 1.10503327584899e-66 143.912798627688

PFKP 2.06875160802741 8.5889522466507 19.8035908641736 1.16282406117514e-67 3.37914816607185e-66 142.779964304933

LDC1P -3.80530803503601 -5.11655030582237 -19.8008980342836 1.20115803041772e-67 3.48591054963061e-66 143.289317238022

PALS1 -1.43558330653762 5.32582795900958 -19.7761316908653 1.61857800251081e-67 4.69108555555287e-66 142.449064656054

ARRDC5 3.31852136938421 -2.00843138910459 19.774930469338 1.64215983866221e-67 4.75312833831593e-66 142.981417747817

ADH1B -4.22274197263948 2.81976231796628 -19.7638550143848 1.87642318181688e-67 5.42400473442385e-66 142.371269625726

DLX1 3.67846807145336 -1.06849806057537 19.7488196843744 2.24875459446265e-67 6.49168218663042e-66 142.667755129559

BHLHE41 2.94487903059221 6.58289978043765 19.7476624006368 2.28030177865544e-67 6.57406791147194e-66 142.226282495123

INSYN1 -3.10837540307315 0.632340532496179 -19.7465187950079 2.3119104386515e-67 6.65641354622546e-66 142.367541270577

LINC00487 3.35376171405043 -2.36088319611154 19.7419525404606 2.44254343410048e-67 7.02327653492076e-66 142.586470234298

MECOM.AS1 -3.32529045690174 -4.71492713531954 -19.7345104945202 2.67143760526174e-67 7.67134375660774e-66 142.493684820656

BCKDHB -1.43624916766518 4.04349072193927 -19.7292733716666 2.8452461424377e-67 8.15973280192797e-66 141.922407601313

CNTN5 -4.13394200446778 -3.30457451995113 -19.7212619347884 3.13323670085203e-67 8.97386915120832e-66 142.296179326073

HILPDA 4.91307926086537 6.75107785028519 19.7177401754942 3.26888193762838e-67 9.35011478834987e-66 142.058150677538

LINC00602 -3.32537352560319 -5.53511544843606 -19.7175060118197 3.27810633865179e-67 9.36424285209902e-66 142.292621532901

DNAJC11 -1.15868056768014 5.05228770457816 -19.7148598636969 3.38417102183002e-67 9.6546069634532e-66 141.716041965716

MLKL 1.94779157338366 4.32119512372962 19.7054645277006 3.78922937161453e-67 1.07960924977695e-65 141.893316711424

USP46.DT -1.49566549562362 0.713030674826704 -19.6956986022756 4.2616800889612e-67 1.2126366534384e-65 141.881085503752

NICN1 -1.06946956710382 3.90864649073023 -19.6852235036694 4.83404220573412e-67 1.37371032928359e-65 141.41794386732

SLC38A3 -4.21571363439868 -2.16108143320189 -19.6751286965962 5.45817193270295e-67 1.54905754864101e-65 141.685533281404

STC2 4.88092287884017 6.17997728072177 19.6739150461649 5.53843562030038e-67 1.56979810130252e-65 141.581907471532

C10orf71 -4.27822452320836 -5.41136315997372 -19.6587878928098 6.64357274610719e-67 1.88059579301399e-65 141.585573797591

SFXN5 -1.36055990696574 3.99143968141513 -19.6497311669661 7.40801722274383e-67 2.0942742609136e-65 140.973643083199

CD300LF 3.32549516168741 1.73887801600408 19.6397049800081 8.35718396504366e-67 2.35955479571187e-65 141.342401672072

COL4A4 -2.47812082472197 4.43445359808339 -19.6383366419458 8.49581689699097e-67 2.39560111806379e-65 140.807667959708

SLC6A2 -4.24031291980618 -4.92532512203556 -19.6351124531041 8.83162810578792e-67 2.48708207468793e-65 141.297364616442

DPEP1 -4.47322734513357 2.12099525568794 -19.6330297186666 9.05557002402817e-67 2.54686450109508e-65 140.827256305884

GNA15.DT 3.52256997626072 -1.99228269508912 19.6320295204282 9.16512199824852e-67 2.57436260961086e-65 141.270279532395

NPY5R -3.04005253407157 -0.622305082257866 -19.6314138430737 9.23321467036972e-67 2.59015969437214e-65 141.123756660453

IL4R 1.51993921207665 6.02922338866736 19.6266766292476 9.77432947978269e-67 2.73844130925245e-65 140.701909377994

SMIM10L2B -1.99563765696825 1.16744309433861 -19.6262074094367 9.82962170989424e-67 2.75040618727681e-65 140.95868614695

GHITM -1.19327708718077 7.7090174052264 -19.609741632198 1.19811290372685e-66 3.34812804157838e-65 140.480826935551

CMTM4 -2.2124640919031 5.7601981671711 -19.6066229266592 1.24387699730403e-66 3.47157650345912e-65 140.423298299185

PKMYT1 3.09073913551103 0.626391200799946 19.576897062431 1.77794588450141e-66 4.95579737423587e-65 140.604293851559

DPEP2 2.48379563521875 2.1053103544028 19.5713788359765 1.89982163664326e-66 5.28876461472169e-65 140.49225816331

UHRF1 3.16376595231879 1.55988506888231 19.563916149475 2.07802392844253e-66 5.7774881562665e-65 140.436728879681

TCEAL6 -3.13758076290031 -5.3193482420544 -19.5533583593905 2.3590212859246e-66 6.55040561134819e-65 140.328301155188

ITGAD 4.43865387739312 -0.1196465600629 19.5441069894726 2.63628301391706e-66 7.31100161207228e-65 140.216585209114

PPP1R36 -2.97740041773027 -1.05186284725077 -19.5436907915153 2.64949453238697e-66 7.33832750522844e-65 140.111941235518

GLRX5 -1.18408928269801 4.83007060581162 -19.5364691315405 2.88955821220082e-66 7.99310324192717e-65 139.579896021118

TUBB8P7 -3.50696427198535 -4.25275577667051 -19.515862190161 3.70084585816402e-66 1.02243376691973e-64 139.87014701667

LPCAT1 2.36301235335798 7.48151458411462 19.515757042449 3.70552117970266e-66 1.02243376691973e-64 139.334987782061

PP7080 -2.47398272359802 4.40020636645462 -19.5139798316032 3.78544237878813e-66 1.04316862930211e-64 139.316222612108

ADTRP -2.6983673951555 1.06217384890373 -19.5013545774864 4.40500239668556e-66 1.21237427424143e-64 139.42083137516

SUCLG2 -1.31950995424353 6.0741968186439 -19.4986473200974 4.55051825814453e-66 1.2508487483677e-64 139.119291330023

P4HA1 1.64967951791638 6.97257259659084 19.4898497886389 5.0573749918388e-66 1.38842733287253e-64 139.019533363848

ABCA13 -4.16977631274747 -2.70766498647231 -19.4838200806648 5.4369739034666e-66 1.48898296460237e-64 139.430175439095

CAPS -2.01709285398494 3.02158121227222 -19.4838154177813 5.43727820323385e-66 1.48898296460237e-64 139.035638310802

TYMS 2.28784168532588 4.4656972197588 19.4696284989256 6.44655282333548e-66 1.76316043614956e-64 139.076871325963

IRF2BPL -1.37271201458104 5.129620479756 -19.4585598111289 7.3622760187305e-66 2.01109772296647e-64 138.639001854636

SPHK2 -1.11639445031497 3.57827566968796 -19.4575936181156 7.4481269062613e-66 2.03200895483806e-64 138.714064818977

OVOL1 -3.09660202848323 0.73790221120256 -19.4559932090369 7.59253685272754e-66 2.0688242873149e-64 138.876819788991

KIF18B 3.55309193711116 0.678299834378542 19.453913173032 7.7844133218792e-66 2.1184655581946e-64 139.135674886237

OCLN -2.77731939562854 2.43198113997385 -19.4371304360154 9.52073452938068e-66 2.58776880187259e-64 138.490269862671

HLA.F.AS1 1.6812839287797 1.83100727579915 19.4335134780968 9.94292074470631e-66 2.69916331719338e-64 138.82813511194

ST6GAL1 -2.50746213447766 6.83771809304292 -19.4303354646051 1.03292798843325e-65 2.80056765896176e-64 138.335747249997

PHF21B -3.86486903846201 -3.33678758493436 -19.4264830015445 1.08177983155506e-65 2.92938471610565e-64 138.7746222663

ADGRE4P 3.9043454180349 -1.1044659201077 19.4207827564609 1.15832867674525e-65 3.13279165506362e-64 138.744771640311

LINC02048 5.41067210631407 -0.368348520993595 19.4091387352395 1.33192597786944e-65 3.59784652588143e-64 138.605055951977

PLOD3 1.36151795502506 6.69468005718313 19.3977810700078 1.52626136193509e-65 4.11770241263796e-64 137.917060459057

TACR3 -4.15298028952801 -5.01304615646361 -19.3872230813196 1.7322320657643e-65 4.66268038664625e-64 138.335826804895

CD40 1.86555497151444 5.46497598563027 19.3841659501907 1.79689945561187e-65 4.82996848751368e-64 137.883245335839

ATP2B3 -4.23994093449616 -4.01489055425522 -19.3644245638571 2.27666074920131e-65 6.11202301625261e-64 138.043685025874

ADRB1 -3.06735836673311 -1.91147463157787 -19.3470799951157 2.80269018129061e-65 7.51499245788266e-64 137.800058110358

RELT 2.21796720792576 2.68532752051745 19.3446906218462 2.88409848288097e-65 7.72379952774483e-64 137.741645602581

RBM11 -4.04319193125599 -2.46567292579102 -19.3434203920163 2.92833385073729e-65 7.83266580662937e-64 137.743838183694

CTSV -2.64033573027426 0.213643332720182 -19.332882591003 3.32244130064055e-65 8.87595473629559e-64 137.490760986375

LRRN1 -3.33792079496346 -1.76178378216584 -19.326378982702 3.59167824595102e-65 9.58350973245025e-64 137.534140082075

CCNP -3.73119818607356 -2.48492198952028 -19.3152109087723 4.10583899579283e-65 1.09420609237879e-63 137.423329161326

TMCC1 1.74966690834082 6.33993731987792 19.3139957453233 4.16604172270422e-65 1.10889780470469e-63 136.947868777975

APOO -1.41203407348929 3.33518913885208 -19.3015848476373 4.83374910997457e-65 1.28505984550212e-63 136.856493983721

CDC45 2.82373540893674 0.74899789682698 19.2994079546006 4.96143302917693e-65 1.31740213835484e-63 137.286864428322

MPC1 -1.71029439933528 5.30922356501693 -19.2986299102798 5.00788134306304e-65 1.32812173531501e-63 136.724618528962

EPCAM -3.53309928253859 5.35559564873272 -19.2940622931473 5.28945750852737e-65 1.40109715071332e-63 136.698009029783

ATRNL1 -3.49717583665278 0.380856767250119 -19.2794060261243 6.30423653253474e-65 1.6678750719792e-63 136.771645078515

ACADSB -1.97287341609106 5.61411975633879 -19.2764414038682 6.53203746518713e-65 1.72605338242726e-63 136.463281567612

TYMP 3.13686627496849 6.09196818314555 19.2382294728493 1.03205459949497e-64 2.72385134815985e-63 136.197499646958

PNP -1.41873400468726 6.07328278732834 -19.2292919750516 1.14855832054432e-64 3.02767731952412e-63 135.897195171645

SGPP1 -1.35217135923574 4.99388823094358 -19.222716885881 1.24257370681077e-64 3.27156183312479e-63 135.819498309258

PDGFRA -3.47139639362594 2.68539417492949 -19.2131620580173 1.39306476077993e-64 3.66337475539395e-63 135.77881710222

LY86.AS1 -3.12834151074661 -3.83724238017449 -19.1977234204685 1.67564402669676e-64 4.4011837638707e-63 136.072856721065

GRAMD1A 1.32286021529412 5.93744196465158 19.1969545954319 1.69112513406339e-64 4.43651351196725e-63 135.551350220228

GALNT17 -4.40739451362971 -0.865939481331927 -19.1866300015027 1.91340744275585e-64 5.0136322357966e-63 135.727561362442

VSNL1 -3.93695241258454 -2.93094759243433 -19.173356671536 2.24257235149851e-64 5.86909384398767e-63 135.737723057655

DCXR -2.07313781975027 4.70534304385658 -19.1710924427024 2.30412096840923e-64 6.02296118691948e-63 135.207333536941

PART1 -4.8846187100305 -3.53726160858742 -19.1668652104393 2.42358168052901e-64 6.32766194320197e-63 135.650542677844

MRPS9.AS2 -3.0519672520776 -4.55427174520878 -19.1657087367639 2.45732799271178e-64 6.40811320104184e-63 135.701266780348

BTC -2.27739042708926 1.37342800369344 -19.1617451614709 2.57658794929391e-64 6.7111056562479e-63 135.352797676408

CNGA1 -2.92209343374316 0.333552874736674 -19.159982110856 2.63147785542009e-64 6.84591494934467e-63 135.404750586069

PIK3R6 3.34645984779034 1.40072728845843 19.1428064688834 3.23128103717453e-64 8.39633585081747e-63 135.416768662962

ABAT -2.64274599959116 4.4492771353086 -19.1373914567843 3.44734862197028e-64 8.94713888787607e-63 134.812078397472

CTXN3 -5.47355519331403 -1.97574251504942 -19.1121398363958 4.6617224795697e-64 1.20845339675014e-62 134.851474568624

KLHL3 -2.1114606270733 2.61876376624663 -19.0994728951593 5.42343898868268e-64 1.4042465902806e-62 134.469922756445

DARS1.AS1 3.08735847043358 2.01635297162074 19.0957685162731 5.66884608336256e-64 1.46605081017423e-62 134.83623489772

HCLS1 2.07813388329735 5.96141816855451 19.076934119497 7.09907225914747e-64 1.82943426980129e-62 134.175138863642

CTNNAL1 -1.5796328111275 4.52790433345144 -19.0752023853647 7.24743138718431e-64 1.86546664433615e-62 134.06848825087

LRRC37A6P -2.46016232288476 0.00350939315059548 -19.0718845320374 7.54038088561177e-64 1.93858757050911e-62 134.422440204131

SLC22A8 -6.33227059671272 -0.526282544854938 -19.0674444055912 7.95101118107731e-64 2.04175613795632e-62 134.164404421016

UCHL1 -3.55948569023935 2.9427910393212 -19.0641805912904 8.2670310982452e-64 2.12041585199475e-62 133.988401880305

ZDHHC2 -2.09637455004826 4.9168957563788 -19.0570496817928 9.00182025491184e-64 2.30617559238673e-62 133.844908187029

DLGAP2 -3.02246248134575 -2.30816339797408 -19.0523803870144 9.51794919992605e-64 2.4355473520607e-62 134.30318955858

ATG12 1.01761731652492 5.25239421748629 19.0495981912884 9.83943174578725e-64 2.51486668936478e-62 133.829667579152

TMEM116 -1.48690254607057 3.66266970305061 -19.0469678675573 1.01533339383221e-63 2.59206549712796e-62 133.782265144478

KCNMB2 -3.43919978521545 -1.32625963343873 -19.0421892685932 1.07494265472976e-63 2.74104105412012e-62 134.114631642695

FAM78A 2.5175958871161 3.52425000169028 19.0418857754338 1.07884444828133e-63 2.74778411751653e-62 134.08812866665

C1QB 3.00345533608933 7.30628430840947 19.0348831393196 1.17290815233028e-63 2.98388380126586e-62 133.626633603075

LONRF2 -3.08483882024275 1.38822554051479 -19.0278112777702 1.27621766552361e-63 3.24292844705667e-62 133.68669927394

PADI2 -2.51303708951997 3.53126816937375 -19.0248338489908 1.32238779625413e-63 3.35634616858786e-62 133.498928761072

LDHD -2.67852460402686 3.18389153031176 -19.0206091291676 1.39077690318468e-63 3.52174364603648e-62 133.464569788608

PSMG3.AS1 -1.24075530108666 2.5410669391557 -18.9973236974599 1.83626307251313e-63 4.6390586038878e-62 133.336181093859

FCGR3A 3.61715188462012 6.37602178482206 18.9962016635119 1.86101105329303e-63 4.69615179533632e-62 133.33012805322

LINC00528 2.14707954331363 -0.825498851064163 18.9952206167613 1.88292222025333e-63 4.74596300798109e-62 133.675839878637

DGKD 2.12524844565666 5.24211336722616 18.9948000347458 1.8923944423345e-63 4.76434282814928e-62 133.290387986629

LINC00871 -4.05112125599056 -5.32321273508921 -18.9946013860815 1.89688488355198e-63 4.77015251556517e-62 133.665596425824

ANXA3 -3.07555493699069 2.48753478081084 -18.9862580813098 2.09541465039607e-63 5.26334440863278e-62 133.092426631494

FAM219A 1.09909951187279 5.02358354204744 18.9804644129767 2.24535920153823e-63 5.6335056981877e-62 133.037593074741

CRABP1 -5.21826912543315 -4.19094366508413 -18.9803322284585 2.24890247585142e-63 5.63592497761251e-62 133.44297163379

HMGN5 -1.19353060677208 2.68653897664535 -18.9795680468185 2.26949651548585e-63 5.68102031533932e-62 133.108592806355

TENT5B -2.69626751491391 0.728052711308242 -18.9769573529648 2.3412840600728e-63 5.85401379459623e-62 133.195370249187

RHEBL1 1.82488231217909 -0.358081993556711 18.9716513527147 2.49425121698602e-63 6.22818866648155e-62 133.391692064965

PDK1 1.93300925774852 5.77442787756659 18.9715713127506 2.49663353857037e-63 6.22818866648155e-62 132.930005369789

FAXC -3.89958880581655 -1.51037994310687 -18.9543129249795 3.06720776776192e-63 7.64283823818715e-62 133.056395541363

LINC01018 -4.11542107293106 -1.88125460276029 -18.9495668042892 3.24580019587226e-63 8.07864142145747e-62 133.014379320751

STRA6 -3.68395351346079 -0.784359213055372 -18.9475236169565 3.32584725406375e-63 8.26845734278215e-62 132.913823983748

RN7SL138P 2.26941464131639 -0.50413149729476 18.9356929723648 3.82966641727311e-63 9.51019320643968e-62 132.968069668114

MYBPH -3.46789491927211 -3.39091979051257 -18.9354770325309 3.8395381410451e-63 9.52388501660143e-62 132.936691597594

NT5C1A -3.32715480703275 -4.94108418305987 -18.9343558319043 3.89120408521901e-63 9.64109783155227e-62 132.952779063738

NAPEPLD -1.19053663935394 4.76162751894507 -18.9309657839944 4.05168318921652e-63 1.00273423254755e-61 132.347457361708

VWF 3.09488866919007 9.3554506653845 18.9292939575362 4.13324189932906e-63 1.02176171070179e-61 132.320316477718

GABRD 5.921302619846 2.79897467892277 18.9241654630907 4.39380479536582e-63 1.08494707562858e-61 132.82381946592

LOC107987457 1.7319944305052 6.11244303550093 18.9166906505791 4.80323927742088e-63 1.18337303189942e-61 132.224323432541

PPP1R18 1.7319944305052 6.11244303550093 18.9166906505791 4.80323927742088e-63 1.18337303189942e-61 132.224323432541

TRADD 1.47524621184932 5.10140247816702 18.916097057789 4.83734205668369e-63 1.19043283744041e-61 132.300581786345

LINGO2 -3.87314265979172 -3.74968909996934 -18.9152963131116 4.8837295206205e-63 1.20049652659302e-61 132.697360812794

FUT11 1.53799186337622 5.74759464688664 18.913943069426 4.96313566316995e-63 1.2186449848006e-61 132.210024592221

DPP10 -4.51000838582885 -4.37873891630019 -18.9030999252898 5.64776924600605e-63 1.38519305648676e-61 132.556425105987

SLC14A1 -2.80513149402871 2.90575011766207 -18.9009590035843 5.79370985932319e-63 1.41939396362993e-61 132.055102357857

NEIL3 3.21894092542182 -0.727902042186106 18.882879148465 7.1864073661784e-63 1.75861769510746e-61 132.343841339534

CLDN10.AS1 -3.27428218103595 -2.94225004070174 -18.8719798474813 8.18276089932556e-63 2.00019993213603e-61 132.175855376728

ADGRA1 -4.06031005309391 -4.92059267942359 -18.8621526328929 9.19886681658488e-63 2.24606521276904e-61 132.088803512955

SRGAP3.AS2 -2.13567821328412 -6.11852831500104 -18.8514844956215 1.04450647763752e-62 2.5475000062291e-61 131.974875566987

ITGB2 2.57103998614477 6.7230180144877 18.8392566115351 1.2082181666671e-62 2.94349962053246e-61 131.313800195738

GAS1RR -2.47259142197308 -2.52199422550896 -18.8321033579883 1.31562534213849e-62 3.19803788673553e-61 131.710926916767

VWA7 -1.81694558286864 2.38860637032961 -18.8256821412525 1.42013931050829e-62 3.44442889595314e-61 131.258929300931

PLA2R1 -2.4156020517563 3.55432296507519 -18.8134693513704 1.64234743948414e-62 3.97895993293202e-61 130.983254167991

TMEM86A -1.54134252115077 3.70518390893156 -18.8133000419647 1.64566036690314e-62 3.98257098537479e-61 130.994838579539

PIP5K1B -2.1293706841111 1.95608518261343 -18.8113650924276 1.68399949564549e-62 4.07084524096692e-61 131.115694378655

AMBP -2.71776735323467 0.486827006469019 -18.8043843997048 1.82988138823838e-62 4.41860751128986e-61 131.165745451893

CBLC -3.18491283148463 -0.282909784763962 -18.8033285948983 1.85301852224802e-62 4.46953794334282e-61 131.204520741771

TREML1 2.75763818122915 -1.35221902219054 18.7928548613272 2.09897821465321e-62 5.05721840405916e-61 131.278407522271

SSTR5 -4.37779196725404 -3.92899376306021 -18.7861558717286 2.27312955275752e-62 5.47078195114649e-61 131.160106698321

NPM1P25 -1.99792227126133 0.385389446401073 -18.777766105578 2.51172943032833e-62 6.03837439394554e-61 130.922952033489

JPH4 -1.82477923198987 0.825388797593345 -18.7706671555409 2.7330537020082e-62 6.56323324725111e-61 130.803677527823

PRDX4 1.39964828899481 6.31559392360835 18.7697214257018 2.76397314929075e-62 6.63019815932501e-61 130.444663712798

LINC00299 3.32219766411358 -1.6800247946125 18.7647460185739 2.93248500241127e-62 7.02670995150147e-61 130.94670448822

MEIOC -1.6964417472833 -1.06223657720754 -18.7617559410106 3.03865299551302e-62 7.27313076790208e-61 130.847715632223

SFTA2 -4.13348185256466 -3.59955446364731 -18.7589675883528 3.14111493663667e-62 7.51015150003513e-61 130.834296072307

TRIM2 -2.28670812703513 5.80531066987595 -18.7326797270054 4.29379815890223e-62 1.02549039526219e-60 129.992966121088

TINCR -2.45164015146002 -1.59964136572038 -18.7285511391757 4.50981058611069e-62 1.07590492072355e-60 130.450624721477

RGS6 -2.60643813509792 -1.11376848737898 -18.7080543366455 5.75398884541365e-62 1.37123138755534e-60 130.176087172435

CDC25C 3.0703812508562 -0.433136496647658 18.7058382230132 5.90755065618306e-62 1.40629307722841e-60 130.246177525864

DYNLL2 -1.0742000060593 6.97552173178826 -18.7033366475325 6.08581679636082e-62 1.4471529320008e-60 129.644509205552

TOX3 -3.92275868261041 1.36107306894922 -18.6988567209196 6.41859627660881e-62 1.52462591774709e-60 129.732422653166

LDHA 1.5165843790238 10.4243218001115 18.6972725463356 6.54057211286691e-62 1.55191229514094e-60 129.608033213644

UQCRFS1 -1.35677729494403 5.68792148226885 -18.6741375344608 8.60976366624417e-62 2.04066339911534e-60 129.285215280628

NDUFS1 -1.30698724366293 6.77092804909734 -18.662186465887 9.92299832115172e-62 2.34937467293747e-60 129.156430313965

TBX21 2.71083913192267 0.735751174110479 18.6587739221015 1.03334469881884e-61 2.44390494624331e-60 129.677732992225

TBC1D4 -1.58810562554589 5.70826329804557 -18.6570026607556 1.05531291533818e-61 2.4931625015011e-60 129.084334932714

EZH2 1.94730027841887 2.61666685841518 18.6493851569812 1.15523190544045e-61 2.72627244380024e-60 129.463924106508

KLRD1 2.61715433395293 2.30307567818592 18.6393406875184 1.30157109302204e-61 3.06830993482316e-60 129.392654206578

SFXN3 1.31203315651179 6.24031573324505 18.6379561163871 1.32314507589575e-61 3.1158070413308e-60 128.880795559738

LAMB4 -2.45577027177641 -0.477512270063031 -18.6206125147677 1.62565431019779e-61 3.82404990750833e-60 129.102834524715

FAM184B -2.18263082190317 -1.11976711428745 -18.6182884199885 1.67112589239907e-61 3.92678646522547e-60 129.133775490718

PIK3R5 3.15425012566908 3.59115478018016 18.6087757566554 1.87087109786177e-61 4.39142278212388e-60 128.980481839914

CSRNP3 -1.81509563260482 2.2342349042513 -18.6074180902822 1.90126061727424e-61 4.45796655249936e-60 128.692001286205

NUAK2 -2.21096809538281 4.34836977585994 -18.6054918220744 1.94522541861548e-61 4.55616410214406e-60 128.487713682342

CDKL1 -1.66409333550007 2.52724048297533 -18.5991794659681 2.09653865397227e-61 4.90531683139786e-60 128.568476346079

LIN52 -1.01045749903712 3.2502107559862 -18.595641607563 2.18642799205654e-61 5.11016159469642e-60 128.4890887944

HSP90AA1 -1.29023989381019 9.200056933641 -18.564797630211 3.15246226992377e-61 7.36012371630815e-60 128.062741353001

PLK2 2.19234332513673 6.3944663005509 18.5578537069368 3.4230773341733e-61 7.98340544116213e-60 127.977057245937

RDH8 -4.12666025677426 -5.1765423305263 -18.5552118253164 3.53202355195945e-61 8.22871116001811e-60 128.461147677107

C1QC 2.88012920562108 7.05991314281525 18.542508791569 4.10622417632314e-61 9.55626378330028e-60 127.789281620315

PCDHA12 -3.57689434605631 -1.99832321662477 -18.5254726274254 5.02525199878816e-61 1.16702265599912e-59 128.022626752044

MISP3 -1.91673161827091 2.42821245603401 -18.5226159409877 5.19833324516376e-61 1.20593605527138e-59 127.658724605148

DOK3 2.63402036877812 3.33950361842827 18.5198231729986 5.37329572034486e-61 1.24520287780166e-59 127.918982612294

PROZ -3.88488847789084 -2.60728211582184 -18.5059172420118 6.3360271059891e-61 1.46675000367775e-59 127.803315227883

PDHA1 -1.19867313432843 6.25847345637639 -18.503219265635 6.54187586649196e-61 1.51280014085131e-59 127.263885895158

HOXB1 -3.42504116117783 -5.36891701856282 -18.500252149006 6.77597946234497e-61 1.56527990687764e-59 127.819766324221

LCP2 2.30162152888836 5.2810212608343 18.4945339817582 7.25099858026882e-61 1.67324257628949e-59 127.370027670959

SLC38A4 -3.7360976984427 0.186212671589334 -18.4844326831399 8.17284281272678e-61 1.88397820660884e-59 127.32445942321

RSPO1 -3.81419039855382 -4.76573828550904 -18.4716637699742 9.50743516199137e-61 2.18931486401472e-59 127.473432073802

PEBP1 -1.52538599071792 9.02086282998419 -18.452780719239 1.18900947156185e-60 2.73509726126748e-59 126.737985935622

MCOLN3 -3.16325220368535 0.0898801139917329 -18.4410740340954 1.36578742359977e-60 3.13843875582817e-59 126.873533923203

MCAM 2.12340493372566 8.30841261099576 18.440259628467 1.37901990120326e-60 3.16551700640702e-59 126.516170214482

SH3BGR -1.23589238812735 2.00801231799476 -18.4387367873958 1.40410756729405e-60 3.21972326002904e-59 126.783469305459

LINC00652 -2.2134320968545 -2.38873861024426 -18.4293350721222 1.56940974538042e-60 3.5950011704191e-59 126.951433160349

CCNE2 1.70305859800508 0.524777430067082 18.4285327629092 1.58438547350675e-60 3.62550531440242e-59 126.946269901326

RAPGEF3 -1.91028229978414 4.65290727308279 -18.4252929422148 1.64632510896057e-60 3.76329943578611e-59 126.349252397981

KIAA1522 -1.4774647419233 5.9376326141511 -18.418537550466 1.78336418896572e-60 4.07229442230594e-59 126.262656703846

LMX1B -4.88367725703277 -2.96838537126467 -18.4163941310457 1.82918247430932e-60 4.17255998027991e-59 126.716324864428

HLA.B 1.62280760731651 11.3340261175763 18.4142423471459 1.87636212925617e-60 4.27571862467519e-59 126.280098014679

SCN3A -3.61942131927572 -1.4269501258775 -18.4058164597603 2.07309958934384e-60 4.7191088881178e-59 126.57067314496

TBX3 -2.01102289600326 3.16671560495488 -18.4034809043027 2.1311911899045e-60 4.84629771831248e-59 126.164103720435

MAGOH.DT -1.77262480378886 -1.16696755366906 -18.4028371274533 2.14748757490453e-60 4.87827920731692e-59 126.606852607768

KCNB2 -2.97277449300553 -5.53806602795725 -18.4015541869131 2.18033563727065e-60 4.94775438019476e-59 126.658470616897

MELK 2.95788112447164 1.21925827724255 18.3923361347606 2.43158109000726e-60 5.50646026527759e-59 126.527191509633

HMOX1 2.80600219684423 7.5761172544105 18.3903727961795 2.48872088486393e-60 5.63002251521029e-59 125.951548116348

MAGEE2 -3.14289193595143 -4.52819535844066 -18.3897358717943 2.50754410950652e-60 5.6667385134484e-59 126.512970150908

ACAA1 -1.34588211933801 5.64191264732347 -18.3853439550313 2.64126823616336e-60 5.96277218645433e-59 125.867627799233

PXK -1.51419696300772 3.98728256714869 -18.3747806724657 2.9927906934166e-60 6.74937616338834e-59 125.783106850761

TRG.AS1 2.78064519859414 0.817954051398505 18.3599163673676 3.56795984101674e-60 8.03820890780814e-59 126.151272271773

TUBAL3 -4.90586324394057 -4.09349971987979 -18.34872288434 4.07285300741766e-60 9.16622623801216e-59 125.976413419271

OTOA 2.84624992349375 -0.964883249965052 18.3269296188261 5.26962290025178e-60 1.18474351069138e-58 125.778483226472

CAPSL -3.66146010061361 -4.15719912310873 -18.3230468058447 5.51707586558254e-60 1.23910235242112e-58 125.713647781458

DEPP1 2.32766651171098 7.96548003148986 18.3197963168488 5.73313169024803e-60 1.28630520356253e-58 125.096327135326

MYZAP -2.83857432912025 -0.755874751702573 -18.3146389834095 6.0934037134052e-60 1.36573488563122e-58 125.4955192337

OTOAP1 2.78467428148232 -1.4342704050598 18.3040872256103 6.90249783495876e-60 1.54549472528026e-58 125.51134579715

LGALS9 2.2552348696392 5.29086034616745 18.2990969861221 7.32167804245905e-60 1.63767277647756e-58 125.061293846485

GDNF -3.56497086159692 -2.66740024027068 -18.2862447746741 8.52206418224063e-60 1.90421951507673e-58 125.232898111113

TYROBP 2.84538464649436 5.80265221899352 18.2828715552191 8.86845288313e-60 1.97959449290133e-58 124.864942515637

IDI1 -1.02507281499066 4.9287425503782 -18.2747205385822 9.76461451883293e-60 2.17740939877608e-58 124.57140121059

ADAP2 2.01779858661492 4.34286780307983 18.2454714212018 1.37921758746495e-59 3.07237940253532e-58 124.523315691939

HPGD -2.86235539319871 2.87876826386628 -18.245120462186 1.38494315779011e-59 3.08199214126144e-58 124.290395811284

ABHD5 -1.03369095481825 3.27667344525638 -18.2433934415598 1.41346557173824e-59 3.14226481578796e-58 124.325785226564

CCNA2 2.52797782809593 2.11972438291265 18.2394253954416 1.48124147641092e-59 3.28959044552926e-58 124.682449708231

KCNG3 -3.74410958924954 -4.90204146293037 -18.2372748307625 1.51932037955418e-59 3.37073180247691e-58 124.717416786728

MBOAT4 2.14764952150263 -1.56604679683423 18.2219288115085 1.82095914191993e-59 4.03584382640733e-58 124.545780992807

LOC102724652 -3.35034522446669 -5.97635461492631 -18.2185712828971 1.89454778031756e-59 4.19468618472945e-58 124.507220074734

PLGLA -2.71537989119911 -6.11270073928991 -18.216134843676 1.94980111860827e-59 4.31265221102698e-58 124.479734593521

UNC13C -3.8834496177686 -2.46953545060104 -18.2139885229111 1.99980685872883e-59 4.41878455852388e-58 124.364284945687

RASAL3 2.83783106113265 3.6204442846693 18.2137428796983 2.0056110083171e-59 4.42713306714682e-58 124.301375196559

AHI1.DT -1.89251536705019 -0.0677437885743737 -18.2068378984037 2.17583094886603e-59 4.79802560298378e-58 124.224951630931

BTG2 -2.0328605677705 6.93202872052672 -18.2054507386101 2.21172921998201e-59 4.87227002462366e-58 123.777084513091

NEK6 2.41564995103523 6.54801155661674 18.2042655225224 2.24286960141332e-59 4.93589419936407e-58 123.801604844832

SORT1 -1.71753018521568 6.84213013106688 -18.1906642185994 2.63316319021474e-59 5.78898543216928e-58 123.594747961044

SLC5A2 -4.03217274425903 -0.673237763373548 -18.1893118028025 2.67549913941224e-59 5.87614901442972e-58 123.900115510949

ARHGAP24 -1.73457950206322 6.01247139771634 -18.1884476906458 2.70290448772744e-59 5.93037869179796e-58 123.553800173709

ZBED5.AS1 -1.08029972190191 3.35652098248481 -18.1772961055996 3.08277923589393e-59 6.75029806031964e-58 123.535513373869

C1QA 2.71762728847556 7.12066584558983 18.164663814146 3.57787587291862e-59 7.81873214508906e-58 123.314307897867

SNRPN -1.21294207641203 5.94877403545175 -18.1618582369484 3.69819060663821e-59 8.07358235033615e-58 123.233689860596

LINC00919 -3.04500457969243 -6.03308408368869 -18.1428918404917 4.62462831190697e-59 1.00860281936231e-57 123.619848976053

SUSD1 -1.64122722082596 3.80250552378687 -18.1386777954132 4.86008907179122e-59 1.05889856915108e-57 123.00856594836

TMEM140 1.40235926895938 6.622892188163 18.1298294284632 5.39417789953007e-59 1.17409332309194e-57 122.868831774013

NDC80 2.33261071545199 1.6561849845297 18.126732677541 5.59462790188137e-59 1.2165114780081e-57 123.373156754764

GLDC -2.9026954191286 3.44887989874667 -18.1254527951049 5.67963047960682e-59 1.2337670464299e-57 122.851197210778

BCAM -2.0835311343176 7.83270793008344 -18.1180989982928 6.1936295400702e-59 1.34408526652586e-57 122.772602926747

PTCRA 2.83846473350408 -2.68087278182547 18.0980437438248 7.84393253282898e-59 1.69884497165423e-57 123.095578018974

CGA -4.05827884348402 -4.99072238470981 -18.0961029578231 8.02527261843417e-59 1.73639883693705e-57 123.057527268384

TMEM74B 3.4754674264067 1.79111624787108 18.0743454958162 1.03684719638218e-58 2.23914564984373e-57 122.785991585104

ERO1A 1.43884783693742 6.74870225026882 18.0743382521919 1.03693561416824e-58 2.23914564984373e-57 122.213663486998

ATP5F1B -1.39763070747174 9.22668462626925 -18.0727527331732 1.05647124040689e-58 2.27683096810768e-57 122.262739594539

TNFRSF14 2.0359043577499 6.68403609118045 18.0660812862939 1.14278055563808e-58 2.46041216574964e-57 122.143500465639

ARRDC2 2.03960005219632 6.04752802149729 18.0588607143488 1.24414509958423e-58 2.67601406114313e-57 122.111168328103

ITGA5 2.00562162958285 7.26329538897294 18.0360769504108 1.62668167187745e-58 3.49536623161632e-57 121.76464302763

LOC105375050 -3.49720001277546 -4.70048280993337 -18.0157439741991 2.0662045583714e-58 4.43543892083401e-57 122.120157095752

SUOX -1.03898643665001 5.24214876486108 -18.0131237845149 2.13086532856739e-58 4.56975466390414e-57 121.487532633793

DGKZ 1.32596085868595 6.10010826148613 18.0101789981008 2.20595114529975e-58 4.72614219394466e-57 121.484664261387

HCAR1 -2.78013276781427 -0.186928473513377 -18.0073059790109 2.28175252105552e-58 4.87420702273961e-57 121.835330608491

ITLN2 -2.88944526717881 -5.46990643943126 -17.9965650744373 2.58890862959166e-58 5.51955319828941e-57 121.905250661324

ITGB6 -2.986390230861 3.82133023654143 -17.9944679138865 2.65353422631204e-58 5.65182099879114e-57 121.298906462945

GCNT4 -2.17121024943558 2.1618468558741 -17.9906671955465 2.77478910256398e-58 5.89858621189988e-57 121.402579874384

CACNB4 -2.094598075987 0.387498774237574 -17.98252007219 3.05367320778789e-58 6.48512348005722e-57 121.531506814361

OR2A4 5.12794303442727 -1.62190355591981 17.9754563062902 3.31802659906568e-58 7.03969274460023e-57 121.658765377531

FOXI2 -4.27069713536833 -1.79068627574799 -17.9714903333618 3.47634202908852e-58 7.3684289390564e-57 121.446685224756

PTH1R -3.19202200153368 4.01609919786333 -17.9641562135102 3.7892469940948e-58 8.02387738003428e-57 120.939741935889

CHD5 -3.36755375078133 -2.3377563289356 -17.9626091979325 3.85875923774546e-58 8.16316220933704e-57 121.428356961042

HK2 3.70890773276337 5.85152415877607 17.9609079996544 3.93667030596422e-58 8.31992806540001e-57 121.181068422991

IPO13 -1.04741263768921 5.2450349973934 -17.9601463328675 3.97206014981265e-58 8.3866116380537e-57 120.865927606438

LILRB3 2.54327347130848 1.33710026385963 17.950599163981 4.44355618644551e-58 9.3460089838685e-57 121.32827509595

LOC102725035 2.54327347130848 1.33710026385963 17.950599163981 4.44355618644551e-58 9.3460089838685e-57 121.32827509595

LOC107987425 2.54327347130848 1.33710026385963 17.950599163981 4.44355618644551e-58 9.3460089838685e-57 121.32827509595

LOC107987462 2.54327347130848 1.33710026385963 17.950599163981 4.44355618644551e-58 9.3460089838685e-57 121.32827509595

MIR4435.2HG 2.03613697179125 4.67357278304859 17.9503934214003 4.45430961942479e-58 9.35961808781634e-57 121.022480235541

NETO2 3.35209915606878 5.49445643774231 17.9447563460042 4.75926733141144e-58 9.99080393788033e-57 120.997584492051

PRRT3 -1.37771167651337 1.04344254984235 -17.940956046784 4.97653722404431e-58 1.04368779229405e-56 121.037627494573

ENOX1 -1.81201082694087 0.720885649280995 -17.9403641969877 5.01125447629236e-58 1.04996111285155e-56 121.022007791077

LOC100288637 2.04486399735219 0.888041539898376 17.9366390756176 5.23538609196398e-58 1.09587061559089e-56 121.167244317993

PRDM1 2.39027907248072 4.78295447958414 17.9360995860723 5.26866533419575e-58 1.10178127797301e-56 120.874639482139

SBK1 -1.68741175164965 1.14862164045922 -17.9323114660414 5.5083667436777e-58 1.14970714851565e-56 120.896255120043

ZNF425 -1.21874113096942 1.31153360810617 -17.9318029536856 5.54136230852855e-58 1.15549036763621e-56 120.911869599902

SH3GL3 -4.04249843197586 -3.83947991382981 -17.9316187697889 5.55336197587172e-58 1.15688864879623e-56 121.105332086353

SNX33 1.30341682118599 6.52675440777377 17.9279118462952 5.80046286345406e-58 1.20721442814344e-56 120.498546726435

SLC30A8 -4.28243713142125 -3.04947583318369 -17.9255132316165 5.96617296383625e-58 1.24052119675274e-56 120.986820505642

AIF1L -3.97428088211213 6.78281724146693 -17.9233048595074 6.12291650532098e-58 1.27190203793516e-56 120.500689466936

AURKB 3.09238444609783 0.771250175944993 17.915824906653 6.68501402151158e-58 1.38734673705691e-56 120.947402722188

LINC01159 -2.40603472709139 0.333924867283354 -17.9082029566369 7.3108100151606e-58 1.51577923397822e-56 120.648188232339

SPACA9 -1.17977015731992 2.87313612227799 -17.9003700213509 8.0149214353337e-58 1.66019031399381e-56 120.338949072837

ITGA6.AS1 2.79334934240304 -0.413478240453953 17.8916171383073 8.88212493217152e-58 1.83807837256386e-56 120.673820668601

RBFOX1 -3.7457193579067 -4.02429445796879 -17.8888917057655 9.17082400736031e-58 1.89602665120951e-56 120.616726672669

RAP1GAP -3.07263062193142 5.22778440470481 -17.8847833049145 9.6238223678424e-58 1.9862870721436e-56 120.001539807291

TAF1D 1.46177426372388 5.66461257585046 17.8847677429059 9.62558005491269e-58 1.9862870721436e-56 120.058120493358

CENPK 2.13227740073033 1.02640971759817 17.8792490402257 1.02695573362478e-57 2.1171758157455e-56 120.494355673445

NPAP1 -3.60113115329858 -4.26763528280464 -17.8737827963463 1.09498105587379e-57 2.25528944524127e-56 120.450369260302

ALB -4.22734607694985 1.11181453268145 -17.8714927666697 1.12480016398426e-57 2.31452523385575e-56 119.986668972906

UTS2R -3.32798969628911 -5.49270689133158 -17.8640948209631 1.22678440828596e-57 2.52200561376039e-56 120.356230561411

GSTO2 -2.53805715609816 2.43498192328623 -17.8522549476892 1.40955849196898e-57 2.89502647791335e-56 119.726287287273

IL11 -3.61472298772946 -3.17210844300852 -17.8506886548437 1.43569181646973e-57 2.94593176200123e-56 120.147808801102

LGALS1 2.04346331755298 7.51365422948294 17.8502268067372 1.44348963415636e-57 2.95915375002055e-56 119.580642339129

IQCH.AS1 -1.17192301663784 2.47485749744526 -17.8459791499149 1.51721957757165e-57 3.10738513858232e-56 119.756680250683

RFC2 1.08639511632334 4.60415177000726 17.8449025708802 1.53649649711402e-57 3.14391928384201e-56 119.684585922875

FAM180A -3.22833824305077 -0.929882292084285 -17.8416274757907 1.59665607800797e-57 3.26395933327484e-56 119.927016519156

OSCAR 2.73330225888982 1.75501983661589 17.8397677407687 1.63185831770319e-57 3.33280372119326e-56 120.022766974936

HTRA4 4.02894718154378 -0.803954282303514 17.8389610381243 1.64736809199675e-57 3.36133846072876e-56 120.062489181631

RGS19 1.79135616457343 3.66500628621176 17.8366929926334 1.69176703152566e-57 3.44871128170991e-56 119.795343304003

AEN 1.38827217096667 4.96427320686306 17.8275279809883 1.88368535273171e-57 3.8363630953631e-56 119.465699876242

TMEM72 -3.88047681389625 3.80121494446502 -17.8161089060563 2.15349322131249e-57 4.38177722209888e-56 119.218867963013

ERBB2 -1.66431732329842 7.05507710478472 -17.8151912511276 2.17678154963989e-57 4.42504253063074e-56 119.192396719187

ESRP2 -1.73159638491364 3.49918306148109 -17.810039424483 2.3122721422559e-57 4.69610437962066e-56 119.174264061142

CDO1 -2.69511519785499 -0.666129179815848 -17.8096480412483 2.32290308995395e-57 4.71331487695114e-56 119.568130431468

SLC36A2 -5.20874532320055 -1.57347814047144 -17.8049511294138 2.45435498568723e-57 4.97541924881476e-56 119.387958833034

RDH10 -1.78696968583692 5.2850552183974 -17.7995940104139 2.61337432776218e-57 5.29287017466051e-56 118.986883607657

MOB3A 1.11531356816749 6.13298468434994 17.7976817803359 2.6725941679676e-57 5.40779632894406e-56 118.983731185172

C3orf52 -2.39904365304538 1.3018987735277 -17.7958667885297 2.73004161594854e-57 5.51892686709745e-56 119.21407783292

BAX 1.25069391483861 5.46440527539802 17.7885449832645 2.9745825337434e-57 6.00772200645976e-56 118.935094942726

DOK7 -3.02198510991214 -0.876088859736094 -17.7795481163563 3.30521586188846e-57 6.66933353922885e-56 119.217591087459

LINC01987 -3.17082447444247 -5.670490570065 -17.763623853303 3.98291491825836e-57 8.02939480707564e-56 119.185474743405

DDIT4 2.20434082338018 7.60517167753937 17.7624491014017 4.03808725801029e-57 8.13311712896763e-56 118.555559585711

MYORG -1.60084727649476 4.26417887981253 -17.7478893710989 4.78866994885669e-57 9.6359856714885e-56 118.403190139535

FAM111B 2.77456743957826 1.52395586261874 17.7382796811929 5.35885329076688e-57 1.07734149920082e-55 118.848304476266

ST6GALNAC2 -2.14834719845377 2.23482634666834 -17.7374759177583 5.40951013199042e-57 1.08652596428664e-55 118.433422387518

SLC22A15 -1.77441056639393 1.87951568882798 -17.7367664788493 5.454619325896e-57 1.09458031339582e-55 118.505224652861

TBC1D30 -1.16332082940119 2.3050115128039 -17.7338443650044 5.64441390778503e-57 1.13162731309015e-55 118.472681437888

CCDC185 -3.22729113798549 -5.56315253509723 -17.714267689235 7.09767725883333e-57 1.42168232023176e-55 118.610197370946

TSPAN6 -1.26199043562779 5.67851903198786 -17.7078441289387 7.65166961606262e-57 1.53124483626205e-55 117.911184779329

FATE1 3.54444630948795 0.0899364799036468 17.7069407615757 7.73296749555453e-57 1.54609824959152e-55 118.51932680949

ARHGAP25 1.78462413098811 4.71992044696366 17.7065813652488 7.76555041204503e-57 1.55119353888867e-55 118.130168108815

TOP2B -1.00828759769626 6.51124566276206 -17.7064733553016 7.77536937051687e-57 1.55173650094891e-55 117.899835736163

GRB14 -2.18603445615269 2.93378603519117 -17.7056935459297 7.84662901457537e-57 1.5645290497766e-55 117.983490969041

CADM4 -2.40211533849691 3.03194832631004 -17.7046082452267 7.94689132317706e-57 1.58307580752405e-55 117.954260763696

PWRN1 -4.01401543158873 -3.74905841081673 -17.6974025273148 8.64579911099332e-57 1.71916877136066e-55 118.367362843909

PWRN3 -4.01401543158873 -3.74905841081673 -17.6974025273148 8.64579911099332e-57 1.71916877136066e-55 118.367362843909

ALAD -1.10892450605969 5.83183422973536 -17.6946121332808 8.93264731356585e-57 1.77459219766686e-55 117.756096422188

RNF223 -3.7144913793413 -4.5963946226412 -17.6926049774894 9.14483758040984e-57 1.815096599861e-55 118.34323705386

ABTB2 -2.04840188693642 3.56270098492374 -17.6910593256151 9.31166288939112e-57 1.8465314802347e-55 117.768482753203

TAF5LP1 3.01992695633549 -0.408215072604259 17.6909474484288 9.32385529287597e-57 1.84727298019237e-55 118.334696170911

RASD1 -2.93536538678754 4.80038151254274 -17.6852469560145 9.96666069853385e-57 1.97283909642265e-55 117.664660250033

IFNGR2 1.11222986659443 6.64327494821092 17.6840010600565 1.01129412901721e-56 1.99998285985639e-55 117.637594245408

APOBEC3H 2.8033148735337 0.203056308229197 17.6821770300923 1.03309758615893e-56 2.04125511304984e-55 118.227509490677

LURAP1 -2.02355256015279 0.770983480507015 -17.6820026494962 1.03520645938556e-56 2.04357423278704e-55 117.985701978805

ANXA4 1.71203114512988 9.14840763636906 17.6783558928659 1.08030683509244e-56 2.13068098080099e-55 117.581729140482

E2F1 2.41593026378208 2.20657047218489 17.6763145685604 1.10640345353142e-56 2.18018346889288e-55 118.083607326823

KIF13B -1.58521713267113 5.89927826822511 -17.6671850491949 1.23103703616006e-56 2.42359030191044e-55 117.441208522303

SHROOM3 -2.45677230681269 4.29329309987767 -17.6607656847082 1.32697482811871e-56 2.61011529422846e-55 117.380472592868

CHP1 -1.05173926609113 6.82201899473223 -17.6528866442403 1.4549901528218e-56 2.85934350805888e-55 117.280286691994

TROAP 3.17166162996551 0.774273724858588 17.6496541466007 1.51101220052878e-56 2.96676995670759e-55 117.844855108009

GTSE1 2.67186807108545 1.1733058867436 17.6433207762806 1.62709515344163e-56 3.18896057292914e-55 117.752189462603

GMDS -1.04585213197393 3.74610570597604 -17.6392733993516 1.70589968388871e-56 3.34041449749284e-55 117.193932008626

MOXD1 -2.96884374795715 3.02540881834034 -17.6387004243879 1.71735955608164e-56 3.35735781209317e-55 117.172323688641

CD276 1.20677221719726 6.00523382422865 17.6386871951389 1.71762505556224e-56 3.35735781209317e-55 117.139053709345

MTNR1A -3.87554777827437 -4.26386864962556 -17.6219357656308 2.08893834539763e-56 4.07585443410486e-55 117.508100919061

CLEC4A 1.96506225358693 1.62533432748637 17.6056389815016 2.52692034292997e-56 4.92602946066446e-55 117.271590436373

HSPB8 2.40593059031587 7.38366106115326 17.6054645915036 2.53207181822089e-56 4.93167249942791e-55 116.735130709909

ITPRIPL1 1.86951788382831 0.968555276615407 17.6036280840547 2.58696298741894e-56 5.03409636367463e-55 117.275637955854

IL17RE -1.72392971684483 1.87989579950918 -17.5973344732139 2.78424292011859e-56 5.41317264531598e-55 116.884638354904

TAC1 -4.28041797340873 -4.4788543439453 -17.596608105381 2.80795752833269e-56 5.45442629925816e-55 117.209424032668

SH3BP2 1.70732266579196 7.15225589338092 17.5828186230906 3.29843438363969e-56 6.40148193478492e-55 116.458781871008

SPTSSB -3.40435054346889 -2.84411486677812 -17.5664188269859 3.99427245669228e-56 7.738194680505e-55 116.829665520614

CTDSPL -1.29701167494908 5.90644024194735 -17.5611171248761 4.24919344094755e-56 8.22476742825746e-55 116.201645938941

STK10 1.2247046289588 5.64523275632173 17.5457298529296 5.08480095743332e-56 9.82477058556944e-55 116.083599625066

ZNF726 -1.69437602858205 -0.10810135191809 -17.5451914796073 5.11683609294902e-56 9.87793455293419e-55 116.50425096654

NCR1 2.6227545210321 -1.5376890936159 17.5403531306288 5.41393105630297e-56 1.04422449579337e-54 116.588937466025

SAMD3 2.67276036368776 0.846613821796933 17.5256937887303 6.42338834924863e-56 1.23783338268193e-54 116.394420942713

LINC01213 -3.53672042704904 -4.7464952565229 -17.5246624575156 6.50110615500152e-56 1.25170636832818e-54 116.396599020177

CD68 3.10015025990699 1.26077100691375 17.520638864393 6.81339264926922e-56 1.31067842926479e-54 116.333956863075

STX4 1.17077499844424 5.68513481592144 17.5195963188777 6.89672328052718e-56 1.32554172250977e-54 115.772601113539

TGFBR3 -1.62901002981283 5.48617169152689 -17.5111549402464 7.61001810426956e-56 1.46006782820547e-54 115.621280517667

PALM -1.73656102698166 4.53450477915353 -17.5072831131425 7.96138197725048e-56 1.52614105569171e-54 115.587572578173

PLCXD3 -2.73978578005088 1.464811983921 -17.5014134070036 8.52518261211366e-56 1.63278541299316e-54 115.72072871735

TTYH3 2.10140547713397 7.62845826253091 17.4993259492859 8.73515503125313e-56 1.67007299123337e-54 115.485224039446

PRAM1 2.58107064365896 1.61615937299188 17.4869814989437 1.00867489784005e-55 1.92679829916946e-54 115.916279514809

BTN3A1 1.78186859505612 5.99786546041216 17.4815100358917 1.0750783358469e-55 2.05005993658485e-54 115.347894969668

RASGRP4 2.02520864773063 1.47042652764722 17.4805671813363 1.08695418763964e-55 2.07089885461979e-54 115.827945661032

SQOR 1.25444558053039 5.63183501426744 17.4787290691177 1.11048425735767e-55 2.11388610418441e-54 115.30756396177

C5 -1.3939383237815 2.31512631264944 -17.465249137967 1.29931229122682e-55 2.47035809855334e-54 115.315196622948

DNAJC3.DT -1.37342252279085 1.96549384647783 -17.465203052138 1.30000998184979e-55 2.47035809855334e-54 115.369905205885

HMGCR -1.07952472111655 5.02610391396983 -17.4646666794004 1.3081576589528e-55 2.48368108784496e-54 115.084598931848

CHAC1 -2.29657498696439 0.659662441727158 -17.4559987209368 1.44712418684417e-55 2.74513931033902e-54 115.347772411611

LOC100505664 -3.44818050585691 -3.71599905347163 -17.4298820288414 1.96142168486057e-55 3.71429359438978e-54 115.27348854851

TRAM1L1 -1.55198792954653 2.19719490473948 -17.4148350229361 2.33684827510849e-55 4.42139786631566e-54 114.735881227638

KIF1C -1.01338168955474 7.07573187942327 -17.4099745098131 2.47283334100458e-55 4.67463901392501e-54 114.45780758607

VWA8 -1.17858555176184 4.9235161127712 -17.4086476336301 2.51130985026864e-55 4.74327175090065e-54 114.435214626581

VASH1 2.1730949425368 5.38462680818747 17.4083471617919 2.52010554749763e-55 4.75577431169824e-54 114.613127391486

ZHX3 -1.27429806924121 6.09435118482774 -17.4065575804725 2.57313266244222e-55 4.85165384575925e-54 114.405981692612

IFI16 1.63619139541005 6.44486812914305 17.4002668641855 2.76852975653798e-55 5.21557592841591e-54 114.360889106691

PAG1 1.76971901224384 5.31140350724134 17.3969709985123 2.8767473578922e-55 5.41477691748649e-54 114.442429400718

CYP39A1 -2.39328574685245 1.37786514125924 -17.3943644138724 2.96531540227195e-55 5.57668136711264e-54 114.524829514923

XK -3.14697150063203 -0.60586757041211 -17.3923748115759 3.03474684335199e-55 5.70234933514799e-54 114.675083272135

GYG2P1 -4.44771956628297 -5.05782408168867 -17.3917996857321 3.05511782593338e-55 5.73569500430602e-54 114.84612044599

RAG2 -2.90444000103464 -5.24442142110135 -17.3908909844081 3.08758268151299e-55 5.79166904198312e-54 114.856471289178

MYO1F 2.74533008281287 4.72465889830057 17.3900749104208 3.11703171504941e-55 5.8418948601179e-54 114.556800351503

MAP4K4 1.24356458284623 7.04889070207647 17.3829187967764 3.38758039394149e-55 6.33265991007728e-54 114.12874786236

MATK 2.49220619828959 1.93621724603224 17.3793561568202 3.53088939287894e-55 6.59491674381055e-54 114.649663732488

JMY -1.33616965496999 4.19769015278997 -17.378234554094 3.57724722379047e-55 6.67579706075945e-54 114.107973017565

SVIP -1.13632146983867 4.80488538774899 -17.3722581737063 3.83470221094888e-55 7.15014909691688e-54 114.016909628322

FAM151A -3.68845471383663 1.70822537103171 -17.3699895994294 3.93720293166739e-55 7.33501241822059e-54 114.117116032068

BHLHB9 -1.1646910049963 2.59195120574652 -17.3623462431284 4.30311200824904e-55 8.00987280377056e-54 114.103904054437

ST8SIA4 3.59558725016613 5.1613338930115 17.3612434300526 4.35863804324741e-55 8.09943173121477e-54 114.256901328714

STRBP -1.09117209435813 4.03934505060636 -17.3559603058943 4.63472459889203e-55 8.60515179775595e-54 113.869746487034

PSMB9 2.13053976851562 5.73010129580477 17.3527295427298 4.81209182646738e-55 8.92687968453241e-54 113.916698108388

PTGFR -2.76465183393822 1.09449446280014 -17.351241962089 4.89602216421378e-55 9.07487466959828e-54 114.026297312984

LINC00943 3.42851264563888 -2.21108610575367 17.344694072946 5.28317886808562e-55 9.78417862748094e-54 114.32511749215

AKIRIN1 -1.09809719302957 5.3972526160508 -17.3439899124064 5.32659256110039e-55 9.85622584570084e-54 113.677655682233

SRL -1.85489338812676 0.978394512175241 -17.3436157381017 5.34980629832972e-55 9.89080516390857e-54 114.043727973981

TBXAS1 1.85813720736158 4.56888532885168 17.342940502522 5.39195373893903e-55 9.96030135731484e-54 113.930460182074

SERPINH1 1.49652814198177 7.55741269946156 17.3351536210412 5.90262960486452e-55 1.08944395908027e-53 113.573197167311

LNX1 -1.68274099297249 3.60945256821844 -17.3338218464373 5.99468326604334e-55 1.10550053512949e-53 113.620659207433

NUSAP1 2.30784752309837 3.85246960168408 17.3334869300129 6.01805770777114e-55 1.10887533800947e-53 113.960922483387

BCAS1 -3.44490705310511 -0.837235397733687 -17.3322784942754 6.10315571357818e-55 1.12360793436246e-53 113.987006997171

RPS2 1.29480071378298 9.18871198812962 17.3287828946944 6.35613961243585e-55 1.16919797096431e-53 113.523495944244

GAB3 1.6710101776447 2.82190771204208 17.3283661839295 6.38698790580219e-55 1.17388432889399e-53 113.954415527046

MISP -3.44566492608628 0.775764842435496 -17.3274345528178 6.45649646238126e-55 1.18566232934805e-53 113.733991835401

ERVFRD.1 -2.59211457098091 -1.99188520579522 -17.326112266736 6.55645016242004e-55 1.20300676237922e-53 114.034825424374

THSD7A -2.19546651438927 4.61993389787709 -17.3225894895003 6.83034733748652e-55 1.25221124468199e-53 113.440730572022

MAMDC2 -2.31262803472653 0.899320976333283 -17.3206568906969 6.98542972718751e-55 1.27956911842606e-53 113.743328377365

SUCLG2.DT -1.84639911697794 -0.116304343140845 -17.3141166888455 7.53680117190054e-55 1.37941135686384e-53 113.81255950211

GOT2 -1.17017793758266 6.60015389730574 -17.3104258933058 7.86691611659052e-55 1.4386252543586e-53 113.296518388783

ZP2 -2.73911310940186 -5.33906687464313 -17.3033978578999 8.53595996261578e-55 1.55966833664751e-53 113.845545364049

AFAP1L2 -1.72025420483973 4.73918504927196 -17.2864968088867 1.03867091085377e-54 1.89466405800397e-53 113.019739778693

WFIKKN2 -3.48963231424761 -4.42369408370229 -17.2857455934939 1.04776870428362e-54 1.90966551248623e-53 113.624894485057

ADAMTS15 -1.99071636334969 2.82773178236584 -17.2805778870583 1.11254686850103e-54 2.02435359844739e-53 113.063858026016

TLE4 -1.03245433014549 3.48993839651391 -17.272475251084 1.22225658286946e-54 2.22212754621018e-53 112.95798610211

MYO9B 1.19436867501037 6.30486225260342 17.2603261214862 1.40731791905056e-54 2.5564520768921e-53 112.7260955646

HJURP 3.12664476621183 1.0416709044307 17.2507542468721 1.57261691714819e-54 2.85198319422733e-53 113.215758176679

LOC107985317 -5.05291760894947 -1.2439249964759 -17.244350630639 1.6938871463662e-54 3.06681986823037e-53 112.848187117224

NPHS1 -5.05291760894947 -1.2439249964759 -17.244350630639 1.6938871463662e-54 3.06681986823037e-53 112.848187117224

DLGAP3 -1.62035183181056 -0.894616296357602 -17.2401724792715 1.7780006395546e-54 3.21644436888963e-53 113.025064466617

LY6L -3.50782032062964 -5.88564579481295 -17.2355904764365 1.87504542632094e-54 3.38919501252204e-53 113.062169860343

PSORS1C1 3.21040340409253 2.01698747970074 17.2306713945519 1.9851223564312e-54 3.58519659959431e-53 112.954051088244

P2RX7 2.82267528836802 3.74947429131993 17.2240199642571 2.144293152808e-54 3.8694664135684e-53 112.745323692688

SHOC1 -2.7803124852396 -1.17714404302254 -17.2171603611127 2.32180029148407e-54 4.1863285288615e-53 112.718263704178

GMFG 1.89381680823898 4.78543095780266 17.2051115040013 2.66983387982778e-54 4.80988291639542e-53 112.308021313138

EVL 1.64426934146042 6.04458838576292 17.2025854835364 2.74915708414561e-54 4.94870920591713e-53 112.09844637446

OAT -1.21559716346607 5.62198119012012 -17.1963456663323 2.95533314758466e-54 5.31109336136247e-53 111.967198055078

GPRC5A -3.18217338481659 2.93739331892526 -17.1831002695373 3.44557585084065e-54 6.18703114777491e-53 111.885569891219

LINC01751 3.81992669079427 -3.05568665157908 17.182219055612 3.4809338524382e-54 6.24538977646403e-53 112.449808153997

DISC1 1.74998254376514 3.34226566000526 17.1766535226559 3.71276775348808e-54 6.65587479220467e-53 112.15234717484

DPP9 1.3807739602079 6.19746072912423 17.15653084586 4.68722660875885e-54 8.3958986132137e-53 111.539416006468

HIGD1A -1.67098115583343 4.93782637329643 -17.1551142550604 4.76474833334053e-54 8.52776784017123e-53 111.496542964283

SCOC -1.09228416766698 5.86742981264552 -17.1528867243074 4.88924484326287e-54 8.74342615055839e-53 111.464515776233

FAM230C -2.53464387166465 -6.07005756985722 -17.1511848460329 4.9865464572302e-54 8.91013898036399e-53 112.091629935179

LOC103344931 -1.36811601382495 2.07241623197569 -17.1490813987361 5.10948218320184e-54 9.12234592724754e-53 111.686860975925

HLA.A 1.35033135654629 10.7629604069197 17.1488916865281 5.12071753663539e-54 9.13494206759945e-53 111.481865590513

GBP2 1.93224655664758 6.76003257643419 17.1468184578584 5.24511987711442e-54 9.34923366024317e-53 111.423405723846

PARVG 3.10605911123332 3.7657350827568 17.142254721837 5.52969612925339e-54 9.84844739303785e-53 111.821534998852

LOC107985688 2.3134380119948 -1.53336343328399 17.1390219021119 5.74055910143416e-54 1.02156708504593e-52 111.948662377297

LOH12CR2 -1.38252041542138 0.356195144938693 -17.1311158117957 6.29067850740615e-54 1.1185532743885e-52 111.689845851799

ELMO1 1.82177824624067 5.19647066853399 17.1299697647038 6.3746752125077e-54 1.131647257668e-52 111.372561591903

C6orf52 -1.91491370675604 -1.70026682261306 -17.1172403097098 7.38641107809995e-54 1.31018864683213e-52 111.635772880892

EME1 2.1658773746757 0.0890593124834031 17.1170200642095 7.40525872165363e-54 1.31246649508757e-52 111.679434127509

DLX5 3.39289152301305 0.539472511618176 17.1061566666777 8.39700094243008e-54 1.4858272193921e-52 111.560008499304

NKIRAS1 -1.06974670720807 2.92625815115938 -17.104107450667 8.59843312809462e-54 1.52023915168488e-52 111.079794164949

GGACT -2.03441790970213 2.65746822516305 -17.1013377298309 8.87837593124798e-54 1.56846523221958e-52 111.001157492477

C5orf58 2.50808461868528 -1.72580681008223 17.0980748267151 9.21986046816387e-54 1.62616312195952e-52 111.478397768975

BAG1 -1.28286224403315 5.82502428740036 -17.0891493733266 1.02224471737206e-53 1.80154143618804e-52 110.729908531639

TSPYL5 -1.77763251442652 4.02346057977464 -17.0816051951558 1.11542743832305e-53 1.96259547581913e-52 110.67542393676

ZNF98 -3.50173043402556 -2.74197966898573 -17.0742880502244 1.21389786902827e-53 2.13413597199315e-52 111.127456154087

MTTP -3.85069009879857 -0.677331547615071 -17.0737280333532 1.22178244773275e-53 2.14627104745207e-52 110.934230433984

PCED1B.AS1 2.93702264507656 2.56012973204948 17.072248545413 1.24285923188855e-53 2.18154239312936e-52 111.088736625133

PRKCQ -1.73315721114648 2.90200196274695 -17.0641696736536 1.36451908659074e-53 2.39162620372823e-52 110.569004818123

GM2A 1.29129718573558 6.64237399304535 17.0641559129698 1.3647361351069e-53 2.39162620372823e-52 110.45006984248

IL19 -2.37130332876779 -5.69253721722941 -17.0504173943287 1.59957218282293e-53 2.80091754096389e-52 110.931762917218

ALAS1 -1.10723050169649 5.07538433747969 -17.0469788697524 1.66440568727823e-53 2.91211028695685e-52 110.248015941864

PLXNB1 -1.52057344217079 6.11401764389455 -17.0432356904007 1.73796893093592e-53 3.03595803738951e-52 110.205936788418

PELI2 -1.36034962873014 3.67827506169899 -17.0430263011124 1.74217852621956e-53 3.04088077743419e-52 110.266273568454

PDE11A.AS1 -3.83588465079049 -5.11847150626541 -17.0410647888493 1.78211069551022e-53 3.10809776767636e-52 110.812733705305

CD300A 3.10120219145332 3.83541458068717 17.0400986439494 1.80211387521239e-53 3.13861623136993e-52 110.638328858618

TREM2 4.5926441662271 3.66836021755618 17.0400809761338 1.80248175095834e-53 3.13861623136993e-52 110.730579958163

DLGAP5 2.9217601750345 0.979228969287992 17.0379439464572 1.84753599993471e-53 3.21450670434501e-52 110.761429866342

PTPRQ -3.90971920072053 -4.10829906937391 -17.0363523222492 1.88182052255211e-53 3.27155321235729e-52 110.727592018602

STOX1 -2.51640646373057 0.348046209650558 -17.0357561167417 1.89482601781835e-53 3.29154475098445e-52 110.50322278935

PALD1 1.99217541035863 4.76808594719888 17.0284696961086 2.0612093272143e-53 3.57772894579937e-52 110.284455755537

N4BP2L1 1.36274983580457 4.33228490690479 17.0280537817765 2.07113493891742e-53 3.5921041127113e-52 110.265626819633

IGFBP2 -2.68134295805308 4.35971127497349 -17.0269920452443 2.09668970950543e-53 3.63354165121507e-52 110.031069547518

DARS1 1.04859491746599 7.43047304982306 17.0201981424316 2.26782894151977e-53 3.92676102965949e-52 109.931892202833

KIF4A 2.49153121621319 1.76477266930938 17.0201350433231 2.26948207589802e-53 3.92676102965949e-52 110.513351921443

NINL -1.2822991529972 4.05879053630218 -17.0150593210153 2.40647813962095e-53 4.16050370135574e-52 109.919179049624

GNG7 -1.4775666113593 3.72928839004711 -17.014105651828 2.43312464180504e-53 4.20324686145182e-52 109.92513091344

BTN3A2 1.9207338172238 6.55831960093009 17.0111731793641 2.5169203092406e-53 4.34457026207226e-52 109.869531428896

LILRB2 2.51786228558922 3.42291129437847 17.0055603208762 2.68542561708891e-53 4.63177632282904e-52 110.234394447092

KLC3 -3.4393637098298 -1.39942752248812 -17.0039130435841 2.7369836126125e-53 4.71697972290386e-52 110.24234394486

ARL11 2.08356302995789 1.20863485325302 17.0032522813325 2.75794136891313e-53 4.74935324939784e-52 110.330967566864

PRRG1 -1.2759049415257 4.20063681203132 -16.9969733705937 2.96526203730246e-53 5.10235207095831e-52 109.702041251567

SIGLEC17P 2.17458048947741 -0.880910370666822 16.9915683001941 3.15614592483871e-53 5.42653476754527e-52 110.249138705946

FCGR1BP 2.74697784023132 -0.0175358080463512 16.9889330697273 3.25361086970922e-53 5.58971370564116e-52 110.214237689435

ALX1 -3.87104627691879 -3.174421039003 -16.9810244755503 3.56452360090762e-53 6.11905217993985e-52 110.061365192147

ANKRD18DP 2.75657147937168 -3.7080112958477 16.957670022434 4.66670826880884e-53 8.00483326517108e-52 109.868298138042

LINC02910 2.47556741127052 -1.56218524926596 16.9553869444519 4.79122084197134e-53 8.19910329362566e-52 109.838250122644

ANKRD9 -1.46829211331901 3.64961350724823 -16.9532681854363 4.90973667925849e-53 8.39534238277275e-52 109.232798549978

APLP2 -1.06978989165331 9.6130897479693 -16.950781572542 5.05256667612409e-53 8.62607340416717e-52 109.217713511505

GPSM3 1.99830702064801 4.677682206618 16.9408361963128 5.66651051666127e-53 9.66668651995306e-52 109.292144374635

CASP4 1.25603152896134 5.64612871953422 16.9406961509602 5.67566735403633e-53 9.67475496784368e-52 109.085179626636

UGT3A2 -3.69383550609948 -2.24031328128289 -16.939374290508 5.76282790935339e-53 9.81567250998439e-52 109.5378835901

SKA1 2.44151525996752 0.0064537784208352 16.9267551891069 6.66518080974059e-53 1.13349569054678e-51 109.498089826609

SKA3 2.29267465906859 0.663408082095891 16.9216716799589 7.0673624002485e-53 1.20095700258655e-51 109.421645248208

SCPEP1 -1.26003315693054 5.92243188851188 -16.9157634354372 7.56532553745161e-53 1.28457699277335e-51 108.733354402563

CENPA 2.76385746673391 -0.448052992393279 16.9138579903424 7.73327470595851e-53 1.31207493904745e-51 109.356441299911

HS6ST3 -3.72091037181344 -1.09541082516322 -16.9100560696022 8.07958506019011e-53 1.36976859829584e-51 109.113087977418

UST -2.21486682798952 1.76503226602902 -16.905637842864 8.50152656369774e-53 1.43906940353592e-51 108.850025401494

NHSL1 -1.29178782907699 4.33044090201754 -16.8945049543191 9.6648361206259e-53 1.63345447597864e-51 108.515834801083

MYO6 -1.32497811530039 6.67589627096862 -16.8926904860539 9.86895145536578e-53 1.66666303055725e-51 108.479523345605

BTK 2.58371588611776 2.92129887122852 16.890478690786 1.01235915921257e-52 1.70834630936466e-51 108.955977987894

ZNF667.AS1 -1.79286325135007 2.89957525434204 -16.8842783790508 1.08729258517952e-52 1.83196645057271e-51 108.494447039503

TPD52L1 -2.11494985326857 3.64168164840163 -16.8830291402755 1.10304746258727e-52 1.85707983050227e-51 108.404084106586

LAT2 2.32668650781754 3.62895483666287 16.8745196805627 1.2166003034475e-52 2.04667947892519e-51 108.696109792246

PNMA8A -2.47090565896425 3.21463033180055 -16.8655938122872 1.34826685513096e-52 2.26643658347514e-51 108.221703208486

ADPGK.AS1 1.76682261582804 -2.5019594960437 16.8455466815986 1.69815727812844e-52 2.85021743463447e-51 108.581920395438

IL10RB 1.03280180374375 5.40910229748385 16.8409410929944 1.79057357180004e-52 3.00302411853771e-51 107.94387399611

TNFRSF1A 1.05623567595536 7.23113437497256 16.8384667449593 1.84227999879783e-52 3.08737306853749e-51 107.841805628392

ALS2CL -2.10214603401137 3.20990235042476 -16.830269633569 2.02445990205916e-52 3.39007833254397e-51 107.827696231487

TRBC1 2.95516704425313 -1.00633431847594 16.8277686808073 2.08354442612447e-52 3.48634734640874e-51 108.374104090429

DPPA2 -2.95775497970915 -5.50736973536259 -16.817114519999 2.35514989796397e-52 3.93780342159193e-51 108.254667997388

ADAL -1.0463294639971 3.09037648203701 -16.8109783738647 2.52733118069444e-52 4.22245934951955e-51 107.68937832571

SIGLEC9 2.45026047502114 1.82709321185437 16.807149891613 2.64106678179032e-52 4.40574292995906e-51 108.064218951357

CGNL1 -2.7568845008437 6.4175845741184 -16.8029997211287 2.77013657954079e-52 4.61752819776544e-51 107.472344653864

PITPNM3 -1.57609476666834 1.46447292631766 -16.79803887761 2.93270430395763e-52 4.88478560627943e-51 107.710623586714

PRSS35 -2.59250102159111 0.552621936820167 -16.7978944037406 2.93757880231952e-52 4.8891781848506e-51 107.740882647449

FAM184A -1.75312542575829 1.47006354849153 -16.7955245207711 3.01870204212414e-52 5.02037258192837e-51 107.667436339684

IL12RB1 2.63249256950158 2.19557276024433 16.7939009926674 3.07556379524802e-52 5.11104909639202e-51 107.899962107432

LINC01924 -2.61273815706173 -6.0559190942664 -16.7872139899031 3.32125823537753e-52 5.51515624754598e-51 107.914876670348

SACM1L -1.01420516321674 5.42796648109655 -16.7859178531499 3.37110056945767e-52 5.59367203829602e-51 107.241872652862

MACROH2A2 -1.45055746738241 3.17600516439636 -16.7854967243187 3.38745503800649e-52 5.61654438130166e-51 107.355052506346

SLC27A4 -1.14497206936823 4.52788980227857 -16.7826776489086 3.49899161937864e-52 5.79707838197737e-51 107.227101282

LYPLAL1.DT -2.41747097538944 -2.67789971711419 -16.773565390637 3.8852084283831e-52 6.43208028677696e-51 107.7131141689

COL4A3 -2.35649956854992 4.0964190047758 -16.7729713318403 3.91181628349959e-52 6.47122795180293e-51 107.117440070368

PLIN2 2.81764929725943 9.11278001379762 16.7601655409682 4.53174369253503e-52 7.49108887390075e-51 106.950122827845

CSPG4 2.72403237524412 6.62327756696503 16.7595000523801 4.56651533813407e-52 7.54286165413785e-51 107.032271176157

GATA2 -1.72163439205617 3.58264871601709 -16.7589713182159 4.5943313299246e-52 7.58307572151377e-51 106.99544149898

DENND1B -1.1907421973086 4.08906585847908 -16.7466474450806 5.29281663599595e-52 8.72276937755804e-51 106.837433477712

MRO -2.27128969570391 2.01800081715145 -16.7457849397129 5.34549504238357e-52 8.80294673407748e-51 106.967874750917

KIF6 -1.84193926191402 0.0794729269973638 -16.7447733246684 5.40794768885611e-52 8.89908741299493e-51 107.245695324833

NRL -1.26298845065387 0.567611849484561 -16.7399705767495 5.71453001284903e-52 9.39651048689163e-51 107.185379331698

FAM181B -2.81708867425299 -1.95678865314248 -16.7371441826293 5.90299841847954e-52 9.6991146194762e-51 107.249956607016

LMO7.AS1 -2.88386599770333 -1.93887749609977 -16.73311566814 6.1823985688009e-52 1.0150560174606e-50 107.200024850994

ITGAX 3.65862122428358 4.37043090176243 16.7268216057076 6.64557912657469e-52 1.09028408898676e-50 107.036010224735

LRBA -1.27013823899265 6.40550771097492 -16.7262618919794 6.68840856384739e-52 1.0964875644843e-50 106.564955942253

SRD5A3.AS1 1.29240282782585 -0.00298315860239406 16.718187896042 7.33778291024567e-52 1.20204325290554e-50 107.089793537914

COL18A1.AS1 -2.84671439931348 -1.61594137408196 -16.7177473020036 7.37497660727116e-52 1.20723118950335e-50 107.003905796267

C10orf55 -2.03608838298304 -1.27250846061447 -16.7159472507279 7.52889680160885e-52 1.23150435483202e-50 107.00494466022

MAN1A1 -1.67588080407308 6.67258742909983 -16.715366338102 7.57925106902697e-52 1.23881356478269e-50 106.452184840452

CRACR2A 2.27673978268926 1.69795546601424 16.7152382554782 7.5903985877463e-52 1.23970837322885e-50 107.012097168408

GPR27 -2.53808655688002 0.671621033059667 -16.7134876028933 7.7444152048059e-52 1.26391863682318e-50 106.7583915221

EHBP1L1 1.51693554933291 5.70536299570733 16.7079684969682 8.25068279748974e-52 1.34553859084734e-50 106.429823850557

NLRC5 2.13339605918554 5.37479781341559 16.703940686439 8.64088200760548e-52 1.40812225587027e-50 106.489759441201

PYGL 2.04257777924789 5.8055288390385 16.7014363966125 8.89271920267377e-52 1.44808191308517e-50 106.393097803045

MS4A4E 3.14702420442201 -0.877754714569389 16.697927367022 9.25796663988788e-52 1.50643592689106e-50 106.89013906487

MYO5C -1.33635307648296 4.02312685917827 -16.6946031553402 9.61777703261538e-52 1.56265636798323e-50 106.24060493866

MIR99AHG -1.61509890685884 1.80180002106235 -16.693809709139 9.7057023599884e-52 1.57577053248757e-50 106.476511589147

SIRPB2 2.44889725000743 2.13916828619566 16.687321921053 1.04554077986642e-51 1.69564471689749e-50 106.675314798085

NCAPG 2.60229097515034 1.44919904771271 16.6872872428474 1.04595665509441e-51 1.69564471689749e-50 106.718314232425

HLA.DPB1 2.01800426369338 8.41302791366246 16.6854276438318 1.06850123027372e-51 1.73090862751458e-50 106.091732962968

DTL 2.51150451843825 2.10135920253251 16.6845791249889 1.07894863659506e-51 1.74653811522309e-50 106.65079320649

LINC02857 -1.726975003406 -6.32181012013417 -16.6763556007602 1.18563509549415e-51 1.91781522885519e-50 106.650805570138

BRCA2 1.87291003992251 1.87880510278723 16.6761551392886 1.18836316768992e-51 1.92080623546803e-50 106.537868241211

MALRD1 -3.11398728152677 -2.10257277915345 -16.6752519451937 1.2007326149042e-51 1.93936510225437e-50 106.536169455614

TMIGD2 2.51356841003201 -1.55919294477313 16.6651080284609 1.34880184338603e-51 2.17691039021528e-50 106.518302611501

YPEL4 2.47520617325927 0.693682028868529 16.6644269831413 1.35937211999477e-51 2.19235121315467e-50 106.482306007047

ARG2 -2.94600750894466 3.4124210972375 -16.6526819412822 1.55523716109572e-51 2.50638625969209e-50 105.765354305892

GCOM1 -3.32652811816665 -3.46741506974543 -16.6520442697715 1.56664306201711e-51 2.5229072095991e-50 106.326098008825

CD160 1.85699973741383 -0.030044673608308 16.6516846222913 1.57311278705324e-51 2.5314605107124e-50 106.34453099128

FMNL1 2.37769500492698 4.69275273737374 16.64833327079 1.63469725025121e-51 2.62862685870049e-50 105.979310732234

MMP25 2.12255959164953 0.965937137022489 16.6409509244223 1.77897601935572e-51 2.8585266875721e-50 106.194723126167

GREM2 -3.76490449819004 -2.72453293104913 -16.6358692278104 1.88561023513963e-51 3.02764441355667e-50 106.091518713516

GAL3ST4 2.99537536894068 3.51574931273954 16.625950433589 2.11247008921987e-51 3.38941327898104e-50 105.908783709852

FAM217A -2.55875688937511 -3.5588614632834 -16.6076810757895 2.60398745590651e-51 4.17497710006786e-50 105.843426907062

CSNK1E 1.11422795912564 6.53909390547513 16.6046373830802 2.69631575592181e-51 4.31983784561285e-50 105.174886243795

CDC6 2.34099011856255 1.82851189131859 16.5977371318782 2.91791008898608e-51 4.67143510436724e-50 105.668503425891

GLOD5 -3.04876020854814 -1.26338511357638 -16.5959381269559 2.97861176830759e-51 4.7641087492972e-50 105.576995372163

RFX8 3.27733520051321 -0.424294499933286 16.5958928160615 2.98015680240208e-51 4.7641087492972e-50 105.724826362012

ATP6V1A -1.68946001934623 7.12283280279241 -16.595757608392 2.98477193897693e-51 4.76799862444904e-50 105.095239551505

PCDH15 -3.90467670579484 -4.88884137702594 -16.5954212368291 2.99628451283777e-51 4.78289302111351e-50 105.705928245051

HS3ST3B1 -1.42309971346355 1.98962371588694 -16.593839257077 3.05102616639673e-51 4.86672078936261e-50 105.322036547807

ATP1B1 -1.66698769842642 9.86195219086806 -16.5902835995381 3.17773400871508e-51 5.06513649106131e-50 105.107914065387

CORIN -1.59339500330234 0.161038806061297 -16.5784259892289 3.63948365252691e-51 5.79691226375151e-50 105.357323227413

CYP27B1 -2.63422317732103 0.286557787900946 -16.5779269321072 3.66032225374677e-51 5.82585740794815e-50 105.237224938597

MTCP1 3.0455945213894 2.4731914293049 16.5777275266781 3.66868187423587e-51 5.83491302748737e-50 105.43481381128

MS4A6A 2.61707705904325 5.77685956993417 16.5762706598149 3.73033865755503e-51 5.92435252060684e-50 105.031791531772

TGFB1 1.5681016168446 6.76143749341279 16.5725983744921 3.89038315253312e-51 6.17404088833016e-50 104.815193530819

CPNE6 -4.20128679865626 -4.03389405238996 -16.5686064197804 4.0721422125613e-51 6.45780288614673e-50 105.36209634653

BMERB1 -1.17136801145607 3.69570510547787 -16.5638539284411 4.2996120063931e-51 6.81359109323483e-50 104.781663459386

PLA2G4A -1.67322724423536 2.30960452613492 -16.5611966798234 4.43227937380748e-51 7.01873921418948e-50 104.882815045006

PAQR5 -1.98849388225214 5.3651252550336 -16.5518479915461 4.93234456577445e-51 7.80496204169942e-50 104.570803166415

RIN3 1.49264341644051 5.03410192513983 16.5511606950223 4.97125672752498e-51 7.86084466473251e-50 104.714013295246

ACTRT3 -1.87470407223561 0.267814206696909 -16.5467404011482 5.22894400622389e-51 8.26233646912587e-50 104.963540830537

LNCTAM34A 2.00730398099323 0.0965728959193101 16.5380479260758 5.77521095233312e-51 9.11890787148379e-50 105.050501909638

PSMB8.AS1 1.47174756898101 3.8187522911779 16.5344741551227 6.01599452262106e-51 9.49224031067423e-50 104.702683130075

STAT4 1.97906570704894 2.12596397986567 16.5341205565542 6.04035630129599e-51 9.52380275990052e-50 104.908232782006

TICRR 2.21240280922783 0.328921524569699 16.530086764168 6.32533802430557e-51 9.95876166031337e-50 104.95721351796

NAP1L6P -3.94196951366985 -3.71911751448565 -16.5280717307867 6.47268401885698e-51 1.01834099254198e-49 104.902253436855

ENTPD3.AS1 -1.22689842396174 1.47617435642988 -16.518796670169 7.19632821957607e-51 1.13137669483738e-49 104.561064695603

BTBD11 -2.55973894116702 1.61902143449251 -16.5180546581296 7.25759466229343e-51 1.14018846984255e-49 104.404169722815

SGK2 -1.97620169455285 4.01708418546811 -16.5145480504644 7.55424576801918e-51 1.18594060896928e-49 104.170205497151

GDF7 -1.91069919660145 2.60534637482235 -16.5060714798732 8.32228979122878e-51 1.30557788088817e-49 104.192605547419

MCM5 1.08349172389192 5.46102204771368 16.5029487797974 8.62446823284658e-51 1.35201222591389e-49 104.077910929169

RASA3 1.48141614382592 5.08048744584092 16.4930613873381 9.65538178426859e-51 1.51253805112273e-49 104.044391570064

RASSF2 2.23346586043368 5.29813239692818 16.4888136154948 1.01352198327747e-50 1.58656847425233e-49 104.060178800562

SIGLEC10 3.35572861778649 3.28032986016794 16.4877821736113 1.02552806661134e-50 1.60421366067699e-49 104.374684693274

AGL -1.07706524016268 5.14434002861583 -16.4861864244906 1.04438300496117e-50 1.63253947120289e-49 103.820607993413

CPNE4 -3.42739869172175 -3.11924368863807 -16.4860843569191 1.04560071287159e-50 1.63327465177862e-49 104.414903837419

EDA2R 2.34991607195913 3.7521970441255 16.4859325572792 1.04741436602536e-50 1.63493901005372e-49 104.244584220831

THEMIS2 2.30052707623377 4.81157571330299 16.4845630336705 1.06391958851591e-50 1.65951711404983e-49 104.085793832659

CCND1 2.0702598057658 9.16292316774693 16.4840050207972 1.07071884433504e-50 1.66893144830626e-49 103.80205093135

CCDC13 -1.77894733096993 -0.260907855410426 -16.4711954169875 1.23929916891786e-50 1.92894620643604e-49 104.158539929261

MYDGF 1.20071991380165 6.21311561476309 16.4697246983198 1.26027619713506e-50 1.9602004082557e-49 103.652191907928

CKMT1B -4.39247736193906 -3.18954029470144 -16.4581116395194 1.43885484394196e-50 2.23636521370296e-49 104.062012829012

LINC00507 -2.05706208818157 -6.2404340769134 -16.4545871194135 1.49789223357188e-50 2.32647043214259e-49 104.127714506475

ARHGEF38 -2.40795494681757 0.755310472340309 -16.449848031773 1.58110236246725e-50 2.45396519367875e-49 103.754458184886

GRAMD1C -1.62268180347469 4.03315079699223 -16.4482461706982 1.6102588813122e-50 2.49744409746738e-49 103.420327252985

CCDC187 -3.4555740926614 -4.95631436506054 -16.4460567821988 1.65097917921613e-50 2.55878354634115e-49 104.019185158484

MTHFD2L -1.03170186726601 2.81312216937032 -16.4455528897048 1.66049558601266e-50 2.57170872013712e-49 103.558472872653

AK7 -1.72277082360575 1.96295169935498 -16.4418133037208 1.73285248496688e-50 2.68187148399301e-49 103.56709446816

INPP5D 1.68829529576534 5.39686939685241 16.433770881937 1.89930191993667e-50 2.9373987867216e-49 103.354264926523

SNTG1 -3.53523743138129 -4.95315500540632 -16.4313307603231 1.95289083312899e-50 3.01814168149702e-49 103.850488384501

SORD -1.81592221773243 4.15011795850884 -16.4292535327333 1.99969735963295e-50 3.0882958586614e-49 103.194995872236

S100Z 2.23163260264244 -2.14702438274518 16.4251766865709 2.09484140864348e-50 3.23294980953997e-49 103.792242895368

ODF3B 3.67249240152967 3.90994077638985 16.422023806021 2.171509007378e-50 3.34890517559855e-49 103.602014657053

TSPYL4 -1.05831872653959 5.01689021856621 -16.4069547437441 2.57843226575987e-50 3.973658695603e-49 102.92186600443

ISG20 2.45547737665461 3.38877822062318 16.4049150284036 2.63906835151733e-50 4.06423965367922e-49 103.37062333411

RAB7B 2.23687772546478 2.1443007974531 16.4044343041545 2.65356517443239e-50 4.08368730682191e-49 103.446257288111

MGC32805 -2.33567069051221 -5.84034987912657 -16.3917047107059 3.0677458103994e-50 4.71776560131302e-49 103.41358216743

ADAMTS7 2.51279240300318 3.25257538031713 16.3865592900506 3.25293303430589e-50 4.99903977487248e-49 103.173417501596

MOB1B -1.46738257918756 5.49655302442444 -16.3767016522459 3.63944378438758e-50 5.58516608288074e-49 102.572558944593

RORB -3.4225942046918 -2.45186523259501 -16.3716175829206 3.85636874799418e-50 5.91391061402925e-49 103.083321157405

MYBL2 3.75414331662806 1.68207807149971 16.3703216773901 3.91369567821929e-50 5.99761512315049e-49 103.13791232642

LAIR1 2.90765353215368 4.83259431713888 16.3665599404914 4.08496617658253e-50 6.25569487434184e-49 102.81373216282

CYP1A1 -4.42133984396846 -4.31100321760289 -16.3661393037939 4.10457665372679e-50 6.28132448276552e-49 103.072034165633

SHBG -1.9506868024541 -1.49197898082773 -16.3648116932799 4.16708943077023e-50 6.37252661515898e-49 103.020674016404

PNMT -3.52376185444692 -3.75710835470899 -16.3573814779413 4.53490551566326e-50 6.93016015620903e-49 102.980601197959

MESTIT1 -3.3151033081858 -3.79051352999492 -16.3560010390825 4.60672743235787e-50 7.03499752476007e-49 102.972129613898

LINC00921 1.46608970182801 0.568515944990966 16.351277749165 4.86116880281974e-50 7.41837443072764e-49 102.897499841703

SEMA5B 3.80124329826463 6.51325223801141 16.3490588964649 4.98549241182091e-50 7.6027889515368e-49 102.493387298254

APCDD1L -3.31284620892246 0.128188410229115 -16.3340240279321 5.9157197028195e-50 9.01507828910143e-49 102.431904361652

SELPLG 2.39882177739482 4.50759652359006 16.3297785450731 6.20845114160956e-50 9.44800019481851e-49 102.380830153273

LOC101927164 2.19935741987746 -1.85873283710881 16.3263771046848 6.45338067231031e-50 9.81389894446744e-49 102.671994206411

BAZ1A 1.23444932009288 4.92909552413037 16.3247456302954 6.5742577409453e-50 9.99076873524879e-49 102.127597435803

CD37 2.46779549828956 4.3671108719907 16.3212588640091 6.84022259896657e-50 1.038051280939e-48 102.311340514375

RASSF4 1.88370249984684 7.19784712564778 16.3203938264928 6.90785203021426e-50 1.04686054380217e-48 101.940612364822

LINC01587 4.54006565815316 -0.104220145803017 16.3170624381175 7.17459474159246e-50 1.08653096942495e-48 102.561000878539

KIF21B 2.34920574631571 2.7255478575732 16.3157997942368 7.27835901208923e-50 1.10148185243204e-48 102.404162289908

CASP1 1.80854869221647 4.55704859884788 16.3120553707243 7.59496548416966e-50 1.14860055865439e-48 102.106282639707

OXCT1 -2.16556694042265 4.83326736432819 -16.3099619966215 7.77792056442701e-50 1.17545572679408e-48 101.82280765394

EFHD2 1.41011566176014 5.88693664524816 16.2968526002334 9.02796868625803e-50 1.36248756699445e-48 101.721279063753

SHC1 1.06387603519728 7.0756869826456 16.2939893539391 9.32662325209386e-50 1.40561860640005e-48 101.630752202561

KLHL6 2.89304747974925 3.14207420750073 16.2920984388613 9.52924292293756e-50 1.43516571740148e-48 102.135881825158

PRMT5.DT -1.64520635788302 -1.45335302018723 -16.2866377257025 1.01393783198901e-49 1.52600436931515e-48 102.148351927716

WT1.AS -3.6884233965665 -2.95221307632656 -16.2816111086578 1.07354089097938e-49 1.61459663390037e-48 102.077304513094

LOC100505851 -3.34232989687644 -5.03572492586665 -16.2800991819247 1.09214335805075e-49 1.64144489707586e-48 102.142677562249

CHODL -2.86070762689265 -0.705758384150674 -16.2759684845783 1.14462298930505e-49 1.71913719486483e-48 101.907692662479

CDT1 2.47202510586417 1.97198836066647 16.2732898904721 1.17999143475359e-49 1.77104071591141e-48 101.982484739919

CDH16 -3.05539390283217 7.74298308018109 -16.2685274443708 1.24558988017297e-49 1.86821384017982e-48 101.419364556796

TRPM2 2.84755406418996 3.31053431966715 16.2662678625265 1.27797430311793e-49 1.91547136118218e-48 101.83099078181

DNAJC6 -1.74909434460078 2.95453324582427 -16.2641023094667 1.30979927990794e-49 1.96182615927542e-48 101.413728195633

HIF1A.AS3 4.05897839635802 0.381103512030506 16.2617046154829 1.34595915889249e-49 2.01460585611491e-48 101.931123453567

L2HGDH -1.49898989083228 3.46005396928256 -16.2615087402036 1.34895682223892e-49 2.01771070748714e-48 101.346741730873

SAP30.DT 2.79970507127287 2.53607099798428 16.2587521039231 1.39185774920847e-49 2.08045604606379e-48 101.799040506236

CASP4LP 2.66966333411665 2.10113107055975 16.2566402928142 1.42564214803246e-49 2.12949814497289e-48 101.794841651083

SYTL5 -3.03096902054777 -0.876560151159449 -16.2533143164883 1.48051747177547e-49 2.20844698366616e-48 101.658443771483

CALML3 -4.44617087240486 -3.72396193407919 -16.2510976110188 1.518256473619e-49 2.26319636548403e-48 101.733879615147

PSAT1 -3.10905016391474 3.00693352933757 -16.2493979041917 1.54784260656105e-49 2.30572627683563e-48 101.196297088987

ASB5 -3.95870611915161 -3.84395767883098 -16.2481891259438 1.56923274729362e-49 2.33599749500051e-48 101.732325218668

PRDX3 -1.01071813815086 7.3913231714918 -16.2458720588907 1.61106289825351e-49 2.39663427607446e-48 101.109778895333

LILRA6 2.57586560546763 0.536990681284954 16.2423934060606 1.67596161941806e-49 2.491482263207e-48 101.698068872483

ARL14EPL -2.88565781522961 -5.65344937373687 -16.2418984361463 1.68540552633599e-49 2.5038182846377e-48 101.717467596053

FNDC5 -1.99091793816929 -0.802424427260607 -16.2340503189138 1.84244304233645e-49 2.73525188887082e-48 101.496793220414

GPR19 2.06396515622013 -1.36713878610484 16.231278111024 1.90133560532408e-49 2.82076625819058e-48 101.594347656969

TMEM131L 1.16514896448041 4.26633889151633 16.2256664965128 2.02636035313853e-49 3.00420982341495e-48 101.088179615237

DPP9.AS1 2.64906305544763 -1.34673274927166 16.2188947439103 2.18820305393278e-49 3.24195263305716e-48 101.45572996414

PLAT -1.90565715828555 5.56137870975733 -16.2185430848481 2.1969513271032e-49 3.25270849262779e-48 100.783921385968

C7 -4.90345055161123 5.45957929240072 -16.2142809881907 2.30579636278944e-49 3.40924004844639e-48 100.794239663637

CD247 2.92978668974553 2.90509635322802 16.213627516754 2.32295391200148e-49 3.43228612839542e-48 101.269013329719

FKBP10 2.62029635608196 6.9487877426817 16.2106842860566 2.40182423746325e-49 3.54642331495165e-48 100.746105657505

ANGPTL3 -3.45419216776954 1.37344539596132 -16.2105106495625 2.40655980589867e-49 3.55101630238377e-48 100.869318833272

TNFAIP8L2 2.42838592535681 2.31095347677174 16.1983287809609 2.76313990465838e-49 4.07441945590415e-48 101.112705263949

FABP1 -4.56256708292488 -0.82548676890991 -16.1963777196937 2.82495745770283e-49 4.16276435085502e-48 100.858403626348

C1QTNF7 -2.20282784065629 1.21734172798364 -16.1925549272343 2.950108454741e-49 4.34425337341341e-48 100.793220133212

ST8SIA5 -2.62790480990848 -0.501853657544098 -16.1915324815535 2.98450917831727e-49 4.38899589998434e-48 100.953130454849

LOC101928940 3.18601069413894 -2.99704016627781 16.1892828907569 3.06161361680874e-49 4.49633349248128e-48 101.127093840136

ZAP70 3.27022350726232 2.34869309482661 16.1865739858442 3.15710343172416e-49 4.63034773781665e-48 101.014241019538

MYMK -3.21901007067463 -4.93724810418492 -16.180991424749 3.36336622462738e-49 4.92294990668333e-48 101.02406241542

BRIP1 2.18394282383183 1.16515711681201 16.1730655006422 3.67951488173899e-49 5.38209094448742e-48 100.880159863631

ZFAS1 1.55625662968974 6.23546582165347 16.1652188526189 4.02169748787172e-49 5.87474299481689e-48 100.215060188455

ABCD3 -1.01862621453656 6.08677805189971 -16.1651290982462 4.02578985993775e-49 5.87679263922644e-48 100.174128677804

CD4 1.91455734780629 6.24056300109917 16.164113967281 4.07236528564408e-49 5.94081432491189e-48 100.225064049385

CORO1C 1.11554093952309 7.21143716352933 16.160679747361 4.23395441240018e-49 6.17242199961182e-48 100.121507203452

CD244 2.50951027518698 0.920357977331897 16.1586657917978 4.3316763496724e-49 6.31067488462607e-48 100.739275654178

PCGF1 1.02511453821539 3.75118863812451 16.1580908929785 4.35998249160735e-49 6.3476813716919e-48 100.389988217777

TOM1L2 -1.01512788651029 6.01560996912796 -16.1571028611655 4.40906181907407e-49 6.41486204608693e-48 100.082908321708

CAT -1.50268146588251 7.36459777943681 -16.1539525478468 4.56926131998407e-49 6.64351747342727e-48 100.07935113306

CHGA -3.46452951786902 -3.60506421491309 -16.1535059541581 4.59243671035958e-49 6.67277389837021e-48 100.672570393388

HK3 2.69481317451739 1.84550989419888 16.149368188403 4.81281677612737e-49 6.98833787433299e-48 100.59819079265

PEX7 -1.06720233603737 3.08284360384387 -16.1490461496734 4.83040499245401e-49 7.00921914343276e-48 100.153595820637

COLCA1 -2.18983604155971 2.57335118925438 -16.1482058381521 4.87660140810002e-49 7.07155743670934e-48 100.119238067665

PKHD1L1 -2.97579482909576 -2.45310870638962 -16.1468246779611 4.95349091292375e-49 7.17829157295243e-48 100.56379415032

AUH -1.27807058962563 4.48590552922458 -16.1421315024748 5.22391415298605e-49 7.56515546621631e-48 99.9354997176305

DGCR5 4.11278765193774 2.00639782850289 16.1407188216454 5.30816205981619e-49 7.68207056246114e-48 100.54006181141

SLA2 3.15476213857958 1.66104179353842 16.1390158351444 5.41152715549136e-49 7.82647934672089e-48 100.50754966212

PLCXD2 -2.32436188038484 0.877878073394251 -16.1314116568857 5.8981345978833e-49 8.52459889996982e-48 100.139768040824

LINC01485 -3.72331828356096 -3.05570005633842 -16.1307008504654 5.94579372451982e-49 8.58780107481372e-48 100.378729900779

HVCN1 1.47152351528274 3.16630573013111 16.1269712771972 6.20222923496079e-49 8.95226654369869e-48 100.163494063906

SLC25A5 -1.58014428745966 7.57245798744775 -16.1188656893348 6.79824069649095e-49 9.7996011834048e-48 99.6898449881243

CDK18 2.22763082836272 7.78131558402247 16.1076942670196 7.71444465119389e-49 1.11129702678009e-47 99.5293054054693

PITRM1.AS1 1.86610295457274 0.0843934108095847 16.1064962467969 7.81973641268203e-49 1.12572266025257e-47 100.163726247797

ANGPT2 3.23627308720113 6.16320995153534 16.099978723671 8.41818091337302e-49 1.21107641540448e-47 99.6624467444702

FCER1G 2.64041899786515 5.04341385684764 16.0981627899465 8.59291621596001e-49 1.23540130307483e-47 99.7197291497819

MYH10 -2.03020070088941 6.26486464153196 -16.0917827443505 9.2360198190844e-49 1.32698712101546e-47 99.3634796864958

PIMREG 2.59368517021495 0.336577986430517 16.0890371960534 9.52734411348062e-49 1.36794382990731e-47 99.9750552418349

RNF157.AS1 -2.7389426825693 -1.10944524902789 -16.0851244725337 9.95842861916592e-49 1.42890046365485e-47 99.7991760648656

ICOS 3.59940652177707 -0.34845337560993 16.0823657708583 1.02740154924951e-48 1.47321562045601e-47 99.9133665014344

SLC7A4 -3.38594680294453 -3.65639976702034 -16.0805628124952 1.04856408225025e-48 1.5025751402895e-47 99.8550922962536

DLG2 -1.74484177304841 1.29345195371007 -16.0769452482151 1.09234675912993e-48 1.56428923507643e-47 99.5223987803048

CDKL2 -1.75811952324132 2.25083269696674 -16.0760270649985 1.10374663615085e-48 1.57957925604483e-47 99.3804143961533

PCSK1N -3.47245460256859 1.61196335077642 -16.061188887253 1.30532991526332e-48 1.86562293252121e-47 99.1651150257247

PTGDR 2.41281763811438 0.156572883166489 16.0600412910332 1.32237165387438e-48 1.88874429752397e-47 99.6498541586593

HLA.DQB1 2.40006993598763 7.71173043915506 16.0588393569444 1.34045837003129e-48 1.91266711036841e-47 98.9829116064838

SLFN13 2.15422355243486 5.04526543355137 16.0588121066139 1.34087128224244e-48 1.91266711036841e-47 99.2199403871328

FCGR1CP 2.65655751696815 -1.49461581592829 16.0571660341274 1.36605030793546e-48 1.94731228827878e-47 99.6351345843524

ADCY6.DT -2.4628556616794 -2.75590907797569 -16.0547331128305 1.4041318092572e-48 2.0002928570859e-47 99.5607536698512

NCAPH 2.15435978764866 1.06596437965773 16.049929640138 1.48245673264397e-48 2.11049687156148e-47 99.4976559945382

RNU6.796P 3.17067574124609 -2.73001004256656 16.0498127381035 1.4844163039035e-48 2.11191077403667e-47 99.557116304717

LINGO3 2.60386894640437 -2.00121777387047 16.0450517812391 1.56645668530886e-48 2.2271813886828e-47 99.5013589145229

PLIN5 -2.38305620507743 0.436834626029165 -16.0433180376365 1.59744321836351e-48 2.26976116065655e-47 99.1968194802944

TSGA10 -1.14348375217843 1.85481283852578 -16.0369709997132 1.71619104071642e-48 2.43690206710694e-47 99.0634093603342

DMAC2L -1.02952499163059 4.95172599876863 -16.0360759149221 1.73363135968105e-48 2.46006792877338e-47 98.7268997840698

CADM2 -3.43827157674108 -4.00398771151453 -16.0278622552928 1.90215958380235e-48 2.6974622572896e-47 99.2731084879545

ASF1B 2.37293131435592 2.2683062639383 16.0233952732817 2.00057693200757e-48 2.83518856648258e-47 99.1418108552048

SLC44A4 -2.83778551638759 3.88653913941564 -16.0219719852973 2.03299009248819e-48 2.87925680435154e-47 98.590458921472

POLQ 2.38764263090164 0.247016799412864 16.0218019772381 2.03689662823396e-48 2.8829211150775e-47 99.2175476882329

TMIGD3 3.1863723490526 3.34745789450586 16.0155488869789 2.1859085285513e-48 3.09182259381434e-47 99.0198719092703

FXYD5 1.83672561220965 6.01878315833555 16.0113672386493 2.29157825922076e-48 3.23918885502919e-47 98.5192006720502

TBC1D9 -1.26817449338508 5.57696572934427 -16.0078590340223 2.3841515253859e-48 3.36786446569219e-47 98.4001462027662

CEP55 2.7599345243763 1.6886500721192 16.0075975103229 2.39120014063446e-48 3.37563931997964e-47 99.0147817310117

RUFY4 3.72879059936113 -1.52032106241433 16.0056270478877 2.4449810949845e-48 3.44933323877962e-47 99.0577568321896

FRK -1.43415408239076 5.1592147138611 -16.0041056188321 2.48733060632363e-48 3.50681520903163e-47 98.3607489920443

ARHGEF3 -1.05394693085111 5.11977547843486 -16.0035894345227 2.50186443111707e-48 3.52503181258551e-47 98.3567111089946

FAM124A -1.55126499060665 2.46000912626636 -16.0001105467055 2.60205168685559e-48 3.6617174410044e-47 98.5148343442081

CHST11 1.8390860823567 5.08748967683844 16.0001045689161 2.60222723922566e-48 3.6617174410044e-47 98.5065285562243

SLC16A10 -2.11084793401129 2.26760338962094 -15.9960040863613 2.72547550337177e-48 3.83267800355104e-47 98.4431616739555

LOXL2 2.88392792768709 6.1335644659783 15.9890493626302 2.94797443876602e-48 4.14289938330249e-47 98.3689204676513

RGS1 3.50540451839096 6.08346193140466 15.9860077190997 3.05089561198552e-48 4.28478289259123e-47 98.4111269554697

IGSF5 -3.200353556431 -3.49843995755926 -15.9722093866044 3.56470080505077e-48 4.99996191866332e-47 98.637176360646

MSI2 -1.20077977328828 6.39967124026264 -15.969036547048 3.69456238641725e-48 5.1787858775097e-47 97.9691086282616

CEMIP2 1.25976095963839 6.75165668085827 15.9643365222053 3.89565510806391e-48 5.4571635305462e-47 97.9159086655361

LOC100507156 1.99943536518594 0.662698802865037 15.9633744374615 3.93814586459813e-48 5.51315192690986e-47 98.538191174526

LOC101928335 5.41629746938577 -1.14579406642083 15.9610041029942 4.04481593493544e-48 5.65885804264687e-47 98.5555497289186

SLC19A2 -1.64295516733107 3.49617133113165 -15.9595803865351 4.11026699462614e-48 5.74674757732342e-47 97.9319067926598

FCRL6 2.69461074555415 0.814963056954323 15.9544539017851 4.35481751479019e-48 6.08477155695077e-47 98.4511078070542

C11orf21 2.65161484395023 0.47993940957288 15.95367211337 4.39336629573204e-48 6.13471141601484e-47 98.4516597569844

PRPS2 -1.0656270906191 5.18490205358684 -15.9529936598185 4.42709562547982e-48 6.17786211389595e-47 97.7863367265195

PHYKPL 1.59706628812533 5.93109229956853 15.9480429161954 4.68117017524942e-48 6.52824580981018e-47 97.7933109633932

SLC12A5 2.3600625003356 -0.321636325991187 15.9448239945664 4.85411989770821e-48 6.76512003345775e-47 98.3642569491152

GASK1A -1.89209667290684 -0.41168744883152 -15.9433193417091 4.93713588392714e-48 6.876432789768e-47 98.1995423194877

MS4A7 2.58349221555735 5.38364731402009 15.9431440328119 4.9468998528224e-48 6.88564347030114e-47 97.9165862021533

PCCA -1.52242468719492 5.3932057744061 -15.9409449115043 5.0710308630511e-48 7.04944258589412e-47 97.6492666626111

COL5A3 3.54233520165158 4.5795178779868 15.9291245225995 5.79346590048277e-48 8.03838795703175e-47 97.9689374474758

RELB 1.2937046804506 4.48269495820062 15.9240076105117 6.1372292714411e-48 8.50995376071081e-47 97.6733097433372

ZNF469 2.25226828698782 1.95410841207103 15.9064171398776 7.48181574305257e-48 1.03612242986646e-46 97.8401579934117

FERMT3 2.24932046614585 4.48858172436901 15.9055753853174 7.55306209761664e-48 1.04532657390257e-46 97.580973055555

CCDC127 1.03671751280109 5.24649277009894 15.9019560852317 7.86719403446859e-48 1.0881126027547e-46 97.2981795024164

IL32 2.34849310381143 7.44833088575165 15.8973808334897 8.28301667112929e-48 1.14490046372036e-46 97.1724363269684

CTSH -1.44745785945144 7.17991256604093 -15.8963475519197 8.37992150554879e-48 1.15756273489733e-46 97.1725346001107

PYGO1 -2.14680280932549 0.969566787323386 -15.8947176686505 8.53508224179262e-48 1.1782511195824e-46 97.4839960317013

APOBEC3F 1.12693302739263 2.83640901282248 15.8927578757943 8.72544955408928e-48 1.20377051202975e-46 97.5377641042277

CDH18 -3.44951772799562 -5.11745870950066 -15.8915953020202 8.84037369891749e-48 1.21885606588293e-46 97.7724299190907

RNASET2 2.99202469847832 6.99844156929664 15.8834185868498 9.69247598648808e-48 1.33549607649889e-46 97.0855551116394

MAGI2.AS3 -1.57393640801552 4.34026742933279 -15.8827940743933 9.76082954791108e-48 1.34406684379648e-46 97.0165168712418

MILR1 2.25490748030611 1.83838099960263 15.8801304310707 1.00578098661363e-47 1.38408891060879e-46 97.5538902802989

ZNF683 3.54009322375663 0.370013051727853 15.8789425499419 1.01931420113359e-47 1.4018296562223e-46 97.6255515853686

IL2RB 3.32409873868065 4.09001793698566 15.8786610558085 1.02254772632093e-47 1.40539216750259e-46 97.4300142999123

PYHIN1 3.21531638760419 1.29418107165606 15.8758852873983 1.05498617332131e-47 1.44906428947772e-46 97.5680718804901

CKLF 1.15091931503979 2.49410398846387 15.8756957025456 1.05723881058563e-47 1.45124621405325e-46 97.3878975321202

CHSY3 2.41662739750007 1.94489473721717 15.872830919086 1.09186805021753e-47 1.49784008169514e-46 97.4725212017772

SUCLA2 -1.08393916362312 5.37152437357403 -15.8718268615198 1.10427094801266e-47 1.5139042049511e-46 96.8721743990274

PDYN.AS1 -2.87668095305212 -4.67314440111586 -15.8690955856108 1.13872566718648e-47 1.56016125423361e-46 97.5212585653666

EPS8L1 -2.4994802497858 2.00205495191006 -15.8655902331049 1.18452139962466e-47 1.62188885626552e-46 96.9891183869457

PRSS3 -3.72256297999935 -3.19571598454202 -15.8616044945176 1.23882956093283e-47 1.69412655788893e-46 97.3628135550392

NLRC4 1.87254451282449 1.75571091171379 15.8605127105981 1.25413450751682e-47 1.71398382693965e-46 97.3206870684307

KCNH1 -2.51436871948495 -3.09574557047697 -15.8584823997253 1.28309936735708e-47 1.75247315467839e-46 97.3708979302497

HMGCS2 -4.74972351927981 3.277751512625 -15.8573952848201 1.29888176857327e-47 1.77292087998948e-46 96.7786731762511

KRBA1 2.60534903723891 5.4450358394315 15.8549452038915 1.33516507096238e-47 1.821308507849e-46 96.9234326411615

ZMYND15 1.70535754308185 2.1885373808546 15.8546423390352 1.33971986402008e-47 1.82638167114353e-46 97.2157027061043

YEATS2 1.08248065017616 5.66914202284399 15.8486807115083 1.43260068044423e-47 1.95056839063849e-46 96.6637289755091

TMEM38A -1.85348811309916 2.3022359332045 -15.8466734169275 1.46529798346777e-47 1.99260465667214e-46 96.7869958717398

KIF1A -3.84708324806083 -3.45031163944304 -15.8438788863769 1.51206222509958e-47 2.05491889335206e-46 97.1701097925379

UQCRC1 -1.2505612969991 6.94084978801577 -15.8424811703829 1.53600756306686e-47 2.08616365914854e-46 96.5590030087947

KIF14 2.63122751289214 0.323994369975132 15.8409954394275 1.56187570018155e-47 2.11997948298555e-46 97.193597705611

HROB 1.53352906139156 0.772439161209187 15.8406157014404 1.56855679104841e-47 2.12772635349354e-46 97.1415869730522

TNFRSF4 3.24075569390635 2.42143661364608 15.8389801065082 1.59766079354813e-47 2.16586112415678e-46 97.1055458536884

PCYT2 -1.427479516158 4.56491055507878 -15.8375217213621 1.62406556288904e-47 2.20029167673988e-46 96.502987143571

APOL5 3.06664872499047 -3.15263616854821 15.8362086686738 1.64821127968101e-47 2.23162088567963e-46 97.1635598829975

SLC25A15 -1.33110103205161 1.72735261506415 -15.8295952841509 1.77537914061345e-47 2.40082675493971e-46 96.7286291199648

FAM163A 2.99990804705676 -1.2176639028126 15.8279586256759 1.80833287129971e-47 2.4438774419612e-46 97.0653669801151

FTH1P22 3.45129539433416 -1.75053708308653 15.8276639446783 1.81433070794022e-47 2.45046779731877e-46 97.065444746927

LINC01785 -3.09952578348599 -5.7600735986766 -15.8270040399188 1.82783427341704e-47 2.467181122729e-46 97.0567852143271

HNF4A.AS1 -3.83153394198888 -3.88689881004347 -15.8255506553282 1.85792939943348e-47 2.5062550102358e-46 96.9845840156689

STAMBPL1 2.42517080678217 3.4412995209588 15.8246368769058 1.87710354832415e-47 2.53055791742921e-46 96.8180151649119

PLXND1 1.56526746023252 7.69149368280546 15.8232365263672 1.90687141546187e-47 2.56910364007942e-46 96.3251996842025

STK32A -2.9669005018338 0.657872867684546 -15.8119106965289 2.16559931702904e-47 2.91229796154065e-46 96.510002913739

IFFO1 1.81535156493087 4.30819846649026 15.8117640585072 2.16916915786964e-47 2.9153046498724e-46 96.5033946567

JAKMIP1 3.41690412001109 -0.165100571013503 15.8105571707577 2.19877422910361e-47 2.9532767811064e-46 96.8649119321135

SLCO1A2 -4.2313037666066 -3.06284183287007 -15.8092048207927 2.23242668374753e-47 2.99663515478715e-46 96.7513336166331

NFAM1 2.22549786503999 3.55164301153091 15.8069185869907 2.29049104674936e-47 3.0708037327984e-46 96.5917186250523

SOX11 4.9110715967518 0.899636756980051 15.8029152977141 2.39581656427539e-47 3.21004165414532e-46 96.7756969411732

TNFRSF18 2.93205647278652 -0.293502251322544 15.7815783400774 3.04428123751441e-47 4.07638957618888e-46 96.5408878814323

SCN1B 2.05052494284881 3.82852099637937 15.7806766895207 3.07524431952355e-47 4.1153284822136e-46 96.2489365797013

BMP1 1.89198767068373 5.4175089948256 15.7789227497596 3.13637829673712e-47 4.19457006845754e-46 95.985753059029

PDILT -2.72106686172534 -4.7916913252359 -15.7757795368347 3.24898487120739e-47 4.33985735883222e-46 96.4814652794165

TP73 2.74832058159796 -0.375190313402965 15.7689693131921 3.50699191216189e-47 4.68163068151947e-46 96.4000885280158

IFNAR2 1.16792279257556 3.46057175638429 15.7675025032823 3.5651839711027e-47 4.75640813922511e-46 96.0593228200073

E2F8 2.95289399622803 0.163003865694672 15.7541426652192 4.14158496557217e-47 5.51866196662491e-46 96.2300637554951

MAP3K9.DT -2.45491118320423 -2.09996016263276 -15.7519463423259 4.24486765914835e-47 5.6528393025819e-46 96.138696379783

DNM3 -1.2787390676554 2.69419684259963 -15.7515956194891 4.26159678294429e-47 5.67166105345199e-46 95.7217981403391

PRKCQ.AS1 -1.59643280841251 1.66068520415204 -15.7485598954599 4.40917406626369e-47 5.86449670542059e-46 95.8098995855908

C11orf53 -3.47642641169506 -5.52309914760433 -15.7466644331995 4.503893596801e-47 5.98683617827811e-46 96.1571874330854

ENAM -2.72859523833266 2.07824039708139 -15.745381897882 4.56913293904745e-47 6.06986395848048e-46 95.6097532405203

RAP2B 1.24373338520709 5.33008773899484 15.7374960638133 4.99150513567568e-47 6.62291206617612e-46 95.4648213423356

MORN4 -1.01971456771487 3.24630454071949 -15.7371764284232 5.00942322805588e-47 6.64265326472726e-46 95.5089409877164

SASH3 2.77729400422365 4.11466402507481 15.7334013890534 5.22596150564724e-47 6.92558743377254e-46 95.7572136559511

GMIP 1.63410097644933 4.03323407411409 15.7307136689421 5.38579791975002e-47 7.1287608685825e-46 95.6206420977619

CASC2 -1.25443300403573 1.79302141544898 -15.729893272923 5.43555089913275e-47 7.19025991517845e-46 95.6136372568462

PCCA.DT -1.37051059122175 1.08280909890665 -15.729809732436 5.44064287087231e-47 7.19264178204311e-46 95.6974935103581

PREX1 1.39616418839507 6.35141350897737 15.7273193156152 5.59464436668883e-47 7.39176320104298e-46 95.2778794118637

PRELID2 1.9319678073929 2.71039057513024 15.7257524983283 5.69375355002442e-47 7.51816292016216e-46 95.7471255620947

MXD3 2.48589690820689 1.6440407502542 15.7253139314859 5.7218075747249e-47 7.54608696019694e-46 95.8456542920371

DTX2 1.28564996921021 3.57352656630048 15.7211267100733 5.99669511595596e-47 7.90384670500516e-46 95.5398880934295

AP3S1 1.03401654392424 5.55549704022233 15.7080532898229 6.94263593587459e-47 9.14511290576657e-46 95.0958627339121

CLMP -2.79328945280076 0.931399316326701 -15.7064875043223 7.06548046869198e-47 9.30132196881481e-46 95.320668255981

LY86 2.78484217808815 3.08442392001589 15.705598754015 7.13617013845095e-47 9.388725227909e-46 95.5454053730393

PALM3 -2.36156909312587 2.9407770507503 -15.703144928395 7.33502797980564e-47 9.6445467173702e-46 95.0761282242895

KIFC1 2.13961746614515 2.00152642965802 15.7029526285702 7.3508435630525e-47 9.65953002906713e-46 95.5585738955929

GNLY 3.44070462499225 2.82363174650394 15.702500212304 7.38818656900899e-47 9.70276689258134e-46 95.5650738295918

IL16 2.07171917273302 4.24075125606207 15.7002221411283 7.5791168376573e-47 9.94753394914864e-46 95.294961944997

LOC105371264 -3.05386542100197 -1.71279099696231 -15.6870709743278 8.78160906165639e-47 1.15188777205508e-45 95.3608272914911

BATF 3.11922348111491 0.938962849961716 15.686995050186 8.78907685300425e-47 1.15217574366348e-45 95.4688675441867

SPI1 2.45642334855206 4.53506798933152 15.6860893890695 8.87864643807543e-47 1.1632197878373e-45 95.1454718368006

CDCA2 5.27076552624993 3.33081940609518 15.683832809301 9.10580263485256e-47 1.19226545823507e-45 95.4061231605543

RASD2 3.28019520886818 3.18746130218926 15.6818475917645 9.31043136203006e-47 1.21832848236193e-45 95.3008120438509

ECHS1 -1.28624823020088 7.2347935025089 -15.6629984178948 1.14969850227951e-46 1.50355244585961e-45 94.5590848295035

BID 1.111091425185 4.47717804521241 15.6568857337801 1.23107389793784e-46 1.60901063945189e-45 94.6637258728981

DOK1 1.28861012587061 3.63426040715042 15.6567534268346 1.23289735162617e-46 1.61043071279657e-45 94.8108885348573

P2RY1 2.67742416164961 3.36097679468364 15.647653014513 1.36500643807936e-46 1.78192865539714e-45 94.8649772149157

ASAP3 -1.372995990368 3.91970798989191 -15.6471343966212 1.37294679755848e-46 1.79064043179166e-45 94.4104676806623

SLC24A4 1.71629610303756 0.25653888459189 15.6471101792854 1.37331870392295e-46 1.79064043179166e-45 95.0119195234821

SPC25 2.25844770405134 0.543724113579943 15.6451357445359 1.40398074365723e-46 1.8295283954169e-45 94.9947981252622

KLK5 -3.65093363553314 -5.09274002701162 -15.6411383235282 1.46816564354755e-46 1.91202764055093e-45 94.9733990521496

CSF3R 2.94616436977688 3.63296634782305 15.6408294859089 1.4732446553371e-46 1.91749943139259e-45 94.7854430735271

LOC613206 2.76893342617868 -1.71457641299506 15.63888420873 1.50564122745221e-46 1.95849867520911e-45 94.9603112689145

PIF1 2.3526395296604 -0.231839708358286 15.6386858318553 1.50898466124476e-46 1.96168005961819e-45 94.9447851217877

GPC5.AS1 -3.08148166267648 -5.40551367480148 -15.6352909031086 1.56736435245054e-46 2.03636225886455e-45 94.9185844544238

RELN -2.8225527655867 0.946788872760715 -15.6327155308102 1.61314905428998e-46 2.0946016805347e-45 94.4901727124103

MLC1 2.41206815551893 -0.580380999544062 15.6322234037126 1.62204869370729e-46 2.10490677574735e-45 94.8774281022266

RUNX3 3.05958698965839 4.00725397402825 15.6289752679644 1.68202925021856e-46 2.18144719317663e-45 94.62851069672

NAP1L3 -1.78070025640886 1.13267668998245 -15.6288483044734 1.68441818796444e-46 2.18324974268013e-45 94.5219276721022

LINC00861 3.21511549122703 1.01422483182454 15.6247759201015 1.76286598561338e-46 2.28357500791993e-45 94.7753224338687

TESPA1 2.83718339699429 1.57618225119644 15.6184143123525 1.89276807281815e-46 2.4489437948665e-45 94.6711013840821

CBLN2 -3.63744028342476 -2.8249931780817 -15.6181097455253 1.89922134617906e-46 2.45583929455923e-45 94.63498940027

SLC43A1 -1.40617402884785 2.55127560926417 -15.6175647482885 1.91082377501924e-46 2.46894900562837e-45 94.232683058302

CENPU 1.97635469381892 2.25211830223439 15.6141238708933 1.98572668781946e-46 2.56163431575671e-45 94.5435058754412

MGAT4C -3.08547714728499 -3.41548764345464 -15.6134593983276 2.00052522110394e-46 2.57920222163919e-45 94.6310981300167

GGTA1 2.220483208959 4.05641388490683 15.6106963474177 2.06325057224204e-46 2.65850322849087e-45 94.3398598867645

LINC01447 -2.38795238881932 -5.18364907680974 -15.6096478847188 2.0875624225122e-46 2.68824405534232e-45 94.6353459863733

RPS19 1.25823159454895 9.12179310372256 15.6084432481335 2.11584865402365e-46 2.72306481957472e-45 93.9491888308364

PSORS1C2 3.10665144573454 -2.36332721824129 15.6068112705715 2.15478016194636e-46 2.76990652229493e-45 94.6068493724178

LINC02526 3.49241995570688 -2.69213247015597 15.5929411228767 2.51586346268221e-46 3.23026816979991e-45 94.453277256078

CKAP2L 2.52431244983928 0.904503150280324 15.5912894126337 2.5627004672595e-46 3.28847288966658e-45 94.3892880425837

EXOSC7 -1.05509253731447 4.01637315787263 -15.5887970929002 2.63502599641459e-46 3.37929713026104e-45 93.7678673273307

LINC01503 -1.7281189129833 2.09324523083377 -15.5845254288885 2.76375411259984e-46 3.54022969652077e-45 93.9032695970535

CORO1A 2.65505935608306 5.49806177032339 15.583495189514 2.79572831962326e-46 3.57908910185865e-45 93.8856468554975

ACADM -1.57292223701219 6.25940276131839 -15.5805315716659 2.88977869280157e-46 3.69732633336022e-45 93.6254875775305

CLEC3A -2.60435241907971 -5.58133398859504 -15.5789195478337 2.94225481766702e-46 3.76226416211102e-45 94.294792923214

CRYAA -2.71569110592062 -5.94408035838154 -15.5765444873615 3.02130696303853e-46 3.85883232397902e-45 94.2691688394165

MMUT -1.13297729031519 5.58111606962644 -15.5732166888451 3.13564700738314e-46 4.00252885819765e-45 93.5341620997887

BDNF.AS -1.0549368671449 1.51642117327786 -15.5720669808879 3.17614499307033e-46 4.0494922131602e-45 93.9135940916942

LRRC19 -2.81870881788399 3.23182967674698 -15.5701947659974 3.24321262075757e-46 4.13259040241488e-45 93.5605880744148

UBE2L6 1.09141075656687 6.60303560326341 15.5669357675218 3.36334580961488e-46 4.28316992875956e-45 93.4702364890858

ITPR2 -1.54530343864173 5.47216147905038 -15.562802047983 3.52212956559335e-46 4.48276630150912e-45 93.4206659438785

MS4A14 3.0664648440797 1.21939583323391 15.5515721951626 3.99225980520056e-46 5.06636780040928e-45 93.9526035168361

IGSF6 2.79862390699656 3.77364284716868 15.5489234447489 4.11197744390043e-46 5.21526657466954e-45 93.7412912759009

FMNL1.DT 3.70774100085864 0.231843480586017 15.5487302684468 4.12084741916536e-46 5.22348484054644e-45 93.9481216030345

HOGA1 -2.30201436072491 4.16017666780625 -15.5440604162429 4.34117579813036e-46 5.49957766472711e-45 93.2331830355015

P4HB 1.12317819512602 9.7103809665599 15.5413805988812 4.47287909726082e-46 5.65986258902379e-45 93.2204470673414

MYO1G 2.9799414201591 3.48157277561345 15.5315763232247 4.98960841525383e-46 6.31006439227673e-45 93.5870697847379

LINC02783 6.22845700296553 0.0850380477267789 15.5296169492153 5.0998057830736e-46 6.44569437695242e-45 93.7410102422294

SLC37A2 2.93978326816546 3.64188530413715 15.5285454754321 5.16109004842941e-46 6.5156152991524e-45 93.5364255731373

ZBP1 2.8472607086659 0.460328674905476 15.5279466218828 5.19566169364655e-46 6.55547315192022e-45 93.7082598418703

BCAR3 -1.03568627655369 4.4022236312128 -15.5244264965716 5.40359837439994e-46 6.80996743228154e-45 93.0240512919786

HCK 2.25953776538128 4.18688702215385 15.5204180433384 5.65050736445503e-46 7.11703385795019e-45 93.3250080039643

HPCAL1 1.70512582689724 7.40210673034992 15.5175629203244 5.83320744341855e-46 7.34291948508212e-45 92.9172196333556

HSPA6 2.26131110183161 1.96980369185048 15.5111968738961 6.2620690200876e-46 7.87823801358517e-45 93.4335420163499

CSTA 2.58152440002425 1.24682505231558 15.5048542438033 6.7206245200091e-46 8.43571554484543e-45 93.4183935402445

VAV1 2.5535315278967 3.30837827736376 15.5013603670985 6.98737609947819e-46 8.76047790601819e-45 93.2374548007611

CCL11 -3.81638912032242 -2.72913309600339 -15.4936880333355 7.61078849922529e-46 9.53661474045701e-45 93.2381516632012

GRHL1 -1.57008495038254 1.4410642068675 -15.4929121741265 7.67684021577816e-46 9.61386757795989e-45 92.9949221619011

FBXL8 1.80757460158572 2.62212353429182 15.4878370981801 8.12323077468432e-46 1.01612456851274e-44 93.0994111994569

CHFR 1.09871297625242 4.37376732451076 15.4876433377082 8.14077726625985e-46 1.01773687413945e-44 92.7954518306635

RAP2C.AS1 -1.2122716322737 0.900045243069074 -15.4842202759896 8.45707066219528e-46 1.05546753387181e-44 92.9977324974442

TCIM -2.27154260771506 5.30042888750481 -15.471997852313 9.68981534340759e-46 1.20862748116145e-44 92.4189847282808

KCNIP1 -3.31020289370431 -1.59400707664083 -15.470013155364 9.90625858736494e-46 1.23491995955326e-44 92.9242343005074

MRLN -3.4474135330156 -4.41585587919101 -15.4613383393513 1.09103370633945e-45 1.35931354530422e-44 92.9648588736129

MAL2 -3.56953664553808 4.93392727353525 -15.4531765528919 1.19475232471205e-45 1.487687894697e-44 92.2304236250217

MAPK10 -1.16053519768903 3.98750377934704 -15.4511492210469 1.22200523130397e-45 1.52075628244223e-44 92.2334183400912

FASLG 3.34169745604033 0.439291105586746 15.4505056491848 1.23078560567634e-45 1.53081148781133e-44 92.856127415213

CDH26 1.59096638787226 -0.413449874163218 15.4462978392125 1.28976503184301e-45 1.60325570198324e-44 92.8037117082447

CLDN10 -2.60054811683133 4.4609087634926 -15.4385518825524 1.40580674231998e-45 1.74551674658628e-44 92.0558402484655

LOC101927020 -2.47597169761203 -6.07698952308193 -15.4362005908094 1.44305007166626e-45 1.79006467751522e-44 92.7154357125023

TWSG1 -1.19159810354539 5.8382295253364 -15.4361835783517 1.44332309604256e-45 1.79006467751522e-44 92.0125162805926

CDS1 -1.66706682522996 4.40303636929372 -15.4330381371947 1.49469849181436e-45 1.85273092124897e-44 91.9964680460734

FCGR2A 2.09191012354239 5.32418951347432 15.4294505095171 1.5555265761441e-45 1.92703640977761e-44 92.1295340760236

AQP3 -2.55829081747163 6.60321270280226 -15.4286755046693 1.56898767454831e-45 1.94261119840817e-44 91.9650988559309

ANK2 -2.84366237119003 5.62899739189106 -15.4272857459247 1.59341813321233e-45 1.97174215544106e-44 91.9365686756041

LUCAT1 4.93936319552541 -0.0636785913513009 15.4229190992867 1.67267557569723e-45 2.06864625668996e-44 92.5598183058692

C1QTNF6 2.20324906080023 4.046703479271 15.4177050960264 1.77247995183234e-45 2.19083735222807e-44 92.2002525657613

SOX30 -2.19545025546421 -2.25095251499993 -15.4148653144998 1.82931421897905e-45 2.25753077255992e-44 92.4126408940608

SPINT1.AS1 -1.66538637270773 1.95899598649828 -15.4148543617634 1.82953690486597e-45 2.25753077255992e-44 92.0454078850484

PLEKHO1 1.84307475040771 5.31893244855036 15.4121141458673 1.88610794392743e-45 2.32602239834346e-44 91.907401610391

STK33 -3.16471964008495 0.715448273027971 -15.4085437930751 1.96244265438498e-45 2.41879635229977e-44 91.9977650027078

CD33 2.12544713217647 2.17494615990446 15.4063409789683 2.01106842132863e-45 2.47733248090725e-44 92.2503277595956

APOC1P1 2.98671148414034 -3.04485530342342 15.4018056986635 2.11499910590991e-45 2.60389157529292e-44 92.3369683243509

CFAP61 -2.07639070173678 -1.61808848091727 -15.399229278056 2.17640911950329e-45 2.677988090569e-44 92.2105014977572

LSP1 2.52700539393227 5.14646461633315 15.3919133867437 2.36065408315921e-45 2.90142709107301e-44 91.7954682137683

CKMT1A -4.18324619219962 -3.24875377835947 -15.3885959093815 2.4492520104757e-45 3.00862867818581e-44 92.0840792718013

LST1 2.3895741845731 3.71834078343062 15.3848644755995 2.55287442504191e-45 3.13239555364631e-44 91.8948779151078

PCLAF 2.32666879514867 1.89534692216599 15.3752669192265 2.83993232042918e-45 3.48070897354677e-44 91.9384376058532

LINC01704 2.7247888274007 -2.82631270877922 15.375090057795 2.84551371713778e-45 3.4855948016038e-44 92.0418429147069

RASSF9 -1.90929582217941 1.87536759680217 -15.3701042318639 3.00743924248061e-45 3.68188065915568e-44 91.53155371019

FGD2 2.7021851892614 3.29150917050872 15.3626576217705 3.26655516183786e-45 3.9946295440203e-44 91.7131867120454

SLC12A5.AS1 2.67809832219133 -3.24132247648736 15.353864282743 3.60134566031505e-45 4.39911720038372e-44 91.8079869295551

SNHG26 1.92326614833324 0.305001258521268 15.3494697522684 3.78127259733319e-45 4.61374372247472e-44 91.7182018682199

NCF1B 2.36017438621688 -0.0587553446728523 15.3488675181652 3.80661884500607e-45 4.64207821539718e-44 91.7315403567587

CCDC13.AS1 -2.12382085815897 -1.9254959261428 -15.3460833625278 3.92601619340629e-45 4.7850101435866e-44 91.6390873847895

KLHDC7B.DT 2.50266647895787 -1.51906258280314 15.334349564941 4.47165427349128e-45 5.44699335778178e-44 91.5870288518816

MDS2 2.0035674379948 -2.32619978946001 15.3311323974252 4.63404798563191e-45 5.64166298774452e-44 91.5547555223965

MCCC1 -1.10219949418288 5.05022838534517 -15.3300953865064 4.68763691398932e-45 5.70372658582453e-44 90.8438275100605

ZDHHC3 -1.08167684528882 5.57799580386836 -15.3299637467821 4.69448363848522e-45 5.70887873966708e-44 90.8359273063597

IGF2BP2 -2.45245672626195 1.44908743339637 -15.3277384060595 4.8117472792086e-45 5.84822654574781e-44 91.0703973970649

SH3GL1 1.05477621639259 6.34504973596804 15.3235202054331 5.04210089575346e-45 6.12139060416114e-44 90.7774936940226

BIRC5 2.87822045228274 2.17163000719834 15.3230392202265 5.06905617427317e-45 6.15069875493568e-44 91.3707842192521

EVPL -1.62372704407981 3.859884916764 -15.3223152490436 5.10990006331238e-45 6.19681720774503e-44 90.8003740916985

LIX1 -3.79992846943728 0.19551698105128 -15.3173320139609 5.40008370476969e-45 6.54509313368452e-44 91.0069091554711

AGTR2 -3.42852079447487 -5.31652910167659 -15.3160944145783 5.47466401902873e-45 6.6318089139598e-44 91.3823398104774

CASC11 2.0652804901934 -3.54681796985406 15.3127190860525 5.68333162460303e-45 6.88076709099447e-44 91.35460333279

GZMH 3.1855065928466 2.01037501166196 15.31010870239 5.85013335102114e-45 7.0787909257954e-44 91.2496323497811

CYP4F2 -4.75990294767064 -2.14617388720491 -15.3048099097608 6.20388806602108e-45 7.50268765394348e-44 91.022608955664

SH2B3 1.32459261654799 6.32070018227337 15.3011321644533 6.46188598205633e-45 7.81037579457284e-44 90.5412188046132

CPA1 -2.88668739784906 -4.30408304856466 -15.2993199286012 6.59292475637061e-45 7.96435515207114e-44 91.1880171729499

RNF166 1.25931790734962 4.18392701412203 15.2988437673204 6.62779244116984e-45 8.00205238767318e-44 90.7558330230443

NDUFB4P11 -3.24156225277357 -4.07854137786296 -15.2968907019042 6.77274373487149e-45 8.17254383424333e-44 91.1462004428682

TSPAN33 -1.79463647745255 6.00757162614064 -15.2966425461942 6.79138616201394e-45 8.19051665554585e-44 90.4775123933795

TRDN -3.17773345840681 -4.49043534826935 -15.2943666052117 6.96476896920557e-45 8.39498600573907e-44 91.1324407091737

CFAP221 -1.69777639486359 2.90341925533538 -15.2933746330377 7.04171301600413e-45 8.48305151812229e-44 90.5686496174435

SLC26A4 -2.37898054744094 0.332342064422646 -15.291219027247 7.21185192790641e-45 8.68322866008478e-44 90.8087581825363

CCM2 1.07739759548165 5.50011506375658 15.2816064641436 8.0218691684093e-45 9.64256913846251e-44 90.3695113687506

DEF6 2.4081143528773 3.27282772057472 15.2800716108376 8.15935496860212e-45 9.80244002907434e-44 90.7839242467463

CAPN11 3.02536239865956 0.0832998660096925 15.2778497877143 8.36255069262624e-45 1.00410340816462e-43 90.9531327199797

CASP5 2.48772189722602 -1.98064170882488 15.269186484374 9.20422173820224e-45 1.10455715345927e-43 90.8718607471277

LOC100289473 2.67167160339052 -2.2624614395862 15.2666068029203 9.47080183434467e-45 1.13592443735419e-43 90.8449722503142

TMEM245 -1.23859664499722 6.91262995406797 -15.2643459263916 9.71076146969993e-45 1.16406621172437e-43 90.1280045681014

ISM2 -3.55292003546952 -2.02725776170282 -15.2631677514534 9.83820155523524e-45 1.1786963738298e-43 90.6596691311544

CD99 1.25149206624861 7.16246549065452 15.2620071824457 9.96536804613441e-45 1.19262424924521e-43 90.0850888087637

PLK1 2.38107101234848 1.80379609424865 15.2497451565804 1.14133367647359e-44 1.36367221607312e-43 90.5612712488996

ZMYND12 -1.537422331816 1.70530268838905 -15.2428070602714 1.23237016959486e-44 1.47038291291755e-43 90.1920030269559

FMNL3 1.26845133428244 6.35260332571318 15.2427854761341 1.23266439228708e-44 1.47038291291755e-43 89.8938121403683

DEFB1 -3.7870328356521 5.21025740731496 -15.2381788375829 1.29708995741259e-44 1.54554562918961e-43 89.8587121452578

ARHGAP15 1.86893958614876 3.59791110167426 15.2377418070084 1.30337372398451e-44 1.55218670246504e-43 90.233583920181

NCF1C 2.40626200912738 0.611272507303727 15.2294353213426 1.42875030187225e-44 1.70057082498988e-43 90.3980213080791

CLEC1B 2.34929874791118 -3.02638329162452 15.2269737607021 1.46816481883989e-44 1.74558246932035e-43 90.4106976543187

LDLRAD3 1.52168881911771 4.13782608218307 15.2256038708952 1.49056728235206e-44 1.77125431328111e-43 89.9881356504017

CTSS 1.93450780761684 6.85050315401828 15.2236172985793 1.52366172172809e-44 1.80959671765891e-43 89.6871961259197

GRB10 1.59110216217627 7.13517964121165 15.220230698364 1.58177757034654e-44 1.87759832942873e-43 89.6294083650982

OLFML2A 2.52989854969243 6.65721494610673 15.2195938595862 1.59295050944279e-44 1.88983428245675e-43 89.694340583173

BMP6 -1.86949928047353 2.93351104272527 -15.2146637926764 1.68215127951084e-44 1.99457688069183e-43 89.6835025901372

CKMT2 -2.81633030284478 1.39313287986836 -15.2123513086966 1.72569121180724e-44 2.04509382058696e-43 89.7740770303264

IL1RL1 -2.81104472547153 2.13941205536382 -15.2109340017546 1.75293026575376e-44 2.07624851477056e-43 89.6645674002591

SP2.DT -1.19125898501112 1.20737316816566 -15.2096391027911 1.77819146729802e-44 2.10502806797745e-43 89.9299442018025

RBM47 -1.26621751429965 6.99066316659341 -15.2056477400798 1.858363091645e-44 2.19874437692031e-43 89.4831722061017

PCSK2 -3.18899205435294 -4.32212607727859 -15.1971305663908 2.04169930219824e-44 2.414353617475e-43 90.057303241566

SYPL2 -1.59207839636494 2.97591239804662 -15.1919366310843 2.162244463418e-44 2.55275679411527e-43 89.4451992497246

LINC01615 3.21238523909438 -1.74325321350095 15.1899064620841 2.21126911928263e-44 2.60922592136519e-43 90.0003963008653

SLC2A1 1.92901382719421 7.6734574045984 15.1858782107912 2.31184989713291e-44 2.72643582310013e-43 89.2485227142627

AICDA 3.5952818245365 -1.71238105216558 15.17881345228 2.49939106277993e-44 2.94601903424648e-43 89.878876133097

DNAJC5B 3.3857878611187 -0.622606095336344 15.170335967231 2.74456470444612e-44 3.23325997230518e-43 89.778857753496

TNFRSF1B 1.46222260454931 6.02826454799321 15.1689217775079 2.7877371723952e-44 3.28235023859657e-43 89.1104816325925

FANCI 1.38284077585282 3.45555636408566 15.166254984677 2.87100052834582e-44 3.37674782270944e-43 89.4182901314712

NRP2 1.91013759077841 5.90041487809237 15.1635478476007 2.95806061165591e-44 3.47727264908643e-43 89.0984327203347

ABCC3 2.50773160573973 6.93367322982701 15.1608016887849 3.04906604290223e-44 3.58232474384636e-43 89.0216355839708

XKR4 -3.21435303962916 -2.45753613553723 -15.1593473748262 3.09838633148427e-44 3.63822541470602e-43 89.5604431654641

MAP9 -1.59341825063196 3.67799945681575 -15.1573127438898 3.16872503602955e-44 3.71691616813493e-43 88.9944801285285

ASPM 3.03283730328519 1.89927381268539 15.1566260737949 3.19282137761793e-44 3.74317197237579e-43 89.5605907928601

TNNI2 2.22693747461386 -1.14123324236421 15.1542062325896 3.27920352160761e-44 3.84238255001025e-43 89.6027591062839

BPHL -1.28573995606354 4.3485570903478 -15.1516140882847 3.37432367901138e-44 3.95172000843707e-43 88.8977331981466

SCIN -3.27544036149936 5.0100412096478 -15.1510123617295 3.39679501281356e-44 3.97590580691027e-43 88.8883312697238

ARHGEF26.AS1 -2.8484153380609 -3.8616314340037 -15.1455235575183 3.60878811044224e-44 4.22177979536908e-43 89.4864722114161

BBC3 1.73443516603317 3.04015969010729 15.1419449257225 3.75405993413484e-44 4.3893778352407e-43 89.2372553730062

PEPD -1.59437894592593 6.62295048119446 -15.1402499023932 3.82488932807621e-44 4.46980248590639e-43 88.761710385604

CATSPER1 2.02182473295274 -1.20571102676188 15.1397789996478 3.84480229227593e-44 4.49067153891533e-43 89.4444096598109

HUNK -1.98325801881095 3.26425309666625 -15.1375224769098 3.94166762937785e-44 4.60134950346122e-43 88.794146893089

TRPM6 -2.20270991759118 0.0813993412781655 -15.1343019181242 4.08414649273092e-44 4.76512831316865e-43 89.1324743952666

POLE2 1.35556005041937 0.759014196045279 15.1282579122124 4.36554517688836e-44 5.08801380002887e-43 89.2426164499854

BIN2 2.03482934483878 3.68139880814211 15.1214394216386 4.7062777993483e-44 5.47929082307717e-43 88.9624068652172

ECRG4 -2.54385420902453 1.20751893172028 -15.1210278458343 4.72767377136265e-44 5.5012702303295e-43 88.8097710374614

GSG1L -2.60767067069342 -4.93406126510029 -15.1186562835436 4.85286424980801e-44 5.6433874686668e-43 89.2159524780892

ABLIM3 2.65501187139055 6.352643472143 15.1186168955287 4.85497114405052e-44 5.6433874686668e-43 88.6196662513827

RNU6.339P 3.1410780038783 -2.43292475035164 15.1172691227563 4.92761683807468e-44 5.7247852611614e-43 89.2064102285177

LINC01480 2.47717221633752 -0.703369033671051 15.1147957962191 5.06376228218743e-44 5.87982981682475e-43 89.1678888440096

LRRC43 -2.2127363502523 -0.304519324461538 -15.113534867697 5.13460799622361e-44 5.95892663523498e-43 88.9560365640177

KCNMA1.AS1 3.01544056426486 -2.90432881600873 15.1129051784356 5.17035653783506e-44 5.99722937480412e-43 89.1592706077615

PLG -5.06667995506426 0.532397306249437 -15.1075067741538 5.48719938266384e-44 6.35799406730398e-43 88.5704929831405

ZNF582.DT -1.61137851888622 0.482902662604604 -15.1044881520099 5.67273426268183e-44 6.56600963148231e-43 88.8225217199488

GABRA4 -3.37360322794472 -4.74698178746267 -15.1028997843625 5.77285962900207e-44 6.67836429182543e-43 89.0307216519888

PTP4A3 1.83153189609684 5.43747980574075 15.1018438610306 5.84039486040496e-44 6.75291793039311e-43 88.472117611107

SAMD13 -1.71832768515561 -0.7190529240319 -15.1001211427407 5.95227281514015e-44 6.8786365853653e-43 88.877623164212

INHBB 4.11323017132783 5.28264172159086 15.0994352399926 5.99740981565556e-44 6.9271351322157e-43 88.7391028123678

BUB1 2.66857061096355 1.87526154449868 15.0986740939673 6.04789802309109e-44 6.98175993125249e-43 88.9114546069328

LINC01833 -3.43906652278209 -4.26540609592219 -15.0975893196472 6.12058649564995e-44 7.06194174706644e-43 88.956729289788

VPS13D -1.07640932498202 6.02621615278356 -15.0889195747605 6.73372402324831e-44 7.76528079578076e-43 88.1817118269206

ARHGAP9 2.63642923434617 3.23857533455009 15.0881228019214 6.793057275902e-44 7.82957176425561e-43 88.6934112522219

RNA5SP18 2.84526138313172 -3.25616294212102 15.0832042860994 7.17104381102233e-44 8.26087614139541e-43 88.8342682746252

SAMHD1 1.55032675484839 6.56758707728722 15.0829288249393 7.19282258954148e-44 8.28159915960221e-43 88.1367445000784

SHC3 -1.67981373304931 0.690384745061563 -15.0789476337756 7.5150543718466e-44 8.64805072079851e-43 88.5093288455281

TIMP1 2.06985534814865 8.05866611156005 15.0781777890974 7.57900591141233e-44 8.71705348326808e-43 88.0637254839423

ARHGEF6 1.21277775126276 5.36516988057787 15.0779447505889 7.59847138742059e-44 8.73484456755929e-43 88.1511747113547

SDHD -1.01004352925616 6.32293398633518 -15.075309689178 7.82207543929854e-44 8.98716164957892e-43 88.0344839984757

BLNK -1.36022403443035 3.78630999354176 -15.0678597214835 8.49039769936007e-44 9.74990335912325e-43 88.0146973022048

SERPINB9 1.44937780560226 4.85277976643264 15.0673199385089 8.54097681285654e-44 9.8028343640417e-43 88.1241205523488

IQGAP3 2.76823976264062 2.49957021835646 15.0643998872225 8.819848792972e-44 1.01175934736387e-42 88.4997148667752

PFKFB4 2.48664454863053 4.36564823254183 15.0633153425476 8.92572245399483e-44 1.02336732836909e-42 88.2898905712954

RNF175 2.27913442238831 0.160961867814594 15.0598449985722 9.27308996395437e-44 1.06263678543416e-42 88.5502827057731

HPSE2 -2.83527124371867 -1.36450737405082 -15.058633870084 9.39746617399316e-44 1.07632509591338e-42 88.407943926153

MARCHF3 1.46156435738467 1.74261657356387 15.0553775214113 9.74018714368567e-44 1.11440999817258e-42 88.3831162916927

LIG1 1.03200615350815 4.6045636331971 15.055159000054 9.76362718641915e-44 1.11650729934494e-42 87.9832735082491

CARD16 1.86677441767761 2.93380703564844 15.0468961201426 1.06924785061345e-43 1.22208542256568e-42 88.2156643334207

NCMAP -2.91108347928078 -0.815999738366662 -15.0423064364888 1.12459611791017e-43 1.28333153862616e-42 88.1740968137209

APLN 2.59661072038368 6.04879035407132 15.0412165839811 1.13815331996487e-43 1.29812445204553e-42 87.8054197378704

NYAP1 -2.10876680037758 -1.82181386640219 -15.0385011541848 1.17264504281548e-43 1.33676641213597e-42 88.2563479349963

CNIH2 1.89354201493551 -1.38678294606634 15.0373870855934 1.18709586689549e-43 1.35182938922705e-42 88.3246409853197

PLAU -2.06625050280086 5.28645667475812 -15.0355035412062 1.21193281412593e-43 1.3793941555778e-42 87.6023928069492

TKFC -1.02305524177534 5.43057727867303 -15.0333456294779 1.24102522786433e-43 1.41177117670584e-42 87.5724086195476

KCNE3 2.09553855042361 5.96992062603548 15.0312729747265 1.26962372151125e-43 1.4435529233187e-42 87.6555516964809

ARNT2 -2.19876891218219 4.83083293319946 -15.0291117989089 1.30014376313819e-43 1.47748526551528e-42 87.5351002031805

APOBEC3D 1.96865308138159 2.39713822695725 15.0263299618868 1.34050817782809e-43 1.52256368035745e-42 88.0444288961501

DPT -2.80997021113211 0.70324375079798 -15.0250209962156 1.3599312707017e-43 1.54382223681268e-42 87.815232741319

FOXM1 2.52174981387054 2.68933982417043 15.0237878201339 1.37848643750645e-43 1.56407394178757e-42 88.026287943113

SPC24 2.24650616032685 1.28920239110178 15.0157117883847 1.50640361142102e-43 1.70832579763277e-42 88.0195109876014

SYN2 -2.49404317578255 -0.98225075207818 -15.0134376240026 1.54451450822899e-43 1.75063669856474e-42 87.8976357065972

B3GALT2 -1.92844511621929 -0.908961133844846 -15.0065560717684 1.66579371950364e-43 1.88614456747736e-42 87.8525965623239

DEPDC1 2.66128366140809 0.622059322329995 15.0033072352106 1.72630615043142e-43 1.95364931669487e-42 87.9259203033992

STAB1 1.75223807187001 6.79623970725008 15.0027957029526 1.73603163100805e-43 1.96363867662624e-42 87.2563938991533

EBI3 2.41611802509131 1.97402197358896 15.0027152357756 1.7375664749038e-43 1.96435800186615e-42 87.8433172595139

CYP26B1 -1.83098524745183 2.5762634932991 -14.9914837522874 1.96563023718105e-43 2.21989238103966e-42 87.2749114017346

IQCH -1.35031336709558 1.13311203506688 -14.9882561985508 2.03652600727215e-43 2.29877080769206e-42 87.4971360540736

LINC01275 2.44608816538824 -2.18800713438306 14.9811764290666 2.2010972878731e-43 2.48325136974139e-42 87.7170778506118

GAS2L3 3.32261083046582 4.65341174598333 14.9729787186465 2.40828996432083e-43 2.71560168164619e-42 87.3425590977453

NNT -1.58067488880317 6.45709391520072 -14.9672838448951 2.56356510595708e-43 2.88920001343374e-42 86.8620961082852

C1orf162 2.48041030048831 4.45257992686293 14.9641198616678 2.65410710220576e-43 2.9897011600259e-42 87.1891619876595

LHFPL2 1.98365146501592 5.39372282426988 14.9630346487846 2.68589090094128e-43 3.02394507255382e-42 86.9775649528074

TDGF1P3 -3.12268488447684 -5.59349310160695 -14.95398921824 2.96601924062945e-43 3.33761166145599e-42 87.4175376588794

DNMBP.AS1 -1.74599285361265 -0.978224114955166 -14.9502335362521 3.09072054731888e-43 3.47533342263276e-42 87.2553095914901

TSPAN32 1.87471030334219 0.648682566567942 14.9502079312203 3.09158841971266e-43 3.47533342263276e-42 87.3213843756563

POU5F1P3 2.29316018103339 -2.30384101143528 14.9499311562405 3.10098512347603e-43 3.48410426238157e-42 87.3765742384482

KCNK10 -2.65061044666722 -1.19938726991294 -14.9471947902245 3.19543323626883e-43 3.58837628531257e-42 87.1792859083939

RGL4 1.90180261173044 0.222775115911662 14.9419340641087 3.38514532445847e-43 3.79751441352109e-42 87.2502378082754

NCF1 2.48357325791733 1.40785845325606 14.9414956403025 3.401453810551e-43 3.81385172508831e-42 87.2102273323889

BORCS8.MEF2B 1.80932882631591 -1.57812399202898 14.9333620073925 3.71862494842297e-43 4.16306921095733e-42 87.1907424722985

MEF2B 1.80932882631591 -1.57812399202898 14.9333620073925 3.71862494842297e-43 4.16306921095733e-42 87.1907424722985

FHOD1 1.25324752601331 4.98412109148636 14.9330354034663 3.7319591622848e-43 4.1758578378602e-42 86.6175829209732

S1PR5 2.46897420441737 1.46847644099162 14.9311812353775 3.80856778587184e-43 4.25939773923528e-42 87.0942950170059

INO80E 1.18774529869375 5.33558046426068 14.9300010968416 3.85814222441368e-43 4.3126333519239e-42 86.5344060992687

GOLGA7B 3.46889244947212 1.08961105641544 14.9292003723617 3.89214441286684e-43 4.34841676147132e-42 87.1216447079007

MIR210HG 3.23067346229163 3.97134944928194 14.9269333293817 3.99004216329323e-43 4.4555131014025e-42 86.9112976414612

CYRIA 1.71476899622797 3.99166638145903 14.9249488686889 4.07775046222253e-43 4.55112772476756e-42 86.7344087484318

LYNX1 -1.47793409431167 3.34958048846067 -14.9234012480002 4.14748367636082e-43 4.62659319956677e-42 86.4692525925273

DHRS4.AS1 -1.02931442231804 4.5941938015523 -14.9226678252126 4.18094455691564e-43 4.65916274361436e-42 86.3835844091816

GZMM 2.41668708606288 0.595397903340929 14.9216392545771 4.22832492533194e-43 4.70956088650759e-42 87.0299322960082

SLC39A4 -1.84692959787807 3.37371205047941 -14.9206934365477 4.27236575921863e-43 4.7561899610904e-42 86.4173224424978

AGR3 -4.08905792687873 -4.17097273764482 -14.9154621280482 4.52434734052664e-43 5.03414269004729e-42 86.9432745788737

LINC02685 3.78160171390831 -2.99158338240224 14.9099442674329 4.80620632460275e-43 5.34503953239409e-42 86.9423489017584

SPARC 1.74525823120509 11.042682767923 14.909452950328 4.83213673630455e-43 5.37114364692082e-42 86.281299913373

SEMA4A -1.4771008784883 4.13317473900813 -14.9064138439961 4.99566202528421e-43 5.55008653983405e-42 86.2193754921998

EMP3 1.67203315767232 5.6169009526645 14.905921469515 5.02266983761628e-43 5.57442376645143e-42 86.2868263856452

HMGB1P43 3.72130525883318 -1.09546425569812 14.9021778050124 5.23283693637885e-43 5.80473023201457e-42 86.8520807539646

LTB4R2 2.05134607356188 0.732207612891403 14.8992381046743 5.40399811163919e-43 5.99155610013451e-42 86.7677101025254

VSIR 1.23553513302136 6.16023412863258 14.8959309920723 5.6032330651671e-43 6.20930284853431e-42 86.0972396014862

ERC2 -2.55643824309344 -1.78025850678535 -14.8915675270372 5.87735782349168e-43 6.50647925616837e-42 86.6268914394452

RHOH 2.73307976824727 2.0944251712993 14.8911443202194 5.90464599155691e-43 6.53337867612623e-42 86.6330746308771

RHOG 1.01096138662094 5.57086706565995 14.8853073728654 6.29415460863567e-43 6.96083809020826e-42 86.0100015632284

LPAR5 2.37158715074401 2.37174626293549 14.8851777722367 6.30308776109661e-43 6.96719154492889e-42 86.5276924470408

BIK -2.08125894893567 -1.24186973373714 -14.8833902221734 6.42759817227732e-43 7.10122865817878e-42 86.5264309022193

DAAM1 -1.0073756484371 4.07091745370307 -14.8795829460635 6.70102626632891e-43 7.39583469687301e-42 85.9475942447529

UNC13D 2.20623503937592 3.40000600998767 14.8745035920033 7.08395341477678e-43 7.81451963519015e-42 86.3092889349386

RALGDS 1.11194627560326 5.75004680553028 14.8709183107708 7.367307159571e-43 8.11889880777131e-42 85.8447700929976

MTDHP3 -4.34840498071298 -4.45927792116387 -14.8662975608375 7.74922798111703e-43 8.53547777577371e-42 86.4109427751272

OLFML2B 2.73806628282311 5.38071752993224 14.8636089708883 7.98046960899822e-43 8.78575326778026e-42 85.9903612561618

PPP1R1C -3.29567890004453 -2.70274925235414 -14.8618970370571 8.13128307326951e-43 8.94727739175019e-42 86.3205496704619

NPNT -2.29889303272164 5.49259354241201 -14.8618449289889 8.13591785272889e-43 8.94787180853973e-42 85.7088454724448

EWSAT1 -2.41406269002538 -1.34710317980965 -14.8602607387223 8.27808899802366e-43 9.09879802043179e-42 86.2667709855307

CACFD1 -1.05716308039815 4.77602264478497 -14.8602233308606 8.28147589010151e-43 9.09879802043179e-42 85.6948013224524

SDS 4.28467263174249 2.6683118865448 14.856546453713 8.62121158119069e-43 9.46730335094272e-42 86.2924762528325

TFAP2C -3.55132235860247 -1.990589857071 -14.8561483118729 8.65882296749123e-43 9.50383015110929e-42 86.2030374415419

PLEKHG2 1.6967319410224 5.41654307835138 14.8489594556577 9.36679050440456e-43 1.02654199043507e-41 85.6931136414183

OSTM1.AS1 6.92476499213672 -0.196706494707082 14.8464132716732 9.63114708697375e-43 1.05498474832901e-41 86.2411338450815

PARP15 2.62582435122594 0.897256187589007 14.8428686894595 1.00115939748635e-42 1.09610903373092e-41 86.1665979655907

PLXDC1 2.74981161455964 5.05280353195428 14.8386255775636 1.04867705209483e-42 1.14755831844909e-41 85.7684313563905

TMCC1.DT 1.66040875926733 1.31906520793554 14.8340574844591 1.10235067638205e-42 1.20568915570455e-41 86.0089098397776

WAS 2.32787336451809 3.69668343235488 14.8325846823782 1.12023295219207e-42 1.22463485263899e-41 85.8302156596062

OAS1 1.52548214997778 5.65930368867965 14.8300168362821 1.15210544970354e-42 1.2582189101635e-41 85.4387666080147

HDAC11 -1.16263835388921 4.41929781260355 -14.8187856295214 1.30247854605545e-42 1.42173145189559e-41 85.2571237105445

LINC01879 -1.99550747404941 -5.87963573625184 -14.8154887163439 1.35022731244327e-42 1.47311619864317e-41 85.9153752008089

CECR2 -2.41105161488575 -0.724832826702501 -14.8148694110308 1.35938945743755e-42 1.48237214637638e-41 85.7176330461423

RAD54L 2.46032810978643 0.781482917555752 14.8116458980074 1.40808948639687e-42 1.53471219681949e-41 85.8280529007898

CRTAM 3.14257290731569 0.576949017238663 14.8098224955541 1.4364033666039e-42 1.56479176323006e-41 85.8295822054019

KIT -2.12888704689736 3.84868633401986 -14.8085252577803 1.4568918793655e-42 1.58632078922642e-41 85.1539337683078

RAD51 1.65167527778978 0.866302932425547 14.8047244829925 1.51861412259125e-42 1.65270290941168e-41 85.7185463835892

ATP6V0E2 -1.51100264504281 4.6789188903676 -14.8028197119136 1.55052036608513e-42 1.6865864390273e-41 85.0670515386973

PRR36 -2.59653030291603 -0.168999031676258 -14.8024828985908 1.55623137496109e-42 1.69195642970273e-41 85.5184521905156

TSPYL1 -1.14773604868263 6.90906397194271 -14.7991310757231 1.61422048451197e-42 1.75413029577524e-41 85.0293499405497

PXDC1 1.24956532564245 5.77176257212933 14.7948914051182 1.69066611632508e-42 1.83537638549687e-41 85.0235191636144

LOC102724163 1.63406110799141 -1.41556527651249 14.7936878658517 1.71301768351931e-42 1.85871774766373e-41 85.6704004637132

PROC -2.59783273769484 0.480005392035325 -14.7795892228316 1.99785697518202e-42 2.16348704056752e-41 85.1717774372335

NKAIN2 -2.75836760363455 -3.520684528385 -14.7778197400972 2.03679130043778e-42 2.20455672553079e-41 85.4671568547642

LINC02285 1.95520465160004 -2.07898882798936 14.7745389972566 2.11099093136931e-42 2.28373687243631e-41 85.4683778880237

DLG1.AS1 -2.62613696845729 -3.83611848506652 -14.7721020609322 2.16784552142471e-42 2.34408353189976e-41 85.4201831589946

GPRC6A -3.962791187743 -5.46782425338219 -14.7682399043748 2.26109448929471e-42 2.44370414809878e-41 85.3883220858289

SCD5 -2.62231719413107 4.51866453983914 -14.7669363642781 2.29346180533786e-42 2.47746024874188e-41 84.681626859315

FCGR1A 3.13425077753181 2.29849103528686 14.7636019905857 2.37837320839646e-42 2.56791451201027e-41 85.2549756435093

GAS5 1.51750593198586 6.96040526487589 14.7538053371095 2.64641727544366e-42 2.85309110608142e-41 84.5284763427979

ALOX15B 4.16522550627236 0.533749930967476 14.7524252788147 2.68652268492443e-42 2.8949004059987e-41 85.2147908656281

PARD6A -1.40790454508566 1.83053906934951 -14.7509577878049 2.72983397332246e-42 2.94012133164198e-41 84.8115942815391

FBXO21 -1.09171344398145 6.15446627916479 -14.7505169424205 2.74298049517972e-42 2.95282525917056e-41 84.4879461510738

LINC01224 -3.11173313535935 -3.51450514652555 -14.748411234288 2.80665134547821e-42 3.0198794609914e-41 85.1371623209127

CENPH 1.41590043063044 1.86995275746665 14.740305301142 3.06581196359516e-42 3.29548395673611e-41 84.9395839779576

SNX20 2.76503733028407 2.68272648573591 14.7392295821252 3.10195186264693e-42 3.33269193974549e-41 84.9425217407818

TLCD1 -1.64329341693067 1.50042614658906 -14.7375830483723 3.15809326146749e-42 3.39134211512771e-41 84.690338421521

CA9 9.05675622965958 6.1014883013322 14.7359574657255 3.21451407335676e-42 3.45023457981657e-41 85.0064022627718

NCR3 2.19874851332901 -0.806823122260558 14.7232545135781 3.69145441361191e-42 3.96020389301233e-41 84.9037764046563

PKNOX2 -2.03186581083865 0.313767784926848 -14.7221549105502 3.73591736963056e-42 4.00593730512938e-41 84.6338751040535

CLEC12A 2.97217701981886 1.24752436494763 14.7201055845213 3.82021267057884e-42 4.09061303339879e-41 84.8309805996607

PRF1 2.92239790430192 4.02900548695757 14.7200986485021 3.82050116742183e-42 4.09061303339879e-41 84.6237247671886

SLITRK3 -2.52795198883765 -5.5158130346712 -14.7114250624442 4.19881388150195e-42 4.49347109463575e-41 84.7852382720587

SLC34A1 -4.46658376573442 0.467188780505877 -14.7081793102008 4.34979459603196e-42 4.6527685417076e-41 84.2381957970883

NUF2 2.11527290838473 0.577652535689836 14.6917596787856 5.20064833246078e-42 5.56016477540438e-41 84.526205640638

ADAMTS8 -1.69579607165893 0.0271860424794186 -14.6911573505279 5.23483433476723e-42 5.59124314358105e-41 84.3591756156476

SETMAR -1.06189402942861 2.93668663487073 -14.6880857427946 5.41268380703379e-42 5.77837709990764e-41 83.9957036804704

SLPI -3.97993795172845 1.50060124406113 -14.6849872151081 5.59819610635484e-42 5.97350485899279e-41 83.933122225161

LAT 2.94391516599027 -0.88085218706619 14.6848073543759 5.60915718070329e-42 5.98227973986867e-41 84.4911819093416

LOC101928198 -1.85637540450927 5.31311135401604 -14.6833204890853 5.70059325135765e-42 6.07386954275567e-41 83.7620678561628

MFAP3L -1.85637540450927 5.31311135401604 -14.6833204890853 5.70059325135765e-42 6.07386954275567e-41 83.7620678561628

PLA2G7 3.88123902740682 2.04105294666789 14.6827150974343 5.73824670469927e-42 6.1110090271829e-41 84.4219429283472

BTG3.AS1 -1.71623733284433 -1.66476557838081 -14.679696193391 5.92974702895568e-42 6.308800478275e-41 84.3598818953249

NCKAP1L 2.5604041242184 5.12319425408689 14.6785954259784 6.00114699608137e-42 6.38165767909325e-41 83.990504901467

GIT2 1.24306892817313 5.76397591899258 14.6703932664769 6.56086637441261e-42 6.97008327078457e-41 83.6733505667776

SOX2.OT -1.93112470080877 -1.98036379518488 -14.6690833175806 6.65495695877297e-42 7.06660711467764e-41 84.2521124787471

NBPF8 2.15060503184363 2.12611337122486 14.6642222190615 7.01603046455993e-42 7.44639697630054e-41 84.1365051477988

SNX10.AS1 -1.91977312448847 0.543418343651338 -14.6594595534733 7.38872665368703e-42 7.8381477457778e-41 83.9439862459564

HIBADH -1.15802741884316 6.56090861262075 -14.6590232569333 7.42383991752027e-42 7.87157562918828e-41 83.5022089023212

CYTH4 2.17627806288348 4.23709722982649 14.658388023825 7.47526132899811e-42 7.91841424249131e-41 83.851685137971

PSTPIP1 2.80477202564143 2.2005823614146 14.6567374951487 7.6105359786475e-42 8.05780245840037e-41 84.0865460591218

UBASH3A 2.97872730442826 0.784707295216042 14.656022794006 7.66986640142267e-42 8.11495323421274e-41 84.1555885710231

LILRB4 3.39683362893761 3.75255965597747 14.6559978990853 7.67194132699562e-42 8.11495323421274e-41 83.9947401168467

ICAM3 2.26787209857926 0.899946121524646 14.6554090768436 7.72118160088777e-42 8.16308570508952e-41 84.1224711687936

DDX41 1.0190622807718 6.59584655901189 14.6542834930143 7.81618716541926e-42 8.25953279138816e-41 83.4470870903912

DOCK3 -2.08037075880145 -0.632883252901027 -14.6505282577389 8.14166140408999e-42 8.59931013357073e-41 83.9516621204642

CORO7 1.49645009296457 3.49656344755819 14.6452521925331 8.62190381844974e-42 9.10214802630831e-41 83.7425703776518

ZNF676 -3.31433290854001 -1.98968773521193 -14.636492426305 9.48240741316106e-42 1.00057483920719e-40 83.831659153733

P3H1 1.2375800186339 5.1195102596183 14.6357635507357 9.557753627981e-42 1.0080385619318e-40 83.3680889324934

PLEKHO2 1.11936947663466 5.89593543168373 14.6354314392814 9.5922827325934e-42 1.01119225545279e-40 83.2767696015093

EMILIN2 1.74593577439688 4.1166859386894 14.6290577243923 1.02795785115258e-41 1.08260062271023e-40 83.5085580943599

LILRA4 3.39707748839301 -0.651924626966499 14.6246165971739 1.07872932373816e-41 1.13552369516618e-40 83.8405288800092

AMER2 -2.7288242206593 -5.20741498931384 -14.6232473367223 1.09488146256036e-41 1.15197133371842e-40 83.8305586969943

ACLY 1.30922821352629 8.49606742390486 14.6221383949875 1.10813937159316e-41 1.1653594652274e-40 83.1030604017264

GNA15 1.9725695722159 2.40393109943002 14.6183374946509 1.1548072420521e-41 1.2138529418261e-40 83.6081750049389

C20orf204 1.95203809538535 -0.379814557044293 14.6173382510145 1.16739803531382e-41 1.22649756085158e-40 83.7504252755329

AIDAP2 2.03637793546037 -2.25249486370127 14.6151360146588 1.19563224240447e-41 1.25555749126693e-40 83.7459156286139

MMP14 1.40335872852154 7.71014255751019 14.6111216553434 1.248862231142e-41 1.31082547248541e-40 82.9742862738693

GINS2 1.93252130082956 2.1557646825224 14.6101295894878 1.26237701881226e-41 1.32437469957294e-40 83.5404127887523

KITLG -1.81077520074968 5.12163572206462 -14.6061279511797 1.31838732873506e-41 1.38247208708481e-40 82.9266035821849

BATF3 2.16420935386741 1.38943027359923 14.6034956027629 1.35657607567003e-41 1.42183486722384e-40 83.5323626179252

PATL2 2.5217328415834 0.859369639781675 14.6020221046525 1.3784322438135e-41 1.44404984774959e-40 83.5577788933723

KLF5 -1.75556180519156 3.41621769370129 -14.5949182289015 1.48883076265757e-41 1.55895633236012e-40 82.8791067271116

ZFP42 -2.39848722855578 -5.62982745146308 -14.594183756538 1.50073610119627e-41 1.5699179521035e-40 83.5201187931144

CCDC88B 2.49390214541905 3.57329049700801 14.5917070122579 1.54158667955819e-41 1.61188008174091e-40 83.2518474738098

ANXA2R 1.95401554603188 1.62483765403104 14.5915986669458 1.54339878291761e-41 1.61300304175507e-40 83.3799666828384

LMBR1L 1.3610300112945 4.74307458934392 14.5899987313413 1.57040668833856e-41 1.63966064788641e-40 82.9373658246872

BNIP3 1.47355363969383 7.61437753962222 14.5841752585645 1.67275051980024e-41 1.74401799185089e-40 82.6830156017534

CLEC2B 2.17602308103892 3.69418578966688 14.5793995235885 1.76162406339736e-41 1.83492710473892e-40 83.074791722908

TMPRSS9 2.31426220604388 -1.62398000288043 14.5633884670279 2.09537104942519e-41 2.18152184578793e-40 83.1852623651633

OGFR 1.0434452236011 5.64725082984916 14.5617578738705 2.13271236723498e-41 2.21828478634869e-40 82.4949313387205

WLS -1.6629207960864 6.05646830926538 -14.5592232410422 2.19207802832691e-41 2.27894772374062e-40 82.4231904687534

LINC01788 -3.49153805668648 -1.75685394703616 -14.5586473262128 2.20579505951557e-41 2.29211789993313e-40 82.9584933914788

HMGB3P29 1.84333191113303 -1.6460696852434 14.5538533553009 2.32334887869629e-41 2.41197829197862e-40 83.0814312182096

CD300C 2.39277636307499 1.01909377908479 14.5532604804558 2.33831424174969e-41 2.42636187677854e-40 83.0195688045748

HOMER2 -1.69592358338201 2.05710569756162 -14.5469484390905 2.50372560712946e-41 2.59553679756168e-40 82.5336124823212

RPL17P50 1.64773410243466 0.51994649895562 14.5464843089687 2.51633913470721e-41 2.60737596542232e-40 82.9444481287038

PPP4R4 -2.89822990033117 -2.93547598211789 -14.5320508114945 2.94186123187751e-41 3.04396276042704e-40 82.7818809164847

TMC8 2.57711097904621 3.70042823901965 14.5283518646255 3.06200640023791e-41 3.16528031525066e-40 82.5593841995474

GRIP1 -1.67773446947713 1.44530334927403 -14.525396933831 3.16149029718566e-41 3.26503059850654e-40 82.3979450260212

NCF4 2.10749394068703 3.0927014908638 14.52447922905 3.19303767841978e-41 3.29605349015151e-40 82.5435773762443

PRKD1 -1.1399573939269 4.3357716352226 -14.5241599421585 3.20408704309937e-41 3.30589774092778e-40 82.069286291329

BTBD16 3.60051626279647 0.718196336342707 14.5229772505594 3.24534887583991e-41 3.34689046643368e-40 82.7328710389547

CAPN15 1.27656576730663 5.4608676093423 14.5179965772568 3.42500813933519e-41 3.52884030499255e-40 82.0588991115431

VDR -1.70905419557576 3.99139407892011 -14.5173960164287 3.44733039060922e-41 3.5501654583404e-40 82.0011822326923

CD72 2.50312918433987 2.2396968796209 14.5113378041467 3.68078064043201e-41 3.78701032652357e-40 82.5007430245966

CA2 -1.83580366363202 6.64867540201145 -14.5106278015009 3.70915205827381e-41 3.81440470256271e-40 81.913136881562

PTPN22 2.300104557079 1.94032529723497 14.5072144935289 3.84861967856286e-41 3.95596828954065e-40 82.4648772513337

TTR -4.17196393175889 -3.29430909108792 -14.5040026977945 3.98462387006308e-41 4.09384040585277e-40 82.437092103108

MYB 2.67652426992913 -1.11927537570187 14.5013599744811 4.10011616107954e-41 4.21051872500334e-40 82.5152898928579

MARVELD3 -1.93748472585154 2.65949613214438 -14.4983153577957 4.23731486776247e-41 4.34732590634804e-40 81.9058172008759

IRF6 -2.43160305125003 4.05712369794869 -14.4971352885695 4.29171444147999e-41 4.40107159500996e-40 81.7719930454035

NAT2 -2.77017486963391 -2.49363796925082 -14.4955278691815 4.36693610739858e-41 4.47610951008354e-40 82.3709972266853

SLC9A5 1.74197273405992 0.555283142708886 14.4950899382756 4.3876566266049e-41 4.49523958092812e-40 82.3925990235018

RIC3 -2.83098985957987 0.630023395297982 -14.4946698590247 4.40762460091169e-41 4.51358108733473e-40 82.0580542609357

EFNA3 2.29375320312547 1.66604822799403 14.4912597743907 4.57310794327725e-41 4.68084908123831e-40 82.3143367148434

EPHA5 -3.1250495553281 -5.28703776588872 -14.4881951078053 4.72710659084833e-41 4.8339476055128e-40 82.3750551807347

CPA6 2.75390638656317 -0.810684369906941 14.4863643689542 4.82155623132927e-41 4.92822583364072e-40 82.35184244481

MARCKS 1.13271756844042 7.38654055669838 14.4855813136409 4.86252710753829e-41 4.96777956433073e-40 81.6188670072242

MIA2.AS1 -1.33257231041194 -1.0598760793223 -14.484703399748 4.90887399214419e-41 5.01278613786575e-40 82.2401837417366

GAPDH 1.17902662935354 11.8611072703676 14.482220716948 5.042337402807e-41 5.14666974607853e-40 81.6869897838616

TNFAIP3 1.44688294102391 5.88452105704134 14.4819569686795 5.05672677800515e-41 5.15894725862495e-40 81.6419921810358

DLEU2 1.24002915107573 1.47810270140882 14.472215478301 5.61787367157843e-41 5.72342160116566e-40 82.065191597405

SCCPDH -1.0512470865046 5.69839582286978 -14.4715444008183 5.65874089924667e-41 5.76237021767184e-40 81.4691766057267

GZMB 2.70362146975853 1.84972319295173 14.4701810887587 5.74267868162419e-41 5.84512143593542e-40 82.0934360809851

NAP1L5 -1.09036263905015 2.84563071029294 -14.4696159057301 5.77783934958289e-41 5.87817147609101e-40 81.6471837431995

LOC728488 2.72830733264306 -1.12450426330455 14.4608067870412 6.35444377513114e-41 6.4617803544877e-40 82.0799569890883

LY9 2.59564420044125 0.992526820244051 14.4560230354304 6.69124373833209e-41 6.80110462389633e-40 81.9827210606146

LINC02172 -2.94590785302616 -4.82427401185463 -14.4488413525693 7.23057033164208e-41 7.34586952382028e-40 81.9497254127323

ISX -2.46911057918226 -5.77922653670698 -14.4477801139333 7.31386037272199e-41 7.42358526359005e-40 81.9463693222936

LRRC37B 1.02937733183698 2.60278481257722 14.4437002726891 7.64306550233786e-41 7.754127689071e-40 81.6354928848806

APOC1 5.11923840391559 5.27833342044246 14.4403680934058 7.92287000404454e-41 8.03426812985546e-40 81.6657231367279

GJD2 -3.21852738398995 -5.43271243446853 -14.4371908879408 8.19916546911272e-41 8.31059197571986e-40 81.8281043889741

MIR210 3.04770254055067 -2.48851383798229 14.4305449227571 8.80856034743584e-41 8.91999394219256e-40 81.7635992180782

COL6A2 1.92998595215842 8.24781788422029 14.4300996525799 8.85096673232471e-41 8.95878536366335e-40 81.0247271586209

VSIG1 3.78453529233591 0.836258175221793 14.4272769283547 9.12457087550246e-41 9.23144663622015e-40 81.705081651725

ABCA1 1.50329768501235 6.52367560026267 14.4230378460931 9.55138024226352e-41 9.65878354623715e-40 80.9698014583849

PSCA -3.29480423606169 -2.9181641069166 -14.4133783699426 1.05997524722509e-40 1.07090333229819e-39 81.4869853497572

KRT40 -2.5109953187038 -5.44998479407347 -14.4122197913226 1.0732964273146e-40 1.0838607590622e-39 81.5639802048904

BRWD1.AS2 -1.57313547390074 -1.65654796350538 -14.4117938677748 1.07823548277229e-40 1.0883454967678e-39 81.4819457148691

FAM83F -3.60548716106216 0.805763902795113 -14.4091221040084 1.10973856524351e-40 1.11962681746383e-39 81.0443290217576

DLD -1.02248985747254 6.48824655579527 -14.4087003954999 1.11479414421337e-40 1.12420841871227e-39 80.7993501609329

BCL7A -1.07157770770051 4.58039706503112 -14.4063923517239 1.14287319037214e-40 1.15199298105177e-39 80.7920823632182

RGS13 2.50365292545861 -1.89807083518035 14.4043103541308 1.16880654381077e-40 1.1775901061271e-39 81.4792329435745

UCN 2.20913098595429 -0.6926172790004 14.4012001453711 1.2086440215337e-40 1.21716579735373e-39 81.4350723731183

ABCG1 1.32971588544906 5.86783606665752 14.397321907562 1.26022021936577e-40 1.26852107111009e-39 80.7275600772759

LINC01271 2.41984873910009 -3.03785147808526 14.392111262055 1.33298641403633e-40 1.33991500027304e-39 81.352587189872

PRKCA -1.364889650412 5.20011168611672 -14.3919156697475 1.3357979325484e-40 1.34212378022897e-39 80.6168535571576

LINC00410 -2.17441254536266 -5.91172707208437 -14.3891927467874 1.37555784547903e-40 1.38143683810907e-39 81.3198458956822

ST3GAL6 -1.37028580509948 3.35712622795172 -14.3843962291914 1.44848700544493e-40 1.45267492106416e-39 80.6392631409808

CGAS 1.41201011422916 2.2821620308076 14.3731746072124 1.63451821076527e-40 1.63849203944282e-39 80.9439300766218

TRPM5 -2.62859959299057 -4.21393063752228 -14.3694666649448 1.70107989661644e-40 1.70443369925534e-39 81.09311743891

SEC61G 1.04026257362797 5.53633898555665 14.3671416013661 1.74418876675475e-40 1.74602643700832e-39 80.4101897880837

DOK2 2.11779651197044 2.89402776731246 14.3603472410065 1.87650460807454e-40 1.87676225172782e-39 80.7988747788582

TPX2 2.62520670899806 3.16799069432964 14.35574879616 1.97168798506654e-40 1.97105661196976e-39 80.76394486194

SUCNR1 -2.28294258183107 3.48029771601682 -14.3454057568774 2.20373377336991e-40 2.20160933215881e-39 80.1693302280866

CIPC -1.06167089511137 4.53711876285741 -14.3453806270487 2.20432948280029e-40 2.20160933215881e-39 80.1394954506923

ZNF132 -1.1242025960289 2.14662672189718 -14.3438993252778 2.23972923843581e-40 2.23594349234983e-39 80.4009126445384

ATP6V0E2.AS1 -1.6335093535576 0.302503930891502 -14.3433747130157 2.25240170478616e-40 2.24756778331927e-39 80.5938421687573

LINC02701 -1.84579878695717 -1.34040414639184 -14.342909912237 2.26368907645775e-40 2.25676995382442e-39 80.7103025404725

SCUBE1 -1.9880473107945 1.45795466563348 -14.3422934456946 2.27874658151061e-40 2.27074551052218e-39 80.3939236562703

NLGN3 -1.29759508399775 -0.1037162250095 -14.3407199849763 2.31763334139709e-40 2.30739140817998e-39 80.6243568159434

E2F7 2.49609039903743 0.113392492117085 14.3391103094306 2.35809970894861e-40 2.34660987885492e-39 80.7595849621169

HOXA7 -1.90933652911129 1.15306022001304 -14.3375264231932 2.39860514716141e-40 2.38583151028304e-39 80.3940607564969

PHKG1 1.71315376051451 -0.202345419638647 14.3373113029237 2.40415974911889e-40 2.39026856221543e-39 80.7334910148394

PLEKHB2 -1.15382548810628 7.44483893755937 -14.3372152919094 2.4066429780869e-40 2.39164934061542e-39 80.0548329579345

UGT8 -2.08190458115424 4.71880053128654 -14.3320764334494 2.54335318122074e-40 2.52635895769167e-39 79.9828385378694

IL10RA 2.39167219338696 5.14666991084973 14.3282916805356 2.64896522362058e-40 2.62887543286924e-39 80.1917222506499

UNC5D -3.52364024720788 -0.104427892675469 -14.3281926602663 2.65178625250547e-40 2.63048048007272e-39 80.300763632162

NTN1 -1.90963881440707 1.33666380134461 -14.3209593637935 2.86616807482812e-40 2.83927338799723e-39 80.1892862890971

BICDL1 -2.31286502554545 5.01956503597413 -14.3205671267246 2.87827495409694e-40 2.84997474272227e-39 79.8588950471332

FOXD2.AS1 1.55557080066959 1.50324347514208 14.319638751315 2.90713391442881e-40 2.87594374975159e-39 80.4440482633785

SMPDL3A 1.84666637762483 6.22993166356679 14.3149521606102 3.05728155404916e-40 3.02311193668038e-39 79.8480252360237

HAPLN3 2.48350871255255 2.76344507180017 14.3130393086454 3.12076405936568e-40 3.08309480060209e-39 80.3310063366525

LINC00540 -3.00752187717946 -3.43148861580262 -14.3106122531573 3.20320632071578e-40 3.16168327581762e-39 80.4254250878354

GFI1 2.39690054543552 0.780328165119142 14.3080641064589 3.29209884717511e-40 3.24795648340035e-39 80.4018145532372

IL21R 2.65729730000273 1.78599104153128 14.3001493975891 3.58419042966561e-40 3.53453580593333e-39 80.2724704856474

SLC25A4 -1.23645640569726 5.7821606702393 -14.2998899316394 3.59419130044129e-40 3.54279939055226e-39 79.6283130003529

ATP2B1.AS1 -1.10352517667352 0.691905126834244 -14.2986093166702 3.6439603369671e-40 3.5902373870037e-39 80.1149346060858

ASIC2 -2.89997065657956 -1.66926711633053 -14.2946529269522 3.80210026856284e-40 3.74267104364431e-39 80.161054636892

CAMKK1 1.44957438980764 3.71087202071864 14.285185406184 4.20885157298739e-40 4.14119916364221e-39 79.8364343927254

CHRNA4 -4.11189308229021 -3.68659556481273 -14.2830340434604 4.30716829880822e-40 4.23602830035356e-39 80.0907944222356

SLC5A3 -2.21770655619099 6.50304102915925 -14.281942585279 4.35791964232753e-40 4.28401340277929e-39 79.4630434798334

ITM2C -1.61362278829504 7.01416304333153 -14.2810288133672 4.40086694385511e-40 4.32428710989504e-39 79.4524855281854

AANAT 2.08356698174975 -2.24812760182245 14.2782120815956 4.53592613993168e-40 4.45499298588436e-39 80.1327523647612

TGM7 -2.54534473649235 -5.75571432557966 -14.2729835609496 4.79767493537321e-40 4.70995464342815e-39 80.0769646942458

ADAM1B -1.70917525000307 -1.39814649523567 -14.2653766074096 5.20560699648549e-40 5.10813334953738e-39 79.8940591180067

DCN -4.15571996176501 5.09615420320608 -14.2628404439034 5.34915569407343e-40 5.24663821286296e-39 79.2758005791294

APOBEC3C 2.05824509212318 5.58301288406045 14.2617866962431 5.40995266950544e-40 5.30388944310015e-39 79.3765564410445

CLEC2D 2.39658149051699 3.40183079067333 14.2602344634248 5.5007670431525e-40 5.3905050311216e-39 79.7025747291493

SH2D4A -1.27379991181304 3.83431038483814 -14.2520227236862 6.00709417600438e-40 5.87877425115198e-39 79.1812078524724

CENPI 1.66847062074431 0.13992189534964 14.2509252208587 6.07819430722061e-40 5.94569293624405e-39 79.7965052943079

MEFV 2.13612021428824 0.382574340124479 14.2493951118478 6.17872231616195e-40 6.04132522483611e-39 79.7843605518531

PAGE4 -2.52681498783628 -5.92932800451182 -14.2490172679162 6.20380072827261e-40 6.06313315361992e-39 79.8221812972754

LTB4R 2.34962587472525 3.11677963795068 14.2486681729042 6.22706117258814e-40 6.08314563274782e-39 79.6028546801231

ABCA8 -2.04587074949149 2.31608003099772 -14.2457079188082 6.42783173873056e-40 6.27647037473096e-39 79.2235061198884

ZNF37CP 3.4180545097904 -1.98167898576437 14.239998186759 6.83348154220211e-40 6.66066334262902e-39 79.7262880541797

TLCD4 -1.43855486053869 3.50599715237063 -14.2365891241629 7.08774312413839e-40 6.90541464519823e-39 79.0374355141536

VWA5A -1.23578296509803 4.59846214837897 -14.2356875675368 7.1565473266328e-40 6.96934174371242e-39 78.9599615675453

CDKN2B 2.01265401658899 3.3025319031513 14.2275863466659 7.80545836409243e-40 7.59450942255173e-39 79.3334255457222

APBB1IP 2.89914258864931 5.23095699860412 14.2255710853949 7.97579097593985e-40 7.75678505550572e-39 79.1431319505016

ARHGAP45 1.48906857218541 5.20250041126126 14.2250948071045 8.01658496428541e-40 7.78952562136634e-39 78.9691475306957

GJC1 2.66500869863467 4.72140043406155 14.2194769620078 8.51374723863316e-40 8.26892970692669e-39 79.1310624146852

CD6 2.58362225137022 2.83831452119476 14.2181468083635 8.63589214756383e-40 8.38383612175532e-39 79.3173259309242

AKAP5 -1.4433272966118 1.76528884811341 -14.2166074492844 8.77942987831228e-40 8.51939969497151e-39 79.0682820895248

TET3 1.05708565082018 4.3998989630002 14.2128435138102 9.14049401744837e-40 8.86189954584291e-39 78.9014174081105

C14orf132 -2.12921272779009 2.75119453545515 -14.2123408432202 9.18982107951504e-40 8.90577206433003e-39 78.8175014690179

PTGR1 -1.38704658999319 6.18421980452919 -14.2097665504901 9.44662605074614e-40 9.15058151981185e-39 78.6709297979518

TRIP13 1.850945456344 1.94793206751248 14.2082968228347 9.59643726084974e-40 9.29157924064463e-39 79.2399704059988

PDZRN4 -2.83293373782593 -1.55086230894478 -14.2043762847896 1.00077479158073e-39 9.68553211710087e-39 79.1913151032125

SIRPD 2.4989593976048 -3.56881096463846 14.2023813436284 1.02237392397694e-39 9.89018918134927e-39 79.3285879691646

ARHGAP30 2.10141613052595 4.7604786096145 14.1986306331104 1.06424855117297e-39 1.02907184021163e-38 78.8314331548433

CDKN1C -1.64300639373614 4.34850712157831 -14.1975396144523 1.07674707007319e-39 1.04069675905836e-38 78.5566239991853

GPR12 -2.17232306564209 -5.87790024080381 -14.1966542554914 1.08699704350281e-39 1.05013909777484e-38 79.2657485001875

GNRH1 2.53699907524376 0.272885900140703 14.1965113104313 1.08866104111319e-39 1.0512819147789e-38 79.2360502744654

FANCA 1.83071774912087 2.14301068388361 14.1920399003068 1.14201372627581e-39 1.1023156342891e-38 79.0502900272923

FABP6 6.44263646877266 2.62461389664658 14.1916390798748 1.14692172577466e-39 1.10656425930921e-38 79.1836081425874

AHNAK2 3.96390190728884 5.95955602064003 14.1879286162452 1.19336590468409e-39 1.1503584082515e-38 78.7784738364894

LTA 2.58558185799974 -0.94276373684539 14.1866747192032 1.20948108168465e-39 1.16537875123698e-38 79.1501748133448

BTN2A2 1.13324613353276 4.57095792720149 14.183926057222 1.24557001938652e-39 1.19962281329457e-38 78.5792899539141

DLL4 2.28186402025449 5.95488938131657 14.1779166999288 1.32825275653334e-39 1.27869195984683e-38 78.4591634105232

MAP4K1 2.46257340480742 2.71058703459499 14.1771730375392 1.33885801857184e-39 1.28833396212463e-38 78.8852882402079

C1orf87 -2.77515242141923 -5.16571497630748 -14.1770868664352 1.3400923360118e-39 1.28895412935149e-38 79.0527548550226

GPC2 1.94552376745714 -1.43409783422075 14.1728781073431 1.40177969647099e-39 1.34710165817856e-38 79.0057217942394

AXL 1.59125649819879 6.52790126562946 14.1677014880364 1.48154734925642e-39 1.42313205377145e-38 78.2418960925092

SLC27A3 1.47227983608141 4.87794026718503 14.1673356429056 1.48735296206863e-39 1.42808103163822e-38 78.398041212695

ACY1 -1.48101920187301 2.64086466460869 -14.1655173461692 1.51654575357676e-39 1.45547098607435e-38 78.3750157328842

GPR18 2.21588809123931 -0.566383207466346 14.1569214396431 1.66247431223068e-39 1.59412247236407e-38 78.8282233787786

TMEM25 -1.44823231962279 4.0038464177332 -14.1412330084516 1.96582480776417e-39 1.88334807207674e-38 77.9791379630409

MKI67 2.92436324886221 3.75654080802748 14.1336054900762 2.13265176508318e-39 2.03870686886976e-38 78.3580121625014

GABRB2 -2.8391609446609 -1.08079860866799 -14.1305092312223 2.20433288802232e-39 2.1063089900285e-38 78.3630513115904

GNAI1 -1.17146279340441 5.45850375451478 -14.1299601794619 2.21729230634096e-39 2.11776611759043e-38 77.8154781068528

ETV4 -2.15164269462212 -0.535526784714063 -14.123449453501 2.37688017190973e-39 2.26820796492329e-38 78.2886047755326

TOX.DT -1.86082499796977 -0.411118395819119 -14.1169673084158 2.54713691377353e-39 2.42961951011318e-38 78.2310485970808

VEPH1 -1.69817091952129 4.50248181217096 -14.1087512050263 2.78050994136218e-39 2.64991206927988e-38 77.6053568688625

CCDC178 -2.00009942558768 0.432801059964404 -14.1059474006241 2.8649321471457e-39 2.72917882352114e-38 78.0111453537773

LCP1 1.94379856608247 6.80262763043958 14.1053355340183 2.88369230607858e-39 2.74585307035883e-38 77.5808034435665

LIPG -2.00640917946391 2.79405388829011 -14.1052792088886 2.88542540702825e-39 2.74630668204653e-38 77.6791816922708

LLGL2 -1.34848064347993 5.41948308964263 -14.1020984085561 2.98500078683321e-39 2.8386084505947e-38 77.5199372106844

PPP1R3C 1.86174727698631 5.67745445062972 14.1019562414396 2.98953044209664e-39 2.84167937151535e-38 77.6406669568511

ATP8B1 -1.32449636080969 4.68832502823408 -14.1011661239906 3.01482988515149e-39 2.86448162957459e-38 77.5226230568273

ITGA9.AS1 -1.12996156668025 1.20545711319952 -14.1000390955841 3.05128688306807e-39 2.89786059346747e-38 77.9345368829688

CLMN -1.5413316437192 5.78043661639072 -14.0959252118974 3.18813125917823e-39 3.02650879265082e-38 77.4567250502628

ZBTB8B -2.13193569008413 -4.01109255752624 -14.0951739831272 3.21377269864837e-39 3.04952560936009e-38 78.1770450065258

UBE2C 3.09345298700086 1.73941398493829 14.0924513452076 3.30843804761406e-39 3.13799030618533e-38 78.0877462790916

PPIL6 -1.15194771057841 1.98283493186667 -14.0904328939174 3.38040835094113e-39 3.20486176542805e-38 77.7201426903593

CTSZ 1.17584560970485 8.04306273388851 14.0898486549066 3.40152979839752e-39 3.22348788744064e-38 77.3913497781424

CD80 2.37252596720418 -0.776046328544539 14.0890103872185 3.4320648656024e-39 3.25101488981401e-38 78.1113076837627

LYPD8 -3.09958900447928 -1.65852236623392 -14.0888637458766 3.43743448772646e-39 3.25469046188415e-38 77.9558865318826

PI16 -3.33830807876858 -1.76624233022571 -14.0868710775338 3.51123618676918e-39 3.32312881721381e-38 77.9224557214852

NRXN1 -3.10123354353109 -3.21119288168497 -14.0853826726215 3.56739013365802e-39 3.37481283942982e-38 78.0124156026214

IDH2 -1.25328941128025 7.38859040013233 -14.0805178273878 3.75724800700397e-39 3.5498115303527e-38 77.3177752362695

ITGAL 2.68969245137904 4.8045831081163 14.0801271466679 3.77292478334087e-39 3.56308233752585e-38 77.6355402286798

GPHN -1.31041868208655 4.1587984593872 -14.0787184965118 3.82999269193391e-39 3.61385277620171e-38 77.3086615488079

NEK2 2.38461605451099 0.562679603981404 14.0693242604454 4.23321134700306e-39 3.99087004167635e-38 77.872173096344

RAB15 -1.2212077372076 3.45680593975524 -14.0687291468188 4.26013503702558e-39 4.01452052454162e-38 77.2710371793383

MFSD3 -1.29670331484624 3.17523227369838 -14.0678596236266 4.29978051079787e-39 4.05013377165801e-38 77.2904351986032

LINC02076 2.49155596983802 -2.44810394509073 14.0643914382468 4.4616028099677e-39 4.20074994425783e-38 77.8629530470167

DNAI3 -1.79449861468048 -0.996668041859912 -14.0628632858569 4.53481800982271e-39 4.26784573508422e-38 77.7092585583434

OSMR 1.54391901223491 7.11192910674953 14.0535601268943 5.0071010282344e-39 4.70826931024123e-38 77.0074635436657

SLC28A2 -2.36358498690743 -1.84433646033209 -14.046846988231 5.37805659077609e-39 5.05273734644153e-38 77.5614267171221

TRIM22 1.38449686393388 6.36351581930652 14.0455656091099 5.45191580586437e-39 5.11992763668045e-38 76.9434258982486

TLR5 -1.21116847414854 2.99983477667302 -14.0451329491039 5.47708211643304e-39 5.14135203996612e-38 77.0818215467349

COL23A1 5.57315064610236 6.46352701374359 14.044989958371 5.48542481357701e-39 5.14697245389001e-38 77.3728185012451

PTPN3 -1.47388434426137 5.63088366399001 -14.040888519165 5.73018817719084e-39 5.37432627622968e-38 76.8711271280616

SLC25A30 -1.09582027260173 5.51390964017877 -14.0361040881865 6.02949716139126e-39 5.65262125559345e-38 76.8184443627848

UNC5B 1.78880122555602 6.39662686624088 14.0326106868891 6.25783600794758e-39 5.86417196748192e-38 76.8247153101957

DCLK3 2.89570758166111 -0.979890179450153 14.0313766596752 6.34053880875934e-39 5.9391253573861e-38 77.5051492735579

LINC00158 2.97693159201693 -3.04934853563255 14.0307028093547 6.38615822720442e-39 5.97929373346607e-38 77.5081335630412

TNFSF9 3.86457711560897 2.28749947616724 14.0272188934764 6.62728787288314e-39 6.20022489679475e-38 77.3975652226257

LOC283194 2.00665166921868 -0.818433577076845 14.0272116681968 6.62779726303598e-39 6.20022489679475e-38 77.45577862734

PMP2 -3.27306159549711 -5.16916016753368 -14.0254250615092 6.75496033007621e-39 6.31377878927098e-38 77.4412785085043

SLFN11 1.66468145374667 5.46602961605482 14.0224499789996 6.97213242882599e-39 6.5111970071425e-38 76.7998532434738

JUP -1.28788497001341 7.80345072587991 -14.0217281340107 7.02586552040933e-39 6.55577451825385e-38 76.7056936700987

ACBD7 -1.94277481528713 -2.81456284792913 -14.0211996368126 7.06546760486633e-39 6.5899130844705e-38 77.3631807802471

OR7E14P -1.56828689976423 1.35596923432529 -14.0206672648385 7.10558496468727e-39 6.62098415370176e-38 77.0295916207147

PLSCR3 1.45834835580902 -0.747318993740356 14.0206370287183 7.10787023501776e-39 6.62098415370176e-38 77.3785060973828

BNIPL -1.63773269828861 -0.689004301587298 -14.0199339058569 7.1612198953773e-39 6.66783716973499e-38 77.2399018257395

GCM1 -2.91879591660571 -2.4433548612836 -14.0163671460743 7.43805828735208e-39 6.92265280040481e-38 77.2496502720542

NDUFA4 -1.22412253432132 6.72037515426518 -14.0157100755025 7.49020898059795e-39 6.965256887362e-38 76.6146071011964

LINC01874 -4.0758368877009 -3.71083293320119 -14.0148200930924 7.56142707415709e-39 7.02849280525542e-38 77.2451066931654

VAV3 -1.53069281995012 6.15369482259067 -14.014114108574 7.61840099168145e-39 7.0784403431639e-38 76.5929554602266

USP2 -2.26512533831111 4.19306608499311 -14.0135247435972 7.66629108182871e-39 7.11990901025086e-38 76.6018220230209

NCS1 -1.40867521578879 4.43525690936992 -14.0093695346578 8.01255369250281e-39 7.43201765035076e-38 76.5572658271464

HLX 2.45753280863247 3.97593487865226 14.0093256490221 8.01629274359884e-39 7.43233115510672e-38 76.973866419639

HLA.DQA1 2.13248486172581 6.74303268077655 13.9959878051636 9.23715909292035e-39 8.55337447701646e-38 76.4344041250367

FOXN1 -2.79775919568681 -4.06994643416326 -13.9958219269704 9.25345462612394e-39 8.56483455928363e-38 77.1139555488958

COQ3 -1.01391723751713 2.2551380966845 -13.994189353573 9.41537180645943e-39 8.71101270476537e-38 76.6765263113209

CMTM3 1.53955895238083 5.16242325292326 13.9879387109233 1.0061836208245e-38 9.3051759059999e-38 76.4617466031087

POU2F3 -2.34452641305881 -1.52090419156519 -13.987318092045 1.01283886365007e-38 9.362761289063e-38 76.9187600352791

HS3ST2 4.40028768866187 0.841325549712089 13.9864606786123 1.02210561872727e-38 9.44442878902623e-38 77.018870252077

SPDYC -2.14566738020248 -5.4949681646917 -13.9817124397511 1.07497445277045e-38 9.92036178901717e-38 76.9879721136274

KLRC4.KLRK1 2.76695480638377 -1.32582253113608 13.97653138124 1.13577612900179e-38 1.04726226780912e-37 76.9284313045762

KLRK1 2.76695480638377 -1.32582253113608 13.97653138124 1.13577612900179e-38 1.04726226780912e-37 76.9284313045762

CAPN5 -1.13722856711758 4.80828155654064 -13.9748319649003 1.1564565757369e-38 1.06588129690336e-37 76.1819470727821

CD300LB 2.15502743120972 0.471461356590803 13.9746850869114 1.15826147735733e-38 1.06709477507124e-37 76.8679047323788

DLX6 2.37455666051293 -1.83104736576567 13.9736300643996 1.17130883117064e-38 1.07866042509785e-37 76.9005665624854

TBX19 1.49200809246342 2.01391527326315 13.9708796950428 1.2060151278003e-38 1.11015368946166e-37 76.6952749737381

WDFY3.AS2 -1.28635531134073 3.65481850132289 -13.9665412445386 1.26285599913729e-38 1.16071483617843e-37 76.1609337409515

PRELID3A 1.49722506889206 0.491592729185899 13.9665255197738 1.26306680109473e-38 1.16071483617843e-37 76.7565063016273

FAM72A 1.44926291965134 -1.66720086730904 13.9663295366924 1.26569704155418e-38 1.16264302013802e-37 76.8194190329195

CDKN2C 1.56129814639332 4.29845809497403 13.9655765107123 1.27585409747012e-38 1.17148065512666e-37 76.3558786384739

ANXA2R.AS1 1.49854249734875 1.3251421808844 13.9650092047297 1.28355971749297e-38 1.1780609200493e-37 76.688132790767

CAMK1D 1.25788237849705 4.64931523784265 13.9647639833106 1.286904858735e-38 1.18063525935919e-37 76.2573980339398

RHOBTB3 -2.06153254987493 4.99404734844616 -13.9643718592617 1.29227200804031e-38 1.18506169499391e-37 76.0664968848334

APOBEC3G 2.24256270403179 4.36208799100218 13.9590531693063 1.36731295785455e-38 1.25230050578355e-37 76.3699085941321

SLC41A2 1.17554093197178 5.06426321687334 13.9587553883915 1.37164041014588e-38 1.25573765743267e-37 76.127598160391

CD86 2.46063411753615 3.56699859870558 13.9538670490314 1.44466244523476e-38 1.32148214381395e-37 76.4349340231235

LINC02912 2.50473976072058 -4.00486098768586 13.9538130150544 1.44549088265243e-38 1.3216867053809e-37 76.6964959400858

PSG5 -3.71551543628823 -4.83363674244926 -13.9479731808302 1.53787425353882e-38 1.40556947145896e-37 76.6070275872659

FBP1 -2.30384137325931 5.08380560364123 -13.9431699595005 1.61825191985939e-38 1.4784138463498e-37 75.8445434005185

FGF14.AS2 -1.3329995732127 1.44715390755148 -13.9422780002536 1.63363256608869e-38 1.49184172447707e-37 76.2134303491068

NADSYN1 1.05994858430935 5.84706670359241 13.9388570780008 1.69398472579238e-38 1.54630944915375e-37 75.827866243127

ACKR3 2.12861908254788 5.9422012303863 13.9330201595699 1.80213369137084e-38 1.6429715293086e-37 75.8489944043757

FETUB -3.30060137824463 -5.05975057555416 -13.9316860248408 1.82780372625362e-38 1.6656795175071e-37 76.4505349148143

DPP10.AS1 -2.79254649239294 -5.74168391158784 -13.9315395489121 1.8306441706016e-38 1.66757261609657e-37 76.4577736669487

BAZ2B.AS1 -1.16471805306994 0.81366935381382 -13.9239791975529 1.98338542573499e-38 1.80595507119111e-37 76.1183425715688

HOXD10 -1.65671795675534 3.15285685812352 -13.9216409455855 2.03314828012216e-38 1.85049518390294e-37 75.718840605257

ADHFE1 -1.29818177480665 2.74985785980771 -13.9203147639406 2.06192314435776e-38 1.87590368333264e-37 75.7858692799228

LINC00607 2.05341112747336 -1.66595313831237 13.9177714320627 2.11824749959565e-38 1.92634467784701e-37 76.3099323207795

FAM110A 1.14096827000533 3.31182241952143 13.9164225001235 2.14874040920682e-38 1.95326223637257e-37 75.9413451037933

ERICH4 -2.75202851714514 -1.75341342101442 -13.91615016834 2.15494940016048e-38 1.95809186036203e-37 76.1516809120253

CDKN3 1.92064517018819 0.80500198626746 13.9113366639835 2.26769277265298e-38 2.05967955780488e-37 76.175580821935

PDIA5 1.61396106683541 5.77080725295258 13.9098023667465 2.30485054538488e-38 2.09255915946389e-37 75.5685072424345

PPARGC1A -2.49866850875707 4.91149109466945 -13.9072839441253 2.36716184036222e-38 2.14823869175397e-37 75.4684822913193

ABHD14A -1.04654434723135 3.4255154651728 -13.9067520774587 2.38053458123902e-38 2.1594778830974e-37 75.5744383378566

ITGAM 2.14947911729753 4.14525323057514 13.903348038293 2.46792341535636e-38 2.23782283799927e-37 75.7951472124139

SLC7A3 -2.24166951798226 -5.61720458024872 -13.9022931752364 2.49564738248042e-38 2.2620233201719e-37 76.1513316280836

CEACAM21 1.85367441787835 0.639186563414375 13.898479895071 2.5984823567068e-38 2.35327952511867e-37 76.0457654178229

GYG2 -3.09291017705364 -0.75661194806149 -13.8958951781602 2.67057641789279e-38 2.41756861889855e-37 75.831017103331

ANAPC1P4 2.00411405071016 -0.0838188895669663 13.8946568430666 2.70581947231309e-38 2.44845850635436e-37 76.0415452388082

S1PR4 1.92735519459027 1.56182548138445 13.8906469493785 2.82315266217972e-38 2.55146216404522e-37 75.9095646812784

HAP1 -2.49870787656876 -2.11336889834623 -13.8902353747809 2.83547897366819e-38 2.56154286943245e-37 75.9234078974536

KCNA1 -2.61186883497529 -5.03243030645265 -13.8881638088769 2.89833971430594e-38 2.61724866845982e-37 75.9985765981847

PLAAT3 1.12977599513529 6.76441881475769 13.8866649514317 2.94468599992805e-38 2.65800178258685e-37 75.2415837012108

COL4A1 1.7390885607526 9.91814094150903 13.8859319780067 2.96761848306312e-38 2.67759565278193e-37 75.2651285478521

CAVIN3 2.19502435325438 5.13031130153995 13.8807439013813 3.1351059823182e-38 2.82754729804373e-37 75.4196831711025

IL24 1.656101932146 0.332057811543681 13.8782692375845 3.21828185113884e-38 2.9001696203273e-37 75.8414850709923

LBX2 1.93408918789542 -0.336216595380657 13.8720044803712 3.4388084678901e-38 3.09634451787401e-37 75.8097613554859

PRMT8 -2.8097839194232 -3.88486029353961 -13.8707879740741 3.48334552296295e-38 3.13515443629775e-37 75.790419635522

TSPAN4 1.17435856134193 6.35700430648678 13.8703093814129 3.50102423986039e-38 3.14976874078506e-37 75.0825362459509

MUC12 3.7994965358514 -0.288607198052316 13.8692707186825 3.53969955373695e-38 3.18325326534212e-37 75.7932611411236

VSIG8 -2.23751618027451 -2.22692196639433 -13.8683943206091 3.57266381805551e-38 3.21157640542851e-37 75.7075982035858

IL18BP 1.48901272777885 4.79062048230837 13.8628718725056 3.78753494248684e-38 3.40193181661179e-37 75.1897801574249

TBX15 3.26383489923803 1.48130129333245 13.862802082997 3.79033117244363e-38 3.4030446635748e-37 75.6821900764731

SYT4 -2.58643675138486 -5.23315279765049 -13.8627016926995 3.79435708110464e-38 3.40526017631949e-37 75.73224935845

KIR2DL1 2.47082556380242 -3.00990087130031 13.8625604991451 3.80002651741914e-38 3.40894825472744e-37 75.7363108290716

DEGS1 1.16702439352855 6.84177764649194 13.8621407505568 3.81693085494688e-38 3.42270783640354e-37 74.9824551875023

BTN3A3 1.3802105198998 5.47911584785 13.8615959825385 3.83898183021427e-38 3.44106931647549e-37 75.0701121809797

OIP5 1.55359999842764 -0.372436897170504 13.8614639825656 3.844343969188e-38 3.44446284373372e-37 75.6919852950592

SOD3 -1.69828800412113 5.0127124904766 -13.857372610165 4.01429750712267e-38 3.59526407471933e-37 74.9353440404756

PRR11 2.02151872085899 2.05432694171979 13.8555374706532 4.09294098009979e-38 3.66269611949716e-37 75.5082203010675

LGR5 -3.47110671357368 -2.8925015082248 -13.8544690472578 4.13943263409817e-38 3.70278433700152e-37 75.5466237924205

KRT36 2.54117972699652 -3.82141457395515 13.8517280735132 4.26112502449489e-38 3.80852209244527e-37 75.6226790147663

SLFN12L 2.44141569054451 0.4738905055456 13.8511851960196 4.28564711097249e-38 3.82887352069018e-37 75.576074636883

PKD1P6.NPIPP1 1.61072295860171 0.0118682584715438 13.8505893331016 4.31272449524939e-38 3.85051947979801e-37 75.5636907477556

ASAP1 1.05901731693218 5.88718531820698 13.8505745290862 4.31339938980714e-38 3.85051947979801e-37 74.8940857504894

IGSF9 -2.54467194271842 -1.42408509107577 -13.8495816441512 4.35890470321223e-38 3.88955265329918e-37 75.4480334111336

HASPIN 1.75061951229675 -0.946413529914284 13.8491819914354 4.37735610179651e-38 3.9044229752065e-37 75.5799540698381

RAB37 1.94409907655152 3.30698774017421 13.8464993742674 4.50323957303658e-38 4.01342962437065e-37 75.2853230047099

SAMD14 1.60271771890582 2.71022052690053 13.8363155713286 5.01484497516797e-38 4.4657460163955e-37 75.2153265832511

FHOD3 -1.47457008688928 4.59648480595479 -13.8358069647199 5.0418625113749e-38 4.48797643425971e-37 74.7178782796556

RAPGEFL1 1.79087352064187 2.16212973219487 13.8302521245833 5.34655149053579e-38 4.75338444762729e-37 75.2218501628969

PTTG1 2.37141751240363 2.15940786468757 13.8227816202506 5.78543971522049e-38 5.1414889831929e-37 75.1803107488493

SH3BP1 1.68632113962151 3.25377999521525 13.8176148389667 6.10981002895652e-38 5.42754790905638e-37 74.9676465249956

CD96 2.90746131853976 2.86204553208044 13.8134410670504 6.38502319449173e-38 5.66972417184997e-37 75.0554143152453

NMI 1.03311778947436 4.46691127813989 13.8079298976866 6.76743460127689e-38 6.00197842295876e-37 74.6053932328222

ADAMDEC1 4.61566207582238 0.676502797466202 13.7980516724618 7.51086713615668e-38 6.65861985908446e-37 75.0394699556616

KIF20A 2.61308266326516 2.07257961609182 13.7973582780212 7.56600930196577e-38 6.7047851287858e-37 74.9308998868292

SIPA1 1.10482801738698 5.59590161929567 13.7957895334365 7.69225696496385e-38 6.81389912668647e-37 74.3448570430848

ADGRE1 2.69651490321742 1.11335793351041 13.7955443425389 7.71217796379035e-38 6.82877735181161e-37 74.9730717676154

CD53 1.80791324756571 5.57185838604728 13.7917323864981 8.028583226323e-38 7.10606031773335e-37 74.3725756509452

LAMA2 -1.96517075948093 2.73658704179467 -13.7878836748565 8.36116226497908e-38 7.39144332429562e-37 74.3338650932765

JHY -1.12824197471094 2.52101099182488 -13.7702044900187 1.00741380360056e-37 8.89136262119673e-37 74.2529384668992

XRCC2 1.66787530293263 0.501588114696944 13.7677552075382 1.03375432440445e-37 9.12015876108614e-37 74.672628008317

LOX 3.68281702243274 6.93572617752222 13.7662754063509 1.05000008891736e-37 9.25974654685676e-37 74.1297862786144

AJM1 1.96355948589021 2.43537766205192 13.7546633566383 1.1866331068124e-37 1.04562472916013e-36 74.4136653810732

PRSS53 3.11612222128655 0.633034159150927 13.7470798425275 1.2852864273338e-37 1.13209852061771e-36 74.4951934201543

ZNF331 -1.10484404591373 4.50621161949738 -13.7450469268553 1.3130941330122e-37 1.15566033381859e-36 73.7749950990725

TDRD9 -2.56308716712944 0.378313973443571 -13.7408383770113 1.37258138809619e-37 1.20704310157208e-36 74.1117106658704

POU2F2 2.19629645712788 3.00710364818187 13.7405945368075 1.3761091862168e-37 1.20965865029749e-36 74.2307677447632

PELATON 2.46201272071669 1.05740567455567 13.73764966373 1.41943483096218e-37 1.24724203301233e-36 74.3599785082401

H1.0 -1.5729313019999 6.77082859474575 -13.7361055256621 1.44269302638779e-37 1.26716924058089e-36 73.6760534788421

MFHAS1 -1.21897882045076 4.69046766006225 -13.7343830199012 1.46908616795844e-37 1.28983286574511e-36 73.6527089739508

LOC102724323 2.52813166164191 -2.50420577862419 13.7335338471011 1.48227415063934e-37 1.30088903670367e-36 74.3830007839144

FBXO17 1.52003987017329 6.55404703783864 13.7290103904862 1.55453877037358e-37 1.3632157202638e-36 73.602218220252

LINC01857 2.89591457892371 -1.9936134937253 13.7279203708067 1.57247092086943e-37 1.37838776709826e-36 74.3225785693349

SYNE2 -1.32587342887605 7.62599141519797 -13.7258668820178 1.6068150904876e-37 1.4079282346602e-36 73.5844662237495

MYOZ1 -1.73925780353179 0.225087506155599 -13.7241021252349 1.63692681036594e-37 1.43373793935579e-36 74.0313712589277

P4HA2 1.44504370782526 6.51218234351809 13.723730586191 1.64333761062354e-37 1.43877631430113e-36 73.5454853551318

MKLN1.AS -1.03479949811758 1.73037013652323 -13.7187802685494 1.73117971422498e-37 1.51507690408324e-36 73.8564530366946

HOXA11 -1.84263789371455 0.469104626606683 -13.7153111714842 1.79551113575486e-37 1.57012024208287e-36 73.9079292673942

PAQR8 -1.13237185440908 3.85508156655372 -13.7107858538025 1.88302242962316e-37 1.64467182871922e-36 73.4620529185689

CHST6 -2.58989063394957 -2.20828585244313 -13.7097308318664 1.9040280171533e-37 1.66235414537959e-36 74.0340336264927

AVPI1 -1.0078326849044 2.94144290474212 -13.7080052705936 1.93888850196942e-37 1.69211383520518e-36 73.5577156724742

PAQR4 1.78466006164962 2.3526699103271 13.7058977576184 1.98232913306094e-37 1.72864479428495e-36 73.898319800104

AKNA 1.44564773719896 5.24184381519854 13.7057234423745 1.98596529469054e-37 1.73112483386009e-36 73.4707981239152

SLC2A3 2.13943498365632 6.41496680176265 13.7040496950223 2.021219179162e-37 1.76115242114143e-36 73.3872040061836

NDRG2 -1.53288294941836 6.54030532008598 -13.7038324607995 2.02584023401164e-37 1.76447535407957e-36 73.3321390454934

LOXL3 1.51750902085554 2.62804402793772 13.7012478310182 2.08163548246905e-37 1.81234980870104e-36 73.8016420887813

TOP2A 2.64376349037463 3.82378407678627 13.7010277965899 2.08645557407079e-37 1.81582292553441e-36 73.7676896899083

LAMC2 -2.54078471789733 3.16372567814599 -13.7003459601359 2.10146267576221e-37 1.828155408178e-36 73.352852821138

CDH19 -3.68918217298103 -4.22438668213445 -13.6980767002913 2.1521876172931e-37 1.87153824117414e-36 73.9608325695593

AXIN2 -1.21457901150494 1.17371405745143 -13.6948548148184 2.22630922796391e-37 1.93522416701254e-36 73.6620655512085

OR51E1 2.90019766546476 2.3470081636523 13.6931435359224 2.26670704499344e-37 1.96955662243505e-36 73.8321231175422

SLC5A11 -2.62936965740551 -1.3580971323167 -13.693043452494 2.26909217435438e-37 1.97084544062664e-36 73.7886043944265

PBK 2.32604414118272 0.736921105211941 13.6926742259938 2.27791301361139e-37 1.97772082186927e-36 73.9013298214635

NOD2 2.40099647334199 1.60346201338993 13.6914895547218 2.30644620184544e-37 2.00169852458016e-36 73.8424056424877

DNAJC28 -1.19025334201064 0.716065143736203 -13.6907950749579 2.32333825581896e-37 2.0155581939028e-36 73.6766186494499

GLB1 -1.04545284780006 5.78614864950236 -13.6881415931655 2.38902279060248e-37 2.07171885091412e-36 73.1527529359696

PLEKHN1 2.40833068335655 0.986959400769289 13.6866078676353 2.42782923252032e-37 2.10453598644452e-36 73.8291668144998

RRM2 2.65509964527273 3.01223484989075 13.6797680950659 2.60867904071207e-37 2.26040694197783e-36 73.6265582452641

C11orf16 -2.00324122226061 -3.17514277513075 -13.6786962060797 2.63821261408106e-37 2.28509156779681e-36 73.7783646858847

CLEC7A 2.50457475816205 3.30106185625388 13.6764705062917 2.70060527726932e-37 2.338206304444e-36 73.5508748556268

TNFSF15 -1.37703579347385 2.27776800079943 -13.6756970771578 2.72262914001605e-37 2.35634117214934e-36 73.2739567514324

SEMA6A 1.96929878276826 6.34517471127915 13.6741957281719 2.76589311094872e-37 2.39283698153453e-36 73.0668613610072

KAZN -1.09249911252021 4.82955843336602 -13.6712094160741 2.85399571622371e-37 2.46710317985114e-36 72.9887226953527

GPX2 -2.14703299483974 -1.1917005500357 -13.6693067403429 2.91158137621299e-37 2.51489279898745e-36 73.5640796670473

NLRP2 -2.47191031445485 0.233471375135095 -13.6685544720538 2.93466729587155e-37 2.5338318615836e-36 73.3904874444984

ARHGAP11A 1.60819936717777 2.42365428043408 13.6683443911435 2.941146862343e-37 2.53842347483339e-36 73.4861645321426

LZTS1 2.35212156625773 4.94035859471721 13.6661060007108 3.01107733816868e-37 2.5967274297948e-36 73.2139644206692

KPNA7 2.3062691054828 -3.72008894876097 13.6651945448243 3.04002491822617e-37 2.62065737822471e-36 73.6712166386177

AOAH 2.54518884652543 3.87140602887705 13.6647009260839 3.05581768716944e-37 2.63323280432626e-36 73.368189543802

SIRPA 1.4613556103146 7.44422688582539 13.663224748033 3.10353575361525e-37 2.67329786455475e-36 72.8919388152826

LINC01270 1.67038511211069 -1.11603813059248 13.6601728201062 3.20455767837767e-37 2.75922769683165e-36 73.6040985060723

GALNT6 -1.11784898799167 2.88211998996827 -13.6509157277318 3.53145800951111e-37 3.03830519219868e-36 72.9608136562667

TAGAP 2.07382392485207 3.05711671938091 13.6481526195926 3.63532355086257e-37 3.12643548040141e-36 73.2465814449207

CENPM 2.02596757564877 1.01059724367885 13.6468447704039 3.68554156253729e-37 3.16837685940705e-36 73.3973247539328

TAP2 1.11013201613117 5.21245747496431 13.6442346948656 3.78783713787288e-37 3.25375805715158e-36 72.7961343282497

FAM230I -2.8608486591835 -4.69186534524769 -13.6413443951609 3.90441923290199e-37 3.35126761573477e-36 73.4093046257469

WT1 -3.38177826771787 0.235376871775684 -13.6361306880386 4.12383150127608e-37 3.53820533166024e-36 72.9372365494767

ANXA1 1.37277645242354 7.20322837177039 13.6359835364019 4.13019928471295e-37 3.54227806000126e-36 72.6083706326645

SDR42E1 -1.86145343975454 2.31819373594705 -13.6317973976705 4.31551037029572e-37 3.69975865524019e-36 72.7641266677017

PTPRN 4.68016306687278 0.472824876147825 13.6317470988167 4.31778662601135e-37 3.70025847600887e-36 73.304026309724

LMO7 -1.32055251346125 5.95427707564028 -13.624910855273 4.63855508880526e-37 3.97203543713406e-36 72.4951993136876

ABLIM1 -1.16027807009952 7.32604894078335 -13.6245901546505 4.65417262774353e-37 3.98384780392007e-36 72.514145548377

NTRK2 -2.6817972217772 4.5254837969579 -13.6225144126143 4.75653221740136e-37 4.06987073402005e-36 72.485303633356

GRAP2 1.86599874299427 1.49080484166762 13.6213340766213 4.81573444423954e-37 4.11891369119243e-36 73.0891820683044

HLA.DMA 1.40562253200667 6.79494273737857 13.6205781080335 4.85403673624797e-37 4.14842646840934e-36 72.4558794779041

STAC2 -3.63007217827409 0.388481156279099 -13.6139904301681 5.20092248258339e-37 4.44314929679026e-36 72.6815505950564

LACTB2.AS1 1.79541117272353 -0.64594203708161 13.6107736989557 5.37917185593306e-37 4.59363198779621e-36 73.0828777884875

ANKRD45 -1.88629802415118 -0.0424005437566285 -13.6106011000827 5.38890633428015e-37 4.60014727043063e-36 72.8658157480192

IKZF1 2.45686727177081 4.11986418043778 13.6067725410812 5.60940530799529e-37 4.78650270189852e-36 72.7232243871518

TMEM156 1.89689144624796 0.526094039763651 13.6023918267588 5.87275172463976e-37 5.0092600873752e-36 72.9536048633426

OTOF 2.23157626248493 -2.22552487779819 13.6005511766133 5.9870444136451e-37 5.1047554261173e-36 72.9945775980455

ADAMTS7P3 1.98114040431048 -2.98133914589744 13.6001176299615 6.01428578559106e-37 5.12598234292205e-36 72.9924475118576

TTC29 -2.60482933335977 -4.41589698793015 -13.5979771149634 6.150603195058e-37 5.24012209051081e-36 72.9568384231002

PFKM -1.11380182509386 5.96490213955045 -13.5966243363051 6.23833713719115e-37 5.31072775453986e-36 72.1980660141406

APOD -2.03838917999422 2.35288712256942 -13.5922238340688 6.53244666118691e-37 5.55677527780917e-36 72.332178685149

NEDD4L -1.40990655470325 6.41255083070986 -13.5913017477613 6.59580398202649e-37 5.60848655327724e-36 72.1519227666289

HYAL4 -2.95921676089016 -4.69947202999525 -13.5906691981669 6.63962068163682e-37 5.64354845413495e-36 72.8804972816404

ACSM3 -1.55691385127136 4.63083841669468 -13.5884124843535 6.79831950323293e-37 5.77619269456256e-36 72.1250989002812

RPL18A 1.04104848930898 7.80867663567543 13.5881520616483 6.8168747243717e-37 5.78970708712378e-36 72.1101440453414

TBC1D10C 2.49526828129873 2.79753852352116 13.5878154913402 6.84093025807536e-37 5.80788068880034e-36 72.6763090648417

IGDCC3 -2.31343624661524 -2.65760213786217 -13.5793459141536 7.47491657871987e-37 6.34120155259182e-36 72.7073849809698

RASL10A 1.84965466563308 -0.940228681295668 13.5732783126828 7.96477870962768e-37 6.75152479214483e-36 72.6985640252096

MYH14 -2.93177722977133 3.81069806173 -13.5583519609714 9.31026949162591e-37 7.88288722202638e-36 71.8341663393907

LINC00426 2.41180887962331 -0.272545722292857 13.5578200838565 9.36218180105473e-37 7.92377067770911e-36 72.5324213412901

SCGB2A1 -3.28078730683259 -3.41591630184485 -13.5577261381591 9.37138101723286e-37 7.92848584473828e-36 72.4812168303665

ADRA2C -1.88161295236777 2.21131497631984 -13.5576470102592 9.37913625291272e-37 7.93197618169124e-36 72.0089549096789

PTCHD3 -2.29466623404008 -5.32361719756031 -13.5574158930881 9.40182441610126e-37 7.94808777427701e-36 72.5452834966241

SLC15A3 1.56274083176473 5.10557280344885 13.5573179796882 9.41145279832198e-37 7.95315073479235e-36 71.955294270899

TM7SF2 -1.39142970944815 3.97086489359246 -13.5509472631211 1.0059501447989e-36 8.49421503643367e-36 71.7719663874804

MYL4 1.71760023143396 -0.739836128227575 13.5508762003591 1.00669753656506e-36 8.49724266765399e-36 72.4610866057486

LAMA5.AS1 2.72557910427836 -1.62472039994787 13.5446323691344 1.07457272868049e-36 9.06665553662345e-36 72.4108931383724

PRKAG2.AS2 -2.69205290226123 1.17205119680521 -13.5441478763761 1.08002630997426e-36 9.10915281816576e-36 71.928315487132

ADH4 -3.25692328437401 -4.33484497611755 -13.5398880168556 1.1291791719234e-36 9.50903755069058e-36 72.3352822428697

ATAD5 1.24914431867806 1.70256072247491 13.5394486432509 1.1343740277789e-36 9.54910463368732e-36 72.1849682553663

ADAM18 4.890477856573 -2.11722299116494 13.5379115875169 1.15273518983651e-36 9.69619788433308e-36 72.3435929529687

OGDHL -3.01716187511922 5.68092821414514 -13.5374449909595 1.15836736315484e-36 9.73982377338312e-36 71.608836062067

FAS 1.24364683842978 4.86839300801887 13.5372684722388 1.16050521816227e-36 9.75404635865391e-36 71.7390308013032

DNMT3L -2.55902062584568 -5.11495461991368 -13.5371229862867 1.16227018357892e-36 9.76512507564403e-36 72.3324022796647

PCED1B 1.62772062247999 2.8640026450634 13.5336325057598 1.20542611835284e-36 1.01238189717005e-35 72.0384858797269

ARHGEF1 1.15341488207924 6.23882257374611 13.5304987256065 1.24553005135573e-36 1.04566147569254e-35 71.52989243337

B3GNTL1 1.25278575969126 1.98667027045043 13.5289586275487 1.26572356479807e-36 1.06220649237835e-35 72.0523483304206

GLS -1.18078643408582 7.99448557546387 -13.5262707224323 1.3017514133462e-36 1.09160297911952e-35 71.5076872788924

NIPA2P1 3.26448461178607 -3.61461903511171 13.5259535694675 1.30606926247238e-36 1.09480366677441e-35 72.2221543330657

ITK 2.58791770891141 2.51884422957406 13.522193171375 1.35836546066748e-36 1.13820400352632e-35 72.02071175225

FAM107A -1.80501829371562 4.80505827393936 -13.5189590425905 1.40500815929313e-36 1.17683569586174e-35 71.397548686448

CD7 2.9359823260521 2.20458963751029 13.5187829395716 1.40759323994048e-36 1.17854923649116e-35 72.0325044908494

KIR2DL3 2.39362441433587 -2.96340432038532 13.5150640377622 1.46330466809725e-36 1.22472604028836e-35 72.1098420179843

ATP5MC3 -1.07564981863735 7.2420067794405 -13.5087621574989 1.56277078166951e-36 1.30647398208967e-35 71.3041873638966

RPL32P27 1.72733928330044 -2.94752007877365 13.5082575731843 1.57102026959194e-36 1.31286829642037e-35 72.0382443847665

DLX2 2.64863945868575 -3.09867028440222 13.5042305765748 1.63843179832329e-36 1.36867928473849e-35 71.9979059944898

EHBP1.AS1 2.06534172633301 -0.39929233429851 13.5003243570176 1.70657336940938e-36 1.42505723506699e-35 71.9341409475919

NTN4 -1.95329878304122 6.72327767220596 -13.4998608787022 1.71484357636674e-36 1.43141622132706e-35 71.2175475181222

INTS6L 1.6760288948873 3.2858485120732 13.4995513524183 1.72038892109678e-36 1.43549671984451e-35 71.637733864766

PRC1 1.39295207226158 3.40472187118094 13.4936115697351 1.83033270035515e-36 1.526651164155e-35 71.5334566783116

PLOD1 1.22713935235988 7.8354450215223 13.4924966788188 1.85173605955647e-36 1.54391408277328e-35 71.1140970339773

RPL34.DT -1.46633863738707 -1.43594252621784 -13.4904023279394 1.89261923219359e-36 1.57739924031756e-35 71.7560149342055

TRIM59 1.17928382307934 2.47915666530364 13.4889573207823 1.92134980537334e-36 1.6007341706757e-35 71.5817309216725

CKM -2.27864533495452 -2.88276424458256 -13.4806131839981 2.09595820322106e-36 1.74554019112004e-35 71.696467762953

ADA 2.09874045621751 3.09155107276 13.4803011427044 2.1027859891361e-36 1.74989269689989e-35 71.5039225193321

SVEP1 -1.94183928498838 3.53973858949844 -13.4792165655241 2.12669047344069e-36 1.76911179733915e-35 71.0373902756498

TRIM14 1.54298253766126 5.46571383689954 13.4787120622795 2.13790197870511e-36 1.77776148936997e-35 71.0791177752478

DOCK10 1.45796034319076 4.80088111107967 13.4785416243152 2.14170288874312e-36 1.78024470246114e-35 71.1630056935563

CCNB2 1.79535292766798 1.81218648463028 13.4760184063616 2.1987667651275e-36 1.82628848796394e-35 71.5535248474027

DLGAP1 -2.21837495724774 -0.43652575059523 -13.4743216108654 2.23798993916824e-36 1.85745515156261e-35 71.4615563227069

CNGA3 -2.36732080756983 -5.42953646229378 -13.4738489800316 2.24903887671825e-36 1.86450100807753e-35 71.6791921865798

PXDN 1.73153986016318 6.62920545653871 13.4703193434847 2.33328961393192e-36 1.93288013393685e-35 70.9108337730701

ST3GAL4 -1.38283403225365 4.51340707217561 -13.4692815872611 2.35865324456546e-36 1.95315079020421e-35 70.8929038892392

CBY2 -2.87738953101403 -4.93238871751752 -13.4655267927331 2.45273999306061e-36 2.02952393291759e-35 71.5869437602776

ADAMTS16.DT -3.25253928944632 -3.5339623407982 -13.4645124691994 2.47879195911357e-36 2.05030434074598e-35 71.5191833217864

DTX2P1.UPK3BP1.PMS2P11 1.50976123048293 -1.3612598370753 13.4642446444324 2.48571667556636e-36 2.05525412452333e-35 71.5711882654231

CD99P1 1.3725943698516 2.21634052124678 13.4620866010814 2.54222161643774e-36 2.10038446064325e-35 71.3447981780013

ZGRF1 1.22424407431095 1.41412921710302 13.4610363845719 2.57018095067442e-36 2.12268194690431e-35 71.3948006121536

FAM3D.AS1 -2.84775188409122 -3.8140561132161 -13.4496521252406 2.89363609099218e-36 2.38711323127414e-35 71.3929251744155

FMO4 -1.16399615167894 3.62780413072629 -13.4451825719339 3.03144133368379e-36 2.49890937250064e-35 70.7120051521447

LINC00462 6.74413100656376 1.64686780100668 13.4434363719356 3.08703923798142e-36 2.54378086227783e-35 71.3449491736031

PPARA -1.0026136734283 5.765182228505 -13.4429437497553 3.1029069223374e-36 2.55492935093591e-35 70.5991565467098

NR1H3 1.06192616602173 5.04707850556365 13.4414824157283 3.15045706235842e-36 2.59310501633591e-35 70.6998118825116

GPR141 2.37336443570809 -0.298240605390386 13.4398268167703 3.2052059852166e-36 2.63717493956846e-35 71.3095632886518

ANKLE1 1.86191340997826 -1.40506610102405 13.4393187550916 3.2221963506929e-36 2.65015644906632e-35 71.3156616344316

PCDHGC5 2.18237225871259 -1.32977738705388 13.4348764378973 3.37462853090245e-36 2.77343953688647e-35 71.270244348397

NUP153.AS1 1.35166431003688 -0.411555881267849 13.4309927966281 3.51376342666364e-36 2.88670196101055e-35 71.2018718490735

PGM5 -1.48930123824017 4.17381497291996 -13.4295777345597 3.5658674257035e-36 2.92840664614425e-35 70.4945808678627

GRB7 -1.35593158623192 4.02826897352521 -13.4186641972438 3.99444196489749e-36 3.27790237547521e-35 70.3949229648602

HLA.DPA1 1.71847367678177 8.81954448991261 13.4177902016224 4.03090527355409e-36 3.30658306843009e-35 70.3488082908158

BAIAP3 -1.31455623169047 3.01023308002806 -13.4176649697571 4.03615706649995e-36 3.30964879452996e-35 70.4939555244179

RAP1GAP2 -1.44652909620704 4.00009518635698 -13.4172393351484 4.05405772227479e-36 3.32308039778211e-35 70.3782126862391

RXFP4 -2.33140729202436 -4.63830921166952 -13.4152927405169 4.13693770978314e-36 3.38847450419382e-35 71.0695347177823

ESCO2 1.96032324162861 0.106930550710157 13.4106733157516 4.34043643749943e-36 3.55249278908895e-35 70.9884100127328

MT1F -2.27693620895402 3.79610719676458 -13.4079440453752 4.46532337072687e-36 3.65334000825512e-35 70.2768743912912

CLSPN 1.85824638711445 0.647826478578945 13.406288935095 4.54279547585238e-36 3.7153334406363e-35 70.9124328872697

EVI2A 2.22322213190672 3.32969047421119 13.4049262439689 4.60758403747239e-36 3.76691111002186e-35 70.7042672027094

ZNF710.AS1 -1.45859513192115 3.84080958464142 -13.400467041821 4.82609824275169e-36 3.94408096106405e-35 70.2170416867028

ADGRE2 2.18034805827369 3.03181369196915 13.3999231422901 4.85344768025e-36 3.96494923949545e-35 70.6802533821829

MSH4 2.34540936391826 -2.06667672005602 13.3996105126549 4.86923776666373e-36 3.97636221655091e-35 70.9122820741646

C5orf46 7.27167562106223 0.639453200347629 13.3942809182874 5.1464313071225e-36 4.19958787731695e-35 70.8440028830587

NKPD1 2.21250128535562 -2.78970562046412 13.3938753090731 5.16815980302611e-36 4.21574453809367e-35 70.8560292015777

FCMR 1.83910072001668 2.72986786352366 13.3937433845991 5.17524667748736e-36 4.2199502105646e-35 70.6206571501813

LINC02099 2.61426322625573 -3.77418531679408 13.3923763636771 5.24925423265708e-36 4.27710487495359e-35 70.8414751288442

MICB 1.28584106392785 2.36517121921644 13.3919594084043 5.2720364163022e-36 4.29406678365457e-35 70.5963360073579

SYNGR1 -1.51992944165403 4.03230749950282 -13.3900729628892 5.37634781311791e-36 4.37739674962987e-35 70.0941777068523

DACT2 -3.45966975074246 -0.749781020796816 -13.3896108944805 5.40220989670209e-36 4.39681537700673e-35 70.5120171602837

LINC02280 2.23021722096974 -3.78322529440962 13.3882381920997 5.47977362611574e-36 4.45828343453117e-35 70.7992340602295

MMP11 2.19617749417672 3.63105421395575 13.3871368182919 5.54280743479344e-36 4.50621171400822e-35 70.4832141968925

PLEKHG3 -1.06811878504349 5.3726355990763 -13.3856030213644 5.63179477977856e-36 4.57685427008184e-35 70.0077651362764

MICAL1 1.44126919242696 5.13041660054965 13.382244675583 5.83163890553311e-36 4.73574154599089e-35 70.1176624660493

BCL2L10 -2.90240173223182 -1.03242384494175 -13.3771491175479 6.1484293059785e-36 4.98929170529329e-35 70.4655960331209

LPXN 1.25128741768982 4.27980216355935 13.3709517451082 6.5569072948669e-36 5.31878601019771e-35 70.1093897257368

FCHO1 2.47659797578801 1.85902542526086 13.3701692599423 6.61036789585657e-36 5.36016213833594e-35 70.4952052520556

CTAGE9 3.08504884052376 -1.90379141210744 13.3693392044926 6.66755324076774e-36 5.40452674222913e-35 70.6005553835238

MND1 1.61035781198795 -0.360562761105405 13.3610091573934 7.2694435498979e-36 5.8880337248302e-35 70.4846276531749

PLCB2 2.2394595125803 4.10827001686336 13.3587125346547 7.44470364026716e-36 6.02552254262067e-35 70.1313290311387

CAMK2A -2.47709338172424 -2.33757664917086 -13.3565727483019 7.61178057491634e-36 6.15618952271083e-35 70.3724992720497

SACS 1.33944646323804 4.50007908801401 13.355976477342 7.6589999366799e-36 6.19208751817484e-35 69.9260759310585

RN7SL832P -1.48867450083709 -1.41706353543725 -13.3487332823647 8.25643846173184e-36 6.67263127604386e-35 70.2909966080136

LINC00924 -1.78635962183142 2.07189768278374 -13.3482362058354 8.29910102772883e-36 6.70463049016481e-35 69.8653415015516

EVI2B 2.22090021944691 4.15709433009041 13.3455177873841 8.53632860101005e-36 6.8937320368763e-35 69.9853264222668

SCIMP 2.13478403675705 2.37301303882377 13.343979917087 8.67351270185694e-36 7.00193103338307e-35 70.160321493302

ERCC6L 1.77208866058389 -0.604271629902724 13.3428280078656 8.77770409088244e-36 7.08342568308914e-35 70.307782115384

PANK1 -1.72222415060405 4.15867840785552 -13.3422321335419 8.83208987406141e-36 7.12468290948188e-35 69.587023873168

IGSF3 -1.30030159986136 5.36690152568442 -13.3384903439212 9.18136039122394e-36 7.40091631944639e-35 69.5212496573317

LINC00863 -1.06642945814728 0.658701322844517 -13.3384554463832 9.1846817637572e-36 7.40091631944639e-35 70.0365825652744

MYC 1.76500214480268 6.19726390698964 13.3370287403042 9.32149807774944e-36 7.50839283055874e-35 69.5632769357291

TUBB2A -1.19147509686973 2.93123923347537 -13.3310323949065 9.91907642404298e-36 7.98385182668918e-35 69.6192384704831

ADAMTSL2 -1.58847827972951 3.93793847022722 -13.3290078107767 1.01293380096656e-35 8.1500892314147e-35 69.4663806874976

CLEC4M -2.10952933914275 -5.39843433575899 -13.3285222349871 1.01804236754059e-35 8.18817808533843e-35 70.1803865614361

PPP1R13L 1.59252622686945 5.3186355225797 13.3282427767075 1.02099407366919e-35 8.20587844497711e-35 69.5570908178159

TLCD3B 2.81511899158427 -2.81693852532403 13.3265372152561 1.03919431529918e-35 8.34908579861509e-35 70.1630467238874

RPS2P50 1.64023274243209 -2.84194996021372 13.3229830613409 1.07816609547947e-35 8.65582795169466e-35 70.1248292106234

CXCR6 2.82552433948301 2.02151187344264 13.3222783185431 1.08606493801615e-35 8.71604006260259e-35 70.0074597871397

PPP1R3B 1.33105350002848 6.61281324330198 13.3213222762475 1.0968725934463e-35 8.79954360667478e-35 69.3549014794603

DTHD1 3.04754084451419 -0.896910514682481 13.3198006906933 1.11429483441824e-35 8.93603119873095e-35 70.0826712352155

GTSE1.DT -1.39910889553126 -1.27603083498934 -13.3174332890824 1.14195136632126e-35 9.15446192524523e-35 69.9639691373155

SEMA3B -1.9152384316284 4.24436055787534 -13.3106884747713 1.22455174934338e-35 9.81302874162112e-35 69.2565542964666

TACC3 1.47184367746344 3.8415434979504 13.3096129886916 1.23826198614072e-35 9.91925923135382e-35 69.5793342689105

AXDND1 -1.97976549750352 -2.36208417120815 -13.3087443041772 1.24944747293341e-35 1.00051944397266e-34 69.9067145514014

ZCWPW2 -1.04792782289225 -0.0599947692374795 -13.3062551564701 1.28205909548072e-35 1.02625778071576e-34 69.7760099499302

ACOT2 -1.07959521167641 3.8878954876573 -13.3038803845141 1.31396216246131e-35 1.05141029426097e-34 69.2335973109349

PLCG2 -1.54306366261895 5.36563120637546 -13.3037800323639 1.31532758658331e-35 1.0521176335873e-34 69.1643151635827

CSF1R 1.76242848495181 6.34884216122649 13.3031364252122 1.32411837844025e-35 1.05876176085089e-34 69.2016428325581

TNFSF14 3.52459903234875 1.45016616742793 13.2999815690299 1.36806323479274e-35 1.09310003180715e-34 69.8401510395538

ALDOB -5.07604802565614 4.80875451955716 -13.298945674887 1.38280696299752e-35 1.10447662874214e-34 69.1761668803254

GPRIN1 2.16520652058857 1.86869487217644 13.2965236454982 1.41789992599951e-35 1.1320923303934e-34 69.7175849932448

BLM 1.41789964738562 0.996670989719638 13.2935788212896 1.46176474662034e-35 1.16668900686247e-34 69.7101586016934

TNK2 1.34004796307536 4.98425326786996 13.2909325647625 1.50233400962248e-35 1.19863107383279e-34 69.1851306575115

BDKRB2 -2.10295327168073 3.72590372747322 -13.2871407064861 1.56242874526144e-35 1.24612245876636e-34 69.0359155384739

ADGRB3 -2.41621526007401 -1.08699084401695 -13.2853534291702 1.59157860328721e-35 1.26890796124171e-34 69.560075636532

FAHD2B -1.43788844992109 1.4683027498141 -13.2848933937999 1.59916887409534e-35 1.27449443492361e-34 69.3452670023036

SLAMF6 2.90537313407301 2.42020575624346 13.2836962770731 1.61908990420307e-35 1.28990053505467e-34 69.5847381103303

SPSB4 -2.66693310409157 -2.316624947789 -13.2835172815918 1.6220897078697e-35 1.29181947471124e-34 69.6145950631994

JAML 2.03589442799043 3.74774728315328 13.2818891419617 1.64963133340902e-35 1.31327480979917e-34 69.3656181794593

GPR22 -2.50148221719652 -4.62033835874348 -13.2804349918235 1.67462328536394e-35 1.33268545721261e-34 69.6793451889154

CD48 2.53929919857767 3.94889219146576 13.2792628428359 1.69504283312521e-35 1.34746347880266e-34 69.3638548079265

GAPLINC 2.03737730077749 -2.06642735541098 13.2757858398229 1.75708486705035e-35 1.39576792437846e-34 69.6370840344036

ASS1 -2.18880515744726 7.0254168214774 -13.2751331166876 1.76898145411606e-35 1.40419730173622e-34 68.9056276881132

PPM1J -1.36959255173988 -0.435946694968243 -13.2747265827016 1.77643148853006e-35 1.40959903118545e-34 69.4646204654135

SH2B2 1.35996773747261 2.38160799954007 13.2744197275095 1.78207552610273e-35 1.41356430025129e-34 69.3898084773444

LINC02446 2.84914773204112 -2.28182402110192 13.2671394530605 1.9213490536747e-35 1.52293220420578e-34 69.5511963305743

LINC01146 2.21214936962739 -1.70754209227135 13.2586759410249 2.09693852062971e-35 1.66090603448064e-34 69.4593716493555

CASC22 -2.02372295507336 -6.05516345884429 -13.2582031600923 2.10720546847297e-35 1.6678290873792e-34 69.4603050251602

CSF2RA 1.98909359522335 3.23100189176158 13.257452057812 2.12361952213742e-35 1.67960396008936e-34 69.1758811727859

CLEC12B 2.65508909696183 -3.18648193046176 13.2535881284127 2.21009337901444e-35 1.74673311434367e-34 69.4141272022686

LYPD3 -1.57262856363433 -0.17060885372644 -13.2526339142709 2.23198303734065e-35 1.76275841398645e-34 69.2010756199966

ADAMTSL1 -1.84990832337907 2.15510356770093 -13.2483723451063 2.33240922205642e-35 1.84140674601152e-34 68.8124872630316

SHISA6 -2.36764873403936 0.251945455019859 -13.2450483791291 2.41385400736164e-35 1.90501811566897e-34 69.0071064052087

MRPS6 -1.48483057391819 5.41567753776063 -13.2434814401228 2.45322398857628e-35 1.93469158507244e-34 68.5432102165686

NHLH1 1.70665743024459 -2.53893320121976 13.2433679129204 2.45610112927732e-35 1.93626183182169e-34 69.3061773640809

PLOD2 1.54627687467565 7.77685888164983 13.242673254621 2.47377932866764e-35 1.94949511970335e-34 68.5319984962395

RPL7P50 2.28990152047013 -3.39216323562325 13.2422281998262 2.4851719786544e-35 1.95706173872197e-34 69.2978000406119

HOXA11.AS -1.8986856870534 -0.668349856561571 -13.2421739342171 2.48656465615333e-35 1.95745307748266e-34 69.115366052775

LCN2 -3.45958452603203 -1.40245806056911 -13.2392366048615 2.56311989633191e-35 2.0169916850753e-34 69.043288163453

FOXN4 -2.59181684092088 -4.07820873758913 -13.2377560537742 2.60259264640312e-35 2.04731667033288e-34 69.2287626751814

PPM1F 1.00989134413939 5.92611849379384 13.2311738993682 2.78553740367192e-35 2.18965283749793e-34 68.4461083781986

KSR1 1.72274411585213 6.38544297084325 13.2303946491391 2.80802685241235e-35 2.20574445743232e-34 68.4487247543822

DOCK2 2.50008266573097 4.57498526525761 13.2284866984469 2.86385744551972e-35 2.24879183460088e-34 68.7508133638935

HIF1A -1.22123422324435 7.37658818073978 -13.2284434823154 2.86513476216602e-35 2.24898670824763e-34 68.414444750535

TNFSF13B 2.66013943070609 3.3129019992861 13.2264267478184 2.92537771495308e-35 2.29544988168293e-34 68.9060734477914

TCAF2 1.5893947161321 3.25511071933523 13.225186398334 2.96305373933384e-35 2.32417851276606e-34 68.8053322628668

MFNG 1.17720464389723 4.35327147733291 13.2250052125181 2.96859762642822e-35 2.32769156549465e-34 68.5853829697814

ALKBH6 1.5526220308994 1.06129765893066 13.2181002177605 3.18775447438983e-35 2.49863696301438e-34 68.9370559728892

LINC02360 -2.32167072136107 -5.30613009361238 -13.2133368218647 3.34826020145024e-35 2.62350412987781e-34 68.9969804978495

CXorf65 2.58483342145305 -2.56095508345875 13.213015884116 3.35935940463926e-35 2.63125738600652e-34 68.9971032710285

LINC02453 -1.36150637249644 -1.42693979187027 -13.2109140940218 3.43295838019921e-35 2.68794122115705e-34 68.8811397561457

UBAC2.AS1 -1.28227011304538 -0.62210853280526 -13.2108018620433 3.43693329844569e-35 2.69008966228272e-34 68.8274206425848

SEPTIN1 1.73797495849558 3.310485667754 13.2075674798601 3.55347616089095e-35 2.78031201374687e-34 68.6305260508025

TPI1P2 -1.54060973646442 -1.72490155263558 -13.2059238175207 3.61420140103536e-35 2.82580118843741e-34 68.8390549760539

HCG27 2.54348859606609 0.912064109971687 13.2029061282767 3.72839352210982e-35 2.91299905751398e-34 68.8347453553009

TMEM262 1.45890985435082 -0.151142233537366 13.2019455842508 3.76548996873856e-35 2.94093110389006e-34 68.8372111661992

SCIRT 2.76899186190545 2.59144508863589 13.2016383725108 3.77743197829357e-35 2.94920403792959e-34 68.7252243833753

CCDC142 1.05086935751922 2.65548422814048 13.201513979896 3.78227812089907e-35 2.95193299200027e-34 68.5829697063993

SH2D2A 2.68340732717433 1.78173977101206 13.1989028803277 3.88544391509044e-35 3.03028572007393e-34 68.7504877406006

IRS4 -2.10972624806178 -6.06460353026974 -13.1943098647917 4.07376310832311e-35 3.17602373193667e-34 68.8055459174961

HTATIP2 1.11839873247275 5.32475014138499 13.193013977381 4.12852154929666e-35 3.2175670976027e-34 68.1140886314685

LRIT3 -1.93990511862962 -1.30244788883706 -13.1892699244967 4.29088187127618e-35 3.34171922783316e-34 68.6207861290925

LY6K -2.30359605210357 -1.75700251271887 -13.1804704210663 4.69794571027254e-35 3.65353051980733e-34 68.542728815138

SORD2P -1.83162995100016 -0.14970457380206 -13.1742987827145 5.00617593397555e-35 3.89046808979971e-34 68.3705249455109

NCOA7.AS1 -2.44476104120491 -5.6281270502109 -13.1725704600362 5.09604791431734e-35 3.95609005582866e-34 68.5807543344831

TRBV28 3.21249498950246 2.12070047115425 13.1722888951081 5.11084061902734e-35 3.96616477441777e-34 68.4816795812349

INF2 1.29242516446349 6.99103247504899 13.1632734930884 5.60778218297939e-35 4.35026141443552e-34 67.7201173400142

SCARF1 1.50343252270348 4.70750912371429 13.1613604316779 5.71926977309793e-35 4.43202845218117e-34 67.9133790184036

HAO1 -3.05650195995616 -5.41677211434204 -13.1586309347906 5.88216899904163e-35 4.5566479665387e-34 68.4342735097658

SYTL4 -1.34960386407993 3.42524790501888 -13.1578951518463 5.92686658747059e-35 4.58964619192044e-34 67.7654643391973

GAPT 2.59911887752408 1.92153482294994 13.1564081391633 6.01823543266636e-35 4.65874951859929e-34 68.2995903689287

NOP16 1.12455138607974 3.90339909726268 13.155212872343 6.09269403162042e-35 4.71471822496463e-34 67.9336783616985

KIR2DL4 2.30986871014008 -1.43183028929228 13.1545099191045 6.13691191587386e-35 4.74725437513598e-34 68.3910134265525

BIRC3 2.10475642884559 6.79984222096212 13.1539906280089 6.16978203059631e-35 4.77099245274668e-34 67.6599210738565

GIMAP4 1.29071009234512 6.12441202187453 13.150561814957 6.39126438430618e-35 4.94051293209207e-34 67.6214835649771

SIGLEC1 2.94563751137572 3.85386145598851 13.1496623796844 6.45066249651597e-35 4.98466504725472e-34 68.0836558879755

SORCS2 -2.54462651189721 2.81178587319153 -13.1479249939758 6.56695943419628e-35 5.07273822960379e-34 67.6616834615809

PTCH1 -1.0016879741501 3.90448692110747 -13.1405062951047 7.08750841103759e-35 5.47097567313333e-34 67.5574492412078

SPOCK3 -2.79742531465708 -4.38716726226565 -13.1372171542098 7.33125717977177e-35 5.65524813927236e-34 68.2035470961682

RASSF5 1.77938880146254 4.50892235658557 13.134409709607 7.54590149892544e-35 5.81865156866682e-34 67.7053992615413

CCL5 3.20795370402922 5.24951380010011 13.132307782128 7.71069213511137e-35 5.94152874571892e-34 67.7579600457968

TUBB6 1.24678663586776 5.8201301608104 13.1305817729931 7.84868720946952e-35 6.04359977408518e-34 67.4375177928415

PKP4 -1.04761065657447 7.1493626623041 -13.1285613580898 8.01334781291965e-35 6.16821732144181e-34 67.3814538896514

ERFL 2.11742522461159 -3.56025297175515 13.1275558254799 8.09657482381497e-35 6.22570195724238e-34 68.1251678185914

HSF4 5.43753286137654 4.79312469983904 13.1249244926277 8.3184644497943e-35 6.39406977211941e-34 67.9356560663511

MIR122HG 2.64444961213108 -1.7441374676709 13.1218728005122 8.58339626357294e-35 6.5953923540035e-34 68.0613010225748

CARMIL2 2.47373343081849 1.00674161941947 13.121051282295 8.65614068612034e-35 6.64895052421047e-34 67.9905565142459

MIR155HG 2.87447013793554 0.90749546598595 13.120592438033 8.6970376862991e-35 6.67801702595553e-34 68.0056858009135

PLAC8L1 2.02892128097395 -2.30822015133209 13.1134684523444 9.35725866572223e-35 7.1824437520909e-34 67.9777232998981

FER1L4 5.32934438741919 3.03632652228282 13.112543595381 9.44655299140066e-35 7.24843829076821e-34 67.9111025671566

CBR1 -1.03197368981055 6.03310643387841 -13.1095600795013 9.74043466118012e-35 7.4713133959554e-34 67.16879666267

PTPRC 2.0894269001427 5.81435758661946 13.1095002298991 9.74642217917404e-35 7.47328294321018e-34 67.2955307295345

WDR62 1.52956398358638 1.0723662806504 13.1068063897793 1.00197511052128e-34 7.6801690951321e-34 67.795611789087

LINC02908 1.92620456569959 -0.856273375535575 13.1024813175973 1.0474647531385e-34 8.02040898750371e-34 67.8519221356367

LOC105372990 2.34055424871409 -3.70379748950901 13.1014313397158 1.05881482477228e-34 8.10163878352546e-34 67.8587342558679

SNHG12 2.65400613993457 3.17349095054802 13.1010088543786 1.06341629840503e-34 8.13399942913721e-34 67.6364476086559

CD52 2.49596446102915 3.94610191493466 13.097494404822 1.10247378240494e-34 8.42979690934053e-34 67.4996702705601

AFM -3.81035167291176 -1.41098628996651 -13.0939307999934 1.14353636927852e-34 8.74071363338351e-34 67.5017281953128

ABCB6 1.73288357495955 3.05525720812097 13.0898869220571 1.19198072927653e-34 9.1078163905175e-34 67.4556645456864

PWWP2B -1.06784127014345 2.75696226938043 -13.0892275959013 1.20007077389055e-34 9.16642664167433e-34 67.184055601353

FUT7 2.09890906312175 -1.73608002631265 13.087725046967 1.21871219600934e-34 9.30058640463655e-34 67.7116996440387

ITGB2.AS1 2.87280207218062 0.8889542606831 13.0877090828841 1.21891179530861e-34 9.30058640463655e-34 67.671079634289

IDNK -1.09097635004194 2.87587688292692 -13.0876641644893 1.21947358573846e-34 9.30162522483158e-34 67.1377960715255

SYT14 -3.66413480435179 -3.97834700715932 -13.0851458822531 1.25138548987096e-34 9.541705202425e-34 67.6257216619118

SNAI3 1.32109489089697 0.482510204128973 13.0839151924433 1.2672820852391e-34 9.65954496293342e-34 67.5916556607859

GABRG3 -2.77237429321856 -4.23867580460877 -13.0814682815083 1.2994882784742e-34 9.90157508699327e-34 67.6328348504177

LOC100506403 1.9938949416656 5.51090453910895 13.0792279173603 1.32969015789639e-34 1.01211142530512e-33 67.024472682942

RUNX1 1.9938949416656 5.51090453910895 13.0792279173603 1.32969015789639e-34 1.01211142530512e-33 67.024472682942

RAC2 2.29252678342702 4.80034635771938 13.0735037724495 1.41006872633751e-34 1.07291893720939e-33 67.1092722751101

M1AP -1.4768998581376 -0.501402758800203 -13.0730080160185 1.4172537484823e-34 1.07801065665102e-33 67.3999788260648

ZNF677 -1.1870690088755 2.87017902991254 -13.069300480897 1.47215587729977e-34 1.11899208301329e-33 66.9427159994838

ZDHHC15 -1.8803476497254 1.27540049096588 -13.0680020540314 1.4918799229696e-34 1.13359012366671e-33 67.0996916882213

KIF26B -1.59289454494436 2.52702416861969 -13.0628115329527 1.5733916029799e-34 1.19511041709836e-33 66.8928512429561

SDSL -1.04506910898918 3.82609677794669 -13.0611612630666 1.60022521104018e-34 1.21464819509764e-33 66.7539846699132

ETV7 2.54716890344828 2.56063929039765 13.0578543919705 1.65537469849796e-34 1.25607303077347e-33 67.2433155757213

ZNF582 -1.10929013661697 1.10330627234139 -13.0576411214252 1.65899574717562e-34 1.2583836884078e-33 67.100192536437

S100A5 -2.20672517385392 -2.76641568057768 -13.0568057572619 1.67325523512731e-34 1.26875942585833e-33 67.3428600094242

LINC02159 -2.69289312238905 -3.61762702771502 -13.056739128346 1.67439781869797e-34 1.26918541560897e-33 67.36136522785

TNFSF8 2.13417223891492 1.67279505786871 13.0562181704009 1.68335827691904e-34 1.27509283970578e-33 67.2685287522853

MMEL1 -2.05092417542162 -1.91589480780141 -13.042791609853 1.93151511725233e-34 1.46205056658522e-33 67.1612447044029

OPN4 3.15636367139382 -3.3175217190244 13.0421238085999 1.9447671975758e-34 1.47157193797174e-33 67.2543549757284

PRDM7 -2.12515798325263 -5.28271847270051 -13.0357530314482 2.07583259221287e-34 1.56965984905287e-33 67.1860033508686

ZNF502 -1.10578980749083 1.6913505594005 -13.0348617226027 2.09485762045063e-34 1.5834978754655e-33 66.7873148629509

F3 -1.94161447930097 2.43690956736093 -13.0336354362017 2.12131687205221e-34 1.60294390058634e-33 66.5705336846983

SNORD89 1.59312845955059 -2.36117720431732 13.0316219586747 2.16548488829266e-34 1.63575324105978e-33 67.1436817305001

PPP1R3G 2.01466156802813 2.73599386705883 13.0271177509729 2.26763390988058e-34 1.71232217804493e-33 66.8761474026821

LINC01948 -1.76149602064249 -1.26041575555237 -13.0252899225113 2.31044251126115e-34 1.74404491186839e-33 66.9539980165886

REEP4 1.12661869784132 3.65326917948331 13.0250539755965 2.31602682827663e-34 1.74765657038429e-33 66.6498068586643

SEL1L3 1.60518841889997 6.89540726019362 13.0231456966214 2.3616881447175e-34 1.78149710136388e-33 66.2979951653945

ZNF566.AS1 -1.05839197751806 -0.0129375279051572 -13.0189840583435 2.46440103563322e-34 1.85833525989278e-33 66.8314453707838

ALDH5A1 -1.16314685402161 4.99300504729915 -13.0157497843087 2.54728761417911e-34 1.92017510288569e-33 66.2188154758581

RAB33A 2.10965955529421 0.183367585640499 13.0152394695488 2.56061694717192e-34 1.92955731539821e-33 66.9392978095831

AFMID -1.00799364593895 4.88997689934692 -13.0115374451947 2.65941545162999e-34 2.0026259774111e-33 66.1806957499621

ABCB1 -2.28200484936688 4.84081174920715 -13.0032087789662 2.8957852495595e-34 2.17986893071387e-33 66.0948530517724

GJA1 1.74638239237298 6.93328822653274 13.0016977651616 2.94085772181693e-34 2.21303594334936e-33 66.0826670983714

NOC2LP1 -2.22131007642066 -2.82470006182658 -12.9993960228434 3.01086328543281e-34 2.2649361575409e-33 66.7612094120759

C3orf18 -1.05899806768035 2.81359344935361 -12.999321368662 3.01316140316941e-34 2.26588493267244e-33 66.2563963571242

CD180 2.40596433635997 2.23953630477005 12.9984681703883 3.03955022865642e-34 2.28494293590742e-33 66.6552479389685

SIX1 2.08907076630246 0.371347048887133 12.9979594214974 3.05539487684159e-34 2.29606410741469e-33 66.7556529573774

LRRIQ3 -1.52641966768324 0.020383011103878 -12.9966286953536 3.09722948838583e-34 2.32510326381641e-33 66.5678454024754

ETNPPL -3.54538217104358 -2.9240203840925 -12.9961663271142 3.11189828850775e-34 2.33531295668819e-33 66.6644804631688

PCDH7 -1.9873702073005 1.79152160892468 -12.9943905398582 3.16888174183214e-34 2.3772596190957e-33 66.2627411520641

CXCR3 3.26050105473645 1.49237601023194 12.9902792063281 3.30483013042429e-34 2.47839577351276e-33 66.6651205948493

RAB17.DT -1.87396866193514 -1.0694728081584 -12.9894003792521 3.33463514880749e-34 2.49903230133368e-33 66.5696718841459

TLCD3A 1.56831326964162 4.48389146008238 12.9887588129239 3.35656247131129e-34 2.51460266320074e-33 66.2048133000171

KLRB1 2.10371376070348 2.03670032951928 12.9881408349239 3.37781934617986e-34 2.52966025264114e-33 66.5492421739729

B3GNT8 -1.56116239930043 0.964058308151123 -12.9824300729009 3.58070904326314e-34 2.68068635568446e-33 66.3151534738894

FAM234B -1.07905190578569 4.15128550448777 -12.9814280554952 3.61753956896217e-34 2.70733192467569e-33 65.9114606955227

DYSF 1.42310047776396 6.9129115712546 12.9765266333714 3.80320627010872e-34 2.84433492883935e-33 65.8185932586623

PBX1 -1.70197973299951 5.60300065009109 -12.974789684542 3.87125454173814e-34 2.89423624702715e-33 65.7993854448393

IPCEF1 -1.21959002932323 2.57748501156446 -12.973695139341 3.9147567784777e-34 2.92475828649823e-33 66.0108089649903

RPA4 2.02796990115951 -3.71995865728817 12.9726234605572 3.95782179559481e-34 2.95592206763955e-33 66.5498129878873

LMTK3 -2.13822166617015 0.520857737612749 -12.972051515484 3.98099793688009e-34 2.9722155078456e-33 66.212567103102

ICA1 -1.01489855587643 3.51788868513556 -12.9689100989693 4.1107239175463e-34 3.06592661331533e-33 65.8515002514037

CADM1 -1.48417396825387 5.22087670681465 -12.9685263672137 4.126856053627e-34 3.07690840463701e-33 65.7343319234461

IFNG 3.30191796255421 -1.99487446518964 12.9677618773234 4.15918346657993e-34 3.09995348892125e-33 66.4972684433564

SLC35G2 1.40642285230215 3.15861027643201 12.9635177269721 4.34329300504371e-34 3.23496871299319e-33 66.1258600832932

VWA3B -2.10451334299258 -3.22716571653247 -12.961478741225 4.43460732412932e-34 3.30185600866092e-33 66.3994868798436

CLK4 1.43031668498181 4.96859133959905 12.9609470474863 4.45873142774249e-34 3.31868725784934e-33 65.8240706446535

RIT1 1.0394968364001 6.08535607091313 12.9596150668626 4.51974160433381e-34 3.36180780393147e-33 65.6644068251713

CST7 3.18379772431082 3.08521234907172 12.9569507085837 4.64428587489806e-34 3.45326911276445e-33 66.2142606819744

BEST1 1.7614265498356 2.4796487336449 12.9551836042391 4.72876742330622e-34 3.51369447471985e-33 66.1513996838146

AIM2 2.95158102418314 0.0693949007158911 12.9540592142798 4.78331700008821e-34 3.55301925230889e-33 66.3378696214833

PWAR5 -1.90166313189802 0.350236438245417 -12.9535513361837 4.80816162271981e-34 3.57026014071682e-33 66.0578380844545

KCNE1 -2.00049325867638 -1.96037243287977 -12.9532908660207 4.820953234952e-34 3.57854249468091e-33 66.2552626476523

LRRK1 1.63855946493948 4.78139029318721 12.9497126233435 5.00014883327805e-34 3.70400855775001e-33 65.7703676786457

PTPRT -2.71132266793131 -2.66800586362657 -12.9390298438686 5.57555897678592e-34 4.12606469081283e-33 66.1186297469757

JAK3 2.40672133327498 4.40138261750521 12.9376240041241 5.6560356823601e-34 4.1827867264506e-33 65.8062925863896

NANOS1 -1.52573518104035 0.679773952262492 -12.9282580759418 6.222515909933e-34 4.595493078059e-33 65.8037138917783

SIT1 2.92758761084023 1.16864681908896 12.9208129966268 6.71283944473826e-34 4.95113385238985e-33 65.9649200733013

ACAT1 -1.63407657960822 7.03981519620116 -12.9208087031321 6.71313302733315e-34 4.95113385238985e-33 65.2748521644005

HECW1 -2.76761795898052 -0.121051543640138 -12.9183929039575 6.88036571880588e-34 5.07276086548802e-33 65.6616136729839

TH -2.35440543506452 -5.4512566949873 -12.9167484476937 6.9965691076272e-34 5.15669560569906e-33 65.9795395450585

GPR65 2.02332104241581 2.7944840145284 12.9160205660102 7.04862590786143e-34 5.1933115969149e-33 65.7428960716491

MYO10 -1.33453050839432 5.86848998311924 -12.9149725738833 7.12425455179569e-34 5.24726439906947e-33 65.1899487246615

APOBR 1.93164898642982 3.2232025588267 12.9148006809186 7.13673616079615e-34 5.25468650006329e-33 65.6784904000407

GPRC5B -1.36931716899903 5.97481342906265 -12.9145245729586 7.15683069551126e-34 5.26770701209187e-33 65.1867924822425

NRG3 2.15782393921351 2.0295426715002 12.9091660087142 7.55816554073887e-34 5.55748962186294e-33 65.7478489085411

ZNF385D -1.73832200406491 1.48262597312948 -12.9081907025347 7.63358648972222e-34 5.6110583773932e-33 65.4624655990453

SLC43A3 1.61605742245206 4.69861416819455 12.9065229140716 7.76429791987555e-34 5.70521864300741e-33 65.3454495984988

PDXK -1.07946897986863 6.89438388109713 -12.9064560075409 7.76958790363844e-34 5.70718670447767e-33 65.1152905787238

COL5A2 1.92438741514345 6.20336945310406 12.9017836200844 8.14802730848924e-34 5.98315997219138e-33 65.1258114969081

EOMES 3.28427659631691 1.6605813263478 12.9015823042859 8.16473902293793e-34 5.99341759718719e-33 65.7589284712978

ZNF542P -1.14703629805333 3.00325039611044 -12.9014212189089 8.1781356898757e-34 6.00123570284935e-33 65.2217702880857

FAM177B 1.95039674951155 -0.605257323456865 12.901282891516 8.18965715534498e-34 6.0076729713244e-33 65.8058842228647

PILRA 1.62297268263132 3.2194396780429 12.8943750078888 8.78605726695477e-34 6.43868911652457e-33 65.4375499843952

PTGER3 -3.38937015227067 5.38313258962705 -12.8938691254608 8.83139560904168e-34 6.46974482884304e-33 65.0056098948706

SEMA3G -2.01580725670264 4.50620195550877 -12.8932394037112 8.88815833155436e-34 6.50914624730085e-33 64.9824557109284

PLXNA3 1.33447161015842 5.05644521622696 12.8895537373324 9.2277427902841e-34 6.75557330640128e-33 65.0778964552962

GLRB -2.62412809890522 0.677444951904613 -12.8860347565173 9.5640165982529e-34 6.99941241532554e-33 65.2623761957476

PRR7 2.24861717471838 0.84315287024538 12.8845571101315 9.70883254331422e-34 7.1030169926028e-33 65.5905042910148

NKG7 3.47820515544488 3.89252481112825 12.8831522506384 9.84853917457836e-34 7.20281548132734e-33 65.4163093137052

PPP1R42 -2.59174460312191 -3.35380870525974 -12.8768484556794 1.05004757622451e-33 7.67447815492786e-33 65.5296869493314

RPL36 1.08294659881264 8.10750852274314 12.876120632991 1.05784614138213e-33 7.72889058095073e-33 64.799196293375

PROCR 1.58786412602059 4.83805044685363 12.8742921327792 1.07769376555155e-33 7.8712706746651e-33 64.9878430661489

TRAF3IP2 1.01742247423322 5.23701799124363 12.8701780512042 1.12371656005043e-33 8.19919131445143e-33 64.8266408619547

CD5 2.47612176522501 2.37799862679671 12.8632647537687 1.20550187814443e-33 8.78420558289108e-33 65.2781967377942

PLAC8 2.03417541912017 1.25240698789806 12.8628193897108 1.21096933199978e-33 8.82110427073039e-33 65.3334680362122

EXO1 1.78922146375926 0.463514863606355 12.8627206651917 1.21218465004765e-33 8.82701471425899e-33 65.3699661562974

NLRC3 1.2798178964286 3.17515404202925 12.8621323223109 1.21945249143436e-33 8.87698044480847e-33 65.0814486732029

ADGRE5 1.1710790919248 6.3212863466687 12.8605080523918 1.23974323813641e-33 9.02168131301867e-33 64.6546758388713

CD3G 2.86554595958639 2.0559100399629 12.8601908739627 1.24374456073423e-33 9.04778624691248e-33 65.2942386670989

GAS6.DT -1.67718310596117 0.290814954497046 -12.8593344325058 1.25461323991433e-33 9.12381468613905e-33 65.1383771640091

LINC01684 2.12936133517389 -1.99386657106761 12.8578036366938 1.27427593159554e-33 9.25756380756562e-33 65.3833851135605

ZNF804B -3.66601869731224 -4.34066801347259 -12.855370765948 1.30615877495325e-33 9.4828862820775e-33 65.3119844474108

CTLA4 2.91379618843054 -0.288887981671232 12.8519689999957 1.35207503804259e-33 9.81298432625198e-33 65.3108438447964

LINC01928 -2.46136394096683 -5.62918310865221 -12.8494632399849 1.38692191729905e-33 1.00558741402575e-32 65.3005421292757

ARID5A 1.47183590403274 4.6971285333294 12.8492786927784 1.38952343836863e-33 1.00713949249319e-32 64.7401025004188

ADAMTS14 3.06975700577611 0.442690476743155 12.848010111855 1.40753831761658e-33 1.01985858272132e-32 65.260561308967

NCF2 1.89681284693229 3.80196544720483 12.8466867137984 1.42657952485715e-33 1.03331264026196e-32 64.9042041831142

ABCD1 1.00398575978155 4.57567126915765 12.8463917980651 1.43085763160211e-33 1.03606798619619e-32 64.6722284327032

LINC01738 3.42270041725208 -1.05514969972538 12.8426598287562 1.48611078309317e-33 1.07500757838249e-32 65.2264128039489

TRMT1 1.08547052375716 4.64398137721485 12.838629373975 1.54817126888802e-33 1.11915933638802e-32 64.5934801672266

TCIRG1 1.40528131004949 5.46795451823719 12.8354998621004 1.59813314785451e-33 1.15489430158944e-32 64.4880275254623

TMEM91 3.35087529178615 4.61904903212943 12.8326578317438 1.64489488676232e-33 1.18829381687329e-32 64.8212303569964

LOC105371730 -1.69998056175107 -1.79641700050423 -12.8285693532062 1.71456467544552e-33 1.2368538052314e-32 65.0014002723559

LINC02985 2.76871834216622 0.842382395465368 12.8285191268364 1.71543857733897e-33 1.2368538052314e-32 65.0434322256727

KRT81 2.81792409822918 -2.04385429893789 12.8285150261763 1.71550994538799e-33 1.2368538052314e-32 65.0901715862335

GRPEL2.AS1 2.21671706104986 -3.36416801016897 12.8275390408998 1.73258039288229e-33 1.24874931812852e-32 65.0838086167488

WIF1 -2.75330755332513 -4.37201363889286 -12.8261097181262 1.75788559687372e-33 1.26615273396445e-32 65.0495194398796

C16orf54 1.90201289010968 1.78461305702263 12.824539186579 1.78611531606557e-33 1.28563827411005e-32 64.9003891912935

SDC3 1.09693331918859 6.63425384010178 12.8243434229687 1.78966558464867e-33 1.28776957594097e-32 64.2765673951205

C4orf47 2.28974347694056 2.17083767462953 12.8214242588634 1.84344729169357e-33 1.32566623643444e-32 64.8640449656201

SH2D1A 2.75387033031949 1.40722891626605 12.8214190133215 1.84354536792398e-33 1.32566623643444e-32 64.9407908115321

PCK2 -1.52793531367438 5.47081976685248 -12.8209242052801 1.85282022314071e-33 1.33189737948335e-32 64.2388333113312

IL20RA -3.04027409635757 -2.94394311815838 -12.8187365277642 1.89438678194853e-33 1.36088212839977e-32 64.9023279825179

CD14 1.66358378144212 6.34273484992629 12.815964154065 1.94839913748681e-33 1.39830431367813e-32 64.2274587879789

FOXE1 -3.20892838019492 -4.56601986445514 -12.8086650925237 2.09804704896714e-33 1.50520755617462e-32 64.8687539258992

SLC25A47 -2.32120970954331 -3.76874027123631 -12.7949237033969 2.41151385619944e-33 1.72953108958078e-32 64.7285318116686

LINC03007 -2.14507817211282 -3.23885454456274 -12.7884398206429 2.57520636630641e-33 1.84571940711361e-32 64.6516936717202

CLECL1 2.20033433587523 -0.85769248502795 12.7882060666611 2.58130996230907e-33 1.84948742971607e-32 64.671986293941

LAPTM4B -1.17980833071535 6.40279094009714 -12.7880390136757 2.58568073838299e-33 1.85201183795095e-32 63.9112604869756

CD84 3.02485195405108 4.21376904023987 12.7838191886384 2.69856574390034e-33 1.92970409690622e-32 64.3369754470853

MTFR2 1.50223053265261 -0.21257563373656 12.7792295865181 2.826919305139e-33 2.02082654809298e-32 64.5529928351489

INSC -2.15882993629727 -2.91153796703126 -12.7740703295776 2.97847330732695e-33 2.12777303645034e-32 64.4899260522963

CD200 1.59343007890854 4.99745640361528 12.7719415323952 3.04334008940783e-33 2.17340231940618e-32 63.9280835189622

BCL2A1 2.44490290260293 1.27124218427176 12.7679211666651 3.16970569673402e-33 2.26290684713259e-32 64.3975957885555

PPEF1 2.28344404555981 -1.10243529720987 12.7671752884698 3.19371777037183e-33 2.2785607063642e-32 64.4639034229606

CYSTM1 -1.14254978855136 6.77719180425685 -12.7666468038836 3.21084082606247e-33 2.290029522583e-32 63.7018267418422

RBCK1 1.06710064212223 6.68734112179756 12.7645166418626 3.280790380483e-33 2.33915537307325e-32 63.6716242376797

TTK 1.76786657395542 0.809790539689124 12.7625244887713 3.34758024233055e-33 2.38599709835778e-32 64.3402892271105

AGRP -1.97805431983826 -3.51465622979795 -12.7617755618443 3.37303775888741e-33 2.40335813971198e-32 64.3975983449353

SYT3 -2.30302119600138 -2.2380388550153 -12.7577748240645 3.51233161079063e-33 2.50097695309898e-32 64.284881799671

PRLR -2.19889350651308 3.21101254697852 -12.7575109985087 3.52171597479208e-33 2.50657712348824e-32 63.6748847562991

GCH1 -1.01741589993491 3.39720219807913 -12.7574892571597 3.52249043437166e-33 2.50657712348824e-32 63.7240086353484

HOXD13 2.87129225741589 -2.33956222413256 12.7525359455585 3.70342867660314e-33 2.63361623396708e-32 64.3278721311454

ANO7 1.23580895070482 1.57406557718305 12.7521601306749 3.71752852999593e-33 2.64278305029281e-32 64.1493009292127

MIAT 3.66658753608648 1.36082896148469 12.7513462316791 3.74824793694011e-33 2.66375486718544e-32 64.2723972488282

CST5 -3.23191410899719 -4.70890133857965 -12.7502517726253 3.78995545805033e-33 2.69251939612399e-32 64.2848285383792

TP53AIP1 -2.54400869027259 -3.16318167010374 -12.7457332244688 3.96709582445332e-33 2.81745027792585e-32 64.2027140249928

LILRA2 1.99952849605761 1.7402566112748 12.7442377664673 4.02751893982341e-33 2.85943376841979e-32 64.1015733704643

SBNO2 1.07213106533894 5.84042213098831 12.7426894050651 4.09104516164981e-33 2.90264967264719e-32 63.4889396507197

SDHAP3 2.39654302213602 3.45617974205792 12.7399700284164 4.20503981135166e-33 2.98062714879883e-32 63.9295302258278

HAMP 3.20176104967217 -0.850982109150447 12.7378475732766 4.29620457171923e-33 3.04425935492154e-32 64.1702624703856

CTSW 3.07982415493631 2.99121236875841 12.7316971439699 4.57164451031181e-33 3.23838403513271e-32 63.9429412521355

PIK3CD.AS1 2.00684068673828 -3.87000567335728 12.7294331419561 4.67740335283929e-33 3.31115307643657e-32 64.098328445543

FFAR4 2.63572305382872 -0.0369840427510255 12.7268342250516 4.80181320377845e-33 3.39812253698738e-32 64.0463817335398

PINLYP -1.4997699511114 1.09807932182643 -12.7228403507554 4.99945046496119e-33 3.53684011041751e-32 63.6776294358386

SLFNL1 1.86658189492733 -1.07047597395937 12.7227554320129 5.00373944369635e-33 3.53872873990603e-32 64.0155832642979

FBLN5 -2.25319584302169 5.51345163380703 -12.721848341086 5.04978294124474e-33 3.57013609236562e-32 63.2500784965027

UPK1B -3.50455033951182 -0.654691558745513 -12.7213411009922 5.07571384025075e-33 3.58730836193401e-32 63.6880443673358

C3orf86 -1.80456751916871 -2.84026537423808 -12.714708515611 5.42723500278318e-33 3.83450910170775e-32 63.9058594823944

MYCNOS -2.25020062985731 -4.74043333343035 -12.7138142580175 5.47644799250012e-33 3.86802902327425e-32 63.9334626507011

KCTD13 1.00412709533122 3.61574574407527 12.7122963123239 5.56100313870627e-33 3.92648147302579e-32 63.4758997796238

CHEK2 1.18540799427319 2.44612283517278 12.7116058623021 5.59989257858465e-33 3.95266319508431e-32 63.6530791319239

PGPEP1L -2.61146748193767 -4.12285028032393 -12.7059074954467 5.93136525777452e-33 4.18392914713191e-32 63.8389766389433

NMUR2 -2.7037943331733 -4.75942675549332 -12.7050006959007 5.98588586851744e-33 4.22102497207847e-32 63.8416868879385

GALM -1.02128263187982 5.65742056584568 -12.7036945222913 6.06529660087106e-33 4.27564279415598e-32 63.055841027807

KISS1R 6.24216386159187 0.892979755951003 12.7029328741128 6.11208531214188e-33 4.30723638588315e-32 63.8121053599774

KIF1C.AS1 1.65550611040678 -2.34290045149272 12.6998378949961 6.30593827001088e-33 4.44241357235808e-32 63.7968644095727

FKBP11 1.82417589820248 4.15284619229687 12.697514313076 6.455486839098e-33 4.54630209135703e-32 63.3460980546834

HORMAD1 2.16193802673037 -3.79307156704279 12.6971486746345 6.47933917119048e-33 4.56163011945958e-32 63.7747930357105

PLEK 2.36577356051069 4.60394741036726 12.6949066997958 6.62752429543147e-33 4.6629519777226e-32 63.3182944098226

GZMA 3.2060536955917 3.26954065458569 12.691323222804 6.87141433056211e-33 4.82988154923685e-32 63.5231934556718

PDE8B -1.15349031786816 3.28056959905384 -12.6875999290877 7.1342881419677e-33 5.00982001177443e-32 63.0316758664742

DLX3 -2.26418149976871 -2.50417308411141 -12.6840572794615 7.39369588779008e-33 5.19031276054856e-32 63.5642937972389

MATN1 1.53997642698779 -1.99279931764313 12.6827380105652 7.49267970185071e-33 5.25810949019086e-32 63.6229307248137

ACO1 -1.03048914241799 6.44256178059686 -12.6813490210544 7.59832054403792e-33 5.33053286834223e-32 62.8369291859334

JAG2 1.65751792474253 5.17954802268304 12.6772128455754 7.9217639993621e-33 5.55387579974527e-32 62.9624027527397

RERGL -2.27151979325956 0.816111410644045 -12.6724028320117 8.31517509391222e-33 5.82782300600589e-32 63.1208884883996

CHMP1B2P -3.3059093997967 -2.50826080498144 -12.6718413991586 8.36234412520027e-33 5.85712519769236e-32 63.3859848239065

NPIPP1 1.87564655330503 1.0745947294351 12.6709059723432 8.44152680585577e-33 5.91069161449427e-32 63.4091599548261

HIBCH -1.12821882294035 5.20969240509587 -12.6705576745226 8.47120009603033e-33 5.92956872833283e-32 62.7273118296357

CYP51A1 -1.47295208524985 1.4245025601707 -12.6660989010638 8.86036631004455e-33 6.19998671064372e-32 63.0570958842355

REC8 -1.34981110897424 2.6467515145587 -12.6650858746972 8.9512365247535e-33 6.25956389681402e-32 62.8774533230337

MTFP1 1.52420474725258 3.38126858319523 12.6589615072931 9.52070283658424e-33 6.65353115087545e-32 63.0422990244361

HLA.DRB1 1.48029712457069 9.17307673189845 12.6579957908644 9.61373317904972e-33 6.71639741565772e-32 62.6187854247154

RNASE2 2.64575666218792 -0.407570443951853 12.6558126242859 9.82739297885928e-33 6.86347135720715e-32 63.3416515311443

SC5D -1.11786509889375 4.77830295381947 -12.6546623136066 9.94186520411742e-33 6.94120064874051e-32 62.5787613859289

RASL11A -1.18691613317117 3.97340693248873 -12.6539535634182 1.00130558038382e-32 6.98867162188685e-32 62.6169192124413

ABCG8 -3.08880381617747 -4.20877284421898 -12.6517464019206 1.02380219611083e-32 7.14112652142033e-32 63.286180366723

NID1 1.41705866617643 7.54136880030367 12.6465235188196 1.07906100322248e-32 7.51936227787652e-32 62.4818599534041

KREMEN2 2.30321980060322 -2.49845469900349 12.6453042409701 1.0923828059641e-32 7.60734272107505e-32 63.2539623410573

GRK5 1.27626784927826 4.55104126717645 12.6372009658564 1.18517589017907e-32 8.25092345590419e-32 62.6028595183349

TRPA1 4.04349670257539 1.5378310678931 12.6303527544925 1.26968667374048e-32 8.82801873409186e-32 63.0634826424611

TIGIT 3.12757618729246 1.75014016752175 12.6287433927493 1.29040218916628e-32 8.96919816789142e-32 63.0048624898228

MELTF -2.37899088795956 2.05167079753204 -12.626720715197 1.31691565695849e-32 9.14475940626437e-32 62.4927764938291

LAIR2 2.70309629855586 -2.26105151043002 12.6256285521605 1.33145659560292e-32 9.24279573815456e-32 63.0571837415238

SLCO1C1 2.28769376648364 0.199824592171297 12.6230947859087 1.36580934796335e-32 9.47825712322739e-32 62.9949720847709

MAP7D3 1.00254385446718 4.43285955708047 12.6211585417566 1.39265441546392e-32 9.66148474321681e-32 62.4275395995982

ABI3 1.15169322925217 4.54341344993283 12.6204716924207 1.40230291948716e-32 9.72224800112718e-32 62.4234800076524

ENPP3 5.24820563331368 6.84244972082576 12.6197603478699 1.41236567057398e-32 9.78890802380372e-32 62.5971889033892

SPN 2.2964190830632 3.80872173162296 12.616579595901 1.45824748075638e-32 1.01005014887383e-31 62.635116880533

NCAM2 -2.59458766709654 -1.98472306279849 -12.615480784911 1.47444003488458e-32 1.02094227130332e-31 62.8284573097541

CD3D 3.1654503539097 2.43657670866887 12.6151389739146 1.47951351644108e-32 1.02413078475727e-31 62.825354694312

SLC1A3 2.92073892570141 3.71935505903923 12.6110178317137 1.54206863808905e-32 1.0667561237151e-31 62.6504074125455

NPIPB4 1.9203543896533 1.01589481810394 12.6092869709687 1.5691202348463e-32 1.08512609152203e-31 62.7982241880825

PTGER4 -1.1088507524507 3.41825965986617 -12.6074094999116 1.59899754670462e-32 1.10473896263472e-31 62.208742824304

PON3 -2.07669286237974 -0.731369879117816 -12.6008657103398 1.70762885842048e-32 1.17941888252411e-31 62.6161381919385

HNRNPA1P21 2.29290579089188 -1.63445296906786 12.6004135574032 1.71540068683585e-32 1.18441236048733e-31 62.8003504568629

IDO1 4.02058051999538 4.15932514670356 12.5956338329714 1.79974169288326e-32 1.24186154766586e-31 62.544394888521

FLI1 1.21411231810105 5.28206424287021 12.594085045669 1.82794674729426e-32 1.26092551352972e-31 62.0626783626144

LINC00488 -3.39114603560676 -3.49633010628733 -12.5918142345518 1.870098011391e-32 1.2891877552974e-31 62.6440503392604

GRIK3 5.7068960049544 3.46962863596815 12.5912505215259 1.88071081425962e-32 1.29609503071635e-31 62.6434614511144

LIMD2 1.97882305386367 4.00083187441783 12.5838971983439 2.02476662275667e-32 1.39405245769066e-31 62.2519424145317

ZDBF2 -1.26194059932167 3.00365638697968 -12.5808039498366 2.08859945670634e-32 1.43754847015444e-31 61.9844724808741

HSD3B7 2.02223626220934 5.6239879509266 12.5747745049209 2.21883829722266e-32 1.52574805881708e-31 61.914287742832

TOX -1.72013717250754 2.43690428701824 -12.5729303247893 2.2602650293879e-32 1.55374557053205e-31 61.945121797859

FAM13A.AS1 2.21953536613998 1.65960591116882 12.5713634845348 2.29606609229301e-32 1.57785950675721e-31 62.3926412908053

FBLN1 -2.00708726252768 4.66435867385593 -12.567738105335 2.3810819893432e-32 1.6357681456497e-31 61.7070932108952

LRRC71 2.31055371716989 -2.16287940574844 12.5676337301751 2.38357546569737e-32 1.63696651954383e-31 62.4778338016607

TUBA1B 1.05063163830284 7.41051435080413 12.5670180754566 2.3983361570004e-32 1.64658624061985e-31 61.6864620822281

SHISAL2A 1.61129238433224 -0.501456268499607 12.5590041749769 2.59898247203228e-32 1.78378027516713e-31 62.3646852103525

MANCR 2.76443184099834 -3.17916922232565 12.5583650554901 2.61568588212201e-32 1.7946808032029e-31 62.3894036177003

SIRPG 3.38031010132363 0.954111948335785 12.5573630903162 2.64208761503935e-32 1.81222663689438e-31 62.3407311631413

GUCY1B1 1.17434964055863 5.86864431204687 12.5570867553011 2.64941558357942e-32 1.81668273448262e-31 61.6336677198561

TREH -2.57435795421932 0.518456242785392 -12.5537067264196 2.74070275756578e-32 1.87868812299514e-31 61.9383429950238

RPL37P11 3.11649761123433 -3.36991182059962 12.5516202467666 2.79860994690297e-32 1.91778059484699e-31 62.3218255596741

DDX47 1.52378763380096 0.71088612323363 12.550231020595 2.83783967521686e-32 1.94405361826063e-31 62.2106294085833

TMPRSS12 -2.17835453822554 -5.17588666106043 -12.549248904194 2.86590302690843e-32 1.96266307887903e-31 62.293767447353

SNHG1 1.51655581321112 4.51326605017969 12.5482031955744 2.89608719588328e-32 1.98271282868538e-31 61.7557499078055

RARA 1.05896060551144 5.89245198471061 12.5462789528697 2.95245937280376e-32 2.02067318114252e-31 61.5174241926718

PLB1 1.41893867111857 1.96173155832492 12.5445341452662 3.00451844550444e-32 2.05565878489695e-31 62.0494185083259

FAM102A -1.3346003854734 5.6240648837944 -12.5423487771243 3.07101304737368e-32 2.10049602892823e-31 61.4433941474411

RAET1E -1.48766129092058 -0.562079477570307 -12.5414915852053 3.09749316867377e-32 2.11728239646631e-31 62.0427411863159

LINC02310 2.71406239628859 -3.95613837133969 12.540548378186 3.12689314441727e-32 2.1367103153518e-31 62.2117021356201

KLHDC9 -1.11774274069895 2.27660659245181 -12.5341532141011 3.33369228092683e-32 2.27731095389478e-31 61.6531183051376

PDE7A 1.34066890599619 5.05331483996511 12.5339193724393 3.34150664054193e-32 2.28193576924259e-31 61.5002758975359

POLG2 1.07292530357991 2.95972202799712 12.5326289812425 3.38495710796386e-32 2.31088621306886e-31 61.7848420421392

HCP5 1.19835474615514 5.77789911661046 12.5324064436696 3.39250711947003e-32 2.31531724177947e-31 61.3970675397992

TCEAL5 -2.75020426579989 -5.02807255890028 -12.5319735799947 3.40724089191885e-32 2.3239211988484e-31 62.1178499904345

MAP3K14 1.40411170006672 5.11972876285692 12.5309554793656 3.44214647383363e-32 2.34699615889817e-31 61.4735312773578

ASAP2 -1.28839428705268 4.67477299771407 -12.5279293019005 3.54801435488289e-32 2.41767251940305e-31 61.3132716841715

ZNF831 2.43170990911633 0.831958651354054 12.5233357002643 3.71494272351894e-32 2.52905430956571e-31 61.9763594261081

P2RY2 -1.55984747381656 -0.0223837892633776 -12.5229486583087 3.72935961434022e-32 2.53807834482021e-31 61.8101718875074

FGR 1.47727799228454 3.89802380444693 12.5206478456089 3.81621922974044e-32 2.59638352514066e-31 61.5765659656497

APP -1.02187574424135 10.5987782451436 -12.5201406757721 3.83563498907423e-32 2.60878093421223e-31 61.3394212690674

ZC3H12D 1.75651979252989 1.23360569157651 12.5162387115308 3.98833764884751e-32 2.70926772273126e-31 61.8462145464726

LOC105375924 2.29786251560466 -2.97407891400882 12.5134588067993 4.10080560399346e-32 2.7848012698592e-31 61.9426778204333

FRY -1.24929944850547 5.33485240692166 -12.5127396500826 4.13041128561078e-32 2.80403472582952e-31 61.1492588010881

ADGRL1 -1.15043445435138 4.16178624918797 -12.5123036001444 4.14846575660412e-32 2.81541683785931e-31 61.1895412231166

ADAMTS4 2.74757797318027 5.62201169008712 12.5116385066081 4.17615508139136e-32 2.83332868654596e-31 61.3763568398885

LINC00996 1.92784102754666 -1.79604300758186 12.5093948546098 4.27092743839874e-32 2.89672803573332e-31 61.8951572796904

GMNC -3.58185160553242 -2.83943336708207 -12.5053447980773 4.44745491258847e-32 3.01365061100142e-31 61.7228978373679

AK5 -1.84482042379617 -0.500649895589426 -12.5021799384409 4.59043905229963e-32 3.10861061697873e-31 61.6260440872646

CA4 -2.44289559002378 2.11620747246616 -12.4988324981637 4.74665347169485e-32 3.21340205442837e-31 61.1845591772636

SLCO2B1 1.79575284697651 6.30143143889029 12.4911033584869 5.12785698803693e-32 3.46717384775901e-31 60.9832261841852

ADPRHL1 -1.32059569981384 -0.673781013762242 -12.4876724319123 5.30669370202394e-32 3.58698352831207e-31 61.5332694798988

ZNF844 -1.14625380172847 4.17495522085059 -12.4851636371566 5.44138125180816e-32 3.67574975257384e-31 60.9174795881195

LARGE2 -2.07619728498118 2.6925328426755 -12.4797715130816 5.74248903494233e-32 3.87675665371006e-31 60.9548301426056

KLHL2P1 1.63176148694216 -1.99695444073126 12.4788148880675 5.7976153892812e-32 3.91276371531692e-31 61.5925040333404

TRAT1 2.75748988412478 0.457618372071729 12.4774910950021 5.8747699413289e-32 3.96238726937841e-31 61.549028286704

FJX1 1.52677808919154 3.57111013769961 12.4761711477043 5.95271783234104e-32 4.01124707956055e-31 61.1927729679353

PLA2G5 2.78683142795275 -1.38561915389496 12.4758389890084 5.9724946443239e-32 4.02333309070315e-31 61.5614689319085

NOTUM -2.71172023135006 -3.84927334409301 -12.4709385676792 6.27198255066797e-32 4.22247796302364e-31 61.486349917484

CYS1 -2.74735362495814 6.08549681473651 -12.4701628614307 6.32074099819266e-32 4.25399300996317e-31 60.7544696840328

RNF182 -2.20660773618706 0.223161203291183 -12.4696827411287 6.35110868276185e-32 4.27311508757373e-31 61.1955191854663

HOXB7 -1.05840336330387 4.05011775214108 -12.4675306689933 6.48902176431887e-32 4.36456117622838e-31 60.7585587176887

DOC2B -1.33716910086881 3.5546226842752 -12.4671385081755 6.51447212633327e-32 4.38033105774649e-31 60.7829396034409

CDH8 3.15583656136246 -0.0239310737634721 12.4660121428957 6.58812454391285e-32 4.42849233030229e-31 61.4508661291606

SULT2B1 -2.55172971929847 -1.06246466000806 -12.4621272661191 6.84857179270038e-32 4.60214758259168e-31 61.2404780965144

DIPK2B 1.82448879926181 5.62825680044131 12.4617528694612 6.87420689039749e-32 4.61795398634665e-31 60.7706482471372

VENTX 1.85564974854462 0.27748811017803 12.4607404586144 6.94400641349635e-32 4.66341033048973e-31 61.362445406881

XCL2 2.38645662414698 -0.782694485197359 12.4606639412036 6.94931040309e-32 4.66553856340171e-31 61.4034541446295

SEZ6L2 2.63443128169923 5.60454516405828 12.4599657257179 6.99789555841831e-32 4.69671411664974e-31 60.8441430973767

SKIDA1 -1.54254476349569 0.438500107680868 -12.4547324474748 7.37297835332115e-32 4.94541730985657e-31 61.0768296975659

PKIA -1.97446025644766 2.01585000969819 -12.4540068089483 7.42654256280651e-32 4.97828940567517e-31 60.8013042985116

HEATR9 2.39371518321646 -3.31600751003299 12.4534294020671 7.46944138742379e-32 5.00551066051432e-31 61.3482576009269

RNU6.35P -1.80789734607274 -5.93491238436789 -12.4530327047522 7.49905712369093e-32 5.02381653353826e-31 61.3432099603291

MNDA 2.12355893871965 3.50930811311189 12.4499455866416 7.73355763169496e-32 5.17932684417499e-31 60.9956426113909

SPHKAP -2.39816245717611 -5.36245224442427 -12.4460711440339 8.03821789285927e-32 5.38006663438449e-31 61.2701700070822

LINC02072 2.66720064594784 -2.10732921395202 12.4440037973704 8.2056377499334e-32 5.48876038412288e-31 61.2514510978191

LINC02073 2.66720064594784 -2.10732921395202 12.4440037973704 8.2056377499334e-32 5.48876038412288e-31 61.2514510978191

NFKB2 1.17895307565011 6.00067967112289 12.4438663482384 8.21689104155494e-32 5.49460587304468e-31 60.4992566270457

NEU4 -2.3816196564357 -1.64763119205725 -12.442677687313 8.31485261528121e-32 5.55841156934048e-31 61.0938435047469

PCDHA11 -2.64255714055014 -1.34711980571621 -12.4384298696573 8.67452542283005e-32 5.79353313157411e-31 61.0148825027516

LILRA1 1.68262147694924 1.222253025798 12.4354981418427 8.93175253225652e-32 5.9598652851115e-31 61.043618311147

COL1A1 2.60132217966123 8.69446014852813 12.4348810154999 8.98685836066255e-32 5.99480512074355e-31 60.3766637825573

TUBA1A 1.05669796571411 7.09953757224129 12.4347712369306 8.99669634614243e-32 5.99770607847012e-31 60.37141534104

PLEKHG7 -1.86986309874433 -2.26106063046978 -12.4343279553293 9.03653090057577e-32 6.02242481763594e-31 61.0823462123827

ZIM2 -1.96199944603671 -4.87127044779401 -12.4316589030419 9.28011753045008e-32 6.18287830466236e-31 61.1270839069546

ZNF793.AS1 -1.3188925389433 -0.402265239213583 -12.4310285331435 9.33859467851113e-32 6.21994238066149e-31 60.9488152596778

LINC01014 -2.91104617165358 -4.81299986322107 -12.4259281501351 9.82542811292344e-32 6.54220233247154e-31 61.0621408641968

PDE1B 1.66222994293088 3.74146227776068 12.4251695374259 9.89996454947005e-32 6.58781745735594e-31 60.6746722971995

CCT8P1 -1.06599302044982 -0.205948908700971 -12.4195685509731 1.04679790592073e-31 6.96367568891496e-31 60.8341003122479

DCSTAMP 2.56949800855911 -1.74097067092989 12.4195300234549 1.04719965072106e-31 6.96422823104302e-31 61.0066103975568

TAS2R20 1.63545578464972 -1.61676564598636 12.4183040190702 1.06006423530893e-31 7.04763727843201e-31 60.9901867539052

IQCD -1.01949908823372 1.20743238232784 -12.4162825832766 1.08161972436045e-31 7.18875785780078e-31 60.6559438858514

LMNB1 1.45876954815838 3.72472707654009 12.4117261943193 1.13181979183237e-31 7.51554479213396e-31 60.5203860075306

RPL13P8 2.06475435685863 -1.91039162226543 12.4081445142619 1.17290325686094e-31 7.78598264647089e-31 60.8939760403859

RIMS4 -2.79504329145886 -4.37384645564217 -12.4076040301114 1.17923042677781e-31 7.82560659470863e-31 60.8738773755254

MIR3667HG 1.62449360444443 1.44638758764749 12.4063899049293 1.19356770423475e-31 7.91834700687373e-31 60.7370129027749

TTC24 2.75810966815987 -2.68009658121439 12.4049178686243 1.21118345915504e-31 8.03033741896696e-31 60.8677173715025

SLA 1.85092879994223 4.77939437270245 12.402842860508 1.23645524379598e-31 8.19292190499502e-31 60.3088009129616

LINC01266 -2.37050503652049 -1.97704764510324 -12.3990358303069 1.28419400017637e-31 8.50151211325486e-31 60.6900654663384

FPR3 2.4839553701191 4.53325022786676 12.3950950247562 1.33554304668507e-31 8.83877110817954e-31 60.3544278542929

SPRR2A -2.37226013113456 -5.67760167660868 -12.3921440349723 1.37532814164934e-31 9.09381115868777e-31 60.7387339564825

ZNF99 -2.21696121303255 -2.6714785015786 -12.3916549304663 1.38203524033883e-31 9.13539507172548e-31 60.6659118306543

TASL 1.9664330372454 1.34390933703688 12.3875618462005 1.43945513533554e-31 9.51206926897112e-31 60.5755871857752

MYCN -1.86237602593723 -0.470045688172916 -12.3867745907751 1.45076840490232e-31 9.58393045717365e-31 60.4749247417603

TNIP3 2.93046906361779 -0.884270169434779 12.3853272438528 1.47179892913265e-31 9.71762955873608e-31 60.6620872087706

RAB24 1.55294806734238 3.46109429761544 12.3853205781596 1.47189648283606e-31 9.71762955873608e-31 60.3087194870574

PMEPA1 1.64392647636342 6.62716777503514 12.3835436170754 1.49813352041375e-31 9.88487675773e-31 59.8885909488448

HLA.J 2.40453901058793 2.72261735760763 12.3813534610046 1.53111265555475e-31 1.00963804652499e-30 60.4310627816963

LOC101927692 -2.41933038012075 -3.63488301801088 -12.3804375824979 1.54511719008618e-31 1.0185654888372e-30 60.5920709761413

IL5RA -1.90029752452805 -2.42787889435922 -12.3742828700304 1.64259231233778e-31 1.08249607362839e-30 60.4969702290688

ASPHD1 2.99370631018618 2.82145641275393 12.3696771284735 1.7195174345628e-31 1.13148492916293e-30 60.3446974660888

ENPP1 -1.44987226882681 3.07722653575294 -12.358645346468 1.91866166006483e-31 1.26168760334626e-30 59.7546900853311

GPR82 2.21170317201846 -0.0871044808791158 12.358621374867 1.91911847047223e-31 1.26168760334626e-30 60.3796182622896

C1orf54 1.21966114057421 3.50548985575512 12.3570717162577 1.94888008405246e-31 1.28086846546762e-30 59.9779059779022

DIAPH2 1.06786253409194 5.28898463987711 12.3566036727866 1.95795897760027e-31 1.28644851285324e-30 59.6899778084456

C3AR1 2.09716806063757 4.13882290156623 12.3557010339541 1.97558698621088e-31 1.29764058940987e-30 59.9772704599776

HTR6 4.45091606522942 -1.66354504610626 12.3531994102274 2.02527205164208e-31 1.32987590578529e-30 60.3537488753782

MAP3K12 1.31538897089233 3.7989930275055 12.3519334054116 2.05088827781946e-31 1.34629202568905e-30 59.8938771009003

HSPA8 -1.09366176332334 9.60223579333235 -12.3499957399336 2.0907206182304e-31 1.37161566106841e-30 59.6224653276995

CYTOR 1.85119452488394 3.64380759857023 12.3488337474069 2.11497587725483e-31 1.38711188012154e-30 59.9606531756686

MSR1 2.14299321750828 5.2969171646602 12.348256723834 2.12712443976328e-31 1.39466097756217e-30 59.7289326815082

ORAI3 1.18081866735471 4.78114920693056 12.3477766136896 2.13728551645925e-31 1.40090283116928e-30 59.6823814175498

RTEL1.TNFRSF6B 2.25322287247065 1.1218707576893 12.3407776178979 2.29101678898313e-31 1.50031734760708e-30 60.1452104067214

LINC00926 1.88731531759266 0.349114135455349 12.3406194889953 2.29461428572835e-31 1.50222306728645e-30 60.1736409902793

FRRS1L -2.54636412446476 -4.38782035994566 -12.3388832768403 2.334485477638e-31 1.52786795875481e-30 60.2013707283295

LOC102723878 2.01709143162897 -3.16432360123293 12.3364659937257 2.39114782645182e-31 1.5640153681967e-30 60.1931483292368

LINC01589 -2.59439387842375 -3.99749039232897 -12.3357104142989 2.40913820793346e-31 1.57531110885607e-30 60.1596261031338

CALHM6 2.89773837706494 2.87054400503533 12.3282923401656 2.59307492272607e-31 1.69406476192325e-30 59.9301767649096

METTL26 1.41390569437744 5.74537664921409 12.3264970253908 2.63965209398428e-31 1.72397839838131e-30 59.3755371129545

KLHL21 -1.01651448283464 5.55585067126586 -12.3244244939707 2.69445754537685e-31 1.75924651147655e-30 59.2816991239151

EDARADD -1.514907387035 -0.112159034964437 -12.3242752533055 2.69844745367266e-31 1.76079940893128e-30 59.8558317365543

ARHGAP40 -3.5132399938006 -3.13604220896564 -12.3227283434226 2.74015158497539e-31 1.78747858467067e-30 59.9495431445622

CENPE 1.76084415125956 1.3497477589159 12.3204062859491 2.80396050188765e-31 1.82855711273503e-30 59.9040265591054

STPG2 -1.44679739351275 -1.31890019228146 -12.319975167957 2.81596913318711e-31 1.83584049724158e-30 59.9163641829443

RGS10 1.59748799373331 4.09870783109204 12.3170225955455 2.8995989507124e-31 1.88923482021223e-30 59.5404673914347

DPRXP4 1.65179877013541 -2.57930537738463 12.3153897824411 2.9469040514324e-31 1.91891222395567e-30 59.9828097596203

B3GALT9 -1.45127801242248 0.268704651511081 -12.3152004131918 2.95243983369872e-31 1.92194422656593e-30 59.7273114189608

CPEB4 -1.16018281288637 6.31349272283613 -12.3129407991543 3.01929847084849e-31 1.96488175948339e-30 59.1733580595651

RPL36A 1.1145753052067 5.78107906949389 12.3117219745378 3.05598504407652e-31 1.98816436940173e-30 59.2050478409016

WEE2 -1.66663867937382 -2.9895664498759 -12.3086007662398 3.15196849460158e-31 2.04938909588005e-30 59.8838077537612

LRRC52.AS1 -2.39378502420958 -5.85255704921057 -12.3078724175724 3.17479497549253e-31 2.06361673407014e-30 59.9092240148927

NBPF15 1.2053333680879 3.65557728380171 12.306301151238 3.22460033601269e-31 2.09474408867079e-30 59.452018537696

FDXR 1.28923019600724 4.45104111920115 12.3008948200136 3.40198351530542e-31 2.2080055170766e-30 59.2864419996436

CTF1 -1.43457435862247 2.24788579794584 -12.2992377554065 3.45827214263048e-31 2.24320632629576e-30 59.2954714296573

COL8A1 2.1749604051654 6.12696498969038 12.2992009783123 3.45953187638438e-31 2.24335756957353e-30 59.1335870176472

NKAIN1 3.28608118958728 -0.102243840245106 12.2980959008682 3.49759809734039e-31 2.26736906618747e-30 59.7960321946332

SPATA46 -2.18864943779863 -4.45025813141417 -12.2931959443651 3.67146972987558e-31 2.37937805477376e-30 59.7577492257849

THOC6 1.02607668489979 4.48526606715509 12.2893136913876 3.81531432721231e-31 2.471866705976e-30 59.1336854887593

GAL3ST1 2.69950740208087 6.44537625449893 12.2880957630669 3.86158622345532e-31 2.49962214873132e-30 59.0453438206721

SYNM 1.18387516560467 5.20751702991932 12.2879443340901 3.86737825488283e-31 2.50263005638006e-30 59.0317333030219

SLC43A2 -1.18439440804255 6.44931311563404 -12.2867359539653 3.91390861261907e-31 2.53199067233761e-30 58.9175941852319

SEMA6A.AS1 1.96280286880431 0.154063038430906 12.2838727012639 4.02639816065412e-31 2.60322127233061e-30 59.6271535667694

SIRPG.AS1 2.84388185638752 -3.66924255394854 12.2835218050237 4.04040339950172e-31 2.61150356371816e-30 59.6726661071664

GPR4 1.8390709636144 5.35577277812849 12.283240282949 4.05167476510627e-31 2.61801444831069e-30 59.0437676799993

SIGLEC7 2.05232544159848 1.07278492545023 12.2818100785647 4.1094212891644e-31 2.65389424931319e-30 59.5586004877342

MSC.AS1 1.96914759682979 3.99918557095505 12.2818049041504 4.10963169344064e-31 2.65389424931319e-30 59.2517141580002

TLR6 1.70784103876575 1.59236044833957 12.2790023692534 4.2251794054749e-31 2.72770592460393e-30 59.4730087804982

SNRPF.DT -2.01803668513268 -5.10625089105846 -12.2788353849532 4.23216528472696e-31 2.73140897717479e-30 59.6223026664639

DSCR8 -2.47013683849519 -5.83973761639842 -12.2784539639923 4.24816538204556e-31 2.74092583684209e-30 59.6199257480759

MYBL1 1.56797537805377 2.49587139030681 12.2756880897036 4.36600514567063e-31 2.81612486565349e-30 59.3478768469602

BTBD19 1.84499488894656 3.13566103086617 12.2750807133442 4.39231481173346e-31 2.83225894307499e-30 59.2902521000375

RGS5 2.23483821265052 9.3717276719207 12.2744633422583 4.419219109278e-31 2.84876681991304e-30 58.8029302154704

AFAP1L1 1.39752778754097 5.10368704918193 12.2728577105833 4.48996098980936e-31 2.89351570363622e-30 58.9211866903655

ZNF518B -1.00336330552342 3.70920782417471 -12.2702256003166 4.60837399842857e-31 2.96895038289091e-30 58.8419353010878

AMT -1.19412460138823 3.98044901438109 -12.2700781172479 4.61510013387169e-31 2.97240740422924e-30 58.8035162918465

ZNF853 -1.3135439784935 2.27096886949377 -12.2692415836396 4.65343648928095e-31 2.99621530937704e-30 59.0061354250924

DCDC2 -2.10836987279458 4.73019162176306 -12.2631053311025 4.94450429513914e-31 3.18081402301076e-30 58.6889440740311

KHDRBS2 -2.18581882268708 -3.67143316985967 -12.2608813156491 5.05441302371099e-31 3.24960540768333e-30 59.4231209001342

ZNF205 1.01662656431209 3.89954021825629 12.2541783347356 5.40059075778891e-31 3.46809020951987e-30 58.8806687257842

CDCA5 1.62281921262015 1.89935941063192 12.2476092633935 5.76272698788844e-31 3.69629800018568e-30 59.1351452854364

EML6 -1.99709340215698 1.10367658328976 -12.2467916447893 5.80945891784714e-31 3.72517915879441e-30 58.8934464751214

LINC01687 -2.67967753780685 -5.41482927319865 -12.2455898523767 5.87883411683865e-31 3.76855857891684e-30 59.2947736845542

ENTPD8 -2.19228934864026 -0.371156188943795 -12.2446085532554 5.93609195911443e-31 3.80414714318263e-30 59.0451256871897

PLAC9P1 -2.23045023635555 -4.92747887245757 -12.2440360505897 5.96975290225189e-31 3.82459719064528e-30 59.2789943932069

LOC645967 -1.26444050960989 -1.87397268620029 -12.2430611552939 6.02751066132967e-31 3.86046865422149e-30 59.2051336346546

DUSP4 1.95869169555281 3.61021798024918 12.2362207199217 6.44871576302246e-31 4.12661158328931e-30 58.8650512651556

CFLAR.AS1 2.03748528651999 -0.0394192383502728 12.2332845656997 6.63837805172366e-31 4.24673523314746e-30 59.1419360584924

CCDC113 -1.61564082473823 1.72368897852545 -12.2295684555341 6.88639971659066e-31 4.40411158930804e-30 58.6756642793304

RCSD1 1.26582284394385 4.77320454067714 12.2147738186405 7.96866585671721e-31 5.08881516560027e-30 58.3804114045178

RASGRP3 1.02306267055166 5.34652432535909 12.2120296452386 8.18726992423632e-31 5.22536243149347e-30 58.2556865589109

LGR4 -1.16068851340933 6.42066047212626 -12.2103368585556 8.32508463098106e-31 5.31176859681253e-30 58.166125702544

C1orf210 -2.24565140238827 2.63172420057045 -12.2094055310166 8.40188858125874e-31 5.35920814846023e-30 58.2810506122435

EMB 1.83552749352999 4.10229536960237 12.208135665768 8.50774809183989e-31 5.4251479151146e-30 58.4948324945836

NLRP12 1.6839743213252 -0.664738730988215 12.2072750180339 8.58024761799126e-31 5.46978270700009e-30 58.8998312963515

PKD1L1 1.75875607385215 0.0570850432005799 12.2072077264599 8.58594199250532e-31 5.47181657515949e-30 58.8733590350773

CD2 3.31586581552562 3.63505232473425 12.200646130261 9.15964911624665e-31 5.8340370777423e-30 58.634580244485

HPCA 2.53550695086965 -0.163351369847038 12.1984189811617 9.36292329337355e-31 5.96177047581854e-30 58.8140986148454

LDLR -1.57426910131723 3.78398291816611 -12.1977248132801 9.42719362854604e-31 5.99745159722319e-30 58.0925633912167

DSCC1 1.09250592644036 1.30844995321394 12.1975685345417 9.44172330949098e-31 6.00494701636514e-30 58.659851566607

NINJ2 1.53753929379182 0.787374770412284 12.1949814450398 9.68551594989215e-31 6.1564159410411e-30 58.7018456519686

LOC154761 1.61732962412935 -0.00374092361130358 12.1939910899211 9.78049044675909e-31 6.21497696228631e-30 58.742329204448

TMEM44 1.71378033849255 4.074350003119 12.187840832857 1.03913962415181e-30 6.60125529261325e-30 58.2961446554808

MMP16 2.90812505999975 1.55229737024325 12.1874065217064 1.04359446211214e-30 6.62762853255932e-30 58.6424572322387

FBXO39 2.10204378317845 -2.14478265241241 12.1826886176431 1.09322888121781e-30 6.93881229777893e-30 58.6809178370894

TEK -1.69373389221293 5.08586782336542 -12.1808098877261 1.11364167543277e-30 7.06427040151885e-30 57.8753707166739

IGFALS -1.7661965287619 -1.68367442985625 -12.1734476520519 1.19735505678922e-30 7.59309345792655e-30 58.4844540613817

IGSF22 -1.13434330657528 0.213084382190567 -12.1734168726325 1.1977178647698e-30 7.59319074523196e-30 58.370929368727

ATP6V1G2 -1.16860356023552 -0.19055180901464 -12.1732327259504 1.19989075339496e-30 7.60476004464619e-30 58.4010679685403

ARHGEF26 -1.51491502325542 1.21590598481536 -12.1729339145875 1.20342500866005e-30 7.62494830798728e-30 58.1967470743292

CD101 1.47669704527688 0.820181339857165 12.1720817964693 1.21356060762318e-30 7.68693911837374e-30 58.4717942337711

CCDC73 1.6156182713863 -0.886072596935158 12.169196357977 1.24851669716005e-30 7.90377618280374e-30 58.5320668032414

PKD2L1 2.68622864089194 -1.34005794008611 12.1658265750464 1.29061056768848e-30 8.16788668858855e-30 58.5113006101552

LINC02915 2.21623047761968 -3.10440382819064 12.1657274008951 1.29187056933169e-30 8.17349379027374e-30 58.5195927479581

PNMA2 3.1153386543166 5.52654999683265 12.1567990267051 1.41046730674324e-30 8.92125674508249e-30 57.9328261412332

TMPPE -1.29269006248945 0.678971624588137 -12.1553242181979 1.43107414657537e-30 9.04897665657166e-30 58.1249394151397

N4BP2L2.IT2 1.94358620120444 0.955280685460936 12.1534637338316 1.45749768952363e-30 9.21339225026319e-30 58.3040289689062

ASB9 -1.03513252056331 2.34578363697485 -12.15338417704 1.45863835522224e-30 9.21793637266384e-30 57.8848259527463

FABP5 2.02133016061461 2.79051037009482 12.1518790055462 1.48038728856252e-30 9.35267517113524e-30 58.1437828998982

MIR1270 2.5612397647421 -2.78224277631942 12.1507247042249 1.49728467982208e-30 9.45669425091096e-30 58.3728161770042

PMFBP1 1.82077699991314 -0.663283055391584 12.1492564236454 1.51905587622569e-30 9.5914267735221e-30 58.3352102542233

CLCN5 -1.62833261281016 6.20242246064645 -12.1398404465135 1.66635696482247e-30 1.05123841663584e-29 57.4798947154362

FAM83E -2.79214039877336 -3.72859690677719 -12.1389312882328 1.68131084114562e-30 1.0603661128876e-29 58.2142940657562

IFITM4P 2.04701457582727 -2.6857274226199 12.1381861199502 1.69366691631612e-30 1.06785063826475e-29 58.2493351669129

LYZ 2.49045672492383 6.63251503273332 12.1371310146135 1.71131692901486e-30 1.07866769108052e-29 57.5210420832964

CENPF 2.08086763575775 3.1270382118163 12.1366235993891 1.71987012424618e-30 1.08374630407012e-29 57.9614634044208

IFI44 1.48847968461819 4.56149975878915 12.1363919024804 1.72378983334691e-30 1.08590311986538e-29 57.6811298139988

KCNJ11 -1.75576463362859 0.5029724096674 -12.1352487928233 1.74325876583096e-30 1.09753482597822e-29 57.9138258597151

TACR1 -2.07313514668702 0.0325246374313815 -12.1351005513511 1.74579952202064e-30 1.09881788464047e-29 57.9359958201682

FSCN1 1.41309213068127 6.19063351796242 12.1339373109417 1.76586497290335e-30 1.11112718839208e-29 57.4489144487104

HES5 2.79562044390155 -2.75595945045336 12.1305413725539 1.82576593499836e-30 1.14815720798616e-29 58.1761913308117

LINC01460 -2.31554005596982 -4.13112774445215 -12.1298881987474 1.83751699752758e-30 1.15521458420513e-29 58.1533985789645

UBE2Q2P13 1.56233235145818 -0.182207679030887 12.129800130951 1.83910714659186e-30 1.15588175077572e-29 58.1226487200921

ARPC1B 1.09838083359134 6.75810042992056 12.1259744690584 1.9095209191658e-30 1.1994469861032e-29 57.3370159722642

TAS2R5 1.70339869575595 -1.05579487406616 12.1235326833076 1.95585940910833e-30 1.22820102492081e-29 58.0907292148912

GACAT2 3.59348315495371 -0.998816493552202 12.1223115528774 1.97945113215376e-30 1.24265859209871e-29 58.0863210435876

GATA5 -2.67984036519774 -1.95740472643765 -12.1218360914071 1.98871324105726e-30 1.24811460243608e-29 57.9457647876721

SLAMF8 2.74957476509056 3.50894182836758 12.1195920211343 2.03301368886314e-30 1.27481917195771e-29 57.8087356653619

ATP8B3 3.32465797140621 2.02487998657247 12.1176114389928 2.07292752164742e-30 1.29947461648196e-29 57.9555530389064

CFP 1.5618992493781 1.50066461239606 12.1161338665823 2.10321134284151e-30 1.31808079940107e-29 57.8783136649377

ELOVL2 2.96753051678926 1.2222386513251 12.1143431922558 2.14050296023412e-30 1.34068246460293e-29 57.9543168088848

CCL4 2.71155077411126 3.26584165247202 12.111553374351 2.1999172890092e-30 1.37750121824407e-29 57.7526027670782

SYT17 -1.50453371972441 2.28910697978101 -12.1104912633139 2.22296569695649e-30 1.39153449944401e-29 57.4326389533282

SIPA1L2 1.17121784640025 5.43649453754455 12.1082317005213 2.27280184075498e-30 1.42232355744612e-29 57.2435649339617

WDR31 -1.06124947984018 2.72533793746019 -12.105136692569 2.34287315777149e-30 1.46575456961868e-29 57.3521797238208

TRGV2 2.12101264470835 -3.33100153378852 12.1034737153519 2.38140661681885e-30 1.48900940764928e-29 57.9132107989782

ADCY7 2.14779075749487 2.66496299416563 12.1022448867671 2.41028478477892e-30 1.5066348227052e-29 57.6711846155347

LINC02328 1.47997503331998 -0.777010340562254 12.0924647272313 2.65289750082643e-30 1.65639340244457e-29 57.7792669456386

CHRNA1 3.47679312184534 -2.13286653329632 12.0895489872981 2.72982144238594e-30 1.70393567496315e-29 57.7754540811661

CRYGS 1.93415334187534 0.784150255853704 12.086172782384 2.82166990718231e-30 1.76026127552541e-29 57.6596138368025

IKZF3 2.83949961228635 3.34642402879736 12.0790374975085 3.02602199008386e-30 1.88666643507283e-29 57.4365150599987

SLC2A13 -1.06013041658437 4.80873077912383 -12.078305894061 3.04778960201842e-30 1.89969612586733e-29 56.8828823528657

RPL7AP64 2.00531410457633 -2.81816917600189 12.0778636146287 3.06102435760725e-30 1.90740134835447e-29 57.6626122984857

CILP -1.9300860915109 -0.163926319372233 -12.07579398279 3.12371979010858e-30 1.94535903600008e-29 57.398718172373

ASAP1.IT2 2.23812673869694 -2.60062831893324 12.075268227197 3.13984898968576e-30 1.95373348438505e-29 57.6370524305378

EXPH5 -1.37322361627798 3.23138585302809 -12.071217305444 3.26693440027224e-30 2.03165388301506e-29 56.924043123353

GRAMD4 1.50049101064855 5.97286421414969 12.0694890465769 3.3226986951964e-30 2.06574493843889e-29 56.8430152857702

C3orf85 -3.42696555017955 -2.81231774274963 -12.0607301041336 3.62021513951225e-30 2.24623967756278e-29 57.3629978602746

RGS18 1.94280703937028 1.92987868514354 12.0589624601164 3.6833969027406e-30 2.28414507705988e-29 57.3084149813886

CCN6 -2.05148987354615 -1.38779045751934 -12.0586641378755 3.69416765724086e-30 2.29017434932438e-29 57.32925627164

RN7SL834P 1.87977952159886 -2.39269377025341 12.058041702569 3.71674132899117e-30 2.30351526552592e-29 57.4677931108143

ALDH4A1 -2.08109583605507 5.55108377185462 -12.0547793129687 3.8373213750096e-30 2.37757255480819e-29 56.6486971485203

ESYT3 -1.83628609042429 -0.0929065777956782 -12.0530980196143 3.90097477458945e-30 2.41632658019e-29 57.1721664417577

MUC1 -2.2977103337335 6.03263870325421 -12.0430437469017 4.30415768701962e-30 2.66379943173152e-29 56.5452227072018

C19orf84 1.90909398467401 -2.85118112653758 12.0414863389032 4.37021521334631e-30 2.70391599822358e-29 57.3093257978313

FAM225A 2.00314633421918 -1.7066150873327 12.0404544891088 4.4145356924453e-30 2.73056463308823e-29 57.292414427824

CDH1 -1.92535776964391 6.56948393990176 -12.0379852791901 4.52241812654695e-30 2.79650264061773e-29 56.4998163533853

APOL6 1.07141299258432 6.62124496471776 12.0347390275441 4.66825095052537e-30 2.88341684629256e-29 56.4502833178394

FARS2.AS1 3.75114777956502 0.354972267635935 12.0297141090257 4.90326580453754e-30 3.02601151162268e-29 57.1725774077705

PLCG1.AS1 1.45371812431456 -1.70192388593252 12.0296365215626 4.90698534404739e-30 3.02745202494262e-29 57.185141692234

PFN2 -1.58273317155995 5.95693073534528 -12.0272573314429 5.02241663602398e-30 3.09779482774575e-29 56.3782807021575

CYGB 1.89865024579754 5.00372026273324 12.0252917848899 5.11981400533386e-30 3.15697786282621e-29 56.5814938945488

IL3RA 1.40441901011209 4.14006740023339 12.0234338185774 5.21360741597231e-30 3.21122488432565e-29 56.6389884393801

SCAMP5 -1.19833435712415 4.68088986828762 -12.0210455248997 5.33668779719635e-30 3.28514474456709e-29 56.3284527917668

ADAM8 2.01135458180906 2.82537895329096 12.0202380869571 5.37894944310605e-30 3.31022760293429e-29 56.8534045508744

LINC02762 2.29323118336467 2.84553353175956 12.0192230320296 5.43255053665859e-30 3.34227271614865e-29 56.8671948220535

ACTG1P25 -1.94754897877078 -2.19448167026666 -12.0187046389793 5.4601295071083e-30 3.35829468389636e-29 56.9989246730537

PRRT2 2.21434129642148 1.18339298315739 12.0177057044188 5.51366712952839e-30 3.39026921163714e-29 56.9811834433869

IL15RA 1.38900728037923 4.02008672606461 12.014809809547 5.67184140027672e-30 3.48654712011947e-29 56.5745008268269

TMCC2 -1.03325064566537 1.66910135447494 -12.0133533323839 5.75309245740649e-30 3.53351122742282e-29 56.6394456681235

RAB44 1.64794541328013 -2.51519493333985 12.0125897686914 5.79615023403263e-30 3.55895675932327e-29 57.0269790258907

ETNK2 -1.69512761131735 4.55351631426915 -12.0106250769443 5.9084197201266e-30 3.62687348718895e-29 56.2256136272274

FCRL3 2.89490878035929 0.485034410521958 12.0097453702026 5.95938800161267e-30 3.65713299632804e-29 56.9665425029452

DRD4 1.8047245071261 -0.765409309068126 12.0096605197114 5.96432712271998e-30 3.65913645740594e-29 56.9803617407996

MARCHF4 2.58619345520068 -0.325187960862446 12.0090700499247 5.99881115174306e-30 3.6782272755062e-29 56.9743064174036

HCST 2.00526996422772 1.91605356679369 12.0083445086626 6.04145520532822e-30 3.70333578126332e-29 56.8270944355022

KCNT1 2.02153234531522 -2.34761362020068 12.0051838248173 6.23077228612229e-30 3.81724325115308e-29 56.9554377081652

ZNF197.AS1 -1.50217098665709 -2.82287790427263 -12.0041436334612 6.29435892503476e-30 3.85511843017894e-29 56.912579760201

VGLL3 -1.86304622926655 2.1837833292755 -12.0029526492176 6.36795659080379e-30 3.89800995458922e-29 56.3626754497725

ICAM1 1.41227787847326 6.32631391962223 11.9996652168389 6.57557865192388e-30 4.02397424476316e-29 56.1331819808305

CDCA7 2.14485255767232 1.22099473576178 11.9953314618427 6.85960673211017e-30 4.19309051515535e-29 56.7586433897052

DENND3.AS1 1.17463353994831 0.33800060597385 11.9941386669107 6.93990056469276e-30 4.24098565548744e-29 56.7582005252367

KIR3DX1 1.98537628357355 -3.25022859086116 11.9925787072705 7.04632298935979e-30 4.30481678184175e-29 56.8368260233608

ANLN 2.3381732091669 2.75192404771191 11.9900390477526 7.22306575106803e-30 4.41040008263118e-29 56.5947813864438

FAM72C 1.95827320797777 -3.71098191901278 11.9900373830217 7.22318303927927e-30 4.41040008263118e-29 56.8129332914209

VAMP5 1.13095409957453 5.46571710911074 11.9856171921585 7.54138398927655e-30 4.60083373304468e-29 56.0453137445376

MICALL2 1.88150262759145 4.29071085797669 11.9854377976881 7.55458863663869e-30 4.60760327871799e-29 56.3095758206247

FAM9C 2.26567001852947 -3.33711120108472 11.9784922320779 8.08390527099753e-30 4.92356694222712e-29 56.7009666300012

PCDHB10 1.673075808199 2.24277751259719 11.9775776615642 8.15629864366309e-30 4.96594747199655e-29 56.4716293458891

GPR55 1.98894514134877 -0.961710938753339 11.9775558390389 8.15803387382401e-30 4.96594747199655e-29 56.6746231513508

LOC729867 1.48873484818807 -2.01750439340415 11.9768406035337 8.2151099483632e-30 4.99790639481016e-29 56.6770731750432

TRIM50 -2.78291545302984 -2.18399754431292 -11.975659060884 8.31026897648628e-30 5.05439209415961e-29 56.5344658673222

GOT1 -1.23123365352072 6.48983039734041 -11.9748509305584 8.37598483844502e-30 5.09294370268612e-29 55.8723270404939

EXOSC5 1.20541392951786 4.08013109067822 11.9746239075114 8.39453882659725e-30 5.10280547921084e-29 56.1496254803247

ACAP1 1.65858914718703 3.59254008826127 11.9685395661489 8.90731862246642e-30 5.4130042785528e-29 56.2250131896828

OAS2 1.30832627952025 5.49436348605128 11.9681558506417 8.94068272350831e-30 5.43025957634316e-29 55.8892004453948

MATN1.AS1 1.41305944637868 0.582129117005589 11.9679866173161 8.95543705731773e-30 5.43770953080201e-29 56.501866932632

EPHB2 -1.42319438072912 2.5180680072621 -11.9577292149836 9.89644137539824e-30 6.00241280534493e-29 55.9207845013287

SEPTIN5 1.71002754815429 3.82991087951481 11.9556239781577 1.01014132415858e-29 6.12493738710316e-29 56.0638319201772

FHDC1 -1.29675712199338 2.02711009408969 -11.9535673030086 1.03057341975233e-29 6.24372634927852e-29 55.974391067339

DOCK6 1.12625480647837 6.35114418036358 11.9449257787416 1.12101225398838e-29 6.77849496026788e-29 55.5893870146546

RNASE6 1.79812366473963 3.90725213385695 11.943468249276 1.13702597600763e-29 6.87342424721843e-29 55.9485730482137

SLC39A14 1.61631659617632 7.78131195521377 11.9428037439804 1.14440215287667e-29 6.91610073197284e-29 55.5525557863228

CYP1A2 -2.75044884104008 -5.03438450660934 -11.9421545675909 1.15165413878822e-29 6.95800328862011e-29 56.3402196539782

MIPOL1 -1.02725332443437 2.39019464777255 -11.9399688230138 1.17640895749775e-29 7.10363773091969e-29 55.8102422798245

ADAP1 -1.15610176246452 2.30823085015152 -11.9375647492013 1.20424812865004e-29 7.2697332473451e-29 55.7906195128435

FAM43B -1.99168712550457 -0.695750106822425 -11.9345286878792 1.24034395133874e-29 7.48349982567792e-29 56.0750018679963

RUFY1.AS1 1.86957854189471 -1.3319956908732 11.9338649125684 1.248377857714e-29 7.52989272001765e-29 56.2566880247938

LINC01428 3.84516199149214 -0.245526323356844 11.9329452875293 1.25959401106015e-29 7.5933539099855e-29 56.243688287523

SLC17A9 2.83840329051585 2.29146165865492 11.9307345672759 1.28696877270474e-29 7.7562406480741e-29 56.0957635991404

LINC00323 -2.63713060106951 -1.68896728604917 -11.9294528890086 1.30310963457927e-29 7.85135231443639e-29 56.0669184892974

NFKBIE 1.18079418163051 4.96397253105339 11.9283175845923 1.31757523577061e-29 7.93632073519712e-29 55.5574954511214

LINC01783 -2.81819895980665 -3.71726672859475 -11.9263205740561 1.34340903339194e-29 8.08747041507277e-29 56.1505211232445

MIR223HG 1.83186450503559 0.0012825316138504 11.9246755308795 1.36506772541129e-29 8.21559487838418e-29 56.1335672600155

MSC 3.14313326902825 4.29787369335444 11.9242465731562 1.37077226694669e-29 8.24765593325607e-29 55.8599879581288

TNKS2.DT -1.61951919325087 -2.25790410613002 -11.9230439703898 1.38689196222363e-29 8.34234793572059e-29 56.0969956553547

TRBV2 2.55336465888051 -2.40109243503217 11.9229968353285 1.38752758668886e-29 8.34387461527564e-29 56.1628695401701

ZDHHC20P1 1.67508755078108 -3.11742238001443 11.9215317892427 1.40742912953981e-29 8.46122387010552e-29 56.1496843393086

EML5 -2.0340235199096 -1.2424151467627 -11.9189914200775 1.44261348697297e-29 8.67036098207378e-29 55.9662803359754

CTBP1.AS 1.82997639565719 0.113532434320981 11.9180996921666 1.45517035227422e-29 8.74342527034606e-29 56.0640953633037

HLA.DRA 1.44793154186126 9.95859274556606 11.918038659311 1.45603375102912e-29 8.74620823563479e-29 55.3561498612977

GCAT -1.30657643914895 2.8182403985734 -11.9175550030981 1.46289381807882e-29 8.7850009910625e-29 55.4944055331587

NXPH4 4.15704380157731 3.2854393438884 11.914972376844 1.50007308029531e-29 9.00332244539783e-29 55.9483830085091

TMEM233 2.02273527152115 2.33539466015795 11.910776840665 1.56248564996324e-29 9.36762658673436e-29 55.8436426028561

LINC00845 -2.42033495376445 -4.68908039029881 -11.9099062233884 1.5757570123567e-29 9.44460175288836e-29 56.0284911144102

SHISA4 -1.07945353282259 2.88147654995712 -11.9094597510876 1.58260630394955e-29 9.48305334801465e-29 55.4339596185276

TYRO3 -1.40990773546872 2.99878247167501 -11.9082481784663 1.60134252096752e-29 9.59006251320999e-29 55.3622164528969

LINC01819 -2.42751112796366 -3.96098681217892 -11.9071097837388 1.61914809113774e-29 9.69403924263922e-29 55.9882086607983

PLVAP 1.70875271219842 9.04774792398795 11.8999578514269 1.7356043906393e-29 1.03799022300604e-28 55.1534986016378

PRPH2 -1.55534219025594 -0.697244989177673 -11.8975285458631 1.77702745173222e-29 1.06189173920438e-28 55.7452509964378

RNY3P16 2.05026086656199 -3.74629202680315 11.8958652296693 1.80595504213623e-29 1.07888287413349e-28 55.9040407511496

SNORD99 2.47544894505182 -2.51068122944711 11.895418084652 1.81381114271261e-29 1.08327999184746e-28 55.8975030570577

SLC22A7 -3.35296863383685 0.0354148652380182 -11.8922130121954 1.87112653705941e-29 1.11690052483909e-28 55.4264070249255

CYP24A1 -3.19235685189015 0.66491676375525 -11.8905846812701 1.90093206175801e-29 1.13438198649912e-28 55.3468815283827

NPIPB3 1.96677469337231 0.316597089310895 11.885241995097 2.00208301792259e-29 1.19376589879024e-28 55.7424824827729

TNFAIP6 6.36693877073821 4.02284410258391 11.8850899298263 2.00503904830646e-29 1.19520235468197e-28 55.7242592411508

TRIM9 2.6936338350568 3.33136332981632 11.8815147143454 2.07580322550694e-29 1.23671013868602e-28 55.5145180216957

MEST -1.77507116899246 3.39766455930564 -11.8812818028823 2.08049849373569e-29 1.23916962615443e-28 55.0396121032153

GPR155 -1.28202867566616 4.28125072567612 -11.8795719455759 2.11529253201365e-29 1.25920696546157e-28 54.9753749744153

PPP1R9A -1.08220914172465 4.19007003938338 -11.8789081109958 2.12895640806824e-29 1.26665081365411e-28 54.9820991699505

PRSS8 -2.14920670518851 4.91502006754323 -11.8778996711817 2.14988155262023e-29 1.27875235627136e-28 54.9353754315112

HOXD3 -1.17464147742273 1.17185990424207 -11.8761305460459 2.18708638357975e-29 1.30052785688077e-28 55.3679979567385

ST14 -1.94548685926191 5.40421461433631 -11.8738330602778 2.23636013724187e-29 1.3291046526828e-28 54.8933551548568

NT5DC3 2.14174168682177 4.82533525210976 11.8729595992682 2.2553814469671e-29 1.33968064040696e-28 55.1683951955356

CABYR 1.38454438848893 0.170825960551314 11.871389908125 2.28996987495921e-29 1.35985629558379e-28 55.5945706520367

KIR3DL2 2.18011800535087 -3.44281905066899 11.8694949605741 2.33242903362098e-29 1.38469360694701e-28 55.6501053386904

ADAM11 1.83331731557611 -0.579667232385175 11.8693928771028 2.33473846751068e-29 1.38568820560866e-28 55.6225566238655

EPGN -2.23205897984242 -4.88554846042574 -11.8664664023109 2.40192013482181e-29 1.42517406207605e-28 55.613501076613

CRYZL2P.SEC16B 1.57264202601571 -0.840862427445683 11.8628209175875 2.48830320326497e-29 1.47562794846538e-28 55.5616594068621

TRPC5 -2.10502200078239 -4.63191870422995 -11.8586993425439 2.58969463613012e-29 1.53409045495667e-28 55.5377399563621

MEF2C 1.23854723651572 6.23253276902375 11.8585212603462 2.59416691741773e-29 1.5363232966485e-28 54.7648177592034

ELMOD1 -2.86374565518915 -1.45296027823263 -11.8544737267774 2.69791243529262e-29 1.59603358008797e-28 55.307429095724

SLC16A11 -1.87324706828675 -0.0238979474575456 -11.8511445982189 2.78632935589686e-29 1.64744738675363e-28 55.2112701955698

DOCK8 1.15961675399698 6.35033822992733 11.8499525499013 2.81868433236481e-29 1.66612682486254e-28 54.6735384679003

GDPD5 1.33861741517169 3.30463434924857 11.8472186351673 2.89430747532321e-29 1.70990271041466e-28 55.0584524335482

CHD3 -1.06442957408962 6.57630126916947 -11.8447329022082 2.9648156268052e-29 1.75061107518438e-28 54.6143623813719

C5orf47 -2.22320906348995 -3.83550841538697 -11.837420194065 3.18230202402178e-29 1.87852096517958e-28 55.3186345117368

SLC6A1 2.19276586134602 2.37378873759959 11.8370835789768 3.19268664554753e-29 1.88414208115447e-28 55.1433464256701

PLP1 -2.47069155577532 -2.82413087034502 -11.8367036557403 3.20444782532178e-29 1.89057230903771e-28 55.2541810083823

RAD51AP1 1.30035820514527 1.50269693674037 11.8366222408309 3.20697376592699e-29 1.89155189492045e-28 55.1564077135321

LHFPL3.AS2 -2.26776142581305 2.17181532328095 -11.8334313105167 3.30754961347516e-29 1.94877006479571e-28 54.6812945333908

INSR 1.25019651228425 8.23273966002645 11.8295428857408 3.43435986102668e-29 2.02184983952091e-28 54.4656169206514

IGFLR1 1.62193331767119 0.738996877461394 11.8291819657152 3.44637272506183e-29 2.02782937966549e-28 55.1646741817155

MYMX -2.20372038506314 -3.71964584401554 -11.8276111290103 3.49914514488647e-29 2.05832621402972e-28 55.220566332579

CPVL -1.62190603967573 6.01275317590275 -11.8275801949404 3.50019240782737e-29 2.05838817783239e-28 54.4493287492201

TMEM92.AS1 2.76891599341582 -2.9586354257039 11.8259628505566 3.55538354939315e-29 2.08859668561528e-28 55.2315963581152

RPS2P29 1.25397443071904 -1.22482081775977 11.8236293431543 3.63654221508601e-29 2.13569892572627e-28 55.1899049465365

MS4A4A 2.1478002716159 3.99143919907231 11.8222862807351 3.68408626503282e-29 2.16303968698985e-28 54.8078187451883

PHLDA3 1.56742432157496 5.39623358935914 11.8209959666178 3.73034522231438e-29 2.1896114462325e-28 54.5111759799896

ANKZF1 1.3315197385636 5.21040107261265 11.8191379878201 3.79797217666946e-29 2.22751170093284e-28 54.487573597724

HLA.DQB1.AS1 2.22859795140748 0.509323143940336 11.8190036682977 3.80290815729024e-29 2.22980820931751e-28 55.1070265320075

PTGDS -2.3489979451165 3.26306473461452 -11.8128017665839 4.03790120346637e-29 2.36632488601101e-28 54.3705579935632

UNC5A 2.96344699447634 -0.180495557938189 11.8104674753743 4.13004216527351e-29 2.41967322889335e-28 55.061184956818

UBTFL6 2.5925229051427 -1.73970678447858 11.8086105650634 4.20483061706939e-29 2.46282936142636e-28 55.0588530459565

IRS1 -1.39573049254745 4.26423501164256 -11.8083026027578 4.21736359995532e-29 2.46950821944865e-28 54.2877748945261

LINC02481 1.30640307796413 -1.40149768425691 11.8074333982698 4.25293795149763e-29 2.48900517016812e-28 55.0381528730375

B2M 1.08594272937914 11.7813028981597 11.806100186991 4.30808376047643e-29 2.52060386660486e-28 54.3404281180797

RTL4 -2.79607438202559 -3.26032296429669 -11.8028829151311 4.44410674365492e-29 2.59879755603669e-28 54.9385755068379

CARD9 1.57059459477486 1.79298435485212 11.8028126461985 4.44712482544648e-29 2.59986674185345e-28 54.8254553295582

SOWAHB -1.80423763074279 2.67126912202474 -11.7995456789939 4.58971629622664e-29 2.68251057024447e-28 54.329738440186

RIN1 1.89687971407506 2.75194034575531 11.7980431794997 4.65681280381647e-29 2.72027078860736e-28 54.7120511525408

PIWIL4 1.31040827253828 1.55908451463381 11.7979739231509 4.65992896843657e-29 2.72136364904448e-28 54.779184746888

LOC101928844 -2.54961358455041 -4.39301161082767 -11.7959113141264 4.75369207811856e-29 2.77537892020104e-28 54.9267801390601

IL27 1.88370122366886 -3.05757019496051 11.7942813779263 4.82911187715999e-29 2.81865870330067e-28 54.927174632226

LOC105375431 2.6555677721829 -3.46868253106832 11.7897971368288 5.04280210990833e-29 2.94024425047563e-28 54.8852629885343

PCSK9 -2.83741190226277 -4.02084695039739 -11.7837579938583 5.34552225306077e-29 3.11508527456366e-28 54.7924985515372

P2RY8 1.82526741364386 4.52245714040264 11.7762990745988 5.74446499257195e-29 3.34578340838686e-28 54.2404683892189

GIMAP2 1.29445906985134 3.65305951927258 11.7741553610851 5.86450540804687e-29 3.41297035105322e-28 54.2913982921897

CDCA8 1.4930745508928 1.62587405256709 11.766335351222 6.32393550794693e-29 3.67740720210655e-28 54.4853368244333

KRTAP5.8 -2.8409765468743 -3.68339742873584 -11.7614311698024 6.63014448893279e-29 3.85444393499995e-28 54.5617942819177

PRR18 -2.68477584542902 -4.42405985356343 -11.7609677439118 6.65983165090957e-29 3.8705790109413e-28 54.5910702571603

NUDT1 1.14934394946911 2.91286862699517 11.7609157916947 6.66316795141236e-29 3.8705790109413e-28 54.2662722404362

UCA1 -3.095865901587 -4.39948000286227 -11.7605737489055 6.68517501028503e-29 3.88230479669834e-28 54.5764400446667

HES4 2.15226430655312 2.59992364268586 11.758262068336 6.83581590731333e-29 3.96767822104962e-28 54.365608416179

SH3PXD2A.AS1 1.96718660680678 -3.05260712686982 11.7516043925903 7.28878940974284e-29 4.2272270427577e-28 54.5190647554684

PEAK3 1.43579249711451 -0.206406425024053 11.7512338134035 7.31486205248006e-29 4.24122261695003e-28 54.4641516615427

DUSP10 1.33517411255872 2.84755801766813 11.7502724173719 7.38293575907761e-29 4.27934363510763e-28 54.1883983457458

SNHG15 1.56502118882171 3.96177692638173 11.7502500629807 7.38452608245589e-29 4.27934363510763e-28 54.0583538919422

TAS2R2P -2.81944949875636 -3.90419851759162 -11.7500362575352 7.39975373088316e-29 4.28703123756601e-28 54.4657561880078

PPM1K.DT -2.10478950071531 -4.0089654491134 -11.7490193481344 7.47260864172278e-29 4.32694532717456e-28 54.4788551332319

BACH2 -1.20107967122165 0.891479191669235 -11.7476402515403 7.57255294082006e-29 4.38365561366201e-28 54.1616093601654

PLD4 2.45836986591397 2.8483627871077 11.74460421961 7.79728623669621e-29 4.51016665247545e-28 54.2313703605026

TRGV7 1.92867024556386 -1.89202345779863 11.7420806840705 7.98912259666571e-29 4.61990727983423e-28 54.4215495099787

CYP2D8P 2.15431837303279 -2.48832081198361 11.7354279520833 8.51765174131217e-29 4.92163520631663e-28 54.3627673357691

STRA8 3.08062104807559 0.334736651491465 11.7343474061816 8.60671962301469e-29 4.97178545920539e-28 54.3250671843604

PHGDH -1.95186216467961 4.52487053449959 -11.7341965500412 8.61922794928774e-29 4.97769525305986e-28 53.5604480901467

DUXAP8 2.64307011890641 0.396102809757004 11.7337909898509 8.65294492180156e-29 4.99584690557806e-28 54.3101466878965

SCGB3A2 3.74832228112019 -2.95390638039098 11.7294287313148 9.02400782366211e-29 5.2059567838038e-28 54.3068506196541

SNHG20 1.12709597122343 2.65929979994779 11.7285465039571 9.10095413552503e-29 5.24896148650379e-28 53.9845275091302

NBEAL1 -1.08021600603325 4.04272874275286 -11.7194009788399 9.93808235959984e-29 5.72120426249566e-28 53.4602248252607

P2RX5 2.05803274230257 -1.33722204673173 11.7178572215763 1.00867569003516e-28 5.8052646442819e-28 54.185435536111

ATF7.NPFF 2.05808749561349 0.191905103063677 11.7156880893321 1.02994042029577e-28 5.9245296142994e-28 54.1273390164958

PLEKHG4B -2.11514528999539 0.0850371141484372 -11.7148828707642 1.0379472157145e-28 5.96744554196502e-28 53.8782291385444

SLC10A6 2.39007890095535 -0.521388017311528 11.7105399382019 1.08221111266041e-28 6.22029443581484e-28 54.106416835107

COL4A2 1.31441761468007 9.81832753544052 11.7073884428608 1.11550237391135e-28 6.40972276119398e-28 53.3306888210204

TRIB3 2.79285909403522 4.94497601220516 11.7073649243598 1.11575460502365e-28 6.40972276119398e-28 53.6569728926735

SYNE1 -1.13501846590668 6.99502148721216 -11.707235134978 1.11714759016058e-28 6.41603844619689e-28 53.305106272844

FADS3 1.25003749404361 4.70965751622721 11.7027987468034 1.16581611579371e-28 6.68731744343422e-28 53.4357672045205

LOC112267871 -2.62115210114376 -2.65041133402064 -11.7027901710736 1.16591220699604e-28 6.68731744343422e-28 53.9580568960457

MET 1.10035031923274 7.96138248864602 11.6981545952008 1.2190238899527e-28 6.98828149715015e-28 53.2037958208169

GPR85 1.97746039743409 1.38541729382483 11.6963916887474 1.23984783076502e-28 7.10579455696512e-28 53.8686406331444

TRAF3IP3 1.91819697073742 2.71282935751704 11.6939625658812 1.26912205266622e-28 7.26785225810139e-28 53.7153757847157

C9orf24 -1.68903991064618 -0.911074229718273 -11.6937096463335 1.27220931037849e-28 7.28362327998457e-28 53.8000418191393

SLC4A3 -2.27792635150487 0.826962846695174 -11.6933829190195 1.27620855979106e-28 7.30460598667207e-28 53.5463268524714

CDK5R1 1.28690148678633 1.61713539235723 11.6919784294594 1.29354284583186e-28 7.40188316574066e-28 53.7576877313048

ACRBP 1.2988641214308 1.79387977206199 11.6900799162416 1.31734713480739e-28 7.53612223480257e-28 53.7254447739724

PGF 3.81106843067229 5.8179830220588 11.6890869721828 1.32997004119998e-28 7.60236340840786e-28 53.477463282587

GPR171 1.89226557868985 0.517750568535794 11.6845693427618 1.38893655681502e-28 7.93735109207078e-28 53.8073377164035

CBARP -1.52545887072965 -0.945680417896583 -11.682406411107 1.4180815806383e-28 8.10178739390557e-28 53.7038320226419

CNTNAP1 1.66100757139428 3.34128159411029 11.6804766515698 1.44459760865095e-28 8.2489656498169e-28 53.4874892469043

TRBV19 2.680348244194 -1.12150557014847 11.6800855668229 1.45003113272753e-28 8.27782924333716e-28 53.8255742244955

ADGRD1 -1.4865563048092 0.789026999565214 -11.67867752326 1.46976259824414e-28 8.38827946185142e-28 53.4932128409783

ALOX12 1.14996637945428 1.02287119531762 11.678156542525 1.47713078646571e-28 8.42813030721546e-28 53.6701943447677

SLAMF7 2.98944119986668 3.41271146992464 11.6780088453471 1.47922633085093e-28 8.43788384444932e-28 53.5803205518002

SIRPB1 2.61717156625996 1.73156676052989 11.6720429361009 1.56638902782748e-28 8.92809061687897e-28 53.6435342669815

PHYH -1.11113028530034 5.68877253512248 -11.669385454292 1.6068416635903e-28 9.15168962352813e-28 52.9264375074106

CLEC5A 2.37820005035836 1.6431523518648 11.6693833105243 1.60687471218951e-28 9.15168962352813e-28 53.6107489011945

KRT23 -2.69769594835987 -2.73990789611892 -11.6668051843412 1.64711267709017e-28 9.37841410433861e-28 53.6142152075232

PLLP -1.18729096803991 4.74320571402236 -11.6650649899587 1.67483752653643e-28 9.53130845505225e-28 52.9002820539885

POLR2F 1.38622328633904 -2.10357964512909 11.6646166304346 1.68205568572965e-28 9.56989401204116e-28 53.6829863772739

TRBV5.1 2.53200302341493 -1.45377819606283 11.6637170558466 1.69663140965955e-28 9.65030874421917e-28 53.6727050007735

MACROD1 -1.15300574918786 3.64512697782528 -11.660496806627 1.74984598761534e-28 9.9478107095104e-28 52.9347727453021

TIFAB 2.57468454857609 -1.46516273015491 11.6559604994122 1.82763571452816e-28 1.03819400232867e-27 53.5991628990228

FLT1 1.74228771980024 8.50406853214624 11.6556235966748 1.83354833991037e-28 1.04128201330721e-27 52.7997029489095

LGR6 -1.37099857158473 -0.350481948627701 -11.6542066576901 1.85862449253246e-28 1.05524866290756e-27 53.3949992009588

ARAP1.AS2 1.98760752406063 -2.37763291352273 11.6535151787644 1.87098541428033e-28 1.06199075995501e-27 53.5814102779007

CCR5 2.91710784824621 3.46127053454661 11.6488160387677 1.95718072439946e-28 1.11062763880295e-27 53.2916606328521

AMZ1 2.73396248604991 -0.306050406419906 11.6470954337218 1.98971950490944e-28 1.12879907426755e-27 53.5020625441554

ADAM20P1 1.7595252143636 -2.10886884600463 11.6444670048155 2.04046787021738e-28 1.15728897918143e-27 53.4929210683203

IL9R 1.98283026666473 -2.24257345112169 11.6443580707645 2.04259866677823e-28 1.15819690360936e-27 53.4935623342262

GPA33 1.84240392542092 -2.30681010731921 11.6427950935798 2.07341592692675e-28 1.175061157965e-27 53.4786811664633

HSD17B3 2.62428971509215 -0.715573275909973 11.6379307960857 2.172315024505e-28 1.23047175299398e-27 53.4199736174627

P2RX3 2.32530447205417 -3.27647074702086 11.6364009131214 2.20437926827956e-28 1.24831044699956e-27 53.4226059688221

MYO15B 1.90377077755384 6.51085131934663 11.6356647639865 2.21997522631193e-28 1.25649102876443e-27 52.6474665588639

PMCH 2.88927135998084 -3.69716240298827 11.6354421903265 2.22471220977835e-28 1.2588460880447e-27 53.4128615319382

TRAV4 2.49228965423821 -2.58755305950046 11.6347836084818 2.23878760764329e-28 1.26648267123554e-27 53.4056825042147

TESMIN -1.14945010360515 1.69557076617208 -11.6290186223306 2.36584065198781e-28 1.33697222052986e-27 52.9281733017338

GLIPR1 1.54400214483613 4.53877454639945 11.6285181096632 2.37720387078468e-28 1.34304643713179e-27 52.7964411320752

SMG1P7 1.77642800152456 0.321738085656184 11.6280082612743 2.38883484184362e-28 1.34926874641532e-27 53.2754170313201

MTCL1 2.09922394781452 4.68919929859322 11.6273762749929 2.40333067317649e-28 1.35710556074744e-27 52.8315132446464

TPRG1 1.86870608313806 0.667541516138783 11.6222661274775 2.52380082733605e-28 1.42439616425038e-27 53.2051735882314

LOC105371485 -2.74085825216621 -4.76586393447984 -11.6207629985942 2.56036622542556e-28 1.44466003419119e-27 53.2608005736135

EIF4EBP1 1.8061738413608 4.85517034335718 11.6116555356016 2.79345720511683e-28 1.57414699080501e-27 52.6207396014137

TEN1.CDK3 1.7557792908913 0.119736451398534 11.6104566395967 2.82567269788586e-28 1.5918903188167e-27 53.1188874449149

WNT4 -2.0971759527198 -1.06223975850847 -11.6100345643926 2.83710197197971e-28 1.59791725241424e-27 52.9947370408975

POU5F1 3.1506747100676 4.14787606916641 11.6046499121747 2.98700970714576e-28 1.68061594053183e-27 52.8183069240874

FBP2 -2.04900583992651 -4.75097741002083 -11.6042739629043 2.99776549589502e-28 1.68623344612082e-27 53.1104040351128

TAFAZZIN 1.03883475454005 4.38853007699583 11.6018168142542 3.06901841706899e-28 1.72586874596523e-27 52.5009715192703

KCNE4 2.02865682048983 4.67361243364842 11.5982310163772 3.17603267753339e-28 1.78512968369695e-27 52.5531970316641

EN1 2.82943878994285 -2.74860474161146 11.5976609621673 3.19338384577983e-28 1.79442060122979e-27 53.0544671408564

LINC02499 -2.98225095721138 -4.55209679799244 -11.5970303259687 3.21268882992218e-28 1.80480434448045e-27 53.0252479574955

LOC105371855 -2.28685171802252 -4.08480569222389 -11.5921425461639 3.36630172868692e-28 1.89012825480461e-27 52.9844750049379

MPHOSPH10P1 -2.10342473252068 -3.37845265815704 -11.5870724821109 3.53336317074747e-28 1.982402705272e-27 52.9174457283078

PAQR9.AS1 2.67243572978293 -3.51013680831513 11.5869595512978 3.53717667486559e-28 1.98403290235723e-27 52.9536828381522

ZNF516.AS1 -1.11149273607515 -1.20343946842658 -11.5833782126138 3.66025937095392e-28 2.0504395804526e-27 52.8083367478442

CD27 3.21666541638462 2.02557692342238 11.580896708206 3.74803180598157e-28 2.09799536516689e-27 52.789782568245

ZDHHC23 -1.22439211053102 2.86968779976633 -11.5797884755857 3.7879037596457e-28 2.11977108475128e-27 52.2612368051389

C12orf75.AS1 2.83371132881713 -0.613613512285694 11.5787945469552 3.82402190095494e-28 2.1394354992721e-27 52.858840607467

SERTM1 -2.59084187304538 -4.55976133261862 -11.577269588253 3.88010387167162e-28 2.17025620444433e-27 52.8475240838293

LPCAT2 -1.07690638156937 4.69278533535889 -11.5762436780814 3.91829221718056e-28 2.19105526668492e-27 52.0589262865955

MARCHF11 -2.07909860788903 -5.97302466482046 -11.5704093499662 4.14268337990923e-28 2.31534680054108e-27 52.7952394087948

KCNQ5 1.94261364979535 -0.85071770103574 11.5693268564007 4.1857004490077e-28 2.33759549992756e-27 52.7668607436438

MIR621 -1.28808495485729 -0.648604818585523 -11.5633672446683 4.43060582558898e-28 2.47247265338601e-27 52.5647880434404

PSMB10 1.29059558894355 4.74840809737619 11.5629475636732 4.44837976165861e-28 2.48112411770101e-27 52.1056292728184

TRGC2 2.26546466946906 0.406261934234374 11.5619105887884 4.49260135176871e-28 2.50514971523862e-27 52.6633080096881

H2BC20P 1.46288851166122 2.5383663724751 11.5613144123766 4.51822278880288e-28 2.51815155837055e-27 52.4393069929604

CRH -2.02435304761185 -5.8446229442101 -11.5602089338745 4.56611722628114e-28 2.54354727876426e-27 52.6985104746763

FBXL22 -1.07735505644046 -0.186614795317556 -11.5502448956972 5.0212343374537e-28 2.79350901161853e-27 52.4168013225029

CDHR3 1.56830752964262 2.73212896076209 11.5485664085618 5.10222158586198e-28 2.8378429197211e-27 52.3012114098218

ZNF45.AS1 -1.87125945570553 -4.16038288033746 -11.5451227548962 5.2724666378283e-28 2.93104078953096e-27 52.5466954490398

CXCL11 3.21091102839505 1.81507442608276 11.5447035575472 5.29357253246967e-28 2.94202544639012e-27 52.460547663031

TLR10 2.24213218391978 0.199856807273215 11.5382222874661 5.6307905481996e-28 3.12864647469631e-27 52.4481257571608

FREM2 -2.40708253519138 4.0150225622411 -11.5345205199365 5.83288804925221e-28 3.23846805234524e-27 51.6742371216779

TVP23A 1.20714990855125 0.508164117306342 11.5274638060256 6.23833745727925e-28 3.46181788862172e-27 52.2841675266625

PDZRN3 -1.63689888318458 2.91627226194995 -11.5272801962913 6.24925274444958e-28 3.46699467439596e-27 51.7162996011746

ZNF750 -1.68815498005526 -1.67771148814188 -11.5270350405807 6.26385644558035e-28 3.47421459150425e-27 52.2762094522652

ZNF300P1 -1.94972718162035 0.337532072035063 -11.5258342212268 6.33587978472669e-28 3.5132702597217e-27 52.0553409127004

STMN3 1.99398579855308 5.5428127010135 11.5249884554503 6.38710117651398e-28 3.54077427727955e-27 51.7115129442483

ELAVL4 1.76934335394431 -2.37651062022861 11.5231263339041 6.50133101645291e-28 3.60318505459156e-27 52.345796289096

LOC105369519 2.38791612636297 -2.67002791584741 11.5194518682598 6.73272954727052e-28 3.72953963996205e-27 52.3141676019152

NLRP3 1.61306864023258 2.27111182065546 11.5183790107454 6.80182409479065e-28 3.76590478701444e-27 52.0711598584177

CCR8 2.63095306680861 -2.89211351615894 11.5174122669636 6.86468831425469e-28 3.79878535658161e-27 52.2958968425836

HCN4 -2.25414068033215 -2.47096485705796 -11.5140674653782 7.08668022017955e-28 3.91964623770143e-27 52.1741058086881

NEBL -1.82675326641015 5.73103837469045 -11.5098485805485 7.37689011547371e-28 4.07912904082609e-27 51.4190561071141

SEMA6B 1.64545761150917 4.70728832603454 11.5095404386008 7.39854394017319e-28 4.09006781494067e-27 51.6585007821246

LINC00887 6.10400651638972 3.92668697755503 11.5092391421917 7.41977781903195e-28 4.1007689600229e-27 52.1376799093124

PROSER2 -1.2541030751545 3.04931891016597 -11.5088080832094 7.45026211918774e-28 4.11657593149456e-27 51.5646383313594

C21orf58 1.1802454492294 1.03641501701307 11.5084356181696 7.47670294936339e-28 4.13014129303433e-27 52.0628853541776

UBAP1L 2.05216784473327 1.84994380944831 11.5070011262377 7.57941027031044e-28 4.18581886876659e-27 52.033714700092

KRT8P33 1.09635285617124 -0.935040702630887 11.5023543137392 7.92184404834088e-28 4.37272184865858e-27 52.1225512953596

C6orf132 -1.63693538470065 2.48395002888797 -11.5023246312118 7.92408013388036e-28 4.37285159509312e-27 51.5531232594416

KCNIP2 1.83624446442749 0.227831166348645 11.5020137984665 7.94753394805235e-28 4.3846871842158e-27 52.0901081320256

CACNG8 1.93606648450107 -0.866634193591677 11.5002950245976 8.07847517019974e-28 4.45580307658695e-27 52.1151194485899

CD3E 2.9633779152389 4.04340657959896 11.4997594657482 8.11971225306195e-28 4.47741791234325e-27 51.8175411785938

LINC01033 2.36309393490351 -3.43145274398487 11.4982744365305 8.23515486593059e-28 4.53993035532748e-27 52.1160844981081

BEX1 -3.1653598980898 -0.92363448282909 -11.4980523610689 8.25255818050197e-28 4.54837714800781e-27 51.8275600153459

DLX4 2.44446917943788 -1.00045456089194 11.4961927970414 8.39972849001586e-28 4.62832240777399e-27 52.0815666887513

TRAV19 2.52398931486084 -2.03267427646455 11.4940186398183 8.5751083976133e-28 4.72257670899807e-27 52.0712005659877

CDH6 2.09512392654081 7.6153465065012 11.4909710896812 8.82708568426516e-28 4.85889933144198e-27 51.2384968636476

RAB29 -1.08867685628356 5.48340092549963 -11.4902703184556 8.88605998741161e-28 4.89013016632853e-27 51.2273039033338

PLD5 -2.49589100723793 -2.30352973329354 -11.4901058650023 8.89995643555066e-28 4.89654451123083e-27 51.923202301334

C19orf67 3.46264888805779 -2.00421162537854 11.4891337200212 8.9825459634629e-28 4.93949614845382e-27 52.0266476425898

TLR2 1.57259408827574 4.42406704392976 11.4862585480487 9.23129504974551e-28 5.07372964592778e-27 51.4680055412332

MAPK8IP1 -1.07317714918266 3.74203728194364 -11.4842096426241 9.41272648333985e-28 5.17084745702428e-27 51.2556623369526

MCUB 1.57618691450531 3.07950189394884 11.4841354089049 9.41936604378078e-28 5.17319442459767e-27 51.6555933468485

SPATA17 -1.20348664659254 1.04711788008611 -11.478986789319 9.89140903476944e-28 5.42971518806372e-27 51.5907545452346

STING1 1.0628528892113 5.89713942450022 11.468546023238 1.09220656838203e-27 5.99096138023407e-27 51.0582596399178

LRATD2 -1.10856507535504 6.16345092477778 -11.4681947344095 1.0958540019098e-27 6.00945985037263e-27 51.0208841412686

MCIDAS -2.51349146077794 -4.99360916965534 -11.466241618933 1.11635515885615e-27 6.12034854151618e-27 51.805743666851

CSF2RB 1.45464591183075 3.68379749825527 11.464374627558 1.13630863529875e-27 6.22817973593771e-27 51.3611773531237

CLMAT3 1.74814489110291 -2.72302409645373 11.4559273397005 1.23111315789103e-27 6.74104631405481e-27 51.7145825621094

OR51E2 2.55412571076905 0.833374432969173 11.4549240219623 1.24288352906446e-27 6.80379102220585e-27 51.6423202796937

ITGA4 1.78311561347777 4.49894129893452 11.4532019393337 1.26334744613006e-27 6.91408257958431e-27 51.1650154719357

TRBV20.1 2.55744813858208 -0.751043125338084 11.4501532143188 1.30040100001868e-27 7.11508839594595e-27 51.6456232867081

PHACTR1 -1.04746949966259 3.91243781756337 -11.4444777439294 1.37228077704586e-27 7.50461757276855e-27 50.8671414312065

TMEM121B 1.49633643230285 -0.142080300208242 11.4432729536952 1.38804042621461e-27 7.58890353616907e-27 51.5440376305427

LOC105274304 -1.37551495835498 -2.16743654110735 -11.4380263834049 1.45879450306769e-27 7.96776712710278e-27 51.4848821807709

NPM2 -1.73536655019955 0.131740708348809 -11.436573010123 1.47902086914216e-27 8.0762226520149e-27 51.2597258197942

KRBOX1 -1.95871881325797 -2.33307476970784 -11.4362911776474 1.48297524156338e-27 8.09579264398813e-27 51.4502628335026

CDK3 2.02167275245502 -1.52797106133961 11.430438620647 1.56750939729706e-27 8.55300445920913e-27 51.4670454061187

TRBV7.9 2.65358934059063 -1.39632339912142 11.4302893848463 1.56972645620673e-27 8.56296361644674e-27 51.4666322628007

CHRM1 -2.41935557811204 -4.064802606447 -11.4282397121881 1.60049387848411e-27 8.72862309121869e-27 51.4340259975863

SLC11A1 2.05776150538748 3.03292688251307 11.4256269857841 1.64058389184432e-27 8.9437125235381e-27 51.1561741723179

NPIPB12 1.92785125310947 -2.23325747177908 11.4207039711697 1.71885545582085e-27 9.36244971985371e-27 51.3815328334129

PRG2 2.04483149250432 -4.10218035321093 11.4176893491257 1.7686065254272e-27 9.62624119555679e-27 51.3584758302109

HS3ST5 -2.54025654501447 -4.74807511696813 -11.4136656225869 1.83724843408851e-27 9.99238178948137e-27 51.3093101712807

TAFA1 -1.55853569299184 -2.89693886892531 -11.4114288966844 1.87654405595388e-27 1.02035623923265e-26 51.2614084293629

EPHX2 -1.45884260709794 5.87765629715491 -11.4018588100939 2.05432971814114e-27 1.11647021463661e-26 50.3976592855603

CXCL13 4.56084583868898 0.279397826996494 11.398858057267 2.11345088181713e-27 1.14831531875559e-26 51.1606138404555

XRCC3 1.35774901253137 2.46019473301637 11.3987870128979 2.1148709125933e-27 1.14880124416857e-26 50.906661119189

KIF18A 1.47792294724487 0.916353865380406 11.398177333118 2.12709621970206e-27 1.15515491275222e-26 51.0522432350625

OPRD1 1.56554500393387 -1.25889729687691 11.3962433224351 2.16634385136316e-27 1.17588455498855e-26 51.1405973788308

LINC01356 1.65155158071562 -2.45602086020962 11.3950740010877 2.19042169399144e-27 1.18865868584045e-26 51.1417299995823

GYPC 1.0071672922208 5.74544264911122 11.3917883055322 2.25951185905337e-27 1.22554263231306e-26 50.3439024107377

HDAC10 1.40828375903802 2.68425176322933 11.3915011313395 2.26565240158684e-27 1.22826350612447e-26 50.816298825216

TSPAN18 1.53225521226109 6.48658902458273 11.3893491867655 2.31219767097141e-27 1.25287517242098e-26 50.301342141189

IL6R 1.2925387653911 5.41052549614322 11.3772870987467 2.59124685565555e-27 1.40164647367923e-26 50.2553878692845

LINC02613 -2.05000049346061 -3.07116891374242 -11.3764366774979 2.61213926338762e-27 1.41224837513136e-26 50.9223452258951

LBX2.AS1 1.48587464160961 3.64853383843378 11.3713852088926 2.73973634557125e-27 1.48086713726857e-26 50.4955116219627

SMTNL1 2.09120571214903 -1.84286945912129 11.371063885934 2.74805922895128e-27 1.48499847503146e-26 50.9138699470494

SMCO3 -1.92869733670047 -0.641484714154924 -11.3695996063765 2.78630626618846e-27 1.50529421100164e-26 50.6919725067276

G6PC1 -3.3161398231298 1.02988937932222 -11.3677299163645 2.83591277547799e-27 1.53095854452867e-26 50.3059965975303

SCAT1 2.24919335816888 -3.75933653269353 11.362648850377 2.97520267998558e-27 1.60536059668457e-26 50.8429102875985

CCDC8 -2.05503201940476 3.27126762945272 -11.3576658817065 3.11840476647682e-27 1.68179909580005e-26 50.0604231464748

MLF1 -1.10979951584873 3.47454601200374 -11.3573328155616 3.12821722109715e-27 1.68667483179462e-26 50.092622661635

BLOC1S5.TXNDC5 -1.60232920230927 -0.900492740397478 -11.3552800048502 3.18937622579582e-27 1.71922641002259e-26 50.6116758335319

LOC101927495 -1.95249028507793 -5.79121637070264 -11.3485303576361 3.39896961926219e-27 1.83130382371146e-26 50.7085989520613

VN1R85P -2.67620057643103 -4.59057099312313 -11.346066952697 3.47882802520663e-27 1.87386810043974e-26 50.6722795427081

ATG16L2 2.11335026293206 4.19217085836704 11.3454201335917 3.50010391171548e-27 1.88486374526167e-26 50.2524791022725

EGFR 1.30153454025335 8.00985467445041 11.3405414003619 3.66480301638616e-27 1.97209899820455e-26 49.8203834922583

PRC1.AS1 1.60487058467732 -3.00454838218456 11.3364671876886 3.80822924869312e-27 2.04827058261607e-26 50.5960362798602

C10orf95 -1.30266961002474 -1.83309901108461 -11.3338596683169 3.9029370882619e-27 2.09869301648099e-26 50.4932591472645

CRYBG3 -1.21264830105409 3.8324787203176 -11.3337728326903 3.90613101183005e-27 2.09989375157496e-26 49.8224891401397

CEACAM1 -1.35720607314284 4.31533965568069 -11.3301746499744 4.0407861401967e-27 2.17174863555628e-26 49.7511181292393

SYDE2 -1.1348115284224 2.16555698916527 -11.3287714829317 4.09453876468157e-27 2.19847556817166e-26 50.0153004043163

LOC100288123 1.92897738982717 0.205868083284243 11.3282715543394 4.11386130592254e-27 2.2083078142551e-26 50.464419700008

CNTNAP4 -1.90641712097404 -5.58002177987837 -11.3257444762056 4.21293025920334e-27 2.26037723924308e-26 50.4951957762831

C2orf92 1.76223520988903 0.886441498839146 11.3133899247284 4.73246917773771e-27 2.53663598089532e-26 50.2767454337833

PTCH2 1.4267212712304 0.823541910078559 11.3115640279209 4.81447360909552e-27 2.5799581113184e-26 50.2450460027202

CD36 2.23390631123218 6.47915078591854 11.3096362637691 4.90258630993534e-27 2.62653146925759e-26 49.6046348842457

MTMR11 1.60065030211417 5.40577876873988 11.309376482604 4.91458209331277e-27 2.63231280600892e-26 49.6543185370007

VPS13B.DT -1.04191024872512 -0.753024316514657 -11.3056498502315 5.0899109569432e-27 2.72421807842468e-26 50.1676071684483

CNTFR -2.76202191739381 -1.79265484824049 -11.3025897860088 5.23851757433649e-27 2.80306867169381e-26 50.1108853995528

EYA2 -1.54262711728507 1.35440935155666 -11.3001050056371 5.36235312565531e-27 2.8679271379086e-26 49.8259253494499

MIR924HG 2.25802669110649 -1.72630642493209 11.3000130087474 5.36699347244153e-27 2.86970659048849e-26 50.2498445222951

WTAPP1 -2.38442986638396 -4.17305689859693 -11.2949277168363 5.62980236954514e-27 3.0087569376784e-26 50.1910554321228

IL18RAP 1.60242621169483 0.399795560816249 11.2913938244577 5.81992943389427e-27 3.10960679508292e-26 50.0956733504129

RPLP0P2 -1.91562747105086 -1.69327136024642 -11.286325424448 6.10380698601551e-27 3.25968949328927e-26 50.0097187185641

KNTC1 1.08207663244162 3.82503004016009 11.286290315313 6.10582066336481e-27 3.25996821296143e-26 49.6276528456912

FAIM2 -1.7394000726696 -0.29320359726583 -11.2855650403302 6.1475666951383e-27 3.28145517803755e-26 49.8928716771342

NRM 1.05147498939615 4.08903208967914 11.2810020378701 6.41679317328729e-27 3.42265514317421e-26 49.5332301909775

TDRD6 1.73686237204489 0.700113929407163 11.2804103682572 6.45255045576268e-27 3.44088787481166e-26 49.9795233909637

PCAT18 -2.18668538224998 -3.63398095631644 -11.2784338786713 6.5734407100234e-27 3.50449865421179e-26 50.0261310051225

RN7SL124P 2.31901319144585 -4.5934369818552 11.2742651671254 6.83584754945928e-27 3.6435067438618e-26 50.0171158702879

FCGR2B 2.32136959803533 2.64057640172574 11.2727429392889 6.93424007671724e-27 3.69414793750614e-26 49.78546217624

CDC37L1.DT -1.07887420981078 -0.851965848075747 -11.2689968585326 7.18241192641466e-27 3.8254264642442e-26 49.8314398280792

GLIPR2 1.13946672688834 4.20737161854161 11.2668880344892 7.32598134682288e-27 3.90094226052925e-26 49.391013179079

KIF2C 1.4971339295584 1.82634122653703 11.2649245014312 7.46222232087489e-27 3.97251996048914e-26 49.7335470681074

ALOX5 2.32052046192014 4.74167057914307 11.2643012939749 7.50598822037374e-27 3.99387291404498e-26 49.436446888413

CDCA7L 1.64070197615753 3.88391117116541 11.2614490661887 7.70956956678312e-27 4.10020013976421e-26 49.4599364997454

IL4I1 2.2095353660373 3.3463404532622 11.2596492502374 7.84084830970952e-27 4.1690038470093e-26 49.5812024583896

ASCL4 -2.36369999539301 -4.95934207903853 -11.2583311880794 7.9383950402084e-27 4.21984302636035e-26 49.8619049126881

KCNJ14 1.20906098949899 0.397488519384364 11.2581651123866 7.95077117424463e-27 4.22513636682013e-26 49.7674985497586

FGF7 -2.44128698005878 0.690551790243155 -11.2581456903575 7.9522197761091e-27 4.22513636682013e-26 49.4360665467379

MGC27382 -1.76918872349715 -1.85664256312845 -11.2563574914428 8.08672364231388e-27 4.29555594933119e-26 49.7483978494725

SPTA1 2.05336315105361 -2.02282574094031 11.2547376060558 8.2105181659356e-27 4.36025403354048e-26 49.8303615135503

DLK2 1.92323955414683 0.295515622563179 11.2545215270474 8.22717306551478e-27 4.36803724491483e-26 49.7729723292475

MYH15 2.1253886292125 -0.352472818383925 11.2542591805666 8.24743930192595e-27 4.37773356971066e-26 49.8031700490259

WNT10A -1.82839716756034 -0.0529487748968274 -11.252409932928 8.3917079997743e-27 4.45322959978309e-26 49.5531775661859

RFTN1 1.00842090234348 5.85697421419361 11.252039807036 8.42088315577237e-27 4.46762708431885e-26 49.0281223379511

LRRC25 1.99750983002109 3.51849420143105 11.2514448734873 8.46799017454128e-27 4.49043895375517e-26 49.4602765348079

CXCL9 3.48665901956985 5.06473076868915 11.2474658944648 8.78985164265284e-27 4.65998612195275e-26 49.373700190228

ARHGAP27P1.BPTFP1.KPNA2P3 1.84942216186408 1.31047267321345 11.2460544893335 8.90691853777975e-27 4.72090445806696e-26 49.6225744627737

CDCA3 1.50243284257657 1.43226364368179 11.2442590351532 9.05808187563199e-27 4.79869729038026e-26 49.5763045623033

RRN3P2 1.42771306115155 -0.30176038138074 11.2367526678333 9.71816703299462e-27 5.13966854240153e-26 49.6198653954952

NCOA7 -1.16407800813507 6.07286854538201 -11.2343928802494 9.93539459956938e-27 5.25074191497919e-26 48.8300554626959

SLC27A6 -1.81252085023667 -5.77037339492948 -11.2297004974085 1.03817885440449e-26 5.48267822747738e-26 49.602307331249

RTP2 2.93427284811064 -3.4520657487254 11.2246363350962 1.08859363007866e-26 5.74753239867334e-26 49.5565898577396

IRF7 1.62227221624354 4.32734824403814 11.2218364664455 1.11750380245137e-26 5.89874652052413e-26 49.0194362174784

WDFY4 2.04825816773565 3.45288422537676 11.2196815478053 1.1402730666021e-26 6.01549909310278e-26 49.1711290652592

DHRS9 2.3148395131581 0.0532466545844558 11.2196651467895 1.14044811891845e-26 6.01549909310278e-26 49.4728968100853

ZG16 -1.66172913949024 -5.83633216421427 -11.2191789644644 1.14564941843204e-26 6.04147604753752e-26 49.5053412925909

SNORD63B 1.95498153279859 -2.4016483202854 11.2172128213483 1.16692526934356e-26 6.15218767453918e-26 49.4842725547081

NPY1R -1.51052773875353 4.40810228873058 -11.216761385819 1.17186547223555e-26 6.17674292444851e-26 48.6862176725793

RIMS3 -1.11642752784933 1.66791730471889 -11.2146653958655 1.19507624413215e-26 6.29452908243429e-26 49.0393813563616

LY6G5B 2.18903111446431 0.617140930531826 11.2117944961931 1.22761152005906e-26 6.46277873954485e-26 49.3675686628244

PLXNC1 1.56015628071159 4.55781726954354 11.2113981156606 1.2321722395011e-26 6.4852263848308e-26 48.8624095736587

RND2 -1.34964497020904 0.79013925227013 -11.209978536806 1.2486443737931e-26 6.570340838069e-26 49.0996940986728

FAM193B 1.87342727072581 4.98370640449619 11.208489851627 1.26615351768323e-26 6.65926662381027e-26 48.8153873227861

SELE -1.76252349466773 1.63716313763798 -11.2073981668311 1.27914823116374e-26 6.72599285265186e-26 48.8996162003136

PYCARD 1.85544394250752 3.43032520221786 11.2061529605828 1.29413225434178e-26 6.80150845457697e-26 49.040720491865

CAPN6 -2.90548473003561 2.32032907318992 -11.2058295844526 1.29805200954691e-26 6.82046899846806e-26 48.6946509727791

RGN -1.33618497512253 3.75032706007796 -11.2056823518546 1.29984057442793e-26 6.8282250175417e-26 48.6307196459576

CYSLTR1 1.48395772463716 2.10178014905718 11.2050712642869 1.30729021411711e-26 6.86472058028362e-26 49.1423647006277

FAT2 -1.57662822676579 0.0672868408697582 -11.2050609564291 1.30741623828035e-26 6.86472058028362e-26 49.1219393511101

LSMEM1 1.65882693019294 0.530222569202993 11.2038781301944 1.32195798463078e-26 6.93773963451884e-26 49.2765241026902

RN7SL541P 2.354615818808 -3.79111693448997 11.2001573415833 1.36875797514827e-26 7.17817807317379e-26 49.3301962691461

PRR16 1.67089758368855 1.22779719964004 11.2000843892551 1.36969183882978e-26 7.18135214825986e-26 49.1905736530728

TOX2 1.70642275897062 2.77559000000025 11.1970483870933 1.40912243571441e-26 7.38631628392107e-26 49.0146430389773

TBL1Y -3.21901544362463 -3.91659853764381 -11.1929337081108 1.46436887413191e-26 7.66854852777489e-26 49.2054018102222

CBLN3 1.50781516039047 2.71991737233455 11.1922778718458 1.47337131021791e-26 7.71384361336653e-26 48.9634328038393

PPL -1.4309971712615 5.04541188702274 -11.1920724582013 1.47620224758081e-26 7.72681382428348e-26 48.4397523695356

WNT11 -2.06833164819213 -1.0915950530528 -11.1841213133819 1.5900374052221e-26 8.31667961137352e-26 49.0036828380354

KIAA0895L 1.88825792146453 4.25530823111829 11.1824355814563 1.61527044125461e-26 8.44663913681189e-26 48.6943695225173

RGCC 1.54868359845347 5.95306226478176 11.182033343598 1.62134994861849e-26 8.47640201606693e-26 48.4078530772292

SH3BP4 -1.01695935911782 6.19925733134684 -11.1813301387471 1.63203301413029e-26 8.53021225969606e-26 48.3366004045132

ZNF337 1.20088221313867 1.65145079768264 11.1764097971658 1.70876552668785e-26 8.92913750710418e-26 48.9040517607041

KIRREL2 -2.53735944477538 -3.33957472383135 -11.1748404942752 1.73398487330886e-26 9.05658973145758e-26 49.0348929498528

VKORC1 1.04336782367165 5.67725598464894 11.1705853383022 1.80424369230171e-26 9.42129926113961e-26 48.2879905425539

TTC36.AS1 -1.19264394061099 -1.35378720017468 -11.1704814468595 1.80599401315158e-26 9.42657118983508e-26 48.9473856537766

CREB5 1.53500170047171 5.01857602894797 11.1704742082871 1.80611602854709e-26 9.42657118983508e-26 48.413609819023

CYCS -1.04010483609276 6.59342633262792 -11.1655497516962 1.89105170728844e-26 9.8580994654996e-26 48.1960069921797

ARL4A 1.04334640538129 3.92694038437443 11.1616370694606 1.9613568887292e-26 1.02197262969479e-25 48.4480949589101
[truncated: 385,064 more chars]
