# Supplementary material for: DNER drives glycolytic reprogramming in renal cell carcinoma by activating the JAK2/STAT3 signaling pathway
Source: Front Immunol. 2026 May 22;17:1799104. doi: 10.3389/fimmu.2026.1799104 (PMC13236898; doi:10.3389/fimmu.2026.1799104)
Supplement: Supplementary file 5 [file Table3.docx]

logFC AveExpr t P.Value adj.P.Val B

TMEM255A -3.0922972605362 1.73173325702125 -14.38321117704 7.33443296622791e-40 1.59648602375883e-35 79.6109722771881

TMEM86A -1.32996457495421 3.51804630674156 -13.0201965190504 7.75380061631449e-34 8.43884890076587e-30 66.0067980333891

PDE6G -1.74757450803068 -0.106372026374142 -12.9277204860386 1.93933472827445e-33 1.05533747575875e-29 64.3064798715844

CEBPA -1.74734341237397 3.0055099385167 -12.3602557728603 4.9919093690063e-31 1.810981520586e-27 59.6309128147576

SLC29A3 -1.18445839078978 3.23978499061671 -12.0682950889547 8.23683603695434e-30 2.56130300023407e-26 56.8616469536384

APOC1P1 -2.46004386497519 -2.67590624751001 -11.8725472824569 5.28389685936759e-29 1.43768228672318e-25 53.8463843541415

SCPEP1 -1.04334908276033 5.76769592627467 -11.6316182322308 5.08299379106204e-28 1.22935028722275e-24 52.8365148521464

OTOA -1.72027625491615 -0.627872176515035 -11.6064558050113 6.42878984405142e-28 1.39935468535467e-24 51.8876893144813

GLB1 -1.08460576666099 5.65357117195517 -11.5466519516474 1.12217409747223e-27 2.22057850724346e-24 52.0560576756544

VEGFA 2.10834529976557 10.4186186616388 11.4132494772079 3.86440892969019e-27 6.47050685942817e-24 50.8375955641661

PKD2L1 -2.39191725405035 -1.02163991203831 -11.218013066306 2.32451305086984e-26 3.61411968416313e-23 48.4773166535355

SDS -2.02045747414576 3.17660586932005 -11.1884161081645 3.04619881474712e-26 4.42044064004004e-23 48.8034961425651

COQ2 -1.04131303334772 2.58033779513299 -11.1308788915863 5.14636926333827e-26 7.00131373469276e-23 48.2289517486436

APOE -2.38277780058251 8.42599738031789 -10.9896508789229 1.85128417391247e-25 2.37040603609134e-22 47.0272219622425

CHIT1 -3.27472378461679 1.43104075498101 -10.7699200790313 1.32974923835431e-24 1.52340271953991e-21 45.0793537750459

GPNMB -2.7235493639665 7.32108055903569 -10.6476926070497 3.93922613278032e-24 3.90983787895654e-21 44.0229276664635

PDE3B -1.57966455489281 2.08543184724998 -10.6473357766777 3.95168986709441e-24 3.90983787895654e-21 43.9776020452648

HS3ST2 -2.45180444792711 1.362522355567 -10.6373778807523 4.31575952888192e-24 4.08439728979012e-21 43.8846918109577

PSAP -1.04461258676118 10.8814185278592 -10.3400937366115 5.85002402199305e-23 5.09349891546891e-20 41.3718676459622

LINC01857 -2.20354661962892 -1.64699514940346 -10.2330395138176 1.4782834209841e-22 1.19177019350226e-19 39.906141020759

LY96 -1.3605680432812 2.58932661693089 -10.1393642862109 3.30975690458256e-22 2.57298137650174e-19 39.6630258132908

HTRA4 -2.14098101163617 -0.32702909496377 -10.0785709861297 5.56968611133083e-22 4.04117858617794e-19 38.8804950322862

PLA2G7 -1.82587504040619 2.49930944529529 -10.049744015508 7.12357506009405e-22 5.00189865590539e-19 38.9191036438384

LGMN -1.31522971361602 7.21618899020417 -9.94943933949594 1.67094825162506e-21 1.13661033103508e-18 38.0704546264716

EHD2 1.37777573464771 8.23871147894127 9.9444227567342 1.74347727235164e-21 1.15000817537206e-18 38.0212657450598

SIGLEC7 -1.49989977756436 1.31168457978975 -9.88265206128039 2.9385095225746e-21 1.82750105079661e-18 37.4399698534799

APOC1 -2.37972376385976 5.88812440438281 -9.81925668332213 5.00973910049805e-21 3.02908308334836e-18 36.9889753669014

CCL18 -2.95599319095345 1.8445518323494 -9.76159665148102 8.12212526824237e-21 4.77822434361707e-18 36.5353921831548

PFKFB2 -1.40140622552585 3.77454373097911 -9.74937149988441 8.99613541047855e-21 5.02099690974068e-18 36.4228578705017

FLT1 1.81890194375706 8.73228448635632 9.73978810029588 9.74595610271257e-21 5.30350566219361e-18 36.3299182318555

FTL -1.07290248297532 11.4127199290614 -9.73586587829813 1.00704192061261e-20 5.34641011853041e-18 36.3248996056848

ME1 -1.33443083476665 3.3096382737868 -9.69461991826858 1.42030573481971e-20 7.36090355471921e-18 35.9841404560243

CFAP74 1.82646264696721 -0.217000068493485 9.53135685477802 5.48580030645621e-20 2.71385034705983e-17 33.9263156778312

CDH17 -2.5610632642712 -1.53089244013476 -9.50933721648849 6.57457299329772e-20 3.18019400766914e-17 34.1570653614014

GPR150 -2.03810447804666 -2.48381683048555 -9.45711416348874 1.00889019806276e-19 4.77402455244178e-17 33.509655494099

NPL -1.54323526876574 4.72121411171979 -9.44802760635188 1.0867524911268e-19 5.03305137752279e-17 33.9604453640343

CLIP4 -1.45749131141639 3.43370883400627 -9.42246134470576 1.33924043628157e-19 6.07317637011269e-17 33.7798598396907

PDPN -2.19758836618653 0.628442547431356 -9.40538169968599 1.53948268249068e-19 6.70198390995492e-17 33.5868821670754

ZNF395 1.40233797900873 8.2019666166716 9.35765977174885 2.27016831639371e-19 9.50283725825806e-17 33.2352749719213

CRYBB1 -1.29045378560421 -0.712949624517828 -9.31466027416664 3.21763845858737e-19 1.32147804392587e-16 32.5925553528467

NOTCH4 1.57075313935994 6.70177106789596 9.3110916578971 3.31197421297085e-19 1.33503227210623e-16 32.8744682331739

CD180 -1.38221110298316 2.51909441128591 -9.25050066577172 5.40322581949944e-19 2.0277933864318e-16 32.4284718437425

GPX1 -1.0753848715179 7.5876492303502 -9.23984479026542 5.88756499307586e-19 2.17211232549631e-16 32.3116428787157

PPIF -1.14843127411045 5.48906521873992 -9.22917481589999 6.41560232557194e-19 2.32747359701207e-16 32.2135948944633

DNASE2B -2.34388263864914 -2.37116820623176 -9.16960423896855 1.03499071953089e-18 3.69322016262767e-16 31.3599067998039

TREM2 -1.5254749371322 4.2189235221752 -9.15304978043602 1.18164521080157e-18 4.14852762959963e-16 31.6239641638551

ZNF385A -1.0678270039845 4.53296711408672 -9.15085885585704 1.20253639546317e-18 4.15485868572173e-16 31.6066875155962

LILRA4 -1.92013640466352 -0.248786168685133 -9.14284507645664 1.28211395765248e-18 4.36058976815963e-16 31.4136707158805

INSR 1.20322825589374 8.3946895094741 9.13117947103378 1.40736087791201e-18 4.71292680454011e-16 31.4456845233588

GPR143 -2.41081639705378 1.30694347282854 -9.12626053706623 1.4637364685278e-18 4.82744722885524e-16 31.4531147443748

SPRY4 1.31342595424568 6.76645089749809 9.09097630542716 1.93922254465379e-18 6.20750840139399e-16 31.1340359292788

C1orf127 -1.26559904557196 -1.23625954603847 -9.07535202986595 2.19592035524139e-18 6.92733309746945e-16 30.6751782814502

CTSD -1.09242742352788 10.2675380635395 -9.06937038131768 2.30286486914637e-18 7.16092280095842e-16 30.999810432434

LY86 -1.20188576202399 3.4168081548987 -9.06328310978971 2.4169912353115e-18 7.30703447486466e-16 30.9506332777984

EPAS1 1.39898855548099 9.49438583236509 9.01493655288713 3.54616330362061e-18 1.02919115506546e-15 30.5477782813859

IGSF22 -1.07569717941938 0.0703423654658821 -8.97958249839246 4.68922119228554e-18 1.34302996963789e-15 30.1225338145001

CHRNA1 -2.71771339112302 -1.7123741048845 -8.94429251536389 6.19274725686248e-18 1.75061726675488e-15 29.7931703369993

NDUFA4L2 2.43320514689524 10.0413485248459 8.93687280167197 6.5649985003331e-18 1.80886483995887e-15 29.9404041960791

OTOAP1 -1.42890999761091 -1.10151498443904 -8.92267830619501 7.33992562628522e-18 1.99710201384188e-15 29.5678440647285

BCL2A1 -1.47999184648495 1.56013755856196 -8.90687104374803 8.30977769544378e-18 2.20584062313079e-15 29.7419926556728

PELATON -1.43228391498397 1.34487624328445 -8.88857023098122 9.59188082355962e-18 2.51549963718581e-15 29.5881254005767

ALK -1.78215928984188 -2.04681377703225 -8.88026711626232 1.02363610337084e-17 2.6376274936462e-15 29.1543078605129

M1AP -1.13589340898369 -0.675733521840863 -8.83019608825918 1.51374425406211e-17 3.74428081570113e-15 28.9023575261209

KCNK13 -1.40164942215231 0.581413195713078 -8.82617078763705 1.56200205403695e-17 3.82023581013734e-15 29.0519018808119

KRT36 -1.94413212485539 -3.50059422434726 -8.82139980225431 1.621174175842e-17 3.92089980950586e-15 28.5553118626959

DNAJC5B -1.89893445073347 -0.220552365026656 -8.81328333015781 1.72697705402811e-17 4.13089115769559e-15 28.9084374778723

CSPG4 1.64583390877097 6.94278693312023 8.8104149704542 1.76597873188966e-17 4.17826728880893e-15 28.9693206486852

DOCK6 1.05032392363595 6.47740252218508 8.78893224773982 2.08714896348456e-17 4.83308207320941e-15 28.803402617351

STC2 1.72807844925815 6.74706059179874 8.75262025080372 2.76645197834623e-17 6.27264168881901e-15 28.5362666485774

ADAMDEC1 -2.21841179802375 1.22376152635146 -8.73614965176038 3.1427322895745e-17 6.90988421688567e-15 28.4584771277378

ARAP3 1.31953355055884 5.230123525338 8.71995236086127 3.56204071948425e-17 7.676726766437e-15 28.3276596308146

PLVAP 1.59685971647796 9.27806910803496 8.71219124794778 3.78211934203587e-17 8.07111683510733e-15 28.2218807230249

CD300LF -1.1769142595086 2.13048040731947 -8.70604852191213 3.96579643483285e-17 8.38092145602006e-15 28.234169767575

LILRB4 -1.42172275265427 4.15449238990128 -8.69160718560636 4.43309917257795e-17 9.19002568471469e-15 28.0713435147934

ACP5 -1.86882028126332 5.64951196547081 -8.67235073247228 5.14190235232824e-17 1.05588479719933e-14 27.9147261794287

STC1 1.66402831202356 7.27902990289125 8.6501493992032 6.09905202916449e-17 1.21796390384242e-14 27.7482786359095

PPP1R1A -3.09447600864755 1.60323471206339 -8.64623951008783 6.28498985345889e-17 1.24368521945672e-14 27.7752845560664

HAMP -2.09631001675841 -0.463858931797025 -8.61446618844744 8.01921043784016e-17 1.5585192285756e-14 27.4252221324112

RENBP -1.26185339403036 4.10410184072571 -8.60314296497761 8.74534627270708e-17 1.68460134794704e-14 27.4097407564439

SIGLEC9 -1.09337174408011 2.11445332660779 -8.59988960942205 8.96574717782456e-17 1.71190718262901e-14 27.438925652951

EGLN3 1.90262633722175 8.76707233210992 8.56025351468833 1.21349989151707e-16 2.29689149031757e-14 27.071658437284

SIGLEC8 -1.58506192809873 2.48091726459618 -8.55399561048228 1.27278037265216e-16 2.38832848030341e-14 27.0944590257947

PLA2G2D -2.44988626531177 -0.315483569276896 -8.5374022762606 1.44418831549487e-16 2.68680744131425e-14 26.9025964864127

GPR4 1.53133851238046 5.56964463067021 8.46092019922761 2.57941009853547e-16 4.64016691031583e-14 26.3866601473236

RGS5 1.96411265229575 9.66268355543634 8.43441555192081 3.15082964605582e-16 5.5759438134713e-14 26.1449728762549

DAPL1 -3.1101664345375 -3.623554705673 -8.4016486090454 4.03259163406078e-16 7.0788243627904e-14 25.6407013233226

SLC37A2 -1.19879772174007 3.98864520761872 -8.38793962805002 4.47018913729571e-16 7.78420855612126e-14 25.8179316556828

PMEPA1 1.34855428593529 6.81756241075957 8.38101492156397 4.70874679730304e-16 8.13454694737263e-14 25.7477723002994

ITGAD -1.71890268760289 0.404296157897791 -8.37570228610902 4.9002577896694e-16 8.39873317383731e-14 25.7366471998977

L3MBTL4.AS1 -1.14542536572015 -0.677314183681491 -8.32674660991198 7.06935369199632e-16 1.14258191946714e-13 25.2358698736373

OLFML2A 1.65036960821341 6.95657846859309 8.32642514659449 7.08634902044671e-16 1.14258191946714e-13 25.3502915640254

SCN10A -1.96868271660514 -5.11730095193699 -8.31870070778054 7.50709044254877e-16 1.20152086516882e-13 24.8897631376725

CCR1 -1.31547489249844 3.66706402417806 -8.28179418087618 9.88359491212556e-16 1.55895804675534e-13 25.051805858315

TRIB2 1.20238830149579 6.38833262799717 8.27773478528129 1.01865830331441e-15 1.59518958908236e-13 24.998742002174

FLT4 1.41963425495148 6.10267095917691 8.27181145241663 1.06452251164143e-15 1.65510439363564e-13 24.972479193842

SV2B -2.17230320431303 -0.179950737176293 -8.26577934645971 1.11332487994062e-15 1.71870515330975e-13 24.9317920472337

LDB2 1.46852922466274 6.06509741080284 8.26156550728515 1.14872140639756e-15 1.76086048260956e-13 24.901253552264

CHRNA3 -1.73531376500031 -3.94486589531329 -8.21976350621166 1.56594842800274e-15 2.38363632393956e-13 24.2216140756501

ANGPTL4 2.12455773040392 9.13148586692041 8.17460421730982 2.18561675521751e-15 3.21448107505537e-13 24.2405525039042

NOTCH3 1.41118345624263 8.07027168988115 8.13397377954459 2.94671806125234e-15 4.30477933149528e-13 23.9439998556826

TMEM150B -1.14666635773718 0.694159679251771 -8.11760239818189 3.32267565426619e-15 4.7582026951587e-13 23.8790514844551

NRP1 1.00454372128961 8.69374091665327 8.04600016299759 5.60583276371231e-15 7.87239753340166e-13 23.3237626727452

PODXL 1.36507334935278 8.00413161294481 8.04218958663933 5.76350089338467e-15 8.04193102219899e-13 23.2869915234018

CDH13 1.512410872094 6.3021213169185 8.03990185925235 5.86025346358863e-15 8.12484949948622e-13 23.3000360607235

PLEKHG2 1.09214049929972 5.61232702852762 8.02080736121396 6.7328858971777e-15 9.15967045774169e-13 23.1797158290113

SEMA3F 1.0142497868222 6.68566697753925 8.01300000601914 7.12556534408593e-15 9.6336758288645e-13 23.0833226681

HAGHL -1.79929821463305 1.45440752010497 -8.00206829566817 7.71362696970431e-15 1.03643529783675e-12 23.1125636699541

CCDC170 -1.12632505728213 0.982492003807321 -7.98050514154105 9.01751768951778e-15 1.20419820581432e-12 22.9335955303108

PHKA2 1.03358133757375 7.02190467603643 7.91881732304214 1.40722618810346e-14 1.84524653231614e-12 22.4137537762136

DHRS9 -1.54759802070095 0.322768533779355 -7.89057179653539 1.72377671628107e-14 2.24679328043653e-12 22.2938654445365

KCNJ5 -1.33898635016241 3.13321280420903 -7.88009157804142 1.8582966842143e-14 2.40771094793409e-12 22.2107858441727

SEPTIN3 -1.69801497873066 -0.0912505995711259 -7.87575030657321 1.91700629186551e-14 2.46908141745778e-12 22.175629622999

LAMA4 1.15221842140159 7.24827269556929 7.8722534648374 1.96562351733843e-14 2.51680747658268e-12 22.0860373866244

PTCRA -1.3909898284677 -2.34034746661172 -7.86753634351082 2.03313842670346e-14 2.58803065111428e-12 21.8961208004587

CD34 1.27882497759895 7.67728830756742 7.85220763265953 2.2687213941288e-14 2.85452361768795e-12 21.9451811561587

CEBPA.DT -1.23828060385886 -0.599859020802254 -7.84564791809723 2.37758142846416e-14 2.95730371162168e-12 21.8996636590879

ALOX5AP -1.12772393733919 3.47675300084728 -7.83959811222309 2.48253814578376e-14 3.07030726245881e-12 21.9172041946871

GNA15.DT 1.72731124366926 -1.57953104861337 7.83635899460576 2.54059793537755e-14 3.12436131408831e-12 21.5974777756723

DLL4 1.4794621726245 6.22159301821532 7.83071072997364 2.64506166028791e-14 3.2164836401948e-12 21.8281460745015

NCF2 -1.00651304867656 4.02313683180374 -7.79632815585088 3.37873756100652e-14 4.00684837825081e-12 21.5922801756131

SPRY1 1.27171084331288 6.63804695768676 7.7959822848218 3.38705426378531e-14 4.00684837825081e-12 21.5647558711536

SEMA5B 1.82083277752744 6.96116515458504 7.78585475155523 3.63973295261402e-14 4.28249011781348e-12 21.5010061551231

OSCAR -1.0342363396564 2.07808340011163 -7.77744236418316 3.8636819591597e-14 4.52154651639942e-12 21.5513263482546

RBMS3 1.19850476772174 4.72153158068049 7.77228199669007 4.00772574006646e-14 4.61567016846702e-12 21.4917060918478

CD22 -1.21620394581399 1.71595229168833 -7.75400439327661 4.56181377817382e-14 5.18350795104143e-12 21.3913361861654

GJC1 1.53254859227703 5.03243258605696 7.72573655949151 5.57079021015219e-14 6.18633281349852e-12 21.1704490818898

ENPP1 -1.25850090909899 2.89819101325783 -7.72502407007884 5.59887703523319e-14 6.18633281349852e-12 21.1530212598569

CDH5 1.24357688054427 7.12095514606582 7.70456555365288 6.46770538157745e-14 7.11022944650487e-12 20.9229094010012

ANO2 1.37453155712478 1.3778054805182 7.69785421322632 6.78069641062502e-14 7.37977093850374e-12 20.886240797909

PDGFRB 1.29672029561628 7.74625632995764 7.69353677498697 6.98988887140226e-14 7.56959756536383e-12 20.8447208756247

PPM1H -1.19014680475243 3.51230652763304 -7.69043452569827 7.14411739105053e-14 7.66039424881758e-12 20.8792866711389

ANGPT2 1.55240096968039 6.55515427971094 7.67978509658359 7.6995164065216e-14 8.17538407906125e-12 20.7739884110071

ESM1 1.55665276249925 7.62913941604485 7.67614787632452 7.89879205196926e-14 8.34626245607839e-12 20.7261117106048

IGFBP3 1.61148642781441 10.8234936251219 7.65023429980396 9.47341137595504e-14 9.86639930241213e-12 20.5862021251566

APOLD1 1.71520187977323 7.49581325069783 7.64721168935339 9.67612323249203e-14 1.00295321143645e-11 20.5303140463904

CPVL -1.32318737619052 5.80870019411471 -7.64064666904726 1.01312492656573e-13 1.04515119794105e-11 20.4832478312178

SNCA -1.2449955789759 1.71566738973638 -7.63591914290132 1.04719929177109e-13 1.07520693320667e-11 20.586802077919

RTN4R -1.10222072985984 1.30471801185465 -7.63424520793541 1.05953319594086e-13 1.08276333690351e-11 20.5723665382003

PLXNC1 -1.05043140548911 4.73491831810794 -7.6222289823847 1.15237356272911e-13 1.16668443441509e-11 20.3658436608552

CAPG -1.16569402717124 6.87141419280953 -7.62047243015777 1.16660032766104e-13 1.17020227337318e-11 20.3555444523614

MCOLN2 -1.22358146622203 0.890648151104682 -7.61757780841852 1.19042315835764e-13 1.18862114165003e-11 20.4513916314917

TCF4 1.16006724602969 6.76016070996916 7.61036782310553 1.25186580314911e-13 1.24426314781491e-11 20.2818701371576

DIRAS1 -2.14380243892621 -1.48206483556609 -7.57448852227134 1.60725514811548e-13 1.56883958784887e-11 20.075127851085

ABCB5 -2.68602923029954 -3.3552860912249 -7.54883104336967 1.92065710113507e-13 1.85808636090698e-11 19.7932233969716

SLC7A8 -1.41920212928929 4.02499232932444 -7.53689541425262 2.08626791571684e-13 2.00937140360214e-11 19.8001488358727

DIPK2B 1.32891419336239 5.84293910973242 7.52536005435465 2.25966398003345e-13 2.16678880411401e-11 19.7457520086735

KCTD15 1.12070292547119 4.75230575987463 7.52401161681527 2.28083780394222e-13 2.17749984554431e-11 19.7923891238398

KDR 1.57999617966424 7.56264987291736 7.51739494212411 2.38760192290428e-13 2.25960569808076e-11 19.6456038048102

CSTA -1.11569529293357 1.55358780173067 -7.51571672059586 2.41545453101793e-13 2.26625856795979e-11 19.7797506952372

TSPAN10 -1.2678167062862 0.503420673956739 -7.49940927380972 2.7033230766826e-13 2.52546066137983e-11 19.6506690733568

LGI3 -2.16564590961703 -3.42231899872877 -7.47715549738007 3.15133436466993e-13 2.90657182693943e-11 19.2892986606151

ATP6V0D2 -2.90108027460591 -0.866973504713672 -7.47028595798656 3.30385648615708e-13 3.03439004785574e-11 19.4617268058528

SLC5A4 1.69750825497565 0.466628355306028 7.46104498929889 3.52055174863651e-13 3.17974480965025e-11 19.2258972958754

SMTN 1.01522581294418 6.09643953700485 7.44574670117424 3.91044746121701e-13 3.47423305666574e-11 19.1845435180819

FCGR2B -1.24471307569467 2.91603404561687 -7.44264817191701 3.99444672810634e-13 3.53443585084109e-11 19.2401135939042

RAPGEF5 1.12804017022222 6.02256405362428 7.4360739827649 4.17859727996453e-13 3.6675615722979e-11 19.1281772220708

IGSF6 -1.04323385179182 4.10721068308808 -7.43392466199448 4.24059648357568e-13 3.70703066899565e-11 19.1172472087141

RFX8 1.80893328995994 -0.0462763209041965 7.42768148911613 4.42586965572166e-13 3.8229327300037e-11 18.9663827266368

GJA1 1.24717104710866 7.13539265956718 7.4199679175724 4.66581510218913e-13 3.98277636585689e-11 18.9919175989776

CTSK -1.42861528476851 3.86587894942382 -7.41469635562578 4.83711907611109e-13 4.09687046419106e-11 18.9864887785042

KCNJ10 -1.95222685315893 -0.631780243523807 -7.39833708256605 5.40912481701052e-13 4.56358216635147e-11 18.9587424011453

NID1 1.03527759220509 7.71163841957148 7.39594807159928 5.49804806475347e-13 4.60292354713419e-11 18.8296624639042

ENG 1.02408273676113 8.52837457528979 7.39079099406143 5.6949394120725e-13 4.73136435811382e-11 18.803942183315

MCAM 1.0865618212927 8.57699966997115 7.38649606536173 5.86419986526232e-13 4.85346153867547e-11 18.7753002193089

HMGA1 -1.10127442467213 4.38715746017146 -7.37962510505871 6.1453337015746e-13 5.06687419250660e-11 18.7405969343469

DCSTAMP -1.67923797428385 -1.43645148176925 -7.35767473425008 7.13534966597124e-13 5.81723739727597e-11 18.6287170050977

UCHL1 -1.99204438735922 2.51114658901387 -7.35766991997156 7.13558315373126e-13 5.81723739727597e-11 18.6593336138382

CCR3 -1.47526001899213 -3.22973603494427 -7.35648115856212 7.19346771896705e-13 5.82082571891285e-11 18.4867523913222

LOC105377067 -1.47526001899213 -3.22973603494427 -7.35648115856212 7.19346771896705e-13 5.82082571891285e-11 18.4867523913222

SPARCL1 1.364551853744 8.68395260651682 7.34958539211421 7.53847610060663e-13 6.07740775118165e-11 18.5284228179513

ESAM 1.13371025686064 6.84375142187244 7.34294792194112 7.8859252979375e-13 6.33405667749836e-11 18.4818969718054

CCND1 1.22732701487946 9.43754689620252 7.34200751600991 7.93641039240733e-13 6.35117077248274e-11 18.4922891822486

SCIMP -1.03497247893122 2.62050243431481 -7.33868412287015 8.11738645896163e-13 6.44858215519043e-11 18.58021581862

SLC2A1 1.17436200575617 7.90418745877993 7.31651191342669 9.4331346528466e-13 7.44556343718162e-11 18.3031709444328

ETS1 1.02033244208161 8.10141134197787 7.28786832920528 1.14474399931855e-12 8.86748848155402e-11 18.1169728481436

NMRK2 -2.95992057464006 -4.2377187368476 -7.26503544304879 1.33513282191047e-12 1.02063482370823e-10 17.9281045657763

PLCB1 1.37859054161578 5.58276983670407 7.26404938605526 1.34402168124544e-12 1.02291328446397e-10 18.0308723956802

OBI1.AS1 1.25621971143678 -2.45348718885513 7.2596333804055 1.38454957710357e-12 1.04644064738935e-10 17.8427645370855

DLEU1.AS1 -1.32471284197165 -0.455908238046169 -7.25178095242399 1.45960627192961e-12 1.09556033521006e-10 17.9915942377216

DLEU7 -1.32471284197165 -0.455908238046169 -7.25178095242399 1.45960627192961e-12 1.09556033521006e-10 17.9915942377216

GMPR -1.13959651497134 2.28873689806326 -7.22196545279255 1.78283953595571e-12 1.31104960064689e-10 17.8245665226299

TRIM63 -2.31271194828302 -2.37769437287226 -7.20458680432001 2.00272063571279e-12 1.44827973679602e-10 17.6272268322995

PEAR1 1.3598508110084 4.67798519136989 7.19593665256688 2.12189984736264e-12 1.52938390654114e-10 17.6433148583111

CTSS -1.14140238278372 7.08230121315322 -7.18522863645517 2.27912862025957e-12 1.62655057958e-10 17.4571349011607

OR7E47P 2.19125816945119 1.59685417280882 7.1820453488961 2.32804078179352e-12 1.65063399665471e-10 17.4939956655804

CDK18 1.28834323133366 8.05635445237805 7.18066285538644 2.34960340417632e-12 1.66051354865928e-10 17.4130432506487

MEP1A -1.84564016154931 -3.83010490431385 -7.17208370326356 2.48787140990426e-12 1.74688699933504e-10 17.3139862553524

EFNB2 1.17715159299201 6.42990297986762 7.16580759528282 2.59406014541044e-12 1.81559187090511e-10 17.3321169733516

EXOC3L1 1.19109777165471 3.83448598887528 7.16025091664639 2.69178842440326e-12 1.8719539499676e-10 17.445316176445

APOC2 -1.66477590089429 -1.9203846232797 -7.14645403482175 2.9503706020681e-12 2.02589012287748e-10 17.2521089969233

ERC2 -1.75177411260653 -2.08421921388455 -7.1184393867522 3.55284647774592e-12 2.38687682966344e-10 17.0712981484231

ITGA10 1.67804670587296 2.18168965401642 7.11467751496064 3.64245283611334e-12 2.4395467964209e-10 17.127910704762

PTPRB 1.40596730123246 6.85708545516227 7.1101441821369 3.7533910333604e-12 2.49920585994563e-10 16.9651461773359

LRRC77P 1.95894414724037 -2.06689172080936 7.10978741547401 3.76226138980316e-12 2.49920585994563e-10 16.9066427846606

ROBO4 1.20055226512742 6.29144180203118 7.10963834645295 3.76597382304483e-12 2.49920585994563e-10 16.973894035056

DLC1 1.01681934534796 6.07182085972964 7.0967190201871 4.10178033933558e-12 2.68739372427366e-10 16.8938871194409

HILPDA 1.55391859301784 7.33562901049724 7.09636889910036 4.111279047104e-12 2.68739372427366e-10 16.8697192961136

LZTS1 1.34386537712989 5.21306376715042 7.08769620038984 4.35359334111888e-12 2.8372654567705e-10 16.9079604256009

AFAP1L2 1.07357881329467 4.52630417045502 7.08677931820588 4.38001722773874e-12 2.84596522376684e-10 16.9351248787314

HIF1A.AS3 2.08426689340023 0.850880373243003 7.08238468950416 4.5088721857306e-12 2.91230328981596e-10 16.8256467734355

NOSTRIN 1.12877763581741 5.10122559373588 7.08140764973393 4.5380224044931e-12 2.92245957628998e-10 16.8627008222459

MAN1C1 -1.14388665220024 3.18769582656075 -7.07644486456058 4.68897284700447e-12 3.00190799884548e-10 16.8264305260577

RGCC 1.21710469602112 6.13228163675637 7.06908414854168 4.92199106013286e-12 3.12352709638227e-10 16.7198834498879

CES1 -1.91702592418613 1.13210121583059 -7.0558952294539 5.36831791848696e-12 3.38701959802045e-10 16.7834504398726

PPP1R13L 1.00575887313087 5.50456961357278 7.04132413464457 5.9077402074192e-12 3.69522359468085e-10 16.5710130492999

FNDC10 -1.20020299202694 0.83611334388934 -7.04028657376759 5.9481209839854e-12 3.70982090138711e-10 16.691477925739

ARHGEF15 1.29014739132767 5.10489194962512 7.02978386729373 6.37245488037951e-12 3.96312072517774e-10 16.5413248344772

PCDH12 1.24265007426183 5.94667568207075 7.01713930673174 6.92292825116995e-12 4.25681862268973e-10 16.4001415903419

NOVA2 1.31578103270222 3.77740892732621 7.01624883593938 6.96340951067887e-12 4.26964886813935e-10 16.5329956795063

SIGLEC15 -1.339346160592 -0.557566317209983 -6.99378931623387 8.06496532655187e-12 4.88997493768954e-10 16.3634585049981

TIE1 1.15734548424156 6.06498223297053 6.97926654783944 8.86661343660149e-12 5.36109929651402e-10 16.1481261768962

JAG2 1.09121414552723 5.3741574151407 6.97711650911751 8.99176341323192e-12 5.40673243690108e-10 16.1744301358443

BCL6B 1.3279332771441 5.33760902407165 6.96737034181266 9.58118213422687e-12 5.6981855605387e-10 16.1299655904825

LILRA2 -1.02149893020945 1.97269878431455 -6.95780387108069 1.01965932901621e-11 6.03122951486299e-10 16.1605637326411

ADM 1.12550006802667 7.44212084146903 6.94567929999053 1.10326062593563e-11 6.50804174654223e-10 15.9031613986762

NCKAP5.AS2 -1.69145551491806 -3.31292470352259 -6.94422696464666 1.11371564095893e-11 6.53429874845093e-10 15.923667248966

CSPG4P13 2.05689954034554 -0.153717642080618 6.94376195863799 1.1170836319954e-11 6.5364407036677e-10 15.9234739700764

LINC02685 2.28990221618087 -2.54044131501497 6.9320877565827 1.20499526064588e-11 6.957329400127e-10 15.8121682791031

PECAM1 1.04568048212631 9.17679043796941 6.91598746824564 1.3374759987789e-11 7.60126372465283e-10 15.7368375298191

TMEM204 1.23496519539963 5.79814147909348 6.91444252106678 1.35091657755593e-11 7.65344532613173e-10 15.7560596436907

EBF1 1.31679926870058 4.56562299598104 6.91412563563061 1.35368973701508e-11 7.65344532613173e-10 15.8524455586832

GABRD 1.94278935708525 3.49706872593578 6.91005894707619 1.38977872622762e-11 7.83712785849654e-10 15.8771583842691

VIP 1.85406362804656 -1.42179459469184 6.89980975233726 1.48497764856254e-11 8.30938521240638e-10 15.6330216247423

CPEB1 -2.07303243141127 -1.09046076024828 -6.88625883754953 1.62073244702767e-11 8.93126156315222e-10 15.7025626826348

ADA2 -1.09849911535196 6.35351760179003 -6.86141529912006 1.90198854534548e-11 1.03760863825902e-09 15.3775664809522

ADCY4 1.35611575490574 4.24829518585305 6.860495691296 1.91327117963358e-11 1.03943287547638e-09 15.538917752342

AATK 1.0789133000687 2.21513954468839 6.85839141956005 1.93933622499384e-11 1.05008785098112e-09 15.5508342717442

IFITM10 -1.09227920149018 3.05587443609687 -6.85316816580714 2.00555148698316e-11 1.07819123391535e-09 15.4327045269158

CA9 2.42370039101367 7.18292149925384 6.85297041863333 2.0081014619661e-11 1.07819123391535e-09 15.3598563611451

PTAFR -1.04102394384894 4.23901712065129 -6.85274193280042 2.01105178007824e-11 1.07819123391535e-09 15.3502086657218

BTNL9 2.15216476717556 5.3122653588056 6.83725774389563 2.22122732781313e-11 1.18213827003688e-09 15.3737520453561

C3orf70 1.37037368008652 3.25759325412023 6.83179521677659 2.30040254741293e-11 1.22128932315945e-09 15.3931865560394

PSAT1 -1.76762384151484 2.63694844659461 -6.83005117496702 2.32625977926136e-11 1.23201208309445e-09 15.2767817803545

VWF 1.41540121552195 9.75880257950188 6.82730583394371 2.3675414687207e-11 1.24780327238846e-09 15.1862680048155

JAM3 1.01476506487157 5.48179537370565 6.82503733922835 2.40219476666774e-11 1.26300902140233e-09 15.2054384862189

ADGRL4 1.18376380070284 6.40902584855465 6.80585272893013 2.71587560870421e-11 1.41426948264748e-09 15.0446144147116

MCF2L 1.05376531879032 5.95510894021445 6.80159775910878 2.7907145289393e-11 1.4497728675757e-09 15.0339644721176

C9orf153 1.19532066592421 -2.44365443554392 6.78686136132424 3.06588784192311e-11 1.58893287274144e-09 14.9482153834638

SAP30.DT 1.2876376143947 2.86394841257037 6.76039667369239 3.62846467385544e-11 1.83676257106538e-09 14.9571755693338

VWA1 1.06853757535192 7.13381072000758 6.75656670420625 3.71785613299873e-11 1.87764673890913e-09 14.72086216054

CCM2L 1.20618267338588 3.3285679023693 6.75330150833272 3.79576959856452e-11 1.9125582604619e-09 14.9087631006334

SHANK3 1.06149877537105 6.18975474399786 6.7490852935131 3.89874999001958e-11 1.95539380259807e-09 14.6972595879432

ERG 1.13351273561015 5.28934349901526 6.74798192688862 3.92614905155584e-11 1.9601028992022e-09 14.7503267724387

DLX6 1.42749483652898 -1.55422412767095 6.74467541559206 4.00939357373105e-11 1.99708169151954e-09 14.7064955311193

COL8A2 -1.18234235059407 2.49688765419703 -6.73300631224225 4.31724923134733e-11 2.12588167291136e-09 14.7277636769761

ANO5 -2.1053884746913 0.0584601282041833 -6.7243524613783 4.56041984335848e-11 2.22570983700412e-09 14.7382300701986

LOC102723370 -2.1053884746913 0.0584601282041833 -6.7243524613783 4.56041984335848e-11 2.22570983700412e-09 14.7382300701986

PKIB -1.11599482771944 1.77886211428373 -6.71563239282491 4.81903134752574e-11 2.34142534244627e-09 14.6656530662962

CACNA1C 1.41057062464575 4.06988931689731 6.71403727542862 4.86787031726675e-11 2.3546429599099e-09 14.649573707537

CACNA1C.IT2 1.41057062464575 4.06988931689731 6.71403727542862 4.86787031726675e-11 2.3546429599099e-09 14.649573707537

DGAT2 -1.38663238009227 0.560523842596627 -6.7084141382611 5.04394261515446e-11 2.42365339744078e-09 14.6417521683229

NES 1.0530670719918 6.58597193457674 6.69063670914467 5.6426998222999e-11 2.68073346416686e-09 14.3237794268887

ALOX15B -1.63015746078911 1.03530518913265 -6.69035147652124 5.65285367782693e-11 2.68073346416686e-09 14.5255362221856

TRPC6 1.43274666859108 2.64423616872987 6.68615673999281 5.80426635595928e-11 2.734663761259e-09 14.5057238808728

FCGR1A -1.13824122800616 2.67440027028203 -6.68347976201088 5.90296622231551e-11 2.76917814140392e-09 14.4164243817534

GIPC3 1.23885932510863 4.08099178152857 6.67561214822976 6.20267120829621e-11 2.89108231672342e-09 14.4065616130383

NCF1C -1.01595623176425 0.893395338753476 -6.67236648394561 6.3306136136742e-11 2.9444116779668e-09 14.4243463884933

USHBP1 1.35527409771914 3.0354521657276 6.67147990259892 6.36600996872163e-11 2.95456159891607e-09 14.4184369554998

PDZD2 1.57796525502725 5.40630497653022 6.66158502964001 6.77447204803058e-11 3.11754615368883e-09 14.2448044386325

LRRC71 1.56702798851563 -1.88743187438686 6.65800703604939 6.9284114512613e-11 3.18166101391571e-09 14.1902371340642

COL4A1 1.07107918541734 10.1569107921437 6.65515911987705 7.05338739058579e-11 3.22544292711935e-09 14.1389179293005

RARRES1 -1.59847044967644 2.5350112076039 -6.64233985918169 7.64386150283171e-11 3.48083542535853e-09 14.1478563574011

MMRN2 1.11853733546774 6.63483361048209 6.63879570399705 7.81549057478963e-11 3.54416215294679e-09 14.0071146332584

SLC12A8 -1.55658817416241 1.97850599688471 -6.63254460063604 8.12746312113218e-11 3.67797276003501e-09 14.1294497626221

PLPP1 1.01686234807477 7.07383822544402 6.62967068474787 8.27495760916765e-11 3.7369502547459e-09 13.9423458074148

EDNRB 1.45053001677455 7.00839746535792 6.62135105806435 8.7168904127701e-11 3.91968076834533e-09 13.8992851627458

KCNE3 1.09982272231799 6.20801790540748 6.61999933288632 8.7908457941798e-11 3.93724980250847e-09 13.9080518253477

HHATL -2.91047668149213 -3.92395675423529 -6.61131855851954 9.28066396564562e-11 4.1480947133513e-09 13.9634941036342

NDRG1 1.02686602197507 10.9906511549748 6.59814571418994 1.00754732887151e-10 4.46665635591571e-09 13.8114633514942

DOK7 -1.61670497626177 -1.23752177570167 -6.59607926722116 1.02060673592494e-10 4.50619610971161e-09 13.9369725749219

NEURL1B 1.01325359088668 5.77651501564241 6.59069590451434 1.05541244491921e-10 4.63168602591865e-09 13.7483472265626

MOCOS -1.68111160283353 1.79317673892937 -6.58871993851645 1.06847765525608e-10 4.67958815331171e-09 13.8731178498287

FA2H -1.80683859408069 -0.300999336230876 -6.58625125572259 1.0850236905047e-10 4.73300815054425e-09 13.9065961437848

FAM9C 1.54535235898269 -3.05992425423585 6.58424849320319 1.09863126804162e-10 4.77323489250738e-09 13.7503922333896

CGB7 1.34864477796827 -3.54602698569051 6.56334550777396 1.2510240808276e-10 5.40298435860601e-09 13.6275408383308

NIPAL4 -2.08931266089997 -2.85415528441932 -6.5616465046334 1.26428395587015e-10 5.43720584614109e-09 13.6824374603351

RHOJ 1.07145806099966 4.70490897731641 6.56137170039767 1.26644156934512e-10 5.43720584614109e-09 13.6593934390847

JCAD 1.06000523198204 6.43779411630618 6.56021941564603 1.27552804385798e-10 5.46543679737337e-09 13.5350120831406

CYYR1 1.18735734665492 5.53256118306323 6.55658974448199 1.30456988961202e-10 5.56795544846761e-09 13.5685685747214

HOXD9 1.17972796449903 4.33154822204676 6.53872642388092 1.4572110307533e-10 6.13206612272951e-09 13.5621913328793

LINC01117 1.44637228356057 -0.0409263544718185 6.52024529265538 1.63353280229861e-10 6.77278257288263e-09 13.4318669278624

TM4SF19 -1.66637576880313 -2.39176367352532 -6.51402635972396 1.69744065148781e-10 6.99776338275286e-09 13.4079875602208

SLC35F1 1.52862159920937 1.50116083540743 6.50567075404646 1.78717604762559e-10 7.33989830729549e-09 13.4092453292227

BNC1 -2.35888991779958 -2.81942316862231 -6.49997674156873 1.85097320786635e-10 7.57333342399001e-09 13.3374741932899

ADGRA2 1.14881537795977 5.89247431591643 6.49952732254337 1.85610256923486e-10 7.58007216220172e-09 13.2004076460597

RGS5.AS1 1.59883373308642 -3.29273887109093 6.49871867180257 1.86536705163841e-10 7.60364131329835e-09 13.2555989087152

CR1L -1.72278305917521 -1.74314580882638 -6.49524600909231 1.90567006379184e-10 7.73894035047704e-09 13.332328532824

TAL1 1.42533091712824 2.88174951924092 6.49349726014674 1.92628742623263e-10 7.79358706446202e-09 13.3602535166635

GRPEL2.AS1 1.4127481047484 -3.09624717459746 6.49020280721805 1.96572361448592e-10 7.92368628083613e-09 13.2085941987864

EBF2 1.75037492585527 3.28199102773833 6.48723000356922 2.00198769101898e-10 8.02527920265378e-09 13.3219575627655

EGFL7 1.25029959318275 5.75918058484015 6.48470924663647 2.03325026705648e-10 8.12068964459053e-09 13.1245164081491

TMEM63C -1.433285407847 -1.21785348679757 -6.48102973662396 2.0797440146517e-10 8.24896970864518e-09 13.2582292857081

PADI2 -1.14069906402858 3.22653618420899 -6.47802055057695 2.11854044346086e-10 8.37430754037096e-09 13.1314111280549

LINC02204 1.32934554921937 -2.65367399569192 6.47792112257208 2.11983436153094e-10 8.37430754037096e-09 13.1416322407335

CR1 -1.41253843517762 0.796964251021907 -6.47286771944384 2.18662694407762e-10 8.59139146060244e-09 13.2364661919025

LDLRAD2 1.15449102647272 1.73008466824122 6.4667487667615 2.27026672147162e-10 8.88793088602029e-09 13.1960060244294

CD36 1.53723165326621 6.74542961703094 6.46605270252108 2.27997745911154e-10 8.90992268446693e-09 12.976049234945

BGN 1.26229500021948 9.56553904958133 6.4605749028798 2.35783106394387e-10 9.18120013754316e-09 12.9504493325986

TFCP2L1 -1.81525669287186 2.35895340250574 -6.45935177031512 2.37556733702506e-10 9.22965770164073e-09 13.041339213639

GSDMA -1.66506515829788 -0.843504869996531 -6.45646495470807 2.41794797639796e-10 9.3483967321944e-09 13.1312649643275

CFD -1.10906733125877 3.40291223882283 -6.4508071120986 2.50316834700167e-10 9.66072081723145e-09 12.9569206241331

NOL3 1.00063873649895 6.58011756780098 6.43724681429064 2.71957263834374e-10 1.03491149683266e-08 12.7935155969828

LOC101927057 -1.33461743643644 -1.25231452013122 -6.4305574450391 2.83297158228568e-10 1.07057799360438e-08 12.9606446079796

OMG -1.33461743643644 -1.25231452013122 -6.4305574450391 2.83297158228568e-10 1.07057799360438e-08 12.9606446079796

APLN 1.23655672760781 6.34494087048278 6.42732278344511 2.88945420942367e-10 1.08814445980147e-08 12.75065499521

LINC02202 1.13148480425414 0.45468522614968 6.42521427278908 2.92686451860018e-10 1.1003291878475e-08 12.9175172182494

SCIRT 1.50685892412687 2.92072880273658 6.41658135857342 3.08504309036968e-10 1.15183761488983e-08 12.9106851313739

CCDC13.AS2 -1.3634980709208 -3.18186821351659 -6.39642817666977 3.48759824516037e-10 1.28119303063551e-08 12.6965836100209

ELAVL3 1.75639636097663 -1.87722985745446 6.39629776626622 3.4903637027007e-10 1.28119303063551e-08 12.6808436361113

NAPSB -1.16805319668647 3.48517701189615 -6.3952632524621 3.51237760070084e-10 1.28710308475514e-08 12.6160277079296

G0S2 -1.4745653371487 3.78938869254895 -6.39013578669705 3.62350966711682e-10 1.32337139134449e-08 12.5458070048601

C1orf53 -1.23124916375814 -0.480976307529011 -6.3670641995167 4.16765579980262e-10 1.50194310917721e-08 12.6167820969419

AUTS2 1.07953265991619 5.56059968197228 6.36234477898184 4.28843748496168e-10 1.53783226911303e-08 12.4100587623222

HRK -2.17440435760107 -2.8316045803399 -6.36095844499792 4.32456368676593e-10 1.54569421625343e-08 12.5329293681947

KCNIP3 -1.43782805877236 2.66541924893451 -6.3583941346142 4.39217329790894e-10 1.567285838944e-08 12.4456325970294

TDRD6 -1.02333589849997 0.896505003507966 -6.35200084625297 4.56527360026698e-10 1.62638805985289e-08 12.5355354084678

MIR210HG 1.3399089138065 4.35327627493629 6.3517117626204 4.57325643291676e-10 1.62656981658986e-08 12.4722782492227

NRIP2 1.15393102865836 4.708169018699 6.3505180600333 4.60636458838403e-10 1.63567272423092e-08 12.4164103446956

GRM1 -1.47492860936409 -2.44140617812915 -6.34885935377301 4.65275970473733e-10 1.64677431695963e-08 12.4549884584487

DLX1 1.44059776327383 -0.637531360419559 6.34620364342429 4.72799566259732e-10 1.66527963734233e-08 12.4176497965585

ANKRD1 -1.55179617298595 -0.861402234346491 -6.33681896278558 5.00352281195234e-10 1.75099165671651e-08 12.4399787192469

SEMA3E -2.44798635106528 -3.5520440424712 -6.31778134651511 5.61158387683631e-10 1.94502143705567e-08 12.2696574968342

RAPGEF4 1.27963805337351 4.28461135167555 6.31529915524951 5.69601071047852e-10 1.96801690690454e-08 12.2552604095199

RHOB 1.09162573574907 9.33003253057208 6.31490868851793 5.70940431449061e-10 1.96951828389092e-08 12.0900554272143

SPAAR 1.23382209123508 2.35851326399789 6.30555189195619 6.03973405224763e-10 2.06060957860931e-08 12.2711721369675

CFAP161 1.09570980681653 -2.53433941218121 6.30361607567093 6.110374114409e-10 2.07819552106782e-08 12.157479585673

PCDH17 1.39650141607843 5.27761077806174 6.3008588291326 6.2123866219582e-10 2.10959468955014e-08 12.095721543025

ADGRF5 1.06899058391884 7.46308204584993 6.29012900258701 6.6254677570984e-10 2.23591560726761e-08 11.9196832913158

SLC1A3 -1.12850961743619 4.06319755177109 -6.28284745515909 6.92097809340815e-10 2.31767584860331e-08 11.9185291045635

UST -1.28370836160305 1.50147119170572 -6.28204000696399 6.95453075749621e-10 2.32533442393886e-08 12.1017825089522

EDNRA 1.18132577327698 4.86848925662629 6.27954962205263 7.05902150026217e-10 2.34944527517136e-08 11.9919551529093

NETO2 1.10570156777575 5.8918471839731 6.27318312688908 7.33317633942441e-10 2.42585485380321e-08 11.8658504654315

GJA4 1.21168117770838 5.63073714986943 6.26827434451521 7.55164455600196e-10 2.48678739864591e-08 11.8592824940987

WFDC21P -1.4914247578809 -0.774863493432509 -6.24651241928248 8.5993331720629e-10 2.78130289979633e-08 11.928178862332

TBX2 1.08068156171143 5.71662331402034 6.24508071581271 8.67303701071869e-10 2.79682957944169e-08 11.7099570418658

HEYL 1.22857595778101 5.92505732263646 6.24421326660902 8.71799333027675e-10 2.80302157784541e-08 11.703546018532

CES1P1 -1.77063467157471 -5.14092076318622 -6.23915697207011 8.98461199389535e-10 2.87600072457529e-08 11.797332823401

LOC107987423 -1.77063467157471 -5.14092076318622 -6.23915697207011 8.98461199389535e-10 2.87600072457529e-08 11.797332823401

ITGA6.AS1 1.17312681880986 -0.0880859556549127 6.23705757887635 9.09764123971602e-10 2.90790538714976e-08 11.8288033715601

PVALB -2.85934863896862 -2.7357560554231 -6.23004530337989 9.48534683953075e-10 3.01215260469794e-08 11.8225168851265

CNTN3 -2.40528232506649 -1.78898713334208 -6.22966521935068 9.50681692207234e-10 3.01215260469794e-08 11.8297168878101

ADAMTS4 1.40066871401274 5.94288150062514 6.22699258542006 9.65913571787145e-10 3.05152985734264e-08 11.6133971699204

KLK4 -2.78849964506536 -4.43013578256622 -6.22489319954926 9.78045514016958e-10 3.07940576992623e-08 11.7356822462862

LOC100419170 -1.39160494079258 -1.21865763083253 -6.21968738587562 1.0087753826423e-09 3.16854455324315e-08 11.7661082615323

UBTFL6 1.73467578966753 -1.437341878615 6.21897320279285 1.01306416948316e-09 3.17743051543802e-08 11.692460713181

SMCO3 -1.44561036211961 -0.884033464458188 -6.21653297348112 1.02785311278809e-09 3.21455153822677e-08 11.7569976144488

CD248 1.19322915061388 5.83977101797166 6.21522223052464 1.03588377057612e-09 3.23501572450487e-08 11.5390655878733

INSM1 -1.37068007925318 -5.61223913219533 -6.2149809138648 1.03736894184059e-09 3.23501572450487e-08 11.6591643961472

DLX5 1.40495747430967 0.938793747254656 6.21101241663752 1.06209362677648e-09 3.29794464679651e-08 11.7113298673732

ITGAX -1.00752539063357 4.8018578018106 -6.20864514714934 1.07711567397743e-09 3.33507494672356e-08 11.4601517505023

FZD2 -1.00764255521833 1.3061182645923 -6.2030573228891 1.1134032060114e-09 3.43278294408641e-08 11.6732330044267

CPNE9 -1.10926549542 -2.50730606280791 -6.19802780249618 1.14708535443436e-09 3.52663939406393e-08 11.5966409561599

WNT5A -1.07380844402399 2.39926832284364 -6.1952771527387 1.16592545218931e-09 3.5795062507482e-08 11.5647889253919

NRARP 1.28263991349591 3.44534221197817 6.19488787458816 1.16861606430456e-09 3.5827135030588e-08 11.6183158740395

SLC2A3 1.14554724444098 6.66395138219776 6.19105210609082 1.19545482987119e-09 3.65470018002895e-08 11.3584119090651

WIPF3 -1.13317933321061 2.77807940542373 -6.16859580556458 1.36510256660131e-09 4.11553844421202e-08 11.3693296759313

CIB4 2.45680171670915 -1.09630475396295 6.16204776537688 1.41885467788172e-09 4.25402338477292e-08 11.3778869470625

MYCT1 1.21539447451657 4.61962344963947 6.16046442596795 1.43215955701286e-09 4.28211773042569e-08 11.3326216845761

IL1A -1.52725910730259 -3.42954892885124 -6.15734894212439 1.45869611242665e-09 4.35547850194662e-08 11.3592344195637

TLR7 -1.14226664465645 3.25872323100364 -6.14510150626705 1.56775237616175e-09 4.67469396875519e-08 11.1893751828068

SLC16A7 -1.14666795723588 3.66405467319033 -6.14153170701886 1.60101217725527e-09 4.7673368074303e-08 11.1288213176751

GABRR1 1.65577927691031 -2.3646625225941 6.13797665464914 1.63481953518026e-09 4.8481085588922e-08 11.2398257824482

PLTP -1.51117811945087 6.0143784194433 -6.13717330435563 1.64255522114918e-09 4.86442170051077e-08 11.0453332088135

CCL24 -1.65004613578792 -3.44439802997922 -6.12712862085572 1.74235058401132e-09 5.12510069759114e-08 11.1952443449419

DUSP1 1.02868118577411 9.66014856576851 6.12354955619695 1.77932145020112e-09 5.21974258848083e-08 10.9964712820013

KCNN3 1.44344236098851 4.66061179021033 6.12271680539691 1.78803287377122e-09 5.23823843383286e-08 11.1333138503232

HSPG2 1.23788221602942 8.80313634040546 6.0988892126299 2.0557293709066e-09 5.94250480963134e-08 10.8346114354663

KU.MEL.3 -1.16158824198223 -5.68239203706166 -6.09378480835684 2.11797350077926e-09 6.08204870599764e-08 10.9945462914619

C6orf223 2.38800352270415 4.96542144839806 6.09307027037197 2.12683232630112e-09 6.09944127096132e-08 11.0111203305015

SPATA12 -1.27204521315212 -3.32653224996331 -6.09026929853277 2.161909093833e-09 6.18374181937751e-08 10.9907937079086

CSMD2 1.21726247708195 0.96766344862579 6.08704489542181 2.20298854422868e-09 6.29297265646007e-08 11.0298944115422

COL5A3 1.23533705923639 4.99778164278327 6.0857308951616 2.2199469452796e-09 6.33310421466592e-08 10.878128733025

CLEC14A 1.15693409565313 6.08574650623936 6.0854081491745 2.22413175223372e-09 6.33673767681563e-08 10.780600750999

MOV10L1 1.25062733800268 -0.893495555087206 6.07322952459188 2.387800103609e-09 6.7764334882995e-08 10.9040226861591

CD93 1.00725990126804 7.87080225111124 6.06910430909534 2.44586167674903e-09 6.92315619217115e-08 10.6553768552449

RASIP1 1.07829497219629 4.67294014944131 6.06697927881517 2.47630688402253e-09 6.98209481146611e-08 10.7868304947005

LINC03022 1.36113715637196 -1.35240437364018 6.06604825539185 2.4897619297442e-09 7.01095057241163e-08 10.8580210316671

RUNX1T1 1.27659542723172 2.29455570797158 6.05173505926637 2.70582698804071e-09 7.5801462096116e-08 10.845264558633

INHBB 1.41394177588877 5.77147609530656 6.05104987673985 2.71661699915345e-09 7.60059154506082e-08 10.626149444009

HSD11B1.AS1 -1.11854528430835 -1.45581918673288 -6.03869611526912 2.9185251810711e-09 8.10300224698655e-08 10.7546476084967

LOC105375519 -1.18055235044065 -1.49139590498455 -6.0276779838255 3.11091467748384e-09 8.57155440313807e-08 10.6947448146298

EMP1 1.0373474045269 6.82677504856251 6.02102083877439 3.23309972878046e-09 8.82997262187759e-08 10.38764502696

FGD5 1.05241836736609 5.93878511243662 6.00291546815756 3.58959272594803e-09 9.68211460541645e-08 10.3214914818481

SCN4A 2.02122436156173 1.99200077887261 5.9927447325895 3.80639555755215e-09 1.02036714410391e-07 10.5198677270316

ANO1 1.18927648251894 5.41289308952831 5.97729407499592 4.1604093886279e-09 1.09902465002747e-07 10.2283909691631

MFSD2A -1.50068585548595 1.32792492336038 -5.97652545480971 4.17883523010164e-09 1.10255401761967e-07 10.3888575530942

ZNF280B -1.14630014549637 -0.0514055265858713 -5.97418243474739 4.23549637420774e-09 1.11480108316058e-07 10.4217205910022

IBA57.DT 1.06610226239061 -2.75097554713959 5.97174004714952 4.29535859208397e-09 1.12647072860111e-07 10.3421233998267

LRRC70 1.28713773768181 0.51106247678611 5.96971213357565 4.34568890276044e-09 1.13693041281714e-07 10.3796226194846

RBM44 1.12997587383096 -1.12175494157213 5.96395282570748 4.49178677836354e-09 1.16966372402876e-07 10.3130732684161

ATP1B2 1.50230936134078 3.81522199260509 5.95905293504349 4.61984834692566e-09 1.20000285164118e-07 10.2926535553856

RAB3B -1.94590706920323 -1.44785471972888 -5.94196048060527 5.09507399995494e-09 1.30629535638421e-07 10.2433734725925

TPPP3 1.24960233973032 4.87541294751297 5.91205212251597 6.04385508730197e-09 1.52441012381578e-07 9.92302503363531

LOC105371730 -1.0737822882568 -1.99921941856076 -5.90985350642353 6.12003256796586e-09 1.54027653045001e-07 10.0458777884977

DGCR5 1.56634735718343 2.48658701939321 5.90672162192597 6.2301653827272e-09 1.5659585437162e-07 10.0546492718048

ST14 -1.27247425399121 5.16446870807541 -5.90615209076116 6.25039965071982e-09 1.569232401352e-07 9.74623594796542

IGFBP5 1.29711950553877 9.79804752257937 5.90281371549828 6.37030067642885e-09 1.59565402559064e-07 9.75997202537854

MIR10B 1.5406219450639 -3.31494568292554 5.89501335874277 6.65927010302095e-09 1.66039326841302e-07 9.93109033353363

RNF213.AS1 1.06273686330122 1.50037141156328 5.89334402932938 6.72275212454431e-09 1.67430372419858e-07 9.9844960432399

CAMP -1.60298248880405 -3.87847365412263 -5.8904819310891 6.83296941943303e-09 1.69560253698182e-07 9.91737714555691

CALCRL 1.08460038179348 6.52523821290715 5.881997345439 7.17016197204092e-09 1.77154274285374e-07 9.62549945688627

PDE2A 1.3006430369345 4.7864845082902 5.87272411603245 7.55726053432987e-09 1.85455343912918e-07 9.71993974236029

ATP1A3 -1.27499282313762 0.0684372708682961 -5.87051000648969 7.6526571194353e-09 1.87373889222439e-07 9.8592178732738

COL25A1 2.17695619201798 1.36910343596231 5.85824982575039 8.20253137037405e-09 1.98603448652872e-07 9.78571831147626

LINC01738 2.00279026054539 -0.656826447700436 5.85791779025473 8.21794672095758e-09 1.98755606972315e-07 9.74965059433606

GLDN -1.36725599773613 0.436834954119318 -5.85665560081252 8.27680435090029e-09 1.99956937076633e-07 9.7761112534219

HECW2 1.23551911010171 5.15160554530546 5.85519822821942 8.34527498199451e-09 2.01164563159551e-07 9.58521737259464

LINC00989 1.41666899510223 0.00292309274149801 5.85363307349008 8.4194244886828e-09 2.02727447837565e-07 9.74543151468565

BMP7 -2.26781192670789 -2.74659447716632 -5.84110490292073 9.03655326532414e-09 2.15900753535242e-07 9.68851082054374

ATP11A 1.0184896265319 7.95228110634599 5.83794632491347 9.19897373191378e-09 2.18711580996615e-07 9.37369998783489

NAT8L -1.5546645325722 -1.03292353576213 -5.82865447293894 9.69345462140375e-09 2.28847534429604e-07 9.63827186499365

SLCO1C1 1.30532139625427 0.46599556556395 5.82605602069126 9.83630974312993e-09 2.31466977490497e-07 9.61203197372558

SLC26A10 1.62689180331387 -1.840871080088 5.82016286739489 1.01679443729983e-08 2.3785582629513e-07 9.54458262971604

SLAMF7 -1.14384878306538 3.76613519769324 -5.82006799609907 1.01733713548383e-08 2.3785582629513e-07 9.33772554420336

PLEKHB1 -1.14712335015105 1.83578934408369 -5.81863440046187 1.02557226957432e-08 2.39523944118286e-07 9.50854157076735

TMEM236 -1.14222261326008 -1.6352083416103 -5.8161590814232 1.0399446696009e-08 2.42437977854448e-07 9.55910990008084

LOC100129434 1.32193362811482 1.26859248808877 5.81610225861436 1.04027689307693e-08 2.42437977854448e-07 9.57059123347714

S1PR1 1.05453632227844 6.26491459804723 5.80875678249358 1.08410573295566e-08 2.50772895741189e-07 9.23443506546638

NFATC4 1.10405135034132 4.11930377799359 5.8054520302875 1.10440659263991e-08 2.54927023350933e-07 9.40484543858937

TRPC4 1.15984966598093 1.60074530917199 5.79840247805714 1.14895725243685e-08 2.64929581713908e-07 9.47832022187109

REEP6 -1.04469931525661 0.889476071359315 -5.78060052551289 1.26939819005627e-08 2.89027096265217e-07 9.36364906865462

SLAMF9 -1.69688147935231 -4.72295537311043 -5.77420365301366 1.31561244917906e-08 2.98341585260182e-07 9.3044589065415

FOXC2 1.56641083580821 3.28419263842001 5.76522437909771 1.38325887670962e-08 3.12987484088757e-07 9.27633973581332

LOXL2 1.10444119381692 6.47324122675569 5.76429026001135 1.3904877526463e-08 3.13970403649918e-07 8.98821271205079

CST5 -1.94610587082451 -5.06840698423035 -5.75491530149922 1.46510637888914e-08 3.29452175095868e-07 9.20407436346066

TPD52L1 -1.03337617448669 3.3864823217702 -5.75464776752424 1.46729202713131e-08 3.29603153297908e-07 9.02258493800293

EFCC1 1.13740785216201 1.33805754617326 5.75101070486729 1.49732236707211e-08 3.34818636220721e-07 9.22853212874918

HRC 1.35211860631563 2.63920140922353 5.74717557302179 1.52963647737715e-08 3.40445779172478e-07 9.19527861701655

DNAI2 1.18195825043055 -3.27890383577267 5.74598798407469 1.53977990472552e-08 3.42353311401026e-07 9.15461992404057

SORD2P -1.12278691997671 -0.377850570678655 -5.74102114697615 1.58291724448318e-08 3.49800605692035e-07 9.1759246819133

LOC105372273 1.09103947020891 -0.260384772382037 5.7400115722418 1.59182831321634e-08 3.51413051661056e-07 9.15083403603695

ADAMTS5 1.04509631114847 4.95856433325766 5.73544091347631 1.63278657320876e-08 3.59361631335036e-07 8.93929551125012

MIR126 1.62933848925089 -3.32351691377451 5.72785569477667 1.7030343430698e-08 3.73688997435486e-07 9.06008271531858

CXCR6 -1.03413809645435 2.3592722380471 -5.72719166725604 1.7093222518599e-08 3.74314058915839e-07 8.98780210544475

FAM163A 1.36277180380589 -0.872224709009035 5.71840875585229 1.79464830418616e-08 3.90641096372201e-07 9.02557008011271

PIEZO2 1.28709418430352 4.61713442876705 5.71558921676618 1.82290929322124e-08 3.96000664526414e-07 8.89131086914852

EBF3 1.51165771458192 1.69483011272949 5.70623481986975 1.9198093585542e-08 4.1456835622668e-07 8.99428610251395

GADD45G -1.02666714910923 1.92942526662648 -5.70333533368381 1.9508497640878e-08 4.20021234568736e-07 8.88509914784941

TNFAIP8L3 -1.23927189022438 0.998757342359355 -5.70122129013091 1.97378909546073e-08 4.23702832750432e-07 8.92941001453463

EEF1A2 -2.07116560405077 0.14362662452325 -5.69068615302803 2.09207749317381e-08 4.46453439156023e-07 8.88586510102545

PIP -2.14979408401498 -4.86497739655835 -5.69001205087202 2.09987683068751e-08 4.47678932160382e-07 8.87184604149472

IL20RA -1.95482812796639 -3.28441211216439 -5.6893428594575 2.107647345978e-08 4.48895888257369e-07 8.88230349169317

PLXDC1 1.07705703515551 5.37887900976636 5.68609106445459 2.14580679893462e-08 4.55623743457985e-07 8.63796033992928

EDN1 1.49888711357133 5.51003787567181 5.68264230919674 2.18701236949917e-08 4.63080722245996e-07 8.64759697251148

KLK1 -2.62061202226973 -2.97435507002792 -5.67873452287532 2.23463356288814e-08 4.72245327799865e-07 8.84441870122021

NR2F1.AS1 1.06472862325481 2.11725121144614 5.67355718098634 2.2992829267842e-08 4.8262769013801e-07 8.81503835872263

CHRNB4 -1.65427398386382 -3.05987501650589 -5.66201516633113 2.45002944512665e-08 5.10821752222911e-07 8.74104120564748

CXXC4 1.25789535010321 1.70899036054771 5.66074954654698 2.46713308052848e-08 5.13404261604814e-07 8.75686104463599

PRSS21 -1.58387948929632 -3.30505826348964 -5.64281842416421 2.72230721529498e-08 5.6060985009769e-07 8.63853675142711

KIAA0895L 1.13692589681538 4.47167756309567 5.62652300799248 2.9763535827919e-08 6.08321957151467e-07 8.42407743676119

SLC15A1 -2.14553402085617 0.577474914942389 -5.62372208790391 3.02228334087332e-08 6.1380608713742e-07 8.51233781815661

GLI2 1.21010574221868 1.74507028535933 5.62368324054768 3.02292518680256e-08 6.1380608713742e-07 8.564693760193

HACD1 -1.04198367923812 0.201778095588703 -5.62030935428117 3.07917822243718e-08 6.2348346388642e-07 8.54170603027546

TTLL10 1.17542776646851 -2.44402456626883 5.61910378965228 3.09952460203038e-08 6.27020000115197e-07 8.50919076421014

COL8A1 1.16629875024505 6.37892571485528 5.61620861705946 3.14892226309022e-08 6.34653619450785e-07 8.20476536796479

SLC6A1.AS1 1.40898590135988 -3.07311764897363 5.61317813608318 3.2014488032973e-08 6.44047468589393e-07 8.47647634806385

ZNF804A -1.06493499758502 -1.04704132763662 -5.61044368456622 3.24957516938893e-08 6.51921683982385e-07 8.49790214889164

TGFA 1.22850361115831 6.86692230359186 5.60830024756201 3.28779102131205e-08 6.58374858885919e-07 8.14890979221868

MSR1 -1.00427033890906 5.54942946758115 -5.60587739138702 3.33151517709213e-08 6.64684609163744e-07 8.12706365339324

HCN2 -1.38315889881828 -1.3302819455969 -5.59696513934166 3.49727042353177e-08 6.93938790419471e-07 8.42937696111493

NGF 1.4034151125535 2.43047262790162 5.59673509123909 3.50165346984743e-08 6.941756928795e-07 8.41656628491337

GFOD1 1.04562716328142 3.94522501974286 5.59266067428076 3.58017340832054e-08 7.08451223444666e-07 8.29019863989834

APOL5 1.39345348374183 -2.78244424707351 5.58725379088987 3.68702046453784e-08 7.24980799020734e-07 8.34660942978661

ADRA2B 1.37715036560282 2.19991402006761 5.58254586896808 3.78257583673626e-08 7.41092063350479e-07 8.34763275535181

LINC01010 -1.1625564811264 -2.38846037342569 -5.57979486591727 3.83952247539068e-08 7.50223390680691e-07 8.32657046035341

SLC10A6 1.44993542244052 -0.247714201078396 5.57697472509469 3.89876546730309e-08 7.60433941996293e-07 8.3117254447002

MIR122HG 1.42865159974745 -1.43315851249106 5.57649447619932 3.90894229283077e-08 7.61736319499082e-07 8.29813257135907

SYNGR3 -1.44129876851116 -1.19574525932192 -5.57521047626814 3.93627821709533e-08 7.66377173090466e-07 8.3188476304718

C4orf47 1.183218373996 2.43823136801273 5.57295149859327 3.98482297661878e-08 7.73236656922352e-07 8.29113135083885

MCOLN3 -1.61068377416363 -0.290103678119754 -5.57291003934574 3.98571934151183e-08 7.73236656922352e-07 8.29744635335139

DLL1 1.16834178905168 3.82940071788135 5.57186009264771 4.00848506281248e-08 7.75579505442127e-07 8.19815706258967

FOXF1 1.12343511695761 2.6208380553531 5.55658760661891 4.35428207420358e-08 8.35799452461986e-07 8.19615433648478

LRRC17 1.15860271558465 2.7362789119321 5.55247234399975 4.45231347219116e-08 8.53111860468178e-07 8.17177158194681

GDPD2 1.00133722975859 -1.23631302572286 5.54978122774608 4.5175765240933e-08 8.63337034240026e-07 8.16718362729486

S1PR3 1.04947000550625 5.53308842202422 5.54910543439143 4.53411092811882e-08 8.65736776950547e-07 7.90323832898137

KLHL34 -1.40858038036664 -4.62279548555032 -5.54553880879069 4.62235365481835e-08 8.8104003506507e-07 8.13891559355263

UNC5C 1.13955829244955 2.38882233837265 5.53108064467303 4.99748052950129e-08 9.44272210812973e-07 8.07480528202466

S1PR5 1.05985043747912 1.75302865417854 5.52439751043415 5.18072448285533e-08 9.75508908462906e-07 8.05436638918406

SOX17 1.05197609030802 2.49852624633141 5.50823993676254 5.65113971282471e-08 1.05950351532347e-06 7.95153378884915

MYO16 -1.30930090664217 -2.34759152299541 -5.50601697808662 5.71902309997717e-08 1.06938485234559e-06 7.95866967535952

HIF3A 1.49109005092713 1.94789943506362 5.5046169640337 5.76218187207063e-08 1.07568964673552e-06 7.95668831930025

PDGFD 1.10458922997513 6.21148048440574 5.50029885951014 5.8973006823427e-08 1.09902862973077e-06 7.60529150644511

QPCT -1.21043474853794 3.63985282395617 -5.49573479924073 6.04346173103235e-08 1.12530394781335e-06 7.6235022259749

MARCO -1.55421711500931 0.83455590615615 -5.48690876574473 6.33615977503341e-08 1.17378033892044e-06 7.82117960275436

LRRC10B 1.29508480254455 1.30256165251369 5.48600993743564 6.36672902548341e-08 1.17643964938623e-06 7.86699563251929

PNLIPRP3 -1.46767461284257 -6.00193886585722 -5.48065862664618 6.55171565518775e-08 1.20346999718542e-06 7.81269191286309

PRAMENP -1.67767834822043 -4.92918831752623 -5.47481135239295 6.75981703966286e-08 1.23751839783298e-06 7.78683121521802

PYGM 1.01538294195214 1.96159335430042 5.46759505151408 7.02550601641201e-08 1.27649573839099e-06 7.76165749774095

NMRAL2P -1.85911634091292 -2.18765708439077 -5.46567877279513 7.09774676846645e-08 1.28747211591008e-06 7.76392438454229

MIR210 1.4763028217483 -2.12827779629213 5.46233736586846 7.22544226799599e-08 1.30736659889833e-06 7.72575055814838

TMEM52B -1.35225265948645 0.685178997449058 -5.44954203692625 7.73538355503154e-08 1.39268894824129e-06 7.64297359427901

SEZ6L -1.3448838911092 -2.3271598836212 -5.44217018275182 8.04487218461892e-08 1.44363341172795e-06 7.64179617836769

LCN2 -2.07533834292612 -1.81333467877198 -5.43269880018374 8.46019563579189e-08 1.51317237801382e-06 7.60122361275798

H2AW -1.30902618712948 0.518917478748394 -5.43244207463782 8.47173808344956e-08 1.51399279854225e-06 7.56631892366103

RNU6.796P 1.36224879646715 -2.34258541447419 5.43180717735461 8.50034893781074e-08 1.51785968276724e-06 7.57496015990782

IRX3 1.24203727418286 5.74248689623164 5.42031433822754 9.03477588136546e-08 1.60625230676325e-06 7.2387637934047

ADAMTS9.AS2 1.17731982090562 2.7052626228937 5.41917482745895 9.08950789411718e-08 1.61379541868881e-06 7.49880011486872

H3P25 1.18187606625214 -4.20226086910214 5.41210664244923 9.43626199177917e-08 1.671270258544e-06 7.4748614166034

ALDH1A3 -1.10307258621937 3.04612660470227 -5.40643422800458 9.7237982271313e-08 1.71660921338173e-06 7.23421271216879

LINC01844 1.24755138375407 -3.12006393606235 5.39606591850776 1.02715051782056e-07 1.80161042074136e-06 7.39816196082013

C19orf84 -1.01691795465579 -2.61908225732891 -5.3944410201397 1.03600109874998e-07 1.81567116879958e-06 7.39999946242221

TSKU -1.19706266861378 4.70528898763801 -5.38396158090107 1.09488501089878e-07 1.90658896257869e-06 6.99222891117724

ITLN1 -1.39689413034924 -3.27406070725793 -5.37943637391688 1.12130466614701e-07 1.94481583012127e-06 7.32401824377114

RUFY1.AS1 1.08471292596386 -1.11829652469138 5.37236921787632 1.16380696356062e-07 2.00416029872025e-06 7.2901856117785

SLIT3 1.08829009954201 6.56252992049043 5.36619965982455 1.20218578613793e-07 2.06209440558426e-06 6.90417420233398

ERRFI1 1.17643806133468 7.35131463772364 5.36546879742991 1.20681279208964e-07 2.0684011059382e-06 6.88682156406417

OLR1 -1.05964310811149 3.92276580159557 -5.36385124130612 1.21711487894154e-07 2.08257137314874e-06 6.93824447526822

DIO1 -1.3812070403684 -0.930872509810436 -5.36189652958083 1.22967818071114e-07 2.10097370169068e-06 7.24927384500991

F8 1.05751484515278 5.98971536716749 5.3561589980749 1.26728538521523e-07 2.15507820156093e-06 6.87953420227328

ASCL1 -1.54782169232803 -5.17281053019011 -5.3555727279718 1.27119039638665e-07 2.1600313316275e-06 7.20188722212179

FOXL1 1.21291310959339 1.78767028485548 5.34835802846405 1.32021205794312e-07 2.23460776557137e-06 7.17514481265222

ST6GALNAC3 1.11473301614053 3.10865216494459 5.34308928643427 1.3571651644676e-07 2.28825825987345e-06 7.09116669341795

MMP12 -2.18926135736488 -2.99392701065417 -5.33760642178645 1.39668488020685e-07 2.34761697200483e-06 7.12912815187878

FAXC -1.83868982297724 -1.97830621922416 -5.33127107741667 1.44373996829132e-07 2.42110076192582e-06 7.10145499352804

SLCO2A1 1.17684894655015 6.83797956679002 5.33020881241741 1.45177890176695e-07 2.43270757157515e-06 6.71612200982598

MTCP1 1.06807028300266 2.83065727327245 5.32968147820479 1.45578573293641e-07 2.43754523452514e-06 7.04077849944739

LINC02274 2.33996578862617 3.05769097395824 5.32471591280317 1.49404527948266e-07 2.49011359865995e-06 7.05316189616865

DNTT -1.65107957209198 -5.45609343263171 -5.32187400688922 1.51637898822008e-07 2.52347258689499e-06 7.03856125134142

BARX2 1.52538303661582 5.12489360967886 5.32003945750336 1.53096778253862e-07 2.54192034496705e-06 6.82112297056384

LINC00487 1.13582590261313 -1.96794156213369 5.3171684919106 1.55407198686041e-07 2.57439002572226e-06 7.01867620009111

ANKRD44.AS1 -1.24027932120341 -3.49112799769712 -5.31489681205403 1.57259269697154e-07 2.60308937148133e-06 7.0090621474985

SYN3 1.03622088265697 -0.92301331911045 5.31280741010045 1.58981616429499e-07 2.62163094304614e-06 7.00293927098042

CCNO -1.86736933788986 -1.92818004992848 -5.30532239327461 1.65303127048315e-07 2.71353934122223e-06 6.97495176733673

TRIM9 1.3671368635614 3.64511706207156 5.30258093235307 1.6767887889942e-07 2.74632517457011e-06 6.87620913241751

RAMP3 1.07742171608842 5.78742907250353 5.28628150856584 1.82502588161924e-07 2.95575434264926e-06 6.54052098883215

CSP2 1.01014311233735 -2.48717939245459 5.28523234204757 1.83499094605219e-07 2.96747755740848e-06 6.8639365695696

LINC01443 -2.15801569594809 -4.4896031291992 -5.27538102982961 1.93116903288481e-07 3.09998203088523e-06 6.81855147517757

CDH8 1.64570716746295 0.341009894636293 5.27010290072025 1.98469141075018e-07 3.17419382349737e-06 6.80287429939678

IZUMO1 1.21200398768994 -2.3140570630983 5.26969321854702 1.98890535988743e-07 3.17859786847795e-06 6.7895948920893

KCNAB1 1.10990764163593 3.57030382279716 5.26708348137397 2.01595313188865e-07 3.21474372321028e-06 6.68002432550922

PLPP4 -1.52791030504407 -1.57679791241518 -5.26640751516278 2.02301688714742e-07 3.22364630911697e-06 6.78602278887039

LINC00871 -1.3417924883544 -5.77349525329199 -5.26607526525228 2.02649764093358e-07 3.22683058889549e-06 6.76983729387352

PPP4R4 -1.51038958797581 -3.26552479075142 -5.25065310288084 2.1946108545203e-07 3.46914266306053e-06 6.70310800639587

TMEM91 1.32833504159741 5.0145950022206 5.25015469398728 2.20026334061186e-07 3.47555385595779e-06 6.466853703825

ADSS1 1.5729756567875 5.58001755832612 5.24674966000684 2.23925919899026e-07 3.53202572350876e-06 6.40806263160151

BIK -1.07686080243132 -1.49973983178317 -5.24402247837778 2.2709742030185e-07 3.57686653235194e-06 6.67882948135349

TRPM6 1.08473880852416 -0.204305277252591 5.23865538251756 2.33466533643911e-07 3.66921735583178e-06 6.65133593605712

HOXD10 1.23037601543707 2.94751586823964 5.23638664229348 2.3621057618884e-07 3.69733992041938e-06 6.583001096103

HMCN1 1.08384656590179 4.75798676443762 5.22550501748106 2.49812187627549e-07 3.88682050613928e-06 6.34959076688939

CCL3 -1.03310887824268 2.38641363179461 -5.223478411919 2.52427838097974e-07 3.91353045005598e-06 6.40520637856166

SLC38A8 -1.5222988891568 -5.61142744285719 -5.21315538299736 2.66168464624078e-07 4.10608715058278e-06 6.51878545088861

SLC6A1 1.13321316756316 2.63010474256256 5.20854009779708 2.72543519641332e-07 4.18663005789193e-06 6.46000449426102

PGF 1.64322807986745 6.27586154801971 5.20626833474534 2.7573555439029e-07 4.22671536085453e-06 6.15008271215625

KANK3 1.04017112547738 3.58598156842998 5.20319425784042 2.80112613243783e-07 4.28475843462925e-06 6.35756814893317

C4orf48 -1.11417859398377 -0.492309113489886 -5.19899682090845 2.86197991755946e-07 4.36251518666084e-06 6.45376064213725

HID1.AS1 1.21440660065939 -1.55865541423342 5.1963962906978 2.90032223670311e-07 4.41786662885351e-06 6.44319947783582

LIPH -1.65397493242729 -0.226850944545048 -5.18548939176907 3.06662256182554e-07 4.63549814605948e-06 6.3694526812877

COL23A1 1.63266107642417 7.1202733882327 5.18361276853333 3.0961518223134e-07 4.67688665623149e-06 5.99384385176812

EMCN 1.23287672437678 5.70219323487097 5.17721960317875 3.19883120288483e-07 4.80531116585191e-06 6.02113642160762

GLRB -1.67405261678006 0.356093577643882 -5.17638612951928 3.21245797895595e-07 4.81912976071221e-06 6.29969789037277

RN7SKP70 1.06800445166922 -1.94185484487447 5.1760761803906 3.21753977645712e-07 4.82342894725497e-06 6.34656652402704

ZNF503.AS1 1.0354904008345 0.665287687563661 5.17326399739905 3.2640042142213e-07 4.88299517051237e-06 6.3391005992335

SYT14 -2.08230308694409 -4.388241442274 -5.17214390914761 3.28269125457714e-07 4.90757833367999e-06 6.32846702077173

GJC2 1.27246353621149 1.18342713964584 5.1704395882055 3.31132421385391e-07 4.93682151801083e-06 6.32232250337195

PPFIA4 1.81216483694048 4.28762237665451 5.16698779004864 3.37005773600766e-07 5.01065893030592e-06 6.18550781385535

FSIP2.AS2 1.38268241651464 -2.88559665952888 5.16626755624617 3.38243930141439e-07 5.02220711281631e-06 6.29818390293259

SYT3 -1.34739464856007 -2.50524436985175 -5.16454023018352 3.41231353874477e-07 5.05966136225187e-06 6.29813149343557

SGIP1 1.30878418404568 3.05393403835134 5.16430387702104 3.41642109782576e-07 5.06230347422555e-06 6.2314704868019

ACOD1 -1.26856071800315 -5.09199631498188 -5.163364250604 3.43279816375847e-07 5.08311004289324e-06 6.28459324812302

GRIK3 2.1297241473049 4.13405829963274 5.15731120820293 3.54013485112233e-07 5.20620392847245e-06 6.17108323443432

IL1RN -1.04886391921796 0.953605941282569 -5.15653886458491 3.55406208692312e-07 5.22005866707528e-06 6.20296206077504

CCDC188 1.00008496477666 -0.963512993423031 5.15506289801012 3.58082501646645e-07 5.25227884996127e-06 6.25118166976547

PRRT2 1.17651714594396 1.43637257483928 5.15345749754809 3.61015636575047e-07 5.2881745365606e-06 6.23681111175969

LRRC52 -1.41522638476051 -5.97087110513124 -5.14239048938325 3.81877641874143e-07 5.57126717873624e-06 6.18546232975169

OVCH1 1.04023405165956 -2.48327883343395 5.13744475362924 3.91573495885149e-07 5.69324562099365e-06 6.16429372017497

KIF26A 1.51642908664287 3.0433048712632 5.13397345883757 3.98520617522325e-07 5.77536503435982e-06 6.09493158155534

PRR29.AS1 1.04520903405857 -3.68890511564339 5.12800541955395 4.10744218444773e-07 5.93276005500157e-06 6.11850209143881

PAK6.AS1 -1.70959309506814 -4.00113356642556 -5.12492508344628 4.17194445739427e-07 6.00997452045672e-06 6.10735183031934

KRT20 -1.60055669465674 -4.88567863411503 -5.12315988788413 4.20934825982921e-07 6.05319206957088e-06 6.09733519089956

KLK15 -1.89365136751555 -5.69062402613762 -5.12236615839636 4.22627263064576e-07 6.07127379892083e-06 6.09288795369871

TLR8 -1.03188798196922 2.26583075043892 -5.12226461080956 4.22844263295998e-07 6.07127379892083e-06 5.92842443269249

KIF5C 1.03850507334573 2.24609563831564 5.1205108314467 4.26609021066503e-07 6.11725860444965e-06 6.05179416827996

BRSK2 1.24808038123774 -0.86611001898878 5.11860329815151 4.30740652076067e-07 6.16432069279405e-06 6.07994192497676

LOC100506474 1.14491003816269 -1.074035916525 5.11674983988484 4.34792225614287e-07 6.2182144382038e-06 6.07084828705511

LOC105369519 -1.29263357866675 -2.38134988306401 -5.1162152682877 4.35967608184204e-07 6.23093035282046e-06 6.07123821122338

ADRA1B 1.07983427594321 2.93433824635529 5.1096208861799 4.50722167990193e-07 6.41652676955038e-06 5.96495169332821

RNU6.1016P 1.11340062768331 -2.71964043730719 5.10366816739923 4.64454988154725e-07 6.58189565570566e-06 6.00645395631835

PREX2 1.3700047904382 5.22740350303987 5.09977873669007 4.73645525379637e-07 6.69035830690367e-06 5.70832514559012

ASXL3 1.98084660369232 1.49240592181451 5.09838486426953 4.76981798432897e-07 6.72858229557871e-06 5.98458202319407

NNMT 1.2095312069733 9.02336694867087 5.09430051207928 4.86889215183466e-07 6.84190932659683e-06 5.56570130353761

APLNR 1.27834920279385 5.62397558131968 5.0824468208282 5.1678283133987e-07 7.19693658974725e-06 5.57104070650892

IGFN1 -2.36012775175748 -1.87679329322989 -5.07822764845323 5.27845409629878e-07 7.33223422553514e-06 5.88584925076481

IL10 -1.06653047722298 -0.677062841823123 -5.0741656324196 5.38712074646355e-07 7.46412840791039e-06 5.86631766729553

TTLL7 1.04331942675158 3.11901724856579 5.07296647040299 5.41961202621878e-07 7.50436990933232e-06 5.77767919928497

PTGDS -1.30964032549606 2.97999366494858 -5.06827251458159 5.5486267441953e-07 7.66351258508244e-06 5.54711413172307

TRIM67 -1.18597229938267 -3.51287143599877 -5.05427613282237 5.95119360858969e-07 8.12670208771467e-06 5.77970100499695

ALDOC 1.27278938088633 6.04994235358431 5.04960919270655 6.09159560978615e-07 8.30800511517639e-06 5.38070080822108

ITGA8 1.12649718100655 4.7550099364085 5.04598057491526 6.20296751097365e-07 8.43196223104739e-06 5.48060918531752

LINGO1 1.07585129971887 3.89604774775679 5.04541872618989 6.22038715603888e-07 8.44661055679964e-06 5.57289320010951

DCLK3 1.33187432983843 -0.642907055408517 5.04521314555942 6.22677281773151e-07 8.45001021967343e-06 5.74000761095923

IGFBPL1 -1.37153139007486 -5.11678814843834 -5.03909223458834 6.41982740939417e-07 8.65265530775746e-06 5.70790062892281

MLPH -1.0606334110388 1.68546686648411 -5.03560335966608 6.53244222414039e-07 8.7935479216366e-06 5.5718034731681

LOC101928940 1.27000430644344 -2.61154198728013 5.03207787344694 6.6481770402597e-07 8.9193309805087e-06 5.67603992903518

YWHAEP7 -1.53943070027551 -0.685958346230507 -5.02658704013369 6.83238643012122e-07 9.11277913140004e-06 5.63411885001677

CNKSR2 -1.17574644154284 -2.7219288179408 -5.01604827249797 7.19986038294865e-07 9.58528201563567e-06 5.60593958362083

SOX7 1.00905477521396 2.92519007134876 5.00971517633374 7.42979687743845e-07 9.86124320921968e-06 5.48596434445124

ST6GALNAC2 -1.0098017840368 1.97361718730268 -5.00707570264216 7.52770890187154e-07 9.96688805760571e-06 5.41309982975991

HOXD4 1.02008312543164 0.823391142963976 5.00370968257412 7.65438200196469e-07 1.01161465110362e-05 5.54133534367477

EPHA3 1.31909157715949 3.69923003178215 5.00106942032293 7.75518134410683e-07 1.02237349395597e-05 5.40320287149601

RN7SKP275 1.28200003774562 -2.98443385818331 4.99393696166937 8.03393507154475e-07 1.05409683364867e-05 5.5013602716701

HTR7 -1.03334882909998 -0.312128093984936 -4.98813303949967 8.26788473131018e-07 1.08218308446439e-05 5.45782116556877

CFAP61 -1.00162892114514 -1.87242164236243 -4.98246048247871 8.50288963801971e-07 1.10674978581498e-05 5.45260585807027

MGAM2 1.66838836798942 -0.336065825225824 4.98226257850197 8.51120384874205e-07 1.10674978581498e-05 5.45147311480056

COX4I2 1.39845739739637 2.94093514076419 4.98060047097661 8.58134175477072e-07 1.11449920033469e-05 5.37042408359298

FCRLA -1.23825984296295 -1.54130511668001 -4.96792523768317 9.13488006303242e-07 1.18104884364421e-05 5.38303368824754

TRPV1 1.03460957620834 -2.36179417198464 4.96302115247401 9.35816511120864e-07 1.2046078058881e-05 5.36177274866587

F2RL3 1.59482575195048 4.29386875238232 4.95418530099281 9.77383409829698e-07 1.25310248536758e-05 5.1451776186818

HSF4 1.6178245415646 5.43545772836944 4.9518601837643 9.88614396622034e-07 1.26508933399599e-05 5.00856636365623

SPTBN2 -1.48108782219041 1.54194532759323 -4.9449775371841 1.02259455785145e-06 1.30092435656063e-05 5.11812961265748

PLA2G4B 1.17518207550376 -1.54615556571264 4.93612507464035 1.06796036848728e-06 1.35310205709328e-05 5.24096292516389

LOC101926956 1.66198796905787 -1.17026583536937 4.93327001420136 1.08300140472682e-06 1.36738350212811e-05 5.2281750374608

ENPP2 1.21898418801324 8.09548047390666 4.93305859840594 1.08412327921857e-06 1.36758068335889e-05 4.7812711854473

TMPRSS2 -1.85328502232914 -0.578552853630704 -4.93300382045327 1.08441414043159e-06 1.36758068335889e-05 5.18715322219142

CREB3L1 -1.28074787629696 1.80595052875784 -4.92752953916624 1.11386456620135e-06 1.40066377888532e-05 5.02289511532256

RPL36P7 1.02686972909617 -2.80449652679974 4.92372754331625 1.13477105231891e-06 1.42530649139213e-05 5.18404499881974

GYG2 -1.76180938251903 -1.13116280230936 -4.91882481373814 1.16228979340635e-06 1.45567099730012e-05 5.1441900058445

CHRM3 1.58629727426658 2.0443746139031 4.90745231560348 1.22862450275515e-06 1.52732550265399e-05 5.08222183023853

LOC105376291 2.04198989039614 -1.48218980798338 4.90268541043843 1.25750271487191e-06 1.55922721505201e-05 5.09014532916999

OR51E1 1.12190378987905 2.67986409657305 4.90234834415298 1.25956928474463e-06 1.55922721505201e-05 5.01222315853386

RPL37P11 1.61016120508214 -2.98769280116867 4.9002151164192 1.27272438815445e-06 1.56959726668317e-05 5.07814396050353

LRRN1 -1.37340491073809 -2.15872582356037 -4.89901643210778 1.28017444297708e-06 1.57699813810312e-05 5.07363772067097

MSI1 1.10843985606537 -0.0104851239660006 4.88889466102819 1.34478293580467e-06 1.64912057260057e-05 5.02762529360365

NALF1 1.15328830633948 0.435128487682689 4.88606662652246 1.36338955449834e-06 1.66817877643425e-05 5.01158309314837

PTX4 1.04765885400632 -4.09018084577238 4.8790737551246 1.4104702746702e-06 1.71613786857162e-05 4.98344660276726

SAA1 -2.49605617720234 0.619176030930211 -4.87785051569763 1.41886547093611e-06 1.7244246066927e-05 4.82691196930352

OSTM1.AS1 2.99900895345883 0.629354096282293 4.8766952710455 1.42683824206934e-06 1.73218003430693e-05 4.97476777566865

ERVFRD.1 -1.09369178070284 -2.30238411286678 -4.87621632034323 1.43015631089972e-06 1.73507451304008e-05 4.97235119488462

C2 -1.25097980490064 4.73189898576951 -4.87438082388548 1.44294127180057e-06 1.74766351440478e-05 4.51486869450989

GREP1 1.11771831993751 -2.20472796564336 4.86822470306624 1.48663074689979e-06 1.79376338513125e-05 4.93610395667227

HTR3A -1.58206184182012 -4.20173235528478 -4.86038190478289 1.54414064114172e-06 1.85390564455223e-05 4.90135280451415

RNU6.339P 1.29074905313609 -2.05103363455382 4.86007179476225 1.54645815003592e-06 1.85566452876692e-05 4.89985974113055

RHCG -2.24681907675653 -2.08321706768453 -4.85919069000335 1.5530611204442e-06 1.86256095915751e-05 4.88224597959269

GDF6 2.05502693642311 3.04084076764468 4.85423647718163 1.59069689927321e-06 1.89828395868859e-05 4.81570709005614

TLL2 -1.02696535359988 -1.33582771658831 -4.85359199819948 1.59565698149597e-06 1.90211749815021e-05 4.86496333193986

HES5 1.45434007931762 -2.41567513795805 4.85075308917371 1.61768403149821e-06 1.92521204557799e-05 4.85820081250083

DOCK3 -1.02513092426968 -0.893328755394135 -4.85015759034964 1.62234154460961e-06 1.92861876062337e-05 4.84154146076487

LOC613206 1.08996106356385 -1.39170767880571 4.85004741625559 1.62320465358663e-06 1.92861876062337e-05 4.85576841346384

NEUROG3 -1.13559236783292 -5.25607045702233 -4.84113120425401 1.69454143578615e-06 2.00462409960637e-05 4.81524825084032

NR2F1 1.31039942534891 4.58723952074228 4.83862554581301 1.7151265416184e-06 2.02567332780291e-05 4.54885532207773

GPR17 1.26393083879593 -0.419979697877756 4.83649355691018 1.73283137041458e-06 2.04325787864649e-05 4.79537318405789

STK33 -1.70207470855459 0.332770271126689 -4.83557565567828 1.74050808668417e-06 2.05017944510272e-05 4.70305126268632

STARD13.AS 1.07243513189263 -0.821743250366077 4.83151824641892 1.77483597869563e-06 2.0792709767636e-05 4.77363305016914

FOXN1 -1.24033450216616 -4.37551819186265 -4.82283536135191 1.8505036232222e-06 2.15515850008976e-05 4.73480799134738

ELF5 -1.56128689454907 -4.17717571647479 -4.82024161295553 1.87370361878731e-06 2.18101105187932e-05 4.72354205176431

IGF2BP2 -1.286653319096 1.14073502102269 -4.81588818522287 1.91327450527991e-06 2.21876644413574e-05 4.58389524600476

SLC30A2 -1.74684198426551 -0.880342160862904 -4.8152722065107 1.9189381134451e-06 2.22296572194569e-05 4.66856993595446

SOX18 1.04884723131337 4.21442220742494 4.8096132382204 1.97173082802155e-06 2.27633805118796e-05 4.43293779875539

SLC7A4 -1.46754125287528 -4.04131259114992 -4.80954971449002 1.97233131094799e-06 2.27633805118796e-05 4.67642209348699

GAL -1.40934527042453 -4.87476341512771 -4.80874933182231 1.97991235163603e-06 2.28266695752444e-05 4.67260575651015

VWC2L 1.15313548344055 -3.23909931880918 4.80387748328759 2.02666640719636e-06 2.33039871555431e-05 4.65099917694319

NPTX2 2.41315019034232 5.83767108536041 4.79847126741445 2.07979346508379e-06 2.38267707128836e-05 4.33990322018935

FRZB 1.36078483714744 6.1007151346792 4.79678711689218 2.09661592974413e-06 2.3969033058162e-05 4.20015981135198

IL22RA2 -1.18591378191868 -5.59858192420671 -4.79607393418782 2.10377912583127e-06 2.40005032662313e-05 4.61662194544889

HCN4 -1.34510851329505 -2.73439600624285 -4.79167336036455 2.14850264754014e-06 2.44466581960304e-05 4.59740002373604

MIR17HG 1.33552110960092 -1.52407322113503 4.78673455266309 2.19978683509474e-06 2.49259552522162e-05 4.57619622865345

C10orf71 -1.34567621030762 -5.88357737390528 -4.78463775391281 2.22191451842238e-06 2.51505009477379e-05 4.56647681558512

IL23R -1.10511322410745 -3.50874227095278 -4.78412308970919 2.22737848498632e-06 2.51861545364661e-05 4.56473471043707

ADCY5 1.20141985552071 5.64928932574882 4.77573811564251 2.31824185354013e-06 2.60781242511669e-05 4.13246185301631

CUBN 1.62828208743388 7.86686205817406 4.77553653238544 2.32046964137992e-06 2.60897017995438e-05 4.05105761696598

AQP1 1.15671967762578 8.86227879927793 4.77344135992683 2.34374672898833e-06 2.63169895037745e-05 4.05837792005934

CLGN -1.72400393494322 -0.45920584628221 -4.77143779144932 2.36621629124321e-06 2.65218486155978e-05 4.45835829420784

GABRG1 2.04327098254931 -3.24362507459873 4.76569260716805 2.43180318536574e-06 2.71451589414646e-05 4.48367700804671

LOC100129066 -1.12275709628786 -2.97323079846252 -4.7624119517142 2.47003646517543e-06 2.75154983303345e-05 4.46962031215879

PDE1C 1.07498568132926 3.13947717390523 4.76118984002175 2.48442655212844e-06 2.76475014111349e-05 4.32976565586481

LINC01359 1.04542141114049 -0.564519200684207 4.75676375268462 2.53722116629919e-06 2.81773944524666e-05 4.44377728305977

HMGB1P43 1.51391028633032 -0.654380613349308 4.75529178217118 2.55501698319811e-06 2.83172376136829e-05 4.43836944507619

TRIM50 -1.54284165997595 -2.51855296147133 -4.75178614564415 2.59788451278046e-06 2.86900822880225e-05 4.42022898426157

LINC01537 1.12119508109593 -0.496863569022151 4.7483698456535 2.64032513775234e-06 2.90997252017495e-05 4.40701928764263

ALPK2 1.47865866551891 5.93651069376697 4.7347359991915 2.81643989960988e-06 3.07902484976536e-05 3.95148554706244

CCDC26 -1.0694477040423 -4.40838903868489 -4.73442480912233 2.8205887584105e-06 3.07902484976536e-05 4.34788911685247

LINC00976 -1.0694477040423 -4.40838903868489 -4.73442480912233 2.8205887584105e-06 3.07902484976536e-05 4.34788911685247

LINC00977 -1.0694477040423 -4.40838903868489 -4.73442480912233 2.8205887584105e-06 3.07902484976536e-05 4.34788911685247

MSTN 1.17958874298165 -0.983587227646101 4.73076109042838 2.86987864496682e-06 3.12639717055947e-05 4.33160690269536

CCDC185 -1.08479593843373 -5.91712262323771 -4.72934575371252 2.88914095094572e-06 3.14093357900189e-05 4.32571285182949

ST6GALNAC5 -1.60331249185271 -2.99815459506606 -4.72926175633734 2.89028803176404e-06 3.14093357900189e-05 4.32439469657958

HCG27 1.10437871917258 1.20659529715451 4.72851794818005 2.90046472671758e-06 3.14884866366391e-05 4.29486844116576

DGKI 1.0989728037388 2.58214538250509 4.7265059552366 2.92816563122473e-06 3.16943716036145e-05 4.22087639639301

CYP11A1 -2.04915102727902 -2.83557895151796 -4.72481908175089 2.9515863283785e-06 3.19002877903748e-05 4.3008632564763

COSMOC -1.08850044491413 -0.658561875230158 -4.72441113793137 2.95727726045425e-06 3.19459325698797e-05 4.27621054660058

TEK 1.04836243807522 4.87276515679567 4.71210364094592 3.13402034608825e-06 3.35060023935673e-05 3.91036270672706

LOX 1.38808715850864 7.37284087544702 4.70750798592925 3.20258713362386e-06 3.41245261403184e-05 3.74470338603896

ARHGAP33 1.07386747071802 3.12081169138306 4.70132562364076 3.2971069550227e-06 3.50088424829166e-05 4.07222151405124

PLCXD2 -1.09896043788087 0.592776228738488 -4.69922452031667 3.3298364921195e-06 3.53219059083651e-05 4.10768891720848

NR0B2 -1.96043222110798 -4.7637811136655 -4.69820559035807 3.34582096682854e-06 3.54396520608063e-05 4.19125566200167

LINC01348 1.07758481741048 -1.51745276765875 4.69800049958977 3.34904723973307e-06 3.54565716280495e-05 4.19014445759794

TPSP2 -1.56465439571046 -5.41861535215611 -4.69627012809374 3.37638699560979e-06 3.57112807256746e-05 4.18297854029558

VTN -1.22485272433538 -0.770825813886757 -4.69563962640208 3.38640213110689e-06 3.57998131072383e-05 4.15026576659385

PTGES -1.19740685477276 1.82092062221583 -4.68739567730537 3.52000982691737e-06 3.70145187934833e-05 3.94589111925083

LINC02072 -1.19479898029264 -1.79100943946074 -4.68473811458126 3.56415032654559e-06 3.73883663411652e-05 4.12488451931735

LINC02073 -1.19479898029264 -1.79100943946074 -4.68473811458126 3.56415032654559e-06 3.73883663411652e-05 4.12488451931735

FBXO40 -1.18194583164732 -4.61995863864348 -4.68045807374802 3.63636004480165e-06 3.8054158218845e-05 4.11498630310356

SLC6A3 3.81445285831878 5.80290679278854 4.6690274206158 3.8361702064429e-06 3.98577168895668e-05 3.91435994083996

LINC00517 1.19147908854206 -3.28991113248847 4.66668987882274 3.87831102119374e-06 4.01996171420592e-05 4.05602850689218

DLK2 1.01412546607661 0.518005695962973 4.66277479441277 3.9498900925159e-06 4.08662058864518e-05 4.02255482504382

GSDMC -1.56677274815753 -3.66516830225192 -4.66233811427568 3.95795213304729e-06 4.09082355555747e-05 4.03681495564944

GALNT15 1.38582524079231 4.37312702340307 4.66197912519367 3.96459167532589e-06 4.0957411958623e-05 3.79072035337628

FCN3 1.31202699797926 4.06785573542179 4.66054052805086 3.99130625215409e-06 4.11942926461063e-05 3.80996607320538

MPO -1.19895405115801 -2.69074941496014 -4.65738277702263 4.05055332627746e-06 4.16607863291992e-05 4.01414123182273

SCN4B 1.16532164106072 4.70915332335779 4.65045680419385 4.18347571030775e-06 4.28324157037953e-05 3.66754444770096

OPCML 2.40951042349588 1.14699929233367 4.64908015181373 4.21039104303573e-06 4.30472436983366e-05 3.97171631750921

AGAP11 1.17047816635912 -1.64304513590277 4.6482437020078 4.22682590290175e-06 4.31949856471655e-05 3.97670847117793

KCNG3 -1.29125413242575 -5.31489410507871 -4.6322025470177 4.55419626213658e-06 4.61934715926966e-05 3.90894114982266

LINC01235 1.77343583432561 2.30795190221922 4.62901342721388 4.62212121462923e-06 4.672557355742e-05 3.8410022650353

C11orf96 1.04634772772796 4.97083597581684 4.62897877147238 4.62286466427293e-06 4.672557355742e-05 3.53114723413548

FHL5 1.29839728830848 3.31103345260718 4.62751578307006 4.6543543045876e-06 4.69904128701105e-05 3.74399624794308

INPP5J -1.12936908445879 0.575333510185804 -4.62517959306965 4.70506653943834e-06 4.74363980379594e-05 3.78121986871021

PCSK6 1.38536862025177 5.86359838319461 4.62298949620685 4.75308919323102e-06 4.7756698741733e-05 3.44505591486009

KCNH2 -1.18965134983565 0.343302815716501 -4.62087682591632 4.79986000001991e-06 4.81246211978044e-05 3.77807046364206

FSIP2.AS1 1.01784267901528 -2.7424656832676 4.61952151063828 4.83009670930765e-06 4.84054857603589e-05 3.85495291745144

CABP1 1.62861728627831 2.75386852865584 4.6183264586199 4.85690969377962e-06 4.86294173433767e-05 3.76755652481603

NT5DC4 -1.16279329497358 -3.68251005766073 -4.6134471220149 4.9678766394418e-06 4.95808210961621e-05 3.82875696514639

STPG3 1.52808450486941 -1.1580059791798 4.6119076740862 5.00338954036568e-06 4.98438352975468e-05 3.82181892329826

LINC02783 2.33714138721367 0.819927778241428 4.60413720243963 5.18639643309422e-06 5.14315677262697e-05 3.78314697638918

TSHR 1.12186359719704 -0.296446980510304 4.60146060612443 5.25090995695617e-06 5.19660298895577e-05 3.77276203091451

SH3GL2 -1.88100330859495 -1.99812327819846 -4.60140621597684 5.25222886741521e-06 5.19660298895577e-05 3.75842814555004

TNFAIP6 1.96557704494752 4.78281913854719 4.60023399156687 5.28073164295083e-06 5.2200583865627e-05 3.54206813901531

LRRTM2 1.04249472483163 -1.18967928393135 4.60011449643941 5.2836455084235e-06 5.22056794288943e-05 3.77136123897671

PITX1 -2.00412255127172 -2.64781344470791 -4.59171777634107 5.49230890708635e-06 5.38518414326796e-05 3.72713717539623

BDNF 1.51394618478007 1.30756369474909 4.59151607722205 5.49741727446296e-06 5.38645794787037e-05 3.70640970979251

SAA2 -2.29707377913699 -1.22579213146417 -4.58480268235522 5.67007165505521e-06 5.5345493146003e-05 3.65493384957716

LINC02898 -1.09523994261098 -3.72297376344235 -4.58247413498757 5.73116375884305e-06 5.58666554136752e-05 3.69792061807251

ANKRD2 -1.46663811656199 -1.55666499516965 -4.57532702800373 5.92264727766827e-06 5.73990486611777e-05 3.64660611135283

MARCHF4 1.23602445589815 -0.0280461317064994 4.57434891917699 5.94932540888052e-06 5.75806874944874e-05 3.65574828091598

TRAV36DV7 -1.09432354476905 -4.00143697747281 -4.57331042256841 5.977776850165e-06 5.77789825477538e-05 3.65955605236472

ALOX12B 1.34227007229256 -0.887249362501345 4.56512514829947 6.20664289007658e-06 5.97259044156927e-05 3.62352899740358

CYP17A1 -1.34023073660837 -0.2713434596843 -4.56402682254864 6.23798534676335e-06 5.99480031094913e-05 3.55566021659045

HTR1F 1.253071720509 0.285032653828081 4.55662965822517 6.45306532426568e-06 6.14990687010907e-05 3.57653991968835

MTTP -1.75888048614657 -1.14193295410074 -4.55551532124522 6.48607617297386e-06 6.17325841963804e-05 3.54352016034201

ARSI -1.48209748265446 -1.63170640704531 -4.55514249382322 6.49715684856237e-06 6.17840162178493e-05 3.56240740241182

RNF165 1.15573148685643 2.30118828654077 4.55425662587238 6.52355831833723e-06 6.19538804167742e-05 3.49401403408202

CCDC60 1.00782673718492 -3.33926169430512 4.54896526850863 6.68341275432472e-06 6.333384650561e-05 3.55807165634382

DLG1.AS1 -1.09366711086657 -4.12785883260535 -4.54125531523084 6.9230770095361e-06 6.5320596994613e-05 3.52531214218104

LINC02367 1.04472976577666 -2.49468092314176 4.53988541771167 6.9665139471148e-06 6.56511192529087e-05 3.51970142359806

FER1L4 1.7591317944191 3.66670257159919 4.53984228358369 6.96788588730894e-06 6.56511192529087e-05 3.37321497923176

CASP12 1.08821393534278 -0.319356946405469 4.53885519713426 6.9993524415907e-06 6.58974500848204e-05 3.50814447724199

VSNL1 -1.46277784340968 -3.38951222430417 -4.53810183006797 7.0234601017237e-06 6.60958305379247e-05 3.51072063527239

ADTRP -1.02248677812329 0.73769144594271 -4.53680754657621 7.06506308115408e-06 6.6372562834476e-05 3.40075345304114

RN7SL124P 1.2492595271328 -4.28915520335112 4.53621481829304 7.0841943344029e-06 6.65235798433771e-05 3.50514922767045

PDE4C 1.25748994680929 1.13944379335624 4.53607011798631 7.08887230815277e-06 6.65388027302981e-05 3.46956200422119

DUSP15 -1.07648818160661 0.936233566569159 -4.53281900339472 7.19476108759699e-06 6.73584363843972e-05 3.3586282082469

STRIP2 -1.16680823713215 1.0526304477139 -4.52309405957744 7.52063433899341e-06 7.00477739224945e-05 3.31298047436246

MMP16 1.22660185839657 1.88695226791465 4.52118079418386 7.58639096168581e-06 7.06300137138644e-05 3.37826156078287

TCHH -1.0600430222798 -0.750745751973486 -4.509492707072 8.00022686679959e-06 7.38824515102361e-05 3.35632838231736

RPL21P13 1.35136787051717 -4.05818381444082 4.50678173559803 8.0992598703143e-06 7.4638691616059e-05 3.38284165152871

MTMR9LP 1.1305331704017 2.67022257384313 4.50316932793824 8.23305036291141e-06 7.57433673920087e-05 3.24838125009149

FOLH1 1.06582746870824 4.32067540052176 4.49985036062322 8.35783817299809e-06 7.67940327191428e-05 3.05532925558093

SLC38A4 -1.63541953804119 -0.26148682065333 -4.49472388247422 8.55415484287641e-06 7.83662830239439e-05 3.25019080797005

MAT1A -2.07950868358736 -1.54752464681376 -4.49370851814152 8.59355873180367e-06 7.85949550063741e-05 3.28567154240705

TTLL2 -1.35893362371712 -3.57765971407849 -4.48782668430317 8.82526378496718e-06 8.03763668650128e-05 3.30237684151482

C1QL4 2.47395054573133 0.943283261579305 4.48741085769412 8.8418692100351e-06 8.04939218297089e-05 3.29235565596812

PRLR -1.06415910758477 2.94532732039202 -4.48705058458359 8.85628039196569e-06 8.05914110752162e-05 2.94251085448777

CDCA2 1.29483059451 3.96057834151349 4.48449701874153 8.95907191084543e-06 8.14246840431618e-05 3.06949777657188

MEOX2 1.63509916971811 0.250745188358575 4.48386186693764 8.98481636929587e-06 8.15905289572228e-05 3.27732708479477

GALR1 1.55406573005099 -0.906921816105201 4.48043103730586 9.12510755258444e-06 8.27185739726006e-05 3.27093462758251

PLIN1 1.03706862992991 0.17642638650596 4.48035990038347 9.12803852998514e-06 8.27185739726006e-05 3.25577168506946

CCDC169 -1.08871168977931 -1.36673506780412 -4.47788979781575 9.23037277802334e-06 8.34721745987677e-05 3.23982200370903

RBP7 1.13890384439317 4.36430931484715 4.4751567111353 9.34488308415067e-06 8.43325332059318e-05 2.94840227693629

C2CD4C 1.05483455326531 0.612918207277506 4.47070518034999 9.53431146848042e-06 8.5899568598681e-05 3.20512538945334

LINC00299 1.01984359622042 -1.2881499457434 4.46841947665248 9.63300010830697e-06 8.6609464418636e-05 3.22143195423766

THSD7B 1.34776690051865 1.42659125248233 4.46661042452283 9.71180159662965e-06 8.71379989092488e-05 3.1683637532502

KIF1A -1.59945580240018 -3.89635039471315 -4.4604361968829 9.98542463818871e-06 8.94701846943443e-05 3.18961241049041

GLYATL2 1.26088015576542 -0.0857336266851759 4.46037501990552 9.98817240810661e-06 8.94701846943443e-05 3.18067616540306

THRSP -1.77683617982157 -2.99429031026476 -4.45741464777432 1.01220066923989e-05 9.04828417550086e-05 3.17131958964989

NGFR 1.20230575156823 4.49854859065618 4.45728518406211 1.01278985902665e-05 9.04983450797744e-05 2.85637503537752

NXPH4 1.5107887028088 3.77338698251554 4.45516444707375 1.02248822701233e-05 9.1218588958166e-05 2.97755749932388

CES3 1.86854794413464 5.09845327378673 4.45190217062113 1.03758082732862e-05 9.23722775806219e-05 2.84835987221253

UBE2Q2L -1.00897549381097 -5.26037307781752 -4.44930324029086 1.04975697186592e-05 9.32655510473693e-05 3.14583028506674

IGLV3.16 -1.57854148309533 -4.19951104962717 -4.44559938316991 1.06734646357723e-05 9.46351546748905e-05 3.12924319582284

SLC2A12 -1.04104790694416 -0.821867360362127 -4.44406572767771 1.0747120599745e-05 9.52494194196451e-05 3.08280490879974

LOC100533848 1.09021254129944 -4.07128917210004 4.44284490503018 1.08060999846543e-05 9.56164139699065e-05 3.11967836743328

ANKRD22 -1.14873454841167 1.19881324445053 -4.44212983122409 1.08407898406388e-05 9.57287920735025e-05 2.95564295507204

PLAC9 1.0076848704255 2.95980441674256 4.44076079928795 1.09075024056637e-05 9.62008123436311e-05 2.95216300986148

ATRNL1 -1.44056769068707 -0.0400074130610513 -4.42895868413819 1.14991466071703e-05 0.000100401894985269 2.97146195767423

GALNTL6 -1.1318064511666 -3.32306670025616 -4.42631334435241 1.16359108622397e-05 0.00010143326861769 3.04946320394703

NFASC 1.03729449328182 4.76879690887236 4.4189782072497 1.20233302208602e-05 0.000104559260454441 2.65426270675318

WNT6 1.27069967961438 -1.18247295259245 4.41403322581723 1.22914431240415e-05 0.000106507899076835 2.99875870520454

IGHE -1.55774648259807 -4.40623249832151 -4.40864035031357 1.25903606199542e-05 0.000108555518542194 2.97871421136334

CST6 -1.20445954473657 -2.76917589414619 -4.40760646277241 1.26484562748693e-05 0.000108949420843823 2.970327184452

EGFL8 1.04243680766064 0.313944380869976 4.40660796872576 1.27048065448632e-05 0.000109306531249817 2.94827243970111

PLAAT5 -1.24359210872123 -1.08703235811586 -4.404533019427 1.28226760815843e-05 0.000109929574741176 2.9239639722906

TAGLN3 -1.47833867053726 -3.64642114521214 -4.40429848668072 1.28360645720574e-05 0.000110001030527549 2.95985028349867

SLC9A2 -1.74867076988386 -2.02873747232629 -4.40359014104557 1.28765823041557e-05 0.000110225307928908 2.93432094412557

CPVL.AS2 1.03567279292756 0.259107591591653 4.40357539282032 1.28774272092256e-05 0.000110225307928908 2.93723761612181

TJP3 -1.10943248490614 0.0311808115571065 -4.39544609485655 1.33513049585476e-05 0.000113655790001058 2.84515877139015

CYP2J2 1.96546256402019 6.09202799059143 4.39516671202242 1.33678838214478e-05 0.000113740259152387 2.50218828064303

EPB41L4B -1.25615262367208 -0.897798716123464 -4.39510281378928 1.33716783741884e-05 0.000113740259152387 2.87732070661057

LINC02365 -1.4708289951518 -3.52738290645828 -4.39439952413456 1.34135108884312e-05 0.000113962486927588 2.91933231071416

LINC01485 -1.55138457118649 -3.4870502280188 -4.3862368169359 1.39082889632581e-05 0.000117432787379069 2.88573108017416

WFIKKN2 -1.22890826718177 -4.81088956820071 -4.37877152897922 1.43760374792596e-05 0.000120959879323944 2.85854980819879

ADRA1A 1.23722780040141 -0.740644486120062 4.37685877608761 1.44982833437548e-05 0.000121753137941169 2.8455260001589

CAPN11 1.01442082616396 0.437897244500962 4.36917077967816 1.49997325982706e-05 0.000125528327361229 2.79122038321934

CAPN13 1.22461271627913 0.914179984194001 4.36624812431866 1.51946788086148e-05 0.000127013277122549 2.77048278370744

XKR3 -1.170767443216 -4.56028418537455 -4.36554766340628 1.52417593623391e-05 0.000127357917865657 2.80502592458632

LYZ -1.08856626567532 6.93221341469226 -4.363572967888 1.5375237928322e-05 0.000128227127963903 2.26722628853568

RBM11 -1.51398468572931 -2.94531766237664 -4.36329869267383 1.53938655834767e-05 0.000128333309902542 2.78965223426483

MIR23A 1.23800937621738 -3.81292247646239 4.36306623317434 1.54096701767224e-05 0.000128415884661836 2.79616493536267

PSG9 -1.44204350474612 -5.64061897564834 -4.362690845469 1.54352250147965e-05 0.000128483060042976 2.79435008556376

DUSP13 -1.06959441724524 -5.47085547229971 -4.35921591715408 1.56737150392242e-05 0.000130267184138523 2.78051483268139

IGLVI.70 -1.43786666368739 -5.01406897571686 -4.35490880316322 1.59742106464949e-05 0.000132309985974982 2.76249558827182

SMKR1 -1.14708568442424 -1.44615987329092 -4.3515563675516 1.62119042272958e-05 0.000134085518955174 2.72164484366895

MANSC4 -1.03002431014371 -4.4971526981146 -4.35153798846311 1.62132166072503e-05 0.000134085518955174 2.74878420856818

HOXB9 -1.18762828506321 1.02728073753406 -4.34822866103528 1.64511855159803e-05 0.000135744107326134 2.57475384360239

LY6G5B 1.05384910367808 0.862180610744886 4.34589779290342 1.66207946272488e-05 0.00013693597148044 2.68536282841389

SLC28A3 -1.03646921088878 -2.35282203718523 -4.34415908059673 1.67484015851141e-05 0.000137726655573547 2.71104982281344

CAPN12 1.29131885879682 4.29348251388131 4.34013660108924 1.70472164233228e-05 0.000139761491482662 2.42187035823559

TSHZ2 1.09277778077033 2.71638895054651 4.33978757801629 1.7073382878193e-05 0.000139923315176818 2.56142040849259

HOXD13 1.25538610728622 -1.99829105491117 4.33686662292615 1.72938782788257e-05 0.000141570458253178 2.68904643498802

TRAV26.1 -1.06896080394796 -2.91497855149059 -4.3356663668692 1.73852690594605e-05 0.000142265094592961 2.68140501164066

AGTR1 1.83714880081403 2.69451041343173 4.33116050523599 1.77324930294487e-05 0.000144625903875032 2.56959862822089

ARHGEF16 1.03899801397053 4.82857723735598 4.32917478749147 1.78876079918711e-05 0.000145609410306305 2.26661017153004

LINC00323 -1.38760989342992 -1.9997121539057 -4.32894268098438 1.79058234564924e-05 0.000145679370767132 2.63689534664379

STPG3.AS1 1.39229867881584 -1.14145826994849 4.32757736423604 1.80133312754192e-05 0.000146359157100429 2.64987545271164

ERVV.2 -1.53899243326987 -4.89949489215239 -4.31361213078944 1.91489853410775e-05 0.000154433480518427 2.59711784633441

SLC6A11 -1.02355600989466 -4.65664212523628 -4.31206268870564 1.92791198708931e-05 0.000155310363519516 2.59121893494255

ASIP -1.0903225687154 -2.39152668297252 -4.31177730846401 1.93031803163579e-05 0.00015536637082774 2.58080003872168

CLEC18B 1.85219910496285 3.68543077422791 4.309853807136 1.94661006292108e-05 0.00015635373151145 2.41620691198978

LHFPL3.AS1 1.4703492588995 -2.82745527323987 4.30429206790573 1.99446004492446e-05 0.000159223846595082 2.56062700719376

CYP19A1 -1.18201016068654 -2.80428949370261 -4.29563941336716 2.07114216895999e-05 0.000164354909193409 2.52001857091891

REG3A -1.13429939416592 -5.67171573937045 -4.29275214041633 2.09735048331856e-05 0.000166071400401583 2.51549515981251

HSD11B2 1.33122987266727 4.86038239415257 4.29094026647959 2.11395857134454e-05 0.000167143248174561 2.12040215895219

MROH5 1.10987436460732 -3.41495374512837 4.284563999437 2.17340906308028e-05 0.000171407953174161 2.48276869962794

GABRE 1.45875616577143 3.96840740651331 4.28138105849305 2.20367981575165e-05 0.000173351609844636 2.23559330027993

ADORA2A.AS1 1.32206542542404 2.77525095945158 4.28077686630182 2.20947113269622e-05 0.000173560296446765 2.33525703486408

CHRNA6 -1.15096317869251 -1.75937711079912 -4.28002080011391 2.21673866092114e-05 0.000173817544784836 2.44271106599003

DGKB 1.07745823743834 -1.06838402659749 4.27519946995931 2.26362213862812e-05 0.000177065067898818 2.44004662167913

SCIN -1.22563959889125 4.60413275385846 -4.27089800913192 2.30624778362582e-05 0.000179478353615242 1.87994651716942

UGT3A2 -1.50113811827934 -2.67487423562852 -4.26839528129226 2.33140011249575e-05 0.000181176673504802 2.40742570957626

HTR6 2.0429983915166 -1.13940639547576 4.26714953485175 2.3440171880556e-05 0.000182027192766344 2.41101764479529

SLCO4A1.AS1 1.42994117807768 -2.04658396185717 4.26123881012869 2.40477493963416e-05 0.000186280199683334 2.38908622596055

ARX -1.98025485290284 -3.73697137468181 -4.26071512967678 2.41022984304821e-05 0.000186636332243438 2.38332154456421

TMEFF1 -1.01030583399749 -5.48318538427752 -4.26001797020091 2.41751010817154e-05 0.000187000506483902 2.3862477965459

AQP4.AS1 1.25759922366049 -1.67227428678559 4.25032195796629 2.52096366050559e-05 0.000194106176152194 2.34494884755483

FENDRR 1.11221932448134 -1.23939437395517 4.24936841369024 2.53136273318777e-05 0.000194769079580411 2.33923135183146

SLC25A27 1.11910541489924 2.16766151965379 4.24176291923528 2.61578320278752e-05 0.000200132699385153 2.20446720341555

FBXL16 1.32633401846502 5.0524191734469 4.24103723445746 2.62397711266842e-05 0.000200618580300153 1.91679962273035

PRSS53 1.14718706886431 0.993551889345383 4.2332083947213 2.71394549941897e-05 0.000206554026873611 2.23177567558412

XKR4 -1.39569236272353 -2.83716549771282 -4.23204316837526 2.72758510158939e-05 0.000207519555771745 2.26740656381902

TSKU.AS1 -1.12647153721925 -3.35509101025293 -4.22911989136307 2.7620919103566e-05 0.00020955892161984 2.26111099236786

GP9 1.09826148349567 -3.57004943182953 4.22886743489849 2.76509139161524e-05 0.00020956735487914 2.26402986000906

IGHV3OR16.17 -1.32472146100836 -4.88677629099845 -4.22404597281677 2.82297561238454e-05 0.000213138085864635 2.24425345330682

GREB1 -1.02430480557582 1.07284260803776 -4.22301581288849 2.83549213647081e-05 0.00021400886731817 2.08233852248434

TMEM61 -1.77069637652548 -3.65524582454457 -4.22273218484391 2.83894751718437e-05 0.000214195392050441 2.23476411519426

TNFRSF17 -1.25100475740061 -2.04365675081058 -4.21701647147276 2.90944240651542e-05 0.000218529443970397 2.19749637134732

CALCA -1.56812825567338 -4.84487022860528 -4.21392210371766 2.94829990802313e-05 0.000221142812191384 2.20433244152053

PRSS55 1.04959630287767 -4.64093427273584 4.21186622418715 2.97438956607174e-05 0.000222562865880659 2.19840929923746

MYH11 1.11980036282421 7.15349946837577 4.2065304626028 3.04313238874386e-05 0.000227237950963251 1.60428828014485

HAPLN2 1.04658011675171 -1.27728030681702 4.20389048636422 3.07770163899873e-05 0.00022926875967175 2.16124306972228

CASQ2 1.31640487880791 2.11012617665325 4.20155354140704 3.10861480703029e-05 0.000231492365735984 2.05774094164838

OR51E2 1.09096859345469 1.12690855580896 4.20142645148063 3.11030441071292e-05 0.000231538974377524 2.09796628663059

MCIDAS -1.15946179537619 -5.26101107711307 -4.19793167415081 3.15711028003008e-05 0.000234542045957047 2.1433473306952

KRT79 -1.01618115330659 -5.63698848413598 -4.19659013565032 3.17525527327521e-05 0.000235809558285163 2.13869057091646

KCNK3 1.5704680326391 5.53766543055773 4.19174856172774 3.24156993123676e-05 0.00023999745814024 1.68728604375878

STEAP4 1.10788430148462 5.13797172782079 4.19061197279452 3.25732784165484e-05 0.000241000187387154 1.67242202855205

ELSPBP1 -1.29350100169191 -5.50308450268324 -4.18806234485789 3.29294231497338e-05 0.000243139333005514 2.10524496592192

TMEM40 -1.00684225847335 -4.54580306027475 -4.18555594927134 3.32831426254259e-05 0.000245460799706278 2.09452324006294

SEC14L4 1.19149163186839 -0.497344167169095 4.18551510647828 3.32889364971256e-05 0.000245460799706278 2.08364280820741

SLC4A10 -1.01000505914298 -2.17482088080503 -4.18541979928297 3.33024603100474e-05 0.000245477363213276 2.08074989946109

LINC01914 -1.33041742039258 -3.33805163456279 -4.18179834426955 3.38202293236156e-05 0.000248788418954762 2.07605174894375

DNER -1.53247821826255 -0.412074095742777 -4.18130026292381 3.38920383137827e-05 0.000249148260039212 1.98690863513067

SLPI -1.87353018971452 1.02947805530369 -4.17925449802168 3.41885047952425e-05 0.00025107327391297 1.8410489140388

TNIP3 -1.09978888466105 -0.532665249741217 -4.17749710916679 3.44451513707534e-05 0.000252787461189207 1.9975361166333

COL21A1 1.47231872303181 3.00183410305154 4.1758457546275 3.46879842242272e-05 0.000254312345102308 1.90254094506008

LRRC52.AS1 -1.03289843565681 -6.09964882745966 -4.17396904868148 3.49659344140376e-05 0.000256177547758451 2.0515249229881

TRAV12.3 -1.11888417031929 -2.38336064991298 -4.16526897133185 3.62824026536139e-05 0.000263956904599336 2.00435644288409

SLC13A3 -1.38711186988455 1.19442776971032 -4.162572141524 3.66999699731408e-05 0.00026637153931489 1.79571547560821

TRIM7 1.19916108838209 1.33838396804484 4.16094682889207 3.69538329926132e-05 0.000267858569155501 1.93281219844927

MEG3 1.34534216198449 2.08386613880044 4.1589983045666 3.72603833513629e-05 0.000269719575792856 1.89432050239723

LINC01806 -1.30024765642081 -1.3687793504089 -4.15769463641921 3.74668322328954e-05 0.000271033744504298 1.94550691986254

LMNTD2.AS1 1.58082732795525 2.08481067493649 4.15749614076527 3.74983613705758e-05 0.000271081644620832 1.90161361846534

MYO3A 1.48939955034318 2.93144588365674 4.15499344140663 3.78980644621507e-05 0.000273516965897756 1.83456010233064

DRD1 1.25066328400694 -1.7485998089885 4.15460857670473 3.79598893956377e-05 0.000273872360780526 1.97292701134523

LOC442497 1.06229443337041 -0.578745484508738 4.15231292270273 3.8330663887244e-05 0.00027618125151726 1.95463242906168

SLC4A3 -1.27560864365523 0.543796467200247 -4.14897271884935 3.88763075384451e-05 0.000279465186984589 1.80424039329901

LINC01179 1.58693360872418 -3.96320294313881 4.14817932531299 3.90069957958134e-05 0.000280127112335028 1.9523072748194

LINC01163 1.06571907047662 -4.30081988286874 4.14248484222301 3.99573402284032e-05 0.000286008360654933 1.93047772029067

IGSF11 -1.1996468566769 -1.5065902864483 -4.13869955305785 4.06012100540571e-05 0.00028985455534492 1.87951207060053

HDC 1.07504529347303 -0.30489051931006 4.13697956466339 4.08970278300204e-05 0.000291583886267951 1.89119867274626

TMEM151A -1.39000897968162 -3.67720307820862 -4.13240060268575 4.16945859731583e-05 0.000296493320116869 1.88699202181959

BFSP2 -1.15483331487162 -3.90814824701583 -4.12978304773906 4.21571337290332e-05 0.000299098542985614 1.87830253696091

SMOC1 -1.47066485366603 -0.374141669721837 -4.12887507561229 4.2318718740727e-05 0.000300073676603651 1.77431572390116

IL13RA2 1.28899196951953 -0.106364488960587 4.12735804862599 4.259000815021e-05 0.000301579930841126 1.85342816254112

MUC4 1.3415173658278 1.35174154536841 4.12153594986868 4.36466108903271e-05 0.000307704885103377 1.78400861010177

VSIG2 1.14415316698354 0.404364117638561 4.10968356612038 4.58751449434965e-05 0.000321081762053083 1.76738751532644

ZNF366 1.08605579038362 2.53544904050401 4.10198107329472 4.7380962756137e-05 0.000330029253220107 1.62415682159683

CKMT1B -1.78784249115114 -3.70093721403819 -4.101307163776 4.75149229174341e-05 0.000330856470615415 1.76658727834892

MAPK15 1.46302927935412 2.69216443957006 4.0999212496628 4.77915484643045e-05 0.000332463609914515 1.6344931638127

PIGR -1.93505420968648 4.59911727329397 -4.09812352037309 4.81526532631113e-05 0.000334654790414477 1.1753896682818

IRX6 -1.7857088656942 -0.444873276419073 -4.09297967241068 4.92002682593376e-05 0.00034073886070665 1.63226723150834

CLVS2 1.72386528661925 -2.19146522608038 4.09135520129867 4.95355911406738e-05 0.000342820709173863 1.73346131189835

TRBV4.1 -1.25382059453661 -2.50532845735692 -4.08815050914275 5.02034835221624e-05 0.00034691403994505 1.7086320538589

CCDC85A 1.10816081367855 0.966814536741232 4.08375144490951 5.11342468312447e-05 0.000352339079067966 1.64756674829126

MIR3671 1.00570869322578 -3.55827306367155 4.08167296447725 5.15796995400186e-05 0.000354622653154638 1.69831735532594

HSD3BP2 1.043896027285 -4.25974213142235 4.08122632901054 5.16759011105955e-05 0.000355171878583622 1.69742316958378

LINC00173 1.31911750246005 1.00558886958165 4.07948562462083 5.20524642121992e-05 0.00035742144747853 1.63880150587902

LINC02593 1.35241274007214 1.34721008296442 4.07831264562263 5.23076815118827e-05 0.000358608284557213 1.62060672898704

MGAT3.AS1 1.0273412700763 -3.31245675542847 4.07420636917538 5.32105271275113e-05 0.000363881100843399 1.66976697681493

PART1 -1.68232251040985 -4.10031646257511 -4.07339289734254 5.33911334945745e-05 0.000364657923682586 1.66311979265013

CCDC187 -1.15433465994398 -5.33804028559651 -4.07225315991521 5.36451570825316e-05 0.000365589898001085 1.66268370482422

RIMKLA 1.05433310855269 4.54576422454645 4.07185437195237 5.37343094475165e-05 0.000366082852502063 1.2776018713294

KLRG2 -1.26501766845318 -3.80808589547089 -4.07118835858513 5.38835162369277e-05 0.000366984511241929 1.65509747324926

CRACD -1.04372994980871 -0.387253171877485 -4.05971813437744 5.65157855151174e-05 0.00038287553791085 1.53666840850925

NALF2 -1.20688166110995 -0.319453013835591 -4.05763978513827 5.70056276362401e-05 0.00038535450209877 1.5134919807257

OR2A4 1.5184694548462 -1.00114446761624 4.05480064949486 5.76812985160242e-05 0.000389438221091284 1.59060611181773

LINC02473 1.39659530772098 -2.83237341912943 4.05193302498454 5.83714662390238e-05 0.000393609574233219 1.58561212581435

BPIFA2 -1.22603014512216 -5.11142902145709 -4.04783118114181 5.93723257604155e-05 0.000399368793209816 1.57035120136006

LINC00402 -1.00511875220821 -4.07482790050336 -4.04589373566189 5.9850713624476e-05 0.000401841605016647 1.56127827926599

HAO1 -1.05407778718639 -5.7431554067574 -4.04170790140923 6.08967926407119e-05 0.000407357248128573 1.54856309238467

LINC01886 1.84905977073564 0.0676212787778702 4.03661999821032 6.21916874988766e-05 0.000414998915324355 1.51393974969544

TIFAB -1.00661061410225 -1.16120082723767 -4.03614875486125 6.23129348716691e-05 0.000415425927519639 1.48078694077861

FOS 1.02198098191499 7.89559922007786 4.03494776450365 6.26229562347849e-05 0.000416822951897821 0.924063791837933

NR4A1AS 1.18761640030567 -0.690992835759685 4.0339322306962 6.28862462219149e-05 0.000418120459827228 1.50704088685909

GFPT2 -1.07695243810471 2.54006444885874 -4.03160478103376 6.34936366245287e-05 0.000421603628464051 1.14893686989708

KCTD16 1.51440823421715 1.75089483173917 4.02953516705015 6.40384139909937e-05 0.000424200899982337 1.42112791580501

PSORS1C2 1.02949322853427 -1.99517664623865 4.02587699280738 6.50122112591446e-05 0.00042947520560783 1.48516816494596

NLGN4X 1.06226064546259 1.34866509262824 4.02529249414686 6.51690997265675e-05 0.000430381005384768 1.40366107705248

KLF1 -1.0513315535277 -4.31633336028921 -4.02432780952635 6.54288222054393e-05 0.000431572476650242 1.48109087442466

LINC01714 1.07602277129531 -2.63403623050309 4.02204546965537 6.60472147371099e-05 0.000434992351946345 1.47281314606807

DAAM2.AS1 1.2266684965887 1.02884877465129 4.02128912259585 6.6253365291045e-05 0.000435822605708727 1.41229320183893

ZNF711 1.26429709455809 3.05404417112176 4.0203330899751 6.65148161393477e-05 0.00043730661813158 1.27925279822936

FAM153CP 1.94165002123459 0.593396266531354 4.02031729368576 6.65191442382351e-05 0.00043730661813158 1.44346136025959

RNU6.485P 1.01860351456819 -2.25000396086576 4.0159646755432 6.772197570913e-05 0.000444408877075862 1.44898928194433

ADH4 -1.34145081148141 -4.69746411743065 -4.01561857671152 6.78184996066377e-05 0.000444908161825703 1.44890529640711

ADORA1 -1.03471172190033 1.69867465684418 -4.01424909403217 6.82017179621294e-05 0.000447018005083309 1.200410085403

LINC02154 -1.37206317354995 -3.508929971697 -4.00812065518127 6.9941931996034e-05 0.000456089285128122 1.41707595110477

HOXD11 1.04736873568219 0.600026665074953 3.99952013936737 7.24552755782621e-05 0.000470459735852874 1.34140220267885

TCF23 1.16022457114329 -3.15687004298072 3.99837311301318 7.27968769228858e-05 0.000472019547208953 1.38605034143573

WNT11 -1.02724242356468 -1.33525318753967 -3.99396955853857 7.41225630297457e-05 0.000477910494510804 1.32602601095056

HAPLN1 1.59565154474605 3.29795989978251 3.99077031344426 7.51000180398706e-05 0.000483926019145608 1.16951870443019

DIO3OS 1.41925655502347 -1.09406826342123 3.98525890817889 7.68126287044574e-05 0.000493792229477237 1.33108468245379

GPR20 1.10323845873953 -0.676191896544984 3.98421695769045 7.71405385790121e-05 0.000495607468491546 1.3201355517577

ZNF385B -1.26462202616803 1.91569941617987 -3.98269763650319 7.76210602893603e-05 0.000498400477675075 1.01486103244498

APOH -1.62467440998624 -1.58170199403277 -3.97890375147231 7.88333891559453e-05 0.000504695994634547 1.2604846556745

SERPINA4 -1.65718183320707 -2.61901293116204 -3.97584227754178 7.98247426505815e-05 0.000510442177812928 1.28056153210232

CHIA -1.14544777293254 -4.9405058138992 -3.97430300869676 8.03276357535935e-05 0.000513356326320749 1.29673890918391

SCN1A 1.33938309097285 -1.93790696535371 3.96734338928794 8.26391288493814e-05 0.000525172206374093 1.26857389175419

ONECUT2 -1.33166020622917 -1.80472277956916 -3.96508423534154 8.3402915985919e-05 0.00052866373682746 1.22620523407255

LINC02608 -1.12949461996512 -4.95137652422771 -3.96421637305451 8.3698102466947e-05 0.000530380377408453 1.25964623257666

KRTAP5.AS1 -1.05910289244625 -3.15063946127809 -3.96170199902787 8.45589176746035e-05 0.000534590171659336 1.244689098555

CPSF1P1 1.05109199893769 -1.4668125868358 3.9605710246562 8.49488449761583e-05 0.000536743543859517 1.24065042458511

COL26A1 -1.29599163161914 -3.07260061311115 -3.96018228721744 8.50832634524696e-05 0.000537280938662578 1.23651644899082

MYBPH -1.01762012471995 -3.78805030520926 -3.95660201732842 8.63307691641743e-05 0.000543738961920307 1.22888804814038

LOC285097 1.0241375119528 -3.26252743550259 3.9554064451586 8.67511991781702e-05 0.000545913082541553 1.22755707795602

NPR3 1.30521592360464 6.98958968300762 3.95173076353732 8.80559581666384e-05 0.000553004628220778 0.608793802401332

TAL2 1.34990573524352 -0.251740127080114 3.9451026727825 9.04558557865131e-05 0.000566605068461879 1.17220568691874

DPP10 -1.35906067170082 -4.87684253466217 -3.94248116370653 9.14220429764836e-05 0.000571834370537103 1.17927821100242

RAB42 -1.09137213016834 3.82390562956176 -3.93614112814281 9.37993017126882e-05 0.00058435300526047 0.619140568745317

HLA.DPB2 -1.05872022291803 0.612066114802941 -3.93522358865209 9.41481532722983e-05 0.000586358469893596 0.999937528393863

COLEC12 -1.02348011474885 1.7928007377294 -3.93093733255861 9.57941410737414e-05 0.000595757448214894 0.863244546566413

TRBV18 -1.00455574184873 -2.51371645363021 -3.92986945402683 9.62084425923935e-05 0.00059799233863753 1.12149304970793

LINC02303 -1.33039741771724 -5.41301613335779 -3.91748459901428 0.000101138963723781 0.000624005618870616 1.08931660816111

WNT2 -1.47061116813161 -3.37189634213938 -3.91570291890578 0.000101867644206424 0.000627611947761458 1.07497329890015

DOC2A 1.85693363039585 3.87395492432803 3.91431553959853 0.000102438499999331 0.000630771946106203 0.855117035192817

CLEC6A -1.06411900516114 -3.81584775460553 -3.91293820010667 0.000103008218951466 0.000633384153083775 1.06924420903718

EHF -1.37695100096794 -1.39371505106959 -3.91122634942448 0.000103720484520806 0.0006372237613786 1.01137147781375

SSTR5 -1.30488398678897 -4.42375167424635 -3.90623011476417 0.00010582606396942 0.000648512368925213 1.04612284920189

HPSE2 1.20873246836055 -1.69901957781776 3.90094529559408 0.000108097251305676 0.000661499260379717 1.02450334340921

CTAGE9 1.17147119581274 -1.53434369059985 3.90014398821704 0.000108445619211077 0.00066325816054159 1.02065699323034

SOWAHA -1.43872823536694 -0.812413847419164 -3.89595166304111 0.000110285613831942 0.000672621730535132 0.923133266430538

HSD11B1 -1.01487173154586 0.0234714386712114 -3.89467512722173 0.00011085172046898 0.000675695715331361 0.891481417130718

TNNT3 1.41112531659404 -1.69383310284595 3.89287074767828 0.000111656601269263 0.00068003056514495 0.995836606656169

IGLC6 -1.26243134052358 -4.48948717142278 -3.88709414204833 0.000114270717165707 0.000693042825451641 0.977131497833011

NRXN2 1.14322351344726 4.47676073858057 3.8802137530826 0.00011745987249299 0.000709419823683383 0.575069188688507

LINC01929 1.18631872042666 -0.89635966037346 3.87731874557149 0.000118826750577297 0.000716284098536699 0.933106831069558

SOX21.AS1 1.0796227212095 -4.35913996257255 3.87603743218269 0.000119436512361416 0.000719054165107196 0.940751954224134

LOC107985075 1.1503130484658 -0.445075789350498 3.87539117318664 0.000119745180636811 0.000720224743553873 0.91874457784454

IGHJ2 -1.32014504515787 -4.3837218274019 -3.87129012362995 0.000121721586669132 0.000728986658787093 0.919696581643147

STAP1 -1.1338237617231 -1.0290095225295 -3.87021069716969 0.000122246895595152 0.000731632712790671 0.851573623927718

RPL32P20 1.00599198482903 -2.7322656328518 3.85565252121113 0.000129544406670542 0.000768964575947011 0.864425829668764

ADAM7 -1.45101451959445 -4.29526774466063 -3.85538277559395 0.000129683428121125 0.000769579929092835 0.861902641393513

DNAH11 1.98631745556015 4.2164092875528 3.85303807036159 0.000130897795205949 0.000775816704249183 0.598533664035593

STRA8 1.22520992876382 0.697485771557163 3.85120599054197 0.000131854134187787 0.000780546352696644 0.798584026218782

KY -1.23085333496759 -2.20381078947161 -3.85063777301793 0.000132152080534262 0.000781884842889175 0.822694846909912

MBL2 -1.25967823698053 -5.26969513786375 -3.85040001636356 0.000132276937550013 0.000782198342746844 0.847170333501239

MIR140 1.35154759406327 -1.74852942969762 3.84984112640176 0.000132570875638735 0.000783510792839625 0.84093973539086

EPN3 -1.33525764122464 -1.95570759208146 -3.84604152765645 0.000134585627065378 0.000793909307407069 0.794400606913769

TNN 1.20008283946243 -0.154972958178752 3.84460814018764 0.000135353174361162 0.000796925222158348 0.801203435496339

SNCB -1.19381575330196 -4.8154407647623 -3.8442298896114 0.000135556407770487 0.000797690275193345 0.82415792556464

UNC5B.AS1 -1.07709289284343 -3.01590218755128 -3.83730231977641 0.000139329959618346 0.000817465021836261 0.792003774355289

UPB1 1.53733655963408 4.22934744135169 3.836338013541 0.000139863032376977 0.000819929605642247 0.482048388100869

KLK2 -1.06923834614113 -5.49695617105922 -3.83541361762887 0.000140375850569898 0.000822050346880543 0.794431616166305

LTO1P1 1.00942424809961 -0.688473685693757 3.83375996212539 0.000141297666231052 0.00082700357645908 0.771265969860292

IGKV1.13 -1.07364661810027 -5.39197588941248 -3.83162276561008 0.000142497498720548 0.000833353856703432 0.780738021445028

LINC01124 -1.29687814934198 -3.10075406590435 -3.82220485229685 0.000147900312239684 0.000861017945044453 0.736914379578126

C18orf63 1.07148385420127 -3.4973121146246 3.81901829061542 0.000149771744390009 0.00087015260104866 0.735755811632736

RPL7L1P9 1.02489361202252 -3.64346972649207 3.81645400699837 0.000151293930437597 0.000877488671418912 0.726870663453552

PANX2 -1.27072902131601 -1.09633637868545 -3.81544991134648 0.000151893941942845 0.000880030725118424 0.65424540960766

SLC5A11 1.07600750167493 -1.68100990850969 3.81509621326285 0.000152105833710193 0.000880600259581731 0.715810238396891

GABRB3 1.72742442498339 2.86448140962966 3.81346457464051 0.000153086925761551 0.000885528331929758 0.558565168539115

LHFPL3 1.33314820724166 -2.0121725515083 3.80849361641989 0.00015611287721582 0.000899684669938248 0.694722906170704

F10 -1.0828356947721 1.99587740802403 -3.80562317850431 0.00015788580727095 0.000908218913019757 0.359306859255396

PGGHG 1.19546171496582 5.85112879632733 3.80228834787204 0.000159969430300659 0.000918726711471158 0.109533067106209

FSIP2 1.15077722533812 0.391255024486702 3.80135270400838 0.000160558671612224 0.000921402743206771 0.627480068411661

LINC01354 1.34412510938642 -1.5773811372092 3.79973200202366 0.000161584192008131 0.00092655508625948 0.66167331064944

ANXA8 -1.31178496437502 -4.8055080141468 -3.79072145901161 0.000167399362776992 0.000954869478398002 0.633903845970837

SLC28A1 1.23441076944854 6.63670206300959 3.77936175208426 0.000175012164313275 0.000992830279021908 -0.0240722383642762

ZPLD1 -1.47285183995017 -3.10600410324023 -3.7720472478962 0.000180085592171683 0.00101736908507683 0.557448606611398

CLEC18C 1.99170120557595 1.89770207914579 3.76717313948897 0.000183542981139211 0.00103421694808626 0.4712286569533

NOTUM -1.07379404404081 -4.14406657966348 -3.7644576686182 0.000185496213254657 0.00104495240008129 0.540125603710691

FMO2 1.53690865951585 4.60210092564 3.75751383718107 0.000190580389543468 0.00107026917935827 0.147739488988014

KANK4 1.029597914452 -0.00224329096715987 3.75368979638747 0.000193436091101111 0.0010843480285856 0.468790688149519

LOC105372316 1.09050560202765 -0.25946054861897 3.74702664992319 0.000198508545102024 0.00110821633783938 0.455526267534222

NDUFB4P11 -1.11345687164371 -4.44300264133744 -3.74665189343698 0.000198797526463653 0.0011092606404856 0.478753203394673

ASB17 1.18361993913813 -4.12200725506007 3.74628167397033 0.000199083398416141 0.00111047198435197 0.481048114710822

MGAM 1.69679645395161 4.94297935854422 3.74623864187075 0.000199116651579259 0.00111047198435197 0.0729644667643203

RAMACL -1.13094055589442 -4.66988047824715 -3.74360637582354 0.000201160721680045 0.00111986328102546 0.468777357756917

C19orf81 -1.25038577674172 -3.7035366383831 -3.74299108750515 0.00020164136242712 0.00112196511655192 0.462672146806578

LOXHD1 1.06672070998283 -0.532327645654328 3.74125109676523 0.000203006442185768 0.00112869507715393 0.441117527723872

C9orf50 1.06565394828914 -1.31034568518534 3.73517181216919 0.000207844495539928 0.00115227344806063 0.432122587465004

MMP8 -1.10625937021304 -4.839145707077 -3.72787986523423 0.000213790871312772 0.00118111317153937 0.414640023591779

SPIC -1.15838653305829 -3.73616122437111 -3.72769999673552 0.000213939553962847 0.00118159802543879 0.409986622916808

TTR -1.69590960351378 -3.77022265996087 -3.72730882823989 0.000214263237332385 0.00118282218793153 0.405917827477487

GRIN1 -1.01770192273033 -4.48416660409974 -3.72601365953245 0.000215338255407542 0.00118748351248406 0.4073226258436

KISS1R 2.18030463287852 1.63471568890596 3.72238380361921 0.00021837824119415 0.00120096997879562 0.334883962513755

NCAM2 -1.15551773019187 -2.27930445630039 -3.71851565465206 0.000221662244582644 0.00121626470325949 0.358431237262182

RHBG -1.70226033754632 -3.57053905160918 -3.71812229855452 0.000221998790171327 0.00121780435122462 0.3719680206114

IGHV3.71 -1.18682334816798 -4.52566599822913 -3.7150682629955 0.000224628159761364 0.00123005815183034 0.36908308943485

SPTBN5 1.01144719695501 1.65784840863396 3.70617755937031 0.000232450479699689 0.00126652054859152 0.21748322663908

RAP2CP1 1.01079632069873 -3.9301641498736 3.70543834022737 0.000233112282246403 0.00126980857048485 0.33899034092616

MTCO1P53 -1.0967765111437 -2.17150630618854 -3.69889057111467 0.000239052225363178 0.00129567972845625 0.287626896525166

CKMT1A -1.61522163565308 -3.73668366926162 -3.69874380242316 0.000239186987288376 0.00129608741655616 0.307377554958395

CCBE1 -1.29088824659146 -1.0757096456755 -3.69847193600704 0.000239436801866223 0.00129647285229405 0.233808347075612

PPP2R2C -1.28833129043435 -0.756606137924839 -3.68839285714393 0.000248873498294458 0.00133924089922756 0.189854029432895

ZBED2 -1.16995680411246 -0.9411171196774 -3.68723000862073 0.000249984465765923 0.00134390831362983 0.19969146071567

ASPA 1.05013289001428 5.35205702410908 3.68678212474169 0.000250413610048241 0.0013452006539783 -0.265965574444543

CYSLTR2 -1.1576574482259 2.45086955544568 -3.68447766071739 0.000252632630435132 0.00135511445704325 -0.123778196398535

AQP9 -1.10860399792892 2.00327931526979 -3.68374521734455 0.000253341780849994 0.00135858352888934 -0.0658778309896952

DPF3 1.0216117873746 2.2602412516164 3.68250817601307 0.000254543734739712 0.00136368532465649 0.0947910344050982

CCL11 -1.41747879896338 -3.16879466294737 -3.67597424738665 0.00026098180633747 0.00139317746197834 0.224741437707709

CR2 -1.32335778861812 -2.87269966646984 -3.67594721187279 0.000261008760506623 0.00139317746197834 0.221298438779118

FARS2.AS1 1.35260578179231 0.808542124721977 3.67523931475667 0.000261715459919652 0.00139660711352564 0.176052202875992

LINC01428 1.4772408774586 0.205779147352264 3.6738993526165 0.000263058083251906 0.00140239659518595 0.197453495020339

IGLV4.69 -1.33892595377069 0.315435467860625 -3.66954613089361 0.0002674647796378 0.00142344886512861 0.0476879676748121

SOHLH2 -1.16474162492732 -3.03276115778583 -3.66686380603298 0.000270214508029288 0.00143597636627771 0.194948666772459

EDN2 1.60365573157858 2.22378994512158 3.66553082217787 0.000271590850946283 0.00144223421628391 0.075987963377897

TMEM252.DT 1.30993805789293 -0.554586251478657 3.6556535815025 0.000281996581732901 0.00148841406270127 0.150491808060234

FGF11 1.24372327727875 0.0322404051839608 3.65537688289051 0.000282293410188539 0.0014896195538361 0.131440957813421

PEG10 1.25011712372978 4.71250129467205 3.65516749068802 0.000282518230809504 0.00149044457829144 -0.268022900497916

LOC400541 1.32723741328906 0.872254776789514 3.65052852514835 0.000287542365405968 0.00151255066887185 0.0859643752402883

SLC9A3 1.61594955599873 2.14905396162509 3.64041543854216 0.000298787550520072 0.00156076520570444 -0.00754211680749872

LINC02188 1.31711477705299 3.36373776418119 3.63744823952299 0.000302164480558396 0.00157651348233811 -0.142537174244203

TRBV27 -1.09252216900402 -3.24166721187296 -3.63325243384719 0.000307000889713937 0.0015986814273692 0.0831986080723111

BICDL2 1.23320021666063 2.13762762963613 3.62981357433803 0.000311018888036015 0.00161689709478862 -0.0647080459984055

LINC02048 1.22515633442753 0.28499000298856 3.62966046194366 0.000311198929172669 0.00161706065679195 0.0350595441584476

CCL7 -1.10680264895893 -4.50365402087032 -3.62703722604864 0.00031429876872717 0.00162927870895077 0.068033102711758

ITIH1 -1.41531144743642 -2.75584994455589 -3.62695795678237 0.000314392889751021 0.00162937857885964 0.0492600572808737

HPN.AS1 1.06243621849887 -1.3340997202705 3.62225567556222 0.000320023764245959 0.0016534434551013 0.0444408699650616

SLC52A1 -1.0326127887401 -1.72665964903049 -3.6212786392492 0.000321205559893626 0.00165797520090219 0.0103287743052567

IGKV1.33 -1.19341967473642 -5.0912425655974 -3.61908502040538 0.000323873839310386 0.00167016390909007 0.0427586294055926

IGKV1D.43 -1.24033150079234 -4.52336569235641 -3.61504408088974 0.000328843710796043 0.00169178469697411 0.02716933007357

SLC38A5 -1.09620773491518 1.67921388741784 -3.61205471893919 0.00033256622793507 0.00170696901116707 -0.276269281477539

CES4A 1.29419096851191 4.10280915322056 3.60871083710232 0.000336777052534537 0.00172323133580613 -0.342208935942578

LINC00488 -1.3946110388181 -3.87401689156647 -3.60562079166087 0.000340712656434455 0.0017396885743863 -0.00786460565921665

SAA2.SAA4 -2.03368479362775 -3.60219244073086 -3.60369170151311 0.000343191455511878 0.00174947269604849 -0.0228257057898711

LINC00706 -1.09470456316963 -5.15670859930033 -3.59891158561323 0.000349406796037007 0.0017765797078574 -0.0247265216134611

ANO3 1.12958378592462 1.52427147962472 3.59632668826681 0.000352811636255606 0.00179012841174261 -0.146722584176543

LINC00588 -1.03143929504381 -6.16817382561633 -3.59277512344441 0.000357540549274667 0.00181116712498526 -0.043276855591043

MYOCD 1.05657345227002 1.29969974742618 3.58862014334498 0.000363148345284787 0.00183592691936724 -0.162542000083008

HOXB.AS4 -1.36799335101197 -3.09118292738786 -3.58797109032736 0.000364031756253245 0.00183848706226552 -0.0743477537091302

ST8SIA2 -1.27255476158287 -4.37199169225689 -3.58337330394808 0.000370347630086193 0.00186562297248002 -0.0798632481639281

APOA1 -1.15017294770051 -3.06639050161613 -3.58310724322349 0.000370716237923177 0.00186704774430213 -0.0882725756296425

GPR15 -1.19049572424457 -2.9262665906663 -3.58086107089521 0.000373841884272864 0.00187840148132103 -0.0981822930441192

VSTM2L -1.25467046516286 -1.82592103760042 -3.5789941703209 0.000376458539674141 0.00188899337385998 -0.137005982410657

CLEC18A 1.5682491834415 2.5935703238974 3.57759597931937 0.000378429482989238 0.00189624184075201 -0.258739272845564

IL1RL1 1.42204730788213 1.7860226334852 3.57477249482773 0.000382439097372945 0.00191325024879726 -0.2200044215042

CHRDL1 -1.70457905309924 -0.346637452907538 -3.56761797102102 0.000392778192769229 0.00195779228479129 -0.280963675479754

POPDC3 -1.07291453219432 -4.33318343538491 -3.56757135612424 0.000392846407361523 0.00195779228479129 -0.132090934894379

TRPA1 1.19880530149988 2.02699011581427 3.56411173865995 0.000397940187900159 0.00197942506170539 -0.284661050556346

GPRACR -1.02969204582125 -4.28833585681956 -3.560412081637 0.000403455824645463 0.00200319866219384 -0.155930831808353

CHL1.AS2 -1.03653798896937 -5.66361082298787 -3.55840534926965 0.000406477441535985 0.00201464510195175 -0.158603219225605

TERLR1 1.2840276178832 -2.82683358836926 3.55617809246319 0.00040985592598541 0.00202942082368617 -0.168335322179956

CLNK -1.05619556830712 -0.686252775710594 -3.54856617490064 0.000421601904317233 0.00207577666846261 -0.290569082036449

HCAR2 -1.03845770898397 -1.53787771139463 -3.54811670932842 0.000422305239577688 0.00207876936903834 -0.245304060699965

LEP -1.18008762959497 -3.80435481888575 -3.54646575948999 0.00042489812946218 0.00208775566230322 -0.204867280044787

CPN2 -1.80886167288084 -1.83610859628168 -3.54537453679328 0.000426620117863983 0.00209515718097029 -0.269427348702516

SCEL -1.30413295916933 -3.94535683659488 -3.54104190074969 0.000433521858303291 0.00212191860553157 -0.222662349257518

GABRQ 1.00594755163845 0.675039061215319 3.54094599721599 0.000433675805320071 0.00212191860553157 -0.293029943382868

AQP6 -1.51198048558152 -2.6067142976645 -3.53197963031747 0.000448297091182863 0.00217862977981187 -0.277296025040027

IGLV10.54 -1.54867454359307 -1.49419499075857 -3.53099456111206 0.000449931254474739 0.00218608339646242 -0.32322602094059

NPIPA5 1.04663775952633 -0.553850054034261 3.53009199058139 0.000451433450781472 0.00219289264074097 -0.277346870544257

C10orf90 -1.03022887949438 -4.43873583230151 -3.52846229218581 0.000454157740552664 0.00220514198942892 -0.261122491612024

ARHGAP40 -1.51555930516314 -3.54905502449978 -3.52762858231164 0.000455557355190239 0.00221045852662192 -0.27093127694145

BHMT 1.20327629958025 7.17540350135391 3.52686221630072 0.000456847473884553 0.00221425049299601 -0.935845232546784

PCOLCE2 1.3958688690839 4.29530181978427 3.52294053432909 0.000463503007697077 0.00224002441575095 -0.650375637657253

PCP4 -1.66723484365893 -2.87511017848841 -3.52093067989159 0.0004669490130466 0.00225167903566357 -0.30852493756051

GRIK5 -1.01371243864385 -0.591807180595296 -3.5165116645465 0.000474610119366876 0.00228255379325205 -0.400661220002287

IGKV1D.13 -1.38259377734946 -3.57215074843007 -3.51517818403727 0.000476944926108552 0.00229226323837599 -0.310687423701141

MALRD1 -1.03776766437289 -2.47057786968264 -3.51122459472164 0.00048393061656618 0.00231918047793836 -0.336527648947323

IGKV3D.11 -1.3550388640011 -2.68852966477037 -3.50966776386274 0.000486707587121738 0.00232941162024601 -0.342203695107726

GSTA1 1.63951958412745 6.48283172019556 3.50459095912781 0.000495867138016852 0.0023685626493774 -0.965230512693952

HCAR3 -1.08207063790216 -3.6454750239416 -3.50415442797072 0.000496662205487504 0.00236998118889055 -0.344699250833499

AKR1D1 -1.08388265603921 -4.38060072396745 -3.50053264615153 0.000503304787859187 0.00239830020081675 -0.353216543717495

UCN3 1.16308328578255 -2.6413034805045 3.4983090617312 0.000507424040890117 0.00241581345101819 -0.359931411521455

RIMS2 -1.36395276963797 -2.47573417391536 -3.49703593886516 0.000509796704133388 0.00242394461160189 -0.391005587221741

KCNK15 -1.28151851643288 -1.80127414053345 -3.49122275822778 0.000520762858400771 0.00247121106143658 -0.432241238067534

GPRC6A -1.16398612788344 -5.90596200043926 -3.48140426005867 0.000539786839885734 0.00254933812774818 -0.411324151781246

GCKR -1.24706235278503 -3.37576713760154 -3.47692356332834 0.000548682535199978 0.00258678205408229 -0.436474941470434

MYOC 1.20410293136282 0.0497126871997749 3.47566650613544 0.000551202708998906 0.0025952907996494 -0.470857396275215

SCGN 2.36407908845537 3.35401042290734 3.47514821488289 0.00055224493468342 0.0025984585323586 -0.608103231224598

LINC01751 1.02572017863457 -2.59490870614411 3.4707249085149 0.000561214899799041 0.00263331854363564 -0.450362710208122

LINC01671 1.03933052378342 4.33178553203977 3.46953181694795 0.000563657553183094 0.00264307065061103 -0.877939124921799

TENM2 1.18586013655619 0.5052084683562 3.46922038789792 0.000564296783714645 0.00264549818891163 -0.512225388046752

GJB3 -1.21283042689381 -3.01383807918837 -3.46818636694856 0.000566424045187756 0.00265221196603322 -0.468644371497942

LOC285626 -1.08660918713566 -4.6893803204748 -3.46450184904004 0.000574065138461996 0.00268262685034398 -0.469324404828478

KRT23 -1.15306898450248 -3.06723027778266 -3.46220383869566 0.000578879462394369 0.00270222373106117 -0.486694558892461

G6PC2 1.12077854321145 -3.58718337769177 3.46204876309202 0.000579205699287715 0.00270316690746048 -0.474903188843746

RIPOR3 1.22888947615633 3.77367297927949 3.45509649319412 0.000594008987624956 0.00275982788337938 -0.833447851726349

PDK4 1.03174432189686 8.4793691234697 3.44645081844429 0.000612910709569839 0.00283253235991649 -1.19823685590144

CTNNA2 -1.23019550182656 -4.47475780161789 -3.44483967723343 0.000616494549400339 0.00284714236648159 -0.53413625403738

A4GNT 1.02026180494066 -0.94436382911939 3.44479452654206 0.000616595264188644 0.00284714236648159 -0.547920107600007

TENM3 -1.06154887375659 1.25188564349667 -3.44186265320697 0.000623168173406756 0.00287383509121713 -0.801961525893223

PLA2G4F -1.67643069137575 -3.45727573564981 -3.43238751461867 0.000644860049396463 0.0029619473929548 -0.5846034381729

IGLV7.46 -1.3263029390027 -0.495413486518126 -3.42996100471676 0.00065052735846072 0.00298420000244773 -0.703327583833398

EPO 2.29105615150556 -0.410231320229572 3.42931724929315 0.000652038678589313 0.00298987274422869 -0.592967892520893

ANKRD30B -1.07416409824523 -4.98372428393036 -3.4274986289417 0.000656325894660907 0.00300383636439949 -0.587440383831806

LINC01114 1.17865007805018 -0.92092567102277 3.42167695524472 0.000670227322308161 0.00305717479561646 -0.620621968605565

GALNT9 1.60963839270795 2.2370041449138 3.42094124146057 0.000672003523420251 0.00306344708954455 -0.753148733141494

MTAPP2 1.00614893338577 -3.40493054754424 3.41828454857128 0.000678454061105089 0.00308629248653594 -0.616353894832213

LINC00887 1.40708074983748 4.66515979641043 3.41403559361974 0.000688890763549769 0.00312212376314616 -1.05738396694265

CYP1D1P 1.11953486456625 -4.29356672022732 3.41396879560789 0.00068905602784739 0.00312212376314616 -0.628019575112319

SSTR5.AS1 -1.13029051315836 -4.41542292315736 -3.40888704183848 0.000701737643542558 0.0031696873390726 -0.649220966953214

NEFM -1.3716566101299 -1.33950638208455 -3.40809898747797 0.000703723619021569 0.00317668021883917 -0.726158044664905

IGLV5.37 -1.33953183867255 -4.36363979949758 -3.40745652194339 0.000705346570401658 0.00318025919522585 -0.654796854078845

SOX11 1.16179362201093 1.48078004410814 3.39985099173005 0.000724826015426716 0.00325103809556837 -0.794860784041482

IGLV3.12 -1.06157354440892 -4.03191505696716 -3.39740221436832 0.000731203773512585 0.00327511127208713 -0.687210754088274

ACAN 1.05528227601122 4.76181691719305 3.39421490137367 0.000739583335334328 0.00330564896513805 -1.19562374487484

TREML4 -1.03178332725141 -4.52285465986852 -3.39334308920778 0.000741890890649375 0.00331528208104392 -0.697906510737988

CAMK2B -1.00182712260458 -0.319634351424097 -3.39279587651938 0.00074334270364932 0.00331904423186354 -0.822078727251768

CRTAC1 -1.24298316789898 0.99988778702596 -3.39242453182793 0.000744329426913304 0.00332276838302336 -0.96012979985378

TRBV13 -1.00143077022684 -3.71082259221429 -3.38958514139004 0.000751914570160618 0.00335250398375383 -0.713393348051628

SPAG6 -1.04390034762351 -3.26642726265814 -3.38679465732309 0.000759439145828523 0.00338120513136622 -0.725431438805973

RGS7BP 1.10479669533484 1.31640585801694 3.38372475831202 0.000767798132945097 0.00341074733873794 -0.838687399155291

CLDN2 1.23651224265158 7.147406504022 3.3818276024093 0.000773006616202327 0.00342803369767206 -1.42198071022438

CST2 -1.13966966944661 -3.70990633290425 -3.37894459025616 0.000780984693929965 0.00345873730066603 -0.747819214564857

IGKV1D.16 -1.2935752936291 -2.87386439011916 -3.37792235578363 0.000783831853601523 0.00346993450423924 -0.762548607010842

LINC00189 -1.01338019592881 -3.00291767957615 -3.37782554562103 0.000784101992557543 0.00347042457747053 -0.756228387485392

HOXB13 -1.62999482262861 -3.63285905635215 -3.37696668826217 0.000786502344617531 0.00347821953175331 -0.758049643713634

TROAP.AS1 1.04505076317138 -3.72264998584328 3.37637298337845 0.00078816563947127 0.00348415951957171 -0.74850384195449

IGKV1.39 -1.33563438345088 -4.34686001657581 -3.37480092118183 0.000792585678554537 0.00349943457709871 -0.75848390823497

LINC01014 -1.04261333771872 -5.12808952962607 -3.37157162114757 0.000801737701939002 0.00353198113728781 -0.76466719125659

IGHA2 -1.13053959636463 3.98100378316478 -3.37099265762162 0.000803388878644699 0.00353851997601359 -1.39833965025215

UCA1 -1.12655021135546 -4.74627502182489 -3.37084666282806 0.000803805748244719 0.00353963983856824 -0.768538711102152

IGKV3D.15 -1.3834429848308 -3.16230176537 -3.36520086828818 0.000820081858322708 0.0036032946730138 -0.798708488721987

SLC36A2 -1.37258861556889 -2.21793691926566 -3.36121595446897 0.000831753884587144 0.00364574845062593 -0.83667743694399

TM4SF4 -1.13682399132935 -1.75844800899222 -3.3611144453582 0.00083205321880261 0.00364632623589217 -0.848054797070061

IGKV1.9 -1.25343348836427 0.665922390128353 -3.36094106775708 0.000832564713524028 0.00364769751939427 -1.0142350422455

IGF2BP3 -1.25968795188138 -0.954665969309698 -3.35932604152866 0.000837343400184251 0.00366581934670366 -0.896667994354165

CA4 1.46433467031413 1.80817226677539 3.35625411259148 0.000846503396026165 0.00369808519133562 -0.942490899827912

PSCA -1.05553110812668 -3.29355506868428 -3.35622776135241 0.000846582372785657 0.00369808519133562 -0.821923047614715

TCN1 -1.31031515989862 -3.62303488111223 -3.35564374912177 0.000848334463875344 0.00370499523980229 -0.822847446139553

EYA4 1.04589665612967 0.201696079092992 3.35463790111905 0.000851360010044103 0.00371522721303729 -0.873788468890024

GCGR -1.49558016050306 -4.15956942183722 -3.35210788584042 0.000859014607312536 0.00374337756904344 -0.83170651568156

KCNJ3 1.24626442675829 4.07451067855123 3.35040388918044 0.000864206089412368 0.00376148249314917 -1.20432061909555

F2 -1.64765330311123 -1.23513331186437 -3.3472677139822 0.000873837256385101 0.00379354119659643 -0.940121269706197

MAPK4 -1.36491669074223 -3.11149089479989 -3.34311138724621 0.000886755105321807 0.00384119370697309 -0.869204268037389

PTGER3 1.39894721640947 4.97482327945506 3.34273512015727 0.000887933262843803 0.00384476692506884 -1.37029032853588

NLRP6 1.06451566788234 1.57207368618023 3.3408079897102 0.000893990287383038 0.0038679856920222 -0.991203280854513

SCN9A 1.12541630966929 3.24536642948006 3.33898440961962 0.000899757195805247 0.00389054725488534 -1.1564586948764

IGLV6.57 -1.19353000906642 1.00076669905564 -3.33336410699197 0.000917749128600167 0.00395733860583198 -1.13529418728897

NRAP -1.40459911594789 -2.4002746492697 -3.33179320306315 0.00092283742487653 0.00397849123138987 -0.924726650696578

IGKV4.1 -1.141259477055 3.30238700779549 -3.33024734165762 0.000927870186023702 0.00399464998797032 -1.45741907800119

ANKFN1 -1.10666068924552 -4.21284725369777 -3.32813147374803 0.000934800028666823 0.00401812642653845 -0.904648969385452

LGI4 1.43493621253911 4.98902024356429 3.32383390113831 0.000949023603065783 0.0040656163684182 -1.40415716081465

TUBB4A -1.09001791742909 1.70850535048549 -3.32372886656544 0.0009493737371068 0.00406631604399916 -1.24119309966309

MCCD1 -1.36901803073177 -3.6381629834687 -3.32115345482877 0.000957996537750695 0.00409519062003523 -0.931206478766926

LINC01612 -1.07301529996154 -5.74425285672198 -3.31948523586168 0.000963620703907556 0.00411519165429778 -0.92611966410369

LOC93429 1.13043421635518 -2.94943470761581 3.31159530920302 0.000990638377828947 0.00421650871533099 -0.953448775260799

LOC107984827 1.07187948427051 0.424786253186819 3.31032287367411 0.000995060910385624 0.00423036930397732 -1.02201840008715

FIBCD1 1.56647630723616 -0.63131888561649 3.30587673541388 0.00101065843637199 0.00428943977656253 -0.985891991939581

TBC1D3 1.13691474394452 -1.59397201187279 3.30239829683127 0.00102301907385482 0.00433230664992177 -0.989421290453024

TBC1D3L 1.13691474394452 -1.59397201187279 3.30239829683127 0.00102301907385482 0.00433230664992177 -0.989421290453024

IGHV1.12 -1.06825020748288 -4.54625121077671 -3.29962537023361 0.00103297302407245 0.00436851055274628 -0.991502684763034

MZB1 -1.02953119810654 1.24887218706811 -3.29842093827664 0.00103732449908615 0.00438350657573445 -1.25828187713221

BRINP2 -1.05366921814131 -3.92592537447743 -3.29018542053951 0.00106753733017725 0.00448073371885235 -1.02350653147313

SLC6A18 2.34628929860586 -0.111136905741058 3.28720951823762 0.00107865406150125 0.00451694170001881 -1.04354292940905

INSYN2B 1.41149097590303 -0.0248483867734725 3.28292429573774 0.00109485060632763 0.00456846680478909 -1.07661517813079

CREG2 1.03142453797984 0.778601594108215 3.28075307700202 0.00110314282378447 0.00459473973312601 -1.13659091108154

GDA -1.13519314825692 4.59249970295921 -3.2756519885975 0.0011228543449643 0.00466523583257072 -1.74986037408398

CAPN14 1.08665792323596 -1.04585807581618 3.27292371460791 0.00113353043512598 0.00470151619309969 -1.08697580197798

SPINK5 1.17481621522308 0.714172757687841 3.27266540488165 0.00113454609602465 0.00470483232466539 -1.1522891716024

IGHV1.69D -1.30133536675202 0.0562589814006931 -3.27151965237252 0.00113906131568788 0.00471547121692244 -1.24736858832304

ACTBL2 -1.13151476806675 -4.6660934846785 -3.26402210956727 0.00116902146246267 0.00481386495902857 -1.10070798509049

IGHG4 -1.21451754086032 3.00275537210456 -3.26175781149596 0.00117821215824022 0.00484254985808436 -1.64859507218332

ENPP3 1.18582016954711 7.47435279383965 3.26094172745717 0.00118154099253376 0.00485348231448996 -1.81698448872914

ALPL 1.03421650166159 5.68875869985017 3.26084991286491 0.00118191605223165 0.0048541069262125 -1.74795329108731

AMPD1 -1.06052378917255 -3.22711310801836 -3.25783865077561 0.00119427827434435 0.00489471948741357 -1.12765698635636

IGHV4.28 -1.16569809601516 -1.77303783406198 -3.25481043202716 0.0012068308271726 0.00493408839501615 -1.17989580009444

PRPH 1.26439386868916 -1.26860056436713 3.245359527415 0.00124679689851945 0.00507461258228736 -1.1668067819527

LINC02177 1.02051349936687 -2.83700936469738 3.24148328403274 0.00126354044239966 0.00513220466686198 -1.16947752276689

RERGL 1.15326042243755 0.527581043755436 3.23841895212109 0.00127692398888291 0.00517880743552084 -1.24877201170699

CYP4A22 2.15032881246886 0.478173865417405 3.23832147366556 0.00127735187432728 0.00517880743552084 -1.20970722254168

IGDCC4 -1.09206072653387 1.00836690632666 -3.23587819269199 0.00128812020466985 0.00521646744093928 -1.43132520014477

LHFPL3.AS2 1.39956680584951 1.8866643532601 3.22465176068536 0.00133868728484084 0.00538219544313457 -1.36046512173198

RBM46 1.02767113967133 -2.2052823935684 3.22334842135351 0.00134467541307144 0.00540227938654965 -1.22759256103183

CD177 -1.05284706510026 -2.05668204170383 -3.22193829644954 0.00135118206524294 0.00542341763015944 -1.26585678209516

SLC38A3 -1.1490291172 -2.67212685098551 -3.21524835120348 0.00138244980335496 0.00553056145370842 -1.2687113840167

KCNG1 -1.02905892346098 -1.91477928027146 -3.21156011028206 0.00139997289690726 0.00559038892808296 -1.30230695202661

NETO1 1.11867264379494 -1.63425452492187 3.20487080491256 0.00143227931422531 0.00569318614566937 -1.28701547865827

IGHV3OR16.9 -1.02768686334933 -4.70426812264554 -3.20093790702061 0.00145159379729698 0.00575010776810982 -1.29130799868421

PSG4 -1.39441247073392 -4.07386728030186 -3.19973223352376 0.00145756290436436 0.00577060235345561 -1.29970391793443

SLC6A20 -1.48933406434364 -0.387126744152272 -3.19530095631068 0.00147969693356768 0.00584123379633073 -1.45870146163364

LINC00671 1.94740769626491 2.2282641088816 3.18820779417028 0.00151577433250554 0.00595664558506014 -1.46800405319786

IGKV1D.12 -1.0513040270093 -4.81207872293483 -3.185503569675 0.00152974128109606 0.00600069894857055 -1.33717257708314

LINC00494 -1.11487981972133 -0.0396438112420525 -3.18528070251037 0.00153089764536343 0.00600415298137403 -1.49332678570689

C14orf180 1.73597495803256 -1.17462597135966 3.18441222560947 0.00153541151534091 0.006018078328611 -1.3489617551336

FOSB 1.45157088135259 5.59501747348289 3.18438482217999 0.00153555414329515 0.006018078328611 -1.92374860731616

IGLV3.25 -1.13843043804137 1.42198437184292 -3.18049241411295 0.00155593791382799 0.00609028961882644 -1.65969520931956

SPINK2 -1.11529679867566 -3.75848602817614 -3.1790464923544 0.00156357335925752 0.0061135802606356 -1.36198453934344

LYPD6B -1.21853621206572 -2.77663427058143 -3.17052793266122 0.00160926392111666 0.00625626857848658 -1.40231934855955

AHSG -1.06355027976325 -5.00836801209863 -3.15238802276977 0.00171069469141298 0.00660342105834127 -1.43487729861212

NTSR1 1.03368313308682 -1.33614624290947 3.14890594242072 0.00173082713696905 0.00666930683136933 -1.45755020809874

TTC4P1 1.11099660478112 -2.79211262721647 3.14876570332087 0.0017316425200485 0.0066696280834686 -1.44717942993729

SERTM2 -1.29196869392807 -3.77640961321578 -3.1480965321443 0.00173553811843588 0.00668208857351809 -1.45488149757144

GABRB2 1.11868390175268 -1.42938289162802 3.13901129429008 0.00178923391663611 0.00684227945597651 -1.48518969493148

CCDC144A 1.32786199297302 -1.85531162298825 3.13784752143117 0.00179622163857841 0.00686281889922671 -1.48379379481258

FAM153B 1.30298980650679 -0.874999518425773 3.1311315727215 0.0018370404803981 0.00699669392973173 -1.51392700086799

FAM135B -1.0283929223281 -0.282094654664946 -3.12419825397105 0.00188007572858656 0.00713452029012267 -1.65576693341499

LINC02671 1.08031830042781 -4.00644178323451 3.12176156274542 0.00189541953969052 0.00717944766616129 -1.52252376484868

NTM 1.10188143821276 2.93267045587932 3.11476685575821 0.00194010814554041 0.00731768047201144 -1.80957289045511

F5 -1.10835658727793 1.89553924123966 -3.10207000607325 0.00202371448364244 0.00756616165672363 -1.94866262423993

SULT4A1 -1.37597084060413 -2.84021093586465 -3.09504955574061 0.00207135397645687 0.00771380017203366 -1.62591834115289

L1CAM -1.00122856524304 1.22147392217267 -3.0867559755787 0.0021289609853417 0.00789187564167793 -1.90891427518584

ADAMTS20 1.06006936908351 -3.69847913075834 3.07946046403347 0.00218084958654511 0.00804858476607789 -1.64643814065686

IGKV1.5 -1.06084099773304 2.89949673202656 -3.07641100054023 0.00220288111472069 0.00811750689421455 -2.18062283028341

SLC17A4 1.98240670471789 4.30149075679172 3.06891536774562 0.00225790717061846 0.00828800428041351 -2.01972751834337

IGKV2D.29 -1.21194137292549 -1.49752697267714 -3.06509912316823 0.00228640510271249 0.00837989221598633 -1.76220888133615

ADGRG2 1.0172546643049 1.59942817675584 3.05924529637497 0.00233076139953037 0.00850522772566263 -1.86049347168225

IGKV3.15 -1.11213033789102 1.30837414679819 -3.04591922169381 0.00243469974355658 0.00881358877731518 -2.04133722459745

AQP4 1.25189692375803 0.785432676814406 3.04454385917978 0.00244566576585525 0.00884592999756917 -1.83523025593368

DUSP9 -1.22507593167142 -2.26264623187443 -3.04193113547067 0.00246662243180296 0.00890302449149588 -1.79654682990351

ANGPTL7 -1.08112409718122 -3.92005531095371 -3.04154308958704 0.0024697489652913 0.00890757348814749 -1.762574928583

IGLV1.40 -1.10586707741886 2.340417486104 -3.03315692048592 0.00253821211129916 0.00911530822694588 -2.23062671601635

ADAMTS9.AS1 1.08720280193194 5.6902785733629 3.0319888309705 0.00254788509448875 0.00914725628430425 -2.43464535578067

FABP7 2.69938279056443 5.02754901107341 3.02368651380983 0.00261761685943508 0.00935133204978228 -2.15863415741768

LINC01234 -1.48636179771294 -2.62403375080928 -3.0211436956205 0.0026393215952342 0.00941074443821354 -1.84846087958171

KLC3 -1.0231426731084 -1.80727465303417 -3.02032710442948 0.00264632669198554 0.00942918099396023 -1.87046578273977

SLC7A10 1.03357333097816 -3.75188839659874 3.0186761666012 0.00266054128420482 0.00947048276913921 -1.8203569561448

ZAN 1.10715209862148 -4.01717283300592 3.0152372219561 0.00269037560008572 0.00955324725727012 -1.82924253611989

GSTA2 1.82404034500628 5.0479584036334 3.01386439307049 0.0027023708638125 0.00958802063449173 -2.32114653798077

FAM153A 1.5516976269899 0.482026779262742 3.00415358875946 0.0027886294360925 0.00983156736887357 -1.92014338688696

MTND1P23 -1.04179386744689 -0.0587515549675974 -3.00353363234057 0.00279422107362577 0.00984359070780476 -2.03115413463432

LINC02275 1.12007792305804 1.18921189850918 3.00098163776165 0.00281734684615752 0.00991194258935036 -1.99009892561674

IGHV3.74 -1.00001513894172 1.25251859426509 -3.00029496882856 0.00282359918665434 0.0099289170098693 -2.16096823391741

DRAIC 1.02800806332688 2.19563174250379 3.00015135004328 0.00282490848725688 0.0099289170098693 -2.08111584460869

LINC00593 1.02800806332688 2.19563174250379 3.00015135004328 0.00282490848725688 0.0099289170098693 -2.08111584460869

PCAT29 1.02800806332688 2.19563174250379 3.00015135004328 0.00282490848725688 0.0099289170098693 -2.08111584460869

IGLV3.21 -1.14664897242463 2.08080383089199 -2.99893100907106 0.00283605617733313 0.00996166448475232 -2.29284991775508

IGKV1.16 -1.16671946479269 0.146012539037378 -2.99730427052277 0.0028509789047254 0.0100092351321222 -2.06773823035247

SLC9A4 -1.21530725778626 -4.37106054313281 -2.99180414797764 0.00290196777217552 0.0101505917558966 -1.90178000451007

MXRA5Y 1.0848346238849 -3.01993444607016 2.98376835857271 0.00297796522931253 0.0103631285605829 -1.92140941789158

PADI1 1.43656026178489 1.03782980654956 2.98135631123604 0.00300112965865196 0.0104303990547465 -2.01880880909156

LOC102724058 1.07057362215422 -1.93233737976667 2.97895993880621 0.00302430668023016 0.010492522076597 -1.94134458740255

SCN1A.AS1 1.07057362215422 -1.93233737976667 2.97895993880621 0.00302430668023016 0.010492522076597 -1.94134458740255

LINC01060 1.36755090881893 -0.63605961321606 2.97548682048227 0.00305818836343018 0.0105830820519531 -1.96572768226968

KCNIP1 -1.038992156374 -2.00251442121684 -2.97446270105471 0.00306824513542965 0.0106145091962646 -1.99187861548342

SYT9 1.43768678064774 1.73309771535869 2.96524743482577 0.00316010815652241 0.0108838725068075 -2.11317322572263

TPSD1 1.2496952629339 -1.57127056191249 2.96151318475597 0.00319804385687492 0.0109884484029355 -1.99226236073603

IQSEC3 1.23600599413845 2.3889258606648 2.96078135233508 0.00320552703370962 0.0110077514790949 -2.19826400998658

USP32P1 1.35204214222245 -1.67476177153137 2.95369646421813 0.00327880357491741 0.0112163629444016 -2.01244080793203

IGKV1D.8 -1.09520491782404 -2.66487831236022 -2.94717751590181 0.00334757523469432 0.0114085909086569 -2.04509608569317

IGHV1.67 -1.02198790959396 -4.15644643914704 -2.94552960972464 0.00336516715976575 0.0114595734616116 -2.03122426088785

B4GALNT2 -1.23971557361007 -0.513719426003746 -2.94423220820092 0.00337907668528913 0.0114977683345521 -2.17912668179525

VWDE -1.10133397095712 -2.36648377853703 -2.940910111383 0.00341493273816237 0.011594578211134 -2.07297183385469

PRR15L -1.10433344821744 0.432052626099922 -2.94032299166958 0.00342130563833555 0.0116107826363657 -2.25874810086323

REN 2.31062538898082 3.19481055825851 2.93632109268691 0.00346503432646419 0.0117353978814604 -2.25938739067005

MUC20 1.02358780002675 3.64182797027692 2.93494095818962 0.00348023293570768 0.0117789945748255 -2.45510101593428

QRFPR 1.17271020217357 3.02381250917026 2.93438442803535 0.00348637886965313 0.0117966747793782 -2.33506241270775

IGHV4.30.2 -1.09689386572119 -0.554550531944144 -2.92448054473089 0.00359742007899697 0.0121042629741007 -2.2162467375052

IGLV2.18 -1.17286484910138 -1.87075943277232 -2.92283839289915 0.00361614042845912 0.0121620099978785 -2.14478721338386

IGLC3 -1.04783343946187 3.46576506791575 -2.91588628557527 0.00369638198446122 0.0123624955390585 -2.72760773095034

FABP6 1.1616969489718 3.40470446841631 2.91133798254727 0.00374975364782466 0.0125070315127489 -2.45303830418993

CDH4 1.6100194184864 3.34598969260372 2.90703655888987 0.00380087363046454 0.0126465326068972 -2.40556220068023

SLC23A3 1.28353162899791 3.40976809683436 2.90688874921089 0.00380264149815349 0.012648520374852 -2.45003514035837

IGHJ3 -1.06912187427714 -3.40200497198627 -2.9043264831236 0.00383340662528418 0.0127333682302092 -2.15022688682298

ZIC5 -1.00662742994911 -5.00509788016667 -2.90086902330876 0.00387527983589106 0.0128371961935536 -2.14982032203804

C1orf116 -1.0522227693047 0.0227526856221477 -2.90005751314195 0.00388516819856212 0.0128631412172286 -2.32683278334373

IGKV1.17 -1.1253441906692 0.2319910976687 -2.89794542057748 0.00391101218657496 0.0129280185672251 -2.35689022444798

IGLC2 -1.01514589990343 4.2588204902315 -2.89760437076256 0.00391519999341226 0.0129379320262038 -2.86191149270706

CYP4A11 2.47444609192687 3.59339368398104 2.89165077963913 0.00398896612701417 0.0131398041293459 -2.40624731371516

FOXJ1 -1.03534779968987 -2.67078375033318 -2.89098194429845 0.00399733170883924 0.0131571059127361 -2.19833010028508

LINC01018 -1.10148249696071 -2.36406826629506 -2.89065380609986 0.00400144179615883 0.0131629716755311 -2.21227964802889

IGLV2.23 -1.05378920474638 2.39775428733281 -2.8898724992846 0.00401124352822725 0.0131932211965734 -2.64611034361191

PKHD1 1.12172230576961 5.17243289822137 2.88676317674126 0.00405046789638584 0.0132905148392697 -2.78607634360187

SLC24A2 -1.0061100044298 -4.46364444678259 -2.88647562817762 0.00405411292613935 0.0132980524507648 -2.19160629630434

ISM2 -1.10930254742926 -2.44250048010392 -2.88065555281874 0.00412853424250374 0.0134832415388716 -2.23693959506069

IGHV3.30 -1.06720088851174 1.12297385484734 -2.87512239781772 0.00420043726163421 0.0136688470435031 -2.50353630280415

B4GALNT1 1.03694472253066 2.3015191658456 2.87495233228481 0.00420266518898189 0.0136740527905185 -2.44645608834558

PNCK 1.52854072849611 5.19475833215951 2.86872829789852 0.00428494584155388 0.0138940574802998 -2.79410737446703

LOC101927023 1.46576706368056 -0.0370303771132692 2.86401856637002 0.00434817836580328 0.0140566673699911 -2.28913367177237

IGHV3.21 -1.07150643563481 0.876097719964601 -2.86165954804446 0.00438016819824789 0.014141667334806 -2.51414940159376

LOC643201 -1.03430880241756 -4.30803676407628 -2.86119123840213 0.00438654417718078 0.0141601523216215 -2.26097374736039

IGLV3.9 -1.14682968572879 -1.07899835727793 -2.85900125463736 0.00441647265554394 0.0142314374971466 -2.36420966033154

IGLV3.10 -1.1612607012407 0.138644031778808 -2.85877174727131 0.0044196198396242 0.0142394708480018 -2.46014488738055

RNF224 1.01910157798619 -1.15644872534208 2.85773995858001 0.0044337937257716 0.0142661327463223 -2.2840042847026

GGT3P 1.02750209067692 -1.39688103771643 2.85501770383125 0.0044713883786193 0.0143616217850681 -2.2875007598583

CCNYL2 1.00365884663952 -0.0750226619192369 2.84062666467413 0.00467498626007476 0.0148637773052364 -2.37056055841347

SLC34A3 1.04517977012557 -0.622867182090103 2.84026295708427 0.00468023917457495 0.0148744000748975 -2.34447862028166

IGLV1.44 -1.03478141148181 1.81950246642332 -2.83817957547348 0.00471043219819799 0.0149572542170935 -2.69453012217871

LINC00462 1.47257410630996 2.45374488243019 2.83334242835871 0.00478121665089786 0.0151400556939327 -2.53014653852654

CD300LG 1.08426260363161 -0.0233629951366931 2.83199929479911 0.00480104192352457 0.0151851612248415 -2.39221956802978

IGHV3.48 -1.11810580560938 -0.911723333010466 -2.82986059190073 0.0048327641734899 0.0152611022434868 -2.45144515054201

CP 1.20677266852707 7.31939471882637 2.82184853323389 0.00495330066926714 0.0155782464293723 -3.12103270642623

SNAP91 -1.02558272032001 -4.69114152243831 -2.82153944577 0.00495800485371071 0.0155887464467313 -2.36498040523552

LRRTM1 -1.03654626100637 -5.41872945158456 -2.82119971877766 0.0049631800330354 0.0155982587033037 -2.36283981825375

VTCN1 -1.05816305427445 -1.20441998443617 -2.81728503991273 0.00502316815867775 0.0157663015587511 -2.46405900509643

REG3G -1.4020292372481 -3.69387209270153 -2.81401698409133 0.00507375015673261 0.0158976775140213 -2.39358780654001

HABP2 -1.2968089425242 3.03402005957257 -2.79706349990373 0.00534363770496902 0.0165643637032271 -3.03543375762235

PRSS3 -1.00471066585151 -3.62670514946626 -2.78691583361728 0.00551133636962364 0.0169970613144797 -2.46285263127524

A1CF 1.51759328213064 4.13861837887032 2.78028400251898 0.00562349171994508 0.0172817371548842 -2.87276949416005

SLC1A6 1.00944747854748 -4.42344811016885 2.76754326453039 0.00584477006928854 0.0178508643325668 -2.50212294668291

GRIN2A 1.24630502373448 2.22210098311643 2.76264116107935 0.00593198473102407 0.0180640055456353 -2.72244857987984

TRPM3 1.09178707662294 3.23312169017537 2.76059679660664 0.00596870281603929 0.0181402896113833 -2.85193553932142

AOC1 1.30754653876347 6.67232751165882 2.75773879121777 0.00602037867423193 0.0182769292331947 -3.27466376089754

IGHV6.1 -1.08557919801661 -2.93273392045861 -2.75312012975482 0.00610474402582872 0.0184814969694317 -2.56028821952555

ARSF 1.21305300554617 0.155434720420315 2.74813339987937 0.00619703015773188 0.0187140337740496 -2.61768703508577

IGLC7 -1.21630446653579 -1.87376105511883 -2.7459744396148 0.00623737391638548 0.0188175908576525 -2.6228245789082

SHISA9 1.13274145784211 3.24668615897595 2.74407369294137 0.00627308863730622 0.0189069953431521 -2.89326836910326

SCNN1G 1.40843687255399 -1.82914661581588 2.73628036866669 0.00642146196887659 0.0192422856107567 -2.59372271155004

IGKV2.29 -1.19364270148887 -4.13516985981528 -2.72536728545705 0.00663455310521424 0.0197696079832608 -2.62041457659451

XDH -1.17961755184023 -2.5855139054042 -2.718264558909 0.00677664638057038 0.020118284474342 -2.66119961851628

CYP2C9 -1.00033425433316 -1.30130759701669 -2.69995470971844 0.00715567561695784 0.0210449367052334 -2.76301782726338

AKR1B10 -1.21142106248011 -1.51757537884893 -2.69987779211274 0.00715730734494003 0.0210449367052334 -2.76170590131087

ADGRF1 -1.24327558935919 -1.54586847058276 -2.69911993958233 0.00717340232477789 0.021086218555495 -2.7661567835226

LINC03017 1.39952053598484 0.806655439995419 2.69827069697916 0.00719147691519097 0.0211336408819984 -2.77186769897339

UGT2B17 -1.01527148694882 -2.98621731900079 -2.68968734788924 0.00737647198159322 0.0215842327792019 -2.72126649219399

NDNF 1.64618051725589 0.242933538639339 2.68968430898488 0.00737653822963582 0.0215842327792019 -2.75349731505544

ORM1 -1.00895284727375 -4.17233058930333 -2.6849434441449 0.00748054307588793 0.0218360316417989 -2.72231883586794

SLC6A15 -1.09382807116239 -4.59710854867876 -2.68038527961309 0.00758178142617074 0.0220709016421735 -2.73179841396745

IGKV3.7 -1.00774929986011 -2.96219952704419 -2.67612910502183 0.00767742279328492 0.0222938182952819 -2.75600598993585

PI3 -1.15628014736385 -2.31137818884065 -2.67448447478385 0.00771466913153298 0.0223918900838707 -2.78387644961648

SLC5A10 1.16980090126681 4.65434532138953 2.6553412961547 0.00816030958182564 0.0235076495620535 -3.3231797634104

IGHV1.69 -1.13846360019172 -1.29339648058726 -2.65339989307516 0.00820677040348872 0.023610464098961 -2.88822191326934

IGKV2.24 -1.01174187416145 -0.263268626185198 -2.64866838194262 0.00832099871563674 0.0238791270986506 -2.9667649154847

HHLA2 1.27962852833245 5.01568110917775 2.64663447334351 0.00837053826378921 0.0239991446770153 -3.38371178635221

CD5L -1.18543409032202 -1.19285784937754 -2.64522265841443 0.00840508098049828 0.0240854920619413 -2.91916606807086

ESRP1 -1.26607614242774 -1.65198395843985 -2.64513661814365 0.00840719024566806 0.0240883651543315 -2.89838364016773

UNC93A -1.24889114089773 -3.47271553582273 -2.62983005208807 0.0087900701689399 0.0249847815836138 -2.86778143626031

ALKAL2 1.15945308405305 2.956385306693 2.61856880507043 0.00908165887355598 0.0256594585540879 -3.18514581253585

APOC3 -1.14657000188282 -4.00990084373438 -2.61825385312283 0.00908993701342418 0.0256795144673854 -2.89111476720549

IGHV2.70 -1.11960028090848 -1.73854139961771 -2.61429981960896 0.00919444039669708 0.0259242725537442 -2.96072070855712

LHCGR -1.08195624546048 -4.66724858550966 -2.61061880759972 0.00929269310249996 0.0261437315189501 -2.90554233456078

CLCNKB -1.05122888964427 -0.371513911288773 -2.60828041643943 0.0093555958383919 0.0262969078789097 -3.07532028707863

NR2E1 1.30615588713076 -1.94554965068988 2.5995682974415 0.00959331878299407 0.0268334322731216 -2.93771638548883

DMRT2 -1.24059879856906 -3.62898281253151 -2.59580010675442 0.00969780205729093 0.0270769699052144 -2.95030991036004

IGKV3D.20 -1.01784135876888 -1.45689852271087 -2.59396511789878 0.00974904941797837 0.027164306026771 -3.0227496395437

SLC22A6 1.97602534548863 1.83739001728328 2.59269242875433 0.00978473495044423 0.0272393305622611 -3.07763743066103

RNU1.14P 1.0319488770248 -0.473679264189368 2.59011204495355 0.00985744603452914 0.0273981020415 -2.99273408626806

FDCSP -1.26700139832241 -4.49300067084306 -2.58994800742826 0.00986208462893391 0.0274055912317125 -2.95821812161336

PCSK1N -1.04928886247852 1.19841031647475 -2.57192792376913 0.0103836803649074 0.028617570334634 -3.32035960614143

PRAME -1.25282798655173 1.36972988712948 -2.56788967959997 0.0105038918067057 0.0288611730568748 -3.36676534302734

CREB3L3 1.40264885099983 2.92494387375196 2.56141143610811 0.0106993324501681 0.029297542018331 -3.29508758132913

KCNS1 -1.27967209366911 -2.28495057171126 -2.55724793122355 0.0108266456399506 0.0295577067157663 -3.08073789715426

LINC02294 1.2837541547341 -2.35183535153569 2.55554684175851 0.0108790495769455 0.0296784399224681 -3.04194474504734

FGF9 -1.15019101673536 -3.0160284311111 -2.54672699977782 0.0111543959681872 0.0303156120663667 -3.07752102690842

NKAIN4 -1.0530742277587 1.35818078267761 -2.54654854158884 0.0111600307490376 0.0303271397396131 -3.39553468896836

LHX8 1.16192592882223 -3.62079606351579 2.54029279384551 0.0113591637545407 0.0307551081167518 -3.07256799460708

LRP2 1.06636900897275 8.06370132332008 2.53579962445389 0.0115041361114465 0.0310721591683654 -3.86905913834109

NEFL -1.01092736580917 3.95194802666087 -2.51033638616591 0.0123572399401063 0.0329349873608784 -3.85827936994652

SLC22A24 1.15277652756952 -1.64178420317223 2.50915940459476 0.0123979975812606 0.0330193580510582 -3.16004795981015

HAO2 1.35290782227668 2.90978866692221 2.47682879655545 0.0135652708165264 0.0355624774013405 -3.51112381384981

PKP3 -1.00397143098489 -0.732486620210777 -2.47401437184757 0.0136713495822555 0.0357888474271745 -3.36212988976262

IGHV1.3 -1.00279655375633 -3.58190224543384 -2.4706629236734 0.0137986273058073 0.0360353593959818 -3.24847554745985

FTCD 1.44212226355362 2.88540267167591 2.46662008631741 0.0139535573842572 0.0364050202065357 -3.52579078908407

NOG 1.15038950575179 -0.72472479182709 2.45138395106628 0.0145513643264912 0.0376891417533002 -3.31257439113029

C4BPA -1.00906804014419 -3.12200487523761 -2.44995726017282 0.0146084851779445 0.0378190885904279 -3.30182717639937

IGKV6.21 -1.01066868333485 -2.68610518041264 -2.44886471695438 0.0146523615947244 0.0379191481194109 -3.31374776910089

KNG1 -1.17839036835106 -2.1975071262523 -2.44147854429609 0.0149520574313238 0.0385343871782648 -3.35808591374589

CHST9 1.18308232616174 3.1969908576926 2.4349163462999 0.0152228500524317 0.039116488855068 -3.66304461437953

TRIM15 1.00409500033344 2.71071589211214 2.42619902780089 0.0155892676539827 0.039879138444499 -3.63389091434962

HEPACAM2 -1.13254986286655 -4.03575978881302 -2.41846530601228 0.0159208334512516 0.0405794826385706 -3.36660262332893

RNF186 1.13028738726657 3.22593411870417 2.41758235031201 0.0159590808977824 0.0406453130454224 -3.71312728803572

SLC17A3 1.21031656073952 6.38144720243689 2.39459133181807 0.0169839284535531 0.0427336921336829 -4.18177502260052

IGLV4.60 -1.09804070426496 -2.41803966841088 -2.39294589302628 0.0170594486750884 0.0428944914130704 -3.45344690902296

CCL20 1.07126248545305 2.77437666219436 2.38846631414596 0.0172665457548429 0.0432448396554672 -3.72036703098602

MGARP 1.04147918461388 1.08221877342276 2.38162916894832 0.0175868999800544 0.043905729081987 -3.58341258176942

IGHV1.24 -1.04064321842088 -0.174133450341567 -2.37225219867019 0.0180347492826039 0.0447874943108316 -3.64546594795431

CIDEC -1.00824934475272 -3.0118474780443 -2.3714666149657 0.0180727200301381 0.0448500553652771 -3.48282536281867

SLITRK5 1.11243025341083 1.11373895717727 2.37119655653343 0.0180857893606937 0.0448681760900638 -3.60474635508517

CPN1 -1.01915996074581 -4.30014865510621 -2.35896884254505 0.0186863059365366 0.0460483212182263 -3.49847423236726

LINC00278 -1.20293907356939 -2.86074024784115 -2.35437103165842 0.0189165946471875 0.046552573847974 -3.52871207847408

ASPG 1.4069489376654 2.16709407506374 2.34525286356976 0.0193806649994713 0.0474425253085348 -3.73666666838019

ZFY.AS1 -1.25152960079287 -3.06714594909675 -2.33562159234686 0.0198816543960287 0.0484509596102056 -3.56618883055768

ADAM18 1.12758849327899 -1.53111498544079 2.32859392579607 0.0202543324320098 0.0491265637805394 -3.57495410202093

LOC101928335 1.01504955983144 -0.503758981722643 2.31567266990716 0.0209555204352561 0.0504801696894887 -3.63156443453067

ATP6V1G3 -1.06594801939014 -5.32561423671716 -2.31543504403667 0.0209686117213955 0.0505061161159251 -3.58930580359323

SLC22A12 1.69310689591703 3.05435174683658 2.31379756496389 0.02105901819964 0.0506566083712635 -3.8718157747182

ADGRF4 -1.0247153453909 -2.76273944452638 -2.3136038646304 0.0210697350382321 0.0506767870251048 -3.61730732862916

SLC5A1 1.47829190896319 3.88358081422191 2.31024900831413 0.0212561062600159 0.0510337403754825 -4.0058441474507

KRT6A -1.03996554306175 -4.1490128216309 -2.3083035093847 0.0213648422402443 0.0512581624412117 -3.61154537899001

NAT8 1.06589267842976 6.8115178511597 2.30429576436047 0.0215903713664451 0.0516948205404699 -4.41693196961006

RGS7 1.22935503727592 -2.46939702965721 2.30361794665019 0.0216287191972765 0.0517411067993316 -3.62039403217296

LOC100505985 1.43331535271749 -1.20467067939186 2.30192248440412 0.0217249011808348 0.0519312457723734 -3.63548077398115

SLC5A12 1.36659027077648 4.88235907553853 2.29719035045774 0.0219953283153569 0.0525084789910479 -4.20378709886714

PROM1 -1.10534878325061 3.53761977363858 -2.29224738589461 0.0222809351324254 0.0530738799548593 -4.32928241815728

SLC10A2 1.48442035990849 2.26649647451891 2.29113934289127 0.022345400314785 0.0531808800187977 -3.85879986006461

SLC5A8 1.79250121702094 3.01433800104523 2.28312850397747 0.0228163222556038 0.0540416633882185 -3.9237814733601

LBP -1.22013058162744 0.336139254271429 -2.26469366373781 0.02393299138764 0.0561549448673881 -3.94906717906016

PCK1 1.15986105147031 5.27429450356749 2.2503264087133 0.0248359397758278 0.0579363306291333 -4.41000295633704

UPK1B -1.11597812211007 -1.0741863724626 -2.23736676791809 0.025675657711638 0.0594935109015567 -3.87748605606106

LINC02577 -1.22133871760626 -3.14077230554493 -2.21611268441062 0.0271061516882296 0.0621662210302069 -3.82197006658397

GRIA4 1.42896686511175 1.35788459589126 2.20350173816951 0.0279871271145164 0.0638302384641322 -3.96708397252249

BMP5 1.07871129270993 -2.07734868625555 2.18999838765362 0.0289577787919542 0.0655406752352594 -3.86634826413628

TEX15 1.41823605884431 -0.146767881786334 2.18864779400877 0.0290564425474994 0.0657249906402806 -3.90615828833041

VIL1 1.10801818176841 2.88530252432389 2.00993514713318 0.0449423923853289 0.0938491795055586 -4.54690646682842

TMEM174 1.27698204263827 1.67075551491528 1.99984613750857 0.0460248085843709 0.0955481171631857 -4.42358713338827

C1QL1 1.03546704597415 3.18865409679634 1.99838459668948 0.0461834236662715 0.095822570102348 -4.61690587809162
