# Supplementary material for: DNER drives glycolytic reprogramming in renal cell carcinoma by activating the JAK2/STAT3 signaling pathway
Source: Front Immunol. 2026 May 22;17:1799104. doi: 10.3389/fimmu.2026.1799104 (PMC13236898; doi:10.3389/fimmu.2026.1799104)
Supplement: Supplementary file 6 [file Table4.docx]

Category Term Count % PValue Genes List Total Pop Hits Pop Total Fold Enrichment Bonferroni Benjamini FDR

GOTERM_CC_ALL GO:0071944~cell periphery 158 47.59036144578313 5.019527983314352E-13 RAB3B, KCNG3, HPSE2, DGKB, AQP6, AQP4, SLC4A3, NALF2, RIMS2, LIPH, ADORA1, ENPP1, SLC16A7, ENPP3, MALRD1, IL13RA2, SH3GL2, RGS7, CCBE1, CHRNB4, SLC15A1, SLC30A2, SLC6A15, WNT5A, KCNK13, DIO1, KCNK15, VWDE, UNC93A, PRLR, PSG9, SLC9A2, SLC9A3, SCNN1G, SLC9A4, C1ORF116, PLPP4, EMCN, SLPI, PSG4, DOK7, CLIP4, ADTRP, PKP3, RHCG, ATP6V0D2, TMEM63C, MTTP, IL20RA, CLCNKB, VTCN1, SLC6A20, LRRC52, KNG1, VTN, SCIN, PODXL, INPP5J, DRD1, PROM1, SLC38A3, TRPM6, WNT2, CD300LG, SLC38A4, SPTBN2, SYT3, ST14, AFAP1L2, ST8SIA2, L1CAM, BMP7, SNAP91, IGSF11, BFSP2, GJB3, LYPD6B, LRRN1, CNTN3, XKR4, MUC20, DPP10, GABRB2, PIGR, DOCK3, TENM3, PSCA, DIRAS1, GRIK5, DUSP15, SLC7A10, CHRDL1, GRM1, GPRC6A, EPB41L4B, PANX2, UCHL1, UPK1B, NMRK2, LRRTM1, DNER, CA4, CA9, ERC2, SLC13A3, CR2, PLA2G4F, CR1, SLC36A2, AHSG, SLC2A12, ANO5, SSTR5, F5, EPN3, SLC7A4, ADGRF1, SLC7A8, ANGPTL7, RGS7BP, CDH17, HCN4, COLEC12, SLC24A2, POPDC3, DAPL1, PTGER3, ITLN1, TMPRSS2, NDNF, SEMA3E, CST6, C10ORF90, SCEL, WNT11, IGFBPL1, SV2B, NCAM2, CTNNA2, ATP6V1G3, RHBG, SNCA, COL26A1, MCOLN3, KCNJ10, KCNIP1, PDE6G, GCGR, KCNIP3, MYO16, GPR143, NFASC, GLRB, KCNS1, SMOC1, TEK, HCN2, TJP3 299 6783 20791 1.6197181918005716 2.2386159592713284E-10 2.2387094805582011E-10 2.0580064731588846E-10

GOTERM_MF_ALL GO:0015318~inorganic molecular entity transmembrane transporter activity 40 12.048192771084338 1.875060233364328E-11 HCN4, SLC24A2, GABRB2, KCNG3, TMEM63C, GRIK5, CLCNKB, AQP6, AQP4, NALF2, SLC4A3, SLC6A20, LRRC52, ATP6V1G3, SLC38A3, TRPM6, RHBG, SLC12A8, SLC38A4, SLC13A3, SLC15A1, MCOLN3, SLC36A2, KCNJ10, KCNIP1, SLC30A2, SLC6A15, KCNIP3, KCNK13, KCNK15, ANO5, SLC9A2, SCNN1G, SLC9A3, SLC9A4, KCNS1, GLRB, RHCG, ATP6V0D2, HCN2 289 758 19144 3.4956313737663316 1.5712966372483095E-8 1.5731755357926712E-8 1.4944230059913693E-8

GOTERM_CC_ALL GO:0005886~plasma membrane 145 43.674698795180724 2.7226015068983615E-11 RAB3B, KCNG3, HPSE2, DGKB, AQP6, AQP4, SLC4A3, NALF2, RIMS2, LIPH, ADORA1, ENPP1, SLC16A7, ENPP3, MALRD1, IL13RA2, SH3GL2, RGS7, CHRNB4, SLC15A1, SLC30A2, SLC6A15, WNT5A, KCNK13, DIO1, KCNK15, VWDE, UNC93A, PRLR, PSG9, SLC9A2, SLC9A3, SCNN1G, SLC9A4, C1ORF116, PLPP4, EMCN, PSG4, DOK7, ADTRP, PKP3, RHCG, ATP6V0D2, TMEM63C, MTTP, IL20RA, CLCNKB, VTCN1, SLC6A20, LRRC52, KNG1, VTN, PODXL, INPP5J, DRD1, PROM1, SLC38A3, TRPM6, CD300LG, SLC38A4, SPTBN2, SYT3, ST14, AFAP1L2, ST8SIA2, L1CAM, SNAP91, IGSF11, BFSP2, GJB3, LYPD6B, CNTN3, XKR4, MUC20, DPP10, GABRB2, PIGR, DOCK3, TENM3, PSCA, DIRAS1, GRIK5, DUSP15, SLC7A10, CHRDL1, GRM1, GPRC6A, EPB41L4B, PANX2, UCHL1, UPK1B, NMRK2, LRRTM1, DNER, CA4, CA9, ERC2, SLC13A3, CR2, PLA2G4F, CR1, SLC36A2, SLC2A12, ANO5, SSTR5, F5, EPN3, SLC7A4, ADGRF1, SLC7A8, RGS7BP, CDH17, HCN4, COLEC12, SLC24A2, POPDC3, DAPL1, PTGER3, ITLN1, TMPRSS2, SEMA3E, CST6, C10ORF90, SCEL, SV2B, NCAM2, CTNNA2, ATP6V1G3, RHBG, SNCA, COL26A1, MCOLN3, KCNJ10, KCNIP1, PDE6G, GCGR, KCNIP3, MYO16, GPR143, NFASC, GLRB, KCNS1, TEK, HCN2, TJP3 299 6269 20791 1.6083254064833543 1.2142795546843388E-8 6.071401360383346E-9 5.581333089141641E-9

GOTERM_MF_ALL GO:0022857~transmembrane transporter activity 50 15.060240963855422 7.050171157521375E-11 GABRB2, KCNG3, GRIK5, AQP6, AQP4, NALF2, SLC4A3, SLC7A10, PANX2, SLC16A7, SLC12A8, SLC15A1, SLC13A3, CHRNB4, SLC36A2, SLC30A2, SLC6A15, KCNK13, SLC2A12, KCNK15, ANO5, SLC7A4, SLC9A2, SLC9A3, SCNN1G, SLC9A4, SLC7A8, RHCG, ATP6V0D2, CDH17, HCN4, SLC24A2, TMEM63C, CLCNKB, SLC6A20, LRRC52, SV2B, ATP6V1G3, SLC38A3, TRPM6, RHBG, SLC38A4, MCOLN3, KCNJ10, KCNIP1, KCNIP3, GLRB, KCNS1, GJB3, HCN2 289 1175 19144 2.8188176396966793 5.908043587687928E-8 2.9575468005802168E-8 2.809493206272268E-8

GOTERM_BP_ALL GO:0055085~transmembrane transport 52 15.66265060240964 1.0655957168663629E-10 GABRB2, KCNG3, GRIK5, AQP6, AQP4, NALF2, SLC4A3, SLC7A10, GRM1, PANX2, SLC16A7, SLC12A8, SLC15A1, SLC13A3, CHRNB4, SLC36A2, SLC30A2, SLC6A15, KCNK13, SLC2A12, KCNK15, ANO5, SLC7A4, SLC9A2, SLC9A3, SCNN1G, SLC9A4, SLC7A8, RHCG, ATP6V0D2, CDH17, HCN4, SLC24A2, TMEM63C, CLCNKB, SLC6A20, LRRC52, SV2B, DRD1, ATP6V1G3, SLC38A3, TRPM6, RHBG, SLC38A4, MCOLN3, KCNJ10, KCNIP1, KCNIP3, GLRB, KCNS1, GJB3, HCN2 286 1312 19554 2.7098115299334813 4.1632803216007375E-7 4.1622168700800137E-7 4.090821957049967E-7

GOTERM_CC_ALL GO:0098590~plasma membrane region 51 15.36144578313253 3.3711639144835046E-10 GABRB2, DGKB, GRIK5, AQP6, AQP4, GRM1, RIMS2, UPK1B, LRRTM1, CA4, ADORA1, ENPP1, SLC16A7, CA9, ENPP3, ERC2, SLC15A1, SLC13A3, PLA2G4F, CHRNB4, CR1, SLC9A2, SLC9A3, SCNN1G, SLC9A4, SLC7A8, RGS7BP, ADTRP, RHCG, ATP6V0D2, CDH17, MTTP, ITLN1, SLC6A20, PODXL, DRD1, PROM1, SLC38A3, TRPM6, RHBG, CD300LG, SLC38A4, SPTBN2, ST14, KCNJ10, PDE6G, SNAP91, GPR143, GLRB, TEK, MUC20 299 1331 20791 2.66438089398923 1.5035392353368593E-7 5.011797019532143E-8 4.6072573497941227E-8

GOTERM_MF_ALL GO:0005215~transporter activity 51 15.36144578313253 5.010737842367855E-10 GABRB2, KCNG3, GRIK5, AQP6, AQP4, NALF2, SLC4A3, SLC7A10, PANX2, SLC16A7, SLC12A8, SLC15A1, SLC13A3, CHRNB4, SLC36A2, SLC30A2, SLC6A15, KCNK13, SLC2A12, KCNK15, ANO5, SLC7A4, SLC9A2, SLC9A3, SCNN1G, SLC9A4, SLC7A8, RHCG, ATP6V0D2, CDH17, HCN4, SLC24A2, TMEM63C, MTTP, CLCNKB, SLC6A20, LRRC52, SV2B, ATP6V1G3, SLC38A3, TRPM6, RHBG, SLC38A4, MCOLN3, KCNJ10, KCNIP1, KCNIP3, GLRB, KCNS1, GJB3, HCN2 289 1286 19144 2.627024060012808 4.1989970445133196E-7 1.4013363499155434E-7 1.3311860201223936E-7

GOTERM_BP_ALL GO:0098660~inorganic ion transmembrane transport 36 10.843373493975903 1.124545887235461E-9 HCN4, SLC24A2, GABRB2, KCNG3, CLCNKB, AQP6, NALF2, SLC4A3, SLC6A20, LRRC52, ATP6V1G3, TRPM6, RHBG, SLC12A8, SLC38A4, SLC13A3, SLC15A1, MCOLN3, SLC36A2, KCNJ10, KCNIP1, SLC30A2, SLC6A15, KCNIP3, KCNK13, KCNK15, ANO5, SLC9A2, SCNN1G, SLC9A3, SLC9A4, KCNS1, GLRB, RHCG, ATP6V0D2, HCN2 286 746 19554 3.2993869401376106 4.393591185203327E-6 2.1962381177708554E-6 2.1585658305484673E-6

GOTERM_MF_ALL GO:0022890~inorganic cation transmembrane transporter activity 33 9.939759036144578 2.0963654903782625E-9 HCN4, SLC24A2, KCNG3, TMEM63C, GRIK5, NALF2, SLC6A20, LRRC52, ATP6V1G3, SLC38A3, TRPM6, RHBG, SLC12A8, SLC38A4, SLC13A3, SLC15A1, MCOLN3, SLC36A2, KCNJ10, KCNIP1, SLC30A2, SLC6A15, KCNIP3, KCNK13, KCNK15, SLC9A2, SCNN1G, SLC9A3, SLC9A4, KCNS1, RHCG, ATP6V0D2, HCN2 289 632 19144 3.458849809469581 1.7567527692552432E-6 4.397126616068406E-7 4.177008239578688E-7

GOTERM_MF_ALL GO:0015075~monoatomic ion transmembrane transporter activity 37 11.144578313253012 2.8116355360902014E-9 HCN4, SLC24A2, GABRB2, KCNG3, TMEM63C, GRIK5, CLCNKB, NALF2, SLC4A3, SLC6A20, LRRC52, ATP6V1G3, SLC38A3, TRPM6, SLC12A8, SLC38A4, SLC13A3, SLC15A1, MCOLN3, CHRNB4, SLC36A2, KCNJ10, KCNIP1, SLC30A2, SLC6A15, KCNIP3, KCNK13, KCNK15, ANO5, SLC9A2, SCNN1G, SLC9A3, SLC9A4, KCNS1, GLRB, ATP6V0D2, HCN2 289 786 19144 3.1182721853896473 2.3561478528222324E-6 4.7179244295593585E-7 4.481747044527781E-7

GOTERM_MF_ALL GO:0015079~potassium ion transmembrane transporter activity 17 5.120481927710843 4.148071744447809E-9 HCN4, SLC24A2, KCNG3, MCOLN3, KCNJ10, KCNIP1, KCNIP3, GRIK5, KCNK13, KCNK15, LRRC52, SLC9A2, SLC9A3, SLC9A4, KCNS1, HCN2, SLC12A8 289 165 19144 6.824955436720142 3.4760781130183105E-6 5.80038698931952E-7 5.510021967208173E-7

GOTERM_BP_ALL GO:0006810~transport 98 29.518072289156628 6.469262008392412E-9 RAB3B, KCNG3, AQP6, AQP4, SLC4A3, NALF2, RIMS2, FGF9, ADORA1, ENPP1, SLC16A7, PTGDS, SH3GL2, SLC12A8, CHRNB4, SLC15A1, SLC30A2, SLC6A15, KCNK13, KCNK15, PRLR, SLC9A2, SLC9A3, SCNN1G, SLC9A4, PLPP4, RHCG, ATP6V0D2, CES1, CALCA, TMEM63C, MTTP, CLCNKB, SLC6A20, LRRC52, VTN, KLC3, SCIN, DRD1, SLC38A3, TRPM6, B4GALNT2, SLC38A4, SPTBN2, SYT3, NR0B2, SNAP91, GJB3, LCN2, XKR4, GABRB2, PIGR, GRIK5, SLC7A10, GRM1, PANX2, UCHL1, LRRTM1, DNER, CA4, CA9, KIF1A, ERC2, SLC13A3, PLA2G4F, CR1, SLC36A2, AHSG, SLC2A12, SYN3, SYNGR3, ANO5, EPN3, SLC7A4, GAL, SLC7A8, CDH17, HCN4, COLEC12, SLC24A2, SV2B, IGF2BP2, ATP6V1G3, RHBG, SNCA, MCOLN3, KCNJ10, KCNIP1, SPAG6, GCGR, KCNIP3, LGI3, GPR143, GLRB, KCNS1, FABP7, CLNK, HCN2 286 3842 19554 1.7439671208541587 2.5275087353837655E-5 6.379591686068393E-6 6.270161925964302E-6

GOTERM_BP_ALL GO:0034220~monoatomic ion transmembrane transport 37 11.144578313253012 6.5331200062144325E-9 HCN4, SLC24A2, GABRB2, KCNG3, TMEM63C, GRIK5, CLCNKB, NALF2, SLC4A3, SLC6A20, LRRC52, PANX2, ATP6V1G3, TRPM6, SLC12A8, SLC38A4, SLC13A3, SLC15A1, MCOLN3, CHRNB4, SLC36A2, KCNJ10, KCNIP1, SLC30A2, SLC6A15, KCNIP3, KCNK13, KCNK15, ANO5, SLC9A2, SCNN1G, SLC9A3, SLC9A4, KCNS1, GLRB, ATP6V0D2, HCN2 286 838 19554 3.0187509387986715 2.552457434146227E-5 6.379591686068393E-6 6.270161925964302E-6

GOTERM_MF_ALL GO:0015267~channel activity 28 8.433734939759036 2.3442177408549523E-8 HCN4, SLC24A2, GABRB2, KCNG3, TMEM63C, GRIK5, CLCNKB, AQP6, AQP4, NALF2, LRRC52, PANX2, TRPM6, RHBG, MCOLN3, CHRNB4, KCNJ10, KCNIP1, KCNIP3, KCNK13, KCNK15, ANO5, SCNN1G, KCNS1, GLRB, GJB3, RHCG, HCN2 289 519 19144 3.573761092332207 1.9644351956671358E-5 2.576443224544437E-6 2.4474675208127724E-6

GOTERM_MF_ALL GO:0022803~passive transmembrane transporter activity 28 8.433734939759036 2.45668007107932E-8 HCN4, SLC24A2, GABRB2, KCNG3, TMEM63C, GRIK5, CLCNKB, AQP6, AQP4, NALF2, LRRC52, PANX2, TRPM6, RHBG, MCOLN3, CHRNB4, KCNJ10, KCNIP1, KCNIP3, KCNK13, KCNK15, ANO5, SCNN1G, KCNS1, GLRB, GJB3, RHCG, HCN2 289 520 19144 3.566888474846952 2.0586767333075073E-5 2.576443224544437E-6 2.4474675208127724E-6

GOTERM_BP_ALL GO:0098739~import across plasma membrane 16 4.819277108433735 2.7918982324620058E-8 HCN4, SLC24A2, SLC15A1, KCNJ10, NALF2, SLC6A20, GRM1, SLC9A2, SCNN1G, SLC9A3, SLC9A4, SLC7A8, SLC38A3, HCN2, SLC12A8, SLC38A4 286 169 19554 6.472958993669053 1.0907351660860432E-4 1.9682248578782486E-5 1.9344637044020986E-5

GOTERM_BP_ALL GO:0071805~potassium ion transmembrane transport 16 4.819277108433735 3.0233868784612114E-8 HCN4, SLC24A2, KCNG3, MCOLN3, KCNJ10, KCNIP1, KCNIP3, KCNK13, KCNK15, LRRC52, SLC9A2, SLC9A3, SLC9A4, KCNS1, HCN2, SLC12A8 286 170 19554 6.434882764294529 1.1811675062756155E-4 1.9682248578782486E-5 1.9344637044020986E-5

GOTERM_MF_ALL GO:0008324~monoatomic cation transmembrane transporter activity 32 9.63855421686747 3.080711522764397E-8 HCN4, SLC24A2, KCNG3, TMEM63C, GRIK5, NALF2, SLC6A20, LRRC52, ATP6V1G3, SLC38A3, TRPM6, SLC12A8, SLC38A4, SLC13A3, SLC15A1, MCOLN3, CHRNB4, SLC36A2, KCNJ10, KCNIP1, SLC30A2, SLC6A15, KCNIP3, KCNK13, KCNK15, SLC9A2, SCNN1G, SLC9A3, SLC9A4, KCNS1, ATP6V0D2, HCN2 289 670 19144 3.163807261271497 2.5816029689318398E-5 2.8719077417770325E-6 2.728141204048027E-6

GOTERM_MF_ALL GO:0046873~metal ion transmembrane transporter activity 26 7.83132530120482 4.05311242283457E-8 HCN4, SLC24A2, KCNG3, GRIK5, NALF2, SLC6A20, LRRC52, SLC38A3, TRPM6, SLC12A8, SLC38A4, SLC13A3, MCOLN3, KCNJ10, KCNIP1, SLC30A2, SLC6A15, KCNIP3, KCNK13, KCNK15, SLC9A2, SCNN1G, SLC9A3, SLC9A4, KCNS1, HCN2 289 464 19144 3.711848228135068 3.3964505990025096E-5 3.4005613227582044E-6 3.2303306009991523E-6

GOTERM_BP_ALL GO:0051234~establishment of localization 100 30.120481927710845 6.987401795656455E-8 RAB3B, KCNG3, AQP6, AQP4, SLC4A3, NALF2, RIMS2, FGF9, ADORA1, ENPP1, SLC16A7, PTGDS, SH3GL2, SLC12A8, CHRNB4, SLC15A1, SLC30A2, SLC6A15, KCNK13, KCNK15, PRLR, SLC9A2, SLC9A3, SCNN1G, SLC9A4, PLPP4, CLIP4, RHCG, ATP6V0D2, CES1, CALCA, TMEM63C, MTTP, CLCNKB, SLC6A20, LRRC52, VTN, KLC3, SCIN, DRD1, SLC38A3, TRPM6, B4GALNT2, SLC38A4, SPTBN2, SYT3, NR0B2, SNAP91, GJB3, LCN2, XKR4, GABRB2, PIGR, GRIK5, SLC7A10, GRM1, PANX2, UCHL1, LRRTM1, DNER, CA4, CA9, KIF1A, ERC2, SLC13A3, PLA2G4F, CR1, SLC36A2, AHSG, SLC2A12, SYN3, SYNGR3, ANO5, EPN3, SLC7A4, ANKFN1, GAL, SLC7A8, CDH17, HCN4, COLEC12, SLC24A2, SV2B, IGF2BP2, ATP6V1G3, RHBG, SNCA, MCOLN3, KCNJ10, KCNIP1, SPAG6, GCGR, KCNIP3, LGI3, GPR143, GLRB, KCNS1, FABP7, CLNK, HCN2 286 4138 19554 1.6522626720790083 2.7296053698477873E-4 3.898970201976302E-5 3.8320907847893045E-5

GOTERM_BP_ALL GO:0006811~monoatomic ion transport 39 11.74698795180723 8.088305879346986E-8 HCN4, SLC24A2, GABRB2, KCNG3, TMEM63C, GRIK5, CLCNKB, NALF2, SLC4A3, SLC6A20, LRRC52, PANX2, ATP6V1G3, SLC38A3, TRPM6, SLC12A8, SLC38A4, SLC13A3, SLC15A1, MCOLN3, CHRNB4, SLC36A2, KCNJ10, KCNIP1, SLC30A2, SLC6A15, KCNIP3, KCNK13, KCNK15, ANO5, SLC9A2, SCNN1G, SLC9A3, SLC9A4, KCNS1, GLRB, LCN2, ATP6V0D2, HCN2 286 1010 19554 2.6400540054005397 3.159601975957216E-4 3.949115345591166E-5 3.881375783851635E-5

GOTERM_BP_ALL GO:0098662~inorganic cation transmembrane transport 30 9.036144578313253 9.793313492269897E-8 HCN4, SLC24A2, KCNG3, NALF2, SLC6A20, LRRC52, ATP6V1G3, TRPM6, RHBG, SLC12A8, SLC38A4, SLC13A3, SLC15A1, MCOLN3, SLC36A2, KCNJ10, KCNIP1, SLC30A2, SLC6A15, KCNIP3, KCNK13, KCNK15, SLC9A2, SCNN1G, SLC9A3, SLC9A4, KCNS1, RHCG, ATP6V0D2, HCN2 286 650 19554 3.1555675094136633 3.8255158540179135E-4 4.250298055645136E-5 4.1773922774249036E-5

GOTERM_BP_ALL GO:0006813~potassium ion transport 16 4.819277108433735 1.2351260909867353E-7 HCN4, SLC24A2, KCNG3, MCOLN3, KCNJ10, KCNIP1, KCNIP3, KCNK13, KCNK15, LRRC52, SLC9A2, SLC9A3, SLC9A4, KCNS1, HCN2, SLC12A8 286 189 19554 5.787989787989788 4.824473785318695E-4 4.8244025113941885E-5 4.7416490632980764E-5

GOTERM_BP_ALL GO:0051179~localization 109 32.831325301204814 2.1299266252047242E-7 RAB3B, KCNG3, AQP6, AQP4, SLC4A3, NALF2, RIMS2, FGF9, ADORA1, ENPP1, SLC16A7, PTGDS, SH3GL2, SLC12A8, CHRNB4, SLC15A1, DGAT2, SLC30A2, SLC6A15, WNT5A, KCNK13, KCNK15, PRLR, SLC9A2, SLC9A3, SCNN1G, SLC9A4, PLPP4, CLIP4, PKP3, RHCG, ATP6V0D2, CES1, CALCA, TMEM63C, MTTP, CLCNKB, SLC6A20, LRRC52, VTN, KLC3, SCIN, DRD1, SLC38A3, TRPM6, B4GALNT2, SLC38A4, SPTBN2, SYT3, NR0B2, SNAP91, IGSF11, GJB3, LCN2, XKR4, DPP10, GABRB2, PIGR, GRIK5, SLC7A10, CHRDL1, GRM1, PANX2, UCHL1, LRRTM1, DNER, CA4, CA9, KIF1A, ERC2, SLC13A3, PLA2G4F, CR1, SLC36A2, AHSG, SLC2A12, SYN3, SYNGR3, ANO5, EPN3, SLC7A4, ANKFN1, GAL, SLC7A8, CDH17, HCN4, COLEC12, SLC24A2, WNT11, SV2B, IGF2BP2, ATP6V1G3, RHBG, SNCA, MCOLN3, KCNJ10, KCNIP1, SPAG6, GCGR, KCNIP3, LGI3, FOXJ1, GPR143, GLRB, KCNS1, FABP7, CLNK, HCN2, TJP3 286 4761 19554 1.5653011135052723 8.31816269914909E-4 7.563175816408774E-5 7.433443921964488E-5

GOTERM_BP_ALL GO:0006812~monoatomic cation transport 33 9.939759036144578 3.071516632451634E-7 HCN4, SLC24A2, KCNG3, TMEM63C, CLCNKB, NALF2, SLC6A20, LRRC52, PANX2, ATP6V1G3, SLC38A3, TRPM6, SLC12A8, SLC38A4, SLC13A3, SLC15A1, MCOLN3, SLC36A2, KCNJ10, KCNIP1, SLC30A2, SLC6A15, KCNIP3, KCNK13, KCNK15, SLC9A2, SCNN1G, SLC9A3, SLC9A4, KCNS1, LCN2, ATP6V0D2, HCN2 286 808 19554 2.7923648134044177 0.0011993219703411162 9.997786638630068E-5 9.82629362665152E-5

GOTERM_CC_ALL GO:0016020~membrane 195 58.734939759036145 3.331589707736085E-7 HPSE2, PLEKHB1, AQP6, AQP4, SLC4A3, LIPH, SMCO3, CKMT1B, PTGDS, SLC12A8, TMEM52B, SLC6A15, KCNK13, KCNK15, UNC93A, PSG9, PLPP4, PSG4, ATP6V0D2, PTGES, CFAP61, HACD1, TMEM63C, MTTP, IL20RA, CLCNKB, SLC6A20, HSD11B1, HSD11B2, INPP5J, PROM1, TRPM6, B4GALNT2, SPTBN2, ST14, AFAP1L2, TMEM86A, ST8SIA2, L1CAM, SNAP91, FA2H, CPEB1, GJB3, LYPD6B, FNDC10, LRRN1, XKR4, HRK, GABRB2, PIGR, TENM3, DIRAS1, GRIK5, HEPACAM2, TFCP2L1, SLC7A10, CHRDL1, GPRC6A, M1AP, EPB41L4B, PANX2, UCHL1, NMRK2, DNER, KIF1A, SLC2A12, TMEM40, SYN3, ANO5, F5, SLC7A4, VSNL1, SLC7A8, RGS7BP, POPDC3, FAXC, PTGER3, TMPRSS2, CST6, C10ORF90, SCEL, CTNNA2, ATP6V1G3, RHBG, BIK, DMRT2, ATRNL1, GCGR, CKMT1A, MOCOS, TMEM61, GPR143, KCNS1, TJP3, RAB3B, KCNG3, DGKB, SERTM2, NALF2, RIMS2, ADORA1, ENPP1, SLC16A7, ENPP3, MALRD1, IL13RA2, SH3GL2, KRT6A, RGS7, CHRNB4, SLC15A1, DGAT2, SLC30A2, DIO1, WNT5A, VWDE, PRLR, SLC9A2, SCNN1G, SLC9A3, C1ORF116, SLC9A4, EMCN, DOK7, CLIP4, ADTRP, PKP3, RHCG, VTCN1, KNG1, LRRC52, VTN, CYP11A1, PODXL, UGT3A2, DRD1, SLC38A3, CD300LG, SLC38A4, SYT3, IGSF11, BFSP2, UST, CNTN3, REEP6, MUC20, DPP10, DOCK3, ST6GALNAC2, PSCA, DUSP15, GRM1, UPK1B, LRRTM1, CA4, CA9, ERC2, PLA2G4F, SLC13A3, CR2, SLC36A2, CR1, SYNGR3, SSTR5, EPN3, ADGRF1, NAT8L, ST6GALNAC3, CDH17, ST6GALNAC5, COLEC12, SLC24A2, HCN4, CAMK2B, DAPL1, ITLN1, SEMA3E, SV2B, KLRG2, MAN1C1, ZPLD1, NCAM2, SNCA, COL26A1, MCOLN3, KCNJ10, KCNIP1, PDE6G, KCNIP3, SYT14, MYO16, NFASC, GLRB, TEK, HCN2 299 10552 20791 1.285002637043874 1.4857788691624574E-4 3.389207415574482E-5 3.115639104003447E-5

GOTERM_CC_ALL GO:0016323~basolateral plasma membrane 18 5.421686746987952 3.7995598829310334E-7 ST14, SLC13A3, KCNJ10, MTTP, AQP4, SLC9A4, SLC7A8, ADORA1, SLC16A7, ENPP1, CA9, TEK, RHCG, SLC38A3, RHBG, CD300LG, CDH17, MUC20 299 269 20791 4.652907461053574 1.6944604538982055E-4 3.389207415574482E-5 3.115639104003447E-5

GOTERM_BP_ALL GO:0098655~monoatomic cation transmembrane transport 29 8.734939759036145 5.313145347201257E-7 HCN4, SLC24A2, KCNG3, TMEM63C, NALF2, SLC6A20, LRRC52, ATP6V1G3, TRPM6, SLC12A8, SLC38A4, SLC13A3, SLC15A1, MCOLN3, SLC36A2, KCNJ10, KCNIP1, SLC30A2, SLC6A15, KCNIP3, KCNK13, KCNK15, SLC9A2, SCNN1G, SLC9A3, SLC9A4, KCNS1, ATP6V0D2, HCN2 286 665 19554 2.981576318418423 0.002073693359404105 1.5963958250898546E-4 1.569012691377356E-4

GOTERM_BP_ALL GO:0065008~regulation of biological quality 78 23.49397590361446 6.451273724269969E-7 RAB3B, GABRB2, KCNG3, DGKB, GRIK5, AQP6, AQP4, SLC4A3, CHRDL1, GRM1, RIMS2, SCPEP1, ARHGAP40, LRRTM1, ADORA1, KIF1A, ERC2, SH3GL2, SLC12A8, CHRNB4, DGAT2, WNT5A, DIO1, KCNK13, KCNK15, PRLR, SSTR5, F5, SLC9A2, SLC9A3, SCNN1G, ALDH1A3, SLC9A4, GAL, SLC7A8, VSNL1, RGS7BP, ADTRP, ATP6V0D2, KANK4, HCN4, SLC24A2, CAMK2B, PFKFB2, POPDC3, CALCA, CCL11, KLK1, CRACD, PTGER3, SEMA3E, KNG1, C10ORF90, VTN, HSD11B2, ADH4, PCSK1N, SCIN, CYP11A1, DRD1, IGF2BP2, CTNNA2, SPTBN2, SNCA, KCNJ10, ST8SIA2, GCGR, L1CAM, NR0B2, BMP5, IGSF11, GLRB, KCNS1, LRRN1, TEK, XKR4, HCN2, TJP3 286 3072 19554 1.7359730113636362 0.0025173396301131357 1.7999053690713213E-4 1.7690314162480293E-4

GOTERM_CC_ALL GO:0016324~apical plasma membrane 22 6.626506024096386 7.767346111947481E-7 SLC15A1, AQP6, SLC6A20, SLC9A2, SCNN1G, SLC9A3, GPR143, SLC9A4, UPK1B, SLC7A8, PODXL, CA4, TEK, ENPP3, RHCG, PROM1, SLC38A3, TRPM6, CD300LG, ATP6V0D2, MUC20, SPTBN2 299 419 20791 3.6510085328182247 3.4636377334218427E-4 5.7737272765476275E-5 5.3076865098307787E-5

GOTERM_MF_ALL GO:0015081~sodium ion transmembrane transporter activity 14 4.216867469879518 1.7555193365886447E-6 HCN4, SLC24A2, SLC13A3, MCOLN3, SLC6A15, GRIK5, SLC6A20, SLC9A2, SCNN1G, SLC9A3, SLC9A4, SLC38A3, HCN2, SLC38A4 289 169 19144 5.487520730533773 0.0014700449191988874 1.338982475816248E-4 1.2719535556919543E-4

GOTERM_CC_ALL GO:0009925~basal plasma membrane 18 5.421686746987952 2.0786604272544794E-6 ST14, SLC13A3, KCNJ10, MTTP, AQP4, SLC9A4, SLC7A8, ADORA1, SLC16A7, ENPP1, CA9, TEK, RHCG, SLC38A3, RHBG, CD300LG, CDH17, MUC20 299 304 20791 4.117210878366485 9.26653904940955E-4 1.31134475385645E-4 1.2054962983882164E-4

GOTERM_CC_ALL GO:0045177~apical part of cell 23 6.927710843373494 2.352187899294081E-6 SLC15A1, AQP6, SLC6A20, SLC9A2, SCNN1G, EPB41L4B, SLC9A3, GPR143, SLC9A4, UPK1B, SLC7A8, PODXL, CA4, TEK, ENPP3, RHCG, PROM1, SLC38A3, TRPM6, CD300LG, ATP6V0D2, MUC20, SPTBN2 299 487 20791 3.2839993681882804 0.0010485269479859616 1.31134475385645E-4 1.2054962983882164E-4

GOTERM_MF_ALL GO:0015291~secondary active transmembrane transporter activity 18 5.421686746987952 3.1084223882746492E-6 SLC24A2, SLC15A1, SLC13A3, SLC36A2, SLC30A2, SLC6A15, SLC2A12, SLC4A3, SLC6A20, SLC9A2, SLC9A3, SLC9A4, SLC7A8, SLC16A7, SLC38A3, CDH17, SLC12A8, SLC38A4 289 299 19144 3.987825624052493 0.0026014723007088403 2.1733053198020257E-4 2.064510536212413E-4

GOTERM_MF_ALL GO:0005267~potassium channel activity 12 3.614457831325301 3.782047448866296E-6 HCN4, KCNG3, MCOLN3, KCNJ10, KCNIP1, KCNS1, KCNIP3, GRIK5, KCNK13, KCNK15, HCN2, LRRC52 289 128 19144 6.210207612456747 0.003164344630301019 2.44087523815294E-4 2.3186860128818752E-4

GOTERM_BP_ALL GO:0030855~epithelial cell differentiation 27 8.132530120481928 4.24364675331757E-6 EHF, ONECUT2, KRT23, TFCP2L1, SCEL, UPK1B, WNT11, MCIDAS, PODXL, PROM1, KRT6A, ST14, MCOLN3, WNT5A, FOXJ1, FOXN1, BMP7, PRLR, BMP5, SLC9A2, FA2H, SLC9A4, BFSP2, RHCG, CCNO, TJP3, CES1 286 657 19554 2.8097518919436726 0.016443271944838522 0.001105045614563895 0.0010860906590657432

GOTERM_CC_ALL GO:0045178~basal part of cell 18 5.421686746987952 4.670078009760086E-6 ST14, SLC13A3, KCNJ10, MTTP, AQP4, SLC9A4, SLC7A8, ADORA1, SLC16A7, ENPP1, CA9, TEK, RHCG, SLC38A3, RHBG, CD300LG, CDH17, MUC20 299 323 20791 3.8750220031684566 0.002080692008966878 2.3142831026144424E-4 2.127479982224039E-4

GOTERM_BP_ALL GO:0030001~metal ion transport 27 8.132530120481928 5.611492855764842E-6 HCN4, SLC24A2, KCNG3, CLCNKB, NALF2, SLC6A20, LRRC52, SLC38A3, TRPM6, SLC12A8, SLC38A4, SLC13A3, MCOLN3, KCNJ10, KCNIP1, SLC30A2, SLC6A15, KCNIP3, KCNK13, KCNK15, SLC9A2, SCNN1G, SLC9A3, SLC9A4, KCNS1, LCN2, HCN2 286 667 19554 2.7676266761724033 0.02168557640807489 0.0013093477109487202 0.0012868883416108903

GOTERM_BP_ALL GO:0006814~sodium ion transport 14 4.216867469879518 5.69864595138972E-6 HCN4, SLC24A2, SLC13A3, MCOLN3, SLC6A15, CLCNKB, SLC6A20, SLC9A2, SCNN1G, SLC9A3, SLC9A4, SLC38A3, HCN2, SLC38A4 286 194 19554 4.933962944272222 0.022018644633246653 0.0013093477109487202 0.0012868883416108903

GOTERM_MF_ALL GO:0005216~monoatomic ion channel activity 22 6.626506024096386 5.936266477380612E-6 HCN4, SLC24A2, GABRB2, KCNG3, MCOLN3, CHRNB4, KCNJ10, KCNIP1, TMEM63C, KCNIP3, GRIK5, KCNK13, CLCNKB, KCNK15, NALF2, ANO5, LRRC52, SCNN1G, KCNS1, GLRB, TRPM6, HCN2 289 455 19144 3.2029202631278757 0.004962253212587475 3.5575196960873815E-4 3.379431701765963E-4

GOTERM_BP_ALL GO:0098657~import into cell 28 8.433734939759036 7.6155544589044475E-6 COLEC12, HCN4, SLC24A2, CALCA, NALF2, SLC6A20, GRM1, LRRTM1, DNER, ADORA1, SLC38A3, SLC12A8, SH3GL2, SNCA, SLC38A4, SLC15A1, KCNJ10, AHSG, SNAP91, EPN3, SLC9A2, SCNN1G, SLC9A3, SLC9A4, PLPP4, SLC7A8, XKR4, HCN2 286 721 19554 2.655170072645801 0.029315789573057915 0.001652575317582265 0.0016242285315407874

GOTERM_CC_ALL GO:0031528~microvillus membrane 7 2.108433734939759 7.909892331618659E-6 SLC7A8, PODXL, MTTP, CA9, PROM1, MUC20, SLC38A4 299 34 20791 14.316053511705684 0.0035216104655326763 3.527811979901922E-4 3.24305585596365E-4

GOTERM_BP_ALL GO:0035725~sodium ion transmembrane transport 12 3.614457831325301 1.0656017414301234E-5 HCN4, SLC9A2, SLC24A2, SCNN1G, SLC9A3, SLC13A3, SLC9A4, MCOLN3, SLC6A15, SLC6A20, HCN2, SLC38A4 286 147 19554 5.581275866990153 0.04077852000244386 0.0021906528431716115 0.0021530763607106543

GOTERM_MF_ALL GO:0022836~gated channel activity 18 5.421686746987952 1.1038484194255618E-5 HCN4, GABRB2, KCNG3, MCOLN3, CHRNB4, KCNJ10, TMEM63C, GRIK5, KCNK13, CLCNKB, KCNK15, NALF2, ANO5, LRRC52, SCNN1G, KCNS1, GLRB, HCN2 289 329 19144 3.6241941081814453 0.009207648394207402 6.174192159320308E-4 5.865114601881151E-4

GOTERM_BP_ALL GO:0003333~amino acid transmembrane transport 10 3.0120481927710845 1.3256039387149908E-5 SLC7A4, SLC36A2, KCNJ10, SLC7A8, SLC6A15, SLC6A20, SLC7A10, SLC38A3, GRM1, SLC38A4 286 98 19554 6.976594833737692 0.0504733570673398 0.0025889044923103773 0.002544496760363425

GOTERM_BP_ALL GO:0032501~multicellular organismal process 130 39.1566265060241 1.689183963018378E-5 DGKB, AQP6, AQP4, SLC4A3, FGF9, PPP4R4, ADORA1, ENPP1, SLC16A7, ENPP3, PRSS3, PTGDS, SH3GL2, KRT6A, CCBE1, CHIA, CHRNB4, DGAT2, IGFBP5, WNT5A, VWDE, PRLR, PSG9, SCNN1G, SLC9A4, HOXB9, PLPP4, EMCN, PSG4, PADI2, ADTRP, PKP3, TAGLN3, PTGES, CALCA, KLK1, TMEM63C, CRACD, MTTP, CLCNKB, SLC6A20, KNG1, HSD11B1, VTN, HSD11B2, PCSK1N, SCIN, PODXL, DRD1, PROM1, SLC38A3, WNT2, SPTBN2, ST14, ST8SIA2, L1CAM, FOXN1, BMP7, BMP5, FA2H, BFSP2, GJB3, CNTN3, REEP6, GABRB2, PIGR, TENM3, ONECUT2, GRIK5, WFIKKN2, TFCP2L1, CHRDL1, GRM1, SCPEP1, UCHL1, CRTAC1, LRRTM1, DNER, ANKRD2, SLC13A3, CR2, AHSG, SLC2A12, FOS, SYNGR3, F5, ALDH1A3, ANKFN1, GAL, ADGRF1, SLC7A8, ANGPTL7, CDH17, HCN4, CAMK2B, SLC24A2, POPDC3, CCL11, PTGER3, NDNF, SEMA3E, HOXD10, SCEL, WNT11, NRAP, NCAM2, IGF2BP2, CTNNA2, SNCA, MCOLN3, KCNJ10, PDE6G, IRX6, SPAG6, BIK, DMRT2, GCGR, EYA4, FOXJ1, MYO16, GPR143, NFASC, GLRB, SMOC1, FABP7, TLL2, CLNK, TEK, NOTUM, TJP3 286 6520 19554 1.363218070273285 0.06386630376010916 0.0031418821712141834 0.0030879891590607396

GOTERM_MF_ALL GO:0005261~monoatomic cation channel activity 18 5.421686746987952 2.5315763301251152E-5 HCN4, SLC24A2, KCNG3, MCOLN3, CHRNB4, KCNJ10, KCNIP1, TMEM63C, KCNIP3, GRIK5, KCNK13, KCNK15, NALF2, LRRC52, SCNN1G, KCNS1, TRPM6, HCN2 289 351 19144 3.397036642711383 0.02099142561744416 0.0013274953381093573 0.001261041459443573

GOTERM_BP_ALL GO:0098719~sodium ion import across plasma membrane 6 1.8072289156626504 2.6564274307259824E-5 HCN4, SLC9A2, SCNN1G, SLC9A3, SLC9A4, HCN2 286 25 19554 16.40895104895105 0.09858362098536055 0.004535089926599016 0.004457299085564163

GOTERM_BP_ALL GO:0043266~regulation of potassium ion transport 10 3.0120481927710845 2.9026433221959906E-5 DPP10, GAL, KCNIP1, KCNS1, KCNIP3, ADORA1, DRD1, UNC93A, LRRC52, RGS7 286 108 19554 6.330613830613831 0.10721360006607872 0.004535089926599016 0.004457299085564163

GOTERM_BP_ALL GO:0098659~inorganic cation import across plasma membrane 10 3.0120481927710845 2.9026433221959906E-5 HCN4, SLC9A2, SLC24A2, SCNN1G, SLC9A3, SLC9A4, KCNJ10, NALF2, SLC12A8, HCN2 286 108 19554 6.330613830613831 0.10721360006607872 0.004535089926599016 0.004457299085564163

GOTERM_BP_ALL GO:0099587~inorganic ion import across plasma membrane 10 3.0120481927710845 2.9026433221959906E-5 HCN4, SLC9A2, SLC24A2, SCNN1G, SLC9A3, SLC9A4, KCNJ10, NALF2, SLC12A8, HCN2 286 108 19554 6.330613830613831 0.10721360006607872 0.004535089926599016 0.004457299085564163

GOTERM_CC_ALL GO:0005576~extracellular region 100 30.120481927710845 3.232070642944542E-5 RAB3B, HPSE2, AQP4, ISM2, RIMS2, FGF9, BPIFA2, LIPH, ENPP1, ENPP3, IL13RA2, PRSS3, PTGDS, KRT6A, CCBE1, CHIA, TMEM52B, IGFBP5, WNT5A, VWDE, PRLR, PSG9, SLC9A3, SCNN1G, C1ORF116, EMCN, SLPI, PSG4, PADI2, RHCG, ATP6V0D2, CALCA, KLK1, KNG1, VTN, PCSK1N, SCIN, PODXL, ARSI, PROM1, WNT2, SPTBN2, ST14, ST8SIA2, BMP7, BMP5, LYPD6B, LCN2, LRRN1, CNTN3, MUC20, GABRB2, PIGR, PSCA, WFIKKN2, CHRDL1, SERPINA4, SCPEP1, UPK1B, CRTAC1, LRRTM1, CA4, SLC13A3, CR2, CR1, SLC36A2, AHSG, F5, EPN3, ALDH1A3, GAL, ADGRF1, ANGPTL7, COLEC12, CCL11, ITLN1, TMPRSS2, NDNF, SEMA3E, CST6, CST5, CPN1, SCEL, WNT11, TTR, IGFBPL1, MAN1C1, ZPLD1, SNCA, COL26A1, SPAG6, LGI3, KLK15, PSAT1, GLB1, SMOC1, TLL2, TEK, NOTUM, CPVL 299 4781 20791 1.4544052929691735 0.014311865503141452 0.0013104577334120597 0.001204680876006602

GOTERM_BP_ALL GO:1905039~carboxylic acid transmembrane transport 11 3.313253012048193 3.328368119332449E-5 SLC13A3, SLC36A2, KCNJ10, SLC7A8, SLC6A15, SLC16A7, SLC6A20, SLC7A10, SLC38A3, GRM1, SLC38A4 286 137 19554 5.489612577203818 0.12194101504773824 0.005000233028504825 0.004914463542352797

GOTERM_BP_ALL GO:0015807~L-amino acid transport 9 2.710843373493976 3.66294702713619E-5 SLC36A2, KCNJ10, SLC7A8, SLC6A15, SLC6A20, SLC7A10, SLC38A3, GRM1, SLC38A4 286 86 19554 7.155065864368191 0.13334470203604676 0.005299063365923689 0.005208168013768828

GOTERM_MF_ALL GO:0022853~active monoatomic ion transmembrane transporter activity 16 4.819277108433735 3.887874278532117E-5 SLC24A2, SLC15A1, SLC13A3, SLC36A2, SLC30A2, SLC6A15, SLC4A3, SLC6A20, SLC9A2, SLC9A3, SLC9A4, SLC38A3, ATP6V1G3, ATP6V0D2, SLC12A8, SLC38A4 289 292 19144 3.629710385362848 0.03205597598923027 0.0019187803056990858 0.0018227269411706453

GOTERM_MF_ALL GO:0015175~neutral L-amino acid transmembrane transporter activity 7 2.108433734939759 4.201606110481885E-5 SLC36A2, SLC7A8, SLC6A15, SLC6A20, SLC7A10, SLC38A3, SLC38A4 289 43 19144 10.783616319304741 0.034597531615943256 0.0019584152926079455 0.0018603778166967012

GOTERM_BP_ALL GO:1903825~organic acid transmembrane transport 11 3.313253012048193 4.259067702126079E-5 SLC13A3, SLC36A2, KCNJ10, SLC7A8, SLC6A15, SLC16A7, SLC6A20, SLC7A10, SLC38A3, GRM1, SLC38A4 286 141 19554 5.333878887070377 0.15329702000954293 0.00594139944446588 0.005839486038736435

GOTERM_BP_ALL GO:0048856~anatomical structure development 111 33.433734939759034 5.6958609624106864E-5 EHF, AQP6, MCIDAS, FGF9, PPP4R4, ADORA1, ENPP1, SH3GL2, KRT6A, CCBE1, DGAT2, IGFBP5, WNT5A, VWDE, PRLR, PSG9, SLC9A2, SLC9A4, HOXB9, PLPP4, EMCN, PADI2, ADTRP, TAGLN3, RHCG, CCNO, CES1, CFAP61, CALCA, HACD1, HSD11B1, FBXO40, VTN, KLC3, SCIN, PODXL, SOHLH2, DRD1, PROM1, WNT2, B4GALNT2, SPTBN2, ST14, ST8SIA2, L1CAM, FOXN1, BMP7, NR0B2, KY, BMP5, FA2H, BFSP2, GJB3, CNTN3, XKR4, GABRB2, TENM3, ONECUT2, BNC1, WFIKKN2, KRT23, TFCP2L1, CHRDL1, UCHL1, UPK1B, CRTAC1, DNER, CA9, ANKRD2, CR2, AHSG, SLC2A12, FOS, SYNGR3, ALDH1A3, ADGRF1, ANGPTL7, CDH17, HCN4, CAMK2B, POPDC3, CCL11, NDNF, SEMA3E, HOXD10, CST6, SCEL, WNT11, NRAP, NCAM2, IGF2BP2, CTNNA2, MCOLN3, KCNJ10, IRX6, SPAG6, BIK, DMRT2, EYA4, MYBPH, FOXJ1, MYO16, NFASC, GLRB, SMOC1, FABP7, ASXL3, TLL2, TEK, NOTUM, TJP3 286 5463 19554 1.3891890646421123 0.1995199155548918 0.0076717354893710826 0.007540141460239526

GOTERM_CC_ALL GO:0099503~secretory vesicle 34 10.240963855421686 6.168511546679936E-5 RAB3B, PIGR, CALCA, KLK1, SERPINA4, KNG1, PCSK1N, TTR, SV2B, BPIFA2, CA4, KIF1A, PRSS3, ATP6V1G3, SNCA, SYT3, CHRNB4, CR1, AHSG, SLC30A2, SPAG6, LGI3, KLK15, SYN3, SYNGR3, SNAP91, F5, NFASC, SLC9A4, GAL, SLPI, GLB1, LCN2, PADI2 299 1111 20791 2.127987380677867 0.02713739080481714 0.002292630124849376 0.002107574778448978

GOTERM_BP_ALL GO:0042391~regulation of membrane potential 20 6.024096385542169 7.965220955089389E-5 HCN4, GABRB2, POPDC3, KCNG3, CHRNB4, KCNJ10, GRIK5, KCNK13, KCNK15, SLC4A3, GRM1, RIMS2, IGSF11, KCNS1, GLRB, ADORA1, RGS7BP, DRD1, HCN2, SNCA 286 477 19554 2.866693055372301 0.2674425984748222 0.010272607723947424 0.010096400679015403

GOTERM_BP_ALL GO:0009888~tissue development 49 14.759036144578314 8.152863272974147E-5 EHF, ONECUT2, BNC1, KRT23, TFCP2L1, UPK1B, MCIDAS, FGF9, DNER, ENPP1, CA9, KRT6A, CR2, DGAT2, IGFBP5, WNT5A, FOS, PRLR, SLC9A2, ALDH1A3, SLC9A4, RHCG, CCNO, CES1, HCN4, POPDC3, HACD1, CCL11, SEMA3E, HOXD10, CST6, VTN, SCEL, WNT11, PODXL, NRAP, PROM1, WNT2, B4GALNT2, ST14, MCOLN3, DMRT2, FOXJ1, FOXN1, BMP7, BMP5, FA2H, BFSP2, TJP3 286 1875 19554 1.7867524475524474 0.27279390415942983 0.010272607723947424 0.010096400679015403

GOTERM_MF_ALL GO:0015293~symporter activity 11 3.313253012048193 8.339382740538136E-5 SLC24A2, SLC13A3, SLC15A1, SLC36A2, SLC6A15, SLC2A12, SLC16A7, SLC6A20, SLC38A3, SLC12A8, SLC38A4 289 148 19144 4.923407836902646 0.06750075906573028 0.003682495852269208 0.0034981516022152075

GOTERM_BP_ALL GO:0032502~developmental process 118 35.54216867469879 1.0565228376070878E-4 EHF, AQP6, RIMS2, MCIDAS, FGF9, PPP4R4, ADORA1, ENPP1, SH3GL2, KRT6A, CCBE1, DGAT2, IGFBP5, WNT5A, VWDE, PRLR, PSG9, SLC9A2, SLC9A4, HOXB9, PLPP4, EMCN, PADI2, ADTRP, TAGLN3, RHCG, CCNO, CES1, CFAP61, CALCA, HACD1, HSD11B1, FBXO40, VTN, KLC3, SCIN, PODXL, SOHLH2, DRD1, PROM1, WNT2, B4GALNT2, RBM11, SPTBN2, SYT3, ST14, ST8SIA2, L1CAM, FOXN1, BMP7, NR0B2, KY, BMP5, FA2H, BFSP2, GJB3, CNTN3, XKR4, GABRB2, TENM3, ONECUT2, BNC1, WFIKKN2, KRT23, TFCP2L1, CHRDL1, M1AP, UCHL1, UPK1B, CRTAC1, DNER, CA9, ANKRD2, CR2, AHSG, SLC2A12, FOS, SYNGR3, ALDH1A3, ADGRF1, ANGPTL7, ELF5, CDH17, HCN4, CAMK2B, POPDC3, CCL11, DAPL1, NDNF, SEMA3E, HOXD10, CST6, SCEL, WNT11, NRAP, NCAM2, IGF2BP2, CTNNA2, ATP6V1G3, MCOLN3, KCNJ10, IRX6, SPAG6, BIK, DMRT2, EYA4, MYBPH, FOXJ1, MYO16, NFASC, GLRB, SMOC1, FABP7, ASXL3, TLL2, TEK, NOTUM, TJP3 286 5983 19554 1.3484429660261184 0.3382088657130139 0.012896181886541515 0.01267497241741753

GOTERM_MF_ALL GO:0022804~active transmembrane transporter activity 20 6.024096385542169 1.0844338218875196E-4 SLC24A2, SLC15A1, SLC13A3, SLC36A2, SLC30A2, SLC6A15, SLC2A12, SLC4A3, SLC6A20, SLC9A2, SLC9A3, SLC9A4, SLC7A8, SLC16A7, SLC38A3, ATP6V1G3, ATP6V0D2, CDH17, SLC12A8, SLC38A4 289 474 19144 2.7950301490663283 0.08687316058041183 0.004549199882818145 0.004321468780221766

GOTERM_CC_ALL GO:0034705~potassium channel complex 9 2.710843373493976 1.1082889246551413E-4 HCN4, DPP10, KCNG3, KCNIP1, KCNS1, KCNIP3, GRIK5, HCN2, LRRC52 299 102 20791 6.135451505016722 0.04823052855779797 0.003761035484717767 0.0034574541451441354

GOTERM_BP_ALL GO:0032328~alanine transport 5 1.5060240963855422 1.1507950419273051E-4 SLC36A2, SLC7A8, SLC7A10, SLC38A3, SLC38A4 286 18 19554 18.99184149184149 0.3621432149236622 0.01362122858717592 0.013387582321087648

GOTERM_CC_ALL GO:0005615~extracellular space 82 24.69879518072289 1.1805940983419E-4 RAB3B, GABRB2, PIGR, PSCA, HPSE2, WFIKKN2, CHRDL1, SERPINA4, RIMS2, SCPEP1, UPK1B, FGF9, BPIFA2, CRTAC1, LIPH, LRRTM1, CA4, ENPP1, ENPP3, IL13RA2, PRSS3, PTGDS, KRT6A, CCBE1, CHIA, SLC13A3, CR2, CR1, SLC36A2, IGFBP5, TMEM52B, AHSG, WNT5A, F5, EPN3, SLC9A3, SCNN1G, ALDH1A3, C1ORF116, GAL, SLPI, ANGPTL7, PADI2, RHCG, ATP6V0D2, COLEC12, CALCA, CCL11, KLK1, ITLN1, TMPRSS2, NDNF, SEMA3E, CST6, CST5, KNG1, CPN1, VTN, SCEL, PCSK1N, WNT11, SCIN, TTR, IGFBPL1, PODXL, MAN1C1, ZPLD1, PROM1, WNT2, SPTBN2, SNCA, ST14, LGI3, BMP7, BMP5, PSAT1, GLB1, SMOC1, LCN2, TLL2, LRRN1, CPVL 299 3831 20791 1.4883528057066582 0.05129521154498107 0.003761035484717767 0.0034574541451441354

GOTERM_BP_ALL GO:0015711~organic anion transport 18 5.421686746987952 1.2416296456097746E-4 SLC13A3, PLA2G4F, CR1, SLC36A2, KCNJ10, SLC6A15, MTTP, SLC4A3, SLC7A10, SLC6A20, NR0B2, GRM1, SLC7A8, FABP7, CA4, SLC16A7, SLC38A3, SLC38A4 286 412 19554 2.9870663317265262 0.3843858065839474 0.014264133516916997 0.014019459439693896

GOTERM_CC_ALL GO:0034702~monoatomic ion channel complex 17 5.120481927710843 1.2983863343976636E-4 HCN4, DPP10, GABRB2, KCNG3, CHRNB4, KCNJ10, KCNIP1, KCNIP3, GRIK5, KCNK13, CLCNKB, KCNK15, LRRC52, SCNN1G, KCNS1, GLRB, HCN2 299 380 20791 3.110781552543566 0.05626680970863929 0.0038605353676090534 0.003548922647353614

GOTERM_BP_ALL GO:0015804~neutral amino acid transport 7 2.108433734939759 1.3056272313285863E-4 SLC36A2, SLC7A8, SLC6A15, SLC6A20, SLC7A10, SLC38A3, SLC38A4 286 54 19554 8.862859362859362 0.3995896031057574 0.014283098966193147 0.014038099572763823

GOTERM_BP_ALL GO:0060429~epithelium development 34 10.240963855421686 1.3164146512620412E-4 EHF, CCL11, ONECUT2, KRT23, SEMA3E, TFCP2L1, SCEL, UPK1B, WNT11, MCIDAS, PODXL, CA9, PROM1, WNT2, KRT6A, ST14, MCOLN3, IGFBP5, DMRT2, WNT5A, FOXJ1, FOXN1, BMP7, PRLR, BMP5, SLC9A2, ALDH1A3, FA2H, SLC9A4, BFSP2, RHCG, CCNO, TJP3, CES1 286 1138 19554 2.042707731635675 0.40211512503701097 0.014283098966193147 0.014038099572763823

GOTERM_BP_ALL GO:0006865~amino acid transport 10 3.0120481927710845 1.4719978630248968E-4 SLC7A4, SLC36A2, KCNJ10, SLC7A8, SLC6A15, SLC6A20, SLC7A10, SLC38A3, GRM1, SLC38A4 286 133 19554 5.140648824859351 0.43738059801868545 0.015539523386419587 0.015272972422033997

GOTERM_MF_ALL GO:0015171~amino acid transmembrane transporter activity 9 2.710843373493976 1.5372918936044786E-4 SLC7A4, SLC36A2, SLC7A8, GRIK5, SLC6A15, SLC6A20, SLC7A10, SLC38A3, SLC38A4 289 102 19144 5.844901282312232 0.12088095994714954 0.006141847136829321 0.005834388758108425

GOTERM_BP_ALL GO:0048878~chemical homeostasis 30 9.036144578313253 1.7081804937214484E-4 SLC24A2, CALCA, CCL11, MTTP, AQP6, AQP4, SLC4A3, GRM1, SV2B, ADORA1, ENPP1, MALRD1, ENPP3, ERC2, SLC38A3, RHBG, SLC12A8, DGAT2, KCNJ10, IGFBP5, SLC30A2, GCGR, WNT5A, SLC9A2, SCNN1G, SLC9A3, SLC9A4, RHCG, ATP6V0D2, CES1 286 959 19554 2.1388100950144744 0.4869813738502796 0.017558297390726257 0.017257118198412212

GOTERM_BP_ALL GO:0042592~homeostatic process 41 12.349397590361445 1.867732273266246E-4 SLC24A2, CALCA, CCL11, CRACD, MTTP, AQP6, AQP4, SLC4A3, GRM1, PCSK1N, SV2B, ADORA1, ENPP1, DRD1, IGF2BP2, MALRD1, ENPP3, PROM1, ERC2, ATP6V1G3, SLC38A3, RHBG, SLC12A8, SH3GL2, DGAT2, KCNJ10, IGFBP5, SLC30A2, GCGR, WNT5A, FOXN1, SLC9A2, SCNN1G, SLC9A3, SLC9A4, EMCN, PKP3, RHCG, ATP6V0D2, TJP3, CES1 286 1517 19554 1.8478548478548478 0.5179903546399964 0.018532981933458138 0.018215083881860158

GOTERM_BP_ALL GO:0051049~regulation of transport 45 13.55421686746988 1.8978988155103057E-4 PFKFB2, DPP10, RAB3B, CAMK2B, CALCA, GRIK5, PTGER3, ITLN1, LRRC52, RIMS2, VTN, SCIN, SV2B, LRRTM1, ADORA1, ENPP1, STAP1, DRD1, ERC2, IL13RA2, SLC38A3, SNCA, RGS7, SYT3, CHRNB4, KCNJ10, KCNIP1, AHSG, KCNIP3, LGI3, WNT5A, UNC93A, SYNGR3, NR0B2, SNAP91, SSTR5, GPR143, GAL, PLPP4, KCNS1, VSNL1, ADTRP, REEP6, PTGES, CES1 286 1728 19554 1.7804851398601398 0.5236390632376144 0.018532981933458138 0.018215083881860158

GOTERM_CC_ALL GO:1902495~transmembrane transporter complex 19 5.72289156626506 1.9166176567434818E-4 HCN4, DPP10, GABRB2, KCNG3, CHRNB4, KCNJ10, KCNIP1, KCNIP3, GRIK5, KCNK13, CLCNKB, KCNK15, LRRC52, SCNN1G, KCNS1, GLRB, ATP6V1G3, ATP6V0D2, HCN2 299 476 20791 2.7755613951266125 0.08193707069635314 0.0053425717181724555 0.004911332745405172

GOTERM_BP_ALL GO:0089718~amino acid import across plasma membrane 6 1.8072289156626504 2.1496811655149982E-4 KCNJ10, SLC7A8, SLC6A20, SLC38A3, GRM1, SLC38A4 286 38 19554 10.795362532204638 0.568276835065685 0.020479645445125812 0.0201283560839319

GOTERM_BP_ALL GO:0007626~locomotory behavior 12 3.614457831325301 2.5959170271246676E-4 ALDH1A3, UCHL1, MCOLN3, CHRNB4, ANKFN1, KCNJ10, GLRB, LRRTM1, DRD1, HOXD10, GRM1, SNCA 286 209 19554 3.9255863753471414 0.6373642700985362 0.02414202835225941 0.023727917778884756

GOTERM_BP_ALL GO:0003013~circulatory system process 20 6.024096385542169 2.693742776086657E-4 HCN4, SLC13A3, CALCA, KLK1, GCGR, SLC4A3, SLC6A20, KNG1, F5, SCNN1G, SCPEP1, HSD11B2, SLC7A8, ADORA1, SLC16A7, TEK, DRD1, SLC38A3, SH3GL2, TJP3 286 525 19554 2.604595404595405 0.6509663974110375 0.024469207635801123 0.024049484924178317

GOTERM_BP_ALL GO:0003018~vascular process in circulatory system 14 4.216867469879518 2.795752714004284E-4 SLC13A3, CALCA, SLC4A3, SLC6A20, KNG1, SCPEP1, SLC7A8, ADORA1, SLC16A7, TEK, DRD1, SLC38A3, SH3GL2, TJP3 286 283 19554 3.3822926190417357 0.6646073365766336 0.024818659320228937 0.024392942429687377

GOTERM_BP_ALL GO:0003008~system process 52 15.66265060240964 3.27147559270479E-4 GABRB2, PIGR, GRIK5, AQP6, AQP4, SLC4A3, GRM1, SCPEP1, UCHL1, ADORA1, SLC16A7, ANKRD2, SH3GL2, CCBE1, SLC13A3, CHRNB4, FOS, F5, SCNN1G, ALDH1A3, SLC9A4, ANKFN1, ADGRF1, SLC7A8, PTGES, HCN4, SLC24A2, CALCA, CCL11, KLK1, CRACD, TMEM63C, PTGER3, CLCNKB, SLC6A20, HOXD10, KNG1, HSD11B2, DRD1, CTNNA2, SLC38A3, SNCA, KCNJ10, PDE6G, GCGR, EYA4, GPR143, BFSP2, GLRB, REEP6, TEK, TJP3 286 2154 19554 1.6505444416307926 0.7215104197235229 0.028396408144677577 0.027909321778652643

GOTERM_CC_ALL GO:0008076~voltage-gated potassium channel complex 8 2.4096385542168677 3.354399092355845E-4 HCN4, DPP10, KCNG3, KCNIP1, KCNS1, KCNIP3, HCN2, LRRC52 299 91 20791 6.112977323679664 0.13897461951761703 0.008800364677592393 0.008090021340387625

GOTERM_CC_ALL GO:0031982~vesicle 88 26.506024096385545 3.700477616559419E-4 RAB3B, GABRB2, PIGR, PSCA, AQP6, AQP4, SERPINA4, RIMS2, SCPEP1, UPK1B, BPIFA2, CRTAC1, DNER, CA4, MALRD1, KIF1A, ENPP3, PRSS3, PTGDS, SH3GL2, KRT6A, SLC13A3, PLA2G4F, CR2, CHRNB4, CR1, SLC36A2, TMEM52B, AHSG, SLC30A2, WNT5A, SYN3, SYNGR3, ANO5, PRLR, F5, EPN3, SLC9A3, SCNN1G, ALDH1A3, SLC9A4, C1ORF116, GAL, ADGRF1, SLPI, PADI2, RHCG, ATP6V0D2, COLEC12, CAMK2B, CALCA, KLK1, MTTP, ITLN1, TMPRSS2, CST6, CST5, KNG1, VTN, SCEL, PCSK1N, SCIN, TTR, SV2B, PODXL, MAN1C1, ZPLD1, PROM1, ATP6V1G3, CD300LG, SNCA, SYT3, MCOLN3, SPAG6, LGI3, ST8SIA2, GCGR, KLK15, BMP7, SNAP91, BMP5, GPR143, NFASC, PSAT1, GLB1, LCN2, REEP6, CPVL 299 4334 20791 1.4118805493777908 0.15216721133894284 0.008944620637846573 0.008222633321787208

GOTERM_CC_ALL GO:1990351~transporter complex 19 5.72289156626506 3.810488612535536E-4 HCN4, DPP10, GABRB2, KCNG3, CHRNB4, KCNJ10, KCNIP1, KCNIP3, GRIK5, KCNK13, CLCNKB, KCNK15, LRRC52, SCNN1G, KCNS1, GLRB, ATP6V1G3, ATP6V0D2, HCN2 299 504 20791 2.6213635398418007 0.15631846142423633 0.008944620637846573 0.008222633321787208

GOTERM_MF_ALL GO:0140828~metal cation:monoatomic cation antiporter activity 6 1.8072289156626504 4.486697481985216E-4 SLC9A2, SLC24A2, SLC9A3, SLC9A4, SLC30A2, SLC38A3 289 43 19144 9.243099702261206 0.3134454671191208 0.017110632669934525 0.016254081332464622

GOTERM_BP_ALL GO:0015808~L-alanine transport 4 1.2048192771084338 4.635488940638827E-4 SLC36A2, SLC7A8, SLC38A3, SLC38A4 286 11 19554 24.862047043865225 0.8365920464199268 0.03878961169944897 0.03812424969641184

GOTERM_BP_ALL GO:1902475~L-alpha-amino acid transmembrane transport 7 2.108433734939759 4.667464797424736E-4 KCNJ10, SLC7A8, SLC6A20, SLC7A10, SLC38A3, GRM1, SLC38A4 286 68 19554 7.0381530234471406 0.8386217359042794 0.03878961169944897 0.03812424969641184

GOTERM_BP_ALL GO:0046942~carboxylic acid transport 14 4.216867469879518 5.517648584698999E-4 SLC13A3, PLA2G4F, SLC36A2, KCNJ10, SLC6A15, SLC7A10, SLC6A20, NR0B2, GRM1, SLC7A8, FABP7, SLC16A7, SLC38A3, SLC38A4 286 304 19554 3.1486474052263524 0.8842520449528699 0.04489986535798811 0.04412969357637388

GOTERM_BP_ALL GO:0015849~organic acid transport 14 4.216867469879518 5.707786848799346E-4 SLC13A3, PLA2G4F, SLC36A2, KCNJ10, SLC6A15, SLC7A10, SLC6A20, NR0B2, GRM1, SLC7A8, FABP7, SLC16A7, SLC38A3, SLC38A4 286 305 19554 3.1383239711108564 0.8925434814196928 0.04549921516614336 0.044718762678654475

GOTERM_BP_ALL GO:0050804~modulation of chemical synaptic transmission 19 5.72289156626506 6.142102444859122E-4 SLC24A2, CAMK2B, CHRNB4, KCNJ10, DGKB, GRIK5, WNT5A, SYN3, SLC7A10, CHRDL1, GRM1, RIMS2, IGSF11, SV2B, LRRTM1, ADORA1, DRD1, ERC2, SNCA 286 517 19554 2.5126536906034005 0.9093234675276826 0.047982104299239464 0.047159062571628343

GOTERM_BP_ALL GO:0099177~regulation of trans-synaptic signaling 19 5.72289156626506 6.313429618090818E-4 SLC24A2, CAMK2B, CHRNB4, KCNJ10, DGKB, GRIK5, WNT5A, SYN3, SLC7A10, CHRDL1, GRM1, RIMS2, IGSF11, SV2B, LRRTM1, ADORA1, DRD1, ERC2, SNCA 286 518 19554 2.507803007803008 0.915197981459828 0.04835344331031909 0.047524031968334615

GOTERM_CC_ALL GO:0005902~microvillus 8 2.4096385542168677 6.313563946922072E-4 SLC7A8, PODXL, MTTP, CA9, TEK, PROM1, MUC20, SLC38A4 299 101 20791 5.507732044107421 0.24548029138816596 0.014079247601636221 0.012942806091190247

GOTERM_MF_ALL GO:0015276~ligand-gated monoatomic ion channel activity 11 3.313253012048193 6.575312324067171E-4 HCN4, GABRB2, SCNN1G, MCOLN3, CHRNB4, KCNJ10, GLRB, TMEM63C, GRIK5, ANO5, HCN2 289 191 19144 3.8149966484900086 0.42373773788146163 0.022991025144595775 0.021840103742840084

GOTERM_MF_ALL GO:0051139~metal cation:proton antiporter activity 5 1.5060240963855422 6.70044636623406E-4 SLC9A2, SLC9A3, SLC9A4, SLC30A2, SLC38A3 289 27 19144 12.267076765346662 0.42975295725608664 0.022991025144595775 0.021840103742840084

GOTERM_MF_ALL GO:0022834~ligand-gated channel activity 11 3.313253012048193 6.850722629498145E-4 HCN4, GABRB2, SCNN1G, MCOLN3, CHRNB4, KCNJ10, GLRB, TMEM63C, GRIK5, ANO5, HCN2 289 192 19144 3.7951268742791235 0.4368939445287354 0.022991025144595775 0.021840103742840084

GOTERM_BP_ALL GO:0010463~mesenchymal cell proliferation 5 1.5060240963855422 6.865894520258335E-4 WNT11, FGF9, WNT5A, WNT2, BMP7 286 28 19554 12.20904095904096 0.9316713618322562 0.051573430761786644 0.05068878666013798

GOTERM_BP_ALL GO:0046903~secretion 20 6.024096385542169 7.208785204300945E-4 RAB3B, SYT3, PLA2G4F, GCGR, LGI3, MTTP, SYN3, SYNGR3, PRLR, RIMS2, SLC9A4, SCIN, GAL, ADORA1, CLNK, CA9, PTGDS, ERC2, SLC38A3, SNCA 286 569 19554 2.4031855666302064 0.9402441410167035 0.05312738680754621 0.052216087545870433

GOTERM_CC_ALL GO:0031253~cell projection membrane 15 4.518072289156627 8.280549130292867E-4 PLA2G4F, PDE6G, MTTP, ITLN1, SLC9A3, SLC7A8, PODXL, CA4, ADORA1, CA9, DRD1, PROM1, TRPM6, MUC20, SLC38A4 299 364 20791 2.865458120474843 0.3088963601737442 0.0175838555617569 0.016164530897579216

GOTERM_CC_ALL GO:0030141~secretory granule 27 8.132530120481928 8.67365072553031E-4 PIGR, CALCA, KLK1, SERPINA4, KNG1, PCSK1N, TTR, SV2B, BPIFA2, CA4, KIF1A, PRSS3, SNCA, CHRNB4, CR1, AHSG, SLC30A2, SPAG6, KLK15, F5, NFASC, SLC9A4, GAL, SLPI, GLB1, LCN2, PADI2 299 926 20791 2.02748181483274 0.3209175208349624 0.0175838555617569 0.016164530897579216

GOTERM_MF_ALL GO:0005416~amino acid:monoatomic cation symporter activity 5 1.5060240963855422 8.856299212966586E-4 SLC36A2, SLC6A15, SLC6A20, SLC38A3, SLC38A4 289 29 19144 11.421071471184824 0.5240710529614723 0.02857859630645756 0.027147963356670652

GOTERM_BP_ALL GO:0042634~regulation of hair cycle 5 1.5060240963855422 8.981953356178298E-4 FA2H, GAL, WNT5A, PKP3, FOXN1 286 30 19554 11.395104895104897 0.9701277736899004 0.06496946260968968 0.06385503506364534

GOTERM_MF_ALL GO:0005249~voltage-gated potassium channel activity 8 2.4096385542168677 9.385850723412728E-4 HCN4, KCNG3, KCNJ10, KCNS1, KCNK13, KCNK15, HCN2, LRRC52 289 103 19144 5.145026371485201 0.5447477583644018 0.02916566206275288 0.027705640839110902

GOTERM_BP_ALL GO:0015816~glycine transport 4 1.2048192771084338 9.899905252903503E-4 SLC36A2, SLC7A8, SLC6A20, SLC7A10 286 14 19554 19.534465534465536 0.9791376428152851 0.07030732712334742 0.06910133866526645

GOTERM_BP_ALL GO:0048513~animal organ development 65 19.57831325301205 0.0010476571537556168 GABRB2, TENM3, ONECUT2, AQP6, TFCP2L1, CHRDL1, UCHL1, FGF9, DNER, ENPP1, ANKRD2, KRT6A, CCBE1, DGAT2, IGFBP5, WNT5A, VWDE, FOS, SYNGR3, PRLR, ALDH1A3, HOXB9, ANGPTL7, PADI2, CDH17, HCN4, POPDC3, HACD1, CCL11, NDNF, SEMA3E, HOXD10, HSD11B1, VTN, SCEL, WNT11, PODXL, NRAP, DRD1, CTNNA2, PROM1, WNT2, B4GALNT2, SPTBN2, ST14, MCOLN3, BIK, EYA4, FOXJ1, L1CAM, FOXN1, BMP7, NR0B2, KY, MYO16, BMP5, FA2H, NFASC, BFSP2, GJB3, ASXL3, SMOC1, CNTN3, NOTUM, TEK 286 3019 19554 1.4720407118552201 0.983349951201893 0.07307408647445426 0.07182063952263952

GOTERM_BP_ALL GO:0050808~synapse organization 15 4.518072289156627 0.0010695723497523377 GABRB2, WNT5A, SEMA3E, L1CAM, CHRDL1, KY, ADGRF1, GLRB, LRRTM1, DNER, DOK7, DRD1, ERC2, SPTBN2, SNCA 286 368 19554 2.7868463058680453 0.9847176321001713 0.0732938525988181 0.07203663597717938

GOTERM_BP_ALL GO:0060078~regulation of postsynaptic membrane potential 8 2.4096385542168677 0.0010908589009005461 GABRB2, CHRNB4, GLRB, GRIK5, ADORA1, RGS7BP, GRM1, SNCA 286 109 19554 5.018027843715918 0.9859384622817673 0.07346370460202642 0.07220357449236546

GOTERM_BP_ALL GO:0006629~lipid metabolic process 34 10.240963855421686 0.001141282641649856 HACD1, DGKB, MTTP, PLCXD2, HSD11B1, SCPEP1, HSD11B2, ADH4, STK33, CYP11A1, LIPH, ADORA1, INPP5J, HAO1, PTGDS, B4GALNT2, SNCA, PLA2G4F, TMEM86A, DGAT2, ST8SIA2, PLAAT5, NR0B2, PRLR, ALDH1A3, FA2H, PLPP4, GLB1, THRSP, ADTRP, ST6GALNAC3, ST6GALNAC5, PTGES, CES1 286 1287 19554 1.8062170929303796 0.9884554025332708 0.07555677963193792 0.07426074680158978

GOTERM_CC_ALL GO:0042995~cell projection 56 16.867469879518072 0.0012046359401370003 GABRB2, TENM3, AQP4, GRM1, RIMS2, UCHL1, LRRTM1, DNER, CA4, ADORA1, CA9, NEFM, KIF1A, ERC2, SH3GL2, RGS7, PLA2G4F, CHRNB4, SSTR5, SLC9A3, SLC7A8, CLIP4, RGS7BP, PVALB, HCN4, CAMK2B, CFAP61, CALCA, MTTP, ITLN1, KLC3, SCIN, SV2B, PODXL, INPP5J, DRD1, NCAM2, CTNNA2, PROM1, TRPM6, SLC38A4, SPTBN2, SNCA, KCNJ10, KCNIP1, PDE6G, SPAG6, LGI3, L1CAM, NFASC, CPEB1, GLRB, CNTN3, TEK, MUC20, HCN2 299 2548 20791 1.528244330919916 0.4158465041730176 0.023359462143526178 0.021473945019833483

GOTERM_BP_ALL GO:0070634~transepithelial ammonium transport 3 0.9036144578313252 0.0012457999177901305 SLC9A4, RHCG, RHBG 286 4 19554 51.27797202797203 0.9923295091172053 0.08110157464813748 0.07971043140660518

GOTERM_BP_ALL GO:0030154~cell differentiation 78 23.49397590361446 0.0013781264253614454 GABRB2, EHF, TENM3, ONECUT2, BNC1, WFIKKN2, KRT23, TFCP2L1, CHRDL1, M1AP, RIMS2, UCHL1, UPK1B, MCIDAS, FGF9, CRTAC1, DNER, ENPP1, SH3GL2, KRT6A, CR2, IGFBP5, WNT5A, FOS, PRLR, SLC9A2, SLC9A4, ADGRF1, ELF5, RHCG, CDH17, CCNO, CES1, CAMK2B, CFAP61, POPDC3, DAPL1, NDNF, SEMA3E, HOXD10, FBXO40, VTN, SCEL, KLC3, WNT11, PODXL, SOHLH2, NRAP, DRD1, NCAM2, CTNNA2, PROM1, WNT2, B4GALNT2, RBM11, SYT3, ST14, MCOLN3, KCNJ10, IRX6, SPAG6, ST8SIA2, EYA4, MYBPH, FOXJ1, L1CAM, FOXN1, BMP7, MYO16, BMP5, FA2H, NFASC, BFSP2, SMOC1, TLL2, CNTN3, TEK, TJP3 286 3833 19554 1.3913146597727863 0.9954291381658297 0.08824527569609517 0.08673159585184573

GOTERM_BP_ALL GO:0048869~cellular developmental process 78 23.49397590361446 0.0014094310636695077 GABRB2, EHF, TENM3, ONECUT2, BNC1, WFIKKN2, KRT23, TFCP2L1, CHRDL1, M1AP, RIMS2, UCHL1, UPK1B, MCIDAS, FGF9, CRTAC1, DNER, ENPP1, SH3GL2, KRT6A, CR2, IGFBP5, WNT5A, FOS, PRLR, SLC9A2, SLC9A4, ADGRF1, ELF5, RHCG, CDH17, CCNO, CES1, CAMK2B, CFAP61, POPDC3, DAPL1, NDNF, SEMA3E, HOXD10, FBXO40, VTN, SCEL, KLC3, WNT11, PODXL, SOHLH2, NRAP, DRD1, NCAM2, CTNNA2, PROM1, WNT2, B4GALNT2, RBM11, SYT3, ST14, MCOLN3, KCNJ10, IRX6, SPAG6, ST8SIA2, EYA4, MYBPH, FOXJ1, L1CAM, FOXN1, BMP7, MYO16, BMP5, FA2H, NFASC, BFSP2, SMOC1, TLL2, CNTN3, TEK, TJP3 286 3836 19554 1.3902265617594083 0.9959560422914798 0.08879415701117899 0.08727106215205226

GOTERM_CC_ALL GO:0043005~neuron projection 36 10.843373493975903 0.0014355067915354921 HCN4, CAMK2B, GABRB2, CALCA, TENM3, GRM1, UCHL1, KLC3, SV2B, LRRTM1, DNER, ADORA1, INPP5J, NEFM, NCAM2, DRD1, CTNNA2, KIF1A, PROM1, ERC2, SNCA, RGS7, CHRNB4, KCNIP1, PDE6G, LGI3, L1CAM, SSTR5, NFASC, CPEB1, GLRB, CLIP4, CNTN3, RGS7BP, HCN2, PVALB 299 1432 20791 1.7480895349488985 0.47307444425061707 0.026676501209367893 0.024523241022064657

GOTERM_MF_ALL GO:0022858~alanine transmembrane transporter activity 4 1.2048192771084338 0.001631815377676925 SLC36A2, SLC7A8, SLC38A3, SLC38A4 289 16 19144 16.560553633217992 0.7455315002984324 0.047588333018239115 0.0452060803522486

GOTERM_BP_ALL GO:0065007~biological regulation 217 65.36144578313254 0.001641211302890448 EHF, HPSE2, PLEKHB1, AQP6, AQP4, SLC4A3, C10ORF71, MCIDAS, PPP4R4, PTGDS, SLC12A8, CCBE1, CHIA, KCNK13, KCNK15, UNC93A, PSG9, HOXB9, PLPP4, SLPI, PSG4, PADI2, TAGLN3, ATP6V0D2, CCNO, CES1, PTGES, HACD1, KLK1, CRACD, MTTP, IL20RA, ADH4, HSD11B2, INPP5J, PROM1, B4GALNT2, SPTBN2, RBM11, AFAP1L2, ST8SIA2, L1CAM, NR0B2, SNAP91, FA2H, CPEB1, PPP1R1A, GJB3, LRRN1, XKR4, HRK, GABRB2, PIGR, TENM3, DIRAS1, GRIK5, WFIKKN2, TFCP2L1, SLC7A10, CHRDL1, SERPINA4, GPRC6A, EPB41L4B, PANX2, UCHL1, NMRK2, DNER, KIF1A, ANKRD2, AHSG, FOS, SYN3, DUSP9, F5, ANKFN1, GAL, VSNL1, SLC7A8, ANGPTL7, RGS7BP, POPDC3, CCL11, PTGER3, TMPRSS2, HOXD10, CST5, C10ORF90, SCEL, CTNNA2, BIK, DMRT2, ATRNL1, GCGR, MYBPH, G0S2, GPR143, KCNS1, SMOC1, FABP7, NOTUM, TJP3, RAB3B, KCNG3, DGKB, RIMS2, ARHGAP40, FGF9, ADORA1, ENPP1, ENPP3, MALRD1, IL13RA2, SH3GL2, KRT6A, RGS7, CHRNB4, DGAT2, IGFBP5, DIO1, WNT5A, VWDE, PRLR, SLC9A2, SCNN1G, SLC9A3, PCP4, SLC9A4, EMCN, DOK7, CLIP4, UCA1, ADTRP, PKP3, RHCG, PVALB, KANK4, PFKFB2, CALCA, VTCN1, PLCXD2, KNG1, LRRC52, VTN, PCSK1N, STK33, SCIN, CYP11A1, PODXL, SOHLH2, STAP1, DRD1, SLC38A3, WNT2, SYT3, BMP7, FOXN1, BMP5, IGSF11, UST, LCN2, REEP6, MUC20, DPP10, DOCK3, ONECUT2, PSCA, BNC1, DUSP15, GRM1, SCPEP1, LRRTM1, ZNF385B, ERC2, TRIM67, CR2, CR1, SYNGR3, SSTR5, ALDH1A3, ADGRF1, ESRP1, ELF5, CDH17, COLEC12, SLC24A2, HCN4, CAMK2B, DAPL1, ITLN1, NDNF, SEMA3E, TTR, IGFBPL1, WNT11, SV2B, IGF2BP2, MAPK4, SNCA, COL26A1, MCOLN3, KCNJ10, KCNIP1, PDE6G, IRX6, KCNIP3, LGI3, EYA4, FOXJ1, TPD52L1, MYO16, GLRB, ASXL3, CLNK, TLL2, THRSP, TEK, HCN2 286 13239 19554 1.1206606672276285 0.9983672347393168 0.10175510077920778 0.10000968558407032

GOTERM_MF_ALL GO:0015459~potassium channel regulator activity 6 1.8072289156626504 0.0016448887455648802 DPP10, KCNIP1, KCNS1, KCNIP3, UNC93A, LRRC52 289 57 19144 6.972864687670734 0.7483086351171485 0.047588333018239115 0.0452060803522486

GOTERM_CC_ALL GO:0031410~cytoplasmic vesicle 57 17.16867469879518 0.00170038546060376 RAB3B, GABRB2, PIGR, AQP6, AQP4, SERPINA4, BPIFA2, DNER, CA4, MALRD1, KIF1A, PRSS3, SH3GL2, CHRNB4, CR1, SLC36A2, AHSG, SLC30A2, WNT5A, SYN3, SYNGR3, PRLR, F5, EPN3, SLC9A3, SLC9A4, GAL, ADGRF1, SLPI, PADI2, RHCG, ATP6V0D2, COLEC12, CAMK2B, CALCA, KLK1, KNG1, PCSK1N, TTR, SV2B, ZPLD1, ATP6V1G3, CD300LG, SNCA, SYT3, MCOLN3, SPAG6, LGI3, ST8SIA2, GCGR, KLK15, SNAP91, GPR143, NFASC, GLB1, LCN2, REEP6 299 2646 20791 1.4979220227667431 0.5318738225881499 0.03033487661717108 0.027886321553901663

GOTERM_BP_ALL GO:0055082~intracellular chemical homeostasis 20 6.024096385542169 0.0017202412614753387 SLC24A2, CALCA, DGAT2, CCL11, SLC30A2, WNT5A, AQP4, SLC4A3, GRM1, SLC9A2, SCNN1G, SLC9A3, SLC9A4, SV2B, ADORA1, ENPP1, RHCG, ERC2, SLC38A3, ATP6V0D2 286 613 19554 2.230689375876978 0.9988016025854167 0.10442771670880786 0.10263645787125278

GOTERM_BP_ALL GO:0141091~transforming growth factor beta receptor superfamily signaling pathway 10 3.0120481927710845 0.0017377884244937304 PSG9, ONECUT2, FGF9, WNT5A, WFIKKN2, VWDE, FOS, CHRDL1, BMP7, BMP5 286 187 19554 3.65618338880371 0.9988811407163981 0.10442771670880786 0.10263645787125278

GOTERM_BP_ALL GO:0051480~regulation of cytosolic calcium ion concentration 6 1.8072289156626504 0.0018026939505032637 CALCA, SV2B, WNT5A, ADORA1, ERC2, GRM1 286 60 19554 6.837062937062938 0.99913214091761 0.1066867056161477 0.10485669812093984

GOTERM_CC_ALL GO:0097708~intracellular vesicle 57 17.16867469879518 0.0018206167163183034 RAB3B, GABRB2, PIGR, AQP6, AQP4, SERPINA4, BPIFA2, DNER, CA4, MALRD1, KIF1A, PRSS3, SH3GL2, CHRNB4, CR1, SLC36A2, AHSG, SLC30A2, WNT5A, SYN3, SYNGR3, PRLR, F5, EPN3, SLC9A3, SLC9A4, GAL, ADGRF1, SLPI, PADI2, RHCG, ATP6V0D2, COLEC12, CAMK2B, CALCA, KLK1, KNG1, PCSK1N, TTR, SV2B, ZPLD1, ATP6V1G3, CD300LG, SNCA, SYT3, MCOLN3, SPAG6, LGI3, ST8SIA2, GCGR, KLK15, SNAP91, GPR143, NFASC, GLB1, LCN2, REEP6 299 2654 20791 1.4934068094351178 0.5563570114106162 0.031230579056844742 0.02870972514194248

GOTERM_MF_ALL GO:0015385~sodium:proton antiporter activity 4 1.2048192771084338 0.0019595706543217737 SLC9A2, SLC9A3, SLC9A4, SLC38A3 289 17 19144 15.58640341949929 0.8067423948937358 0.05324162258416652 0.05057636853346927

GOTERM_MF_ALL GO:0015294~solute:monoatomic cation symporter activity 8 2.4096385542168677 0.0019672113231336856 SLC13A3, SLC15A1, SLC36A2, SLC6A15, SLC6A20, SLC38A3, SLC12A8, SLC38A4 289 117 19144 4.5293821902818445 0.8079782659383282 0.05324162258416652 0.05057636853346927

GOTERM_BP_ALL GO:1901379~regulation of potassium ion transmembrane transport 7 2.108433734939759 0.0020347307221241103 DPP10, GAL, KCNIP1, KCNS1, KCNIP3, LRRC52, RGS7 286 90 19554 5.317715617715618 0.9996500749821848 0.11845308272081646 0.11642124540840101

GOTERM_BP_ALL GO:0048167~regulation of synaptic plasticity 11 3.313253012048193 0.002088401536484538 CAMK2B, RIMS2, IGSF11, SLC24A2, KCNJ10, LRRTM1, ADORA1, DRD1, ERC2, CHRDL1, SNCA 286 230 19554 3.2698996655518395 0.9997163917631153 0.11845308272081646 0.11642124540840101

GOTERM_BP_ALL GO:0055080~monoatomic cation homeostasis 18 5.421686746987952 0.002092489172487541 SLC24A2, CALCA, CCL11, KCNJ10, SLC30A2, WNT5A, SLC4A3, GRM1, SLC9A2, SCNN1G, SLC9A3, SLC9A4, SV2B, ADORA1, RHCG, ERC2, ATP6V0D2, SLC12A8 286 530 19554 2.322021374851564 0.9997208944601784 0.11845308272081646 0.11642124540840101

GOTERM_MF_ALL GO:0005509~calcium ion binding 23 6.927710843373494 0.0021479306834088566 CCBE1, SYT3, PLA2G4F, KCNIP1, DGKB, KCNIP3, ITLN1, VWDE, PCP4, SCIN, VSNL1, CRTAC1, SMOC1, DNER, MAN1C1, PADI2, ENPP1, TLL2, ENPP3, PRSS3, CDH17, PVALB, SNCA 289 746 19144 2.0423202872065085 0.835015176200004 0.056316057605625955 0.053496898583651836

GOTERM_CC_ALL GO:0012505~endomembrane system 95 28.614457831325304 0.0021529406420869807 RAB3B, PIGR, EHF, KCNG3, ST6GALNAC2, GRIK5, HEPACAM2, AQP4, CHRDL1, SERPINA4, PANX2, UCHL1, BPIFA2, LRRTM1, DNER, CA4, MALRD1, KIF1A, PRSS3, PTGDS, SH3GL2, RGS7, CHRNB4, DGAT2, CR1, SLC36A2, IGFBP5, AHSG, SLC30A2, WNT5A, DIO1, SLC2A12, FOS, SYN3, SYNGR3, ANO5, PRLR, F5, EPN3, SLC9A3, SLC9A4, GAL, SLPI, PADI2, ADTRP, NAT8L, ST6GALNAC3, ATP6V0D2, ST6GALNAC5, CES1, PTGES, CAMK2B, CALCA, HACD1, KLK1, MTTP, PTGER3, KNG1, HSD11B1, VTN, HSD11B2, PCSK1N, TTR, SV2B, MAN1C1, UGT3A2, ARSI, DRD1, PROM1, ATP6V1G3, B4GALNT2, CD300LG, SNCA, COL26A1, SYT3, TMEM86A, MCOLN3, ATRNL1, SPAG6, BIK, LGI3, ST8SIA2, GCGR, KCNIP3, KLK15, SNAP91, FA2H, GPR143, NFASC, GLB1, UST, LCN2, NOTUM, REEP6, MUC20 299 5024 20791 1.3148559156849797 0.6175841506261364 0.035563389865584945 0.0326928023428023

GOTERM_BP_ALL GO:0019725~cellular homeostasis 22 6.626506024096386 0.0023140456077682565 SLC24A2, CALCA, DGAT2, CCL11, SLC30A2, WNT5A, AQP4, SLC4A3, GRM1, SLC9A2, SCNN1G, SLC9A3, SLC9A4, SV2B, ADORA1, ENPP1, RHCG, ERC2, SLC38A3, ATP6V1G3, ATP6V0D2, SLC12A8 286 727 19554 2.068987408739816 0.9998827794049752 0.12824365392353473 0.12604387798577826

GOTERM_BP_ALL GO:0014070~response to organic cyclic compound 25 7.530120481927711 0.0023505522788836103 COLEC12, HCN4, GABRB2, CPN1, HSD11B2, ADORA1, UGT3A2, ENPP1, CA9, DRD1, PTGDS, PLA2G4F, IGFBP5, ST8SIA2, FOS, BMP7, NR0B2, SSTR5, SCNN1G, GLB1, PADI2, ADTRP, TEK, HCN2, CES1 286 876 19554 1.9512165916275503 0.9998983950163121 0.12824365392353473 0.12604387798577826

GOTERM_BP_ALL GO:0034330~cell junction organization 19 5.72289156626506 0.0023639383211711473 GABRB2, WNT5A, SEMA3E, L1CAM, CHRDL1, KY, ADGRF1, WNT11, GLRB, LRRTM1, DNER, DOK7, PKP3, DRD1, ERC2, CDH17, TJP3, SPTBN2, SNCA 286 583 19554 2.2282023294030155 0.9999035842331021 0.12824365392353473 0.12604387798577826

GOTERM_BP_ALL GO:0048731~system development 75 22.590361445783135 0.0024438664209153077 GABRB2, TENM3, ONECUT2, WFIKKN2, TFCP2L1, CHRDL1, UCHL1, FGF9, CRTAC1, DNER, ADORA1, SH3GL2, CCBE1, CR2, IGFBP5, AHSG, WNT5A, SLC2A12, VWDE, FOS, SYNGR3, PRLR, PSG9, ALDH1A3, HOXB9, ADGRF1, EMCN, ANGPTL7, PADI2, TAGLN3, ADTRP, CDH17, HCN4, CAMK2B, POPDC3, CALCA, NDNF, SEMA3E, HOXD10, HSD11B1, VTN, WNT11, SCIN, PODXL, NRAP, DRD1, IGF2BP2, NCAM2, CTNNA2, PROM1, WNT2, SPTBN2, ST14, MCOLN3, KCNJ10, DMRT2, IRX6, SPAG6, BIK, ST8SIA2, FOXJ1, L1CAM, FOXN1, BMP7, MYO16, BMP5, FA2H, NFASC, BFSP2, GLRB, SMOC1, FABP7, CNTN3, NOTUM, TEK 286 3728 19554 1.375482082295387 0.9999294982055954 0.13076359233007112 0.12852059164238172

GOTERM_BP_ALL GO:0051580~regulation of neurotransmitter uptake 4 1.2048192771084338 0.002497185629882954 RAB3B, DRD1, SYNGR3, SNCA 286 19 19554 14.393816709606183 0.9999427856876768 0.13181090635571374 0.12954994098811703

GOTERM_BP_ALL GO:0050801~monoatomic ion homeostasis 18 5.421686746987952 0.0025881357794563346 SLC24A2, CALCA, CCL11, KCNJ10, SLC30A2, WNT5A, SLC4A3, GRM1, SLC9A2, SCNN1G, SLC9A3, SLC9A4, SV2B, ADORA1, RHCG, ERC2, ATP6V0D2, SLC12A8 286 541 19554 2.2748083709266704 0.999959932880716 0.1347901113940859 0.13247804343110492

GOTERM_BP_ALL GO:0015695~organic cation transport 7 2.108433734939759 0.002674200710767023 SLC36A2, SLC7A8, SLC6A15, SLC6A20, SLC7A10, SLC38A3, SLC38A4 286 95 19554 5.037835848362165 0.9999713998127847 0.137439841792842 0.13508232274519213

GOTERM_MF_ALL GO:0022821~solute:potassium antiporter activity 4 1.2048192771084338 0.002731018830181652 SLC9A2, SLC24A2, SLC9A3, SLC9A4 289 19 19144 13.94572937534147 0.8989080295049245 0.06943408480370929 0.06595824265620535

GOTERM_BP_ALL GO:0007178~cell surface receptor protein serine/threonine kinase signaling pathway 10 3.0120481927710845 0.0027390413671583933 PSG9, ONECUT2, FGF9, WNT5A, WFIKKN2, VWDE, FOS, CHRDL1, BMP7, BMP5 286 200 19554 3.418531468531469 0.9999778155125002 0.13894409844312577 0.13656077673403988

GOTERM_BP_ALL GO:0002064~epithelial cell development 10 3.0120481927710845 0.0028215099956099892 ST14, SLC9A4, BFSP2, ONECUT2, PODXL, WNT5A, FOXJ1, TFCP2L1, TJP3, BMP5 286 201 19554 3.4015238492850433 0.9999839406692513 0.141292539010931 0.13886893427111216

GOTERM_CC_ALL GO:0030659~cytoplasmic vesicle membrane 32 9.63855421686747 0.0029006366907289843 COLEC12, RAB3B, CAMK2B, GABRB2, PIGR, AQP6, AQP4, SV2B, CA4, ZPLD1, MALRD1, KIF1A, ATP6V1G3, CD300LG, SH3GL2, SNCA, SYT3, MCOLN3, CHRNB4, CR1, SLC36A2, SLC30A2, WNT5A, SYN3, SYNGR3, EPN3, SLC9A3, GPR143, NFASC, SLC9A4, REEP6, ATP6V0D2 299 1276 20791 1.7438273870057979 0.7262565304833062 0.04620299871661168 0.04247360868567441

GOTERM_BP_ALL GO:0098771~inorganic ion homeostasis 16 4.819277108433735 0.0030712638155260654 SLC24A2, CALCA, CCL11, KCNJ10, SLC30A2, WNT5A, GRM1, SCNN1G, SV2B, ADORA1, ENPP1, ENPP3, RHCG, ERC2, RHBG, SLC12A8 286 457 19554 2.3937200654924946 0.999993964743772 0.15185261346132672 0.14924787073170334

GOTERM_CC_ALL GO:0030424~axon 21 6.325301204819277 0.0030924809776137997 HCN4, CALCA, TENM3, LGI3, L1CAM, NFASC, UCHL1, LRRTM1, CLIP4, ADORA1, INPP5J, NEFM, CNTN3, RGS7BP, KIF1A, NCAM2, CTNNA2, ERC2, HCN2, PVALB, SNCA 299 706 20791 2.0683250116062037 0.748769282751991 0.04756022469019843 0.04372128278695372

GOTERM_BP_ALL GO:0009987~cellular process 253 76.20481927710844 0.003116499776982423 EHF, HPSE2, PLEKHB1, AQP6, AQP4, SLC4A3, MCIDAS, LIPH, CKMT1B, PRSS3, PTGDS, SLC12A8, CCBE1, CHIA, SLC6A15, KCNK13, KCNK15, PSG9, HOXB9, PLPP4, PSG4, PADI2, TAGLN3, ATP6V0D2, CCNO, CES1, PTGES, CFAP61, HACD1, KLK1, CRACD, TMEM63C, MTTP, IL20RA, CLCNKB, SLC6A20, ADH4, HSD11B2, INPP5J, HAO1, PROM1, TRPM6, B4GALNT2, SPTBN2, RBM11, ST14, ST8SIA2, L1CAM, NR0B2, SNAP91, FA2H, CPEB1, PPP1R1A, GJB3, XKR4, HRK, GABRB2, PIGR, TENM3, DIRAS1, GRIK5, WFIKKN2, HEPACAM2, KRT23, TFCP2L1, SLC7A10, CHRDL1, GPRC6A, M1AP, EPB41L4B, PANX2, UCHL1, NMRK2, CRTAC1, DNER, NEFM, KIF1A, AHSG, SLC2A12, FOS, SYN3, PLAAT5, ANO5, DUSP9, SLC7A4, ANKFN1, GAL, SLC7A8, RGS7BP, POPDC3, CCL11, PNCK, PTGER3, TMPRSS2, HOXD10, CPN1, SCEL, NRAP, CTNNA2, ATP6V1G3, RHBG, BIK, ATRNL1, GCGR, CKMT1A, MOCOS, MYBPH, G0S2, GPR143, KCNS1, SMOC1, FABP7, NOTUM, TJP3, RAB3B, KCNG3, DGKB, NALF2, RIMS2, ARHGAP40, FGF9, ADORA1, ENPP1, SLC16A7, ENPP3, IL13RA2, GYG2, SH3GL2, KRT6A, RGS7, CHRNB4, SLC15A1, DGAT2, IGFBP5, SLC30A2, DIO1, WNT5A, VWDE, MAT1A, PRLR, SLC9A2, SCNN1G, SLC9A3, SLC9A4, EMCN, DOK7, CLIP4, ADTRP, PKP3, RHCG, PVALB, KANK4, PFKFB2, CALCA, GMPR, VTCN1, PLCXD2, KNG1, LRRC52, FBXO40, VTN, PCSK1N, KLC3, STK33, SCIN, CYP11A1, PODXL, SOHLH2, STAP1, UGT3A2, DRD1, SLC38A3, WNT2, SLC38A4, SYT3, BMP7, FOXN1, KY, BMP5, IGSF11, BFSP2, UST, LCN2, CNTN3, REEP6, MUC20, DPP10, DOCK3, ST6GALNAC2, ONECUT2, BNC1, DUSP15, GRM1, SCPEP1, UPK1B, LRRTM1, CA4, ZNF385B, CA9, ERC2, PLA2G4F, SLC13A3, CR2, SLC36A2, CR1, SYNGR3, SSTR5, EPN3, ALDH1A3, ADGRF1, ESRP1, ELF5, NAT8L, ST6GALNAC3, CDH17, ST6GALNAC5, COLEC12, SLC24A2, HCN4, CAMK2B, CCDC187, DAPL1, PPM1H, ITLN1, NDNF, SEMA3E, TTR, IGFBPL1, WNT11, SV2B, MAN1C1, NCAM2, MAPK4, SNCA, MCOLN3, KCNJ10, KCNIP1, IRX6, SPAG6, KCNIP3, LGI3, EYA4, FOXJ1, TPD52L1, MYO16, NFASC, GLRB, GLB1, PSAT1, CLNK, TLL2, TEK, HCN2 286 16108 19554 1.0738620083666024 0.9999949452271835 0.1521631016111668 0.149553033047944

GOTERM_BP_ALL GO:0006873~intracellular monoatomic ion homeostasis 16 4.819277108433735 0.0031711322075347833 SLC24A2, CALCA, CCL11, SLC30A2, WNT5A, SLC4A3, GRM1, SLC9A2, SCNN1G, SLC9A3, SLC9A4, SV2B, ADORA1, RHCG, ERC2, ATP6V0D2 286 459 19554 2.383289912701678 0.9999959195354488 0.15291904200778844 0.15029600672501275

GOTERM_MF_ALL GO:0001665~alpha-N-acetylgalactosaminide alpha-2,6-sialyltransferase activity 3 0.9036144578313252 0.0032506094062682055 ST6GALNAC2, ST6GALNAC3, ST6GALNAC5 289 6 19144 33.121107266435985 0.9346793883917909 0.07888564193959016 0.07493665867205407

GOTERM_CC_ALL GO:0120025~plasma membrane bounded cell projection 52 15.66265060240964 0.003276730861499272 GABRB2, TENM3, AQP4, GRM1, UCHL1, LRRTM1, DNER, CA4, ADORA1, CA9, NEFM, KIF1A, ERC2, RGS7, PLA2G4F, CHRNB4, SSTR5, SLC9A3, SLC7A8, CLIP4, RGS7BP, PVALB, HCN4, CAMK2B, CFAP61, CALCA, MTTP, ITLN1, KLC3, SV2B, PODXL, INPP5J, DRD1, NCAM2, CTNNA2, PROM1, TRPM6, SLC38A4, SNCA, KCNJ10, KCNIP1, PDE6G, SPAG6, LGI3, L1CAM, NFASC, CPEB1, GLRB, CNTN3, TEK, MUC20, HCN2 299 2428 20791 1.4892199699161952 0.7686495343210111 0.04871406547428918 0.04478198844049005

GOTERM_MF_ALL GO:0046943~carboxylic acid transmembrane transporter activity 10 3.0120481927710845 0.0032908193896134157 SLC13A3, SLC36A2, SLC7A8, GRIK5, SLC6A15, SLC16A7, SLC6A20, SLC7A10, SLC38A3, SLC38A4 289 199 19144 3.328754499139295 0.9368507423273036 0.07888564193959016 0.07493665867205407

GOTERM_BP_ALL GO:0051797~regulation of hair follicle development 4 1.2048192771084338 0.003354605235703403 GAL, WNT5A, PKP3, FOXN1 286 21 19554 13.022977022977024 0.999998012186793 0.15979375671533527 0.15705279877884593

GOTERM_MF_ALL GO:0005342~organic acid transmembrane transporter activity 10 3.0120481927710845 0.0034009656272004705 SLC13A3, SLC36A2, SLC7A8, GRIK5, SLC6A15, SLC16A7, SLC6A20, SLC7A10, SLC38A3, SLC38A4 289 200 19144 3.3121107266435987 0.9424365006293024 0.07926139336725542 0.07529360013552153

GOTERM_CC_ALL GO:0012506~vesicle membrane 32 9.63855421686747 0.003533000824645198 COLEC12, RAB3B, CAMK2B, GABRB2, PIGR, AQP6, AQP4, SV2B, CA4, ZPLD1, MALRD1, KIF1A, ATP6V1G3, CD300LG, SH3GL2, SNCA, SYT3, MCOLN3, CHRNB4, CR1, SLC36A2, SLC30A2, WNT5A, SYN3, SYNGR3, EPN3, SLC9A3, GPR143, NFASC, SLC9A4, REEP6, ATP6V0D2 299 1295 20791 1.7182422747640138 0.7937174500847044 0.05082962476747607 0.04672678510014617

GOTERM_MF_ALL GO:0008373~sialyltransferase activity 4 1.2048192771084338 0.003666198815800367 ST6GALNAC2, ST8SIA2, ST6GALNAC3, ST6GALNAC5 289 21 19144 12.617564672927996 0.9539450575987016 0.08313353530963535 0.07897190422142954

GOTERM_BP_ALL GO:0007275~multicellular organism development 81 24.397590361445783 0.003793162092244909 GABRB2, TENM3, ONECUT2, WFIKKN2, TFCP2L1, CHRDL1, UCHL1, FGF9, CRTAC1, PPP4R4, DNER, ADORA1, SH3GL2, CCBE1, CR2, IGFBP5, AHSG, WNT5A, SLC2A12, VWDE, FOS, SYNGR3, PRLR, PSG9, ALDH1A3, HOXB9, PLPP4, ADGRF1, EMCN, ANGPTL7, PADI2, TAGLN3, ADTRP, CDH17, HCN4, CAMK2B, POPDC3, CALCA, CCL11, NDNF, SEMA3E, HOXD10, HSD11B1, VTN, SCEL, WNT11, SCIN, PODXL, NRAP, DRD1, IGF2BP2, NCAM2, CTNNA2, PROM1, WNT2, SPTBN2, ST14, MCOLN3, KCNJ10, DMRT2, IRX6, SPAG6, BIK, ST8SIA2, FOXJ1, L1CAM, FOXN1, BMP7, MYO16, BMP5, FA2H, NFASC, BFSP2, GLRB, GJB3, SMOC1, FABP7, TLL2, CNTN3, NOTUM, TEK 286 4162 19554 1.3306153241280585 0.999999643903005 0.17850712207600739 0.17544517195335188

GOTERM_MF_ALL GO:0015179~L-amino acid transmembrane transporter activity 6 1.8072289156626504 0.0038158171914333934 SLC36A2, SLC7A8, SLC6A20, SLC7A10, SLC38A3, SLC38A4 289 69 19144 5.760192568075823 0.959391229106236 0.08424922693717413 0.08003174477822143

GOTERM_CC_ALL GO:0031012~extracellular matrix 19 5.72289156626506 0.003929136537768041 COLEC12, CCBE1, COL26A1, HPSE2, AHSG, WNT5A, NDNF, L1CAM, BMP7, KNG1, VTN, IGFBPL1, WNT11, ANGPTL7, SLPI, LRRTM1, SMOC1, LRRN1, WNT2 299 621 20791 2.1274834526252295 0.8272390859089993 0.053432874510482856 0.049119907061206204

GOTERM_CC_ALL GO:0030312~external encapsulating structure 19 5.72289156626506 0.003953553495170256 COLEC12, CCBE1, COL26A1, HPSE2, AHSG, WNT5A, NDNF, L1CAM, BMP7, KNG1, VTN, IGFBPL1, WNT11, ANGPTL7, SLPI, LRRTM1, SMOC1, LRRN1, WNT2 299 622 20791 2.1240630612222953 0.8291176026272877 0.053432874510482856 0.049119907061206204

GOTERM_BP_ALL GO:0051588~regulation of neurotransmitter transport 7 2.108433734939759 0.003995929722182486 RIMS2, RAB3B, CHRNB4, SV2B, DRD1, SYNGR3, SNCA 286 103 19554 4.646547627130151 0.999999839243203 0.18452158269636557 0.18135646594248525

GOTERM_BP_ALL GO:0006887~exocytosis 11 3.313253012048193 0.00401544662805711 RIMS2, RAB3B, SYT3, SCIN, GCGR, LGI3, CLNK, PTGDS, SYNGR3, ERC2, SNCA 286 252 19554 2.9844322344322345 0.9999998510912917 0.18452158269636557 0.18135646594248525

GOTERM_BP_ALL GO:0007267~cell-cell signaling 24 7.228915662650602 0.004105300696210618 HCN4, GABRB2, CHRNB4, CALCA, GRIK5, WNT5A, SYN3, CHRDL1, GRM1, RIMS2, PANX2, GAL, SV2B, FGF9, GLRB, GJB3, ADORA1, TEK, DRD1, ERC2, WNT2, HCN2, PVALB, SNCA 286 864 19554 1.8991841491841492 0.999999895326882 0.1864570292953334 0.1832587136366577

GOTERM_BP_ALL GO:0009719~response to endogenous stimulus 35 10.542168674698797 0.004596655426457064 HCN4, GABRB2, CALCA, DOCK3, ONECUT2, WFIKKN2, NDNF, CHRDL1, CPN1, HSD11B2, FGF9, CYP11A1, ENPP1, DRD1, PTGDS, WNT2, SH3GL2, IGFBP5, GCGR, WNT5A, FOS, BMP7, PRLR, BMP5, SSTR5, PSG9, SCNN1G, CPEB1, GAL, GLB1, PADI2, ADTRP, TEK, HCN2, CES1 286 1462 19554 1.636779772894684 0.999999984778176 0.20637397811196886 0.20283402508239848

GOTERM_MF_ALL GO:0005283~amino acid:sodium symporter activity 4 1.2048192771084338 0.0047748641328656544 SLC6A15, SLC6A20, SLC38A3, SLC38A4 289 23 19144 11.520385136151647 0.9818832722085207 0.10272079506344318 0.09757863368958786

GOTERM_BP_ALL GO:0007417~central nervous system development 27 8.132530120481928 0.005161143206244435 NDNF, SEMA3E, HOXD10, VTN, UCHL1, SCIN, FGF9, DNER, DRD1, CTNNA2, WNT2, SH3GL2, SPTBN2, KCNJ10, WNT5A, FOXJ1, SYNGR3, L1CAM, BMP7, MYO16, BMP5, ALDH1A3, FA2H, NFASC, PADI2, CNTN3, TAGLN3 286 1038 19554 1.7784267755366021 0.9999999983405878 0.2290843791317132 0.22515487237241347

GOTERM_CC_ALL GO:0098793~presynapse 18 5.421686746987952 0.0051617568951262925 RAB3B, CALCA, KCNJ10, GRIK5, LGI3, SYN3, SYNGR3, SNAP91, RIMS2, SV2B, ADORA1, RGS7BP, DRD1, ERC2, ATP6V1G3, SH3GL2, SPTBN2, SNCA 299 589 20791 2.1250120662536696 0.9005505350059528 0.06771010515371548 0.06224471550005235

GOTERM_CC_ALL GO:0005903~brush border 7 2.108433734939759 0.005316492957517463 SLC9A3, SLC15A1, SCIN, MTTP, CA4, ITLN1, TRPM6 299 111 20791 4.385097472053994 0.9072160313326307 0.06774731025865109 0.06227891750234742

GOTERM_MF_ALL GO:0015297~antiporter activity 8 2.4096385542168677 0.005328299526172987 SLC9A2, SLC24A2, SLC9A3, SLC9A4, SLC7A8, SLC30A2, SLC4A3, SLC38A3 289 140 19144 3.7852694018783986 0.988633139874014 0.1117610825614784 0.10616636805899678

GOTERM_BP_ALL GO:0003344~pericardium morphogenesis 3 0.9036144578313252 0.005593464909799866 WNT5A, BMP7, BMP5 286 8 19554 25.638986013986013 0.9999999996963105 0.24008872458987116 0.23597045921672183

GOTERM_BP_ALL GO:0051584~regulation of dopamine uptake involved in synaptic transmission 3 0.9036144578313252 0.005593464909799866 RAB3B, DRD1, SNCA 286 8 19554 25.638986013986013 0.9999999996963105 0.24008872458987116 0.23597045921672183

GOTERM_BP_ALL GO:0051940~regulation of catecholamine uptake involved in synaptic transmission 3 0.9036144578313252 0.005593464909799866 RAB3B, DRD1, SNCA 286 8 19554 25.638986013986013 0.9999999996963105 0.24008872458987116 0.23597045921672183

GOTERM_MF_ALL GO:0005244~voltage-gated monoatomic ion channel activity 9 2.710843373493976 0.005675542261135036 HCN4, KCNG3, KCNJ10, KCNS1, KCNK13, CLCNKB, KCNK15, HCN2, LRRC52 289 178 19144 3.349325453909257 0.9915166718574302 0.1124442996761945 0.10681538360182002

GOTERM_MF_ALL GO:0099094~ligand-gated monoatomic cation channel activity 8 2.4096385542168677 0.0057507435761101474 HCN4, SCNN1G, MCOLN3, CHRNB4, KCNJ10, TMEM63C, GRIK5, HCN2 289 142 19144 3.7319557483308152 0.9920376670835674 0.1124442996761945 0.10681538360182002

GOTERM_MF_ALL GO:0098960~postsynaptic neurotransmitter receptor activity 6 1.8072289156626504 0.005762937885669086 GABRB2, CHRNB4, GLRB, GRIK5, DRD1, GRM1 289 76 19144 5.229648515753051 0.9921190848205533 0.1124442996761945 0.10681538360182002

GOTERM_BP_ALL GO:0032940~secretion by cell 15 4.518072289156627 0.005839455846342973 RAB3B, SYT3, GCGR, LGI3, MTTP, SYN3, SYNGR3, RIMS2, SCIN, GAL, CLNK, PTGDS, ERC2, SLC38A3, SNCA 286 444 19554 2.30981855981856 0.9999999998844857 0.2458503149222887 0.2416332204266939

GOTERM_BP_ALL GO:0045055~regulated exocytosis 8 2.4096385542168677 0.00585357892672116 RIMS2, SYT3, SCIN, CLNK, PTGDS, SYNGR3, ERC2, SNCA 286 147 19554 3.720850577993435 0.9999999998907225 0.2458503149222887 0.2416332204266939

GOTERM_BP_ALL GO:0043268~positive regulation of potassium ion transport 5 1.5060240963855422 0.006012431776634335 GAL, ADORA1, DRD1, LRRC52, RGS7 286 50 19554 6.837062937062938 0.9999999999414695 0.24983572893120973 0.24555027223935336

GOTERM_BP_ALL GO:0090066~regulation of anatomical structure size 17 5.120481927710843 0.006083023313044238 RAB3B, CALCA, CCL11, CRACD, WNT5A, AQP4, SEMA3E, L1CAM, KNG1, SCPEP1, SCIN, ARHGAP40, ADORA1, DRD1, KANK4, SLC12A8, SPTBN2 286 541 19554 2.1484301280974107 0.9999999999556519 0.25010830590263994 0.24581817367133507

GOTERM_MF_ALL GO:0022832~voltage-gated channel activity 9 2.710843373493976 0.006262848251592539 HCN4, KCNG3, KCNJ10, KCNS1, KCNK13, CLCNKB, KCNK15, HCN2, LRRC52 289 181 19144 3.2938117723527496 0.9948294195612412 0.11942112916104863 0.11344295582998304

GOTERM_BP_ALL GO:0030003~intracellular monoatomic cation homeostasis 15 4.518072289156627 0.006533186565544319 SLC24A2, CALCA, CCL11, SLC30A2, WNT5A, SLC4A3, GRM1, SLC9A2, SCNN1G, SLC9A3, SLC9A4, SV2B, ADORA1, ERC2, ATP6V0D2 286 450 19554 2.279020979020979 0.9999999999924457 0.26521740216200795 0.2606681021249228

GOTERM_BP_ALL GO:0007610~behavior 19 5.72289156626506 0.006586300053690418 SLC24A2, MCOLN3, CHRNB4, CALCA, CCL11, KCNJ10, FOS, HOXD10, GRM1, ALDH1A3, ANKFN1, UCHL1, ADGRF1, GAL, GLRB, LRRTM1, DRD1, SPTBN2, SNCA 286 644 19554 2.01714589758068 0.9999999999938698 0.26521740216200795 0.2606681021249228

GOTERM_CC_ALL GO:0005788~endoplasmic reticulum lumen 12 3.614457831325301 0.006721164491069488 VTN, COL26A1, IGFBP5, AHSG, WNT5A, MTTP, ARSI, NOTUM, CHRDL1, KNG1, F5, CES1 299 320 20791 2.6075668896321074 0.9505977121897535 0.08326776008380532 0.07654659559273584

GOTERM_BP_ALL GO:0035524~proline transmembrane transport 3 0.9036144578313252 0.007122696429420677 SLC36A2, SLC7A8, SLC6A20 286 9 19554 22.79020979020979 0.9999999999992569 0.2838903291154813 0.2790207305361835

GOTERM_MF_ALL GO:0008289~lipid binding 24 7.228915662650602 0.007214799352993772 SYT3, MCOLN3, PLA2G4F, DGKB, MTTP, WNT5A, SYT14, PRLR, SNAP91, EPN3, HSD11B1, SLC9A3, HSD11B2, ADH4, SCIN, BPIFA2, FABP7, DOK7, STAP1, PTGDS, PROM1, SH3GL2, SPTBN2, SNCA 289 878 19144 1.8107211261832885 0.9976840091701208 0.12932901625102955 0.12285485810735465

GOTERM_MF_ALL GO:0022843~voltage-gated monoatomic cation channel activity 8 2.4096385542168677 0.007428405207087354 HCN4, KCNG3, KCNJ10, KCNS1, KCNK13, KCNK15, HCN2, LRRC52 289 149 19144 3.556628968207891 0.9980661445588332 0.12932901625102955 0.12285485810735465

GOTERM_BP_ALL GO:0030534~adult behavior 8 2.4096385542168677 0.00749437298905468 UCHL1, CHRNB4, KCNJ10, GLRB, DRD1, HOXD10, SPTBN2, SNCA 286 154 19554 3.5517210062664604 0.9999999999998279 0.29568707974997555 0.2906151303533426

GOTERM_MF_ALL GO:0015101~organic cation transmembrane transporter activity 6 1.8072289156626504 0.007523762438775942 SLC36A2, SLC7A8, SLC6A20, SLC7A10, SLC38A3, SLC38A4 289 81 19144 4.906830706138665 0.9982157389017186 0.12932901625102955 0.12285485810735465

GOTERM_MF_ALL GO:0015180~L-alanine transmembrane transporter activity 3 0.9036144578313252 0.007572052921059103 SLC36A2, SLC7A8, SLC38A3 289 9 19144 22.08073817762399 0.9982870291753908 0.12932901625102955 0.12285485810735465

GOTERM_MF_ALL GO:0005295~neutral L-amino acid:sodium symporter activity 3 0.9036144578313252 0.007572052921059103 SLC6A15, SLC38A3, SLC38A4 289 9 19144 22.08073817762399 0.9982870291753908 0.12932901625102955 0.12285485810735465

GOTERM_MF_ALL GO:0005272~sodium channel activity 5 1.5060240963855422 0.0077073311234225 HCN4, SCNN1G, MCOLN3, GRIK5, HCN2 289 52 19144 6.369443705083844 0.998471948603095 0.12932901625102955 0.12285485810735465

GOTERM_CC_ALL GO:0034703~cation channel complex 10 3.0120481927710845 0.007767027562058818 HCN4, DPP10, SCNN1G, KCNG3, KCNIP1, KCNS1, KCNIP3, GRIK5, HCN2, LRRC52 299 239 20791 2.9094191237178317 0.9691190940985607 0.09362417007238467 0.08606706217416528

GOTERM_BP_ALL GO:0051046~regulation of secretion 19 5.72289156626506 0.007917119437079609 RAB3B, PFKFB2, SYT3, CHRNB4, LGI3, PTGER3, NR0B2, SSTR5, RIMS2, SCIN, GAL, SV2B, VSNL1, ADORA1, ADTRP, IL13RA2, PTGES, CES1, SNCA 286 658 19554 1.9742278997598144 0.9999999999999675 0.30924268521232956 0.3039382151894862

GOTERM_BP_ALL GO:0010959~regulation of metal ion transport 14 4.216867469879518 0.008027454385398548 CAMK2B, DPP10, CALCA, KCNIP1, KCNIP3, UNC93A, LRRC52, GAL, PLPP4, KCNS1, ADORA1, DRD1, RGS7, SNCA 286 414 19554 2.3120502685720075 0.9999999999999789 0.31044788939967055 0.3051227463915349

GOTERM_BP_ALL GO:0150104~transport across blood-brain barrier 6 1.8072289156626504 0.008472279691638206 SLC13A3, SLC7A8, SLC16A7, SLC4A3, SLC6A20, SLC38A3 286 86 19554 4.770043909578794 0.9999999999999963 0.32128858714115366 0.31577749258445703

GOTERM_BP_ALL GO:0010232~vascular transport 6 1.8072289156626504 0.008472279691638206 SLC13A3, SLC7A8, SLC16A7, SLC4A3, SLC6A20, SLC38A3 286 86 19554 4.770043909578794 0.9999999999999963 0.32128858714115366 0.31577749258445703

GOTERM_BP_ALL GO:0140352~export from cell 16 4.819277108433735 0.00859052723642775 RAB3B, SYT3, SLC30A2, GCGR, LGI3, MTTP, SYN3, SYNGR3, RIMS2, SCIN, GAL, CLNK, PTGDS, ERC2, SLC38A3, SNCA 286 513 19554 2.132417290312027 0.9999999999999977 0.3226403787066037 0.31710609673698203

GOTERM_BP_ALL GO:0032879~regulation of localization 46 13.855421686746988 0.008712343527951088 PFKFB2, DPP10, RAB3B, CAMK2B, CALCA, GRIK5, PTGER3, ITLN1, LRRC52, RIMS2, VTN, SCIN, SV2B, LRRTM1, ADORA1, ENPP1, STAP1, DRD1, ERC2, IL13RA2, SLC38A3, TRIM67, SNCA, RGS7, SYT3, CHRNB4, KCNJ10, KCNIP1, AHSG, KCNIP3, LGI3, WNT5A, UNC93A, SYNGR3, NR0B2, SNAP91, SSTR5, GPR143, GAL, PLPP4, KCNS1, VSNL1, ADTRP, REEP6, PTGES, CES1 286 2159 19554 1.4567155864052574 0.9999999999999986 0.3240991792397805 0.318539874321945

GOTERM_BP_ALL GO:0015698~inorganic anion transport 8 2.4096385542168677 0.008859028319626861 GABRB2, GLRB, CLCNKB, AQP6, ENPP1, SLC4A3, ANO5, SLC12A8 286 159 19554 3.440031666446761 0.9999999999999992 0.32644683600436336 0.32084726150044834

GOTERM_CC_ALL GO:0045202~synapse 35 10.542168674698797 0.008972609805511072 RAB3B, CAMK2B, GABRB2, CALCA, DGKB, GRIK5, PPM1H, CHRDL1, GRM1, RIMS2, SV2B, LRRTM1, ADORA1, SLC16A7, NEFM, DRD1, KIF1A, ERC2, ATP6V1G3, SH3GL2, SNCA, SPTBN2, CHRNB4, KCNJ10, LGI3, WNT5A, SYN3, SYNGR3, SNAP91, IGSF11, CPEB1, GLRB, DOK7, RGS7BP, PVALB 299 1552 20791 1.5681244181636385 0.9820442578046125 0.0997943852300693 0.09173923305903231

GOTERM_MF_ALL GO:0015078~proton transmembrane transporter activity 9 2.710843373493976 0.009078509629679026 SLC9A2, SLC9A3, SLC9A4, SLC15A1, SLC36A2, SLC30A2, ATP6V1G3, SLC38A3, ATP6V0D2 289 193 19144 3.0890151854707137 0.9995203890076049 0.1493503839078569 0.14187396421282714

GOTERM_CC_ALL GO:0070062~extracellular exosome 47 14.156626506024098 0.009085976521051625 RAB3B, GABRB2, PIGR, PSCA, KLK1, ITLN1, TMPRSS2, CST6, CST5, SERPINA4, KNG1, RIMS2, SCPEP1, VTN, SCEL, UPK1B, SCIN, TTR, BPIFA2, CRTAC1, PODXL, CA4, MAN1C1, ENPP3, PROM1, PTGDS, KRT6A, SLC13A3, CR2, CR1, SLC36A2, TMEM52B, AHSG, WNT5A, EPN3, SCNN1G, SLC9A3, ALDH1A3, C1ORF116, SLPI, PSAT1, GLB1, LCN2, PADI2, RHCG, CPVL, ATP6V0D2 299 2258 20791 1.4473651468876176 0.9829374208254313 0.0997943852300693 0.09173923305903231

GOTERM_CC_ALL GO:0030054~cell junction 48 14.457831325301203 0.00916221729196132 RAB3B, CAMK2B, GABRB2, CALCA, DGKB, GRIK5, PPM1H, CHRDL1, GRM1, RIMS2, PANX2, EPB41L4B, SCIN, SV2B, PODXL, LRRTM1, NRAP, ADORA1, SLC16A7, NEFM, DRD1, CTNNA2, KIF1A, ERC2, ATP6V1G3, SH3GL2, SNCA, SPTBN2, CHRNB4, KCNJ10, LGI3, WNT5A, SYN3, SYNGR3, L1CAM, SNAP91, IGSF11, NFASC, CPEB1, GLRB, GJB3, DOK7, RGS7BP, TEK, PKP3, CDH17, PVALB, TJP3 299 2319 20791 1.439277972716299 0.9835130159793115 0.0997943852300693 0.09173923305903231

GOTERM_CC_ALL GO:0097060~synaptic membrane 14 4.216867469879518 0.00917392330590323 GABRB2, CHRNB4, DGKB, GRIK5, SNAP91, GRM1, RIMS2, GLRB, LRRTM1, ADORA1, SLC16A7, RGS7BP, DRD1, ERC2 299 428 20791 2.274513174756978 0.9835996605654523 0.0997943852300693 0.09173923305903231

GOTERM_BP_ALL GO:0008344~adult locomotory behavior 6 1.8072289156626504 0.00931046382902434 UCHL1, KCNJ10, GLRB, DRD1, HOXD10, SNCA 286 88 19554 4.66163382072473 0.9999999999999999 0.339875436599711 0.33404551999649007

GOTERM_MF_ALL GO:0015658~branched-chain amino acid transmembrane transporter activity 3 0.9036144578313252 0.009371597592263749 SLC7A8, SLC6A15, SLC6A20 289 10 19144 19.87266435986159 0.9996256913262805 0.15120712269056316 0.143637755404504

GOTERM_BP_ALL GO:0002686~negative regulation of leukocyte migration 5 1.5060240963855422 0.009528551528241941 ADORA1, STAP1, PADI2, ADTRP, BMP5 286 57 19554 5.9974236290025775 0.9999999999999999 0.3446159469380835 0.33870471589741497

GOTERM_BP_ALL GO:0050806~positive regulation of synaptic transmission 8 2.4096385542168677 0.01039858301140656 RIMS2, IGSF11, SLC24A2, LRRTM1, ADORA1, DRD1, SLC7A10, SNCA 286 164 19554 3.335152652225823 1.0 0.3677590319765557 0.3614508253348688

GOTERM_BP_ALL GO:0010951~negative regulation of endopeptidase activity 7 2.108433734939759 0.010450909510854502 VTN, CR1, AHSG, CST5, SERPINA4, KNG1, SNCA 286 126 19554 3.7983682983682985 1.0 0.3677590319765557 0.3614508253348688

GOTERM_BP_ALL GO:0003014~renal system process 7 2.108433734939759 0.010450909510854502 HSD11B2, CHRNB4, TMEM63C, CLCNKB, ADORA1, AQP6, AQP4 286 126 19554 3.7983682983682985 1.0 0.3677590319765557 0.3614508253348688

GOTERM_BP_ALL GO:0015824~proline transport 3 0.9036144578313252 0.010674754580417013 SLC36A2, SLC6A15, SLC6A20 286 11 19554 18.64653528289892 1.0 0.37228206599204333 0.36589627530554386

GOTERM_BP_ALL GO:0070633~transepithelial transport 4 1.2048192771084338 0.01112149496689067 SLC9A4, CLCNKB, RHCG, RHBG 286 32 19554 8.54632867132867 1.0 0.381677966031583 0.3751310065528026

GOTERM_BP_ALL GO:0030901~midbrain development 6 1.8072289156626504 0.01115484013483286 UCHL1, FGF9, WNT5A, PADI2, SYNGR3, WNT2 286 92 19554 4.458954089388873 1.0 0.381677966031583 0.3751310065528026

GOTERM_BP_ALL GO:0001508~action potential 7 2.108433734939759 0.011237318508354337 HCN4, KCNG3, CHRNB4, KCNS1, DRD1, SLC4A3, HCN2 286 128 19554 3.7390187937062933 1.0 0.381677966031583 0.3751310065528026

GOTERM_MF_ALL GO:0008514~organic anion transmembrane transporter activity 11 3.313253012048193 0.01152121431735883 SLC13A3, SLC36A2, SLC7A8, GRIK5, SLC6A15, SLC16A7, SLC4A3, SLC6A20, SLC7A10, SLC38A3, SLC38A4 289 285 19144 2.556717052145936 0.9999393763751007 0.18238299645781245 0.1732529775648111

GOTERM_BP_ALL GO:0008015~blood circulation 14 4.216867469879518 0.011777182205340174 HCN4, CALCA, KLK1, GCGR, KNG1, F5, SCNN1G, SCPEP1, HSD11B2, ADORA1, TEK, DRD1, SH3GL2, TJP3 286 435 19554 2.2004340487099108 1.0 0.39656615253498895 0.389763814537077

GOTERM_CC_ALL GO:0045211~postsynaptic membrane 11 3.313253012048193 0.0119148830554008 GABRB2, CHRNB4, GLRB, DGKB, GRIK5, LRRTM1, ADORA1, SLC16A7, RGS7BP, DRD1, GRM1 299 301 20791 2.5411504572273027 0.9952325783239941 0.12193975665858167 0.11209708571753023

GOTERM_CC_ALL GO:0031526~brush border membrane 5 1.5060240963855422 0.01203308430743878 SLC9A3, MTTP, CA4, ITLN1, TRPM6 299 62 20791 5.607670730391628 0.9954802848158946 0.12193975665858167 0.11209708571753023

GOTERM_BP_ALL GO:0098661~inorganic anion transmembrane transport 7 2.108433734939759 0.012064674165794775 GABRB2, GLRB, CLCNKB, AQP6, SLC4A3, ANO5, SLC12A8 286 130 19554 3.6814954276492737 1.0 0.40277450676576404 0.3958656762605653

GOTERM_BP_ALL GO:0051239~regulation of multicellular organismal process 63 18.97590361445783 0.012175708123377019 SLC4A3, GRM1, RIMS2, EPB41L4B, PANX2, FGF9, LRRTM1, ADORA1, ENPP1, ENPP3, PTGDS, CCBE1, CHIA, CHRNB4, CR1, IGFBP5, AHSG, WNT5A, FOS, UNC93A, PRLR, PSG9, GAL, ANGPTL7, ESRP1, PKP3, ADTRP, PTGES, HCN4, CAMK2B, CALCA, CCL11, DAPL1, PTGER3, IL20RA, SEMA3E, VTCN1, KNG1, VTN, WNT11, SCIN, STAP1, IGF2BP2, PROM1, WNT2, SNCA, AFAP1L2, DMRT2, ST8SIA2, MYBPH, G0S2, FOXJ1, L1CAM, FOXN1, BMP7, BMP5, IGSF11, FA2H, LCN2, CLNK, LRRN1, NOTUM, TEK 286 3230 19554 1.3335447833899847 1.0 0.40303657567720874 0.3961232498783422

GOTERM_CC_ALL GO:1903561~extracellular vesicle 48 14.457831325301203 0.012486769060410283 RAB3B, GABRB2, PIGR, PSCA, KLK1, ITLN1, TMPRSS2, CST6, CST5, SERPINA4, KNG1, RIMS2, SCPEP1, VTN, SCEL, UPK1B, SCIN, TTR, BPIFA2, CRTAC1, PODXL, CA4, MAN1C1, ENPP3, PROM1, PTGDS, KRT6A, SLC13A3, CR2, CR1, SLC36A2, TMEM52B, AHSG, WNT5A, F5, EPN3, SCNN1G, SLC9A3, ALDH1A3, C1ORF116, SLPI, PSAT1, GLB1, LCN2, PADI2, RHCG, CPVL, ATP6V0D2 299 2361 20791 1.4136745526171526 0.9963174918963427 0.12193975665858167 0.11209708571753023

GOTERM_BP_ALL GO:0048706~embryonic skeletal system development 7 2.108433734939759 0.01249403330378859 HOXB9, WNT11, FGF9, DMRT2, WNT5A, HOXD10, BMP7 286 131 19554 3.6533924091176004 1.0 0.40620550258021326 0.3992378198682639

GOTERM_CC_ALL GO:0065010~extracellular membrane-bounded organelle 48 14.457831325301203 0.012576746202454612 RAB3B, GABRB2, PIGR, PSCA, KLK1, ITLN1, TMPRSS2, CST6, CST5, SERPINA4, KNG1, RIMS2, SCPEP1, VTN, SCEL, UPK1B, SCIN, TTR, BPIFA2, CRTAC1, PODXL, CA4, MAN1C1, ENPP3, PROM1, PTGDS, KRT6A, SLC13A3, CR2, CR1, SLC36A2, TMEM52B, AHSG, WNT5A, F5, EPN3, SCNN1G, SLC9A3, ALDH1A3, C1ORF116, SLPI, PSAT1, GLB1, LCN2, PADI2, RHCG, CPVL, ATP6V0D2 299 2362 20791 1.4130760451859006 0.9964641455365537 0.12193975665858167 0.11209708571753023

GOTERM_CC_ALL GO:0043230~extracellular organelle 48 14.457831325301203 0.012576746202454612 RAB3B, GABRB2, PIGR, PSCA, KLK1, ITLN1, TMPRSS2, CST6, CST5, SERPINA4, KNG1, RIMS2, SCPEP1, VTN, SCEL, UPK1B, SCIN, TTR, BPIFA2, CRTAC1, PODXL, CA4, MAN1C1, ENPP3, PROM1, PTGDS, KRT6A, SLC13A3, CR2, CR1, SLC36A2, TMEM52B, AHSG, WNT5A, F5, EPN3, SCNN1G, SLC9A3, ALDH1A3, C1ORF116, SLPI, PSAT1, GLB1, LCN2, PADI2, RHCG, CPVL, ATP6V0D2 299 2362 20791 1.4130760451859006 0.9964641455365537 0.12193975665858167 0.11209708571753023

GOTERM_BP_ALL GO:0007268~chemical synaptic transmission 14 4.216867469879518 0.012687375503091847 GABRB2, CHRNB4, GRIK5, SYN3, CHRDL1, GRM1, RIMS2, SV2B, GLRB, ADORA1, DRD1, ERC2, PVALB, SNCA 286 439 19554 2.180384535737611 1.0 0.40620550258021326 0.3992378198682639

GOTERM_BP_ALL GO:0098916~anterograde trans-synaptic signaling 14 4.216867469879518 0.012687375503091847 GABRB2, CHRNB4, GRIK5, SYN3, CHRDL1, GRM1, RIMS2, SV2B, GLRB, ADORA1, DRD1, ERC2, PVALB, SNCA 286 439 19554 2.180384535737611 1.0 0.40620550258021326 0.3992378198682639

GOTERM_BP_ALL GO:0051798~positive regulation of hair follicle development 3 0.9036144578313252 0.012687422251609324 GAL, WNT5A, FOXN1 286 12 19554 17.092657342657343 1.0 0.40620550258021326 0.3992378198682639

GOTERM_BP_ALL GO:0007628~adult walking behavior 4 1.2048192771084338 0.013134879942265833 UCHL1, KCNJ10, GLRB, DRD1 286 34 19554 8.04360345536816 1.0 0.41374871818137376 0.4066516459545043

GOTERM_BP_ALL GO:0010464~regulation of mesenchymal cell proliferation 4 1.2048192771084338 0.013134879942265833 WNT11, FGF9, WNT5A, WNT2 286 34 19554 8.04360345536816 1.0 0.41374871818137376 0.4066516459545043

GOTERM_BP_ALL GO:0043269~regulation of monoatomic ion transport 15 4.518072289156627 0.013611493349560935 CAMK2B, DPP10, CALCA, KCNJ10, KCNIP1, KCNIP3, UNC93A, LRRC52, GAL, PLPP4, KCNS1, ADORA1, DRD1, RGS7, SNCA 286 492 19554 2.0844704076411396 1.0 0.4225307220226469 0.4152830112250234

GOTERM_BP_ALL GO:0048732~gland development 14 4.216867469879518 0.013774960355419625 CCL11, IGFBP5, ONECUT2, WNT5A, TFCP2L1, FOXN1, BMP7, PRLR, FA2H, VTN, ALDH1A3, HOXB9, WNT11, WNT2 286 444 19554 2.155830655830656 1.0 0.4225307220226469 0.4152830112250234

GOTERM_BP_ALL GO:0034308~primary alcohol metabolic process 6 1.8072289156626504 0.013791920587335322 HSD11B2, ALDH1A3, ADH4, DGAT2, CYP11A1, HAO1 286 97 19554 4.2291110950904764 1.0 0.4225307220226469 0.4152830112250234

GOTERM_BP_ALL GO:0010466~negative regulation of peptidase activity 7 2.108433734939759 0.013846372867101588 VTN, CR1, AHSG, CST5, SERPINA4, KNG1, SNCA 286 134 19554 3.5716000417492957 1.0 0.4225307220226469 0.4152830112250234

GOTERM_BP_ALL GO:0070050~neuron cellular homeostasis 5 1.5060240963855422 0.014165734473228364 SLC24A2, SV2B, ADORA1, ATP6V1G3, ERC2 286 64 19554 5.34145541958042 1.0 0.426375862441133 0.4190621955738632

GOTERM_BP_ALL GO:0009653~anatomical structure morphogenesis 48 14.457831325301203 0.014280889023542513 HACD1, CCL11, TENM3, ONECUT2, AQP6, NDNF, SEMA3E, TFCP2L1, CST6, HOXD10, VTN, UCHL1, WNT11, FGF9, PODXL, NRAP, CA9, IGF2BP2, CTNNA2, PROM1, WNT2, SH3GL2, KRT6A, SPTBN2, CCBE1, CR2, ST14, IGFBP5, SPAG6, ST8SIA2, EYA4, WNT5A, MYBPH, FOXJ1, L1CAM, FOXN1, BMP7, MYO16, BMP5, ALDH1A3, NFASC, HOXB9, EMCN, ASXL3, CNTN3, ADTRP, TEK, CDH17 286 2343 19554 1.4006787066966326 1.0 0.426375862441133 0.4190621955738632

GOTERM_BP_ALL GO:0071495~cellular response to endogenous stimulus 28 8.433734939759036 0.014419927672098633 HCN4, GABRB2, CALCA, DOCK3, ONECUT2, WFIKKN2, NDNF, CHRDL1, FGF9, CYP11A1, ENPP1, DRD1, WNT2, SH3GL2, IGFBP5, GCGR, WNT5A, FOS, BMP7, PRLR, BMP5, SSTR5, PSG9, SCNN1G, CPEB1, PADI2, ADTRP, HCN2 286 1185 19554 1.6155085420908206 1.0 0.426375862441133 0.4190621955738632

GOTERM_BP_ALL GO:0044255~cellular lipid metabolic process 24 7.228915662650602 0.014469130373801903 HACD1, PLA2G4F, DGAT2, DGKB, ST8SIA2, MTTP, PLAAT5, SCPEP1, FA2H, ALDH1A3, ADH4, PLPP4, GLB1, LIPH, INPP5J, HAO1, ADTRP, PTGDS, ST6GALNAC3, B4GALNT2, ST6GALNAC5, PTGES, CES1, SNCA 286 965 19554 1.7004094351244612 1.0 0.426375862441133 0.4190621955738632

GOTERM_BP_ALL GO:0007399~nervous system development 47 14.156626506024098 0.014778982184637456 CAMK2B, GABRB2, TENM3, ONECUT2, NDNF, SEMA3E, CHRDL1, HOXD10, VTN, UCHL1, SCIN, WNT11, FGF9, CRTAC1, DNER, ADORA1, NCAM2, DRD1, IGF2BP2, CTNNA2, PROM1, WNT2, SH3GL2, SPTBN2, ST14, MCOLN3, KCNJ10, IRX6, SPAG6, ST8SIA2, WNT5A, FOXJ1, FOS, SYNGR3, L1CAM, BMP7, MYO16, BMP5, ALDH1A3, FA2H, NFASC, ADGRF1, GLRB, FABP7, PADI2, CNTN3, TAGLN3 286 2288 19554 1.4044665998337327 1.0 0.426375862441133 0.4190621955738632

GOTERM_BP_ALL GO:0072488~ammonium transmembrane transport 3 0.9036144578313252 0.014851263304109363 RHCG, RHBG, HCN2 286 13 19554 15.777837547068318 1.0 0.426375862441133 0.4190621955738632

GOTERM_BP_ALL GO:0002924~negative regulation of humoral immune response mediated by circulating immunoglobulin 3 0.9036144578313252 0.014851263304109363 CR2, CR1, FOXJ1 286 13 19554 15.777837547068318 1.0 0.426375862441133 0.4190621955738632

GOTERM_BP_ALL GO:0006687~glycosphingolipid metabolic process 5 1.5060240963855422 0.014926084863931377 FA2H, GLB1, ST8SIA2, ST6GALNAC3, ST6GALNAC5 286 65 19554 5.259279182356106 1.0 0.426375862441133 0.4190621955738632

GOTERM_BP_ALL GO:0051952~regulation of amine transport 6 1.8072289156626504 0.014954811355462167 RAB3B, SYT3, ADORA1, DRD1, SLC38A3, SNCA 286 99 19554 4.143674507310871 1.0 0.426375862441133 0.4190621955738632

GOTERM_BP_ALL GO:0007167~enzyme-linked receptor protein signaling pathway 18 5.421686746987952 0.01512770576757313 PIGR, DOCK3, IGFBP5, ONECUT2, WNT5A, WFIKKN2, VWDE, FOS, CHRDL1, BMP7, PRLR, BMP5, PSG9, FGF9, CLNK, STAP1, TEK, MUC20 286 651 19554 1.8904321484966649 1.0 0.42817984585609165 0.4208352350848786

GOTERM_BP_ALL GO:0007565~female pregnancy 8 2.4096385542168677 0.015289921378804493 PSG9, HSD11B2, CALCA, IGFBP5, PSG4, FOS, SLC38A3, PRLR 286 177 19554 3.0901979376555646 1.0 0.42965779068784427 0.42228782858439173

GOTERM_MF_ALL GO:0005343~organic acid:sodium symporter activity 4 1.2048192771084338 0.015460813711862767 SLC13A3, SLC6A15, SLC6A20, SLC38A4 289 35 19144 7.570538803756797 0.9999978656806052 0.24021523526394187 0.22819015793249306

GOTERM_CC_ALL GO:0110165~cellular anatomical entity 299 90.06024096385542 0.01564341084929982 EHF, HPSE2, PLEKHB1, AQP6, AQP4, SLC4A3, ISM2, C10ORF71, MCIDAS, BPIFA2, LIPH, PPP4R4, SMCO3, CKMT1B, PRSS3, PTGDS, SLC12A8, CCBE1, CHIA, TMEM52B, SLC6A15, KCNK13, KCNK15, UNC93A, PSG9, HOXB9, PLPP4, SLPI, PSG4, PADI2, TAGLN3, ATP6V0D2, CCNO, CES1, PTGES, CFAP61, HACD1, KLK1, CRACD, TMEM63C, MTTP, IL20RA, CLCNKB, SLC6A20, HSD11B1, ADH4, HSD11B2, INPP5J, ARSI, HAO1, PROM1, TRPM6, B4GALNT2, SPTBN2, RBM11, ST14, AFAP1L2, TMEM86A, ST8SIA2, L1CAM, NR0B2, SNAP91, FA2H, CPEB1, PPP1R1A, GJB3, LYPD6B, FNDC10, LRRN1, XKR4, HRK, GABRB2, PIGR, TENM3, DIRAS1, GRIK5, WFIKKN2, HEPACAM2, KRT23, TFCP2L1, SLC7A10, CHRDL1, SERPINA4, GPRC6A, M1AP, EPB41L4B, PANX2, UCHL1, NMRK2, CRTAC1, DNER, NEFM, KIF1A, ANKRD2, AHSG, SLC2A12, TMEM40, FOS, SYN3, PLAAT5, ANO5, DUSP9, F5, SLC7A4, ANKFN1, GAL, VSNL1, SLC7A8, ANGPTL7, RGS7BP, POPDC3, CCL11, PNCK, FAXC, MCCD1, PTGER3, TMPRSS2, HOXD10, CST6, CST5, CPN1, C10ORF90, SCEL, NRAP, CTNNA2, ATP6V1G3, RHBG, BIK, DMRT2, ATRNL1, GCGR, CKMT1A, MOCOS, MYBPH, TMEM61, G0S2, KLK15, GPR143, KCNS1, SMOC1, FABP7, NOTUM, TJP3, RAB3B, KCNG3, DGKB, SERTM2, NALF2, RIMS2, ARHGAP40, FGF9, ADORA1, ENPP1, SLC16A7, ENPP3, MALRD1, IL13RA2, GYG2, SH3GL2, KRT6A, RGS7, CHRNB4, SLC15A1, DGAT2, IGFBP5, SLC30A2, DIO1, WNT5A, VWDE, MAT1A, PRLR, SLC9A2, SCNN1G, SLC9A3, PCP4, C1ORF116, SLC9A4, EMCN, DOK7, CLIP4, ADTRP, PKP3, RHCG, PVALB, KANK4, PFKFB2, CALCA, GMPR, VTCN1, PLCXD2, KNG1, LRRC52, FBXO40, VTN, PCSK1N, KLC3, STK33, SCIN, CYP11A1, PODXL, SOHLH2, STAP1, UGT3A2, DRD1, SLC38A3, WNT2, CD300LG, SLC38A4, SYT3, BMP7, FOXN1, KY, BMP5, IGSF11, BFSP2, UST, LCN2, CNTN3, REEP6, MUC20, DPP10, DOCK3, ST6GALNAC2, ONECUT2, PSCA, BNC1, DUSP15, GRM1, SCPEP1, UPK1B, LRRTM1, CA4, ZNF385B, CA9, ERC2, TRIM67, PLA2G4F, SLC13A3, CR2, SLC36A2, CR1, SYNGR3, SSTR5, EPN3, ALDH1A3, ADGRF1, ESRP1, ELF5, NAT8L, ST6GALNAC3, CDH17, ST6GALNAC5, COLEC12, SLC24A2, HCN4, CAMK2B, TRIM50, CCDC187, DAPL1, PPM1H, ITLN1, NDNF, SEMA3E, TTR, IGFBPL1, WNT11, SV2B, KLRG2, MAN1C1, ZPLD1, NCAM2, IGF2BP2, MAPK4, SNCA, COL26A1, MCOLN3, KCNJ10, KCNIP1, PDE6G, IRX6, SPAG6, KCNIP3, LGI3, EYA4, FOXJ1, SYT14, TPD52L1, MYO16, NFASC, GLRB, GLB1, PSAT1, ASXL3, CLNK, TLL2, THRSP, TEK, CPVL, HCN2 299 20505 20791 1.013947817605462 0.9991169324517563 0.14844598380399404 0.1364637967704878

GOTERM_BP_ALL GO:0060541~respiratory system development 9 2.710843373493976 0.015693294750389476 HSD11B1, ALDH1A3, CCBE1, WNT11, IGFBP5, FGF9, WNT5A, FOXJ1, WNT2 286 220 19554 2.796980292434838 1.0 0.43784292353586635 0.43033256104817996

GOTERM_BP_ALL GO:0050896~response to stimulus 145 43.674698795180724 0.01585958475753003 DGKB, PLEKHB1, AQP4, RIMS2, ARHGAP40, FGF9, BPIFA2, ADORA1, ENPP1, ENPP3, IL13RA2, PRSS3, PTGDS, SH3GL2, KRT6A, RGS7, CHIA, CHRNB4, DGAT2, IGFBP5, SLC30A2, WNT5A, VWDE, PRLR, PSG9, SCNN1G, HOXB9, PLPP4, SLPI, PSG4, PADI2, ADTRP, CCNO, CES1, PTGES, PFKFB2, CALCA, MTTP, IL20RA, GMPR, VTCN1, PLCXD2, KNG1, VTN, ADH4, HSD11B2, STK33, PCSK1N, CYP11A1, STAP1, UGT3A2, HAO1, DRD1, SLC38A3, TRPM6, WNT2, RBM11, SYT3, AFAP1L2, ST8SIA2, L1CAM, FOXN1, BMP7, NR0B2, BMP5, BFSP2, CPEB1, PPP1R1A, GJB3, LCN2, REEP6, MUC20, GABRB2, PIGR, DOCK3, TENM3, ONECUT2, DIRAS1, GRIK5, WFIKKN2, DUSP15, CHRDL1, GRM1, GPRC6A, EPB41L4B, PANX2, UCHL1, UPK1B, DNER, ZNF385B, CA9, CR2, PLA2G4F, CR1, AHSG, FOS, DUSP9, ANO5, SSTR5, F5, ALDH1A3, ANKFN1, GAL, ADGRF1, ANGPTL7, RGS7BP, CDH17, HCN4, CAMK2B, COLEC12, SLC24A2, CCL11, DAPL1, PTGER3, ITLN1, NDNF, SEMA3E, CPN1, SCEL, WNT11, TTR, IGFBPL1, MAN1C1, ZPLD1, CTNNA2, MAPK4, SNCA, MCOLN3, KCNJ10, PDE6G, ATRNL1, GCGR, KCNIP3, EYA4, G0S2, FOXJ1, MYO16, GPR143, GLRB, GLB1, CLNK, THRSP, TEK, NOTUM, HCN2 286 8616 19554 1.1506199232522352 1.0 0.4393442415809383 0.4318081268379985

GOTERM_BP_ALL GO:0071676~negative regulation of mononuclear cell migration 4 1.2048192771084338 0.016520061790283685 STAP1, PADI2, ADTRP, BMP5 286 37 19554 7.391419391419392 1.0 0.4544180376961132 0.44662336065421876

GOTERM_MF_ALL GO:0008236~serine-type peptidase activity 9 2.710843373493976 0.016558634714188905 DPP10, SCPEP1, ST14, KLK1, TLL2, TMPRSS2, KLK15, CPVL, PRSS3 289 215 19144 2.7729299106783616 0.9999991620479938 0.25259444591280894 0.23994967031288286

GOTERM_BP_ALL GO:0015803~branched-chain amino acid transport 3 0.9036144578313252 0.017161475433040935 SLC7A8, SLC6A15, SLC6A20 286 14 19554 14.650849150849153 1.0 0.468760300989216 0.46071960970240666

GOTERM_BP_ALL GO:0099537~trans-synaptic signaling 14 4.216867469879518 0.017309736852564123 GABRB2, CHRNB4, GRIK5, SYN3, CHRDL1, GRM1, RIMS2, SV2B, GLRB, ADORA1, DRD1, ERC2, PVALB, SNCA 286 457 19554 2.0945050573059327 1.0 0.4695266121258018 0.4614727762291227

GOTERM_BP_ALL GO:0010817~regulation of hormone levels 16 4.819277108433735 0.017546423063445303 PFKFB2, DGAT2, DIO1, NR0B2, SSTR5, BMP5, RIMS2, SCPEP1, ALDH1A3, ADH4, PCSK1N, GAL, SLC7A8, VSNL1, CYP11A1, ADORA1 286 559 19554 1.9569410911092484 1.0 0.47266433438494726 0.46455667683149326

GOTERM_MF_ALL GO:0030594~neurotransmitter receptor activity 6 1.8072289156626504 0.01759340439247322 GABRB2, CHRNB4, GLRB, GRIK5, DRD1, GRM1 289 100 19144 3.974532871972318 0.9999996531961781 0.25903983685613113 0.24607240759754057

GOTERM_BP_ALL GO:0090659~walking behavior 4 1.2048192771084338 0.017746786298461714 UCHL1, KCNJ10, GLRB, DRD1 286 38 19554 7.196908354803091 1.0 0.47478731014925657 0.46664323698489396

GOTERM_MF_ALL GO:0004869~cysteine-type endopeptidase inhibitor activity 4 1.2048192771084338 0.017956697038699756 AHSG, CST6, CST5, KNG1 289 37 19144 7.161320490040213 0.999999745624716 0.25903983685613113 0.24607240759754057

GOTERM_MF_ALL GO:0030551~cyclic nucleotide binding 4 1.2048192771084338 0.017956697038699756 HCN4, POPDC3, PDE6G, HCN2 289 37 19144 7.161320490040213 0.999999745624716 0.25903983685613113 0.24607240759754057

GOTERM_MF_ALL GO:0015386~potassium:proton antiporter activity 3 0.9036144578313252 0.018216150625162977 SLC9A2, SLC9A3, SLC9A4 289 14 19144 14.194760257043994 0.9999997961501396 0.25903983685613113 0.24607240759754057

GOTERM_BP_ALL GO:1901700~response to oxygen-containing compound 35 10.542168674698797 0.018440381522777362 COLEC12, PFKFB2, HCN4, HSD11B2, ADH4, WNT11, CYP11A1, MAN1C1, UGT3A2, ENPP1, STAP1, CA9, DRD1, WNT2, SNCA, RGS7, CHRNB4, DGAT2, IGFBP5, ST8SIA2, GCGR, WNT5A, FOS, BMP7, NR0B2, F5, SCNN1G, CPEB1, GAL, SLPI, GJB3, GLB1, TEK, HCN2, CES1 286 1610 19554 1.4863180297962908 1.0 0.4885274856597247 0.4801477259210658

GOTERM_BP_ALL GO:0098656~monoatomic anion transmembrane transport 7 2.108433734939759 0.018510514049574822 GABRB2, MCOLN3, GLRB, CLCNKB, SLC4A3, ANO5, SLC12A8 286 143 19554 3.3468140251357035 1.0 0.4885274856597247 0.4801477259210658

GOTERM_MF_ALL GO:0017171~serine hydrolase activity 9 2.710843373493976 0.018713340930090295 DPP10, SCPEP1, ST14, KLK1, TLL2, TMPRSS2, KLK15, CPVL, PRSS3 289 220 19144 2.7099087763447622 0.9999998666602635 0.2616748840057626 0.24857554535469942

GOTERM_BP_ALL GO:0060537~muscle tissue development 12 3.614457831325301 0.018863680558128395 HCN4, POPDC3, FGF9, DNER, WNT5A, NRAP, FOS, WNT2, HOXD10, B4GALNT2, BMP7, BMP5 286 365 19554 2.2478015135549385 1.0 0.49438213067064807 0.4859019456335427

GOTERM_BP_ALL GO:0006688~glycosphingolipid biosynthetic process 4 1.2048192771084338 0.01902295162310563 FA2H, ST8SIA2, ST6GALNAC3, ST6GALNAC5 286 39 19554 7.012372243141474 1.0 0.49438213067064807 0.4859019456335427

GOTERM_MF_ALL GO:0004866~endopeptidase inhibitor activity 8 2.4096385542168677 0.019057403343963837 PCSK1N, SLPI, AHSG, WFIKKN2, CST6, CST5, SERPINA4, KNG1 289 179 19144 2.9605459009104793 0.9999999006125299 0.2621174000915682 0.24899590926457668

GOTERM_BP_ALL GO:0060079~excitatory postsynaptic potential 5 1.5060240963855422 0.01911205881496873 CHRNB4, GLRB, GRIK5, ADORA1, SNCA 286 70 19554 4.883616383616384 1.0 0.49438213067064807 0.4859019456335427

GOTERM_BP_ALL GO:0099509~regulation of presynaptic cytosolic calcium ion concentration 3 0.9036144578313252 0.01961336335510405 SV2B, ADORA1, ERC2 286 15 19554 13.674125874125876 1.0 0.5040118241120817 0.49536646000160817

GOTERM_BP_ALL GO:1901615~organic hydroxy compound metabolic process 15 4.518072289156627 0.019767749090691088 PFKFB2, DGAT2, DIO1, NR0B2, HSD11B2, ALDH1A3, ADH4, STK33, CYP11A1, PSAT1, INPP5J, HAO1, DRD1, CES1, SNCA 286 517 19554 1.9836739662658425 1.0 0.5046590061976431 0.49600254090956264

GOTERM_BP_ALL GO:0048857~neural nucleus development 5 1.5060240963855422 0.02002766403930487 ALDH1A3, UCHL1, FGF9, PADI2, SYNGR3 286 71 19554 4.814833054269674 1.0 0.5063065095649749 0.4976217844905118

GOTERM_BP_ALL GO:0030155~regulation of cell adhesion 21 6.325301204819277 0.020091528157340277 COL26A1, HACD1, CR1, TENM3, ONECUT2, DAPL1, WNT5A, FOXJ1, NDNF, SEMA3E, VTCN1, BMP7, PRLR, KNG1, EPB41L4B, VTN, EMCN, PODXL, TEK, ADTRP, B4GALNT2 286 832 19554 1.725700981710597 1.0 0.5063065095649749 0.4976217844905118

GOTERM_BP_ALL GO:0006885~regulation of pH 6 1.8072289156626504 0.020258562815525922 SLC9A2, SLC9A3, SLC9A4, RHCG, SLC4A3, ATP6V0D2 286 107 19554 3.8338670675119273 1.0 0.507243245881053 0.49854245287694876

GOTERM_BP_ALL GO:0060627~regulation of vesicle-mediated transport 16 4.819277108433735 0.020658673835496993 RAB3B, SYT3, AHSG, GRIK5, LGI3, WNT5A, SNAP91, RIMS2, VTN, SV2B, VSNL1, LRRTM1, STAP1, ERC2, IL13RA2, SNCA 286 570 19554 1.9191755612808243 1.0 0.5139667516016003 0.5051506296463246

GOTERM_BP_ALL GO:0006836~neurotransmitter transport 7 2.108433734939759 0.021518214353946567 RIMS2, KCNJ10, SV2B, SLC6A15, SYN3, ERC2, SNCA 286 148 19554 3.233745983745984 1.0 0.5286172658271402 0.5195498421685589

GOTERM_BP_ALL GO:1902600~proton transmembrane transport 7 2.108433734939759 0.021518214353946567 SLC9A2, SLC9A3, SLC9A4, SLC15A1, SLC36A2, ATP6V1G3, ATP6V0D2 286 148 19554 3.233745983745984 1.0 0.5286172658271402 0.5195498421685589

GOTERM_BP_ALL GO:1902476~chloride transmembrane transport 6 1.8072289156626504 0.02175383558620124 GABRB2, GLRB, CLCNKB, SLC4A3, ANO5, SLC12A8 286 109 19554 3.763520882786938 1.0 0.5310655112481378 0.521956092596416

GOTERM_MF_ALL GO:0008509~monoatomic anion transmembrane transporter activity 7 2.108433734939759 0.021895733742516497 GABRB2, MCOLN3, GLRB, CLCNKB, SLC4A3, ANO5, SLC12A8 289 144 19144 3.2201076509034987 0.9999999912348696 0.29629871951566683 0.2814661256900911

GOTERM_BP_ALL GO:0015670~carbon dioxide transport 3 0.9036144578313252 0.022202336716024212 AQP6, RHCG, RHBG 286 16 19554 12.819493006993007 1.0 0.5386479951105004 0.5294085133715338

GOTERM_BP_ALL GO:0042886~amide transport 7 2.108433734939759 0.02280862750754926 RIMS2, SLC13A3, SLC15A1, GAL, MTTP, SLC38A3, CDH17 286 150 19554 3.1906293706293707 1.0 0.5499413521264656 0.5405081543301334

GOTERM_CC_ALL GO:0098794~postsynapse 18 5.421686746987952 0.022867937210253642 GABRB2, CHRNB4, DGKB, GRIK5, WNT5A, SYN3, GRM1, IGSF11, CPEB1, GLRB, LRRTM1, ADORA1, SLC16A7, NEFM, RGS7BP, DRD1, SPTBN2, SNCA 299 694 20791 1.8035044769789788 0.9999669511931333 0.20815028124103516 0.19134891324848524

GOTERM_CC_ALL GO:0009986~cell surface 23 6.927710843373494 0.022868528656526284 ST14, CR1, WNT5A, AQP4, VWDE, VTCN1, SLC4A3, L1CAM, PRLR, PSG9, SCNN1G, SLC9A3, EMCN, PSG4, CA4, ENPP1, ZPLD1, TEK, ENPP3, ADTRP, PROM1, IL13RA2, CDH17 299 969 20791 1.6504723346828611 0.9999669601137359 0.20815028124103516 0.19134891324848524

GOTERM_MF_ALL GO:0030414~peptidase inhibitor activity 8 2.4096385542168677 0.022976074592224255 PCSK1N, SLPI, AHSG, WFIKKN2, CST6, CST5, SERPINA4, KNG1 289 186 19144 2.849127506790192 0.9999999965281844 0.3059829616329548 0.2906655785714719

GOTERM_BP_ALL GO:0030324~lung development 8 2.4096385542168677 0.023349420347379555 HSD11B1, CCBE1, WNT11, IGFBP5, FGF9, WNT5A, FOXJ1, WNT2 286 193 19554 2.834015725207435 1.0 0.5577917166322883 0.5482238607658358

GOTERM_BP_ALL GO:0008610~lipid biosynthetic process 17 5.120481927710843 0.02341982629997319 HACD1, PLA2G4F, DGAT2, DGKB, ST8SIA2, PRLR, FA2H, ALDH1A3, STK33, CYP11A1, LIPH, INPP5J, PTGDS, ST6GALNAC3, ST6GALNAC5, PTGES, CES1 286 632 19554 1.8390833849694608 1.0 0.5577917166322883 0.5482238607658358

GOTERM_BP_ALL GO:0014074~response to purine-containing compound 7 2.108433734939759 0.02415014195968236 HCN4, IGFBP5, ADORA1, ENPP1, FOS, TEK, HCN2 286 152 19554 3.1486474052263524 1.0 0.571239667485356 0.5614411376027347

GOTERM_BP_ALL GO:0002890~negative regulation of immunoglobulin mediated immune response 3 0.9036144578313252 0.024923908058539414 CR2, CR1, FOXJ1 286 17 19554 12.065405183052242 1.0 0.571239667485356 0.5614411376027347

GOTERM_BP_ALL GO:0002713~negative regulation of B cell mediated immunity 3 0.9036144578313252 0.024923908058539414 CR2, CR1, FOXJ1 286 17 19554 12.065405183052242 1.0 0.571239667485356 0.5614411376027347

GOTERM_BP_ALL GO:0001574~ganglioside biosynthetic process 3 0.9036144578313252 0.024923908058539414 ST8SIA2, ST6GALNAC3, ST6GALNAC5 286 17 19554 12.065405183052242 1.0 0.571239667485356 0.5614411376027347

GOTERM_BP_ALL GO:0007218~neuropeptide signaling pathway 6 1.8072289156626504 0.02495547557094521 PCSK1N, GPR143, CALCA, GAL, GLRB, SSTR5 286 113 19554 3.630298904635188 1.0 0.571239667485356 0.5614411376027347

GOTERM_BP_ALL GO:0099565~chemical synaptic transmission, postsynaptic 5 1.5060240963855422 0.025008188207884252 CHRNB4, GLRB, GRIK5, ADORA1, SNCA 286 76 19554 4.498067721751933 1.0 0.571239667485356 0.5614411376027347

GOTERM_BP_ALL GO:0044458~motile cilium assembly 5 1.5060240963855422 0.025008188207884252 CFAP61, KLC3, MCIDAS, SPAG6, FOXJ1 286 76 19554 4.498067721751933 1.0 0.571239667485356 0.5614411376027347

GOTERM_BP_ALL GO:0030323~respiratory tube development 8 2.4096385542168677 0.02574678518640403 HSD11B1, CCBE1, WNT11, IGFBP5, FGF9, WNT5A, FOXJ1, WNT2 286 197 19554 2.7764722587057613 1.0 0.5846915287098496 0.5746622577360759

GOTERM_CC_ALL GO:0070382~exocytic vesicle 9 2.710843373493976 0.026264817127554897 RAB3B, SYT3, SV2B, LGI3, SYN3, ATP6V1G3, SYNGR3, SNAP91, SNCA 299 247 20791 2.5336682328409137 0.9999930076280182 0.23428216877778968 0.21537150044595013

GOTERM_CC_ALL GO:0030658~transport vesicle membrane 9 2.710843373493976 0.02688641280952981 RAB3B, SYT3, SV2B, SLC30A2, CA4, SYN3, ATP6V1G3, SYNGR3, SNCA 299 248 20791 2.5234518286762326 0.9999947405870827 0.23512431594216263 0.21614567160602396

GOTERM_BP_ALL GO:0099536~synaptic signaling 14 4.216867469879518 0.02696456785652503 GABRB2, CHRNB4, GRIK5, SYN3, CHRDL1, GRM1, RIMS2, SV2B, GLRB, ADORA1, DRD1, ERC2, PVALB, SNCA 286 487 19554 1.9654801051104953 1.0 0.5995651973918854 0.5892807969245899

GOTERM_BP_ALL GO:0099504~synaptic vesicle cycle 7 2.108433734939759 0.026989461723396446 RIMS2, SYN3, ATP6V1G3, ERC2, SNAP91, SH3GL2, SNCA 286 156 19554 3.067912856374395 1.0 0.5995651973918854 0.5892807969245899

GOTERM_BP_ALL GO:0050905~neuromuscular process 7 2.108433734939759 0.026989461723396446 ALDH1A3, UCHL1, ANKFN1, GLRB, DRD1, CTNNA2, HOXD10 286 156 19554 3.067912856374395 1.0 0.5995651973918854 0.5892807969245899

GOTERM_BP_ALL GO:0044703~multi-organism reproductive process 8 2.4096385542168677 0.02701573854095541 PSG9, HSD11B2, CALCA, IGFBP5, PSG4, FOS, SLC38A3, PRLR 286 199 19554 2.748568014899673 1.0 0.5995651973918854 0.5892807969245899

GOTERM_BP_ALL GO:0061061~muscle structure development 15 4.518072289156627 0.027545226987762822 POPDC3, IGFBP5, WNT5A, WFIKKN2, MYBPH, FOS, HOXD10, KY, FBXO40, UCHL1, DNER, NRAP, ANKRD2, B4GALNT2, WNT2 286 540 19554 1.8991841491841492 1.0 0.6078624667469016 0.5974357424069009

GOTERM_BP_ALL GO:0035672~oligopeptide transmembrane transport 3 0.9036144578313252 0.027773690805246568 SLC13A3, SLC15A1, CDH17 286 18 19554 11.395104895104895 1.0 0.6094608780072646 0.5990067359625931

GOTERM_BP_ALL GO:0032535~regulation of cellular component size 12 3.614457831325301 0.02799070487688093 RAB3B, CCL11, SCIN, ARHGAP40, CRACD, WNT5A, AQP4, SEMA3E, L1CAM, SLC12A8, KANK4, SPTBN2 286 389 19554 2.1091196721016776 1.0 0.6107915823971894 0.600314614649977

GOTERM_BP_ALL GO:0060031~mediolateral intercalation 2 0.6024096385542169 0.028938349276345688 WNT11, WNT5A 286 2 19554 68.37062937062936 1.0 0.6244927749911948 0.6137807893474646

GOTERM_BP_ALL GO:0060775~planar cell polarity pathway involved in gastrula mediolateral intercalation 2 0.6024096385542169 0.028938349276345688 WNT11, WNT5A 286 2 19554 68.37062937062936 1.0 0.6244927749911948 0.6137807893474646

GOTERM_BP_ALL GO:1990573~potassium ion import across plasma membrane 4 1.2048192771084338 0.029344348441477822 HCN4, KCNJ10, SLC12A8, HCN2 286 46 19554 5.945272119185163 1.0 0.6297748627055625 0.6189722728946888

GOTERM_MF_ALL GO:0005427~proton-dependent oligopeptide secondary active transmembrane transporter activity 2 0.6024096385542169 0.029862211735684045 SLC15A1, CDH17 289 2 19144 66.24221453287197 0.9999999999907443 0.38107389869756925 0.36199749375680895

GOTERM_MF_ALL GO:0003845~11-beta-hydroxysteroid dehydrogenase [NAD(P)+] activity 2 0.6024096385542169 0.029862211735684045 HSD11B1, HSD11B2 289 2 19144 66.24221453287197 0.9999999999907443 0.38107389869756925 0.36199749375680895

GOTERM_MF_ALL GO:1901681~sulfur compound binding 10 3.0120481927710845 0.02997720776405193 VTN, FGF9, HPSE2, LIPH, SMOC1, ENPP1, NDNF, BMP7, KNG1, PTGES 289 286 19144 2.3161613473032157 0.9999999999916196 0.38107389869756925 0.36199749375680895

GOTERM_BP_ALL GO:0006820~monoatomic anion transport 7 2.108433734939759 0.030041835235580197 GABRB2, MCOLN3, GLRB, CLCNKB, SLC4A3, ANO5, SLC12A8 286 160 19554 2.991215034965035 1.0 0.6377359153813927 0.6267967688553934

GOTERM_BP_ALL GO:0031960~response to corticosteroid 7 2.108433734939759 0.030041835235580197 SCNN1G, HSD11B2, GLB1, FOS, PTGDS, SSTR5, CPN1 286 160 19554 2.991215034965035 1.0 0.6377359153813927 0.6267967688553934

GOTERM_BP_ALL GO:0060039~pericardium development 3 0.9036144578313252 0.03074739729749305 WNT5A, BMP7, BMP5 286 19 19554 10.795362532204638 1.0 0.6459451215334199 0.6348651616914488

GOTERM_BP_ALL GO:1903530~regulation of secretion by cell 16 4.819277108433735 0.0308910757913001 RAB3B, PFKFB2, SYT3, CHRNB4, LGI3, NR0B2, SSTR5, RIMS2, GAL, SV2B, VSNL1, ADORA1, ADTRP, IL13RA2, PTGES, SNCA 286 601 19554 1.8201831446423793 1.0 0.6459451215334199 0.6348651616914488

GOTERM_BP_ALL GO:0060711~labyrinthine layer development 4 1.2048192771084338 0.03101663474875881 ST14, WNT2, BMP7, BMP5 286 47 19554 5.818776967713138 1.0 0.6459451215334199 0.6348651616914488

GOTERM_BP_ALL GO:0051346~negative regulation of hydrolase activity 8 2.4096385542168677 0.031093978653880354 VTN, CR1, AHSG, PPP4R4, CST5, SERPINA4, KNG1, SNCA 286 205 19554 2.6681221217806583 1.0 0.6459451215334199 0.6348651616914488

GOTERM_BP_ALL GO:0006821~chloride transport 6 1.8072289156626504 0.0312554091064558 GABRB2, GLRB, CLCNKB, SLC4A3, ANO5, SLC12A8 286 120 19554 3.418531468531469 1.0 0.6459451215334199 0.6348651616914488

GOTERM_CC_ALL GO:0098982~GABA-ergic synapse 5 1.5060240963855422 0.03145941629702108 GABRB2, GLRB, LRRTM1, DRD1, ERC2 299 83 20791 4.18886247330459 0.9999993564991131 0.26982499362445 0.24804539772651235

GOTERM_BP_ALL GO:0030182~neuron differentiation 26 7.83132530120482 0.031510319154964654 GABRB2, TENM3, ONECUT2, NDNF, SEMA3E, HOXD10, UCHL1, WNT11, CRTAC1, NCAM2, DRD1, CTNNA2, PROM1, WNT2, SH3GL2, MCOLN3, IRX6, SPAG6, ST8SIA2, WNT5A, L1CAM, BMP7, MYO16, NFASC, ADGRF1, CNTN3 286 1155 19554 1.5390791027154664 1.0 0.6477858243120628 0.6366742907153121

GOTERM_MF_ALL GO:0061135~endopeptidase regulator activity 8 2.4096385542168677 0.03162085994237525 PCSK1N, SLPI, AHSG, WFIKKN2, CST6, CST5, SERPINA4, KNG1 289 199 19144 2.6630035993114363 0.9999999999979766 0.39502147197954324 0.3752468571724624

GOTERM_BP_ALL GO:0017157~regulation of exocytosis 8 2.4096385542168677 0.03175355103628846 RIMS2, RAB3B, SYT3, VSNL1, SV2B, LGI3, IL13RA2, SNCA 286 206 19554 2.655170072645801 1.0 0.6485494239865799 0.6374247922899335

GOTERM_BP_ALL GO:0007420~brain development 19 5.72289156626506 0.0318795415784494 WNT5A, FOXJ1, NDNF, SEMA3E, SYNGR3, L1CAM, BMP7, MYO16, BMP5, ALDH1A3, NFASC, UCHL1, FGF9, PADI2, CNTN3, DRD1, CTNNA2, WNT2, SPTBN2 286 764 19554 1.7003166990077982 1.0 0.6485494239865799 0.6374247922899335

GOTERM_MF_ALL GO:0042578~phosphoric ester hydrolase activity 12 3.614457831325301 0.03201604302098801 PFKFB2, PLPP4, PDE6G, EYA4, PPM1H, INPP5J, ENPP1, ENPP3, NOTUM, DUSP15, DUSP9, PLCXD2 289 385 19144 2.0646924010245806 0.9999999999985628 0.39502147197954324 0.3752468571724624

GOTERM_CC_ALL GO:0098797~plasma membrane protein complex 19 5.72289156626506 0.03224252614163995 HCN4, DPP10, KCNG3, CHRNB4, KCNIP1, KCNIP3, GRIK5, LRRC52, GRM1, PSG9, VTN, KCNS1, GLRB, GJB3, DRD1, CTNNA2, ATP6V0D2, CDH17, HCN2 299 779 20791 1.6959784648013705 0.999999551384614 0.2713238992296494 0.2494233154353279

GOTERM_MF_ALL GO:0015370~solute:sodium symporter activity 5 1.5060240963855422 0.0325656545732554 SLC13A3, SLC6A15, SLC6A20, SLC38A3, SLC38A4 289 80 19144 4.140138408304498 0.999999999999107 0.3959794809704533 0.3761569086215153

GOTERM_BP_ALL GO:0016192~vesicle-mediated transport 30 9.036144578313253 0.032592705096248856 COLEC12, RAB3B, PIGR, CALCA, RIMS2, VTN, SCIN, LRRTM1, DNER, ADORA1, ENPP1, KIF1A, ERC2, ATP6V1G3, PTGDS, SH3GL2, SNCA, SPTBN2, SYT3, AHSG, GCGR, LGI3, SYN3, SYNGR3, SNAP91, EPN3, PLPP4, CLNK, XKR4, CES1 286 1390 19554 1.4756250943301303 1.0 0.6591474720947275 0.6478410510424114

GOTERM_BP_ALL GO:0021762~substantia nigra development 4 1.2048192771084338 0.03273799528581084 UCHL1, FGF9, PADI2, SYNGR3 286 48 19554 5.6975524475524475 1.0 0.6591474720947275 0.6478410510424114

GOTERM_BP_ALL GO:0044706~multi-multicellular organism process 8 2.4096385542168677 0.033122680664353796 PSG9, HSD11B2, CALCA, IGFBP5, PSG4, FOS, SLC38A3, PRLR 286 208 19554 2.629639591178053 1.0 0.6603014615616227 0.6489752460151229

GOTERM_BP_ALL GO:0032890~regulation of organic acid transport 5 1.5060240963855422 0.03313340667334308 ADORA1, SLC38A3, PTGES, CES1, SNCA 286 83 19554 4.118712612688517 1.0 0.6603014615616227 0.6489752460151229

GOTERM_BP_ALL GO:0001503~ossification 10 3.0120481927710845 0.03360235571601712 CALCA, WNT11, IGFBP5, FGF9, AHSG, ENPP1, TEK, CHRDL1, BMP7, BMP5 286 302 19554 2.2639281248552776 1.0 0.662873655029563 0.6515033183969514

GOTERM_CC_ALL GO:0048471~perinuclear region of cytoplasm 19 5.72289156626506 0.03372781947176621 HCN4, RAB3B, DGAT2, ST8SIA2, SLC2A12, TPD52L1, MYO16, EPN3, SCEL, STK33, KCNS1, GLB1, CA4, KIF1A, ENPP3, PTGDS, SH3GL2, PTGES, SNCA 299 783 20791 1.687314462426906 0.9999997738657387 0.2785668052668098 0.2560815922856323

GOTERM_BP_ALL GO:0002923~regulation of humoral immune response mediated by circulating immunoglobulin 3 0.9036144578313252 0.033840836848813546 CR2, CR1, FOXJ1 286 20 19554 10.255594405594405 1.0 0.662873655029563 0.6515033183969514

GOTERM_BP_ALL GO:0006857~oligopeptide transport 3 0.9036144578313252 0.033840836848813546 SLC13A3, SLC15A1, CDH17 286 20 19554 10.255594405594405 1.0 0.662873655029563 0.6515033183969514

GOTERM_BP_ALL GO:0006874~intracellular calcium ion homeostasis 8 2.4096385542168677 0.03394130338093 SLC24A2, CALCA, CCL11, SV2B, WNT5A, ADORA1, ERC2, GRM1 286 209 19554 2.617057583564761 1.0 0.662873655029563 0.6515033183969514

GOTERM_MF_ALL GO:0015108~chloride transmembrane transporter activity 6 1.8072289156626504 0.034026713074338744 GABRB2, GLRB, CLCNKB, SLC4A3, ANO5, SLC12A8 289 119 19144 3.3399435898927043 0.9999999999997483 0.40783446099100296 0.38741843314639973

GOTERM_BP_ALL GO:0099003~vesicle-mediated transport in synapse 7 2.108433734939759 0.035033779861428346 RIMS2, SYN3, ATP6V1G3, ERC2, SNAP91, SH3GL2, SNCA 286 166 19554 2.8830988288819617 1.0 0.680805692232533 0.6691277656120569

GOTERM_BP_ALL GO:0008544~epidermis development 11 3.313253012048193 0.035390167602648626 FA2H, SCEL, ST14, MCOLN3, CR2, IGFBP5, BNC1, WNT5A, CST6, FOXN1, KRT6A 286 354 19554 2.1245110821382007 1.0 0.6843267062175521 0.6725883832998419

GOTERM_MF_ALL GO:0015174~basic amino acid transmembrane transporter activity 3 0.9036144578313252 0.03585533424498785 SLC7A4, SLC38A3, SLC38A4 289 20 19144 9.936332179930796 0.9999999999999486 0.4236989497400677 0.40248875201768053

GOTERM_BP_ALL GO:0050807~regulation of synapse organization 9 2.710843373493976 0.036220729590140985 CAMK2B, DGKB, ST8SIA2, LRRTM1, WNT5A, LRRN1, KIF1A, CTNNA2, SNCA 286 258 19554 2.385021954789397 1.0 0.6969367969413335 0.6849821719041934

GOTERM_CC_ALL GO:0062023~collagen-containing extracellular matrix 13 3.91566265060241 0.03629205558575207 COLEC12, COL26A1, AHSG, WNT5A, L1CAM, BMP7, KNG1, VTN, IGFBPL1, ANGPTL7, SLPI, SMOC1, WNT2 299 465 20791 1.9439925198690977 0.9999999308701112 0.29429557802264406 0.27054077800287907

GOTERM_BP_ALL GO:0048699~generation of neurons 27 8.132530120481928 0.03671807912648112 GABRB2, TENM3, ONECUT2, NDNF, SEMA3E, HOXD10, UCHL1, WNT11, CRTAC1, DNER, NCAM2, DRD1, CTNNA2, PROM1, WNT2, SH3GL2, MCOLN3, IRX6, SPAG6, ST8SIA2, WNT5A, L1CAM, BMP7, MYO16, NFASC, ADGRF1, CNTN3 286 1231 19554 1.4995995069106363 1.0 0.6997432450076592 0.6877404806923716

GOTERM_BP_ALL GO:0071363~cellular response to growth factor stimulus 14 4.216867469879518 0.036915454591783504 CALCA, DOCK3, ONECUT2, WNT5A, WFIKKN2, NDNF, FOS, CHRDL1, BMP7, BMP5, PSG9, FGF9, WNT2, SH3GL2 286 510 19554 1.876840806252571 1.0 0.6997432450076592 0.6877404806923716

GOTERM_BP_ALL GO:0002921~negative regulation of humoral immune response 3 0.9036144578313252 0.03704991386224006 CR2, CR1, FOXJ1 286 21 19554 9.767232767232768 1.0 0.6997432450076592 0.6877404806923716

GOTERM_BP_ALL GO:0002683~negative regulation of immune system process 15 4.518072289156627 0.037083167362157055 CR2, CALCA, CR1, DAPL1, FOXJ1, VTCN1, BMP5, GAL, ADORA1, CLNK, PADI2, STAP1, ENPP3, ADTRP, IL13RA2 286 563 19554 1.8215975853631272 1.0 0.6997432450076592 0.6877404806923716

GOTERM_BP_ALL GO:0030278~regulation of ossification 6 1.8072289156626504 0.037383113862076864 CALCA, AHSG, WNT5A, ENPP1, NOTUM, BMP7 286 126 19554 3.255744255744256 1.0 0.702011743967655 0.6899700678678514

GOTERM_BP_ALL GO:0032102~negative regulation of response to external stimulus 13 3.91566265060241 0.03802097616759871 CR1, WNT5A, SEMA3E, KNG1, VTN, ADORA1, PADI2, STAP1, TEK, ENPP3, DRD1, CTNNA2, ADTRP 286 460 19554 1.932213438735178 1.0 0.710573841677706 0.6983852990785236

GOTERM_MF_ALL GO:0005200~structural constituent of cytoskeleton 6 1.8072289156626504 0.03839012996880006 EPB41L4B, BFSP2, NEFM, CTNNA2, SPTBN2, KRT6A 289 123 19144 3.2313275381888764 0.9999999999999943 0.4473516533864341 0.424957410904634

GOTERM_BP_ALL GO:0007416~synapse assembly 6 1.8072289156626504 0.038470984859212104 GABRB2, ADGRF1, DNER, WNT5A, DRD1, SPTBN2 286 127 19554 3.2301084742029627 1.0 0.7153267924306237 0.7030567220023463

GOTERM_CC_ALL GO:0098862~cluster of actin-based cell projections 7 2.108433734939759 0.03847144570655962 SLC9A3, SLC15A1, SCIN, MTTP, CA4, ITLN1, TRPM6 299 173 20791 2.8135596496993833 0.9999999748154083 0.3063975854486713 0.2816659417801687

GOTERM_BP_ALL GO:0010976~positive regulation of neuron projection development 7 2.108433734939759 0.038641565080097695 CAMK2B, TENM3, WNT5A, NDNF, BMP7, TRIM67, BMP5 286 170 19554 2.8152612093788565 1.0 0.7153267924306237 0.7030567220023463

GOTERM_MF_ALL GO:0015103~inorganic anion transmembrane transporter activity 7 2.108433734939759 0.039021340858025506 GABRB2, GLRB, CLCNKB, AQP6, SLC4A3, ANO5, SLC12A8 289 165 19144 2.810275768061235 0.9999999999999968 0.4484781504093617 0.4260275159431004

GOTERM_BP_ALL GO:0001516~prostaglandin biosynthetic process 3 0.9036144578313252 0.04037062594432195 PLA2G4F, PTGDS, PTGES 286 22 19554 9.32326764144946 1.0 0.7300354858264885 0.7175131157419072

GOTERM_BP_ALL GO:0060292~long-term synaptic depression 3 0.9036144578313252 0.04037062594432195 SLC24A2, ADORA1, DRD1 286 22 19554 9.32326764144946 1.0 0.7300354858264885 0.7175131157419072

GOTERM_BP_ALL GO:0060713~labyrinthine layer morphogenesis 3 0.9036144578313252 0.04037062594432195 ST14, BMP7, BMP5 286 22 19554 9.32326764144946 1.0 0.7300354858264885 0.7175131157419072

GOTERM_BP_ALL GO:0015802~basic amino acid transport 3 0.9036144578313252 0.04037062594432195 SLC7A4, SLC38A3, SLC38A4 286 22 19554 9.32326764144946 1.0 0.7300354858264885 0.7175131157419072

GOTERM_BP_ALL GO:0046457~prostanoid biosynthetic process 3 0.9036144578313252 0.04037062594432195 PLA2G4F, PTGDS, PTGES 286 22 19554 9.32326764144946 1.0 0.7300354858264885 0.7175131157419072

GOTERM_BP_ALL GO:0050803~regulation of synapse structure or activity 9 2.710843373493976 0.04101982048133411 CAMK2B, DGKB, ST8SIA2, LRRTM1, WNT5A, LRRN1, KIF1A, CTNNA2, SNCA 286 265 19554 2.322021374851564 1.0 0.738356768664014 0.7256916628011136

GOTERM_MF_ALL GO:0008081~phosphoric diester hydrolase activity 5 1.5060240963855422 0.04230115858833882 PDE6G, ENPP1, ENPP3, NOTUM, PLCXD2 289 87 19144 3.8070238237282745 0.9999999999999998 0.47960367642724694 0.4555949107419735

GOTERM_BP_ALL GO:0022008~neurogenesis 30 9.036144578313253 0.042377434960888166 GABRB2, TENM3, ONECUT2, NDNF, SEMA3E, HOXD10, VTN, UCHL1, WNT11, CRTAC1, DNER, NCAM2, DRD1, CTNNA2, PROM1, WNT2, SH3GL2, MCOLN3, KCNJ10, IRX6, SPAG6, ST8SIA2, WNT5A, L1CAM, BMP7, MYO16, FA2H, NFASC, ADGRF1, CNTN3 286 1421 19554 1.4434334138767637 1.0 0.7456806072765486 0.7328898748936687

GOTERM_BP_ALL GO:0030879~mammary gland development 6 1.8072289156626504 0.043014847516620214 HOXB9, CCL11, IGFBP5, WNT5A, WNT2, PRLR 286 131 19554 3.131479207815086 1.0 0.7456806072765486 0.7328898748936687

GOTERM_BP_ALL GO:0003138~primary heart field specification 2 0.6024096385542169 0.04309304293704747 WNT11, WNT5A 286 3 19554 45.58041958041958 1.0 0.7456806072765486 0.7328898748936687

GOTERM_BP_ALL GO:0021502~neural fold elevation formation 2 0.6024096385542169 0.04309304293704747 BMP7, BMP5 286 3 19554 45.58041958041958 1.0 0.7456806072765486 0.7328898748936687

GOTERM_BP_ALL GO:0001661~conditioned taste aversion 2 0.6024096385542169 0.04309304293704747 FOS, DRD1 286 3 19554 45.58041958041958 1.0 0.7456806072765486 0.7328898748936687

GOTERM_BP_ALL GO:0072139~glomerular parietal epithelial cell differentiation 2 0.6024096385542169 0.04309304293704747 FOXJ1, PROM1 286 3 19554 45.58041958041958 1.0 0.7456806072765486 0.7328898748936687

GOTERM_CC_ALL GO:0042734~presynaptic membrane 7 2.108433734939759 0.04317732783823816 RIMS2, GRIK5, ADORA1, RGS7BP, DRD1, ERC2, SNAP91 299 178 20791 2.734527075269625 0.9999999971762253 0.3301746696158031 0.30352379942259927

GOTERM_BP_ALL GO:0072330~monocarboxylic acid biosynthetic process 7 2.108433734939759 0.04347011252686528 FA2H, ALDH1A3, HACD1, PLA2G4F, UST, PTGDS, PTGES 286 175 19554 2.734825174825175 1.0 0.7456806072765486 0.7328898748936687

GOTERM_BP_ALL GO:0010975~regulation of neuron projection development 13 3.91566265060241 0.043631983106762764 CAMK2B, TENM3, WNT5A, NDNF, SEMA3E, L1CAM, BMP7, BMP5, UST, INPP5J, KIF1A, CTNNA2, TRIM67 286 470 19554 1.89110251450677 1.0 0.7456806072765486 0.7328898748936687

GOTERM_BP_ALL GO:0034765~regulation of monoatomic ion transmembrane transport 11 3.313253012048193 0.04365094551115454 DPP10, CALCA, KCNJ10, GAL, KCNIP1, KCNS1, KCNIP3, DRD1, LRRC52, RGS7, SNCA 286 367 19554 2.049255921190526 1.0 0.7456806072765486 0.7328898748936687

GOTERM_BP_ALL GO:0031338~regulation of vesicle fusion 3 0.9036144578313252 0.043799062091752085 GRIK5, ERC2, SNCA 286 23 19554 8.917908178777745 1.0 0.7456806072765486 0.7328898748936687

GOTERM_BP_ALL GO:0015669~gas transport 3 0.9036144578313252 0.043799062091752085 AQP6, RHCG, RHBG 286 23 19554 8.917908178777745 1.0 0.7456806072765486 0.7328898748936687

GOTERM_BP_ALL GO:0060004~reflex 3 0.9036144578313252 0.043799062091752085 ALDH1A3, GLRB, ZPLD1 286 23 19554 8.917908178777745 1.0 0.7456806072765486 0.7328898748936687

GOTERM_BP_ALL GO:0051591~response to cAMP 5 1.5060240963855422 0.044099390752914164 HCN4, IGFBP5, FOS, TEK, HCN2 286 91 19554 3.756627987397218 1.0 0.7456806072765486 0.7328898748936687

GOTERM_BP_ALL GO:0007631~feeding behavior 5 1.5060240963855422 0.044099390752914164 UCHL1, CALCA, GAL, FOS, DRD1 286 91 19554 3.756627987397218 1.0 0.7456806072765486 0.7328898748936687

GOTERM_MF_ALL GO:0004667~prostaglandin-D synthase activity 2 0.6024096385542169 0.0444583711220305 PTGDS, PTGES 289 3 19144 44.161476355247984 1.0 0.4973409782851146 0.47244429045677744

GOTERM_CC_ALL GO:0008021~synaptic vesicle 8 2.4096385542168677 0.04506408628647146 RAB3B, SV2B, LGI3, SYN3, ATP6V1G3, SYNGR3, SNAP91, SNCA 299 226 20791 2.4614200728090685 0.9999999988291395 0.3301746696158031 0.30352379942259927

GOTERM_CC_ALL GO:0099501~exocytic vesicle membrane 6 1.8072289156626504 0.04515841893848428 RAB3B, SV2B, SYN3, ATP6V1G3, SYNGR3, SNCA 299 135 20791 3.0904496469713862 0.9999999988796076 0.3301746696158031 0.30352379942259927

GOTERM_CC_ALL GO:0099634~postsynaptic specialization membrane 6 1.8072289156626504 0.04515841893848428 GABRB2, GRIK5, LRRTM1, SLC16A7, RGS7BP, GRM1 299 135 20791 3.0904496469713862 0.9999999988796076 0.3301746696158031 0.30352379942259927

GOTERM_CC_ALL GO:0030672~synaptic vesicle membrane 6 1.8072289156626504 0.04515841893848428 RAB3B, SV2B, SYN3, ATP6V1G3, SYNGR3, SNCA 299 135 20791 3.0904496469713862 0.9999999988796076 0.3301746696158031 0.30352379942259927

GOTERM_BP_ALL GO:0003206~cardiac chamber morphogenesis 6 1.8072289156626504 0.045402818693654146 WNT11, WNT5A, TEK, WNT2, BMP7, BMP5 286 133 19554 3.084389294915611 1.0 0.7611169892997195 0.7480614751463448

GOTERM_BP_ALL GO:0030258~lipid modification 7 2.108433734939759 0.045470358464762925 FA2H, ADH4, PLPP4, DGKB, INPP5J, HAO1, B4GALNT2 286 177 19554 2.703923195448619 1.0 0.7611169892997195 0.7480614751463448

GOTERM_BP_ALL GO:0051453~regulation of intracellular pH 5 1.5060240963855422 0.0455968703267113 SLC9A2, SLC9A3, SLC9A4, SLC4A3, ATP6V0D2 286 92 19554 3.7157950744907273 1.0 0.7611169892997195 0.7480614751463448

GOTERM_BP_ALL GO:0050878~regulation of body fluid levels 11 3.313253012048193 0.046113890829980006 SCNN1G, VTN, HSD11B2, DGKB, ADORA1, AQP6, AQP4, ADTRP, PRLR, KNG1, F5 286 371 19554 2.0271615177275555 1.0 0.766471734391072 0.7533243697714606

GOTERM_BP_ALL GO:0042311~vasodilation 4 1.2048192771084338 0.04824606468574881 CALCA, ADORA1, DRD1, KNG1 286 56 19554 4.883616383616384 1.0 0.7951440027955057 0.7815048199518552

GOTERM_BP_ALL GO:0072132~mesenchyme morphogenesis 4 1.2048192771084338 0.04824606468574881 WNT11, WNT5A, BMP7, BMP5 286 56 19554 4.883616383616384 1.0 0.7951440027955057 0.7815048199518552

GOTERM_BP_ALL GO:0002009~morphogenesis of an epithelium 13 3.91566265060241 0.04845269408245209 ST14, CCL11, IGFBP5, WNT5A, SEMA3E, FOXN1, BMP7, BMP5, ALDH1A3, WNT11, PODXL, CA9, KRT6A 286 478 19554 1.8594522632179538 1.0 0.7951942146473019 0.781554170514847

GOTERM_BP_ALL GO:0050673~epithelial cell proliferation 7 2.108433734939759 0.048672144429426245 CALCA, IGFBP5, FGF9, FABP7, WNT5A, TEK, WNT2 286 180 19554 2.658857808857809 1.0 0.7954535403403302 0.781809047968901

GOTERM_BP_ALL GO:0046683~response to organophosphorus 6 1.8072289156626504 0.04913056391156783 HCN4, IGFBP5, ENPP1, FOS, TEK, HCN2 286 136 19554 3.0163512957630605 1.0 0.7995999276607664 0.7858843119021204

GOTERM_MF_ALL GO:1904315~transmitter-gated monoatomic ion channel activity involved in regulation of postsynaptic membrane potential 4 1.2048192771084338 0.049857199066617945 GABRB2, CHRNB4, GLRB, GRIK5 289 55 19144 4.817615602390689 1.0 0.5457755258210575 0.5184542241708973

GOTERM_MF_ALL GO:0030552~cAMP binding 3 0.9036144578313252 0.05008905302529371 HCN4, POPDC3, HCN2 289 24 19144 8.280276816608996 1.0 0.5457755258210575 0.5184542241708973

GOTERM_BP_ALL GO:0071407~cellular response to organic cyclic compound 13 3.91566265060241 0.05059261005184404 HCN4, COLEC12, GABRB2, PLA2G4F, IGFBP5, SSTR5, SCNN1G, PADI2, UGT3A2, DRD1, ADTRP, HCN2, CES1 286 482 19554 1.8440211241041116 1.0 0.8199781529564432 0.8059129875063455

GOTERM_BP_ALL GO:0051384~response to glucocorticoid 6 1.8072289156626504 0.05171316378489185 HSD11B2, GLB1, FOS, PTGDS, SSTR5, CPN1 286 138 19554 2.9726360595925816 1.0 0.8334900229310426 0.8191930870538332

GOTERM_BP_ALL GO:1904062~regulation of monoatomic cation transmembrane transport 10 3.0120481927710845 0.05185306594271463 DPP10, CALCA, GAL, KCNIP1, KCNS1, KCNIP3, DRD1, LRRC52, RGS7, SNCA 286 328 19554 2.0844704076411396 1.0 0.8334900229310426 0.8191930870538332

GOTERM_BP_ALL GO:0046513~ceramide biosynthetic process 4 1.2048192771084338 0.05259398531280007 FA2H, ST8SIA2, ST6GALNAC3, ST6GALNAC5 286 58 19554 4.7152158186640944 1.0 0.8419348632450699 0.8274930721960635

GOTERM_BP_ALL GO:0060322~head development 19 5.72289156626506 0.053022513164590154 WNT5A, FOXJ1, NDNF, SEMA3E, SYNGR3, L1CAM, BMP7, MYO16, BMP5, ALDH1A3, NFASC, UCHL1, FGF9, PADI2, CNTN3, DRD1, CTNNA2, WNT2, SPTBN2 286 814 19554 1.5958746413291869 1.0 0.845330352738323 0.8308303185259657

GOTERM_BP_ALL GO:0030641~regulation of cellular pH 5 1.5060240963855422 0.05350531870138346 SLC9A2, SLC9A3, SLC9A4, SLC4A3, ATP6V0D2 286 97 19554 3.5242592459087305 1.0 0.8495600603561131 0.8349874735553297

GOTERM_BP_ALL GO:0048666~neuron development 21 6.325301204819277 0.054028595715434585 GABRB2, TENM3, ONECUT2, SPAG6, ST8SIA2, WNT5A, NDNF, SEMA3E, L1CAM, HOXD10, BMP7, MYO16, NFASC, UCHL1, ADGRF1, CRTAC1, CNTN3, NCAM2, DRD1, CTNNA2, SH3GL2 286 930 19554 1.5438529212722762 1.0 0.8537329418740965 0.8390887772285347

GOTERM_BP_ALL GO:0048593~camera-type eye morphogenesis 6 1.8072289156626504 0.05437381832115424 ALDH1A3, TENM3, WNT5A, PROM1, WNT2, BMP7 286 140 19554 2.9301698301698305 1.0 0.8537329418740965 0.8390887772285347

GOTERM_MF_ALL GO:0099529~neurotransmitter receptor activity involved in regulation of postsynaptic membrane potential 4 1.2048192771084338 0.054420202121586485 GABRB2, CHRNB4, GLRB, GRIK5 289 57 19144 4.648576458447156 1.0 0.5853660202565522 0.5560628344987747

GOTERM_BP_ALL GO:0048596~embryonic camera-type eye morphogenesis 3 0.9036144578313252 0.05469293806850159 ALDH1A3, WNT5A, BMP7 286 26 19554 7.888918773534159 1.0 0.8537329418740965 0.8390887772285347

GOTERM_BP_ALL GO:0060669~embryonic placenta morphogenesis 3 0.9036144578313252 0.05469293806850159 ST14, BMP7, BMP5 286 26 19554 7.888918773534159 1.0 0.8537329418740965 0.8390887772285347

GOTERM_BP_ALL GO:0070848~response to growth factor 14 4.216867469879518 0.054860975015462936 CALCA, DOCK3, ONECUT2, WNT5A, WFIKKN2, NDNF, FOS, CHRDL1, BMP7, BMP5, PSG9, FGF9, WNT2, SH3GL2 286 543 19554 1.762778657806282 1.0 0.8537329418740965 0.8390887772285347

GOTERM_BP_ALL GO:0051262~protein tetramerization 5 1.5060240963855422 0.05517084436864088 ALDH1A3, AQP4, MAT1A, TRPM6, SNCA 286 98 19554 3.488297416868846 1.0 0.8551480877139336 0.8404796489333822

GOTERM_CC_ALL GO:0098858~actin-based cell projection 8 2.4096385542168677 0.055664947545254015 SLC7A8, PODXL, MTTP, CA9, TEK, PROM1, MUC20, SLC38A4 299 238 20791 2.3373148590539894 0.9999999999919417 0.39655499021786483 0.3645460672406381

GOTERM_CC_ALL GO:0098855~HCN channel complex 2 0.6024096385542169 0.056115566381237346 HCN4, HCN2 299 4 20791 34.76755852842809 0.9999999999934868 0.39655499021786483 0.3645460672406381

GOTERM_CC_ALL GO:0098978~glutamatergic synapse 12 3.614457831325301 0.056904751959514235 DGKB, LRRTM1, WNT5A, PPM1H, SLC16A7, RGS7BP, DRD1, SYN3, ERC2, SH3GL2, GRM1, SPTBN2 299 445 20791 1.8751042801848858 0.9999999999955148 0.39655499021786483 0.3645460672406381

GOTERM_BP_ALL GO:1904273~L-alanine import across plasma membrane 2 0.6024096385542169 0.05704212394349504 SLC7A8, SLC38A4 286 4 19554 34.18531468531468 1.0 0.8648321196383262 0.8499975697110943

GOTERM_BP_ALL GO:1905069~allantois development 2 0.6024096385542169 0.05704212394349504 BMP7, BMP5 286 4 19554 34.18531468531468 1.0 0.8648321196383262 0.8499975697110943

GOTERM_BP_ALL GO:0035378~carbon dioxide transmembrane transport 2 0.6024096385542169 0.05704212394349504 RHCG, RHBG 286 4 19554 34.18531468531468 1.0 0.8648321196383262 0.8499975697110943

GOTERM_BP_ALL GO:0060686~negative regulation of prostatic bud formation 2 0.6024096385542169 0.05704212394349504 WNT5A, BMP7 286 4 19554 34.18531468531468 1.0 0.8648321196383262 0.8499975697110943

GOTERM_BP_ALL GO:0007223~Wnt signaling pathway, calcium modulating pathway 2 0.6024096385542169 0.05704212394349504 WNT11, WNT5A 286 4 19554 34.18531468531468 1.0 0.8648321196383262 0.8499975697110943

GOTERM_BP_ALL GO:0006939~smooth muscle contraction 4 1.2048192771084338 0.05712408777949005 CHRNB4, CALCA, PTGER3, DRD1 286 60 19554 4.558041958041958 1.0 0.8648321196383262 0.8499975697110943

GOTERM_MF_ALL GO:0070696~transmembrane receptor protein serine/threonine kinase binding 3 0.9036144578313252 0.05784518651891951 VWDE, BMP7, BMP5 289 26 19144 7.643332446100612 1.0 0.6143305251819426 0.5835773880453019

GOTERM_BP_ALL GO:0002053~positive regulation of mesenchymal cell proliferation 3 0.9036144578313252 0.058514925817393315 FGF9, WNT5A, WNT2 286 27 19554 7.596736596736597 1.0 0.8790742317028396 0.8639953854345113

GOTERM_BP_ALL GO:0048169~regulation of long-term neuronal synaptic plasticity 3 0.9036144578313252 0.058514925817393315 CAMK2B, KCNJ10, SNCA 286 27 19554 7.596736596736597 1.0 0.8790742317028396 0.8639953854345113

GOTERM_BP_ALL GO:0006631~fatty acid metabolic process 10 3.0120481927710845 0.0587519480789153 FA2H, ADH4, HACD1, PLA2G4F, HAO1, ADTRP, PTGDS, PTGES, CES1, SNCA 286 337 19554 2.028802058475649 1.0 0.879253291939629 0.8641713742335473

GOTERM_MF_ALL GO:0035379~carbon dioxide transmembrane transporter activity 2 0.6024096385542169 0.058835675813509396 RHCG, RHBG 289 4 19144 33.121107266435985 1.0 0.6170391500941799 0.5861504202920874

GOTERM_BP_ALL GO:0033993~response to lipid 20 6.024096385542169 0.060291612550617615 DGAT2, WNT5A, FOS, BMP7, SSTR5, CPN1, SCNN1G, HSD11B2, WNT11, SLPI, GLB1, GJB3, PADI2, STAP1, CA9, ADTRP, PTGDS, WNT2, CES1, SNCA 286 885 19554 1.5450989688277823 1.0 0.8988512924531008 0.8834332083275611

GOTERM_MF_ALL GO:0005253~monoatomic anion channel activity 5 1.5060240963855422 0.060563329451434236 GABRB2, MCOLN3, GLRB, CLCNKB, ANO5 289 98 19144 3.3797048231057127 1.0 0.6273164618488065 0.5959132539850999

GOTERM_BP_ALL GO:0051050~positive regulation of transport 20 6.024096385542169 0.060882116085560906 RAB3B, PFKFB2, AHSG, WNT5A, ITLN1, SYNGR3, NR0B2, LRRC52, VTN, SCIN, GAL, VSNL1, ADORA1, STAP1, DRD1, SLC38A3, PTGES, CES1, RGS7, SNCA 286 887 19554 1.5416150929115982 1.0 0.8992096583168563 0.8837854271066081

GOTERM_BP_ALL GO:0046394~carboxylic acid biosynthetic process 9 2.710843373493976 0.06091264584784056 FA2H, ALDH1A3, HACD1, PLA2G4F, PSAT1, UST, HAO1, PTGDS, PTGES 286 288 19554 2.136582167832168 1.0 0.8992096583168563 0.8837854271066081

GOTERM_BP_ALL GO:0032787~monocarboxylic acid metabolic process 14 4.216867469879518 0.061006287622623376 PFKFB2, HACD1, PLA2G4F, SCPEP1, FA2H, ALDH1A3, ADH4, UST, HAO1, ADTRP, PTGDS, PTGES, CES1, SNCA 286 551 19554 1.737184775297298 1.0 0.8992096583168563 0.8837854271066081

GOTERM_MF_ALL GO:0030276~clathrin binding 4 1.2048192771084338 0.061624656117445546 EPN3, SYT3, DNER, SNAP91 289 60 19144 4.416147635524798 1.0 0.6305254449089855 0.5989615966537085

GOTERM_BP_ALL GO:0048545~response to steroid hormone 9 2.710843373493976 0.06198651852155003 SCNN1G, HSD11B2, GLB1, PADI2, FOS, ADTRP, PTGDS, SSTR5, CPN1 286 289 19554 2.129189149950396 1.0 0.9098413632290954 0.8942347653447253

GOTERM_BP_ALL GO:0019755~one-carbon compound transport 3 0.9036144578313252 0.062426391537480176 AQP6, RHCG, RHBG 286 28 19554 7.325424575424576 1.0 0.9098413632290954 0.8942347653447253

GOTERM_BP_ALL GO:0072311~glomerular epithelial cell differentiation 3 0.9036144578313252 0.062426391537480176 PODXL, FOXJ1, PROM1 286 28 19554 7.325424575424576 1.0 0.9098413632290954 0.8942347653447253

GOTERM_BP_ALL GO:0055074~calcium ion homeostasis 8 2.4096385542168677 0.06279880849188803 SLC24A2, CALCA, CCL11, SV2B, WNT5A, ADORA1, ERC2, GRM1 286 240 19554 2.279020979020979 1.0 0.9118667136405749 0.896225374722521

GOTERM_BP_ALL GO:0098609~cell-cell adhesion 15 4.518072289156627 0.06365160568717346 CALCA, TENM3, VWDE, L1CAM, BMP7, BMP5, IGSF11, NFASC, EMCN, PSG4, NCAM2, PKP3, CTNNA2, CDH17, TJP3 286 608 19554 1.6867753956569747 1.0 0.9125417468047239 0.8968888289767881

GOTERM_BP_ALL GO:0016053~organic acid biosynthetic process 9 2.710843373493976 0.0640035245295747 FA2H, ALDH1A3, HACD1, PLA2G4F, PSAT1, UST, HAO1, PTGDS, PTGES 286 291 19554 2.1145555475452382 1.0 0.9125417468047239 0.8968888289767881

GOTERM_BP_ALL GO:0061180~mammary gland epithelium development 4 1.2048192771084338 0.06425288963721637 CCL11, WNT5A, WNT2, PRLR 286 63 19554 4.340992340992341 1.0 0.9125417468047239 0.8968888289767881

GOTERM_BP_ALL GO:0019216~regulation of lipid metabolic process 10 3.0120481927710845 0.06442654185083121 GPRC6A, ADGRF1, GAL, ASXL3, ADORA1, THRSP, MALRD1, CES1, BMP5, SNCA 286 343 19554 1.9933128096393402 1.0 0.9125417468047239 0.8968888289767881

GOTERM_BP_ALL GO:0070664~negative regulation of leukocyte proliferation 5 1.5060240963855422 0.06574408380436934 CR1, GAL, FOXJ1, ENPP3, VTCN1 286 104 19554 3.287049488972566 1.0 0.9125417468047239 0.8968888289767881

GOTERM_BP_ALL GO:0007423~sensory organ development 15 4.518072289156627 0.06578788324743223 GABRB2, MCOLN3, TENM3, WNT5A, EYA4, CHRDL1, BMP7, BMP5, ALDH1A3, BFSP2, ANGPTL7, FGF9, SMOC1, PROM1, WNT2 286 613 19554 1.6730170319077333 1.0 0.9125417468047239 0.8968888289767881

GOTERM_BP_ALL GO:0060560~developmental growth involved in morphogenesis 6 1.8072289156626504 0.06579628391785407 CCL11, WNT11, SPAG6, ST8SIA2, WNT5A, SH3GL2 286 148 19554 2.771782271782272 1.0 0.9125417468047239 0.8968888289767881

GOTERM_BP_ALL GO:0072006~nephron development 6 1.8072289156626504 0.06579628391785407 WNT11, PODXL, FOXJ1, TEK, PROM1, BMP7 286 148 19554 2.771782271782272 1.0 0.9125417468047239 0.8968888289767881

GOTERM_MF_ALL GO:0008201~heparin binding 7 2.108433734939759 0.06589894638839472 VTN, FGF9, LIPH, SMOC1, NDNF, BMP7, KNG1 289 188 19144 2.466465434734595 1.0 0.6590688871332235 0.6260761657272695

GOTERM_MF_ALL GO:0005044~scavenger receptor activity 3 0.9036144578313252 0.06598544281190796 COLEC12, VTN, ENPP1 289 28 19144 7.097380128521997 1.0 0.6590688871332235 0.6260761657272695

GOTERM_BP_ALL GO:0048468~cell development 44 13.253012048192772 0.06616523171367542 CFAP61, GABRB2, TENM3, ONECUT2, WFIKKN2, NDNF, SEMA3E, TFCP2L1, HOXD10, UCHL1, KLC3, CRTAC1, PODXL, DNER, SOHLH2, NRAP, NCAM2, DRD1, CTNNA2, SH3GL2, CR2, ST14, KCNJ10, IRX6, SPAG6, ST8SIA2, WNT5A, MYBPH, FOXJ1, FOS, L1CAM, FOXN1, BMP7, MYO16, BMP5, FA2H, NFASC, SLC9A4, BFSP2, ADGRF1, CNTN3, TEK, CDH17, TJP3 286 2344 19554 1.2834077185613024 1.0 0.9125417468047239 0.8968888289767881

GOTERM_BP_ALL GO:1901698~response to nitrogen compound 23 6.927710843373494 0.0662690632626877 HCN4, COLEC12, GABRB2, CHRNB4, IGFBP5, ST8SIA2, GCGR, FOS, BMP7, SCNN1G, HSD11B2, CPEB1, GAL, CYP11A1, ADORA1, MAN1C1, ENPP1, TEK, DRD1, ADTRP, HCN2, CES1, RGS7 286 1069 19554 1.4710238311735038 1.0 0.9125417468047239 0.8968888289767881

GOTERM_BP_ALL GO:0002068~glandular epithelial cell development 3 0.9036144578313252 0.06642393588928128 SLC9A4, WNT5A, BMP5 286 29 19554 7.072823727996142 1.0 0.9125417468047239 0.8968888289767881

GOTERM_BP_ALL GO:0003156~regulation of animal organ formation 3 0.9036144578313252 0.06642393588928128 WNT5A, WNT2, BMP7 286 29 19554 7.072823727996142 1.0 0.9125417468047239 0.8968888289767881

GOTERM_BP_ALL GO:0001573~ganglioside metabolic process 3 0.9036144578313252 0.06642393588928128 ST8SIA2, ST6GALNAC3, ST6GALNAC5 286 29 19554 7.072823727996142 1.0 0.9125417468047239 0.8968888289767881

GOTERM_BP_ALL GO:0002040~sprouting angiogenesis 4 1.2048192771084338 0.06671619234987156 CCBE1, SEMA3E, TEK, ADTRP 286 64 19554 4.273164335664335 1.0 0.9125417468047239 0.8968888289767881

GOTERM_BP_ALL GO:0030833~regulation of actin filament polymerization 6 1.8072289156626504 0.06731153399470478 CCL11, SCIN, ARHGAP40, CRACD, KANK4, SPTBN2 286 149 19554 2.7531797061998406 1.0 0.9125417468047239 0.8968888289767881

GOTERM_BP_ALL GO:0010631~epithelial cell migration 5 1.5060240963855422 0.06760197949384286 CCBE1, CALCA, WNT5A, ADTRP, PRSS3 286 105 19554 3.255744255744256 1.0 0.9125417468047239 0.8968888289767881

GOTERM_BP_ALL GO:0010243~response to organonitrogen compound 21 6.325301204819277 0.06798206792291825 HCN4, GABRB2, IGFBP5, ST8SIA2, GCGR, FOS, BMP7, SCNN1G, HSD11B2, CPEB1, GAL, CYP11A1, ADORA1, MAN1C1, ENPP1, TEK, DRD1, ADTRP, HCN2, CES1, RGS7 286 958 19554 1.4987298713812283 1.0 0.9125417468047239 0.8968888289767881

GOTERM_BP_ALL GO:0034762~regulation of transmembrane transport 13 3.91566265060241 0.06884345058619652 DPP10, CALCA, KCNJ10, KCNIP1, KCNIP3, ITLN1, LRRC52, GAL, KCNS1, ENPP1, DRD1, RGS7, SNCA 286 507 19554 1.7530930607853685 1.0 0.9125417468047239 0.8968888289767881

GOTERM_BP_ALL GO:0001654~eye development 11 3.313253012048193 0.06906100239707202 ALDH1A3, BFSP2, TENM3, ANGPTL7, FGF9, SMOC1, WNT5A, PROM1, WNT2, CHRDL1, BMP7 286 400 19554 1.880192307692308 1.0 0.9125417468047239 0.8968888289767881

GOTERM_BP_ALL GO:0043434~response to peptide hormone 11 3.313253012048193 0.06906100239707202 SCNN1G, HSD11B2, CPEB1, GAL, IGFBP5, CYP11A1, GCGR, ENPP1, FOS, TEK, BMP7 286 400 19554 1.880192307692308 1.0 0.9125417468047239 0.8968888289767881

GOTERM_BP_ALL GO:0045744~negative regulation of G protein-coupled receptor signaling pathway 4 1.2048192771084338 0.06922216529095872 CALCA, PADI2, RGS7, SNCA 286 65 19554 4.207423345884885 1.0 0.9125417468047239 0.8968888289767881

GOTERM_BP_ALL GO:0032835~glomerulus development 4 1.2048192771084338 0.06922216529095872 PODXL, FOXJ1, TEK, PROM1 286 65 19554 4.207423345884885 1.0 0.9125417468047239 0.8968888289767881

GOTERM_BP_ALL GO:0120035~regulation of plasma membrane bounded cell projection organization 16 4.819277108433735 0.06980552422411025 CAMK2B, TENM3, WNT5A, NDNF, SEMA3E, L1CAM, BMP7, BMP5, MCIDAS, PODXL, UST, INPP5J, STAP1, KIF1A, CTNNA2, TRIM67 286 676 19554 1.6182397484172633 1.0 0.9125417468047239 0.8968888289767881

GOTERM_BP_ALL GO:0042692~muscle cell differentiation 9 2.710843373493976 0.0701850653316414 POPDC3, FBXO40, UCHL1, IGFBP5, DNER, WFIKKN2, NRAP, MYBPH, B4GALNT2 286 297 19554 2.0718372536554357 1.0 0.9125417468047239 0.8968888289767881

GOTERM_BP_ALL GO:0016477~cell migration 21 6.325301204819277 0.07041492547609392 CCBE1, CALCA, CCL11, ONECUT2, WNT5A, FOXJ1, NDNF, SEMA3E, L1CAM, FOXN1, VTN, HOXB9, WNT11, PODXL, DNER, ADORA1, DRD1, CTNNA2, ADTRP, PRSS3, CDH17 286 962 19554 1.4924981463443001 1.0 0.9125417468047239 0.8968888289767881

GOTERM_BP_ALL GO:0001964~startle response 3 0.9036144578313252 0.07050423898544568 GLRB, DRD1, CTNNA2 286 30 19554 6.837062937062938 1.0 0.9125417468047239 0.8968888289767881

GOTERM_BP_ALL GO:0008217~regulation of blood pressure 7 2.108433734939759 0.07052487963701717 SCNN1G, HSD11B2, SCPEP1, CALCA, KLK1, GCGR, ADORA1 286 198 19554 2.417143462598008 1.0 0.9125417468047239 0.8968888289767881

GOTERM_BP_ALL GO:0060028~convergent extension involved in axis elongation 2 0.6024096385542169 0.07078856868454463 WNT11, WNT5A 286 5 19554 27.348251748251744 1.0 0.9125417468047239 0.8968888289767881

GOTERM_BP_ALL GO:0042396~phosphagen biosynthetic process 2 0.6024096385542169 0.07078856868454463 CKMT1A, CKMT1B 286 5 19554 27.348251748251744 1.0 0.9125417468047239 0.8968888289767881

GOTERM_BP_ALL GO:1904557~L-alanine transmembrane transport 2 0.6024096385542169 0.07078856868454463 SLC7A8, SLC38A4 286 5 19554 27.348251748251744 1.0 0.9125417468047239 0.8968888289767881

GOTERM_BP_ALL GO:0006603~phosphocreatine metabolic process 2 0.6024096385542169 0.07078856868454463 CKMT1A, CKMT1B 286 5 19554 27.348251748251744 1.0 0.9125417468047239 0.8968888289767881

GOTERM_BP_ALL GO:0006599~phosphagen metabolic process 2 0.6024096385542169 0.07078856868454463 CKMT1A, CKMT1B 286 5 19554 27.348251748251744 1.0 0.9125417468047239 0.8968888289767881

GOTERM_BP_ALL GO:0046314~phosphocreatine biosynthetic process 2 0.6024096385542169 0.07078856868454463 CKMT1A, CKMT1B 286 5 19554 27.348251748251744 1.0 0.9125417468047239 0.8968888289767881

GOTERM_BP_ALL GO:0048818~positive regulation of hair follicle maturation 2 0.6024096385542169 0.07078856868454463 GAL, WNT5A 286 5 19554 27.348251748251744 1.0 0.9125417468047239 0.8968888289767881

GOTERM_BP_ALL GO:0006066~alcohol metabolic process 10 3.0120481927710845 0.07135525092573791 HSD11B2, ALDH1A3, ADH4, STK33, DGAT2, CYP11A1, INPP5J, HAO1, NR0B2, CES1 286 350 19554 1.9534465534465537 1.0 0.9143711163598841 0.8986868191770596

GOTERM_BP_ALL GO:0019218~regulation of steroid metabolic process 5 1.5060240963855422 0.07139866628002167 GPRC6A, GAL, MALRD1, CES1, BMP5 286 107 19554 3.1948892229266064 1.0 0.9143711163598841 0.8986868191770596

GOTERM_BP_ALL GO:0019233~sensory perception of pain 4 1.2048192771084338 0.0717702811199061 CALCA, ADORA1, GRM1, PTGES 286 66 19554 4.143674507310871 1.0 0.9161265295893898 0.9004121216317632

GOTERM_BP_ALL GO:0150063~visual system development 11 3.313253012048193 0.07270852440206138 ALDH1A3, BFSP2, TENM3, ANGPTL7, FGF9, SMOC1, WNT5A, PROM1, WNT2, CHRDL1, BMP7 286 404 19554 1.8615765422696118 1.0 0.9226220005787178 0.9067961751719655

GOTERM_BP_ALL GO:0007166~cell surface receptor signaling pathway 41 12.349397590361445 0.07275155560118922 PIGR, DOCK3, CCL11, ONECUT2, GRIK5, WFIKKN2, IL20RA, SEMA3E, VTCN1, CHRDL1, GRM1, WNT11, FGF9, DNER, ADORA1, STAP1, IL13RA2, WNT2, SNCA, CR2, CHRNB4, CR1, IGFBP5, GCGR, WNT5A, G0S2, VWDE, FOS, BMP7, NR0B2, PRLR, BMP5, PSG9, PLPP4, ADGRF1, GLRB, CLNK, TEK, NOTUM, CDH17, MUC20 286 2175 19554 1.2888256571015193 1.0 0.9226220005787178 0.9067961751719655

GOTERM_MF_ALL GO:0004528~phosphodiesterase I activity 2 0.6024096385542169 0.07299739605048826 ENPP1, ENPP3 289 5 19144 26.49688581314879 1.0 0.6950329835232518 0.6602399140262595

GOTERM_MF_ALL GO:0005298~proline:sodium symporter activity 2 0.6024096385542169 0.07299739605048826 SLC6A15, SLC6A20 289 5 19144 26.49688581314879 1.0 0.6950329835232518 0.6602399140262595

GOTERM_MF_ALL GO:0004185~serine-type carboxypeptidase activity 2 0.6024096385542169 0.07299739605048826 SCPEP1, CPVL 289 5 19144 26.49688581314879 1.0 0.6950329835232518 0.6602399140262595

GOTERM_BP_ALL GO:0090132~epithelium migration 5 1.5060240963855422 0.07333717217047947 CCBE1, CALCA, WNT5A, ADTRP, PRSS3 286 108 19554 3.1653069153069153 1.0 0.9240483693480412 0.9081980772982926

GOTERM_BP_ALL GO:0030148~sphingolipid biosynthetic process 5 1.5060240963855422 0.07333717217047947 FA2H, HACD1, ST8SIA2, ST6GALNAC3, ST6GALNAC5 286 108 19554 3.1653069153069153 1.0 0.9240483693480412 0.9081980772982926

GOTERM_CC_ALL GO:0005737~cytoplasm 197 59.337349397590366 0.07341021915966489 EHF, PLEKHB1, AQP6, AQP4, C10ORF71, BPIFA2, PPP4R4, CKMT1B, PRSS3, PTGDS, CHIA, UNC93A, SLPI, PADI2, ATP6V0D2, CCNO, CES1, PTGES, CFAP61, HACD1, KLK1, CRACD, MTTP, HSD11B1, ADH4, HSD11B2, INPP5J, ARSI, HAO1, PROM1, B4GALNT2, SPTBN2, AFAP1L2, TMEM86A, ST8SIA2, NR0B2, SNAP91, FA2H, CPEB1, PPP1R1A, GJB3, HRK, GABRB2, PIGR, GRIK5, HEPACAM2, KRT23, TFCP2L1, CHRDL1, SERPINA4, M1AP, EPB41L4B, PANX2, UCHL1, NMRK2, DNER, NEFM, KIF1A, ANKRD2, AHSG, SLC2A12, FOS, SYN3, PLAAT5, ANO5, DUSP9, F5, GAL, VSNL1, RGS7BP, PNCK, FAXC, MCCD1, HOXD10, CST5, C10ORF90, SCEL, NRAP, CTNNA2, ATP6V1G3, BIK, ATRNL1, GCGR, CKMT1A, MOCOS, MYBPH, G0S2, KLK15, GPR143, KCNS1, FABP7, NOTUM, RAB3B, KCNG3, DGKB, RIMS2, ARHGAP40, FGF9, ENPP1, SLC16A7, ENPP3, MALRD1, GYG2, SH3GL2, KRT6A, RGS7, CHRNB4, DGAT2, IGFBP5, SLC30A2, DIO1, WNT5A, MAT1A, PRLR, SLC9A3, PCP4, C1ORF116, SLC9A4, DOK7, CLIP4, PKP3, RHCG, PVALB, KANK4, PFKFB2, CALCA, GMPR, KNG1, FBXO40, VTN, PCSK1N, KLC3, STK33, SCIN, CYP11A1, PODXL, SOHLH2, STAP1, UGT3A2, DRD1, WNT2, CD300LG, SYT3, KY, BFSP2, UST, LCN2, REEP6, MUC20, DOCK3, ST6GALNAC2, BNC1, DUSP15, SCPEP1, LRRTM1, CA4, ERC2, TRIM67, PLA2G4F, SLC36A2, CR1, SYNGR3, EPN3, ALDH1A3, ADGRF1, NAT8L, ST6GALNAC3, ST6GALNAC5, COLEC12, HCN4, CAMK2B, TRIM50, PPM1H, TTR, WNT11, SV2B, MAN1C1, ZPLD1, IGF2BP2, MAPK4, SNCA, COL26A1, MCOLN3, KCNIP1, SPAG6, KCNIP3, LGI3, EYA4, TPD52L1, MYO16, NFASC, GLRB, GLB1, PSAT1, CLNK, THRSP, TEK 299 12799 20791 1.0702725259942707 0.9999999999999983 0.5004755807982757 0.46007844871590364

GOTERM_MF_ALL GO:0042802~identical protein binding 43 12.951807228915662 0.07373442318493634 HCN4, CAMK2B, TRIM50, CALCA, TENM3, ITLN1, PPM1H, AQP4, M1AP, HSD11B1, VTN, TTR, MCIDAS, SOHLH2, SLC16A7, ENPP1, NCAM2, KIF1A, MAPK4, SH3GL2, RBM11, SNCA, CR2, DGAT2, DMRT2, SLC30A2, MAT1A, FOS, TPD52L1, NR0B2, SLC9A3, ALDH1A3, PLPP4, ANGPTL7, PSAT1, GLB1, LCN2, PADI2, THRSP, TEK, RHCG, MUC20, HCN2 289 2230 19144 1.2773162443558272 1.0 0.6950329835232518 0.6602399140262595

GOTERM_CC_ALL GO:0060076~excitatory synapse 4 1.2048192771084338 0.07406140881768206 IGSF11, LRRTM1, SLC16A7, SPTBN2 299 68 20791 4.090301003344481 0.9999999999999988 0.5004755807982757 0.46007844871590364

GOTERM_BP_ALL GO:0007188~adenylate cyclase-modulating G protein-coupled receptor signaling pathway 8 2.4096385542168677 0.07422334964921834 RIMS2, CALCA, ADGRF1, GCGR, PTGER3, ADORA1, DRD1, GRM1 286 250 19554 2.1878601398601396 1.0 0.9322070859480606 0.9162168466345634

GOTERM_MF_ALL GO:0022835~transmitter-gated channel activity 4 1.2048192771084338 0.07455657749355502 GABRB2, CHRNB4, GLRB, GRIK5 289 65 19144 4.07644397125366 1.0 0.6950329835232518 0.6602399140262595

GOTERM_MF_ALL GO:0022824~transmitter-gated monoatomic ion channel activity 4 1.2048192771084338 0.07455657749355502 GABRB2, CHRNB4, GLRB, GRIK5 289 65 19144 4.07644397125366 1.0 0.6950329835232518 0.6602399140262595

GOTERM_BP_ALL GO:0072010~glomerular epithelium development 3 0.9036144578313252 0.07466405883301254 PODXL, FOXJ1, PROM1 286 31 19554 6.616512519738326 1.0 0.934736582697907 0.9187029546792792

GOTERM_BP_ALL GO:0002695~negative regulation of leukocyte activation 7 2.108433734939759 0.07588196639848771 CR1, GAL, DAPL1, CLNK, FOXJ1, ENPP3, VTCN1 286 202 19554 2.369279235615869 1.0 0.9469487563977411 0.9307056517693109

GOTERM_BP_ALL GO:0072359~circulatory system development 21 6.325301204819277 0.07656004555071051 HCN4, CCBE1, POPDC3, CR2, CALCA, SLC2A12, WNT5A, FOXJ1, NDNF, VWDE, SEMA3E, FOXN1, BMP7, BMP5, WNT11, EMCN, FGF9, NRAP, TEK, ADTRP, WNT2 286 972 19554 1.4771432271432272 1.0 0.95236795516266 0.936031894487827

GOTERM_BP_ALL GO:0060291~long-term synaptic potentiation 4 1.2048192771084338 0.07699077501175054 SLC24A2, LRRTM1, DRD1, SNCA 286 68 19554 4.02180172768408 1.0 0.9523954888480648 0.9360589558852332

GOTERM_BP_ALL GO:0006664~glycolipid metabolic process 5 1.5060240963855422 0.07729374551071085 FA2H, GLB1, ST8SIA2, ST6GALNAC3, ST6GALNAC5 286 110 19554 3.1077558804831535 1.0 0.9523954888480648 0.9360589558852332

GOTERM_BP_ALL GO:0002065~columnar/cuboidal epithelial cell differentiation 5 1.5060240963855422 0.07729374551071085 SLC9A4, WNT11, WNT5A, BMP7, BMP5 286 110 19554 3.1077558804831535 1.0 0.9523954888480648 0.9360589558852332

GOTERM_BP_ALL GO:0048880~sensory system development 11 3.313253012048193 0.07784870840422091 ALDH1A3, BFSP2, TENM3, ANGPTL7, FGF9, SMOC1, WNT5A, PROM1, WNT2, CHRDL1, BMP7 286 410 19554 1.8343339587242027 1.0 0.9562171541726002 0.9398150678107046

GOTERM_BP_ALL GO:0048738~cardiac muscle tissue development 7 2.108433734939759 0.07873106817036268 HCN4, FGF9, WNT5A, NRAP, WNT2, BMP7, BMP5 286 204 19554 2.3460510078157135 1.0 0.9610111008544896 0.9445267834563198

GOTERM_BP_ALL GO:0001667~ameboidal-type cell migration 7 2.108433734939759 0.07873106817036268 CCBE1, CALCA, WNT11, WNT5A, SEMA3E, ADTRP, PRSS3 286 204 19554 2.3460510078157135 1.0 0.9610111008544896 0.9445267834563198

GOTERM_MF_ALL GO:0004683~calmodulin-dependent protein kinase activity 3 0.9036144578313252 0.07885152186880164 CAMK2B, STK33, PNCK 289 31 19144 6.410536890277933 1.0 0.7269937016255447 0.6906006915322518

GOTERM_BP_ALL GO:1903509~liposaccharide metabolic process 5 1.5060240963855422 0.07931148811575053 FA2H, GLB1, ST8SIA2, ST6GALNAC3, ST6GALNAC5 286 111 19554 3.0797580797580797 1.0 0.9638921851980727 0.9473584482783926

GOTERM_BP_ALL GO:0070887~cellular response to chemical stimulus 39 11.74698795180723 0.07946064609159739 COLEC12, HCN4, GABRB2, CALCA, CCL11, NDNF, ADH4, UCHL1, STK33, WNT11, CYP11A1, UGT3A2, ENPP1, STAP1, DRD1, WNT2, RBM11, SNCA, PLA2G4F, SYT3, CHRNB4, DGAT2, KCNJ10, IGFBP5, GCGR, WNT5A, FOS, BMP7, PRLR, SSTR5, SCNN1G, CPEB1, HOXB9, GJB3, PADI2, ADTRP, HCN2, PTGES, CES1 286 2067 19554 1.290011874917535 1.0 0.9638921851980727 0.9473584482783926

GOTERM_BP_ALL GO:0048870~cell motility 24 7.228915662650602 0.07989302088013375 CFAP61, CCBE1, CALCA, CCL11, ONECUT2, SPAG6, WNT5A, FOXJ1, NDNF, SEMA3E, L1CAM, FOXN1, VTN, KLC3, HOXB9, WNT11, PODXL, DNER, ADORA1, DRD1, CTNNA2, ADTRP, PRSS3, CDH17 286 1153 19554 1.4231527362490068 1.0 0.9661366549777165 0.9495644184484008

GOTERM_MF_ALL GO:0061134~peptidase regulator activity 8 2.4096385542168677 0.08137003623933556 PCSK1N, SLPI, AHSG, WFIKKN2, CST6, CST5, SERPINA4, KNG1 289 247 19144 2.1454968269756107 1.0 0.7420593522261145 0.7049121617690265

GOTERM_BP_ALL GO:0120254~olefinic compound metabolic process 6 1.8072289156626504 0.0818131467435029 HSD11B2, ALDH1A3, ADH4, DGAT2, CYP11A1, PTGDS 286 158 19554 2.5963530140745332 1.0 0.9863029357411183 0.9693847850256408

GOTERM_BP_ALL GO:0071300~cellular response to retinoic acid 4 1.2048192771084338 0.08237323676982175 WNT11, GJB3, WNT5A, WNT2 286 70 19554 3.906893106893107 1.0 0.9870805520717435 0.9701490628272973

GOTERM_BP_ALL GO:0043436~oxoacid metabolic process 19 5.72289156626506 0.0823830670699919 PFKFB2, HACD1, PLA2G4F, MOCOS, MAT1A, SCPEP1, FA2H, ALDH1A3, ADH4, PSAT1, UST, ENPP1, HAO1, ADTRP, PTGDS, NAT8L, PTGES, CES1, SNCA 286 864 19554 1.5035207847707845 1.0 0.9870805520717435 0.9701490628272973

GOTERM_BP_ALL GO:0090257~regulation of muscle system process 8 2.4096385542168677 0.0830613389073585 CAMK2B, HCN4, CHRNB4, CALCA, IGFBP5, MYBPH, ADORA1, UNC93A 286 257 19554 2.128268618540992 1.0 0.990450311897005 0.9734610208327196

GOTERM_MF_ALL GO:0005217~intracellularly ligand-gated monoatomic ion channel activity 3 0.9036144578313252 0.08330102250879895 HCN4, MCOLN3, HCN2 289 32 19144 6.210207612456747 1.0 0.7435059349455565 0.7062863291437528

GOTERM_MF_ALL GO:0033612~receptor serine/threonine kinase binding 3 0.9036144578313252 0.08330102250879895 VWDE, BMP7, BMP5 289 32 19144 6.210207612456747 1.0 0.7435059349455565 0.7062863291437528

GOTERM_BP_ALL GO:0031344~regulation of cell projection organization 16 4.819277108433735 0.08333813811168049 CAMK2B, TENM3, WNT5A, NDNF, SEMA3E, L1CAM, BMP7, BMP5, MCIDAS, PODXL, UST, INPP5J, STAP1, KIF1A, CTNNA2, TRIM67 286 693 19554 1.5785426694517604 1.0 0.990450311897005 0.9734610208327196

GOTERM_BP_ALL GO:0099175~regulation of postsynapse organization 5 1.5060240963855422 0.0834250262708947 CAMK2B, DGKB, LRRTM1, WNT5A, KIF1A 286 113 19554 3.02524908719599 1.0 0.990450311897005 0.9734610208327196

GOTERM_BP_ALL GO:0003402~planar cell polarity pathway involved in axis elongation 2 0.6024096385542169 0.08433531061239613 WNT11, WNT5A 286 6 19554 22.79020979020979 1.0 0.9922100097952388 0.9751905344608095

GOTERM_BP_ALL GO:0097272~ammonium homeostasis 2 0.6024096385542169 0.08433531061239613 RHCG, RHBG 286 6 19554 22.79020979020979 1.0 0.9922100097952388 0.9751905344608095

GOTERM_BP_ALL GO:0060685~regulation of prostatic bud formation 2 0.6024096385542169 0.08433531061239613 WNT5A, BMP7 286 6 19554 22.79020979020979 1.0 0.9922100097952388 0.9751905344608095

GOTERM_BP_ALL GO:0034329~cell junction assembly 9 2.710843373493976 0.08499607369953542 GABRB2, ADGRF1, WNT11, DNER, WNT5A, PKP3, DRD1, CDH17, SPTBN2 286 311 19554 1.9785712679603356 1.0 0.9925178267567327 0.9754930714078589

GOTERM_BP_ALL GO:0050922~negative regulation of chemotaxis 4 1.2048192771084338 0.08512377674436904 WNT5A, STAP1, PADI2, SEMA3E 286 71 19554 3.851866443415739 1.0 0.9925178267567327 0.9754930714078589

GOTERM_BP_ALL GO:0009311~oligosaccharide metabolic process 4 1.2048192771084338 0.08512377674436904 ST8SIA2, ST6GALNAC3, B4GALNT2, ST6GALNAC5 286 71 19554 3.851866443415739 1.0 0.9925178267567327 0.9754930714078589

GOTERM_MF_ALL GO:0032795~heterotrimeric G-protein binding 2 0.6024096385542169 0.08694675337991935 ADORA1, DRD1 289 6 19144 22.080738177623992 1.0 0.744665270141079 0.7073876284892013

GOTERM_MF_ALL GO:0070324~thyroid hormone binding 2 0.6024096385542169 0.08694675337991935 ALDH1A3, TTR 289 6 19144 22.080738177623992 1.0 0.744665270141079 0.7073876284892013

GOTERM_MF_ALL GO:0004111~creatine kinase activity 2 0.6024096385542169 0.08694675337991935 CKMT1A, CKMT1B 289 6 19144 22.080738177623992 1.0 0.744665270141079 0.7073876284892013

GOTERM_MF_ALL GO:0099106~ion channel regulator activity 6 1.8072289156626504 0.0869811638543811 DPP10, KCNIP1, KCNS1, KCNIP3, UNC93A, LRRC52 289 156 19144 2.547777482033537 1.0 0.744665270141079 0.7073876284892013

GOTERM_BP_ALL GO:0051241~negative regulation of multicellular organismal process 26 7.83132530120482 0.08736449331873299 CALCA, CCL11, DAPL1, PTGER3, SEMA3E, VTCN1, KNG1, VTN, WNT11, ADORA1, ENPP1, ENPP3, PTGDS, CR1, IGFBP5, AHSG, WNT5A, FOXJ1, BMP7, BMP5, PSG9, GAL, ANGPTL7, CLNK, ADTRP, TEK 286 1287 19554 1.3812248357702903 1.0 1.0 0.9830985915492958

GOTERM_BP_ALL GO:0014706~striated muscle tissue development 7 2.108433734939759 0.08756229924800106 HCN4, FGF9, WNT5A, NRAP, WNT2, BMP7, BMP5 286 210 19554 2.279020979020979 1.0 1.0 0.9830985915492958

GOTERM_BP_ALL GO:0061384~heart trabecula morphogenesis 3 0.9036144578313252 0.08758933471120676 TEK, BMP7, BMP5 286 34 19554 6.032702591526121 1.0 1.0 0.9830985915492958

GOTERM_BP_ALL GO:0090130~tissue migration 5 1.5060240963855422 0.08764141987686452 CCBE1, CALCA, WNT5A, ADTRP, PRSS3 286 115 19554 2.9726360595925816 1.0 1.0 0.9830985915492958

GOTERM_BP_ALL GO:0044057~regulation of system process 14 4.216867469879518 0.08765145286296933 HCN4, CAMK2B, CHRNB4, CALCA, IGFBP5, PTGER3, MYBPH, UNC93A, SLC4A3, GRM1, RIMS2, IGSF11, GAL, ADORA1 286 585 19554 1.636220190066344 1.0 1.0 0.9830985915492958

GOTERM_BP_ALL GO:0022408~negative regulation of cell-cell adhesion 7 2.108433734939759 0.08910331089726092 CR1, DAPL1, PODXL, FOXJ1, VTCN1, ADTRP, B4GALNT2 286 211 19554 2.2682199317270406 1.0 1.0 0.9830985915492958

GOTERM_BP_ALL GO:0006082~organic acid metabolic process 19 5.72289156626506 0.08950126866049511 PFKFB2, HACD1, PLA2G4F, MOCOS, MAT1A, SCPEP1, FA2H, ALDH1A3, ADH4, PSAT1, UST, ENPP1, HAO1, ADTRP, PTGDS, NAT8L, PTGES, CES1, SNCA 286 874 19554 1.4863180297962906 1.0 1.0 0.9830985915492958

GOTERM_CC_ALL GO:0098588~bounding membrane of organelle 42 12.650602409638553 0.08957626480715258 COLEC12, RAB3B, CAMK2B, PIGR, ST6GALNAC2, HEPACAM2, AQP4, PANX2, SV2B, CA4, MAN1C1, ENPP1, DRD1, KIF1A, PROM1, ATP6V1G3, B4GALNT2, CD300LG, SH3GL2, SNCA, SYT3, MCOLN3, CHRNB4, CR1, SLC36A2, PDE6G, SLC30A2, ST8SIA2, WNT5A, SYN3, SYNGR3, F5, EPN3, SLC9A3, GPR143, NFASC, SLC9A4, UST, REEP6, ST6GALNAC3, ATP6V0D2, ST6GALNAC5 299 2314 20791 1.2620894193552117 1.0 0.5962837925968665 0.5481532622527248

GOTERM_BP_ALL GO:0044281~small molecule metabolic process 33 9.939759036144578 0.08987844589263562 PFKFB2, HACD1, GMPR, SCPEP1, HSD11B2, ADH4, STK33, TTR, NMRK2, CYP11A1, CA4, INPP5J, ENPP1, CA9, HAO1, ENPP3, PTGDS, B4GALNT2, SNCA, PLA2G4F, DGAT2, MOCOS, MAT1A, NR0B2, ALDH1A3, FA2H, PSAT1, GLB1, UST, ADTRP, NAT8L, PTGES, CES1 286 1718 19554 1.3132891555475956 1.0 1.0 0.9830985915492958

GOTERM_BP_ALL GO:0061245~establishment or maintenance of bipolar cell polarity 4 1.2048192771084338 0.09074054274208193 WNT11, WNT5A, FOXJ1, VWDE 286 73 19554 3.746335855924897 1.0 1.0 0.9830985915492958

GOTERM_BP_ALL GO:0035088~establishment or maintenance of apical/basal cell polarity 4 1.2048192771084338 0.09074054274208193 WNT11, WNT5A, FOXJ1, VWDE 286 73 19554 3.746335855924897 1.0 1.0 0.9830985915492958

GOTERM_CC_ALL GO:0099572~postsynaptic specialization 10 3.0120481927710845 0.09160379864044033 IGSF11, GABRB2, CPEB1, GLRB, GRIK5, LRRTM1, SLC16A7, RGS7BP, SYN3, GRM1 299 375 20791 1.8542697881828314 1.0 0.6008131499064174 0.5523170212144196

GOTERM_BP_ALL GO:0001659~temperature homeostasis 3 0.9036144578313252 0.09203630548244161 ADORA1, DRD1, IGF2BP2 286 35 19554 5.860339660339661 1.0 1.0 0.9830985915492958

GOTERM_BP_ALL GO:0050891~multicellular organismal-level water homeostasis 3 0.9036144578313252 0.09203630548244161 SCNN1G, AQP6, AQP4 286 35 19554 5.860339660339661 1.0 1.0 0.9830985915492958

GOTERM_BP_ALL GO:0048048~embryonic eye morphogenesis 3 0.9036144578313252 0.09203630548244161 ALDH1A3, WNT5A, BMP7 286 35 19554 5.860339660339661 1.0 1.0 0.9830985915492958

GOTERM_BP_ALL GO:0071560~cellular response to transforming growth factor beta stimulus 6 1.8072289156626504 0.09233950706492983 PSG9, ONECUT2, WNT5A, WFIKKN2, FOS, WNT2 286 164 19554 2.5013644891693674 1.0 1.0 0.9830985915492958

GOTERM_BP_ALL GO:0009725~response to hormone 18 5.421686746987952 0.09306353384132862 IGFBP5, GCGR, FOS, BMP7, PRLR, SSTR5, CPN1, SCNN1G, HSD11B2, CPEB1, GAL, GLB1, CYP11A1, PADI2, ENPP1, TEK, ADTRP, PTGDS 286 822 19554 1.4971670665101322 1.0 1.0 0.9830985915492958

GOTERM_BP_ALL GO:0043542~endothelial cell migration 4 1.2048192771084338 0.09360557328251082 CCBE1, CALCA, ADTRP, PRSS3 286 74 19554 3.695709695709696 1.0 1.0 0.9830985915492958

GOTERM_BP_ALL GO:0030858~positive regulation of epithelial cell differentiation 4 1.2048192771084338 0.09360557328251082 FOXJ1, PROM1, FOXN1, BMP7 286 74 19554 3.695709695709696 1.0 1.0 0.9830985915492958

GOTERM_CC_ALL GO:0005911~cell-cell junction 13 3.91566265060241 0.09378345565129009 IGSF11, EPB41L4B, PANX2, NFASC, PODXL, GJB3, NRAP, TEK, PKP3, CTNNA2, CDH17, TJP3, SPTBN2 299 545 20791 1.6586358197048263 1.0 0.6061945104416723 0.557264011840999

GOTERM_BP_ALL GO:0007162~negative regulation of cell adhesion 9 2.710843373493976 0.09507003103302844 CR1, DAPL1, PODXL, FOXJ1, SEMA3E, VTCN1, ADTRP, B4GALNT2, KNG1 286 318 19554 1.9350178123763033 1.0 1.0 0.9830985915492958

GOTERM_BP_ALL GO:0042445~hormone metabolic process 7 2.108433734939759 0.09535219541094894 ALDH1A3, SCPEP1, ADH4, PCSK1N, DGAT2, CYP11A1, DIO1 286 215 19554 2.2260204911367705 1.0 1.0 0.9830985915492958

GOTERM_BP_ALL GO:2000026~regulation of multicellular organismal development 30 9.036144578313253 0.09591864537348077 CAMK2B, CALCA, CCL11, SEMA3E, SCIN, WNT11, FGF9, LRRTM1, ENPP1, PROM1, WNT2, CCBE1, CR1, AHSG, ST8SIA2, WNT5A, FOXJ1, FOS, L1CAM, FOXN1, BMP7, PRLR, PSG9, GAL, ANGPTL7, ESRP1, LRRN1, TEK, NOTUM, PKP3 286 1541 19554 1.3310310714593647 1.0 1.0 0.9830985915492958

GOTERM_BP_ALL GO:0006665~sphingolipid metabolic process 6 1.8072289156626504 0.09599401108049907 FA2H, HACD1, GLB1, ST8SIA2, ST6GALNAC3, ST6GALNAC5 286 166 19554 2.47122756761311 1.0 1.0 0.9830985915492958

GOTERM_BP_ALL GO:0014855~striated muscle cell proliferation 3 0.9036144578313252 0.09654769791722528 FGF9, FOS, WNT2 286 36 19554 5.6975524475524475 1.0 1.0 0.9830985915492958

GOTERM_BP_ALL GO:0009913~epidermal cell differentiation 7 2.108433734939759 0.09674461307851827 FA2H, SCEL, ST14, MCOLN3, WNT5A, FOXN1, KRT6A 286 216 19554 2.2157148407148406 1.0 1.0 0.9830985915492958

GOTERM_BP_ALL GO:0048729~tissue morphogenesis 14 4.216867469879518 0.09693567098715858 ST14, CCL11, IGFBP5, WNT5A, SEMA3E, FOXN1, BMP7, BMP5, ALDH1A3, WNT11, PODXL, CA9, WNT2, KRT6A 286 595 19554 1.6087206910736322 1.0 1.0 0.9830985915492958

GOTERM_BP_ALL GO:0016042~lipid catabolic process 9 2.710843373493976 0.09719275814317468 HSD11B1, PLA2G4F, GLB1, LIPH, ADORA1, HAO1, ADTRP, PLCXD2, CES1 286 320 19554 1.922923951048951 1.0 1.0 0.9830985915492958

GOTERM_CC_ALL GO:0034774~secretory granule lumen 9 2.710843373493976 0.09729303998159178 TTR, SLPI, GLB1, AHSG, LCN2, PADI2, SERPINA4, KNG1, F5 299 325 20791 1.9255878569590943 1.0 0.6198956547398562 0.5698592341778946

GOTERM_MF_ALL GO:0098632~cell-cell adhesion mediator activity 4 1.2048192771084338 0.09748669078706837 NFASC, CNTN3, PKP3, L1CAM 289 73 19144 3.629710385362848 1.0 0.820158514869478 0.7791017119797067

GOTERM_BP_ALL GO:0001842~neural fold formation 2 0.6024096385542169 0.09768524085797402 BMP7, BMP5 286 7 19554 19.534465534465536 1.0 1.0 0.9830985915492958

GOTERM_BP_ALL GO:0060710~chorio-allantoic fusion 2 0.6024096385542169 0.09768524085797402 BMP7, BMP5 286 7 19554 19.534465534465536 1.0 1.0 0.9830985915492958

GOTERM_BP_ALL GO:0021856~hypothalamic tangential migration using cell-axon interactions 2 0.6024096385542169 0.09768524085797402 NDNF, SEMA3E 286 7 19554 19.534465534465536 1.0 1.0 0.9830985915492958

GOTERM_BP_ALL GO:0071321~cellular response to cGMP 2 0.6024096385542169 0.09768524085797402 HCN4, HCN2 286 7 19554 19.534465534465536 1.0 1.0 0.9830985915492958

GOTERM_BP_ALL GO:0021828~gonadotrophin-releasing hormone neuronal migration to the hypothalamus 2 0.6024096385542169 0.09768524085797402 NDNF, SEMA3E 286 7 19554 19.534465534465536 1.0 1.0 0.9830985915492958

GOTERM_BP_ALL GO:0072201~negative regulation of mesenchymal cell proliferation 2 0.6024096385542169 0.09768524085797402 WNT11, WNT5A 286 7 19554 19.534465534465536 1.0 1.0 0.9830985915492958

GOTERM_BP_ALL GO:0002072~optic cup morphogenesis involved in camera-type eye development 2 0.6024096385542169 0.09768524085797402 ALDH1A3, WNT5A 286 7 19554 19.534465534465536 1.0 1.0 0.9830985915492958

GOTERM_BP_ALL GO:0008064~regulation of actin polymerization or depolymerization 6 1.8072289156626504 0.09779337177855135 CCL11, SCIN, ARHGAP40, CRACD, KANK4, SPTBN2 286 167 19554 2.456429797747163 1.0 1.0 0.9830985915492958

GOTERM_BP_ALL GO:0002704~negative regulation of leukocyte mediated immunity 4 1.2048192771084338 0.09944589099933684 CR2, CR1, FOXJ1, IL13RA2 286 76 19554 3.5984541774015457 1.0 1.0 0.9830985915492958

GOTERM_BP_ALL GO:0048592~eye morphogenesis 6 1.8072289156626504 0.0997191400380167 ALDH1A3, TENM3, WNT5A, PROM1, WNT2, BMP7 286 168 19554 2.441808191808192 1.0 1.0 0.9830985915492958

GOTERM_BP_ALL GO:0051259~protein complex oligomerization 8 2.4096385542168677 0.09998412160628412 ALDH1A3, KCNG3, KCNS1, ITLN1, AQP4, MAT1A, TRPM6, SNCA 286 269 19554 2.0333272675280107 1.0 1.0 0.9830985915492958

Category Term Count % PValue Genes List Total Pop Hits Pop Total Fold Enrichment Bonferroni Benjamini FDR

KEGG_PATHWAY hsa04080:Neuroactive ligand-receptor interaction 16 4.819277108433735 7.524170115754755E-4 GABRB2, CHRNB4, CALCA, GCGR, GRIK5, PTGER3, PRLR, KNG1, GRM1, SSTR5, GAL, GLRB, LYPD6B, ADORA1, DRD1, PRSS3 142 368 8840 2.7066748315982854 0.15833091293594626 0.1723034956507839 0.1723034956507839

KEGG_PATHWAY hsa01100:Metabolic pathways 38 11.44578313253012 0.006648675105636503 PFKFB2, HACD1, ST6GALNAC2, HPSE2, DGKB, GMPR, HSD11B1, HSD11B2, ADH4, NMRK2, CYP11A1, CA4, MAN1C1, INPP5J, CKMT1B, ENPP1, CA9, HAO1, ENPP3, GYG2, ATP6V1G3, PTGDS, B4GALNT2, PLA2G4F, CHIA, DGAT2, PDE6G, CKMT1A, MOCOS, MAT1A, ALDH1A3, PSAT1, GLB1, NAT8L, ST6GALNAC3, ATP6V0D2, ST6GALNAC5, PTGES 142 1561 8840 1.515460475859642 0.7829506658915226 0.7612732995953796 0.7612732995953796

KEGG_PATHWAY hsa00604:Glycosphingolipid biosynthesis - ganglio series 3 0.9036144578313252 0.023153746055892793 GLB1, ST6GALNAC3, ST6GALNAC5 142 15 8840 12.450704225352112 0.9953204582736161 1.0 1.0

KEGG_PATHWAY hsa04974:Protein digestion and absorption 6 1.8072289156626504 0.02606465948956429 SLC9A3, COL26A1, SLC15A1, SLC36A2, SLC7A8, PRSS3 142 105 8840 3.557344064386318 0.9976373540904582 1.0 1.0

KEGG_PATHWAY hsa04024:cAMP signaling pathway 9 2.710843373493976 0.028016207715266635 CAMK2B, HCN4, POPDC3, PTGER3, ADORA1, FOS, DRD1, HCN2, SSTR5 142 226 8840 2.479122522747102 0.9985074932374158 1.0 1.0

KEGG_PATHWAY hsa04964:Proximal tubule bicarbonate reclamation 3 0.9036144578313252 0.051368895639894566 SLC9A3, CA4, SLC38A3 142 23 8840 8.120024494794857 0.9999943075981317 1.0 1.0

KEGG_PATHWAY hsa04610:Complement and coagulation cascades 5 1.5060240963855422 0.05177891248289454 VTN, CR2, CR1, KNG1, F5 142 88 8840 3.537131882202305 0.9999948441467499 1.0 1.0

KEGG_PATHWAY hsa04966:Collecting duct acid secretion 3 0.9036144578313252 0.07292196149321349 CLCNKB, ATP6V1G3, ATP6V0D2 142 28 8840 6.670020120724346 0.9999999705129621 1.0 1.0

KEGG_PATHWAY hsa05226:Gastric cancer 6 1.8072289156626504 0.09158847533203282 WNT11, FGF9, WNT5A, CTNNA2, WNT2, CDH17 142 150 8840 2.4901408450704228 0.99999999972028 1.0 1.0
