# Supplementary material for: DNER drives glycolytic reprogramming in renal cell carcinoma by activating the JAK2/STAT3 signaling pathway
Source: Front Immunol. 2026 May 22;17:1799104. doi: 10.3389/fimmu.2026.1799104 (PMC13236898; doi:10.3389/fimmu.2026.1799104)
Supplement: Supplementary file 7 [file Table5.docx]

id HR L95CI H95CI pvalue

MAT1A 1.516775813 1.336256324 1.721682305 1.17E-10

MOCOS 1.74355653 1.444777758 2.10412249 6.77E-09

GYG2 1.811438282 1.461024538 2.245895647 6.08E-08

EMCN 0.675031697 0.581962078 0.782985369 2.08E-07

PSAT1 1.360875632 1.20972722 1.530909162 2.90E-07

DNER 1.377053592 1.210863857 1.566052685 1.08E-06

TENM3 1.668600568 1.355450061 2.054098439 1.38E-06

IGF2BP2 1.492797826 1.264709787 1.762021115 2.18E-06

ADORA1 1.59137856 1.306046635 1.939046933 4.06E-06

TEK 0.659273663 0.550336483 0.789774576 6.15E-06

SMKR1 1.853888594 1.416795212 2.425829006 6.81E-06

CA4 0.676943541 0.570323706 0.803495546 8.12E-06

PKP3 1.39275062 1.202067955 1.613681058 1.03E-05

WNT5A 1.879569637 1.419787247 2.488247467 1.04E-05

L1CAM 1.315673077 1.163204019 1.488127291 1.27E-05

ONECUT2 2.704664252 1.727875783 4.233642714 1.35E-05

PPP1R1A 1.222520457 1.114171457 1.341405991 2.20E-05

MXRA5Y 12.89121113 3.771645801 44.06122235 4.56E-05

ZNF385B 0.617757029 0.48783432 0.782281465 6.38E-05

SLC4A3 1.543988011 1.243966164 1.91636963 8.14E-05

SCNN1G 1.247526479 1.11673831 1.393632064 9.08E-05

HOXD10 1.481746318 1.214618568 1.807622745 0.000105783

HACD1 2.116064529 1.448115065 3.092108632 0.000107392

PANX2 1.490488917 1.215022856 1.828407754 0.00012916

SLC12A8 1.540779425 1.233930914 1.923933673 0.00013613

ASXL3 0.264726251 0.13260728 0.528477684 0.000164491

ARSI 1.490332551 1.208271357 1.838238654 0.000193525

PODXL 0.743884265 0.636077435 0.869962946 0.00021235

HCN4 5.443169899 2.184129017 13.56517785 0.000276088

MCOLN3 0.273971484 0.13562495 0.553440752 0.000307332

HSD11B2 0.776063348 0.676096504 0.890811174 0.000314174

KCNIP3 1.399150892 1.161077771 1.6860397 0.000416573

COLEC12 1.324759009 1.132777457 1.549277329 0.000430327

MUC20 0.735028636 0.617598052 0.874787565 0.000527874

DUSP9 1.521300148 1.199211464 1.929896612 0.000547002

PRR15L 0.746208628 0.630584809 0.883033192 0.000654167

TAGLN3 1.791388123 1.276396888 2.514164237 0.000748624

VTN 1.239640593 1.093378228 1.405468631 0.00079767

UCHL1 1.202775674 1.079692705 1.339889874 0.000802161

CCL11 1.570000489 1.195972822 2.061001296 0.001158299

SLPI 1.116881927 1.043710831 1.195182804 0.001386335

IL13RA2 1.358001071 1.121468834 1.644421007 0.001724154

NDNF 1.168782903 1.056873511 1.292542068 0.002388349

PRLR 0.613775296 0.445062967 0.846442284 0.002914882

POPDC3 1.641212532 1.180962987 2.280832341 0.003172266

MYBPH 2.878450186 1.411447926 5.870195649 0.00364025

TLL2 3.22218037 1.453398152 7.143566491 0.003971229

G0S2 1.189069167 1.056683213 1.338041019 0.004034263

MTTP 1.366183946 1.102987104 1.692185309 0.004266938

PTGES 1.203627962 1.059041801 1.367953814 0.004532488

MCIDAS 3.801525009 1.505259681 9.600730411 0.004725574

ST6GALNAC3 0.616609525 0.438574619 0.86691589 0.005410504

ALDH1A3 1.330021227 1.085068549 1.630271623 0.006030991

ST8SIA2 2.024956332 1.219307557 3.362931792 0.006408996

FABP7 1.095828088 1.026031058 1.170373146 0.006424704

GAL 1.652620265 1.144808356 2.385686411 0.007319371

SYT3 2.044812868 1.192646035 3.505868082 0.00931071

C1orf53 1.292418272 1.060542197 1.574991543 0.01100314

SH3GL2 0.668313626 0.488145596 0.914979273 0.01192591

ENPP1 1.427106514 1.081563769 1.883044773 0.011927867

HSD11B1 1.252138851 1.049839213 1.493420785 0.012386793

SYN3 0.090197756 0.013661144 0.595531026 0.012483324

PTGER3 0.849975979 0.746808272 0.967395774 0.013815033

ZPLD1 1.424484004 1.072338946 1.89226987 0.014605342

SLC6A15 1.546226833 1.0859681 2.201554006 0.015630765

CCNO 1.403420678 1.058456232 1.860813456 0.018538913

F5 0.798796045 0.661270879 0.964922458 0.019786549

CR1 1.747910235 1.089361843 2.804568757 0.020626121

SLC13A3 1.167211228 1.023599122 1.330972273 0.020989978

SERPINA4 1.250250691 1.032113233 1.51449157 0.022426143

CRACD 1.83797317 1.08637108 3.109568576 0.023283188

STK33 0.60681805 0.391918403 0.939553089 0.025123494

PLCXD2 0.663966898 0.462374363 0.953452604 0.026545311

SMCO3 1.316648792 1.028934372 1.684814978 0.028764438

CLIP4 1.341817275 1.029165318 1.749450323 0.029828088

HPSE2 1.715521685 1.052556896 2.796062296 0.030350879

CD300LG 0.631978396 0.416948641 0.957903814 0.0305684

GJB3 1.386964886 1.026360786 1.874264509 0.033226667

UNC93A 1.304508955 1.021326913 1.666208529 0.033256521

PIGR 0.91267819 0.8389444 0.992892352 0.033509404

LINC00189 1.371140029 1.022581678 1.838508375 0.03492902

LINC00488 1.660670548 1.03426977 2.666448106 0.035778736

HOXB9 1.169759494 1.009537762 1.355409699 0.036954479

ATRNL1 0.526453915 0.283560866 0.977404707 0.042117247

SLC7A4 1.666399432 1.015292967 2.735059887 0.04338346

CHRDL1 1.150253293 1.001446418 1.321171672 0.047657191

ANO5 0.70456512 0.496669014 0.999482541 0.049661828

LOC102723370 0.70456512 0.496669014 0.999482541 0.049661828

RERGL 0.797343031 0.635403684 1.000554333 0.050561875

ATP6V0D2 0.814327755 0.662696379 1.000653864 0.050731228

CLCNKB 0.819353793 0.670470785 1.001297376 0.051499722

NCAM2 2.637629568 0.99016746 7.026174884 0.052355735

TMEM63C 1.621754677 0.987371438 2.663727277 0.056163353

NRAP 1.408994516 0.985153761 2.01518344 0.060376105

FNDC10 1.243129493 0.986302671 1.566832354 0.065306023

LINC01806 0.65198864 0.411760115 1.032370964 0.068136773

SORD2P 0.580912406 0.322387563 1.046750131 0.070624457

REEP6 1.267889599 0.979474193 1.64123164 0.071471693

ATP6V1G3 0.784925705 0.600501932 1.025988977 0.076360898

IRX6 1.162890275 0.983694071 1.374730041 0.077158362

PRSS3 1.358910538 0.966555169 1.910535383 0.077685999

SCIN 0.872978322 0.749313075 1.017053054 0.081333048

FBXO40 1.44E-05 4.93E-11 4.180468066 0.082363306

SLC9A3 0.880912122 0.763231201 1.016738003 0.083081329

DUSP15 0.787555732 0.600449301 1.03296653 0.084418336

CR2 1.396949781 0.955471319 2.042414724 0.084540896

CST5 1.267404881 0.965392543 1.663898426 0.087942389

CLNK 0.705156411 0.471719995 1.054111698 0.088558168

DMRT2 0.77689863 0.579885761 1.040845495 0.090704769

ST14 0.876949085 0.752954935 1.021362186 0.091372224

STAP1 0.804437992 0.623123408 1.03851095 0.094924276

PADI2 1.206255747 0.967110907 1.504535744 0.09624649

HEPACAM2 0.759021537 0.547436617 1.052384285 0.098182571

FGF9 0.726918288 0.497605494 1.061905874 0.099076538

SNAP91 1.692641301 0.900359108 3.182102061 0.102244075

BMP5 0.696870072 0.45186962 1.074708004 0.102260848

INPP5J 0.772528801 0.566464438 1.053553776 0.103017316

ARHGAP40 1.283849206 0.94863821 1.737510429 0.105569681

PNCK 1.078396816 0.984070958 1.181764062 0.106065254

ADGRF1 0.732446912 0.501237042 1.070308922 0.107644903

NOTUM 1.452946273 0.920555396 2.293238281 0.108612794

PLA2G4F 0.731932172 0.497518666 1.076793174 0.113117011

SLC38A4 0.808246407 0.620732341 1.052405701 0.113945966

CHRNB4 1.54756646 0.900428917 2.65980124 0.11401925

TMEM61 0.797178573 0.601132352 1.057160999 0.115491516

AQP6 0.631836638 0.356121276 1.121015686 0.116539806

SPAG6 0.347795985 0.091621403 1.320237874 0.120715569

SLC9A2 0.716246895 0.46898912 1.093862505 0.122416897

NALF2 1.263677461 0.937161636 1.703954434 0.124922008

DOK7 1.448400534 0.90019108 2.330465335 0.126847595

CPVL 0.879640151 0.746068979 1.037125008 0.126970397

CNTN3 0.705318176 0.449321073 1.107167589 0.129152908

RHCG 0.883333531 0.752537569 1.036862688 0.129209813

TMEM52B 0.828430378 0.648345955 1.058535008 0.132301848

PART1 0.600258316 0.307126351 1.173165525 0.135477972

PFKFB2 0.786406601 0.571924925 1.08132259 0.139198084

RHBG 0.805806679 0.603220674 1.076429292 0.143891974

GPR143 1.106434516 0.96600087 1.267283888 0.144157472

KLK1 0.876232573 0.732678753 1.047912906 0.147811752

UGT3A2 1.251194128 0.920657782 1.700400276 0.152202693

TRIM50 0.791078391 0.571422873 1.095169707 0.157899383

CTNNA2 0.338340972 0.074390954 1.538824373 0.160840755

KIF1A 1.426764015 0.86175383 2.362223972 0.167098507

AFAP1L2 0.832405899 0.641030341 1.080915421 0.168756133

DRD1 1.683918707 0.800077761 3.544133269 0.169903555

KCNS1 1.141170875 0.942008419 1.382440899 0.177186225

DPP10 0.180285253 0.014902433 2.18103792 0.178012883

M1AP 0.506024997 0.187634062 1.36468451 0.178396599

PTGDS 1.083810796 0.963238025 1.219476194 0.181053159

LINC00871 1.657171857 0.781181763 3.515466814 0.18804402

RAP2CP1 0.549228926 0.223197305 1.351505623 0.192123828

CALCA 0.639996314 0.326148281 1.25585602 0.194428686

GPRC6A 0.635137393 0.319518911 1.262521543 0.195341448

RGS7 0.848724233 0.657315812 1.09587022 0.208436399

FAXC 1.450207644 0.809279054 2.598735504 0.211685536

C10orf71 0.219343026 0.020189183 2.383026743 0.212583289

LINC01014 0.635490107 0.311143963 1.297944758 0.213409048

DIRAS1 0.803435266 0.566608805 1.139248493 0.219335479

KRT6A 1.22918879 0.882069832 1.71290869 0.222917239

RAB3B 1.382546623 0.820293135 2.330185494 0.223905805

MAPK4 1.285370446 0.856662964 1.928619835 0.225262759

LOC285626 0.616430385 0.280899923 1.352746613 0.227622324

GCGR 0.824253101 0.601571288 1.129364363 0.229033724

KRT23 1.233509297 0.872138776 1.744613619 0.235424391

DAPL1 0.795063403 0.544191489 1.161587102 0.235780552

SLC24A2 0.433603164 0.108466048 1.733369175 0.23723223

NR0B2 0.856423305 0.662198762 1.107614391 0.237565995

CPEB1 1.249889955 0.860088676 1.816353295 0.242147776

DGAT2 1.250046544 0.855455713 1.826647877 0.248811497

LINC02048 1.176852526 0.88919239 1.557572786 0.254818316

CPN1 1.252449207 0.849755427 1.84597705 0.255388742

CA9 1.07768073 0.944765264 1.229295571 0.265306859

RBM11 0.670134787 0.33099665 1.356752807 0.266043543

TMPRSS2 0.879463729 0.701170801 1.103092784 0.2665028

LINC01612 0.798309174 0.535814107 1.189400444 0.268153358

LRRC52 0.617501464 0.256827214 1.484687125 0.281470557

NMRK2 0.877033161 0.688827956 1.116660784 0.287037862

SSTR5 1.438184178 0.725764992 2.849922153 0.29769667

ELF5 0.398767871 0.07062424 2.251575587 0.297884615

CKMT1A 0.76729661 0.46474004 1.266824541 0.300468902

ANGPTL7 1.229988341 0.829457186 1.823929367 0.303112951

AQP4 0.829747 0.581616112 1.183736266 0.303238263

SLC9A4 0.698084022 0.349578618 1.394024913 0.30841551

FOXJ1 1.203943036 0.841890565 1.721695067 0.309173378

KLK15 0.835462013 0.589661656 1.183724205 0.31191378

C10orf90 1.651548604 0.623859643 4.372157781 0.31246503

LIPH 1.12124939 0.89591605 1.403256694 0.317411554

LRRTM1 0.751097172 0.427898151 1.318414116 0.318747697

EPN3 0.816307145 0.544450611 1.22390781 0.326002452

SERTM2 0.839006927 0.589839556 1.193430681 0.328877034

WFIKKN2 0.303505778 0.025872856 3.560324259 0.342550716

CDH17 1.127498612 0.879796214 1.444940431 0.343064479

LOC105371730 0.635929726 0.249285436 1.622263306 0.343445165

COL26A1 1.230269088 0.798053923 1.896566114 0.34801723

ESRP1 0.891317397 0.699531898 1.135683313 0.351999178

KLRG2 1.278019563 0.761440913 2.145056791 0.353174053

PCP4 0.920433912 0.772535182 1.096647254 0.353569918

SLC6A20 0.890816397 0.697635171 1.137491177 0.353911135

RGS7BP 0.866331164 0.638242288 1.175932243 0.357357207

KCNG3 0.436481182 0.074125401 2.570182684 0.359438065

ERC2 1.846949229 0.495967723 6.877910192 0.36039788

MALRD1 0.496036328 0.106914274 2.301395596 0.370558557

DIO1 0.866823131 0.633930876 1.185274877 0.370654474

SLC30A2 1.111933759 0.880525551 1.404157644 0.372813823

ISM2 1.200498293 0.802900827 1.794986508 0.373271996

ANKRD2 1.101154943 0.884744477 1.37049989 0.388079402

GLRB 0.901124176 0.710663278 1.142629436 0.390127958

KNG1 0.916254878 0.748300212 1.121906675 0.397241482

KCNIP1 1.129455045 0.851236693 1.498606333 0.398840831

LINC01124 1.321126049 0.680647897 2.564283303 0.410499136

GMPR 0.914175109 0.736609585 1.134544197 0.415435614

PRAMENP 0.56620765 0.143339165 2.236591111 0.417069979

FOS 0.953384696 0.848001971 1.071863522 0.424433507

TJP3 0.882139854 0.648151791 1.200599509 0.425200919

B4GALNT2 0.867950016 0.611845315 1.231254391 0.427284381

SPTBN2 1.072677867 0.899919255 1.278601163 0.433606576

SLC16A7 0.911655232 0.723242163 1.149152116 0.433613654

SOHLH2 1.549874016 0.507311603 4.734978369 0.441902523

SLC38A3 1.123491532 0.833645997 1.514111778 0.444363129

IGSF22 0.692102314 0.263147632 1.820292318 0.455721773

ADH4 0.778783449 0.403164034 1.50435954 0.456699312

SV2B 1.141595934 0.804150032 1.620644439 0.45885148

MYO16 0.587165538 0.139435771 2.472560414 0.467920771

VSNL1 1.196583746 0.736976477 1.942820032 0.467983503

SCPEP1 0.880610995 0.62459483 1.241566033 0.468199078

LRRN1 1.156770521 0.77871807 1.718360071 0.470744212

CKMT1B 0.84614379 0.534692468 1.339011407 0.475605785

TRIM67 0.485181407 0.06465412 3.640928044 0.48185993

ST6GALNAC2 0.880688741 0.617416888 1.256221968 0.483217971

NDUFB4P11 1.375634752 0.561634611 3.369398776 0.485330286

LINC01485 0.818999302 0.467274549 1.435472696 0.485560662

SYT14 1.33211639 0.594013693 2.987362242 0.486466052

PLAAT5 0.872925575 0.594423549 1.281912637 0.488182272

IGFBP5 1.050271522 0.913810651 1.207110323 0.489746513

HCN2 0.903123696 0.676355572 1.205922511 0.489748449

CES1 0.94051811 0.789860985 1.119911392 0.491140109

GRM1 0.720748924 0.283451735 1.832689476 0.49162608

PSG4 0.862812918 0.562439328 1.323602553 0.499134282

XKR4 1.66405862 0.378802814 7.31011226 0.500048862

TMEM40 0.465230087 0.049580938 4.365367863 0.502934754

CCDC185 2.277312383 0.203447973 25.49129201 0.504240708

PVALB 0.963832892 0.86392132 1.07529913 0.509418251

LGI3 1.163657935 0.741022368 1.827339969 0.51037021

DGKB 0.518433279 0.068616966 3.917005949 0.524318279

RIMS2 1.293792138 0.584846359 2.862115953 0.52488387

NFASC 0.917710284 0.702626396 1.198634396 0.528539086

BPIFA2 0.804805655 0.404733509 1.600342273 0.535790192

SLC15A1 0.941875323 0.775617822 1.143770938 0.545629284

LINC01018 0.871000634 0.553459699 1.370726912 0.550531273

SLC36A2 1.081499474 0.835007941 1.400754477 0.552732805

LOC643201 0.733492495 0.260341977 2.066555865 0.557566831

NAT8L 0.899589278 0.629669439 1.285215416 0.560995645

TMEM86A 1.097236088 0.802082094 1.501002254 0.561619406

FA2H 0.926931559 0.714193272 1.203038658 0.568419014

CRTAC1 0.95530254 0.815908751 1.118511037 0.569882014

GABRB2 0.791996979 0.352274086 1.780599937 0.572640232

ENPP3 0.971454444 0.878323059 1.074460845 0.57327784

SLC2A12 0.890305645 0.579212534 1.368485824 0.596298107

TPD52L1 1.064980707 0.83969798 1.350704578 0.603636734

BFSP2 1.312243891 0.464329769 3.708536787 0.608191919

YWHAEP7 0.89985435 0.601025801 1.347259719 0.608340017

KCNK15 0.92111421 0.672150199 1.262294334 0.609272283

EYA4 0.826291155 0.396240439 1.723087819 0.610847958

LINC02608 0.866646632 0.499327137 1.504176979 0.610916795

KANK4 0.861446828 0.481962953 1.539725478 0.614725119

ANKFN1 1.396735936 0.376643856 5.179617946 0.61728898

MCCD1 0.940695972 0.737958871 1.199130395 0.621558197

SYNGR3 0.929682228 0.695477793 1.242755775 0.622461596

SLC7A8 0.95109982 0.778552224 1.16188849 0.623513249

PCSK1N 1.028434365 0.91750783 1.152771899 0.630172022

SOWAHA 1.07464769 0.798560255 1.446187249 0.634647698

ST6GALNAC5 1.089669307 0.754532043 1.573663052 0.646989781

IGSF11 1.123269281 0.682886582 1.847647778 0.647096638

SNCA 0.930362499 0.682572253 1.268106601 0.647818353

THRSP 1.040839071 0.875386994 1.237562335 0.650423478

IGFBPL1 1.248525296 0.46155144 3.377338431 0.661987387

LINC01443 0.879013769 0.488663142 1.581181677 0.666846398

AHSG 0.911144234 0.589806432 1.407553005 0.674952876

PLPP4 0.933161684 0.670849197 1.298042441 0.681205817

VWDE 0.856924758 0.399152069 1.839699945 0.692026308

PSCA 0.932511213 0.659088992 1.31936229 0.693104925

UPK1B 0.967088774 0.817806033 1.143621664 0.695651288

TFCP2L1 0.972052849 0.840083477 1.124753393 0.703385884

PROM1 0.981861067 0.890986119 1.082004685 0.711817658

PLEKHB1 1.045578356 0.816771584 1.338482042 0.723551327

WNT2 1.080418327 0.694347429 1.681152279 0.731685383

CST6 1.044544622 0.812186547 1.343377911 0.734242342

HRK 1.079237389 0.687031594 1.695341743 0.740699701

CCBE1 1.047931666 0.793756672 1.38349801 0.74115674

KCNK13 1.073425314 0.701632737 1.642229395 0.743966024

UST 1.039705244 0.822617708 1.314081844 0.744533856

LINC02303 1.048277442 0.787069057 1.396174306 0.747115039

CYP11A1 0.953150873 0.705604302 1.287544001 0.754487294

CFAP61 1.107196848 0.579386285 2.115833412 0.757941509

EPB41L4B 0.940451954 0.633780484 1.395514536 0.760441855

LCN2 1.023722904 0.880091372 1.190795203 0.761149231

HAO1 1.086859435 0.634894857 1.860565446 0.76137863

CHIA 1.2645354 0.269330571 5.937126906 0.766122786

UCA1 0.923629706 0.537594431 1.586868806 0.773573198

GLB1 1.0453782 0.76572143 1.427171213 0.779939971

LYPD6B 0.946849788 0.643913361 1.392306133 0.781305296

CCDC187 0.798013949 0.156666144 4.064862046 0.785902268

BNC1 0.937257868 0.578479334 1.518554352 0.792411109

NEFM 0.959551695 0.701408642 1.312700472 0.796224083

BMP7 1.046577263 0.73612425 1.487960717 0.799825671

PSG9 0.915062395 0.444824036 1.882405443 0.809409221

KLC3 0.947105537 0.59692207 1.502723628 0.817519091

SMOC1 1.01819404 0.870552767 1.190874514 0.821526267

WNT11 0.960693419 0.665535107 1.386751557 0.830456215

CAMK2B 0.958501205 0.649250166 1.415054795 0.831132177

ADTRP 1.027028714 0.802347583 1.314627229 0.832319481

KCNJ10 0.95494486 0.61301446 1.48759898 0.838474168

EHF 1.028368015 0.768508669 1.376094788 0.850698304

PDE6G 1.034393176 0.720031066 1.486004275 0.854842198

FOXN1 0.868918282 0.186339643 4.051843019 0.858047495

SCEL 0.968025461 0.662557196 1.41432815 0.866593074

CCDC169 1.150224133 0.198617784 6.661113253 0.875888116

ITLN1 0.951171263 0.461275919 1.96135704 0.892154299

SLC7A10 0.962602984 0.474002758 1.954850451 0.916020111

VTCN1 1.010550107 0.830032835 1.230326652 0.916750407

KY 1.047029217 0.440872985 2.486589604 0.917060725

DOCK3 0.964326105 0.440144364 2.112772336 0.927671459

BIK 1.011973771 0.730704863 1.401511014 0.942890051

TRPM6 0.975462679 0.44439381 2.141180673 0.950615829

MAN1C1 1.007072573 0.779854855 1.300492215 0.956917526

IL20RA 1.013304338 0.561721951 1.827925149 0.964977867

PPM1H 0.994734813 0.78096612 1.267016997 0.965888579

TTR 1.004090223 0.817424665 1.233382377 0.968972251

PPP4R4 1.02429178 0.289926284 3.618760034 0.970268242

GRIK5 1.005571653 0.730083946 1.385011073 0.972865284

C1orf116 0.996663696 0.785669794 1.264320621 0.97803292

SEMA3E 1.002660591 0.625884732 1.606251455 0.991182762

LINC00323 0.999470107 0.724723221 1.378375174 0.997421325
